# Occupation and SARS-CoV-2 seroprevalence studies: a systematic review

**DOI:** 10.1136/bmjopen-2022-063771

**Published:** 2023-02-28

**Authors:** Emily Boucher, Christian Cao, Sean D’Mello, Nathan Duarte, Claire Donnici, Natalie Duarte, Graham Bennett, SeroTracker Consortium, Anil Adisesh, Rahul Arora, David Kodama, Niklas Bobrovitz

**Affiliations:** 1 Cumming School of Medicine, University of Calgary, Calgary, Alberta, Canada; 2 Faculty of Engineering, University of Waterloo, Waterloo, Ontario, Canada; 3 Faculty of Engineering, McGill University, Montreal, Québec, Canada; 4 Faculty of Arts and Science, University of Toronto, Toronto, Ontario, Canada; 5 Department of Economics, McGill University, Montreal, Québec, Canada; 6 St. Michael’s Hospital, Unity Health Toronto, Toronto, Ontario, Canada; 7 Division of Occupational Medicine, University of Toronto, Toronto, Ontario, Canada; 8 Canadian Health Solutions, Saint John, New Brunswick, Canada; 9 Institute of Biomedical Engineering, Oxford University, Oxford, UK; 10 Division of Emergency Medicine, University of Toronto Department of Medicine, Toronto, Ontario, Canada; 11 Temerty Faculty of Medicine, University of Toronto, Toronto, Ontario, Canada; 12 Department of Critical Care Medicine, University of Calgary, Calgary, Alberta, Canada

**Keywords:** COVID-19, public health, occupational & industrial medicine

## Abstract

**Objective:**

To describe and synthesise studies of SARS-CoV-2 seroprevalence by occupation prior to the widespread vaccine roll-out.

**Methods:**

We identified studies of occupational seroprevalence from a living systematic review (PROSPERO CRD42020183634). Electronic databases, grey literature and news media were searched for studies published during January–December 2020. Seroprevalence estimates and a free-text description of the occupation were extracted and classified according to the Standard Occupational Classification (SOC) 2010 system using a machine-learning algorithm. Due to heterogeneity, results were synthesised narratively.

**Results:**

We identified 196 studies including 591 940 participants from 38 countries. Most studies (n=162; 83%) were conducted locally versus regionally or nationally. Sample sizes were generally small (median=220 participants per occupation) and 135 studies (69%) were at a high risk of bias. One or more estimates were available for 21/23 major SOC occupation groups, but over half of the estimates identified (n=359/600) were for healthcare-related occupations. ‘Personal Care and Service Occupations’ (median 22% (IQR 9–28%); n=14) had the highest median seroprevalence.

**Conclusions:**

Many seroprevalence studies covering a broad range of occupations were published in the first year of the pandemic. Results suggest considerable differences in seroprevalence between occupations, although few large, high-quality studies were done. Well-designed studies are required to improve our understanding of the occupational risk of SARS-CoV-2 and should be considered as an element of pandemic preparedness for future respiratory pathogens.

**STRENGTHS AND LIMITATIONS OF THIS STUDY**

- We conducted a comprehensive search of the COVID-19 seroprevalence literature, including non-English articles, government reports, unpublished data.
- Occupations were classified using the Standard Occupational Classification 2010 coding system to improve interpretability and facilitate comparison with other datasets.
- Seroprevalence may underestimate the true prevalence of infection because antibody titres decline over time, but where possible we prioritised prevalence estimates for IgG antibodies, which appear to be more robust than other immunoglobulin types.
- We did not adjust for differences in serological test performance.

## Introduction

Occupation is a social determinant of health and an important risk factor for SARS-CoV-2 infection. Essential workers in health and social care occupations have an increased risk of COVID-19 compared with non-essential workers, but the risks for other occupations are not well defined.^1–3^ Studies using confirmed COVID-19 cases to examine occupational COVID-19 risk are affected by variable testing rates. For example, testing rates may be higher in workplaces offering testing or paid sick leave, and are impacted by geographic (eg, urban vs rural) and socioeconomic factors (eg, deprivation), potentially biasing results.^4–6^ Few high-quality, prospective studies using frequent, serial molecular or antigen testing covering a broad range of occupations have been conducted, in part due to the costs and administrative burden of such studies.^7 8^

Serological testing for SARS-CoV-2 antibodies provides evidence of previous infection and/or vaccination depending on vaccination status and the specific antigens targeted and can be used to obtain more accurate estimates of the cumulative incidence of infection.^9^ Accurate data on the occupational risks of COVID-19 and other respiratory infections are essential for informing the development of occupational safety guidelines and regulations, transmission control measures and resource allocation (testing, personal protective equipment (PPE), etc). The objectives of this review were to describe and synthesise studies of SARS-CoV-2 seroprevalence across a broad range of occupations globally prior to the widespread roll-out of vaccines.

## Methods

We identified seroprevalence studies with sample frames or subgrouping variables related to occupation or employment status from a database compiled via a living systematic review (PROSPERO CRD42020183634). The database has been described previously and includes >1000 cohort and cross-sectional studies reporting antibody testing for SARS-CoV-2 in humans identified from electronic databases, grey literature and news media.^10–12^ We restricted the current review to studies published during January–December 2020 before vaccines were rolled-out, because differential vaccination rates by occupation may obscure results. We excluded studies that only reported seroprevalence for mixed occupation groups or workplaces (eg, ‘hospital staff’) rather than specific occupations, included children <18 years and that could not be machine-translated using Google Translate if unavailable in English or French (online supplemental file 1).

10.1136/bmjopen-2022-063771.supp1 Supplementary data

We extracted study information, sample characteristics, seroprevalence estimates and study-level risk of bias from the living review database. Risk of bias was assessed with a modified Joanna Briggs Institute Checklist for Prevalence Studies by one reviewer and verified independently as described previously. Overall risk of bias was assessed qualitatively based on whether seroprevalence estimates were very likely (corresponding to a low risk of bias), likely (moderate risk) or unlikely (low risk) to be correct for the author’s stated target population (online supplemental file 1).^12 13^ If multiple estimates were reported, the most recent estimate using laboratory-based methods (eg, ELISA) and anti-spike and/or IgG antibodies were prioritised, because non-IgG and anti-nucleocapsid antibodies may decline more rapidly.^14^ Free-text descriptions of occupations were extracted from the original studies by one researcher and reviewed by a second.

For each seroprevalence estimate, we identified the relevant Standard Occupational Classification (SOC) 2010 codes by applying the National Institute for Occupational Safety and National Institute for Health Industry and Occupation Computerised Coding System (NIOCCS) to occupation descriptions.^15^ NIOCCS was chosen, because many studies were conducted in the USA. Coding was manually verified if there was insufficient information for NIOCCS classification, or if the probability of correct classification to the six-digit level was <0.8 based on our review of a subset of the NIOCCS coded data (online supplemental file 1). Anticipating substantial heterogeneity and an insufficient number of estimates relative to covariates for meta-regression, we planned to summarise data using the median/IQR.

### Patient and public involvement

It was not possible or appropriate to involve patients or the public in this study.

## Results

**Figure 1:**
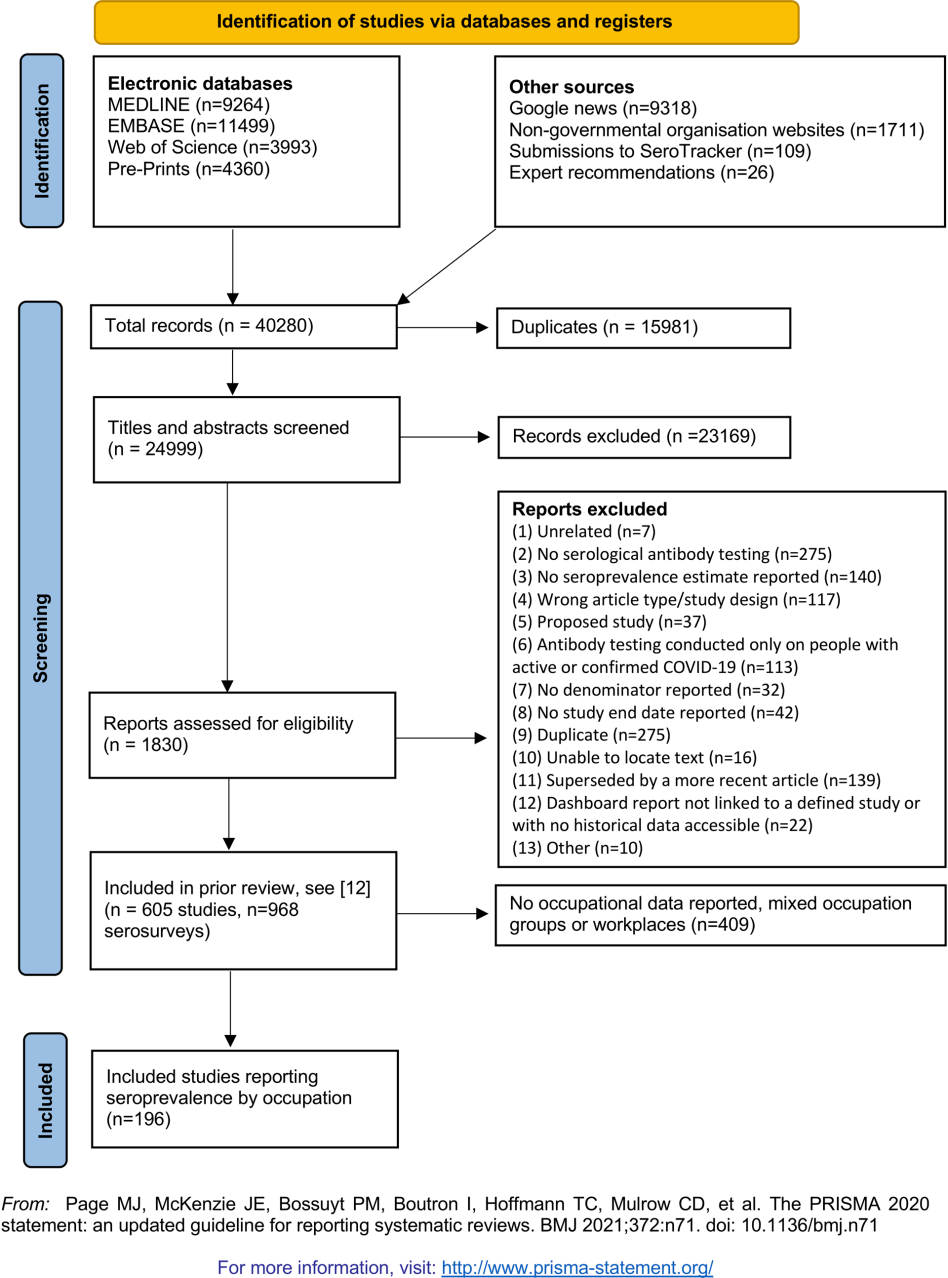
PRISMA flow diagram, Page *et al*.^18^ PRISMA, Preferred Reporting Items for Systematic Reviews and Meta-Analyses.

We identified 196 studies of occupational seroprevalence conducted in 2020 during the first and second waves of the pandemic (figure 1). There were 591 940 participants from 38 countries, including the USA (n=44 studies), UK (n=16) and Italy (n=15). Most studies (n=162; 83%) were conducted locally (eg, city, county) as opposed to regionally (eg, state; n=20; 10%) or nationally (n=14; 7%). Most were restricted to one occupational group (n=103), limiting direct comparisons (ie, using the same reference group). Sample sizes were often small (median=220, IQR 64–568 participants). Overall, 135 studies (69%) were at a high risk of bias, 47 moderate (24%), 2 low (1%) and 12 unclear (6%). Common reasons for bias were inadequate statistical analysis (ie, no adjustment for test or sample characteristics; 92%), non-probability sampling (74%) and small sample size (46%).

**Figure 2:**
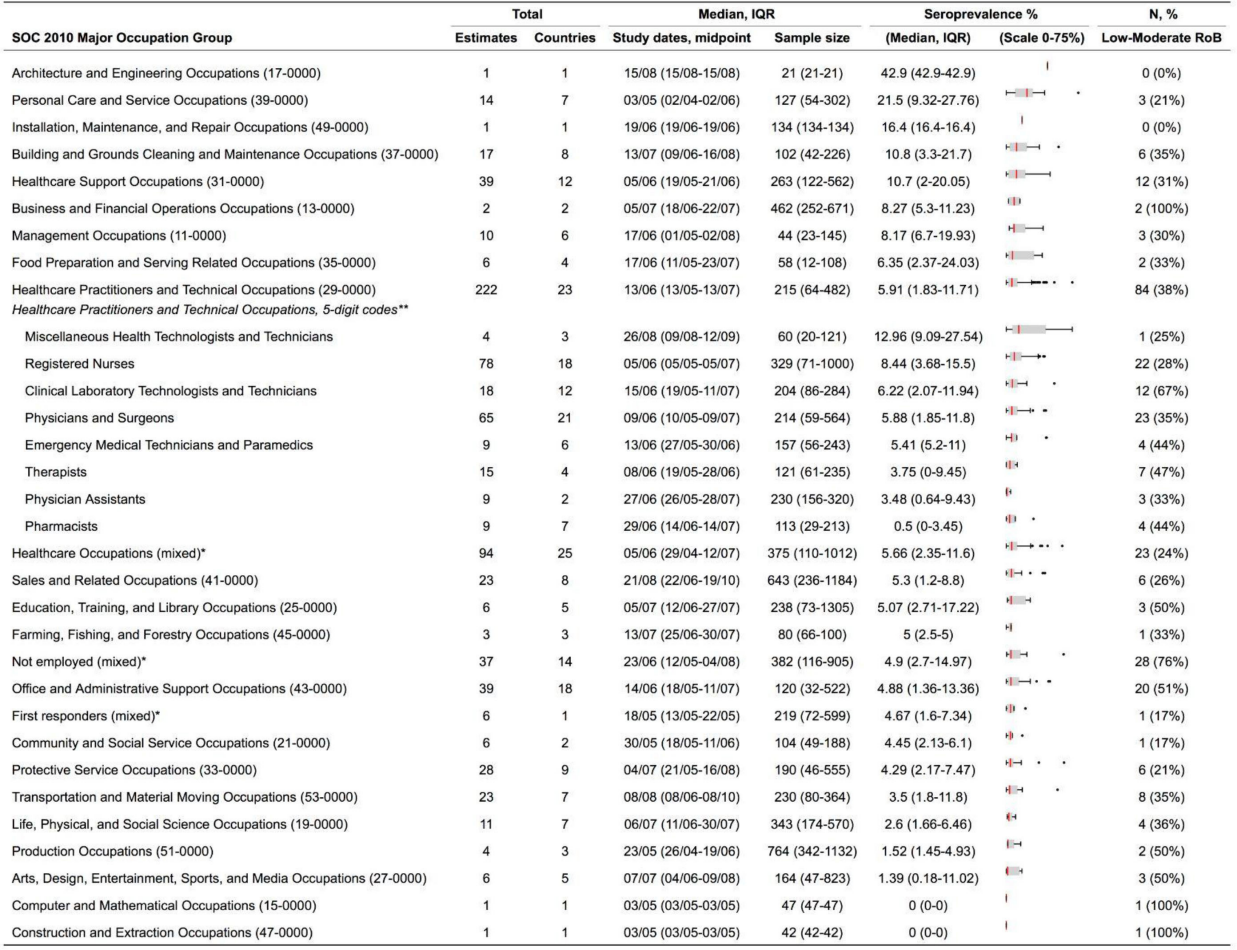
Seroprevalence by SOC 2010 major occupation group. *Estimates are a mix of ‘Healthcare Practitioners and Technical Occupations’ and ‘Healthcare Support Occupations’. SOC, Standard Occupational Classification.

At least one estimate was available for all 23 major SOC occupation groups, except for ‘legal’ and ‘military-specific’ occupations (figure 2; all studies). Over half of the 600 estimates identified (n=359) were for healthcare-related occupations. For SOC groups with three or more estimates, the highest median seroprevalence was reported for ‘personal care and service occupations’ (median 22% (IQR 9%–28%); n=14, eg, ‘personal care aids’). The next highest was reported for ‘building and grounds cleaning and maintenance’ occupations (11% (3%–22%); n=17, for example, ‘maids and housekeeping cleaners’) and ‘healthcare support’ (11% (2%–20%); n=39, eg, ‘nursing assistants’) occupations. The lowest median seroprevalence was 1% (0%–11%; n=6, eg, ‘athletes’) for ‘arts, design, entertainment, sports and media occupations.’ Individual estimates are listed in online supplemental file 2.

10.1136/bmjopen-2022-063771.supp2 Supplementary data

## Discussion

This review is the first comprehensive synthesis of occupational COVID-19 seroprevalence studies worldwide. We identified 196 studies representing 21 out of 23 major SOC groups conducted during the first and second waves of the SARS-CoV-2 pandemic in 2020, prior to the widespread roll-out of vaccines, and described occupational groups with high seroprevalence.

Seroprevalence studies may estimate the cumulative incidence of infection more accurately than diagnostic testing studies when access to testing and test performance are poor, and also can identify asymptomatic infections.^6 8^ The data identified suggest considerable differences in seroprevalence by occupation, though we did not statistically test for differences due to considerable variation in geography, study dates and workplace determinants of infection (eg, PPE, ventilation). ‘Caring and personal service’ occupations had the highest median seroprevalence (22%), which was four times higher than the unemployed (5%) and median seroprevalence across all occupational groups (5%). The UK Office for National Statistics reported a slightly lower cumulative incidence for positive diagnostic or rapid tests for COVID-19 across 25 occupational groups of 4% (mean),^4^ but the discrepancy between the true cumulative incidence and confirmed infections is likely greater in regions with less access to testing: some national, population-based serosurveys have estimated there are 10–20 serologically identifiable cases per 1 confirmed case.^12^

In future pandemics, large, well-reported, high-quality seroprevalence studies across a broad range of occupations are needed at an early stage to inform appropriate workplace policy. It has been suggested that 20% of the US workforce was exposed to disease or infection at work at least once a month prior to the pandemic.^16^ Accurate data on the occupational risks of respiratory infections, including SARS-CoV-2, are needed to inform understanding of transmission, occupational health and safety agency guidelines and allocation of resources (eg, PPE and vaccines) during outbreaks and pandemics. For governments, there are also issues of occupational disease recognition and compensation to be considered.

As such, future population-based studies on respiratory infections should collect data on occupation. In the case of epidemic infection, collaboration between academic centres with the capacity to conduct large-scale studies and government agencies with expertise in disease surveillance and access to workplace data (eg, public health, occupational health and safety) may be beneficial.^12^ Other authors have suggested the utility of occupational surveillance systems.^17^ However, the routine completion of the occupation field in electronic health records would also serve this purpose as well as informing patient reported outcome measures.

### Strengths and limitations

Despite the large number of studies of occupational seroprevalence conducted, many studies had methodological limitations. Only two studies were at a low risk of bias and most occupational subgroups had small sample sizes (median 220 participants). Many were limited to one major SOC group (n=103 studies), which precluded comparisons. Detailed descriptions of occupations were often lacking, potentially contributing to coding errors and misclassification, and workplace determinants of infection (eg, use of PPE) were poorly reported.

In conclusion, our review shows that a large number of seroprevalence studies covering a broad range of occupations were published in the first year of the pandemic. Results suggest considerable differences in seroprevalence between occupations, although few large, well-reported, high-quality studies were done. Carefully designed, adequately powered seroprevalence studies with coverage of a broad range of occupations could improve our understanding of the occupational risk of SARS-CoV-2 and other respiratory infections and should be considered an element of pandemic preparedness and response.

## Supplementary Material

Reviewer comments

Author's manuscript

## Data Availability

SeroTracker data are available in a public, open access repository. All data relevant to the study are included in the article or uploaded as online supplemental information. Seroprevalence data can be downloaded (or requested) from https://serotracker.com.
