# Supplementary material for: Occupation and SARS-CoV-2 seroprevalence studies: a systematic review
Source: BMJ Open. 2023 Feb 28;13(2):e063771. doi: 10.1136/bmjopen-2022-063771 (PMC9979591; doi:10.1136/bmjopen-2022-063771)
Supplement: Supplementary data [file bmjopen-2022-063771supp001.pdf]

Supplementary File 1 Materials

Table of Contents

Supplementary files **2**
S1. PRISMA checklist 2
S2. Search strategy 4
S3. Detailed eligibility criteria 8
S4. Tool for assessing study risk of bias 9
S5. Details of occupational coding 12
References for supplementary files **14**

Boucher E, *et al. BMJ Open* 2023; 13:e063771. doi: 10.1136/bmjopen-2022-063771

Supplementary Material

S1. PRISMA checklist

Section/topic	#	Checklist item	Reported on page #
**TITLE**			
Title	1	Identify the report as a systematic review, meta-analysis, or both.	0
**ABSTRACT**			
Structured summary	2	Provide a structured summary including, as applicable: background; objectives; data sources; study eligibility criteria, participants, and interventions; study appraisal and synthesis methods; results; limitations; conclusions and implications of key findings; systematic review registration number.	1
**INTRODUCTION**			
Rationale	3	Describe the rationale for the review in the context of what is already known.	3, lines 14-30
Objectives	4	Provide an explicit statement of questions being addressed with reference to participants, interventions, comparisons, outcomes, and study design (PICOS).	3, line 30-32
**METHODS**			
Protocol and registration	5	Indicate if a review protocol exists, if and where it can be accessed (e.g., Web address), and, if available, provide registration information including registration number.	3, line 39
Eligibility criteria	6	Specify study characteristics (e.g., PICOS, length of follow-up) and report characteristics (e.g., years considered, language, publication status) used as criteria for eligibility, giving rationale.	4, lines 39-45
Information sources	7	Describe all information sources (e.g., databases with dates of coverage, contact with study authors to identify additional studies) in the search and date last searched.	4, lines 39-40
Search	8	Present full electronic search strategy for at least one database, including any limits used, such that it could be repeated.	Suppl. File 2
Study selection	9	State the process for selecting studies (i.e., screening, eligibility, included in systematic review, and, if applicable, included in the meta-analysis).	4, lines 41-43
Data collection process	10	Describe method of data extraction from reports (e.g., piloted forms, independently, in duplicate) and any processes for obtaining and confirming data from investigators.	4, lines 41-49, 57-58
Data items	11	List and define all variables for which data were sought (e.g., PICOS, funding sources) and any assumptions and simplifications made.	4, lines 44-45 (see reference to previous study)
Risk of bias in individual studies	12	Describe methods used for assessing risk of bias of individual studies (including specification of whether this was done at the study or outcome level), and how this information is to be used in any data synthesis.	4, see reference and Suppl. File 1
Summary measures	13	State the principal summary measures (e.g., risk ratio, difference in means).	4, lines 57-78
Synthesis of results	14	Describe the methods of handling data and combining results of studies, if done, including measures of consistency (e.g., I^2) for each meta-analysis.	4, lines 57-58
Risk of bias across studies	15	Specify any assessment of risk of bias that may affect the cumulative evidence (e.g., publication bias, selective reporting within studies).	4, lines 47-48
Additional analyses	16	Describe methods of additional analyses (e.g., sensitivity or subgroup analyses, meta-regression), if done, indicating which were pre-specified.	NA
**RESULTS**			
Study selection	17	Give numbers of studies screened, assessed for eligibility, and included in the review, with reasons for exclusions at each stage, ideally with a flow diagram.	Suppl File 1
Study characteristics	18	For each study, present characteristics for which data were extracted (e.g., study size, PICOS, follow-up period) and provide the citations.	Suppl. File 2
Risk of bias within studies	19	Present data on risk of bias of each study and, if available, any outcome level assessment (see item 12).	Suppl. File 2
Results of individual studies	20	For all outcomes considered (benefits or harms), present, for each study: (a) simple summary data for each intervention group (b) effect estimates and confidence intervals, ideally with a forest plot.	Suppl. File 2
Synthesis of results	21	Present results of each meta-analysis done, including confidence intervals and measures of consistency.	NA – see narrative synthesis on page 5 & Figure 1
Risk of bias across studies	22	Present results of any assessment of risk of bias across studies (see Item 15).	5, lines 72-75 Figure 1
Additional analysis	23	Give results of additional analyses, if done (e.g., sensitivity or subgroup analyses, meta-regression [see Item 16]).	NA
**DISCUSSION**			
Summary of evidence	24	Summarize the main findings including the strength of evidence for each main outcome; consider their relevance to key groups (e.g., healthcare providers, users, and	6, lines 110-118

Boucher E, *et al. BMJ Open* 2023; 13:e063771. doi: 10.1136/bmjopen-2022-063771

		policy makers).	
Limitations	25	Discuss limitations at study and outcome level (e.g., risk of bias), and at review-level (e.g., incomplete retrieval of identified research, reporting bias).	6, lines 131-136
Conclusions	26	Provide a general interpretation of the results in the context of other evidence, and implications for future research.	6, lines 119-120
**FUNDING**			
Funding	27	Describe sources of funding for the systematic review and other support (e.g., supply of data); role of funders for the systematic review.	9

S2. Search strategy

Database: Ovid MEDLINE(R) and Epub Ahead of Print, In-Process & Other Non-Indexed Citations and Daily
Dates: January 1, 2020 to December 31, 2020
Notes: Covid-19 search terms were adapted from Ovid Expert Searches

#	Search terms
1	exp Coronavirus/
2	exp Coronavirus Infections/
3	(coronavirus* or corona virus* or OC43 or NL63 or 229E or HKU1 or HCoV* or ncov* or covid* or sars-cov* or sarscov* or Sars-coronavirus* or Severe Acute Respiratory Syndrome Coronavirus*).tw,kf.[EB2]
4	or/1-3
5	4 not ((MERS or MERS-CoV or Middle East respiratory syndrome or camel* or dromedar* or equine or coronary or coronal or covidence* or covidien or influenza virus or HIV or bovine or calves or TGEV or feline or porcine or BCoV or PED or PEDV or PDCoV or FIPV or FCoV or SADS-CoV or canine or CCov or zoonotic or avian influenza or H1N1 or H5N1 or H5N6 or IBV).mp. or (animals/ not humans/))
6	((pneumonia or covid* or coronavirus* or corona virus* or ncov* or 2019-ncov or sars* or virus).tw,kf. or exp pneumonia/) and Wuhan.tw,kf.
7	(2019-ncov* or 2019nCov* or ncov19 or ncov-19 or 2019-novel CoV or sars-cov2* or sars-cov-2* or sarscov2* or sarscov-2* or Sars-coronavirus2 or Sars-coronavirus-2 or SARS-like coronavirus* or coronavirus 2 or coronavirus2* or corona or coronavirus-19 or covid19 or covid-19 or covid 2019 or ((novel or new or nouveau) adj2 (CoV or nCoV or covid or coronavirus* or corona virus or Pandemi*2)) or ((covid or covid19* or covid-19) and pandemic*2) or (coronavirus* and pneumonia)).tw,kf.
8	COVID-19.rx,px,ox. or severe acute respiratory syndrome coronavirus 2.os.
9	or/6-8
10	5 or 9
11	immunoglobulins/ or antibodies/ or antibodies, blocking/ or exp antibodies, neutralizing/ or antibodies, viral/ or antigen-antibody complex/ or immune sera/ or exp immunoglobulin isotypes/ or immunoglobulin a/ or immunoglobulin d/ or immunoglobulin e/ or immunoglobulin g/ or immunoglobulin m/
12	serologic tests/ or complement fixation tests/ or hemagglutination inhibition tests/ or neutralization tests/
13	immunoassay/ or fluoroimmunoassay/ or exp immunoblotting/ or immunoenzyme techniques/ or exp enzyme-linked immunosorbent assay/ or exp enzyme-linked immunospot assay/ or immunosorbent techniques/ or serologic tests/ or complement fixation tests/ or hemagglutination inhibition tests/ or neutralization tests/ or Serology/di
14	(enzyme linked immunosorbent or enzyme-linked immunosorbent or ELISA or immunofluorescence or complement fixation or hemagglutination inhibition or immunoblot or western blot or neutrali*).tw,kf.
15	(antibod* or immunoglobulin* or immune globulin* or titer* or isotype* or IgG or IgM or IgA or neutrali* or sera or serum or serolog* or saliva).tw,kf.
16	or/11-14
17	seroepidemiologic studies/
18	incidence/ or prevalence/
19	(seroconver* or seroprevalence or sero-prevalence or seroincidence or sero-incidence or seroepidemiolog* or sero-epidemiolog*).mp.
20	(inciden* or prevalen* or count* or rate*).mp.
21	(serosurvey or sero-survey or screen* or diagnostic).mp.
22	(seroconver* or seroprevalence or sero-prevalence or seroincidence or sero-incidence or seroepidemiolog* or sero-epidemiolog* or inciden* or prevalen* or silent or asymptomatic or serosurvey or sero-survey).tw,kf.
23	or/17-21
24	10 and (16 and 23)
25	10 and 15
26	10 and 22
27	or/24-26
28	limit 27 to yr="2020-Current"
29	remove duplicates from 28

Boucher E, *et al. BMJ Open* 2023; 13:e063771. doi: 10.1136/bmjopen-2022-063771

Database: Embase
Dates: January 1, 2020 to December 31, 2020
Notes: Covid-19 search terms were adapted from Ovid Expert Searches

#	Searches
1	exp Coronavirus/
2	exp Coronavirus Infections/
3	(coronavirus* or corona virus* or OC43 or NL63 or 229E or HKU1 or HCoV* or ncov* or covid* or sars-cov* or sarscov* or Sars-coronavirus* or Severe Acute Respiratory Syndrome Coronavirus*).tw,kw.
4	or/1-3
5	4 not ((MERS or MERS-CoV or Middle East respiratory syndrome or camel* or dromedar* or equine or coronary or coronal or covidence* or covidien or influenza virus or HIV or bovine or calves or TGEV or feline or porcine or BCoV or PED or PEDV or PDCoV or FIPV or FCoV or SADS-CoV or canine or CCov or zoonotic or avian influenza or H1N1 or H5N1 or H5N6 or IBV).mp. or (animals/ not humans/))
6	((pneumonia or covid* or coronavirus* or corona virus* or ncov* or 2019-ncov or sars*).tw,kw. or exp pneumonia/) and Wuhan.tw,kw.
7	(2019-ncov or ncov19 or ncov-19 or 2019-novel CoV or sars-cov2 or sars-cov-2 or sarscov2 or sarscov-2 or Sars-coronavirus2 or Sars-coronavirus-2 or SARS-like coronavirus* or coronavirus-19 or covid19 or covid-19 or covid 2019 or ((novel or new or nouveau) adj2 (CoV or nCoV or covid or coronavirus* or corona virus or Pandemi*2)) or ((covid or covid19 or covid-19) and pandemic*2) or (coronavirus* and pneumonia)).tw,kw.
8	(coronavirus disease 2019 or severe acute respiratory syndrome coronavirus 2).sh,dj.
9	6 or 7 or 8
10	5 or 9
11	virus antibody/ec [Endogenous Compound]
12	neutralizing antibody/ec [Endogenous Compound]
13	exp immunoglobulin/ or exp immunoglobulin A antibody/ or exp immunoglobulin class/ or exp immunoglobulin M antibody/ or exp immunoglobulin G antibody/ or exp immunoglobulin antibody/
14	11 or 12 or 13
15	serology/
16	serodiagnosis/ or complement fixation test/ or hemagglutination inhibition test/ or hemolytic plaque assay/
17	fluorescent antibody technique/
18	immunofluorescence test/ or viral disease immunofluorescence assay/
19	enzyme linked immunosorbent assay/
20	western blotting/
21	(enzyme linked immunosorbent or enzyme-linked immunosorbent or ELISA or immunoassay or immunofluorescence or fluorescent antibody or complement fixation or hemagglutination inhibition or hemolytic plaque assay or immunoblot or western blot or neutrali*).tw,kw.
22	(antibod* or immunoglobulin* or immune globulin* or titer* or isotype* or IgG or IgM or IgA or neutrali* or sera or serolog* or serum or saliva).tw,kw.
23	15 or 16 or 17 or 18 or 19 or 20 or 21
24	14 or 23
25	exp seroepidemiology/
26	*prevalence/
27	*incidence/
28	(seroconver* or seroprevalence or sero-prevalence or seroincidence or sero-incidence or seroepidemiolog* or sero-epidemiolog* or inciden* or prevalen* or count* or rate* or serosurvey or sero-survey or screen* or diagnostic).mp.
29	(seroconver* or seroprevalence or sero-prevalence or seroincidence or sero-incidence or seroepidemiolog* or sero-epidemiolog* or inciden* or prevalen* or silent or asymptomatic or serosurvey or sero-survey).tw,kw.
30	25 or 26 or 27 or 28
31	10 and (24 and 30)
32	10 and 22
33	10 and 29
34	31 or 32 or 33
35	limit 34 to yr="2020-Current"
36	remove duplicates from 35

Database: Web of Science Core Collection
Date: January 1, 2020 to December 31, 2020

#	Searches
1	TS=(coronavirus* or corona virus* or OC43 or NL63 or 229E or HKU1 or HCoV* or ncov* or covid* or sars-cov* or sarscov* or Sars-coronavirus* or Severe Acute Respiratory Syndrome Coronavirus*)
2	TS=(MERS or MERS-CoV or Middle East respiratory syndrome or camel* or dromedar* or equine or coronary or coronal or covidence* or covidien or influenza virus or HIV or bovine or calves or TGEV or feline or porcine or BCoV or PED or PEDV or PDCoV or FIPV or FCoV or SADS-CoV or canine or CCov or zoonotic or avian influenza or H1N1 or H5N1 or H5N6 or IBV)
3	#1 NOT #2
4	TS=((pneumonia or covid* or coronavirus* or corona virus* or ncov* or 2019-ncov or sars* or virus) AND Wuhan)
5	TS=(2019-ncov* or 2019nCov* or ncov19 or ncov-19 or 2019-novel CoV or sars-cov2* or sars-cov-2* or sarscov2* or sarscov-2* or Sars-coronavirus2 or Sars-coronavirus-2 or SARS-like coronavirus* or corona or coronavirus-19 or covid19 or covid-19 or covid 2019 or ((novel or new or nouveau) adj2 (CoV or nCoV or covid or coronavirus*)) or (coronavirus* and pneumonia)).
6	TS=(COVID-19 or "severe acute respiratory syndrome coronavirus")
7	#6 OR #5 OR #4 OR #3
8	TS=(antibod* or immunoglobulin* or immune globulin* or titer* or isotype* or IgG or IgM or IgA or neutralization or sera or serolog* or saliva or serum).
9	TS=("enzyme linked immunosorbent assay" or "enzyme-linked immunosorbent assay" or "immunoenzyme" or ELISA or "lateral flow immunoassay" or LFIA or "immunofluorescence assay" or immunochromatography or "complement fixation test" or "hemagglutination inhibition" or immunoblot or "western blot" or "neutralization assay")
10	#9 OR #8
11	TI=(seroconversion or seroprevalence or seroincidence or seroepidemiolog* or incidence or prevalence or asymptomatic or sero-survey*) or AK=(seroconversion or seroprevalence or seroincidence or seroepidemiolog* or incidence or prevalence or asymptomatic or sero-survey*)
12	ALL=(prevalence or incidence or seroconversion or seroconvert or seroprevalence or seroincidence or seroepidemiolog* or serosurvey or sero-survey or survey or screen* or diagnostic test)
13	#12 AND #10 AND #7
14	#11 AND #7
15	TI=(antibod* or immunoglobulin* or immune globulin* or titer* or isotype* or IgG or IgM or IgA or neutralization or sera or serolog* or saliva or serum).
16	#15 AND #7
17	#16 OR #14 OR #13

Database: Europe PMC [Secondary search for pre-prints]
Dates: January 1, 2020 to December 31, 2020

#	Searches
	("2019-nCoV" OR "2019nCoV" OR "COVID-19" OR "SARS-CoV-2" OR "COVID19" OR "COVID" OR "SARS-nCoV" OR ("wuhan" AND "coronavirus") OR "Coronavirus" OR "Corona virus" OR "corona-virus" OR "corona viruses" OR "coronaviruses" OR "SARS-CoV" OR "Severe Acute Respiratory Syndrome Coronavirus" OR ("SARS" AND "coronavirus")) AND ABSTRACT:(sera* OR sero* OR immun* OR Ig* OR “enzyme-linked immunosorbent assay” OR ELISA OR “neutralization assay" OR seroprevalence) AND (SRC:"PPR")

Sources: Health organizations
Dates: January 1, 2020 to December 31, 2020

Source	Search strategy	
WHO Situation Reports	1	“antibod”, “sero”, “immun”, “ELISA”
National Institutes of Health	1	("COVID" OR "SARS-CoV-2")
	2	("sero*" OR "antibod*" OR "immun*" OR "RDT" OR "ELISA" OR "LFIA")
	3	allintext:(1 AND 2) site:nih.gov -site:ncbi.nlm.nih.gov
	3	2 AND 3
United States Centres for Disease Control and Prevention	1	("COVID" OR "SARS-CoV-2")
	2	("sero*" OR "antibod*" OR "immun*" OR "RDT" OR "ELISA" OR "LFIA")
	3	allintext:(1 AND 2) site:cdc.gov
	5	2 AND 3
European Centres for Disease Control and Prevention	1	("COVID" OR "SARS-CoV-2")
	2	("sero*" OR "antibod*" OR "immun*" OR "RDT" OR "ELISA" OR "LFIA")
	3	allintext:(1 AND 2) site:ecdc.europa.eu
	5	2 AND 3

Sources: Google News
Dates: January 1, 2020 to December 31, 2020

Source	Search strategy	
Google news	1	(antibody OR antibodies OR surveillance OR screen OR serology OR serological OR serosurvey OR ELISA OR LFIA OR assay OR blood OR serum OR immune OR immunity OR herd immunity OR random test)

Boucher E, *et al. BMJ Open* 2023; 13:e063771. doi: 10.1136/bmjopen-2022-063771

S3. Detailed eligibility criteria

This study included eligible studies from the SeroTracker database. Eligibility criteria for the database and also for this review specifically are outlined below:

Eligibility criteria for inclusion in SeroTracker database	Eligibility criteria for inclusion in this review
Study performed serologic testing to determine the prevalence of SARS-CoV-2 antibodies in a human population over a specified time period.	Studies included in the SeroTracker database (https://serotracker.com) with relevant subgrouping (i.e., "Occupation," or "Employment status") and/or sample frame variables (i.e., "Healthcare workers and caregivers," "Non-essential workers and unemployed persons," "Essential non-healthcare workers," or "Multiple populations") variables. We also manually searched for potentially relevant studies not falling into these categories.
Reported sample size, sampling date, location and prevalence.	Study published between January 01 and December 31, 2020.
Article in English or French or could be fully extracted using machine translation.	Article written in English or French or machine-translatable using Google Translate.
Article did not report identical information to previously included studies (peer-reviewed studies were prioritised over news stories and pre-prints where available).	Reported seroprevalence data that could be fit into the 23 major SOC 2010 occupation categories or combined categories for healthcare workers, first-responders or unemployed persons.<br><br>Studies that only reported seroprevalence for mixed occupation groups or workplaces rather than specific occupations (e.g., "hospital staff") were excluded.
Studies conducted only in people previously diagnosed with COVID-19 (molecular or antigen testing, or clinical or self-assessment).	Seroprevalence estimates did not include people <18 years (i.e., possibly affected by COVID-19 exposure at school, which could impact occupational seroprevalence estimates).
Cohort or cross-sectional design (case reports, case-control studies, trials, and reviews were excluded, as were dashboards not associated with a defined serology study).	

Boucher E, *et al. BMJ Open* 2023; 13:e063771. doi: 10.1136/bmjopen-2022-063771

S4. Tool for assessing study risk of bias

**Item 1: Was the sample frame appropriate to address the target population?**	
Yes	Sample frame described and it approximated the target population
No	Sample frame did not approximate the target population (e.g., blood donors do not represent general population, doctors do not represent all health care providers)
Exclude	Sample frame not described
*Notes	The term "target population" should not be taken to infer every individual from everywhere or with similar disease or exposure characteristics. Instead, give consideration to specific population characteristics in the study, including age range, gender, morbidities, medications, and other potentially influential factors. For example, a sample frame may not be appropriate to address the target population if a certain group has been used (such as those working for one organisation, or one profession) and the results then inferred to the target population (i.e. working adults). A sample frame may be appropriate when it includes almost all the members of the target population (i.e. a census, or a complete list of participants or complete registry data).

**Item 2: Were study participants recruited in an appropriate way?**	
Yes	Probability sampling method (simple or stratified random) or entire sample (e.g., an entire town) was used
No	Non-probability sampling
Exclude	Sampling method not reported

**Item 3: Was the sample size adequate?**	
Yes	$\geq$599
No	<599
Exclude	Sample size not reported
*Notes	To calculate the required sample size we used an assumed prevalence of 2.5%, which was the global average estimated by the WHO in April, 2020.[1] Based on guidance by the Joanna Briggs Institute and published medical statistical recommendations we selected a precision value that was half the assumed prevalence (1.25%) [2,3]. We calculated a minimum sample size of 599 using these inputs:<br>Sample size calculation: $n = \frac{Z^2 P(1-P)}{d^2}$<br>Where n = sample size;<br>Z = Z statistic for level of confidence (95%);<br>P = expected prevalence (2.5% WHO global estimate);<br>d = precision (1.25%)<br><br>In cases where the sample size calculation was provided and the required sample for 80% power was below our threshold (n<599), this item was marked as yes.

**Item 4: Were the study subjects and setting described in detail?**	
Yes	Average age and distribution of gender/sex provided
No	Neither age or gender/sex is provided, or only one of age and gender/sex is provided

**Item 5: Was data analysis conducted with sufficient coverage of the identified sample?**	
Yes	The demographic characteristics (gender/sex, age, and ethnicity) of the sample is at least somewhat representative of the population
No	The demographic characteristics (gender/sex, age, and ethnicity) of the sample is not representative of the population

Boucher E, *et al. BMJ Open* 2023; 13:e063771. doi: 10.1136/bmjopen-2022-063771

Unclear	Information is not provided about demographic characteristics of the sample (gender/sex, age, and ethnicity)

**Item 6: Were valid methods used for the identification of the condition?**	
Yes	The test used met the FDA standards for Emergency Use Authorizations for COVID-19 serological tests: sensitivity minimum 90%, specificity minimum 95%, as reported in the study [4].
No	The test used did not meet the FDA standards for Emergency Use Authorizations for COVID-19 serological tests: sensitivity minimum 90%, specificity minimum 95%.
Exclude	Test sensitivity and specificity not reported

**Item 7: Was the condition measured in a standard, reliable way for all participants?**	
Yes	The same serology test was used for all participants
No	Different serology tests were used for participants
Unclear	No details were provided about which participants received which serology tests

**Item 8: Was there appropriate statistical analysis?**	
Yes	Does all of the following: corrects for population characteristics or the sample is somewhat representative of the population (probability sampling), corrects for test characteristics), and provides the information necessary to determine the numerator, denominator, prevalence estimate, and confidence interval.
No	Does not correct for population characteristics and the sample is not likely representative of the population (non-probability sampling), does not correct for test or provide the information necessary to correct for test characteristics, or does not provide the information necessary to determine the numerator, denominator, prevalence estimate, and confidence interval.

**Item 9: Was the response rate adequate, and if not, was the low response rate managed appropriately?**	
Yes	Response rate > 60% or the demographics of the sample were a reasonable match to those of the target population [5]
No	Response rate < 60% and the demographics of the sample were not a reasonable match to those of the target population
Unclear	Response rate not provided and it was unclear if the demographics of the sample differed from the target population

**Item 10: Overall risk of bias**	
Low	The estimates are very likely correct for the target population. To obtain a low risk of bias classification, all criteria must be met or departures from the criteria must be minimal and unlikely to impact on the validity and reliability of the prevalence estimate. These include sample sizes that are just below the threshold when all other criteria are met, reporting only some of characteristics of the sample, test characteristics below the threshold but corrections for the test performance, and response rates that are just below the threshold in the context of probability based sampling of an appropriate sampling frame with population weighted seroprevalence estimates.
Moderate	The estimates are likely correct for the target population. To obtain a moderate risk of bias classification, most criteria must be met and departures from the criteria are likely to have only a small impact on the validity and reliability of the prevalence estimates.
High	The estimates are not likely correct for the target population. To obtain a high risk of bias, many criteria must not be met or departures from criteria are likely to have a major impact on the validity and reliability of the prevalence estimates.
Unclear	There was insufficient information to assess the risk of bias.

S5. Details of occupational coding

For each seroprevalence estimate, we identified the relevant Standard Occupational Classification (SOC) 2010 codes. This was done by applying the National Institute for Occupational Safety & Health (NIOSH) Industry and Occupation Computerized Coding System (NIOCCS) to text occupation descriptions extracted by members of the research team. There is no standard cut-off for manually verifying results from the National Institute for Occupational Safety & Health (NIOSH) Industry and Occupation Computerized Coding System (NIOCCS). However, NIOCCS reports the probability of correct classification to the six-digit level. After manually verifying a subset of records from the first round of classification, we decided to manual perform a second round of classification for any observations for which the probability of correct classification was <0.8. This cut-off was chosen based on the observation that that most codes with a probability of correct classification to of ≥0.8 to the six-digit level were correctly coded at the two- and three-digit level, which we used in our main analyses and are more likely to be coded correctly than the more granular, 6-digit codes and consideration of the number of records that could feasibly be verified manually

References for supplementary files

1. Boseley S. WHO warns that few have developed antibodies to Covid-19. The Guardian [Internet]. 2020 Apr 20; Available from: https://www.theguardian.com/society/2020/apr/20/studies-suggest-very-few-have-had-covid-19-without-symptoms
2. Munn Z, Moola S, Lisy K, Riitano D, Tufanaru C. Methodological guidance for systematic reviews of observational epidemiological studies reporting prevalence and cumulative incidence data. Int J Evid Based Healthc. 2015 Sep;13(3):147–53.
3. Naing L, Winn T, Ruslil B. Practical issues in calculating the sample size for prevalence studies. Arch Orofac Sci. 2006;1:9–14.
4. U.S. Food & Drug Administration. Emergency Use Authorization for SARS-CoV-2 Antibody Tests [Internet]. 2020 [cited 2020 May 5]. Available from: https://www.fda.gov/media/137470/download.
5. Morton MBS, Bandara DK, Robinson EM, Carr PEA. In the 21st century, what is an acceptable response rate? Aust N Z J Public Health. 2012 April; 36 (2): 106-8.
6 Bobrovitz N, Arora RK, Cao C, Boucher E, Liu M, Donnici C, Yanes-Lane M, Whelan M, Perlman-Arrow S, Chen J, Rahim H. Global seroprevalence of SARS-CoV-2 antibodies: A systematic review and meta-analysis. PloS one. 2021 Jun 23;16(6):e0252617.

33
