# Supplementary material for: Occupation and SARS-CoV-2 seroprevalence studies: a systematic review
Source: BMJ Open. 2023 Feb 28;13(2):e063771. doi: 10.1136/bmjopen-2022-063771 (PMC9979591; doi:10.1136/bmjopen-2022-063771)
Supplement: Supplementary data [file bmjopen-2022-063771supp002.pdf]

Supplementary File I. List of all estimates, included studies and references

SOC 2010 Major Group	Study	N	SOC 2010 Occupation Title	Study Type	Study Dates	Country	Serum positive prevalence (95% CIs)	Overall Risk of Bias (JBI)
Not employed (mixed)*	Merkely et al., 2020[1]	n=209	Homemaker (Unpaid)	Cross-sectional survey	05/01 - 05/16	Hungary	0.73% (0-1.74%)	Moderate
Not employed (mixed)*	Siddiqui et al., 2020[2]	n=37	Homemaker (Unpaid)	Prospective cohort	04/15 - 08/15	India	18.9%	High
Not employed (mixed)*	Biggs et al., 2020[3]	n=157	Retired (Unpaid)	Cross-sectional survey	04/28 - 05/03	United States of America	1.91%	Moderate
Not employed (mixed)*	Carrat et al., 2020[4]	n=5381	Retired (Unpaid)	Prospective cohort	05/04 - 06/23	France	4.3% (3.5-5%)	Moderate
Not employed (mixed)*	Merkely et al., 2020[1]	n=2767	Retired (Unpaid)	Cross-sectional survey	05/01 - 05/16	Hungary	1.09% (0.66-1.52%)	Moderate
Not employed (mixed)*	Richard et al., 2020[5]	n=1635	Retired (Unpaid)	Cross-sectional survey	04/06 - 06/30	Switzerland	4.3%	Low
Not employed (mixed)*	Siddiqui et al., 2020[2]	n=10	Retired (Unpaid)	Prospective cohort	04/15 - 08/15	India	20%	High
Not employed (mixed)*	Alemu et al., 2020[6]	n=32	Student (Unpaid)	Cross-sectional survey	04/23 - 04/28	Ethiopia	15.6%	Moderate
Not employed (mixed)*	Biggs et al., 2020[3]	n=16	Student (Unpaid)	Cross-sectional survey	04/28 - 05/03	United States of America	12.5%	Moderate
Not employed (mixed)*	Brehm et al., 2020[7]	n=73	Student (Unpaid)	Cross sectional study with prospective cohort follow up of a subset of the sample	03/20 - 07/17	Germany	2.7%	Moderate
Not employed (mixed)*	Carrat et al., 2020[4]	n=81	Student (Unpaid)	Prospective cohort	05/04 - 06/23	France	7.2% (0.1-12.6%)	Moderate

Not employed (mixed)*	Iversen et al., 2020[8]	n=688	Student (Unpaid)	Cross-sectional survey	04/15 - 04/22	Denmark	14.97%	Low
Not employed (mixed)*	Lumley et al., 2020[9]	n=620	Student (Unpaid)	Prospective cohort	04/23 - 11/30	The United Kingdom	6.77%	Moderate
Not employed (mixed)*	Merkely et al., 2020[1]	n=774	Student (Unpaid)	Cross-sectional survey	05/01 - 05/16	Hungary	0.69% (0-1.49%)	Moderate
Not employed (mixed)*	Richard et al., 2020[5]	n=666	Student (Unpaid)	Cross-sectional survey	04/06 - 06/30	Switzerland	10.5%	Low
Not employed (mixed)*	Shakiba et al., 2020[10]	n=114	Student (Unpaid)	Cross-sectional survey	04/11 - 04/19	Iran (Islamic Republic of)	17.5% (11.3-23.7%)	Moderate
Not employed (mixed)*	Siddiqui et al., 2020[2]	n=14	Student (Unpaid)	Prospective cohort	04/15 - 08/15	India	21.4%	High
Not employed (mixed)*	Tilley et al., 2020[11]	n=790	Student (Unpaid)	Cross-sectional survey	04/29 - 05/08	United States of America	4% (3-5.1%)	Moderate
Not employed (mixed)*	Tsitsilonis et al., 2020[12]	n=1395	Student (Unpaid)	Cross-sectional survey	06/15 - 07/15	Greece	0.42% (0.03-1.5%)	Moderate
Not employed (mixed)*	Arnaldo et al., 2020[13]	n=513	Military, Rank Not Specified	Cross-sectional survey	07/06 - 07/13	Mozambique	3.7%	High
Not employed (mixed)*	Arnaldo et al., 2020[14]	n=116	Military, Rank Not Specified	Cross-sectional survey	11/02 - 11/12	Mozambique	1.7%	High
Not employed (mixed)*	Mabunda et al., 2020[15]	n=324	Military, Rank Not Specified	Cross-sectional survey	09/21 - 10/02	Mozambique	2.8%	High
Not employed (mixed)*	Mahomed et al., 2020[16]	n=116	Military, Rank Not Specified	Cross-sectional survey	11/26 - 12/03	Mozambique	18.1%	High
Not employed (mixed)*	Payne et al., 2020[17]	n=382	Military, Rank Not Specified	Cross-sectional survey	04/20 - 04/24	United States of America	59.7%	High
Not employed (mixed)*	World et al., 2020[18]	n=6900	Military, Rank Not Specified	Cross-sectional survey	08/15 - 10/15	Republic of Korea	0.36%	Unclear
Management Occupations (11-0000)	Shakiba et al., 2020[10]	n=16	Farmers, Ranchers, and Other Agricultural Managers	Cross-sectional survey	04/11 - 04/19	Iran (Islamic Republic of)	19.7% (9.1-31%)	Moderate
Management Occupations (11-	Favara et al., 2020[19]	n=43	Medical and Health Services Managers	Cross-sectional survey	07/13 - 07/13	The United Kingdom	9.3%	High

0000)								
Management Occupations (11-0000)	Galan et al., 2020[20]	n=170	Medical and Health Services Managers	Cross-sectional survey	04/14 - 04/27	Spain	27.6%	High
Management Occupations (11-0000)	Hunter et al., 2020[21]	n=44	Medical and Health Services Managers	Cross-sectional survey	04/29 - 05/08	United States of America	4.55%	High
Management Occupations (11-0000)	Leidner et al., 2020[22]	n=257	Medical and Health Services Managers	Cross sectional study with prospective cohort follow up of a subset of the sample	04/08 - 05/22	United States of America	3.11%	High
Management Occupations (11-0000)	Martin et al., 2020[23]	n=2078	Medical and Health Services Managers	Cross-sectional survey	05/29 - 07/13	The United Kingdom	6.79%	Moderate
Management Occupations (11-0000)	Siddiqui et al., 2020[2]	n=15	Medical and Health Services Managers	Prospective cohort	04/15 - 08/15	India	20%	High
Management Occupations (11-0000)	Baracco et al., 2020[24]	n=45	Managers, All Other	Cross-sectional survey	04/23 - 05/05	Italy	6.67%	High
Management Occupations (11-0000)	Goenka et al., 2020[25]	n=71	Managers, All Other	Cross-sectional survey	07/12 - 08/23	India	7.04%	Moderate
Management Occupations (11-0000)	Goenka et al., 2020[26]	n=13	Managers, All Other	Cross-sectional survey	08/01 - 08/31	India	38.46%	High
Business and Financial Operations Occupations (13-0000)	Satpati et al., 2020[27]	n=43	Management Analysts	Cross-sectional survey	07/26 - 08/08	India	2.33%	Moderate
Business and Financial	Poustchi et al., 2020[28]	n=880	Financial Specialists	Cross-sectional survey	04/17 - 06/02	Iran (Islamic Republic of)	14.2% (12.1-16.5%)	Moderate

Operations Occupations (13-0000)								
Computer and Mathematical Occupations (15-0000)	Biggs et al., 2020[3]	n=47	Computer User Support Specialists	Cross-sectional survey	04/28 - 05/03	United States of America	0%	Moderate
Architecture and Engineering Occupations (17-0000)	Siddiqui et al., 2020[2]	n=21	Engineers	Prospective cohort	04/15 - 08/15	India	42.9%	High
Life, Physical, and Social Science Occupations (19-0000)	Jones et al., 2020[29]	n=245	Medical Scientists	Cross-sectional survey	01/15 - 06/15	The United Kingdom	1.9%	High
Life, Physical, and Social Science Occupations (19-0000)	Anna et al., 2020[30]	n=505	Medical Scientists, Except Epidemiologists	Prospective cohort	04/28 - 07/31	France	8.71%	Moderate
Life, Physical, and Social Science Occupations (19-0000)	Erber et al., 2020[31]	n=635	Medical Scientists, Except Epidemiologists	Cross-sectional survey	04/14 - 05/29	Germany	1.24%	High
Life, Physical, and Social Science Occupations (19-0000)	Favara et al., 2020[19]	n=38	Medical Scientists, Except Epidemiologists	Cross-sectional survey	07/13 - 07/13	The United Kingdom	2.6%	High
Life, Physical, and Social Science Occupations (19-0000)	Hanrath et al., 2020[32]	n=468	Medical Scientists, Except Epidemiologists	Cross-sectional survey	05/29 - 07/06	The United Kingdom	6.2%	High
Life, Physical, and Social Science Occupations (19-0000)	Leidner et al., 2020[22]	n=2654	Medical Scientists, Except Epidemiologists	Cross sectional study with prospective cohort follow up of a subset of the sample	04/08 - 05/22	United States of America	2.22%	High

Life, Physical, and Social Science Occupations (19-0000)	Martin et al., 2020[23]	n=1154	Medical Scientists, Except Epidemiologists	Cross-sectional survey	05/29 - 07/13	The United Kingdom	9.71%	Moderate
Life, Physical, and Social Science Occupations (19-0000)	Rosser et al., 2020[33]	n=102	Medical Scientists, Except Epidemiologists	Cross-sectional survey	04/20 - 05/20	United States of America	0.98%	High
Life, Physical, and Social Science Occupations (19-0000)	Silva et al., 2020[34]	n=69	Chemists	Cross-sectional survey	06/05 - 07/31	Brazil	4%	High
Life, Physical, and Social Science Occupations (19-0000)	Tsitsilonis et al., 2020[12]	n=250	Physical Scientists, All Other	Cross-sectional survey	06/15 - 07/15	Greece	1.42% (0-7.24%)	Moderate
Community and Social Service Occupations (21-0000)	Jones et al., 2020[29]	n=211	Healthcare Social Workers	Cross-sectional survey	01/15 - 06/15	The United Kingdom	6.3%	High
Community and Social Service Occupations (21-0000)	Leidner et al., 2020[22]	n=235	Social Workers, All Other	Cross sectional study with prospective cohort follow up of a subset of the sample	04/08 - 05/22	United States of America	3.4%	High
Community and Social Service Occupations (21-0000)	Rosser et al., 2020[33]	n=117	Social Workers, All Other	Cross-sectional survey	04/20 - 05/20	United States of America	1.71%	High
Community and Social Service Occupations (21-0000)	Sabourin et al., 2020[35]	n=91	Social Workers, All Other	Cross-sectional survey	07/15 - 08/15	United States of America	5.49%	High
Community and Social Service	Yogo et al., 2020[36]	n=35	Social Workers, All Other	Cross-sectional survey	05/20 - 06/08	United States of America	0%	High

Occupations (21-0000)								
Community and Social Service Occupations (21-0000)	Biggs et al., 2020[3]	n=6	Religious Workers	Cross-sectional survey	04/28 - 05/03	United States of America	16.67%	Moderate
Education, Training, and Library Occupations (25-0000)	Campos et al., 2020[37]	n=2715	Postsecondary Teachers	Cross-sectional survey	05/13 - 07/10	Portugal	2.6%	High
Education, Training, and Library Occupations (25-0000)	Goncalves et al., 2020[38]	n=1636	Postsecondary Teachers	Cross-sectional survey	06/15 - 06/30	Portugal	3.05%	Moderate
Education, Training, and Library Occupations (25-0000)	Tsitsilonis et al., 2020[12]	n=312	Postsecondary Teachers	Cross-sectional survey	06/15 - 07/15	Greece	1.2% (0.14-3.7%)	Moderate
Education, Training, and Library Occupations (25-0000)	Fontanet et al., 2020[39]	n=42	Elementary and Middle School Teachers	Retrospective cohort	04/28 - 04/30	France	7.1%	Moderate
Education, Training, and Library Occupations (25-0000)	Siddiqui et al., 2020[2]	n=8	Elementary and Middle School Teachers	Prospective cohort	04/15 - 08/15	India	25%	High
Education, Training, and Library Occupations (25-0000)	Torres et al., 2020[40]	n=165	Elementary and Middle School Teachers	Cross-sectional survey	05/04 - 05/19	Chile	20.6% (14.7-27.6%)	High

Arts, Design, Entertainment, Sports, and Media Occupations (27-0000)	Halatoko et al., 2020[41]	n=55	Fine Artists, Including Painters, Sculptors, and Illustrators	Cross-sectional survey	04/23 - 05/08	Togo	0%	High
Arts, Design, Entertainment, Sports, and Media Occupations (27-0000)	Slusser et al., 2020[42]	n=5603	Athletes, Coaches, Umpires, and Related Workers	Cross-sectional survey	04/08 - 04/21	United States of America	0.7% (0.28-1.15%)	Unclear
Arts, Design, Entertainment, Sports, and Media Occupations (27-0000)	Vince et al., 2020[43]	n=272	Athletes, Coaches, Umpires, and Related Workers	Prospective cohort	05/29 - 07/31	Croatia	14%	Moderate
Arts, Design, Entertainment, Sports, and Media Occupations (27-0000)	Vince et al., 2020[43]	n=43	Coaches and Scouts	Prospective cohort	05/29 - 07/31	Croatia	16.3%	Moderate
Arts, Design, Entertainment, Sports, and Media Occupations (27-0000)	Mack et al., 2020[44]	n=1007	Umpires, Referees, and Other Sports Officials	Prospective cohort	06/16 - 06/30	Germany	2.09% (1.37-3.17%)	High
Arts, Design, Entertainment, Sports, and Media Occupations (27-0000)	Khan et al., 2020[45]	n=44	Media and Communication Workers	Cross-sectional survey	07/01 - 07/15	India	0%	Moderate
Healthcare Practitioners and Technical Occupations (29-0000)	Akinbami et al., 2020[46]	n=566	Healthcare Practitioners and Technical Occupations	Cross-sectional survey	05/18 - 06/13	United States of America	4.6% (3-6.7%)	Moderate

Healthcare Practitioners and Technical Occupations (29-0000)	Khan et al., 2020[45]	n=355	Healthcare Practitioners and Technical Occupations	Cross-sectional survey	07/01 - 07/15	India	4.8% (3-7.6%)	Moderate
Healthcare Practitioners and Technical Occupations (29-0000)	Leidner et al., 2020[22]	n=402	Healthcare Practitioners and Technical Occupations	Cross sectional study with prospective cohort follow up of a subset of the sample	04/08 - 05/22	United States of America	1.49%	High
Healthcare Occupations (mixed)*	Hanrath et al., 2020[32]	n=102	Healthcare Practitioners and Technical Occupations and Healthcare Support Occupations*	Cross-sectional survey	05/29 - 07/06	The United Kingdom	6.62%	High
Healthcare Occupations (mixed)*	Jones et al., 2020[29]	n=413	Healthcare Practitioners and Technical Occupations and Healthcare Support Occupations*	Cross-sectional survey	01/15 - 06/15	The United Kingdom	7.8%	High
Healthcare Occupations (mixed)*	Martin et al., 2020[23]	n=550	Healthcare Practitioners and Technical Occupations and Healthcare Support Occupations*	Cross-sectional survey	05/29 - 07/13	The United Kingdom	10.36%	Moderate
Healthcare Occupations (mixed)*	Amendola et al., 2020[47]	n=117	Healthcare Practitioners and Technical Occupations and Healthcare Support Occupations*	Cross-sectional survey	04/15 - 04/15	Italy	4.27%	High
Healthcare Occupations (mixed)*	Arnaldo et al., 2020[48]	n=543	Healthcare Practitioners and Technical Occupations and Healthcare Support Occupations*	Cross-sectional survey	08/10 - 08/21	Mozambique	3.7%	High

Healthcare Occupations (mixed)*	Bal et al., 2020[49]	n=190	Healthcare Practitioners and Technical Occupations and Healthcare Support Occupations*	Cross-sectional survey	04/10 - 05/28	France	3.68%	High
Healthcare Occupations (mixed)*	Barallat et al., 2020[50]	n=429	Healthcare Practitioners and Technical Occupations and Healthcare Support Occupations*	Cross-sectional survey	05/04 - 05/22	Spain	7.69%	High
Healthcare Occupations (mixed)*	Bardai et al., 2020[51]	n=35	Healthcare Practitioners and Technical Occupations and Healthcare Support Occupations*	Cross-sectional survey	06/10 - 07/27	Canada	11%	High
Healthcare Occupations (mixed)*	Bardai et al., 2020[51]	n=20	Healthcare Practitioners and Technical Occupations and Healthcare Support Occupations*	Cross-sectional survey	06/10 - 07/27	Canada	15%	High
Healthcare Occupations (mixed)*	Bardai et al., 2020[51]	n=44	Healthcare Practitioners and Technical Occupations and Healthcare Support Occupations*	Cross-sectional survey	06/10 - 07/27	Canada	11%	High
Healthcare Occupations (mixed)*	Bardai et al., 2020[51]	n=99	Healthcare Practitioners and Technical Occupations and Healthcare Support Occupations*	Cross-sectional survey	06/10 - 07/27	Canada	12%	High
Healthcare Occupations (mixed)*	Biggs et al., 2020[3]	n=59	Healthcare Practitioners and Technical Occupations and Healthcare Support Occupations*	Cross-sectional survey	04/28 - 05/03	United States of America	10.17%	Moderate

Healthcare Occupations (mixed)*	Blairon et al., 2020[52]	n=588	Healthcare Practitioners and Technical Occupations and Healthcare Support Occupations*	Cross-sectional survey	05/25 - 06/19	Belgium	19.2%	High
Healthcare Occupations (mixed)*	Borraz et al., 2020[53]	n=289	Healthcare Practitioners and Technical Occupations and Healthcare Support Occupations*	Prospective cohort	03/20 - 04/21	Spain	5.88%	High
Healthcare Occupations (mixed)*	Brunner et al., 2020[54]	n=762	Healthcare Practitioners and Technical Occupations and Healthcare Support Occupations*	Cross-sectional survey	05/04 - 05/29	United States of America	4.5%	High
Healthcare Occupations (mixed)*	Brunner et al., 2020[54]	n=764	Healthcare Practitioners and Technical Occupations and Healthcare Support Occupations*	Cross-sectional survey	05/04 - 05/29	United States of America	2%	High
Healthcare Occupations (mixed)*	Carozzi et al., 2020[55]	n=17098	Healthcare Practitioners and Technical Occupations and Healthcare Support Occupations*	Cross-sectional survey	04/01 - 04/30	Italy	3.1%	High
Healthcare Occupations (mixed)*	Carrat et al., 2020[4]	n=568	Healthcare Practitioners and Technical Occupations and Healthcare Support Occupations*	Prospective cohort	05/04 - 06/23	France	11.6% (8.3-14.4%)	Moderate
Healthcare Occupations (mixed)*	Cavlek et al., 2020[56]	n=558	Healthcare Practitioners and Technical Occupations and Healthcare Support Occupations*	Cross-sectional survey	04/25 - 05/24	Croatia	1.25%	High

Healthcare Occupations (mixed)*	Chibwana et al., 2020[57]	n=500	Healthcare Practitioners and Technical Occupations and Healthcare Support Occupations*	Prospective cohort	05/22 - 06/19	Malawi	12.3% (8.2-16.5%)	High
Healthcare Occupations (mixed)*	Coffman et al., 2020[58]	n=1100	Healthcare Practitioners and Technical Occupations and Healthcare Support Occupations*	Cross-sectional survey	07/01 - 07/31	United States of America	2.2%	Unclear
Healthcare Occupations (mixed)*	Cooper et al., 2020[59]	n=118	Healthcare Practitioners and Technical Occupations and Healthcare Support Occupations*	Cross-sectional survey	06/10 - 08/07	The United Kingdom	8.47%	Moderate
Healthcare Occupations (mixed)*	Cooper et al., 2020[59]	n=27	Healthcare Practitioners and Technical Occupations and Healthcare Support Occupations*	Cross-sectional survey	06/10 - 08/07	The United Kingdom	14.81%	Moderate
Healthcare Occupations (mixed)*	Cooper et al., 2020[59]	n=24	Healthcare Practitioners and Technical Occupations and Healthcare Support Occupations*	Cross-sectional survey	06/10 - 08/07	The United Kingdom	12.5%	Moderate
Healthcare Occupations (mixed)*	Cooper et al., 2020[59]	n=1068	Healthcare Practitioners and Technical Occupations and Healthcare Support Occupations*	Cross-sectional survey	06/10 - 08/07	The United Kingdom	5.43%	Moderate
Healthcare Occupations (mixed)*	Cooper et al., 2020[59]	n=174	Healthcare Practitioners and Technical Occupations and Healthcare Support Occupations*	Cross-sectional survey	06/10 - 08/07	The United Kingdom	5.75%	Moderate

Healthcare Occupations (mixed)*	Cooper et al., 2020[59]	n=319	Healthcare Practitioners and Technical Occupations and Healthcare Support Occupations*	Cross-sectional survey	06/10 - 08/07	The United Kingdom	11.29%	Moderate
Healthcare Occupations (mixed)*	Cooper et al., 2020[59]	n=5698	Healthcare Practitioners and Technical Occupations and Healthcare Support Occupations*	Cross-sectional survey	06/10 - 08/07	The United Kingdom	7.2%	Moderate
Healthcare Occupations (mixed)*	Cooper et al., 2020[59]	n=412	Healthcare Practitioners and Technical Occupations and Healthcare Support Occupations*	Cross-sectional survey	06/10 - 08/07	The United Kingdom	4.61%	Moderate
Healthcare Occupations (mixed)*	Denyer et al., 2020[60]	n=5850	Healthcare Practitioners and Technical Occupations and Healthcare Support Occupations*	Cross-sectional survey	05/12 - 05/18	Japan	1.79%	Unclear
Healthcare Occupations (mixed)*	Dimeglio et al., 2020[61]	n=8758	Healthcare Practitioners and Technical Occupations and Healthcare Support Occupations*	Cross-sectional survey	06/10 - 07/10	France	3.2% (2.8-3.5%)	High
Healthcare Occupations (mixed)*	Erber et al., 2020[31]	n=603	Healthcare Practitioners and Technical Occupations and Healthcare Support Occupations*	Cross-sectional survey	04/14 - 05/29	Germany	2.8%	High
Healthcare Occupations (mixed)*	Fuereder et al., 2020[62]	n=62	Healthcare Practitioners and Technical Occupations and Healthcare Support Occupations*	Retrospective cohort	04/01 - 06/04	Austria	3.2% (0.4-11.2%)	High

Healthcare Occupations (mixed)*	Fusco et al., 2020[63]	n=115	Healthcare Practitioners and Technical Occupations and Healthcare Support Occupations*	Cross-sectional survey	03/23 - 04/02	Italy	1.74%	High
Healthcare Occupations (mixed)*	Geraci et al., 2020[64]	n=230	Healthcare Practitioners and Technical Occupations and Healthcare Support Occupations*	Cross-sectional survey	03/16 - 05/20	United States of America	2.17%	High
Healthcare Occupations (mixed)*	Gudo et al., 2020[65]	n=1427	Healthcare Practitioners and Technical Occupations and Healthcare Support Occupations*	Cross-sectional survey	06/17 - 06/30	Mozambique	7% (6-9%)	High
Healthcare Occupations (mixed)*	Hackner et al., 2020[66]	n=130	Healthcare Practitioners and Technical Occupations and Healthcare Support Occupations*	Cross-sectional survey	04/01 - 04/30	Austria	2.3%	High
Healthcare Occupations (mixed)*	Halatoko et al., 2020[41]	n=370	Healthcare Practitioners and Technical Occupations and Healthcare Support Occupations*	Cross-sectional survey	04/23 - 05/08	Togo	1.4%	High
Healthcare Occupations (mixed)*	Haq et al., 2020[67]	n=76	Healthcare Practitioners and Technical Occupations and Healthcare Support Occupations*	Cross-sectional survey	06/15 - 06/29	Pakistan	35.5% (24.8-47.3%)	Moderate
Healthcare Occupations (mixed)*	He et al., 2020[68]	n=1059	Healthcare Practitioners and Technical Occupations and Healthcare Support Occupations*	Repeated cross sectional study	05/13 - 06/10	China	9.3%	High

Healthcare Occupations (mixed)*	Herzberg et al., 2020[69]	n=871	Healthcare Practitioners and Technical Occupations and Healthcare Support Occupations*	Prospective cohort	04/14 - 06/16	Germany	2.64%	High
Healthcare Occupations (mixed)*	Jeremias et al., 2020[70]	n=100	Healthcare Practitioners and Technical Occupations and Healthcare Support Occupations*	Cross-sectional survey	03/01 - 04/30	United States of America	12%	High
Healthcare Occupations (mixed)*	Jespersen et al., 2020[71]	n=17948	Healthcare Practitioners and Technical Occupations and Healthcare Support Occupations*	Cross-sectional survey	05/18 - 06/19	Denmark	3.36% (2.38-3.82%)	Moderate
Healthcare Occupations (mixed)*	Kassem et al., 2020[72]	n=74	Healthcare Practitioners and Technical Occupations and Healthcare Support Occupations*	Cross-sectional survey	06/01 - 06/14	Egypt	12.2%	High
Healthcare Occupations (mixed)*	Kern et al., 2020[73]	n=1316	Healthcare Practitioners and Technical Occupations and Healthcare Support Occupations*	Cross-sectional survey	04/09 - 04/16	Germany	1.06% (0.58-1.78%)	High
Healthcare Occupations (mixed)*	Khalil et al., 2020[74]	n=190	Healthcare Practitioners and Technical Occupations and Healthcare Support Occupations*	Cross-sectional survey	05/15 - 05/28	The United Kingdom	22%	High
Healthcare Occupations (mixed)*	Kumar et al., 2020[75]	n=635	Healthcare Practitioners and Technical Occupations and Healthcare Support Occupations*	Repeated cross sectional study	07/11 - 07/24	India	0%	High

Healthcare Occupations (mixed)*	Lackermair et al., 2020[76]	n=151	Healthcare Practitioners and Technical Occupations and Healthcare Support Occupations*	Cross-sectional survey	04/02 - 04/06	Germany	2.6% (0.8-7.1%)	High
Healthcare Occupations (mixed)*	Lahner et al., 2020[77]	n=1084	Healthcare Practitioners and Technical Occupations and Healthcare Support Occupations*	Cross-sectional survey	04/07 - 04/27	Italy	0.7%	High
Healthcare Occupations (mixed)*	Liu et al., 2020[78]	n=116	Healthcare Practitioners and Technical Occupations and Healthcare Support Occupations*	Cross-sectional survey	02/07 - 04/21	China	0%	High
Healthcare Occupations (mixed)*	Liu et al., 2020[78]	n=304	Healthcare Practitioners and Technical Occupations and Healthcare Support Occupations*	Cross-sectional survey	02/07 - 04/21	China	0%	High
Healthcare Occupations (mixed)*	Liu et al., 2020[79]	n=3832	Healthcare Practitioners and Technical Occupations and Healthcare Support Occupations*	Cross-sectional survey	02/29 - 04/29	China	4% (3.4-4.7%)	Moderate
Healthcare Occupations (mixed)*	Lorenzo et al., 2020[80]	n=38	Healthcare Practitioners and Technical Occupations and Healthcare Support Occupations*	Cross-sectional survey	05/02 - 05/31	Italy	5.3%	High
Healthcare Occupations (mixed)*	Mahomed et al., 2020[81]	n=569	Healthcare Practitioners and Technical Occupations and Healthcare Support Occupations*	Cross-sectional survey	08/31 - 10/12	Mozambique	0.7%	High

Healthcare Occupations (mixed)*	Mahumane et al., 2020[82]	n=380	Healthcare Practitioners and Technical Occupations and Healthcare Support Occupations*	Cross-sectional survey	11/02 - 11/17	Mozambique	1.3%	High
Healthcare Occupations (mixed)*	Majdoubi et al., 2020[83]	n=276	Healthcare Practitioners and Technical Occupations and Healthcare Support Occupations*	Cross-sectional survey	05/17 - 06/19	Canada	0.6% (0-2.71%)	High
Healthcare Occupations (mixed)*	Majiya et al., 2020[84]	n=185	Healthcare Practitioners and Technical Occupations and Healthcare Support Occupations*	Cross-sectional survey	06/26 - 06/30	Nigeria	25.41%	Moderate
Healthcare Occupations (mixed)*	Majiya et al., 2020[84]	n=43	Healthcare Practitioners and Technical Occupations and Healthcare Support Occupations*	Cross-sectional survey	06/26 - 06/30	Nigeria	37.21%	Moderate
Healthcare Occupations (mixed)*	Malfertheiner et al., 2020[85]	n=139	Healthcare Practitioners and Technical Occupations and Healthcare Support Occupations*	Prospective cohort	03/15 - 06/07	Germany	0%	High
Healthcare Occupations (mixed)*	Martin et al., 2020[86]	n=326	Healthcare Practitioners and Technical Occupations and Healthcare Support Occupations*	Cross-sectional survey	04/15 - 05/18	Belgium	11%	High
Healthcare Occupations (mixed)*	Martin et al., 2020[23]	n=4631	Healthcare Practitioners and Technical Occupations and Healthcare Support Occupations*	Cross-sectional survey	05/29 - 07/13	The United Kingdom	13.65%	Moderate

Healthcare Occupations (mixed)*	Melo et al., 2020[87]	n=471	Healthcare Practitioners and Technical Occupations and Healthcare Support Occupations*	Cross-sectional survey	06/01 - 06/30	Brazil	13.59%	High
Healthcare Occupations (mixed)*	Morcuende et al., 2020[88]	n=6	Healthcare Practitioners and Technical Occupations and Healthcare Support Occupations*	Cross-sectional survey	03/01 - 04/21	United States of America	0%	High
Healthcare Occupations (mixed)*	Moscola et al., 2020[89]	n=8156	Healthcare Practitioners and Technical Occupations and Healthcare Support Occupations*	Cross-sectional survey	04/20 - 06/23	United States of America	11.6%	High
Healthcare Occupations (mixed)*	Nishida et al., 2020[90]	n=49	Healthcare Practitioners and Technical Occupations and Healthcare Support Occupations*	Cross-sectional survey	06/12 - 06/19	Japan	0%	Moderate
Healthcare Occupations (mixed)*	Olalla et al., 2020[91]	n=498	Healthcare Practitioners and Technical Occupations and Healthcare Support Occupations*	Cross-sectional survey	04/15 - 04/25	Spain	2.2%	High
Healthcare Occupations (mixed)*	Pallett et al., 2020[92]	n=504	Healthcare Practitioners and Technical Occupations and Healthcare Support Occupations*	Prospective cohort	04/08 - 06/12	The United Kingdom	10.6% (7.6-13.6%)	High
Healthcare Occupations (mixed)*	Pere et al., 2020[93]	n=3569	Healthcare Practitioners and Technical Occupations and Healthcare Support Occupations*	Cross-sectional survey	05/02 - 06/26	France	11.9%	High

Healthcare Occupations (mixed)*	Poulikakos et al., 2020[94]	n=281	Healthcare Practitioners and Technical Occupations and Healthcare Support Occupations*	Cross-sectional survey	05/04 - 05/06	The United Kingdom	6%	High
Healthcare Occupations (mixed)*	Psichogiou et al., 2020[95]	n=1495	Healthcare Practitioners and Technical Occupations and Healthcare Support Occupations*	Cross-sectional survey	04/13 - 05/15	Greece	1.26% (0.43-3.26%)	Moderate
Healthcare Occupations (mixed)*	Satpati et al., 2020[27]	n=18	Healthcare Practitioners and Technical Occupations and Healthcare Support Occupations*	Cross-sectional survey	07/26 - 08/08	India	5.56%	Moderate
Healthcare Occupations (mixed)*	Seetharam et al., 2020[96]	n=728	Healthcare Practitioners and Technical Occupations and Healthcare Support Occupations*	Cross-sectional survey	08/16 - 08/29	India	27.3% (24.1-30.6%)	Unclear
Healthcare Occupations (mixed)*	Shakiba et al., 2020[10]	n=43	Healthcare Practitioners and Technical Occupations and Healthcare Support Occupations*	Cross-sectional survey	04/11 - 04/19	Iran (Islamic Republic of)	14.5% (4.5-25%)	Moderate
Healthcare Occupations (mixed)*	Shields et al., 2020[97]	n=516	Healthcare Practitioners and Technical Occupations and Healthcare Support Occupations*	Cross-sectional survey	04/24 - 04/25	The United Kingdom	24.4%	High
Healthcare Occupations (mixed)*	Silva et al., 2020[98]	n=61	Healthcare Practitioners and Technical Occupations and Healthcare Support Occupations*	Cross-sectional survey	04/09 - 04/29	Brazil	4.91%	High

Healthcare Occupations (mixed)*	Solodky et al., 2020[99]	n=85	Healthcare Practitioners and Technical Occupations and Healthcare Support Occupations*	Cross-sectional survey	03/01 - 04/16	France	5.88%	High
Healthcare Occupations (mixed)*	Soriano et al., 2020[100]	n=108	Healthcare Practitioners and Technical Occupations and Healthcare Support Occupations*	Retrospective cohort	04/26 - 05/16	Spain	13%	High
Healthcare Occupations (mixed)*	Statistica et al., 2020[101]	n=64660	Healthcare Practitioners and Technical Occupations and Healthcare Support Occupations*	Cross-sectional survey	05/25 - 07/15	Italy	2.5%	Unclear
Healthcare Occupations (mixed)*	Steensels et al., 2020[102]	n=3056	Healthcare Practitioners and Technical Occupations and Healthcare Support Occupations*	Cross-sectional survey	04/22 - 04/30	Belgium	6.4% (5.5-7.3%)	High
Healthcare Occupations (mixed)*	Stock et al., 2020[103]	n=98	Healthcare Practitioners and Technical Occupations and Healthcare Support Occupations*	Cross-sectional survey	04/04 - 04/20	United States of America	15.3%	High
Healthcare Occupations (mixed)*	Takita et al., 2020[104]	n=175	Healthcare Practitioners and Technical Occupations and Healthcare Support Occupations*	Cross-sectional survey	04/20 - 05/20	Japan	4% (1.62-8.07%)	High
Healthcare Occupations (mixed)*	Tong et al., 2020[105]	n=191	Healthcare Practitioners and Technical Occupations and Healthcare Support Occupations*	Cross-sectional survey	05/12 - 05/15	China	0%	High

Healthcare Occupations (mixed)*	Trieu et al., 2020[106]	n=607	Healthcare Practitioners and Technical Occupations and Healthcare Support Occupations*	Prospective cohort	03/06 - 04/09	Norway	5.27%	High
Healthcare Occupations (mixed)*	Tu et al., 2020[107]	n=325	Healthcare Practitioners and Technical Occupations and Healthcare Support Occupations*	Cross sectional study with prospective cohort follow up of a subset of the sample	03/19 - 03/20	China	43.08%	High
Healthcare Occupations (mixed)*	Valdivia et al., 2020[108]	n=1153	Healthcare Practitioners and Technical Occupations and Healthcare Support Occupations*	Cross-sectional survey	04/13 - 04/30	Spain	3.5%	High
Healthcare Occupations (mixed)*	Vasquez et al., 2020[109]	n=1147	Healthcare Practitioners and Technical Occupations and Healthcare Support Occupations*	Cross-sectional survey	05/19 - 06/06	Peru	58.3%	High
Healthcare Occupations (mixed)*	Viegas et al., 2020[110]	n=1443	Healthcare Practitioners and Technical Occupations and Healthcare Support Occupations*	Cross-sectional survey	08/03 - 08/21	Mozambique	2.63%	High
Healthcare Occupations (mixed)*	Vlachoyiannopoulosa et al., 2020[111]	n=321	Healthcare Practitioners and Technical Occupations and Healthcare Support Occupations*	Cross-sectional survey	04/25 - 05/10	Greece	2.18%	High
Healthcare Occupations (mixed)*	Volta et al., 2020[112]	n=76	Healthcare Practitioners and Technical Occupations and Healthcare Support Occupations*	Cross-sectional survey	04/27 - 04/27	Italy	11.8%	High

Healthcare Occupations (mixed)*	Ward et al., 2020[113]	n=5416	Healthcare Practitioners and Technical Occupations and Healthcare Support Occupations*	Cross-sectional survey	09/15 - 09/28	The United Kingdom	10.67%	Moderate
Healthcare Occupations (mixed)*	Ward et al., 2020[113]	n=1692	Healthcare Practitioners and Technical Occupations and Healthcare Support Occupations*	Cross-sectional survey	09/15 - 09/28	The United Kingdom	6.68%	Moderate
Healthcare Occupations (mixed)*	Xiong et al., 2020[114]	n=797	Healthcare Practitioners and Technical Occupations and Healthcare Support Occupations*	Cross-sectional survey	02/12 - 03/17	China	4.39%	Unclear
Healthcare Occupations (mixed)*	Zhang et al., 2020[115]	n=63	Healthcare Practitioners and Technical Occupations and Healthcare Support Occupations*	Cross-sectional survey	01/21 - 02/16	China	0%	High
Healthcare Occupations (mixed)*	Zhao et al., 2020[116]	n=1060	Healthcare Practitioners and Technical Occupations and Healthcare Support Occupations*	Cross-sectional survey	01/14 - 02/21	China	8.3%	High
First responders (mixed)*	Ahmad et al., 2020[117]	n=40	Healthcare Practitioners and Technical Occupations and Protective Service Occupations (i.e. first responders)*	Cross-sectional survey	04/21 - 05/22	United States of America	20%	High
First responders (mixed)*	Halbrook et al., 2020[118]	n=679	Healthcare Practitioners and Technical Occupations and Protective Service Occupations (i.e. first responders)*	Cross-sectional survey	05/19 - 08/31	United States of America	8.1%	Moderate

First responders (mixed)*	Iwuji et al., 2020[119]	n=683	Healthcare Practitioners and Technical Occupations and Protective Service Occupations (i.e. first responders)*	Cross-sectional survey	05/12 - 05/13	United States of America	0.7%	High
First responders (mixed)*	Magyar et al., 2020[120]	n=70	Healthcare Practitioners and Technical Occupations and Protective Service Occupations (i.e. first responders)*	Cross-sectional survey	05/01 - 05/14	United States of America	4.29%	High
First responders (mixed)*	Martinez et al., 2020[121]	n=79	Healthcare Practitioners and Technical Occupations and Protective Service Occupations (i.e. first responders)*	Cross-sectional survey	04/16 - 04/17	United States of America	5.06%	High
First responders (mixed)*	Staletovich et al., 2020[122]	n=359	Healthcare Practitioners and Technical Occupations and Protective Service Occupations (i.e. first responders)*	Cross-sectional survey	05/17 - 05/22	United States of America	0%	Unclear
Healthcare Practitioners and Technical Occupations (29-0000)	Hibino et al., 2020[123]	n=806	Health Diagnosing and Treating Practitioners	Cross-sectional survey	06/01 - 07/30	Japan	0.74% (0.27-1.61%)	Unclear
Healthcare Practitioners and Technical Occupations (29-0000)	Jones et al., 2020[29]	n=856	Dentists, General	Cross-sectional survey	01/15 - 06/15	The United Kingdom	7.9%	High
Life, Physical, and Social Science	Calcagno et al., 2020[124]	n=343	Life, Physical, and Social Science Occupations	Cross-sectional survey	04/17 - 05/20	Italy	6.71%	Moderate

Occupations (19-0000)								
Healthcare Practitioners and Technical Occupations (29-0000)	Goenka et al., 2020[25]	n=49	Dietitians and Nutritionists	Cross-sectional survey	07/12 - 08/23	India	18.37%	Moderate
Healthcare Practitioners and Technical Occupations (29-0000)	Goenka et al., 2020[26]	n=6	Dietitians and Nutritionists	Cross-sectional survey	08/01 - 08/31	India	0%	High
Healthcare Practitioners and Technical Occupations (29-0000)	Akinbami et al., 2020[46]	n=321	Pharmacists	Cross-sectional survey	05/18 - 06/13	United States of America	4.4% (2.4-7.2%)	Moderate
Healthcare Practitioners and Technical Occupations (29-0000)	Alharbi et al., 2020[125]	n=5	Pharmacists	Cross-sectional survey	04/18 - 06/17	Saudi Arabia	0%	High
Healthcare Practitioners and Technical Occupations (29-0000)	Calcagno et al., 2020[124]	n=29	Pharmacists	Cross-sectional survey	04/17 - 05/20	Italy	3.45%	Moderate
Healthcare Practitioners and Technical Occupations (29-0000)	Chau et al., 2020[126]	n=17	Pharmacists	Cross-sectional survey	08/23 - 08/30	Viet Nam	0%	High
Healthcare Practitioners and Technical Occupations (29-0000)	Hanrath et al., 2020[32]	n=189	Pharmacists	Cross-sectional survey	05/29 - 07/06	The United Kingdom	4.76%	High

Healthcare Practitioners and Technical Occupations (29-0000)	Khan et al., 2020[127]	n=109	Pharmacists	Cross-sectional survey	06/15 - 06/29	India	0%	Moderate
Healthcare Practitioners and Technical Occupations (29-0000)	Mahomed et al., 2020[81]	n=404	Pharmacists	Cross-sectional survey	08/31 - 10/12	Mozambique	0.5%	High
Healthcare Practitioners and Technical Occupations (29-0000)	Martin et al., 2020[23]	n=113	Pharmacists	Cross-sectional survey	05/29 - 07/13	The United Kingdom	0%	Moderate
Healthcare Practitioners and Technical Occupations (29-0000)	Rosser et al., 2020[33]	n=213	Pharmacists	Cross-sectional survey	04/20 - 05/20	United States of America	1.88%	High
Healthcare Practitioners and Technical Occupations (29-0000)	Costa et al., 2020[128]	n=652	Physicians and Surgeons	Cross-sectional survey	05/14 - 05/28	Brazil	5.8%	Moderate
Healthcare Practitioners and Technical Occupations (29-0000)	Mohr et al., 2020[129]	n=372	Physicians and Surgeons	Cross-sectional survey	05/13 - 07/08	United States of America	1.61%	Moderate
Healthcare Practitioners and Technical Occupations (29-0000)	Nishida et al., 2020[90]	n=63	Physicians and Surgeons	Cross-sectional survey	06/12 - 06/19	Japan	3.2% (0.88-11%)	Moderate
Healthcare Practitioners and	Noor et al., 2020[130]	n=157	Physicians and Surgeons	Cross-sectional survey	07/13 - 07/15	Pakistan	17.83%	Moderate

Technical Occupations (29-0000)								
Healthcare Practitioners and Technical Occupations (29-0000)	Singhal et al., 2020[131]	n=208	Physicians and Surgeons	Cross-sectional survey	06/01 - 06/30	India	12.5%	High
Healthcare Practitioners and Technical Occupations (29-0000)	Morcuende et al., 2020[88]	n=23	Anesthesiologists	Cross-sectional survey	03/01 - 04/21	United States of America	13.04%	High
Healthcare Practitioners and Technical Occupations (29-0000)	Morcuende et al., 2020[88]	n=3	Obstetricians and Gynecologists	Cross-sectional survey	03/01 - 04/21	United States of America	100%	High
Healthcare Practitioners and Technical Occupations (29-0000)	Urbieta et al., 2020[132]	n=23	Pediatricians, General	Cross-sectional survey	04/14 - 04/16	Spain	4.3%	High
Healthcare Practitioners and Technical Occupations (29-0000)	Iversen et al., 2020[8]	n=1944	Psychiatrists	Cross-sectional survey	04/15 - 04/22	Denmark	1.85%	Low
Healthcare Practitioners and Technical Occupations (29-0000)	Leidner et al., 2020[22]	n=301	Surgeons	Cross sectional study with prospective cohort follow up of a subset of the sample	04/08 - 05/22	United States of America	2.66%	High
Healthcare Practitioners and Technical	Akinbami et al., 2020[46]	n=2297	Physicians and Surgeons, All Other	Cross-sectional survey	05/18 - 06/13	United States of America	6.1% (5.1-7.1%)	Moderate

Occupations (29-0000)								
Healthcare Practitioners and Technical Occupations (29-0000)	Alharbi et al., 2020[125]	n=18	Physicians and Surgeons, All Other	Cross-sectional survey	04/18 - 06/17	Saudi Arabia	27.78%	High
Healthcare Practitioners and Technical Occupations (29-0000)	Amendola et al., 2020[47]	n=214	Physicians and Surgeons, All Other	Cross-sectional survey	04/15 - 04/15	Italy	4.67%	High
Healthcare Practitioners and Technical Occupations (29-0000)	Baracco et al., 2020[24]	n=417	Physicians and Surgeons, All Other	Cross-sectional survey	04/23 - 05/05	Italy	17%	High
Healthcare Practitioners and Technical Occupations (29-0000)	Barallat et al., 2020[50]	n=1821	Physicians and Surgeons, All Other	Cross-sectional survey	05/04 - 05/22	Spain	11.81%	High
Healthcare Practitioners and Technical Occupations (29-0000)	Bianchi et al., 2020[133]	n=34	Physicians and Surgeons, All Other	Cross-sectional survey	04/15 - 05/15	Italy	5.88%	Unclear
Healthcare Practitioners and Technical Occupations (29-0000)	Blairon et al., 2020[52]	n=323	Physicians and Surgeons, All Other	Cross-sectional survey	05/25 - 06/19	Belgium	11.8%	High
Healthcare Practitioners and Technical Occupations (29-0000)	Brehm et al., 2020[7]	n=275	Physicians and Surgeons, All Other	Cross sectional study with prospective cohort follow up of a	03/20 - 07/17	Germany	3.3%	Moderate

				subset of the sample				
Healthcare Practitioners and Technical Occupations (29-0000)	Brousseau et al., 2020[134]	n=432	Physicians and Surgeons, All Other	Cross-sectional survey	07/06 - 09/24	Canada	7.2%	High
Healthcare Practitioners and Technical Occupations (29-0000)	Calcagno et al., 2020[124]	n=700	Physicians and Surgeons, All Other	Cross-sectional survey	04/17 - 05/20	Italy	7.86%	Moderate
Healthcare Practitioners and Technical Occupations (29-0000)	Chau et al., 2020[126]	n=64	Physicians and Surgeons, All Other	Cross-sectional survey	08/23 - 08/30	Viet Nam	0%	High
Healthcare Practitioners and Technical Occupations (29-0000)	Chen et al., 2020[135]	n=17	Physicians and Surgeons, All Other	Cross-sectional survey	02/19 - 02/19	China	41.18%	High
Healthcare Practitioners and Technical Occupations (29-0000)	Erber et al., 2020[31]	n=860	Physicians and Surgeons, All Other	Cross-sectional survey	04/14 - 05/29	Germany	1.63%	High
Healthcare Practitioners and Technical Occupations (29-0000)	Favara et al., 2020[136]	n=15	Physicians and Surgeons, All Other	Prospective cohort	06/01 - 06/07	The United Kingdom	13.33%	High
Healthcare Practitioners and Technical Occupations (29-0000)	Favara et al., 2020[19]	n=82	Physicians and Surgeons, All Other	Cross-sectional survey	07/13 - 07/13	The United Kingdom	10.9%	High

Healthcare Practitioners and Technical Occupations (29-0000)	Fujita et al., 2020[137]	n=42	Physicians and Surgeons, All Other	Cross-sectional survey	04/10 - 04/20	Japan	4.7%	High
Healthcare Practitioners and Technical Occupations (29-0000)	Galan et al., 2020[20]	n=564	Physicians and Surgeons, All Other	Cross-sectional survey	04/14 - 04/27	Spain	39.36%	High
Healthcare Practitioners and Technical Occupations (29-0000)	Godbout et al., 2020[138]	n=490	Physicians and Surgeons, All Other	Cross-sectional survey	07/27 - 10/02	United States of America	1.43%	High
Healthcare Practitioners and Technical Occupations (29-0000)	Goenka et al., 2020[25]	n=255	Physicians and Surgeons, All Other	Cross-sectional survey	07/12 - 08/23	India	3.92%	Moderate
Healthcare Practitioners and Technical Occupations (29-0000)	Goenka et al., 2020[26]	n=29	Physicians and Surgeons, All Other	Cross-sectional survey	08/01 - 08/31	India	20.69%	High
Healthcare Practitioners and Technical Occupations (29-0000)	Hanrath et al., 2020[32]	n=899	Physicians and Surgeons, All Other	Cross-sectional survey	05/29 - 07/06	The United Kingdom	7.01%	High
Healthcare Practitioners and Technical Occupations (29-0000)	Houlihan et al., 2020[139]	n=72	Physicians and Surgeons, All Other	Cross-sectional survey	03/26 - 04/08	The United Kingdom	22%	High
Healthcare Practitioners and	Hunter et al., 2020[21]	n=279	Physicians and Surgeons, All Other	Cross-sectional survey	04/29 - 05/08	United States of America	1.08%	High

Technical Occupations (29-0000)								
Healthcare Practitioners and Technical Occupations (29-0000)	Insua et al., 2020[140]	n=116	Physicians and Surgeons, All Other	Cross-sectional survey	06/08 - 06/09	Argentina	0.9% (0.1-5.5%)	High
Healthcare Practitioners and Technical Occupations (29-0000)	Iversen et al., 2020[8]	n=4698	Physicians and Surgeons, All Other	Cross-sectional survey	04/15 - 04/22	Denmark	4.07%	Low
Healthcare Practitioners and Technical Occupations (29-0000)	Iversen et al., 2020[8]	n=113	Physicians and Surgeons, All Other	Cross-sectional survey	04/15 - 04/22	Denmark	7.08%	Low
Healthcare Practitioners and Technical Occupations (29-0000)	Jeremias et al., 2020[70]	n=79	Physicians and Surgeons, All Other	Cross-sectional survey	03/01 - 04/30	United States of America	11.4%	High
Healthcare Practitioners and Technical Occupations (29-0000)	Kassem et al., 2020[72]	n=30	Physicians and Surgeons, All Other	Cross-sectional survey	06/01 - 06/14	Egypt	6.66%	High
Healthcare Practitioners and Technical Occupations (29-0000)	Kassem et al., 2020[72]	n=30	Physicians and Surgeons, All Other	Cross-sectional survey	06/01 - 06/14	Egypt	3.33%	High
Healthcare Practitioners and Technical	Kassem et al., 2020[72]	n=30	Physicians and Surgeons, All Other	Cross-sectional survey	06/01 - 06/14	Egypt	0%	High

Occupations (29-0000)								
Healthcare Practitioners and Technical Occupations (29-0000)	Kassem et al., 2020[72]	n=30	Physicians and Surgeons, All Other	Cross-sectional survey	06/01 - 06/14	Egypt	3.33%	High
Healthcare Practitioners and Technical Occupations (29-0000)	Khan et al., 2020[127]	n=980	Physicians and Surgeons, All Other	Cross-sectional survey	06/15 - 06/29	India	2.8% (1.9-4%)	Moderate
Healthcare Practitioners and Technical Occupations (29-0000)	Kohler et al., 2020[141]	n=268	Physicians and Surgeons, All Other	Cross-sectional survey	03/19 - 04/03	Switzerland	1.49%	High
Healthcare Practitioners and Technical Occupations (29-0000)	Kumar et al., 2020[142]	n=201	Physicians and Surgeons, All Other	Cross-sectional survey	06/01 - 06/30	India	7% (4.2-11.4%)	High
Healthcare Practitioners and Technical Occupations (29-0000)	Leidner et al., 2020[22]	n=1081	Physicians and Surgeons, All Other	Cross sectional study with prospective cohort follow up of a subset of the sample	04/08 - 05/22	United States of America	3.33%	High
Healthcare Practitioners and Technical Occupations (29-0000)	Lumley et al., 2020[9]	n=1859	Physicians and Surgeons, All Other	Prospective cohort	04/23 - 11/30	The United Kingdom	10.11%	Moderate
Healthcare Practitioners and Technical	Martin et al., 2020[23]	n=1243	Physicians and Surgeons, All Other	Cross-sectional survey	05/29 - 07/13	The United Kingdom	10.3%	Moderate

Occupations (29-0000)								
Healthcare Practitioners and Technical Occupations (29-0000)	Mesnil et al., 2020[143]	n=111	Physicians and Surgeons, All Other	Cross-sectional survey	06/08 - 06/22	France	11%	High
Healthcare Practitioners and Technical Occupations (29-0000)	Missaglia et al., 2020[144]	n=377	Physicians and Surgeons, All Other	Cross-sectional survey	04/01 - 04/30	Italy	14.9%	High
Healthcare Practitioners and Technical Occupations (29-0000)	Mohr et al., 2020[129]	n=272	Physicians and Surgeons, All Other	Cross-sectional survey	05/13 - 07/08	United States of America	2.94%	Moderate
Healthcare Practitioners and Technical Occupations (29-0000)	Moscola et al., 2020[89]	n=3746	Physicians and Surgeons, All Other	Cross-sectional survey	04/20 - 06/23	United States of America	8.7%	High
Healthcare Practitioners and Technical Occupations (29-0000)	Nishida et al., 2020[90]	n=149	Physicians and Surgeons, All Other	Cross-sectional survey	06/12 - 06/19	Japan	1.3% (0.37-4.8%)	Moderate
Healthcare Practitioners and Technical Occupations (29-0000)	Nishida et al., 2020[90]	n=46	Physicians and Surgeons, All Other	Cross-sectional survey	06/12 - 06/19	Japan	0%	Moderate
Healthcare Practitioners and Technical Occupations (29-0000)	Nishida et al., 2020[90]	n=40	Physicians and Surgeons, All Other	Cross-sectional survey	06/12 - 06/19	Japan	0%	Moderate

Healthcare Practitioners and Technical Occupations (29-0000)	Nishida et al., 2020[90]	n=59	Physicians and Surgeons, All Other	Cross-sectional survey	06/12 - 06/19	Japan	1.7% (0.3-9%)	Moderate
Healthcare Practitioners and Technical Occupations (29-0000)	Nishida et al., 2020[90]	n=925	Physicians and Surgeons, All Other	Cross-sectional survey	06/12 - 06/19	Japan	0.43% (0.17-1.1%)	Moderate
Healthcare Practitioners and Technical Occupations (29-0000)	Noor et al., 2020[130]	n=303	Physicians and Surgeons, All Other	Cross-sectional survey	07/13 - 07/15	Pakistan	19.8%	Moderate
Healthcare Practitioners and Technical Occupations (29-0000)	Orth-Holler et al., 2020[145]	n=377	Physicians and Surgeons, All Other	Cross-sectional survey	03/20 - 03/27	Austria	0.3% (0.01-1.5%)	High
Healthcare Practitioners and Technical Occupations (29-0000)	Plebani et al., 2020[146]	n=2337	Physicians and Surgeons, All Other	Cross-sectional survey	02/22 - 05/29	Italy	3.6% (2.8-4.4%)	High
Healthcare Practitioners and Technical Occupations (29-0000)	Rosser et al., 2020[33]	n=2533	Physicians and Surgeons, All Other	Cross-sectional survey	04/20 - 05/20	United States of America	1.07%	High
Healthcare Practitioners and Technical Occupations (29-0000)	Rudberg et al., 2020[147]	n=439	Physicians and Surgeons, All Other	Cross-sectional survey	04/14 - 05/08	Sweden	19.1%	Moderate
Healthcare Practitioners and	Schmidt et al., 2020[148]	n=34	Physicians and Surgeons, All Other	Cross-sectional survey	04/20 - 04/30	Germany	8.82%	High

Technical Occupations (29-0000)								
Healthcare Practitioners and Technical Occupations (29-0000)	Sotgiu et al., 2020[149]	n=115	Physicians and Surgeons, All Other	Cross-sectional survey	04/02 - 04/16	Italy	6.09%	High
Healthcare Practitioners and Technical Occupations (29-0000)	Venugopal et al., 2020[150]	n=157	Physicians and Surgeons, All Other	Cross-sectional survey	03/01 - 05/01	United States of America	25%	Moderate
Healthcare Practitioners and Technical Occupations (29-0000)	Yogo et al., 2020[36]	n=110	Physicians and Surgeons, All Other	Cross-sectional survey	05/20 - 06/08	United States of America	1.82%	High
Healthcare Practitioners and Technical Occupations (29-0000)	Brzostek et al., 2020[151]	n=998	Physician Assistants	Cross-sectional survey	04/17 - 05/07	United States of America	28.3%	Moderate
Healthcare Practitioners and Technical Occupations (29-0000)	Hoffmann et al., 2020[152]	n=156	Physician Assistants	Prospective cohort	07/01 - 07/31	Germany	1.3%	High
Healthcare Practitioners and Technical Occupations (29-0000)	Mohr et al., 2020[129]	n=156	Physician Assistants	Cross-sectional survey	05/13 - 07/08	United States of America	0.64%	Moderate
Healthcare Practitioners and Technical	Morcuende et al., 2020[88]	n=6	Physician Assistants	Cross-sectional survey	03/01 - 04/21	United States of America	9.43%	High

Occupations (29-0000)								
Healthcare Practitioners and Technical Occupations (29-0000)	Morcuende et al., 2020[88]	n=53	Physician Assistants	Cross-sectional survey	03/01 - 04/21	United States of America	9.43%	High
Healthcare Practitioners and Technical Occupations (29-0000)	Patel et al., 2020[153]	n=230	Physician Assistants	Prospective cohort	06/02 - 06/27	United States of America	3.48%	High
Healthcare Practitioners and Technical Occupations (29-0000)	Self et al., 2020[154]	n=919	Physician Assistants	Cross-sectional survey	04/03 - 06/19	United States of America	5.66%	Moderate
Healthcare Practitioners and Technical Occupations (29-0000)	Shah et al., 2020[155]	n=248	Physician Assistants	Cross-sectional survey	05/25 - 07/09	United States of America	0%	High
Healthcare Practitioners and Technical Occupations (29-0000)	Shah et al., 2020[155]	n=320	Physician Assistants	Cross-sectional survey	05/25 - 07/09	United States of America	0.63%	High
Healthcare Practitioners and Technical Occupations (29-0000)	Lumley et al., 2020[9]	n=386	Occupational Therapists	Prospective cohort	04/23 - 11/30	The United Kingdom	11.4%	Moderate
Healthcare Practitioners and Technical Occupations (29-0000)	Akinbami et al., 2020[46]	n=235	Physical Therapists	Cross-sectional survey	05/18 - 06/13	United States of America	10.6% (7-15.3%)	Moderate

Healthcare Practitioners and Technical Occupations (29-0000)	Brehm et al., 2020[7]	n=15	Physical Therapists	Cross sectional study with prospective cohort follow up of a subset of the sample	03/20 - 07/17	Germany	0%	Moderate
Healthcare Practitioners and Technical Occupations (29-0000)	Cooper et al., 2020[59]	n=84	Physical Therapists	Cross-sectional survey	06/10 - 08/07	The United Kingdom	10.71%	Moderate
Healthcare Practitioners and Technical Occupations (29-0000)	Costa et al., 2020[128]	n=159	Physical Therapists	Cross-sectional survey	05/14 - 05/28	Brazil	10.7%	Moderate
Healthcare Practitioners and Technical Occupations (29-0000)	Akinbami et al., 2020[46]	n=409	Respiratory Therapists	Cross-sectional survey	05/18 - 06/13	United States of America	8.3% (5.8-11.4%)	Moderate
Healthcare Practitioners and Technical Occupations (29-0000)	Brunner et al., 2020[54]	n=42	Respiratory Therapists	Cross-sectional survey	05/04 - 05/29	United States of America	0%	High
Healthcare Practitioners and Technical Occupations (29-0000)	Godbout et al., 2020[138]	n=25	Respiratory Therapists	Cross-sectional survey	07/27 - 10/02	United States of America	0%	High
Healthcare Practitioners and Technical Occupations (29-0000)	Hunter et al., 2020[21]	n=94	Respiratory Therapists	Cross-sectional survey	04/29 - 05/08	United States of America	0%	High

Healthcare Practitioners and Technical Occupations (29-0000)	Rosser et al., 2020[33]	n=135	Respiratory Therapists	Cross-sectional survey	04/20 - 05/20	United States of America	0%	High
Healthcare Practitioners and Technical Occupations (29-0000)	Self et al., 2020[154]	n=235	Respiratory Therapists	Cross-sectional survey	04/03 - 06/19	United States of America	4.26%	Moderate
Healthcare Practitioners and Technical Occupations (29-0000)	Yogo et al., 2020[36]	n=121	Respiratory Therapists	Cross-sectional survey	05/20 - 06/08	United States of America	0%	High
Healthcare Practitioners and Technical Occupations (29-0000)	Rosser et al., 2020[33]	n=253	Therapists, All Other	Cross-sectional survey	04/20 - 05/20	United States of America	1.58%	High
Healthcare Practitioners and Technical Occupations (29-0000)	Schmidt et al., 2020[148]	n=80	Therapists, All Other	Cross-sectional survey	04/20 - 04/30	Germany	3.75%	High
Healthcare Practitioners and Technical Occupations (29-0000)	Yogo et al., 2020[36]	n=22	Therapists, All Other	Cross-sectional survey	05/20 - 06/08	United States of America	4.55%	High
Healthcare Practitioners and Technical Occupations (29-0000)	Calcagno et al., 2020[124]	n=13	Veterinarians	Cross-sectional survey	04/17 - 05/20	Italy	0%	Moderate
Healthcare Practitioners and	Akinbami et al., 2020[46]	n=6426	Registered Nurses	Cross-sectional survey	05/18 - 06/13	United States of America	7.7% (7.1-8.4%)	Moderate

Technical Occupations (29-0000)								
Healthcare Practitioners and Technical Occupations (29-0000)	Alharbi et al., 2020[125]	n=70	Registered Nurses	Cross-sectional survey	04/18 - 06/17	Saudi Arabia	10%	High
Healthcare Practitioners and Technical Occupations (29-0000)	Alharbi et al., 2020[125]	n=9	Registered Nurses	Cross-sectional survey	04/18 - 06/17	Saudi Arabia	33.33%	High
Healthcare Practitioners and Technical Occupations (29-0000)	Alharbi et al., 2020[125]	n=76	Registered Nurses	Cross-sectional survey	04/18 - 06/17	Saudi Arabia	26.32%	High
Healthcare Practitioners and Technical Occupations (29-0000)	Alharbi et al., 2020[125]	n=21	Registered Nurses	Cross-sectional survey	04/18 - 06/17	Saudi Arabia	14.29%	High
Healthcare Practitioners and Technical Occupations (29-0000)	Alharbi et al., 2020[125]	n=43	Registered Nurses	Cross-sectional survey	04/18 - 06/17	Saudi Arabia	27.91%	High
Healthcare Practitioners and Technical Occupations (29-0000)	Amendola et al., 2020[47]	n=216	Registered Nurses	Cross-sectional survey	04/15 - 04/15	Italy	6.02%	High
Healthcare Practitioners and Technical	Bampoe et al., 2020[156]	n=52	Registered Nurses	Cross-sectional survey	05/11 - 06/05	The United Kingdom	13.5% (5.6-25.8%)	High

Occupations (29-0000)								
Healthcare Practitioners and Technical Occupations (29-0000)	Bampoe et al., 2020[156]	n=40	Registered Nurses	Cross-sectional survey	05/11 - 06/05	The United Kingdom	12.5% (4.2-26.8%)	High
Healthcare Practitioners and Technical Occupations (29-0000)	Baracco et al., 2020[24]	n=1014	Registered Nurses	Cross-sectional survey	04/23 - 05/05	Italy	17.9%	High
Healthcare Practitioners and Technical Occupations (29-0000)	Barallat et al., 2020[50]	n=2243	Registered Nurses	Cross-sectional survey	05/04 - 05/22	Spain	10.64%	High
Healthcare Practitioners and Technical Occupations (29-0000)	Brehm et al., 2020[7]	n=444	Registered Nurses	Cross sectional study with prospective cohort follow up of a subset of the sample	03/20 - 07/17	Germany	2.3%	Moderate
Healthcare Practitioners and Technical Occupations (29-0000)	Brousseau et al., 2020[134]	n=1189	Registered Nurses	Cross-sectional survey	07/06 - 09/24	Canada	11.9%	High
Healthcare Practitioners and Technical Occupations (29-0000)	Calcagno et al., 2020[124]	n=1833	Registered Nurses	Cross-sectional survey	04/17 - 05/20	Italy	8.18%	Moderate
Healthcare Practitioners and Technical	Chau et al., 2020[126]	n=144	Registered Nurses	Cross-sectional survey	08/23 - 08/30	Viet Nam	0%	High

Occupations (29-0000)								
Healthcare Practitioners and Technical Occupations (29-0000)	Chen et al., 2020[135]	n=25	Registered Nurses	Cross-sectional survey	02/19 - 02/19	China	8%	High
Healthcare Practitioners and Technical Occupations (29-0000)	Cooper et al., 2020[59]	n=3471	Registered Nurses	Cross-sectional survey	06/10 - 08/07	The United Kingdom	7.52%	Moderate
Healthcare Practitioners and Technical Occupations (29-0000)	Costa et al., 2020[128]	n=370	Registered Nurses	Cross-sectional survey	05/14 - 05/28	Brazil	11.4%	Moderate
Healthcare Practitioners and Technical Occupations (29-0000)	Dimcheff et al., 2020[157]	n=412	Registered Nurses	Cross-sectional survey	06/08 - 07/08	United States of America	7%	Moderate
Healthcare Practitioners and Technical Occupations (29-0000)	Erber et al., 2020[31]	n=958	Registered Nurses	Cross-sectional survey	04/14 - 05/29	Germany	2.5%	High
Healthcare Practitioners and Technical Occupations (29-0000)	Favara et al., 2020[136]	n=45	Registered Nurses	Prospective cohort	06/01 - 06/07	The United Kingdom	28.89%	High
Healthcare Practitioners and Technical Occupations (29-0000)	Favara et al., 2020[19]	n=237	Registered Nurses	Cross-sectional survey	07/13 - 07/13	The United Kingdom	16.5%	High

Healthcare Practitioners and Technical Occupations (29-0000)	Finkenzeller et al., 2020[158]	n=251	Registered Nurses	Prospective cohort	06/29 - 07/29	Germany	12%	Moderate
Healthcare Practitioners and Technical Occupations (29-0000)	Finkenzeller et al., 2020[158]	n=887	Registered Nurses	Prospective cohort	06/29 - 07/29	Germany	20%	Moderate
Healthcare Practitioners and Technical Occupations (29-0000)	Fujita et al., 2020[137]	n=50	Registered Nurses	Cross-sectional survey	04/10 - 04/20	Japan	6%	High
Healthcare Practitioners and Technical Occupations (29-0000)	Galan et al., 2020[20]	n=687	Registered Nurses	Cross-sectional survey	04/14 - 04/27	Spain	30.71%	High
Healthcare Practitioners and Technical Occupations (29-0000)	Godbout et al., 2020[138]	n=937	Registered Nurses	Cross-sectional survey	07/27 - 10/02	United States of America	1.39%	High
Healthcare Practitioners and Technical Occupations (29-0000)	Goenka et al., 2020[25]	n=224	Registered Nurses	Cross-sectional survey	07/12 - 08/23	India	9.38%	Moderate
Healthcare Practitioners and Technical Occupations (29-0000)	Goenka et al., 2020[26]	n=43	Registered Nurses	Cross-sectional survey	08/01 - 08/31	India	34.88%	High
Healthcare Practitioners and	Grant et al., 2020[159]	n=1345	Registered Nurses	Cross-sectional survey	05/15 - 06/05	The United Kingdom	34.7%	High

Technical Occupations (29-0000)								
Healthcare Practitioners and Technical Occupations (29-0000)	Grant et al., 2020[159]	n=108	Registered Nurses	Cross-sectional survey	05/15 - 06/05	The United Kingdom	25%	High
Healthcare Practitioners and Technical Occupations (29-0000)	Hanrath et al., 2020[32]	n=749	Registered Nurses	Cross-sectional survey	05/29 - 07/06	The United Kingdom	8.99%	High
Healthcare Practitioners and Technical Occupations (29-0000)	Haq et al., 2020[67]	n=209	Registered Nurses	Cross-sectional survey	06/15 - 06/29	Pakistan	38.8% (32.1-45.7%)	Moderate
Healthcare Practitioners and Technical Occupations (29-0000)	Houlihan et al., 2020[139]	n=106	Registered Nurses	Cross-sectional survey	03/26 - 04/08	The United Kingdom	24%	High
Healthcare Practitioners and Technical Occupations (29-0000)	Houlihan et al., 2020[139]	n=22	Registered Nurses	Cross-sectional survey	03/26 - 04/08	The United Kingdom	23%	High
Healthcare Practitioners and Technical Occupations (29-0000)	Hunter et al., 2020[21]	n=317	Registered Nurses	Cross-sectional survey	04/29 - 05/08	United States of America	2.2%	High
Healthcare Practitioners and Technical	Iversen et al., 2020[8]	n=9963	Registered Nurses	Cross-sectional survey	04/15 - 04/22	Denmark	4.03%	Low

Occupations (29-0000)								
Healthcare Practitioners and Technical Occupations (29-0000)	Iversen et al., 2020[8]	n=1786	Registered Nurses	Cross-sectional survey	04/15 - 04/22	Denmark	4.65%	Low
Healthcare Practitioners and Technical Occupations (29-0000)	Jeremias et al., 2020[70]	n=1043	Registered Nurses	Cross-sectional survey	03/01 - 04/30	United States of America	9.5%	High
Healthcare Practitioners and Technical Occupations (29-0000)	Jones et al., 2020[29]	n=1962	Registered Nurses	Cross-sectional survey	01/15 - 06/15	The United Kingdom	10.5%	High
Healthcare Practitioners and Technical Occupations (29-0000)	Kassem et al., 2020[72]	n=28	Registered Nurses	Cross-sectional survey	06/01 - 06/14	Egypt	10.71%	High
Healthcare Practitioners and Technical Occupations (29-0000)	Kassem et al., 2020[72]	n=28	Registered Nurses	Cross-sectional survey	06/01 - 06/14	Egypt	7.14%	High
Healthcare Practitioners and Technical Occupations (29-0000)	Kassem et al., 2020[72]	n=28	Registered Nurses	Cross-sectional survey	06/01 - 06/14	Egypt	3.57%	High
Healthcare Practitioners and Technical Occupations (29-0000)	Kassem et al., 2020[72]	n=28	Registered Nurses	Cross-sectional survey	06/01 - 06/14	Egypt	0%	High

Healthcare Practitioners and Technical Occupations (29-0000)	Khan et al., 2020[127]	n=321	Registered Nurses	Cross-sectional survey	06/15 - 06/29	India	2.8% (1.5-5.3%)	Moderate
Healthcare Practitioners and Technical Occupations (29-0000)	Kohler et al., 2020[141]	n=398	Registered Nurses	Cross-sectional survey	03/19 - 04/03	Switzerland	0.75%	High
Healthcare Practitioners and Technical Occupations (29-0000)	Kumar et al., 2020[142]	n=308	Registered Nurses	Cross-sectional survey	06/01 - 06/30	India	6.8% (4.5-10.2%)	High
Healthcare Practitioners and Technical Occupations (29-0000)	Leidner et al., 2020[22]	n=110	Registered Nurses	Cross sectional study with prospective cohort follow up of a subset of the sample	04/08 - 05/22	United States of America	0%	High
Healthcare Practitioners and Technical Occupations (29-0000)	Leidner et al., 2020[22]	n=3504	Registered Nurses	Cross sectional study with prospective cohort follow up of a subset of the sample	04/08 - 05/22	United States of America	2.34%	High
Healthcare Practitioners and Technical Occupations (29-0000)	Lumley et al., 2020[9]	n=4528	Registered Nurses	Prospective cohort	04/23 - 11/30	The United Kingdom	13.21%	Moderate
Healthcare Practitioners and Technical Occupations (29-0000)	Mansour et al., 2020[160]	n=285	Registered Nurses	Cross-sectional survey	03/24 - 04/04	United States of America	32.63%	High

Healthcare Practitioners and Technical Occupations (29-0000)	Martin et al., 2020[161]	n=580	Registered Nurses	Cross-sectional survey	04/01 - 04/15	Spain	5.52%	High
Healthcare Practitioners and Technical Occupations (29-0000)	Martin et al., 2020[161]	n=74	Registered Nurses	Cross-sectional survey	04/01 - 04/15	Spain	9.46%	High
Healthcare Practitioners and Technical Occupations (29-0000)	Martin et al., 2020[161]	n=676	Registered Nurses	Cross-sectional survey	04/01 - 04/15	Spain	5.92%	High
Healthcare Practitioners and Technical Occupations (29-0000)	Martin et al., 2020[161]	n=337	Registered Nurses	Cross-sectional survey	04/01 - 04/15	Spain	5.93%	High
Healthcare Practitioners and Technical Occupations (29-0000)	Martin et al., 2020[161]	n=339	Registered Nurses	Cross-sectional survey	04/01 - 04/15	Spain	5.9%	High
Healthcare Practitioners and Technical Occupations (29-0000)	Meissner et al., 2020[162]	n=439	Registered Nurses	Cross-sectional survey	04/14 - 05/06	United States of America	1.37%	High
Healthcare Practitioners and Technical Occupations (29-0000)	Mohr et al., 2020[129]	n=410	Registered Nurses	Cross-sectional survey	05/13 - 07/08	United States of America	1.46%	Moderate
Healthcare Practitioners and	Moscola et al., 2020[89]	n=11468	Registered Nurses	Cross-sectional survey	04/20 - 06/23	United States of America	13.1%	High

Technical Occupations (29-0000)								
Healthcare Practitioners and Technical Occupations (29-0000)	Mostafa et al., 2020[163]	n=4040	Registered Nurses	Cross-sectional survey	04/22 - 05/14	Egypt	1.31%	High
Healthcare Practitioners and Technical Occupations (29-0000)	Nishida et al., 2020[90]	n=489	Registered Nurses	Cross-sectional survey	06/12 - 06/19	Japan	0.2% (0.04-1.1%)	Moderate
Healthcare Practitioners and Technical Occupations (29-0000)	Noor et al., 2020[130]	n=460	Registered Nurses	Cross-sectional survey	07/13 - 07/15	Pakistan	39.78%	Moderate
Healthcare Practitioners and Technical Occupations (29-0000)	Paradiso et al., 2020[164]	n=606	Registered Nurses	Cross sectional study with prospective cohort follow up of a subset of the sample	03/26 - 04/17	Italy	0.33%	High
Healthcare Practitioners and Technical Occupations (29-0000)	Plebani et al., 2020[146]	n=3230	Registered Nurses	Cross-sectional survey	02/22 - 05/29	Italy	4.7% (4-5.5%)	High
Healthcare Practitioners and Technical Occupations (29-0000)	Poustchi et al., 2020[28]	n=1245	Registered Nurses	Cross-sectional survey	04/17 - 06/02	Iran (Islamic Republic of)	15.9% (13.9-18%)	Moderate
Healthcare Practitioners and Technical	Rudberg et al., 2020[147]	n=636	Registered Nurses	Cross-sectional survey	04/14 - 05/08	Sweden	21.9%	Moderate

Occupations (29-0000)								
Healthcare Practitioners and Technical Occupations (29-0000)	Schmidt et al., 2020[148]	n=154	Registered Nurses	Cross-sectional survey	04/20 - 04/30	Germany	0%	High
Healthcare Practitioners and Technical Occupations (29-0000)	Self et al., 2020[154]	n=1445	Registered Nurses	Cross-sectional survey	04/03 - 06/19	United States of America	5.05%	Moderate
Healthcare Practitioners and Technical Occupations (29-0000)	Siddiqui et al., 2020[2]	n=59	Registered Nurses	Prospective cohort	04/15 - 08/15	India	10.2%	High
Healthcare Practitioners and Technical Occupations (29-0000)	Siddiqui et al., 2020[2]	n=70	Registered Nurses	Prospective cohort	04/15 - 08/15	India	10%	High
Healthcare Practitioners and Technical Occupations (29-0000)	Sotgiu et al., 2020[149]	n=64	Registered Nurses	Cross-sectional survey	04/02 - 04/16	Italy	7.8% (1.2-14.4%)	High
Healthcare Practitioners and Technical Occupations (29-0000)	Sydney et al., 2020[165]	n=81	Registered Nurses	Cross-sectional survey	04/28 - 05/04	United States of America	18.52%	High
Healthcare Practitioners and Technical Occupations (29-0000)	Urbieta et al., 2020[132]	n=83	Registered Nurses	Cross-sectional survey	04/14 - 04/16	Spain	4.8%	High

Healthcare Practitioners and Technical Occupations (29-0000)	Urbieta et al., 2020[132]	n=23	Registered Nurses	Cross-sectional survey	04/14 - 04/16	Spain	8.7%	High
Healthcare Practitioners and Technical Occupations (29-0000)	Venugopal et al., 2020[150]	n=142	Registered Nurses	Cross-sectional survey	03/01 - 05/01	United States of America	28%	Moderate
Healthcare Practitioners and Technical Occupations (29-0000)	Yogo et al., 2020[36]	n=1129	Registered Nurses	Cross-sectional survey	05/20 - 06/08	United States of America	2.48%	High
Healthcare Practitioners and Technical Occupations (29-0000)	Yogo et al., 2020[36]	n=12	Registered Nurses	Cross-sectional survey	05/20 - 06/08	United States of America	0%	High
Healthcare Practitioners and Technical Occupations (29-0000)	Zhou et al., 2020[166]	n=2406	Registered Nurses	Cross-sectional survey	03/16 - 03/25	China	1.37%	Moderate
Healthcare Practitioners and Technical Occupations (29-0000)	Godbout et al., 2020[138]	n=141	Nurse Practitioners	Cross-sectional survey	07/27 - 10/02	United States of America	1.42%	High
Healthcare Practitioners and Technical Occupations (29-0000)	Dimcheff et al., 2020[157]	n=214	Nurse Practitioners	Cross-sectional survey	06/08 - 07/08	United States of America	3.7%	Moderate
Healthcare Practitioners and	Akinbami et al., 2020[46]	n=719	Health Technologists and Technicians	Cross-sectional survey	05/18 - 06/13	United States of America	4.2% (2.8-5.9%)	Moderate

Technical Occupations (29-0000)								
Healthcare Practitioners and Technical Occupations (29-0000)	Blairon et al., 2020[52]	n=61	Health Technologists and Technicians	Cross-sectional survey	05/25 - 06/19	Belgium	6.6%	High
Healthcare Practitioners and Technical Occupations (29-0000)	Yogo et al., 2020[36]	n=65	Health Technologists and Technicians	Cross-sectional survey	05/20 - 06/08	United States of America	4.62%	High
Healthcare Practitioners and Technical Occupations (29-0000)	Silva et al., 2020[34]	n=224	Clinical Laboratory Technologists and Technicians	Cross-sectional survey	06/05 - 07/31	Brazil	7.59%	High
Healthcare Practitioners and Technical Occupations (29-0000)	Costa et al., 2020[128]	n=66	Medical and Clinical Laboratory Technologists	Cross-sectional survey	05/14 - 05/28	Brazil	3%	Moderate
Healthcare Practitioners and Technical Occupations (29-0000)	Akinbami et al., 2020[46]	n=293	Medical and Clinical Laboratory Technicians	Cross-sectional survey	05/18 - 06/13	United States of America	3.4% (1.7-6.2%)	Moderate
Healthcare Practitioners and Technical Occupations (29-0000)	Akinbami et al., 2020[46]	n=365	Medical and Clinical Laboratory Technicians	Cross-sectional survey	05/18 - 06/13	United States of America	5.5% (3.4-8.3%)	Moderate
Healthcare Practitioners and Technical	Alharbi et al., 2020[125]	n=80	Medical and Clinical Laboratory Technicians	Cross-sectional survey	04/18 - 06/17	Saudi Arabia	20%	High

Occupations (29-0000)								
Healthcare Practitioners and Technical Occupations (29-0000)	Baracco et al., 2020[24]	n=256	Medical and Clinical Laboratory Technicians	Cross-sectional survey	04/23 - 05/05	Italy	12.1%	High
Healthcare Practitioners and Technical Occupations (29-0000)	Brehm et al., 2020[7]	n=105	Medical and Clinical Laboratory Technicians	Cross sectional study with prospective cohort follow up of a subset of the sample	03/20 - 07/17	Germany	0%	Moderate
Healthcare Practitioners and Technical Occupations (29-0000)	Calcagno et al., 2020[124]	n=216	Medical and Clinical Laboratory Technicians	Cross-sectional survey	04/17 - 05/20	Italy	6.94%	Moderate
Healthcare Practitioners and Technical Occupations (29-0000)	Calcagno et al., 2020[124]	n=157	Medical and Clinical Laboratory Technicians	Cross-sectional survey	04/17 - 05/20	Italy	11.46%	Moderate
Healthcare Practitioners and Technical Occupations (29-0000)	Chau et al., 2020[126]	n=33	Medical and Clinical Laboratory Technicians	Cross-sectional survey	08/23 - 08/30	Viet Nam	0%	High
Healthcare Practitioners and Technical Occupations (29-0000)	Galan et al., 2020[20]	n=192	Medical and Clinical Laboratory Technicians	Cross-sectional survey	04/14 - 04/27	Spain	21.35%	High
Healthcare Practitioners and Technical	Goenka et al., 2020[25]	n=72	Medical and Clinical Laboratory Technicians	Cross-sectional survey	07/12 - 08/23	India	15.28%	Moderate

Occupations (29-0000)								
Healthcare Practitioners and Technical Occupations (29-0000)	Haq et al., 2020[67]	n=32	Medical and Clinical Laboratory Technicians	Cross-sectional survey	06/15 - 06/29	Pakistan	50% (31.8-68.1%)	Moderate
Healthcare Practitioners and Technical Occupations (29-0000)	Iversen et al., 2020[8]	n=1292	Medical and Clinical Laboratory Technicians	Cross-sectional survey	04/15 - 04/22	Denmark	1.93%	Low
Healthcare Practitioners and Technical Occupations (29-0000)	Khan et al., 2020[127]	n=397	Medical and Clinical Laboratory Technicians	Cross-sectional survey	06/15 - 06/29	India	2.5% (1.4-4.6%)	Moderate
Healthcare Practitioners and Technical Occupations (29-0000)	Lumley et al., 2020[9]	n=452	Medical and Clinical Laboratory Technicians	Prospective cohort	04/23 - 11/30	The United Kingdom	8.63%	Moderate
Healthcare Practitioners and Technical Occupations (29-0000)	Nishida et al., 2020[90]	n=140	Medical and Clinical Laboratory Technicians	Cross-sectional survey	06/12 - 06/19	Japan	0%	Moderate
Healthcare Practitioners and Technical Occupations (29-0000)	Rosser et al., 2020[33]	n=225	Medical and Clinical Laboratory Technicians	Cross-sectional survey	04/20 - 05/20	United States of America	0.44%	High
Healthcare Practitioners and Technical Occupations (29-0000)	Iversen et al., 2020[8]	n=342	Radiologic Technologists	Cross-sectional survey	04/15 - 04/22	Denmark	3.51%	Low

Healthcare Practitioners and Technical Occupations (29-0000)	Martin et al., 2020[23]	n=241	Radiologic Technologists	Cross-sectional survey	05/29 - 07/13	The United Kingdom	9.96%	Moderate
Healthcare Practitioners and Technical Occupations (29-0000)	Akinbami et al., 2020[46]	n=1158	Emergency Medical Technicians and Paramedics	Cross-sectional survey	05/18 - 06/13	United States of America	5.2% (4-6.6%)	Moderate
Healthcare Practitioners and Technical Occupations (29-0000)	Buntinx et al., 2020[167]	n=10	Emergency Medical Technicians and Paramedics	Cross-sectional survey	04/14 - 04/16	Belgium	10%	High
Healthcare Practitioners and Technical Occupations (29-0000)	Haq et al., 2020[67]	n=157	Emergency Medical Technicians and Paramedics	Cross-sectional survey	06/15 - 06/29	Pakistan	42% (34.2-50.1%)	Moderate
Healthcare Practitioners and Technical Occupations (29-0000)	Iversen et al., 2020[8]	n=323	Emergency Medical Technicians and Paramedics	Cross-sectional survey	04/15 - 04/22	Denmark	4.95%	Low
Healthcare Practitioners and Technical Occupations (29-0000)	Mesnil et al., 2020[143]	n=212	Emergency Medical Technicians and Paramedics	Cross-sectional survey	06/08 - 06/22	France	11%	High
Healthcare Practitioners and Technical Occupations (29-0000)	Reuben et al., 2020[168]	n=10	Emergency Medical Technicians and Paramedics	Cross-sectional survey	05/28 - 07/15	United States of America	0%	High

Healthcare Practitioners and Technical Occupations (29-0000)	Saberian et al., 2020[169]	n=243	Emergency Medical Technicians and Paramedics	Cross-sectional survey	03/20 - 05/20	Iran (Islamic Republic of)	41.56%	High
Healthcare Practitioners and Technical Occupations (29-0000)	Self et al., 2020[154]	n=56	Emergency Medical Technicians and Paramedics	Cross-sectional survey	04/03 - 06/19	United States of America	5.36%	Moderate
Healthcare Practitioners and Technical Occupations (29-0000)	Tarabichi et al., 2020[170]	n=111	Emergency Medical Technicians and Paramedics	Cross-sectional survey	04/20 - 05/19	United States of America	5.41%	High
Healthcare Practitioners and Technical Occupations (29-0000)	Baracco et al., 2020[24]	n=188	Health Technologists and Technicians, All Other	Cross-sectional survey	04/23 - 05/05	Italy	13.8%	High
Healthcare Practitioners and Technical Occupations (29-0000)	Chau et al., 2020[126]	n=22	Health Technologists and Technicians, All Other	Cross-sectional survey	08/23 - 08/30	Viet Nam	0%	High
Healthcare Practitioners and Technical Occupations (29-0000)	Goenka et al., 2020[25]	n=99	Health Technologists and Technicians, All Other	Cross-sectional survey	07/12 - 08/23	India	12.12%	Moderate
Healthcare Practitioners and Technical Occupations (29-0000)	Goenka et al., 2020[26]	n=16	Health Technologists and Technicians, All Other	Cross-sectional survey	08/01 - 08/31	India	68.75%	High
Healthcare Support	Jeremias et al., 2020[70]	n=155	Healthcare Support Occupations	Cross-sectional survey	03/01 - 04/30	United States of America	5.8%	High

Occupations (31-0000)								
Healthcare Support Occupations (31-0000)	Ward et al., 2020[113]	n=979	Nursing, Psychiatric, and Home Health Aides	Cross-sectional survey	09/15 - 09/28	The United Kingdom	11.09% (8.96-13.59%)	Moderate
Healthcare Support Occupations (31-0000)	Ward et al., 2020[113]	n=257	Nursing, Psychiatric, and Home Health Aides	Cross-sectional survey	09/15 - 09/28	The United Kingdom	8.95%	Moderate
Healthcare Support Occupations (31-0000)	Vijh et al., 2020[171]	n=169	Nursing, Psychiatric, and Home Health Aides	Cross-sectional survey	05/04 - 05/14	Canada	26.63%	High
Healthcare Support Occupations (31-0000)	Akinbami et al., 2020[46]	n=641	Nursing Assistants	Cross-sectional survey	05/18 - 06/13	United States of America	12.8% (10.3-15.6%)	Moderate
Healthcare Support Occupations (31-0000)	Bampoe et al., 2020[156]	n=108	Nursing Assistants	Cross-sectional survey	05/11 - 06/05	The United Kingdom	15.7% (9.5-24%)	High
Healthcare Support Occupations (31-0000)	Baracco et al., 2020[24]	n=257	Nursing Assistants	Cross-sectional survey	04/23 - 05/05	Italy	22.2%	High
Healthcare Support Occupations (31-0000)	Barallat et al., 2020[50]	n=832	Nursing Assistants	Cross-sectional survey	05/04 - 05/22	Spain	13.94%	High
Healthcare Support Occupations (31-0000)	Bhattacharya et al., 2020[172]	n=121	Nursing Assistants	Cross-sectional survey	06/01 - 06/15	United States of America	1.65%	High
Healthcare Support	Brousseau et al., 2020[134]	n=132	Nursing Assistants	Cross-sectional survey	07/06 - 09/24	Canada	16.7%	High

Occupations (31-0000)								
Healthcare Support Occupations (31-0000)	Brunner et al., 2020[54]	n=95	Nursing Assistants	Cross-sectional survey	05/04 - 05/29	United States of America	1.05%	High
Healthcare Support Occupations (31-0000)	Brzostek et al., 2020[151]	n=570	Nursing Assistants	Cross-sectional survey	04/17 - 05/07	United States of America	39.5%	Moderate
Healthcare Support Occupations (31-0000)	Brzostek et al., 2020[151]	n=263	Nursing Assistants	Cross-sectional survey	04/17 - 05/07	United States of America	45.6%	Moderate
Healthcare Support Occupations (31-0000)	Calcagno et al., 2020[124]	n=476	Nursing Assistants	Cross-sectional survey	04/17 - 05/20	Italy	9.24%	Moderate
Healthcare Support Occupations (31-0000)	Costa et al., 2020[128]	n=553	Nursing Assistants	Cross-sectional survey	05/14 - 05/28	Brazil	10.5%	Moderate
Healthcare Support Occupations (31-0000)	Galan et al., 2020[20]	n=472	Nursing Assistants	Cross-sectional survey	04/14 - 04/27	Spain	33.26%	High
Healthcare Support Occupations (31-0000)	Garcia et al., 2020[173]	n=2424	Nursing Assistants	Cross-sectional survey	05/01 - 05/30	Spain	22.4%	High
Healthcare Support Occupations (31-0000)	Garcia et al., 2020[174]	n=2424	Nursing Assistants	Cross-sectional survey	05/01 - 05/30	Spain	22.4%	High
Healthcare Support	Hanrath et al., 2020[32]	n=1434	Nursing Assistants	Cross-sectional survey	05/29 - 07/06	The United Kingdom	11.44%	High

Occupations (31-0000)								
Healthcare Support Occupations (31-0000)	Iversen et al., 2020[8]	n=501	Nursing Assistants	Cross-sectional survey	04/15 - 04/22	Denmark	1.2%	Low
Healthcare Support Occupations (31-0000)	Khan et al., 2020[127]	n=624	Nursing Assistants	Cross-sectional survey	06/15 - 06/29	India	2.4% (1.5-4%)	Moderate
Healthcare Support Occupations (31-0000)	Mughal et al., 2020[175]	n=121	Nursing Assistants	Cross-sectional survey	05/14 - 05/19	United States of America	0.83%	High
Healthcare Support Occupations (31-0000)	Rao et al., 2020[176]	n=1000	Nursing Assistants	Cross-sectional survey	05/23 - 06/06	India	1%	Unclear
Healthcare Support Occupations (31-0000)	Rudberg et al., 2020[147]	n=428	Nursing Assistants	Cross-sectional survey	04/14 - 05/08	Sweden	25.5%	Moderate
Healthcare Support Occupations (31-0000)	Siddiqui et al., 2020[2]	n=28	Nursing Assistants	Prospective cohort	04/15 - 08/15	India	10.7%	High
Healthcare Support Occupations (31-0000)	Yogo et al., 2020[36]	n=154	Nursing Assistants	Cross-sectional survey	05/20 - 06/08	United States of America	3.24%	High
Healthcare Support Occupations (31-0000)	Brousseau et al., 2020[134]	n=201	Orderlies	Cross-sectional survey	07/06 - 09/24	Canada	17.9%	High
Healthcare Support	Kassem et al., 2020[72]	n=9	Orderlies	Cross-sectional survey	06/01 - 06/14	Egypt	0%	High

Occupations (31-0000)								
Healthcare Support Occupations (31-0000)	Kassem et al., 2020[72]	n=9	Orderlies	Cross-sectional survey	06/01 - 06/14	Egypt	33.33%	High
Healthcare Support Occupations (31-0000)	Kassem et al., 2020[72]	n=9	Orderlies	Cross-sectional survey	06/01 - 06/14	Egypt	11.11%	High
Healthcare Support Occupations (31-0000)	Kassem et al., 2020[72]	n=9	Orderlies	Cross-sectional survey	06/01 - 06/14	Egypt	22.22%	High
Healthcare Support Occupations (31-0000)	Hanrath et al., 2020[32]	n=122	Orderlies	Cross-sectional survey	05/29 - 07/06	The United Kingdom	9.02%	High
Healthcare Support Occupations (31-0000)	Lumley et al., 2020[9]	n=377	Orderlies	Prospective cohort	04/23 - 11/30	The United Kingdom	15.38%	Moderate
Healthcare Support Occupations (31-0000)	Rosser et al., 2020[33]	n=3959	Medical Assistants	Cross-sectional survey	04/20 - 05/20	United States of America	1.39%	High
Healthcare Support Occupations (31-0000)	Yogo et al., 2020[36]	n=106	Phlebotomists	Cross-sectional survey	05/20 - 06/08	United States of America	0%	High
Healthcare Support Occupations (31-0000)	Cavlek et al., 2020[56]	n=300	Healthcare Support Workers, All Other	Cross-sectional survey	04/25 - 05/24	Croatia	0.67%	High
Healthcare Support	Erber et al., 2020[31]	n=383	Healthcare Support Workers, All Other	Cross-sectional survey	04/14 - 05/29	Germany	2.34%	High

Occupations (31-0000)								
Healthcare Support Occupations (31-0000)	Khan et al., 2020[127]	n=141	Healthcare Support Workers, All Other	Cross-sectional survey	06/15 - 06/29	India	0%	Moderate
Protective Service Occupations (33-0000)	Shukla et al., 2020[177]	n=1713	Protective Service Occupations	Cross-sectional survey	04/24 - 05/21	United States of America	1.46%	Moderate
Protective Service Occupations (33-0000)	Martinez et al., 2020[121]	n=18	First-Line Supervisors of Fire Fighting and Prevention Workers	Cross-sectional survey	04/16 - 04/17	United States of America	0%	High
Protective Service Occupations (33-0000)	Martinez et al., 2020[121]	n=47	First-Line Supervisors of Fire Fighting and Prevention Workers	Cross-sectional survey	04/16 - 04/17	United States of America	14.89%	High
Protective Service Occupations (33-0000)	Martinez et al., 2020[121]	n=13	First-Line Supervisors of Fire Fighting and Prevention Workers	Cross-sectional survey	04/16 - 04/17	United States of America	7.69%	High
Protective Service Occupations (33-0000)	Akinbami et al., 2020[46]	n=330	Firefighters	Cross-sectional survey	05/18 - 06/13	United States of America	6.7% (4.2-9.9%)	Moderate
Protective Service Occupations (33-0000)	Gray et al., 2020[178]	n=132	Firefighters	Cross-sectional survey	05/01 - 05/31	United States of America	14%	High
Protective Service Occupations (33-0000)	Reuben et al., 2020[168]	n=62	Firefighters	Cross-sectional survey	05/28 - 07/15	United States of America	4.84%	High
Protective Service Occupations (33-0000)	Sabourin et al., 2020[35]	n=42	Firefighters	Cross-sectional survey	07/15 - 08/15	United States of America	2.38%	High
Protective Service Occupations (33-0000)	Tarabichi et al., 2020[170]	n=185	Firefighters	Cross-sectional survey	04/20 - 05/19	United States of America	5.41%	High

Protective Service Occupations (33-0000)	Martinez et al., 2020[121]	n=7	Fire Inspectors and Investigators	Cross-sectional survey	04/16 - 04/17	United States of America	14.29%	High
Protective Service Occupations (33-0000)	Akinbami et al., 2020[46]	n=785	Police and Sheriff's Patrol Officers	Cross-sectional survey	05/18 - 06/13	United States of America	4% (2.7-5.6%)	Moderate
Protective Service Occupations (33-0000)	Chughtai et al., 2020[179]	n=154	Police and Sheriff's Patrol Officers	Cross-sectional survey	05/20 - 05/30	Pakistan	15.6%	High
Protective Service Occupations (33-0000)	Gudo et al., 2020[65]	n=564	Police and Sheriff's Patrol Officers	Cross-sectional survey	06/17 - 06/30	Mozambique	6% (4-8%)	High
Protective Service Occupations (33-0000)	Gujski et al., 2020[180]	n=4026	Police and Sheriff's Patrol Officers	Cross-sectional survey	06/22 - 07/08	Poland	4.2%	Moderate
Protective Service Occupations (33-0000)	Halatoko et al., 2020[41]	n=196	Police and Sheriff's Patrol Officers	Cross-sectional survey	04/23 - 05/08	Togo	0%	High
Protective Service Occupations (33-0000)	Langa et al., 2020[181]	n=471	Police and Sheriff's Patrol Officers	Cross-sectional survey	09/28 - 10/09	Mozambique	1.5%	High
Protective Service Occupations (33-0000)	Macicame et al., 2020[182]	n=456	Police and Sheriff's Patrol Officers	Cross-sectional survey	09/14 - 09/30	Mozambique	4.39%	High
Protective Service Occupations (33-0000)	Mahomed et al., 2020[81]	n=554	Police and Sheriff's Patrol Officers	Cross-sectional survey	08/31 - 10/12	Mozambique	2.9%	High
Protective Service Occupations (33-0000)	Reuben et al., 2020[168]	n=220	Police and Sheriff's Patrol Officers	Cross-sectional survey	05/28 - 07/15	United States of America	3.64%	High
Protective Service Occupations (33-0000)	Sabourin et al., 2020[35]	n=125	Police and Sheriff's Patrol Officers	Cross-sectional survey	07/15 - 08/15	United States of America	4%	High

Protective Service Occupations (33-0000)	Shukla et al., 2020[177]	n=1643	Police and Sheriff's Patrol Officers	Cross-sectional survey	04/24 - 05/21	United States of America	1.52%	Moderate
Protective Service Occupations (33-0000)	Siddiqui et al., 2020[2]	n=27	Police and Sheriff's Patrol Officers	Prospective cohort	04/15 - 08/15	India	7.4%	High
Protective Service Occupations (33-0000)	Viegas et al., 2020[110]	n=559	Police and Sheriff's Patrol Officers	Cross-sectional survey	08/03 - 08/21	Mozambique	3.94%	High
Protective Service Occupations (33-0000)	Denyer et al., 2020[60]	n=38216	Security Guards	Cross-sectional survey	05/12 - 05/18	Japan	0.23%	Unclear
Protective Service Occupations (33-0000)	Mahumane et al., 2020[82]	n=407	Security Guards	Cross-sectional survey	11/02 - 11/17	Mozambique	4.9%	High
Protective Service Occupations (33-0000)	Siddiqui et al., 2020[2]	n=9	Security Guards	Prospective cohort	04/15 - 08/15	India	0%	High
Protective Service Occupations (33-0000)	Silva et al., 2020[34]	n=32	Security Guards	Cross-sectional survey	06/05 - 07/31	Brazil	34%	High
Protective Service Occupations (33-0000)	Thani et al., 2020[183]	n=61	Security Guards	Cross-sectional survey	07/26 - 09/09	Qatar	60.1%	Moderate
Food Preparation and Serving Related Occupations (35-0000)	Thani et al., 2020[183]	n=93	Food Preparation and Serving Related Occupations	Cross-sectional survey	07/26 - 09/09	Qatar	29.2%	Moderate
Food Preparation and Serving Related Occupations (35-0000)	Siddiqui et al., 2020[2]	n=8	Cooks, All Other	Prospective cohort	04/15 - 08/15	India	37.5%	High
Food Preparation and Serving	Brunner et al., 2020[54]	n=8	Food Preparation Workers	Cross-sectional survey	05/04 - 05/29	United States of America	0%	High

Related Occupations (35-0000)								
Healthcare Support Occupations (31-0000)	Rosser et al., 2020[33]	n=335	Healthcare Support Occupations	Cross-sectional survey	04/20 - 05/20	United States of America	3.58%	High
Food Preparation and Serving Related Occupations (35-0000)	Biggs et al., 2020[3]	n=24	Food Servers, Nonrestaurant	Cross-sectional survey	04/28 - 05/03	United States of America	4.17%	Moderate
Food Preparation and Serving Related Occupations (35-0000)	Leidner et al., 2020[22]	n=113	Food Servers, Nonrestaurant	Cross sectional study with prospective cohort follow up of a subset of the sample	04/08 - 05/22	United States of America	1.77%	High
Food Preparation and Serving Related Occupations (35-0000)	Hanrath et al., 2020[32]	n=340	Other Food Preparation and Serving Related Workers	Cross-sectional survey	05/29 - 07/06	The United Kingdom	8.53%	High
Building and Grounds Cleaning and Maintenance Occupations (37-0000)	Martin et al., 2020[23]	n=528	Building and Grounds Cleaning and Maintenance Occupations	Cross-sectional survey	05/29 - 07/13	The United Kingdom	8.14%	Moderate
Building and Grounds Cleaning and Maintenance Occupations (37-0000)	Brousseau et al., 2020[134]	n=102	Building Cleaning and Pest Control Workers	Cross-sectional survey	07/06 - 09/24	Canada	10.8%	High
Building and Grounds Cleaning and Maintenance	Chau et al., 2020[126]	n=42	Building Cleaning and Pest Control Workers	Cross-sectional survey	08/23 - 08/30	Viet Nam	0%	High

Occupations (37-0000)								
Building and Grounds Cleaning and Maintenance Occupations (37-0000)	Finkenzeller et al., 2020[158]	n=57	Building Cleaning and Pest Control Workers	Prospective cohort	06/29 - 07/29	Germany	19.3%	Moderate
Building and Grounds Cleaning and Maintenance Occupations (37-0000)	Chau et al., 2020[126]	n=6	Janitors and Cleaners, Except Maids and Housekeeping Cleaners	Cross-sectional survey	08/23 - 08/30	Viet Nam	0%	High
Building and Grounds Cleaning and Maintenance Occupations (37-0000)	Epstude et al., 2020[184]	n=45	Janitors and Cleaners, Except Maids and Housekeeping Cleaners	Cross-sectional survey	06/15 - 06/30	Germany	0%	High
Building and Grounds Cleaning and Maintenance Occupations (37-0000)	Thani et al., 2020[183]	n=105	Janitors and Cleaners, Except Maids and Housekeeping Cleaners	Cross-sectional survey	07/26 - 09/09	Qatar	54.5%	Moderate
Building and Grounds Cleaning and Maintenance Occupations (37-0000)	Brunner et al., 2020[54]	n=23	Maids and Housekeeping Cleaners	Cross-sectional survey	05/04 - 05/29	United States of America	0%	High
Building and Grounds Cleaning and Maintenance Occupations (37-0000)	Goenka et al., 2020[25]	n=226	Maids and Housekeeping Cleaners	Cross-sectional survey	07/12 - 08/23	India	26.11%	Moderate
Building and Grounds Cleaning and Maintenance Occupations (37-0000)	Goenka et al., 2020[26]	n=10	Maids and Housekeeping Cleaners	Cross-sectional survey	08/01 - 08/31	India	10%	High

Building and Grounds Cleaning and Maintenance Occupations (37-0000)	Hanrath et al., 2020[32]	n=515	Maids and Housekeeping Cleaners	Cross-sectional survey	05/29 - 07/06	The United Kingdom	13.2%	High
Building and Grounds Cleaning and Maintenance Occupations (37-0000)	Khan et al., 2020[127]	n=276	Maids and Housekeeping Cleaners	Cross-sectional survey	06/15 - 06/29	India	3.3% (1.7-6.2%)	Moderate
Building and Grounds Cleaning and Maintenance Occupations (37-0000)	Leidner et al., 2020[22]	n=137	Maids and Housekeeping Cleaners	Cross sectional study with prospective cohort follow up of a subset of the sample	04/08 - 05/22	United States of America	8.03%	High
Building and Grounds Cleaning and Maintenance Occupations (37-0000)	Moscola et al., 2020[89]	n=7314	Maids and Housekeeping Cleaners	Cross-sectional survey	04/20 - 06/23	United States of America	20.9%	High
Building and Grounds Cleaning and Maintenance Occupations (37-0000)	Shakiba et al., 2020[10]	n=159	Maids and Housekeeping Cleaners	Cross-sectional survey	04/11 - 04/19	Iran (Islamic Republic of)	25% (13.6-37.5%)	Moderate
Building and Grounds Cleaning and Maintenance Occupations (37-0000)	Shields et al., 2020[97]	n=29	Maids and Housekeeping Cleaners	Cross-sectional survey	04/24 - 04/25	The United Kingdom	34.5%	High
Building and Grounds Cleaning and Maintenance Occupations (37-0000)	Siddiqui et al., 2020[2]	n=46	Maids and Housekeeping Cleaners	Prospective cohort	04/15 - 08/15	India	21.7%	High

Personal Care and Service Occupations (39-0000)	Biggs et al., 2020[3]	n=10	Hairdressers, Hairstylists, and Cosmetologists	Cross-sectional survey	04/28 - 05/03	United States of America	10%	Moderate
Personal Care and Service Occupations (39-0000)	Biggs et al., 2020[3]	n=48	Childcare Workers	Cross-sectional survey	04/28 - 05/03	United States of America	0%	Moderate
Personal Care and Service Occupations (39-0000)	Chen et al., 2020[135]	n=11	Personal Care Aides	Cross-sectional survey	02/19 - 02/19	China	9.09%	High
Personal Care and Service Occupations (39-0000)	Galan et al., 2020[20]	n=337	Personal Care Aides	Cross-sectional survey	04/14 - 04/27	Spain	27.89%	High
Personal Care and Service Occupations (39-0000)	Galan et al., 2020[20]	n=168	Personal Care Aides	Cross-sectional survey	04/14 - 04/27	Spain	27.38%	High
Personal Care and Service Occupations (39-0000)	Godbout et al., 2020[138]	n=86	Personal Care Aides	Cross-sectional survey	07/27 - 10/02	United States of America	2.32%	High
Personal Care and Service Occupations (39-0000)	Hassan et al., 2020[185]	n=403	Personal Care Aides	Cross-sectional survey	05/11 - 06/17	Sweden	20.1%	High
Personal Care and Service Occupations (39-0000)	Kumar et al., 2020[142]	n=292	Personal Care Aides	Cross-sectional survey	06/01 - 06/30	India	18.5% (14.5-23.3%)	High
Personal Care and Service Occupations (39-0000)	Ladhani et al., 2020[186]	n=208	Personal Care Aides	Prospective cohort	04/10 - 04/13	The United Kingdom	75% (68.7-80.4%)	High

Personal Care and Service Occupations (39-0000)	Lindahl et al., 2020[187]	n=1005	Personal Care Aides	Cross-sectional survey	04/01 - 04/20	Sweden	22.9% (20.4-25.7%)	High
Personal Care and Service Occupations (39-0000)	Regan et al., 2020[188]	n=305	Personal Care Aides	Cross-sectional survey	04/15 - 05/06	United States of America	23.6%	Unclear
Personal Care and Service Occupations (39-0000)	Siddiqui et al., 2020[2]	n=5	Personal Care Aides	Prospective cohort	04/15 - 08/15	India	40%	High
Personal Care and Service Occupations (39-0000)	Venugopal et al., 2020[150]	n=72	Personal Care Aides	Cross-sectional survey	03/01 - 05/01	United States of America	28%	Moderate
Personal Care and Service Occupations (39-0000)	Viegas et al., 2020[110]	n=85	Personal Care Aides	Cross-sectional survey	08/03 - 08/21	Mozambique	1.18%	High
Sales and Related Occupations (41-0000)	Arnaldo et al., 2020[13]	n=928	Sales and Related Occupations	Cross-sectional survey	07/06 - 07/13	Mozambique	6.5%	High
Sales and Related Occupations (41-0000)	Arnaldo et al., 2020[48]	n=1123	Sales and Related Occupations	Cross-sectional survey	08/10 - 08/21	Mozambique	1.6%	High
Sales and Related Occupations (41-0000)	Langa et al., 2020[181]	n=871	Sales and Related Occupations	Cross-sectional survey	09/28 - 10/09	Mozambique	0.2%	High
Sales and Related Occupations (41-0000)	Mabunda et al., 2020[15]	n=1585	Sales and Related Occupations	Cross-sectional survey	09/21 - 10/02	Mozambique	8.3%	High
Sales and Related Occupations (41-0000)	Macicame et al., 2020[182]	n=1288	Sales and Related Occupations	Cross-sectional survey	09/14 - 09/30	Mozambique	4.97%	High

Sales and Related Occupations (41-0000)	Mahomed et al., 2020[81]	n=1556	Sales and Related Occupations	Cross-sectional survey	08/31 - 10/12	Mozambique	0.8%	High
Sales and Related Occupations (41-0000)	Mahumane et al., 2020[82]	n=643	Sales and Related Occupations	Cross-sectional survey	11/02 - 11/17	Mozambique	1.9%	High
Sales and Related Occupations (41-0000)	Arnaldo et al., 2020[14]	n=472	Sales and Related Occupations	Cross-sectional survey	11/16 - 11/21	Mozambique	6.8%	High
Sales and Related Occupations (41-0000)	Arnaldo et al., 2020[14]	n=460	Sales and Related Occupations	Cross-sectional survey	11/02 - 11/12	Mozambique	5.9%	High
Sales and Related Occupations (41-0000)	Mahomed et al., 2020[16]	n=517	Sales and Related Occupations	Cross-sectional survey	11/26 - 12/03	Mozambique	8.9%	High
Sales and Related Occupations (41-0000)	Mahomed et al., 2020[16]	n=1001	Sales and Related Occupations	Cross-sectional survey	11/07 - 11/21	Mozambique	4.5%	High
Sales and Related Occupations (41-0000)	Biggs et al., 2020[3]	n=19	Retail Sales Workers	Cross-sectional survey	04/28 - 05/03	United States of America	0%	Moderate
Sales and Related Occupations (41-0000)	Poustchi et al., 2020[28]	n=753	Cashiers	Cross-sectional survey	04/17 - 06/02	Iran (Islamic Republic of)	16.1% (12.9-19.2%)	Moderate
Sales and Related Occupations (41-0000)	Alali et al., 2020[189]	n=525	Cashiers	Cross-sectional survey	05/23 - 06/26	Kuwait	38.1% (34-42.3%)	High
Sales and Related Occupations (41-0000)	Denyer et al., 2020[60]	n=19075	Retail Salespersons	Cross-sectional survey	05/12 - 05/18	Japan	0.04%	Unclear
Sales and Related Occupations (41-0000)	Kern et al., 2020[73]	n=300	Retail Salespersons	Cross-sectional survey	04/09 - 04/16	Germany	0.33% (0.01-1.84%)	High

Sales and Related Occupations (41-0000)	Khan et al., 2020[45]	n=132	Retail Salespersons	Cross-sectional survey	07/01 - 07/15	India	5.3% (2.5-10.7%)	Moderate
Sales and Related Occupations (41-0000)	Thani et al., 2020[183]	n=171	Retail Salespersons	Cross-sectional survey	07/26 - 09/09	Qatar	40.3%	Moderate
Sales and Related Occupations (41-0000)	Siddiqui et al., 2020[2]	n=4	Sales Representatives, Wholesale and Manufacturing, Except Technical and Scientific Products	Prospective cohort	04/15 - 08/15	India	25%	High
Sales and Related Occupations (41-0000)	Biggs et al., 2020[3]	n=34	Real Estate Sales Agents	Cross-sectional survey	04/28 - 05/03	United States of America	0%	Moderate
Sales and Related Occupations (41-0000)	Gudo et al., 2020[65]	n=1493	Door-to-Door Sales Workers, News and Street Vendors, and Related Workers	Cross-sectional survey	06/17 - 06/30	Mozambique	10% (8-11%)	High
Sales and Related Occupations (41-0000)	Viegas et al., 2020[110]	n=1246	Door-to-Door Sales Workers, News and Street Vendors, and Related Workers	Cross-sectional survey	08/03 - 08/21	Mozambique	5.22%	High
Sales and Related Occupations (41-0000)	Shakiba et al., 2020[10]	n=46	Sales and Related Workers, All Other	Cross-sectional survey	04/11 - 04/19	Iran (Islamic Republic of)	8.7% (0.8-20%)	Moderate
Office and Administrative Support Occupations (43-0000)	Calcagno et al., 2020[124]	n=539	Office and Administrative Support Occupations	Cross-sectional survey	04/17 - 05/20	Italy	3.34%	Moderate
Office and Administrative Support Occupations (43-0000)	Costa et al., 2020[128]	n=120	Office and Administrative Support Occupations	Cross-sectional survey	05/14 - 05/28	Brazil	14.2%	Moderate

Office and Administrative Support Occupations (43-0000)	Rosser et al., 2020[33]	n=972	Office and Administrative Support Occupations	Cross-sectional survey	04/20 - 05/20	United States of America	1.34%	High
Office and Administrative Support Occupations (43-0000)	Tsitsilonis et al., 2020[12]	n=504	Office and Administrative Support Occupations	Cross-sectional survey	06/15 - 07/15	Greece	0.48% (0-2.37%)	Moderate
Office and Administrative Support Occupations (43-0000)	Khan et al., 2020[45]	n=37	Hotel, Motel, and Resort Desk Clerks	Cross-sectional survey	07/01 - 07/15	India	10.8% (4.1-25.5%)	Moderate
Office and Administrative Support Occupations (43-0000)	Brunner et al., 2020[54]	n=26	Receptionists and Information Clerks	Cross-sectional survey	05/04 - 05/29	United States of America	0%	High
Office and Administrative Support Occupations (43-0000)	Favara et al., 2020[136]	n=10	Receptionists and Information Clerks	Prospective cohort	06/01 - 06/07	The United Kingdom	0%	High
Office and Administrative Support Occupations (43-0000)	Moscola et al., 2020[89]	n=9645	Receptionists and Information Clerks	Cross-sectional survey	04/20 - 06/23	United States of America	12.6%	High
Office and Administrative Support Occupations (43-0000)	Biggs et al., 2020[3]	n=11	Shipping, Receiving, and Traffic Clerks	Cross-sectional survey	04/28 - 05/03	United States of America	18.18%	Moderate
Office and Administrative	Silva et al., 2020[34]	n=82	Stock Clerks and Order Fillers	Cross-sectional survey	06/05 - 07/31	Brazil	4.88%	High

Support Occupations (43-0000)								
Office and Administrative Support Occupations (43-0000)	Khan et al., 2020[45]	n=186	Secretaries and Administrative Assistants	Cross-sectional survey	07/01 - 07/15	India	3.8% (1.8-7.7%)	Moderate
Office and Administrative Support Occupations (43-0000)	Alemu et al., 2020[6]	n=48	Executive Secretaries and Executive Administrative Assistants	Cross-sectional survey	04/23 - 04/28	Ethiopia	2.1%	Moderate
Office and Administrative Support Occupations (43-0000)	Barallat et al., 2020[50]	n=1181	Executive Secretaries and Executive Administrative Assistants	Cross-sectional survey	05/04 - 05/22	Spain	6.52%	High
Office and Administrative Support Occupations (43-0000)	Lumley et al., 2020[9]	n=1557	Executive Secretaries and Executive Administrative Assistants	Prospective cohort	04/23 - 11/30	The United Kingdom	6.74%	Moderate
Office and Administrative Support Occupations (43-0000)	Reuben et al., 2020[168]	n=18	Executive Secretaries and Executive Administrative Assistants	Cross-sectional survey	05/28 - 07/15	United States of America	0%	High
Office and Administrative Support Occupations (43-0000)	Akinbami et al., 2020[46]	n=964	Medical Secretaries	Cross-sectional survey	05/18 - 06/13	United States of America	8% (6.4-9.9%)	Moderate
Office and Administrative Support	Alharbi et al., 2020[125]	n=8	Medical Secretaries	Cross-sectional survey	04/18 - 06/17	Saudi Arabia	25%	High

Occupations (43-0000)								
Office and Administrative Support Occupations (43-0000)	Dimcheff et al., 2020[157]	n=357	Medical Secretaries	Cross-sectional survey	06/08 - 07/08	United States of America	4.2%	Moderate
Office and Administrative Support Occupations (43-0000)	Erber et al., 2020[31]	n=557	Medical Secretaries	Cross-sectional survey	04/14 - 05/29	Germany	3.78%	High
Office and Administrative Support Occupations (43-0000)	Finkenzeller et al., 2020[158]	n=240	Medical Secretaries	Prospective cohort	06/29 - 07/29	Germany	7.1%	Moderate
Office and Administrative Support Occupations (43-0000)	Goenka et al., 2020[25]	n=75	Medical Secretaries	Cross-sectional survey	07/12 - 08/23	India	8%	Moderate
Office and Administrative Support Occupations (43-0000)	Goenka et al., 2020[25]	n=75	Medical Secretaries	Cross-sectional survey	07/12 - 08/23	India	8%	Moderate
Office and Administrative Support Occupations (43-0000)	Iversen et al., 2020[8]	n=2631	Medical Secretaries	Cross-sectional survey	04/15 - 04/22	Denmark	2.7%	Low
Office and Administrative Support Occupations (43-0000)	Leidner et al., 2020[22]	n=793	Medical Secretaries	Cross sectional study with prospective cohort follow up of a	04/08 - 05/22	United States of America	3.15%	High

				subset of the sample				
Office and Administrative Support Occupations (43-0000)	Mesnil et al., 2020[143]	n=184	Medical Secretaries	Cross-sectional survey	06/08 - 06/22	France	14.13%	High
Office and Administrative Support Occupations (43-0000)	Nishida et al., 2020[90]	n=98	Medical Secretaries	Cross-sectional survey	06/12 - 06/19	Japan	1% (0.18-5.6%)	Moderate
Office and Administrative Support Occupations (43-0000)	Noor et al., 2020[130]	n=91	Medical Secretaries	Cross-sectional survey	07/13 - 07/15	Pakistan	43.96%	Moderate
Office and Administrative Support Occupations (43-0000)	Thani et al., 2020[183]	n=82	Medical Secretaries	Cross-sectional survey	07/26 - 09/09	Qatar	31.6%	Moderate
Office and Administrative Support Occupations (43-0000)	Zhou et al., 2020[166]	n=505	Medical Secretaries	Cross-sectional survey	03/16 - 03/25	China	1.39%	Moderate
Office and Administrative Support Occupations (43-0000)	Chau et al., 2020[126]	n=20	Data Entry Keyers	Cross-sectional survey	08/23 - 08/30	Viet Nam	0%	High
Office and Administrative Support Occupations (43-0000)	Jones et al., 2020[29]	n=1233	Office Clerks, General	Cross-sectional survey	01/15 - 06/15	The United Kingdom	6.1%	High

Office and Administrative Support Occupations (43-0000)	Rosser et al., 2020[33]	n=218	Office Clerks, General	Cross-sectional survey	04/20 - 05/20	United States of America	0%	High
Office and Administrative Support Occupations (43-0000)	Satpati et al., 2020[27]	n=47	Office Clerks, General	Cross-sectional survey	07/26 - 08/08	India	4.26%	Moderate
Office and Administrative Support Occupations (43-0000)	Baracco et al., 2020[24]	n=194	Office and Administrative Support Workers, All Other	Cross-sectional survey	04/23 - 05/05	Italy	14.4%	High
Office and Administrative Support Occupations (43-0000)	Brzostek et al., 2020[151]	n=286	Office and Administrative Support Workers, All Other	Cross-sectional survey	04/17 - 05/07	United States of America	45.5%	Moderate
Office and Administrative Support Occupations (43-0000)	Kassem et al., 2020[72]	n=7	Office and Administrative Support Workers, All Other	Cross-sectional survey	06/01 - 06/14	Egypt	14.28%	High
Office and Administrative Support Occupations (43-0000)	Kassem et al., 2020[72]	n=7	Office and Administrative Support Workers, All Other	Cross-sectional survey	06/01 - 06/14	Egypt	0%	High
Office and Administrative Support Occupations (43-0000)	Kassem et al., 2020[72]	n=7	Office and Administrative Support Workers, All Other	Cross-sectional survey	06/01 - 06/14	Egypt	0%	High

Office and Administrative Support Occupations (43-0000)	Kassem et al., 2020[72]	n=7	Office and Administrative Support Workers, All Other	Cross-sectional survey	06/01 - 06/14	Egypt	14.28%	High
Farming, Fishing, and Forestry Occupations (45-0000)	Satpati et al., 2020[27]	n=53	Agricultural Workers	Cross-sectional survey	07/26 - 08/08	India	0%	Moderate
Farming, Fishing, and Forestry Occupations (45-0000)	Addetia et al., 2020[190]	n=120	Fishers and Related Fishing Workers	Retrospective cohort	05/01 - 05/31	United States of America	5%	High
Farming, Fishing, and Forestry Occupations (45-0000)	Arnaldo et al., 2020[13]	n=80	Fishers and Related Fishing Workers	Cross-sectional survey	07/06 - 07/13	Mozambique	5%	High
Construction and Extraction Occupations (47-0000)	Biggs et al., 2020[3]	n=42	Construction Trades Workers	Cross-sectional survey	04/28 - 05/03	United States of America	0%	Moderate
Installation, Maintenance, and Repair Occupations (49-0000)	Blairon et al., 2020[52]	n=134	Other Installation, Maintenance, and Repair Occupations	Cross-sectional survey	05/25 - 06/19	Belgium	16.4%	High
Production Occupations (51-0000)	Picon et al., 2020[191]	n=40	Butchers and Other Meat, Poultry, and Fish Processing Workers	Cross-sectional survey	06/13 - 06/17	Brazil	15%	Moderate
Production Occupations (51-0000)	Picon et al., 2020[191]	n=1087	Miscellaneous Food Processing Workers	Cross-sectional survey	06/13 - 06/17	Brazil	1.47%	Moderate
Production Occupations (51-0000)	Bontadi et al., 2020[192]	n=1267	Production Workers, All Other	Cross-sectional survey	04/11 - 04/29	Italy	1.58%	High

Production Occupations (51-0000)	Xu et al., 2020[193]	n=442	Production Workers, All Other	Cross-sectional survey	03/09 - 04/10	China	1.4% (0.6-2.9%)	High
Transportation and Material Moving Occupations (53-0000)	Arnaldo et al., 2020[13]	n=248	Transportation and Material Moving Occupations	Cross-sectional survey	07/06 - 07/13	Mozambique	4.8%	High
Transportation and Material Moving Occupations (53-0000)	Arnaldo et al., 2020[48]	n=367	Transportation and Material Moving Occupations	Cross-sectional survey	08/10 - 08/21	Mozambique	7.4%	High
Transportation and Material Moving Occupations (53-0000)	Arnaldo et al., 2020[14]	n=112	Transportation and Material Moving Occupations	Cross-sectional survey	11/16 - 11/21	Mozambique	16.1%	High
Transportation and Material Moving Occupations (53-0000)	Biggs et al., 2020[3]	n=14	Transportation and Material Moving Occupations	Cross-sectional survey	04/28 - 05/03	United States of America	0%	Moderate
Transportation and Material Moving Occupations (53-0000)	Gudo et al., 2020[65]	n=554	Transportation and Material Moving Occupations	Cross-sectional survey	06/17 - 06/30	Mozambique	3% (1-4%)	High
Transportation and Material Moving Occupations (53-0000)	Langa et al., 2020[181]	n=230	Transportation and Material Moving Occupations	Cross-sectional survey	09/28 - 10/09	Mozambique	0.4%	High
Transportation and Material Moving Occupations (53-0000)	Mabunda et al., 2020[15]	n=473	Transportation and Material Moving Occupations	Cross-sectional survey	09/21 - 10/02	Mozambique	8.7%	High
Transportation and Material Moving Occupations (53-0000)	Macicame et al., 2020[182]	n=282	Transportation and Material Moving Occupations	Cross-sectional survey	09/14 - 09/30	Mozambique	3.19%	High

Transportation and Material Moving Occupations (53-0000)	Mahomed et al., 2020[81]	n=334	Transportation and Material Moving Occupations	Cross-sectional survey	08/31 - 10/12	Mozambique	1.5%	High
Transportation and Material Moving Occupations (53-0000)	Mahumane et al., 2020[82]	n=287	Transportation and Material Moving Occupations	Cross-sectional survey	11/02 - 11/17	Mozambique	1%	High
Transportation and Material Moving Occupations (53-0000)	Thani et al., 2020[183]	n=435	Transportation and Material Moving Occupations	Cross-sectional survey	07/26 - 09/09	Qatar	53.4%	Moderate
Transportation and Material Moving Occupations (53-0000)	Halatoko et al., 2020[41]	n=212	Air Transportation Workers	Cross-sectional survey	04/23 - 05/08	Togo	0.9%	High
Transportation and Material Moving Occupations (53-0000)	Viegas et al., 2020[110]	n=623	Air Transportation Workers	Cross-sectional survey	08/03 - 08/21	Mozambique	2.25%	High
Transportation and Material Moving Occupations (53-0000)	Viegas et al., 2020[110]	n=362	Air Transportation Workers	Cross-sectional survey	08/03 - 08/21	Mozambique	3.31%	High
Transportation and Material Moving Occupations (53-0000)	Khan et al., 2020[127]	n=57	Ambulance Drivers and Attendants, Except Emergency Medical Technicians	Cross-sectional survey	06/15 - 06/29	India	3.5% (0.9-13.3%)	Moderate
Transportation and Material Moving Occupations (53-0000)	Martinez et al., 2020[121]	n=30	Heavy and Tractor-Trailer Truck Drivers	Cross-sectional survey	04/16 - 04/17	United States of America	16.67%	High
Transportation and Material Moving Occupations (53-0000)	Siddiqui et al., 2020[2]	n=9	Heavy and Tractor-Trailer Truck Drivers	Prospective cohort	04/15 - 08/15	India	11.1%	High

Transportation and Material Moving Occupations (53-0000)	Halatoko et al., 2020[41]	n=122	Taxi Drivers and Chauffeurs	Cross-sectional survey	04/23 - 05/08	Togo	0.8%	High
Transportation and Material Moving Occupations (53-0000)	Poustchi et al., 2020[28]	n=718	Taxi Drivers and Chauffeurs	Cross-sectional survey	04/17 - 06/02	Iran (Islamic Republic of)	14.1% (11.4-16.9%)	Moderate
Transportation and Material Moving Occupations (53-0000)	Alemu et al., 2020[6]	n=8	Parking Lot Attendants	Cross-sectional survey	04/23 - 04/28	Ethiopia	12.5%	Moderate
Transportation and Material Moving Occupations (53-0000)	Alemu et al., 2020[6]	n=110	Laborers and Freight, Stock, and Material Movers, Hand	Cross-sectional survey	04/23 - 04/28	Ethiopia	10%	Moderate
Transportation and Material Moving Occupations (53-0000)	Khan et al., 2020[45]	n=97	Laborers and Freight, Stock, and Material Movers, Hand	Cross-sectional survey	07/01 - 07/15	India	2.1% (0.5-7.9%)	Moderate
Transportation and Material Moving Occupations (53-0000)	Satpati et al., 2020[27]	n=63	Laborers and Freight, Stock, and Material Movers, Hand	Cross-sectional survey	07/26 - 08/08	India	12.7%	Moderate
Not employed (mixed)*	Carrat et al., 2020[4]	n=6295	Unemployed	Prospective cohort	05/04 - 06/23	France	4.9% (4.1-5.6%)	Moderate
Not employed (mixed)*	Carrat et al., 2020[4]	n=1457	Unemployed	Prospective cohort	05/04 - 06/23	France	8.3% (6.4-10%)	Moderate
Not employed (mixed)*	Carrat et al., 2020[4]	n=306	Unemployed	Prospective cohort	05/04 - 06/23	France	7.2% (2.3-11.1%)	Moderate
Not employed (mixed)*	Carrat et al., 2020[4]	n=125	Unemployed	Prospective cohort	05/04 - 06/23	France	3.8% (0.5-6.3%)	Moderate
Not employed (mixed)*	Carrat et al., 2020[4]	n=402	Unemployed	Prospective cohort	05/04 - 06/23	France	7.8% (4.7-10.4%)	Moderate

Not employed (mixed)*	Chamie et al., 2020[194]	n=230	Unemployed	Cross-sectional survey	04/25 - 04/28	United States of America	4.3%	Moderate
Not employed (mixed)*	McLaughlin et al., 2020[195]	n=241	Unemployed	Cross-sectional survey	05/04 - 05/19	United States of America	19.3% (14.6-24.5%)	Moderate
Not employed (mixed)*	Merkely et al., 2020[1]	n=1095	Unemployed	Cross-sectional survey	05/01 - 05/16	Hungary	0.43% (0.16-0.84%)	Moderate
Not employed (mixed)*	Munoz et al., 2020[196]	n=905	Unemployed	Cross-sectional survey	07/15 - 07/16	Argentina	20%	Moderate
Not employed (mixed)*	Richard et al., 2020[5]	n=549	Unemployed	Cross-sectional survey	04/06 - 06/30	Switzerland	6%	Low
Not employed (mixed)*	Satpati et al., 2020[27]	n=47	Unemployed	Cross-sectional survey	07/26 - 08/08	India	2.13%	Moderate
Not employed (mixed)*	Ward et al., 2020[113]	n=59369	Unemployed	Cross-sectional survey	09/15 - 09/28	The United Kingdom	3.35%	Moderate

1. Merkely B, Szabó AJ, Kosztin A, et al. Novel coronavirus epidemic in the Hungarian population, a cross-sectional nationwide survey to support the exit policy in Hungary. *GeroScience*. 2020;42(4):1063-1074. doi:10.1007/s11357-020-00226-9

2. Siddiqui S, Naushin S, Pradhan S, et al. SARS-CoV-2 antibody seroprevalence and stability in a tertiary care hospital-setting. *medRxiv*. Published online September 2020. doi:10.1101/2020.09.02.20186486

3. Biggs HM, Harris JB, Breakwell L, et al. Estimated Community Seroprevalence of SARS-CoV-2 Antibodies Two Georgia Counties, April 28May 3, 2020. *MMWR Morbidity and Mortality Weekly Report*. 2020;69(29):965-970. doi:10.15585/mmwr.mm6929e2

4. Carrat F, Lamballerie X de, Rahib D, et al. Seroprevalence of SARS-CoV-2 among adults in three regions of France following the lockdown and associated risk factors: A multicohort study. *medRxiv*. Published online September 2020:2020.09.16.20195693. doi:10.1101/2020.09.16.20195693

5. Richard A, Wisniak A, Perez-Saez J, et al. Seroprevalence of anti-SARS-CoV-2 IgG antibodies, risk factors for infection and associated symptoms in Geneva, Switzerland: A population-based study. *medRxiv*. Published online December 2020. doi:10.1101/2020.12.16.20248180

6. Alemu BN, Addissie A, Mamo G, et al. *Sero-Prevalence of Anti-SARS-CoV-2 Antibodies in Addis Ababa, Ethiopia*. Microbiology; 2020. doi:10.1101/2020.10.13.337287

7. Brehm T, Schwinge D, Lampalzer S, et al. Seroprevalence of SARS-CoV-2 antibodies among hospital workers in a German tertiary care center: A sequential follow-up study. *International Journal of Hygiene and Environmental Health*. 2021;232:113671. doi:10.1016/j.ijheh.2020.113671

8. Iversen K, Bundgaard H, Hasselbalch RB, et al. Risk of COVID-19 in health-care workers in Denmark: An observational cohort study. *The Lancet Infectious diseases*. Published online August 2020. doi:10.1016/S1473-3099(20)30589-2

9. Lumley SF, O'Donnell D, Stoesser NE, et al. Antibody Status and Incidence of SARS-CoV-2 Infection in Health Care Workers. *New England Journal of Medicine*. Published online December 2020:NEJMoa2034545. doi:10.1056/NEJMoa2034545

10. Shakiba M, Nazemipour M, Salari A, et al. Seroprevalence of SARS-CoV-2 in Guilan Province, Iran, April 2020. *Emerging Infectious Disease journal*. 2021;27(2). doi:10.3201/eid2702.201960

11. Tilley K, Ayvazyan V, Martinez L, et al. A Cross-Sectional Study Examining the Seroprevalence of Severe Acute Respiratory Syndrome Coronavirus 2 Antibodies in a University Student Population. *Journal of Adolescent Health*. 2020;67(6):763-768. doi:10.1016/j.jadohealth.2020.09.001

12. Tsitsilonis OE, Paraskevis D, Lianidou E, et al. Seroprevalence of Antibodies against SARS-CoV-2 among the Personnel and Students of the National and Kapodistrian University of Athens, Greece: A Preliminary Report. *Life*. 2020;10(9):214. doi:10.3390/life10090214

13. Paulo Arnaldo. *Inquérito Sero-Epidemiológico de SARS-CoV-2 Na Cidade de Pemba (InCOVID 2020)*. República de Moçambique Ministério da Saúde; 2020.

14. Paulo Arnaldo. *Inquérito Sero-Epidemiológico de SARS-CoV-2 Nas Cidades de Xai-Xai E Chókwè (InCOVID 2020)*. República de Moçambique Ministério da Saúde; 2020.

15. Nedio Mabunda. *Inquérito Sero-Epidemiológico de SARS-CoV-2 Na Cidade de Beira (InCOVID 2020)*. República de Moçambique Ministério da Saúde; 2020.

16. Mussagy Mahomed. *Inquérito Sero-Epidemiológico de SARS-CoV-2 Na Cidade de Maxixe E Vila de Massinga (InCOVID 2020)*. República de Moçambique Ministério da Saúde; 2020.

17. Payne DC, Smith-Jeffcoat SE, Nowak G, et al. SARS-CoV-2 Infections and Serologic Responses from a Sample of U.S. Navy Service Members - USS Theodore Roosevelt, April 2020. *MMWR Morbidity and mortality weekly report*. 2020;69(23):714-721. doi:10.15585/mmwr.mm6923e4

18. COVID-19 Serology Tests Still Show Low Antibody Rate of 0.07%. *KBS World Radio*.

19. Favara DM, McAdam K, Cooke A, et al. SARS-CoV-2 antigen and antibody prevalence among UK staff working with cancer patients during the COVID-19 pandemic. *medRxiv*. Published online September 2020:2020.09.18.20197590. doi:10.1101/2020.09.18.20197590

20. Galán MI, Velasco M, Casas ML, et al. Hospital-Wide SARS-CoV-2 seroprevalence in health care workers in a Spanish teaching hospital. *Enfermedades Infecciosas y Microbiología Clínica*. Published online December 2020:S0213005X20304183. doi:10.1016/j.eimc.2020.11.015

21. Hunter BR, Dbeibo L, Weaver CS, et al. Seroprevalence of severe acute respiratory coronavirus virus 2 (SARS-CoV-2) antibodies among healthcare workers with differing levels of coronavirus disease 2019 (COVID-19) patient exposure. *Infection Control & Hospital Epidemiology*. Published online August 2020:1-2. doi:10.1017/ice.2020.390

22. Leidner R, Frary A, Cramer J, et al. Longitudinal SARS-CoV-2 serosurveillance of over ten thousand health care workers in the Providence Oregon cohort. *medRxiv*. Published online August 2020:2020.08.16.20176107. doi:10.1101/2020.08.16.20176107

23. Martin CA, Patel P, Goss C, et al. Demographic and occupational determinants of anti-SARS-CoV-2 IgG seropositivity in hospital staff. *Journal of Public Health*. 2020;(fdaa199). doi:10.1093/pubmed/fdaa199

24. Baracco A, Perotti G, Filippin A, et al. *SARS-CoV-2 Antibody Prevalence in Health Care Workers of Lodi Hospital, the COVID-19 Italian Epicentre*. Social Science Research Network; 2020.

25. Goenka M, Afzalpurkar S, Goenka U, et al. Seroprevalence of COVID-19 Amongst Health Care Workers in a Tertiary Care Hospital of a Metropolitan City from India. *The Journal of the Association of Physicians of India*. 2020;68(11):14-19.

26. Goenka MK, Shah BB, Goenka U, et al. COVID-19 prevalence among health-care workers of Gastroenterology department: An audit from a tertiary-care hospital in India. *JGH Open*. 2021;5(1):56-63. doi:10.1002/jgh3.12447

27. Satpati P, Sarangi S, Gantait K, et al. *Sero-Surveillance (IgG) of SARS-CoV-2 Among Asymptomatic General Population of Paschim Medinipur, West Bengal, India*. Infectious Diseases (except HIV/AIDS); 2020. doi:10.1101/2020.09.12.20193219

28. Poustchi H, Darvishian M, Mohammadi Z, et al. SARS-CoV-2 antibody seroprevalence in the general population and high-risk occupational groups across 18 cities in Iran: A population-based cross-sectional study. *The Lancet Infectious Diseases*. 2020;0(0). doi:10.1016/S1473-3099(20)30858-6

29. Jones CR, Hamilton FW, Thompson A, Morris TT, Moran E. SARS-CoV-2 IgG seroprevalence in healthcare workers and other staff at North Bristol NHS Trust: A sociodemographic analysis. *Journal of Infection*. 2020;0(0). doi:10.1016/j.jinf.2020.11.036

30. Anna F, Goyard S, Lalanne AI, et al. High seroprevalence but short-lived immune response to SARS-CoV-2 infection in Paris. *medRxiv*. Published online November 2020:2020.10.25.20219030. doi:10.1101/2020.10.25.20219030

31. Erber J, Kappler V, Haller B, et al. Strategies for infection control and prevalence of anti-SARS-CoV-2 IgG in 4,554 employees of a university hospital in Munich, Germany. Published online October 2020. doi:10.1101/2020.10.04.20206136

32. Hanrath AT, Loeff IS van der, Lendrem DW, et al. SARS-CoV-2 testing of 11,884 healthcare workers at an acute NHS hospital trust in England: A retrospective analysis. *medRxiv*. Published online December 2020:2020.12.22.20242362. doi:10.1101/2020.12.22.20242362

33. Rosser JI, Röltgen K, Dymock M, et al. Severe acute respiratory coronavirus virus 2 (SARS-CoV-2) seroprevalence in healthcare personnel in northern California early in the coronavirus disease 2019 (COVID-19) pandemic. *Infection Control & Hospital Epidemiology*. Published online December 2020:1-7. doi:10.1017/ice.2020.1358

34. Silva VO, de Oliveira EL, Castejon MJ, et al. Prevalence of antibodies against sars-cov-2 in professionals of a public health laboratory at são paulo, sp, brazil. *medRxiv*. Published online October 2020. doi:10.1101/2020.10.19.20213421

35. Sabourin KR, Schultz J, Romero J, et al. Risk Factors of SARS-CoV-2 Antibodies in Arapahoe County First Responders - the COVID-19 Arapahoe SErosurveillance Study (CASES) Project. *Journal of Occupational and Environmental Medicine*. Published online December 2020. doi:10.1097/JOM.0000000000002099

36. Yogo N, Greenwood KL, Thompson L, et al. Point prevalence survey to evaluate the seropositivity for coronavirus disease 2019 (COVID-19) among high-risk healthcare workers. *Infection Control and Hospital Epidemiology*. Published online December 2020:1-6. doi:10.1017/ice.2020.1370

37. Figueiredo-Campos P, Blankenhaus B, Mota C, et al. Seroprevalence of anti-SARS-CoV-2 antibodies in COVID-19 patients and healthy volunteers up to 6 months post disease onset. *European Journal of Immunology*. 2020;50(12):2025-2040. doi:10.1002/eji.202048970

38. Gonçalves J, Sousa RL, Jacinto MJ, et al. Evaluating SARS-CoV-2 Seroconversion Following Relieve of Confinement Measures. *Frontiers in Medicine*. 2020;7. doi:10.3389/fmed.2020.603996

39. Fontanet A, Grant R, Tondeur L, et al. SARS-CoV-2 infection in primary schools in northern France: A retrospective cohort study in an area of high transmission. *medRxiv*. Published online June 2020:2020.06.25.20140178. doi:10.1101/2020.06.25.20140178

40. Torres JP, Piñera C, De La Maza V, et al. Severe Acute Respiratory Syndrome Coronavirus 2 Antibody Prevalence in Blood in a Large School Community Subject to a Coronavirus Disease 2019 Outbreak: A Cross-sectional Study. *Clinical Infectious Diseases*. Published online July 2020:ciaa955. doi:10.1093/cid/ciaa955

41. Halatoko WA, KONU YR, Gbeasor-Komlanvi FA, et al. Prevalence of SARS-CoV-2 among high-risk populations in Lom&Eacute (Togo) in 2020. *medRxiv*. Published online August 2020:2020.08.07.20163840. doi:10.1101/2020.08.07.20163840

42. Slusser S. MLB antibody study: 0.7% of those tested had been exposed to coronavirus. *San Francisco Chronicle*. Published online May 2020.

43. Vince A, Zadro R, Šostar Z, et al. SARS-CoV-2 Seroprevalence in a Cohort of Asymptomatic, RT-PCR Negative Croatian First League Football Players. *medRxiv*. Published online November 2020:2020.10.30.20223230. doi:10.1101/2020.10.30.20223230

44. Mack D, Gärtner BC, Rössler A, et al. Prevalence of SARS-CoV-2 IgG antibodies in a large prospective cohort study of elite football players in Germany (MayJune 2020): Implications for a testing protocol in asymptomatic individuals and estimation of the rate of undetected cases. *Clinical Microbiology and Infection*. 2020;27(3):473.e1-473.e4. doi:10.1016/j.cmi.2020.11.033

45. Khan SMS, Qurieshi MA, Haq I, et al. Seroprevalence of SARS-CoV-2 specific IgG antibodies in District Srinagar, northern India A cross-sectional study. *PLOS ONE*. 2020;15(11):e0239303. doi:10.1371/journal.pone.0239303

46. Akinbami LJ, Vuong N, Petersen LR, et al. SARS-CoV-2 Seroprevalence among Healthcare, First Response, and Public Safety Personnel, Detroit Metropolitan Area, Michigan, USA, MayJune 2020 - Volume 26, Number 12December 2020 - Emerging Infectious Diseases journal - CDC. Published online December 2020. doi:10.3201/eid2612.203764

47. Amendola A, Tanzi E, Folgori L, et al. Low seroprevalence of SARS-CoV-2 infection among healthcare workers of the largest children hospital in Milan during the pandemic wave. *Infection Control & Hospital Epidemiology*. Published online August 2020:1-2. doi:10.1017/ice.2020.401

48. Paulo Arnaldo. *Inquérito Sero-Epidemiológico de SARS-CoV-2 Na Cidade de Quelimane (InCOVID 2020)*. República de Moçambique Ministério da Saúde; 2020.

49. Bal A, Brengel-Pesce K, Gaymard A, et al. Clinical and microbiological assessments of COVID-19 in healthcare workers: A prospective longitudinal study. *medRxiv*. Published online November 2020:2020.11.04.20225862. doi:10.1101/2020.11.04.20225862

50. Fernández-Rivas G, Quirant-Sánchez B, González V, et al. Seroprevalence of SARS-CoV-2 IgG Specific Antibodies among Healthcare Workers in the Northern Metropolitan Area of Barcelona, Spain, after the first pandemic wave. *medRxiv*. Published online June 2020:2020.06.24.20135673. doi:10.1101/2020.06.24.20135673

51. Bardai G, Ouellet J, Engelhardt T, Bertolizio G, Wu Z, Rauch F. Prevalence of SARS-CoV-2 infections in a pediatric orthopedic hospital. von Ungern-Sternberg B, ed. *Pediatric Anesthesia*. 2021;31(2):247-248. doi:10.1111/pan.14047

52. Blairon L, Mokrane S, Wilmet A, et al. Large-scale, molecular and serological SARS-CoV-2 screening of healthcare workers in a 4-site public hospital in Belgium after COVID-19 outbreak. *Journal of Infection*. Published online July 2020:S0163445320305144. doi:10.1016/j.jinf.2020.07.033

53. Moreno Borraz LA, Giménez López M, Carrera Lasfuentes P, et al. Prevalencia de infección por coronavirus SARS-CoV-2 en pacientes y profesionales de un hospital de media y larga estancia en España. *Revista Española de Geriatría y Gerontología*. 2020;56(2):75-80. doi:10.1016/j.regg.2020.10.005

54. Brunner WM, Hirabayashi L, Krupa NL, et al. Severe acute respiratory coronavirus virus 2 (SARS-CoV-2) IgG results among healthcare workers in a rural upstate New York hospital system. *Infection Control & Hospital Epidemiology*. Published online October 2020:1-4. doi:10.1017/ice.2020.1296

55. Carozzi FM, Cusi MG, Pistello M, et al. Detection of asymptomatic SARS-CoV-2 infections among healthcare workers: Results from a large-scale screening program based on rapid serological testing. *medRxiv*. Published online August 2020. doi:10.1101/2020.07.30.20149567

56. Vilibic-Cavlek T, Stevanovic V, Tabain I, et al. Severe acute respiratory syndrome coronavirus 2 seroprevalence among personnel in the healthcare facilities of Croatia, 2020. *Revista da Sociedade Brasileira de Medicina Tropical*. 2020;53. doi:10.1590/0037-8682-0458-2020

57. Chibwana MG, Jere KC, kamng'ona R, et al. High SARS-CoV-2 seroprevalence in Health Care Workers but relatively low numbers of deaths in urban Malawi. *medRxiv*. Published online August 2020:2020.07.30.20164970. doi:10.1101/2020.07.30.20164970

58. Coffman B. New Co-Immunity Project data show COVID-19 infection among health care workers may be lower than the general population | UofL News. *UofLNews*. Published online August 2020.

59. Cooper DJ, Lear S, Watson L, et al. A prospective study of risk factors associated with seroprevalence of SARS-CoV-2 antibodies in healthcare workers at a large UK teaching hospital. *medRxiv*. Published online November 2020:2020.11.03.20220699. doi:10.1101/2020.11.03.20220699

60. Denyer S. Japanese firm's blanket testing of employees could serve as model. *LMT Online*. Published online June 2020.

61. Dimeglio C, Herin F, Miedougé M, et al. Screening for SARS-CoV-2 antibodies among healthcare workers in a university hospital in southern France. *Journal of Infection*. 2020;0(0). doi:10.1016/j.jinf.2020.09.035

62. Fuereder T, Berghoff AS, Heller G, et al. SARS-CoV-2 seroprevalence in oncology healthcare professionals and patients with cancer at a tertiary care centre during the COVID-19 pandemic. *ESMO Open*. 2020;5(5). doi:10.1136/esmoopen-2020-000889

63. Fusco FM, Pisaturo M, Iodice V, et al. COVID-19 among healthcare workers in a specialist infectious diseases setting in Naples, Southern Italy: Results of a cross-sectional surveillance study. *Journal of Hospital Infection*. 2020;105(4):596-600. doi:10.1016/j.jhin.2020.06.021

64. Geraci L. Antibody tests show just 2% exposure rate to COVID-19. *The Lancaster News*. Published online May 2020.

65. Eduardo Samo Gudo. *Inquérito Sero-epidemiológico de SARS-CoV-2 na Cidade de Nampula*. República de Moçambique Ministério da Saúde; 2020:19.

66. Hackner K, Errhalt P, Willheim M, et al. Diagnostic accuracy of two commercially available rapid assays for detection of IgG and IgM antibodies to SARS-CoV-2 compared to ELISA in a low-prevalence population. *Research Square*. Published online August 2020. doi:10.21203/rs.3.rs-50887/v1

67. Haq M, Rehman A, Noor M, et al. Seroprevalence and Risk Factors of SARS CoV-2 in Health Care Workers of Tertiary-Care Hospitals in the Province of Khyber Pakhtunkhwa, Pakistan. *medRxiv*. Published online September 2020:2020.09.29.20203125. doi:10.1101/2020.09.29.20203125

68. He L, Zeng Y, Zeng C, et al. Positive Rate of Serology and RT-PCR for COVID-19 among healthcare workers during different periods in Wuhan, China. *Journal of Infection*. Published online August 2020. doi:10.1016/j.jinf.2020.08.027

69. Herzberg J, Vollmer T, Fischer B, et al. Prospective Sero-epidemiological Evaluation of SARS-CoV-2 among Health Care Workers in a German Secondary Care Hospital. *International Journal of Infectious Diseases*. 2021;102:136-143. doi:10.1016/j.ijid.2020.10.026

70. Jeremias A, Nguyen J, Levine J, et al. Prevalence of SARS-CoV-2 Infection Among Health Care Workers in a Tertiary Community Hospital. *JAMA Internal Medicine*. Published online August 2020. doi:10.1001/jamainternmed.2020.4214

71. Jespersen S, Mikkelsen S, Greve T, et al. Severe Acute Respiratory Syndrome Coronavirus 2 Seroprevalence Survey Among 17 971 Healthcare and Administrative Personnel at Hospitals, Prehospital Services, and Specialist Practitioners in the Central Denmark Region. *Clinical Infectious Diseases*. Published online October 2020:ciaa1471. doi:10.1093/cid/ciaa1471

72. Kassem AM, Talaat H, Shawky S, et al. SARS-CoV-2 infection among healthcare workers of a gastroenterological service in a tertiary care facility. *Arab Journal of Gastroenterology*. 2020;21(3):151-155. doi:10.1016/j.ajg.2020.07.005

73. Kern PM, Müller H-H, Menzel T, Weisser H. Studie zur Immunität gegen SARS-CoV-2: Keine signifikante humorale Immunität gegen SARS-CoV-2 im medizinischen Personal eines Klinikums der Maximalversorgung und in der Stadtregion Fulda. *Der Klinikarzt*. 2020;49(06):268-273. doi:10.1055/a-1198-1243

74. Khalil A, Hill R, Wright A, Ladhani S, O'Brien P. SARS-CoV-2-Specific Antibody Detection in Healthcare Workers in a UK Maternity Hospital: Correlation With SARS-CoV-2 RT-PCR Results. *Clinical Infectious Diseases*. 2020;(ciaa893). doi:10.1093/cid/ciaa893

75. Kumar A, Sathyapalan D, Ramachandran A, Subhash K, Biswas L, Beena KV. SARS-CoV-2 antibodies in healthcare workers in a large university hospital, Kerala, India. *Clinical Microbiology and Infection*. 2021;27(3):481-483. doi:10.1016/j.cmi.2020.09.013

76. Lackermair K, William F, Grzanna N, et al. Infection with SARS-CoV-2 in primary care health care workers assessed by antibody testing. *Family Practice*. Published online August 2020:cmaa078. doi:10.1093/fampra/cmaa078

77. Lahner E, Dilaghi E, Prestigiacomo C, et al. Prevalence of Sars-Cov-2 Infection in Health Workers (HWs) and Diagnostic Test Performance: The Experience of a Teaching Hospital in Central Italy. *International Journal of Environmental Research and Public Health*. 2020;17(12). doi:10.3390/ijerph17124417

78. Liu M, Cheng S-Z, Xu K-W, et al. Use of personal protective equipment against coronavirus disease 2019 by healthcare professionals in Wuhan, China: Cross sectional study. *BMJ*. 2020;369. doi:10.1136/bmj.m2195

79. Liu T, Wu S, Tao H, Zeng G, Zhou F, Wang X. Prevalence of IgG Antibodies to SARS-CoV-2 in Wuhan Implications for the Longevity of Antibodies Against SARS-CoV-2. *Research Square*. Published online November 2020. doi:10.21203/rs.3.rs-99748/v1

80. Lorenzo D, Carrisi C. COVID-19 exposure risk for family members of healthcare workers: An observational study. *International Journal of Infectious Diseases*. 2020;98:287-289. doi:10.1016/j.ijid.2020.06.106

81. Mussagy Mahomed. *Inquérito Sero-Epidemiológico de SARS-CoV-2 Na Cidade de Tete (InCOVID 2020)*. República de Moçambique Ministério da Saúde; 2020.

82. Arlete Mahumane. *Inquérito Sero-Epidemiológico de SARS-CoV-2 Na Cidade de Chimoio (InCOVID 2020)*. República de Moçambique Ministério da Saúde; 2020.

83. Majdoubi A, Michalski C, O'Connell SE, et al. Antibody reactivity to SARS-CoV-2 is common in unexposed adults and infants under 6 months. *medRxiv*. Published online November 2020:2020.10.05.20206664. doi:10.1101/2020.10.05.20206664

84. Majiya H, Aliyu-Paiko M, Balogu VT, et al. Seroprevalence of COVID-19 in Niger State. *medRxiv*. Published online August 2020. doi:10.1101/2020.08.04.20168112

85. Fill Malfertheiner S, Brandstetter S, Roth S, et al. Immune response to SARS-CoV-2 in health care workers following a COVID-19 outbreak: A prospective longitudinal study. *Journal of Clinical Virology*. 2020;130:104575. doi:10.1016/j.jcv.2020.104575

86. Martin C, Montesinos I, Dauby N, et al. Dynamics of SARS-CoV-2 RT-PCR positivity and seroprevalence among high-risk healthcare workers and hospital staff. *Journal of Hospital Infection*. 2020;106(1):102-106. doi:10.1016/j.jhin.2020.06.028

87. de Melo MS, Borges LP, de Souza DRV, et al. *Anti-SARS-CoV-2 IgM and IgG Antibodies in Health Workers in Sergipe, Brazil*. Infectious Diseases (except HIV/AIDS); 2020. doi:10.1101/2020.09.24.20200873

88. Morcuende M, Guglielminotti J, Landau R. Anesthesiologists' and Intensive Care Providers' Exposure to COVID-19 Infection in a New York City Academic Center: A Prospective Cohort Study Assessing Symptoms and COVID-19 Antibody Testing. *Anesthesia and analgesia*. 2020;131(3):669-676. doi:10.1213/ANE.0000000000005056

89. Moscola J, Sembajwe G, Jarrett M, et al. Prevalence of SARS-CoV-2 Antibodies in Health Care Personnel in the New York City Area. *JAMA*. 2020;324(9):893-895. doi:10.1001/jama.2020.14765

90. Nishida T, Iwahashi H, Yamauchi K, et al. Seroprevalence of SARS-CoV-2 Antibodies Among 925 Staff Members in an Urban Hospital Accepting COVID-19 Patients in Osaka Prefecture, Japan. *medRxiv*. Published online January 2020:2020.09.10.20191866. doi:10.1101/2020.09.10.20191866

91. Olalla J, Correa AM, Martín-Escalante MD, et al. Search for asymptomatic carriers of SARS-CoV-2 in healthcare workers during the pandemic: A Spanish experience. *QJM: An International Journal of Medicine*. 2020;(hcaa238). doi:10.1093/qjmed/hcaa238

92. Pallett SJC, Rayment M, Patel A, et al. Point-of-care serological assays for delayed SARS-CoV-2 case identification among health-care workers in the UK: A prospective multicentre cohort study. *The Lancet Respiratory Medicine*. 2020;8(9):885-894. doi:10.1016/S2213-2600(20)30315-5

93. Péré H, Wack M, Védie B, et al. Sequential SARS-CoV-2 IgG assays as confirmatory strategy to confirm equivocal results: Hospital-wide antibody screening in 3,569 staff health care workers in Paris. *Journal of Clinical Virology*. 2020;132:104617. doi:10.1016/j.jcv.2020.104617

94. Poulikakos D, Sinha S, Kalra PA. SARS-CoV-2 antibody screening in healthcare workers in a tertiary centre in North West England. *Journal of clinical virology : the official publication of the Pan American Society for Clinical Virology*. 2020;129:104545-104545. doi:10.1016/j.jcv.2020.104545

95. Psichogiou M, Karabinis A, Pavlopoulou I, et al. Antibodies against SARS-CoV-2 among health care workers in a country with low burden of COVID-19. *medRxiv*. Published online June 2020. doi:10.1101/2020.06.23.20137620

96. Kolthur-Seetharam U, Shah D, Shastri J, et al. *SARS-CoV2 Serological Survey in Mumbai by NITI-BMC-TIFR: Preliminary Report of Round-2*. NITI-BMC-TIFR; 2020.

97. Shields AM, Faustini SE, Perez-Toledo M, et al. SARS-CoV-2 seroconversion in health care workers. *medRxiv*. Published online May 2020:2020.05.18.20105197. doi:10.1101/2020.05.18.20105197

98. Ismael Amaral Silva PA, Ismael C, Marchon da Silva C, Domenge C. 1761P Universal screening of SARS-CoV-2 of oncology healthcare workers a Brazilian experience. *Annals of Oncology*. 2020;31:S1024. doi:10.1016/j.annonc.2020.08.1825

99. Solodky ML, Galvez C, Russias B, et al. Lower detection rates of SARS-COV2 antibodies in cancer patients versus health care workers after symptomatic COVID-19. *Annals of Oncology*. 2020;31(8):1087-1088. doi:10.1016/j.annonc.2020.04.475

100. Soriano V, Meiriño R, Corral O, Guallar MP. Severe Acute Respiratory Syndrome Coronavirus 2 Antibodies in Adults in Madrid, Spain. *Clinical Infectious Diseases*. 2020;(ciaa769). doi:10.1093/cid/ciaa769

101. Instituto Nazionale di Statistica. *PRIMI RISULTATI DELL'INDAGINE DI SIEROPREVALENZA SUL SARS-CoV-2*. Instituto Nazionale di Statistica; 2020.

102. Steensels D, Oris E, Coninx L, et al. Hospital-Wide SARS-CoV-2 Antibody Screening in 3056 Staff in a Tertiary Center in Belgium. *JAMA*. 2020;(7501160). doi:10.1001/jama.2020.11160

103. Stock AD, Bader ER, Cezayirli P, et al. COVID-19 Infection Among Healthcare Workers: Serological Findings Supporting Routine Testing. *Frontiers in Medicine*. 2020;7. doi:10.3389/fmed.2020.00471

104. Takita M, Matsumura T, Yamamoto K, et al. Geographical Profiles of COVID-19 Outbreak in Tokyo: An Analysis of the Primary Care ClinicBased Point-of-Care Antibody Testing. *Journal of Primary Care & Community Health*. 2020;11:215013272094269. doi:10.1177/2150132720942695

105. Tong X, Ning M, Huang R, et al. Surveillance of SARS-CoV-2 infection among frontline health care workers in Wuhan during COVID-19 outbreak. *Immunity, Inflammation and Disease*. 2020;8(4):840-843. doi:10.1002/iid3.340

106. Trieu M-C, Bansal A, Madsen A, et al. SARS-CoV-2Specific Neutralizing Antibody Responses in Norwegian Health Care Workers After the First Wave of COVID-19 Pandemic: A Prospective Cohort Study. *The Journal of Infectious Diseases*. 2020;2021-(jiaa737). doi:10.1093/infdis/jiaa737

107. Tu D, Shu J, Wu X, et al. Immunological detection of serum antibodies in pediatric medical workers exposed to varying levels of SARS-CoV-2. *The Journal of Infection*. 2021;82(1):159-198. doi:10.1016/j.jinf.2020.07.023

108. Valdivia A, Torres I, Huntley D, et al. Caveats in interpreting SARS-CoV-2 IgM+/IgG- antibody profile in asymptomatic health care workers. *Journal of Medical Virology*. 2020;n/a(n/a). doi:10.1002/jmv.26400

109. Chafloque-Vasquez RA, Pampa-Espinoza L, Salinas JCC. Seroprevalence of COVID-19 in workers in a hospital in the Peruvian Amazon. *ACTA MEDICA PERUANA*. 2020;37(3). doi:10.35663/amp.2020.373.1050

110. Edna Viegas. *Inquérito Sero-Epidemiológico de SARS-CoV-2 Na Cidade de Maputo (InCOVID 2020)*. República de Moçambique Ministério da Saúde; 2020.

111. Vlachoyiannopoulos P, Alexopoulos H, Apostolidi I, et al. Anti-SARS-CoV-2 antibody detection in healthcare workers of two tertiary hospitals in Athens, Greece. *Clinical Immunology*. 2020;221:108619. doi:10.1016/j.clim.2020.108619

112. Dalla Volta A, Valcamonico F, Pedersini R, et al. The Spread of SARS-CoV-2 Infection Among the Medical Oncology Staff of ASST Spedali Civili of Brescia: Efficacy of Preventive Measures. *Frontiers in Oncology*. 2020;10:1574. doi:10.3389/fonc.2020.01574

113. Ward H, Cooke G, Atchison C, et al. Declining prevalence of antibody positivity to SARS-CoV-2: A community study of 365,000 adults. *medRxiv*. Published online October 2020:2020.10.26.20219725. doi:10.1101/2020.10.26.20219725

114. Xiong S, Guo C, Dittmer U, Zheng X, Wang B. The prevalence of antibodies to SARS-CoV-2 in asymptomatic healthcare workers with intensive exposure to COVID-19. *medRxiv*. Published online June 2020:2020.05.28.20110767. doi:10.1101/2020.05.28.20110767

115. Zhang J, Liu J, Li N, et al. Serological detection of 2019-nCoV respond to the epidemic: A useful complement to nucleic acid testing. *medRxiv*. Published online March 2020:2020.03.04.20030916. doi:10.1101/2020.03.04.20030916

116. Zhao D, Wang M, Wang M, et al. Asymptomatic infection by SARS-CoV-2 in healthcare workers: A study in a large teaching hospital in Wuhan, China. *International Journal of Infectious Diseases*. 2020;99:219-225. doi:10.1016/j.ijid.2020.07.082

117. Ahmad K, Rezvanizadeh V, Dahal S, et al. COVID-19 IgG/IgM antibody testing in Los Angeles County, California. *European Journal of Clinical Microbiology & Infectious Diseases*. Published online November 2020. doi:10.1007/s10096-020-04111-3

118. Halbrook M, Gadoth A, Martin-Blais R, et al. Incidence of SARS-CoV-2 infection among asymptomatic frontline health workers in Los Angeles County, California. *medRxiv*. Published online November 2020:2020.11.18.20234211. doi:10.1101/2020.11.18.20234211

119. Iwuji K, Islam E, Berdine G, Nugent K, Test V, Tijerina A. Prevalence of Coronavirus Antibody Among First Responders in Lubbock, Texas. *Journal of Primary Care & Community Health*. 11:2150132720971390. doi:10.1177/2150132720971390

120. Parker-Magyar A. Few among Long Hill first responders test positive for COVID-19 antibodies. *Echoes Sentinel*. Published online June 2020.

121. Caban-Martinez AJ, Schaefer-Solle N, Santiago K, et al. Epidemiology of SARS-CoV-2 antibodies among firefighters/paramedics of a US fire department: A cross-sectional study. *Occupational and Environmental Medicine*. 2020;77(12):857-861. doi:10.1136/oemed-2020-106676

122. Staletovich J. South Florida Cities Begin Testing Employees For COVID-19 Antibodies. *WLRN*. Published online May 2020.

123. Hibino M, Iwabuchi S, Munakata H. SARS-CoV-2 IgG seroprevalence among medical staff in a general hospital that treated patients with COVID-19 in Japan: Retrospective evaluation of nosocomial infection control. *Journal of Hospital Infection*. 2020;107:103-104. doi:10.1016/j.jhin.2020.10.001

124. Calcagno A, Ghisetti V, Emanuele T, et al. Risk for SARS-CoV-2 Infection in Healthcare Workers, Turin, Italy. *Emerging Infectious Diseases*. 2021;27(1):303-305. doi:10.3201/eid2701.203027

125. Alharbi SA, Almutairi AZ, Jan AA, Alkhalify AM. Enzyme-Linked Immunosorbent Assay for the Detection of Severe Acute Respiratory Syndrome Coronavirus 2 (SARS-CoV-2) IgM/IgA and IgG Antibodies Among Healthcare Workers. *Cureus*. Published online September 2020. doi:10.7759/cureus.10285

126. Chau NVV, Toan LM, Man DNH, et al. Absence of SARS-CoV-2 antibodies in health care workers of a tertiary referral hospital for COVID-19 in southern Vietnam. *Journal of Infection*. 2020;82(1):e36-e37. doi:10.1016/j.jinf.2020.11.018

127. Khan MS, Haq I, Qurieshi MA, et al. SARS-CoV-2 seroprevalence in healthcare workers of dedicated-COVID hospitals and nonCOVID hospitals of District Srinagar, Kashmir. *medRxiv*. Published online October 2020:2020.10.23.20218164. doi:10.1101/2020.10.23.20218164

128. Costa SF, Giavina-Bianchi P, Buss L, et al. SARS-CoV-2 seroprevalence and risk factors among oligo/asymptomatic healthcare workers(HCW): Estimating the impact of community transmission. *Clinical Infectious Diseases*. 2020;(ciaa1845). doi:10.1093/cid/ciaa1845

129. Mohr N, Harland K, Krishnadasan A, Santibanez S, Talan D. Diagnosed and Undiagnosed COVID-19 in US Emergency Department Health Care Personnel: A Cross-sectional Analysis. *Annals of Emergency Medicine*. Published online December 2020. doi:10.1016/j.annemergmed.2020.12.007

130. Noor M, Haq M, Ul Haq N, et al. Does Working in a COVID-19 Receiving Health Facility Influence Seroprevalence to SARS-CoV-2? *Cureus*. Published online November 2020. doi:10.7759/cureus.11389

131. Singhal T, Shah S, Naik R, Kazi A, Thakkar P. Prevalence of COVID-19 Antibodies in Healthcare Workers at the Peak of the Pandemic in Mumbai, India: A Preliminary Study. *Indian Journal of Medical Microbiology*. 2020;38(3):461-463. doi:10.4103/ijmm.IJMM_20_308

132. Dacosta-Urbieta A, Rivero-Calle I, Pardo-Seco J, et al. Seroprevalence of SARS-CoV-2 Among Pediatric Healthcare Workers in Spain. *Frontiers in Pediatrics*. 2020;8. doi:10.3389/fped.2020.00547

133. Sartore-Bianchi A, Patelli G, Tosi F, et al. INCIDENCE OF SARS-COV-2 INFECTION IN PATIENTS WITH ACTIVE CANCER: MONO-INSTITUTIONAL SERIES OF A COMPREHENSIVE CANCER INSTITUTION IN LOMBARDY DURING THE COVID-19 PANDEMIC (NIGUARDA CANCER CENTER, MILANO, ITALY). In: *Tumori Journal*. Vol 106. AIOM Abstracts.; 2020:1-215. doi:10.1177/0300891620953388

134. Brousseau N, Morin L, Ouakki M, et al. *COVID-19 : Étude de séroprévalence chez des travailleurs de la santé de centres hospitaliers au Québec*. Institut National de Sante Publique du Quebec; 2020:20.

135. Chen Y, Tong X, Wang J, et al. High SARS-CoV-2 antibody prevalence among healthcare workers exposed to COVID-19 patients. *The Journal of Infection*. 2020;81(3):420-426. doi:10.1016/j.jinf.2020.05.067

136. Favara DM, Cooke A, Doffinger R, McAdam K, Corrie P, Ainsworth NL. COVID-19 Serology in Oncology Staff Study: Understanding SARS-CoV-2 in the Oncology Workforce. *Clinical Oncology (Royal College of Radiologists (Great Britain)*. 2021;33(1):e61-e63. doi:10.1016/j.clon.2020.07.015

137. Fujita K, Shinpei Kada, Osamu Kanai, et al. Quantitative SARS-CoV-2 antibody screening of healthcare workers in the southern part of Kyoto city during the COVID-19 peri-pandemic period. *medRxiv*. Published online May 2020.

138. Godbout EJ, Pryor R, Harmon M, et al. Severe acute respiratory coronavirus virus 2 (SARS-CoV-2) seroprevalence among healthcare workers in a low prevalence region. *Infection Control & Hospital Epidemiology*. Published online December 2020:1-3. doi:10.1017/ice.2020.1374

139. Houlihan CF, Vora N, Byrne T, et al. Pandemic peak SARS-CoV-2 infection and seroconversion rates in London frontline health-care workers. *The Lancet*. 2020;396(10246):e6-e7. doi:10.1016/S0140-6736(20)31484-7

140. Insúa C, Stedile G, Figueroa V, et al. Seroprevalence of SARS-CoV-2 antibodies among physicians from a children's hospital. *Archivos Argentinos De Pediatria*. 2020;118(6):381-385. doi:10.5546/aap.2020.eng.381

141. Kohler PP, Kahlert CR, Sumer J, et al. Prevalence of SARS-CoV-2 antibodies among Swiss hospital workers: Results of a prospective cohort study. *Infection Control & Hospital Epidemiology*. Published online October 2020:1-5. doi:10.1017/ice.2020.1244

142. Kumar N, Bhartiya S, Singh T. Duration of anti-SARS-CoV-2 antibodies much shorter in India. *Vaccine*. 2021;39(6):886-888. doi:10.1016/j.vaccine.2020.10.094

143. Mesnil M, Joubel K, Yavchitz A, Miklaszewski N, Devys J-M. Seroprevalence of SARS-Cov-2 in 646 professionals at the Rothschild Foundation Hospital (ProSeCoV study). *Anaesthesia Critical Care & Pain Medicine*. 2020;39(5):595-596. doi:10.1016/j.accpm.2020.08.003

144. Missaglia R, Belingheri M, Antolini L, et al. SARS-CoV-2 pandemia in Lombardy: The impact on family Paediatricians. *Italian Journal of Pediatrics*. 2020;46(1):184. doi:10.1186/s13052-020-00950-0

145. Orth-Höller D, Eigentler A, Weseslindtner L, Möst J. Antibody kinetics in primary- and secondary-care physicians with mild to moderate SARS-CoV-2 infection. *Emerging Microbes & Infections*. 2020;9(1):1692-1694. doi:10.1080/22221751.2020.1793690

146. Plebani M, Padoan A, Fedeli U, et al. SARS-CoV-2 serosurvey in health care workers of the Veneto Region. *Clinical Chemistry and Laboratory Medicine (CCLM)*. 2020;58(12):2107-2111. doi:10.1515/cclm-2020-1236

147. Rudberg A-S, Havervall S, Månberg A, et al. SARS-CoV-2 exposure, symptoms and seroprevalence in healthcare workers in Sweden. *Nature Communications*. 2020;11(1):5064. doi:10.1038/s41467-020-18848-0

148. Schmidt SB, Grüter L, Boltzmann M, Rollnik JD. Prevalence of serum IgG antibodies against SARS-CoV-2 among clinic staff. Adrish M, ed. *PLOS ONE*. 2020;15(6):e0235417. doi:10.1371/journal.pone.0235417

149. Sotgiu G, Barassi A, Miozzo M, et al. SARS-CoV-2 specific serological pattern in healthcare workers of an Italian COVID-19 forefront hospital. *BMC Pulmonary Medicine*. 2020;20(1):203. doi:10.1186/s12890-020-01237-0

150. Venugopal U, Jilani N, Rabah S, et al. SARS-CoV-2 seroprevalence among health care workers in a New York City hospital: A cross-sectional analysis during the COVID-19 pandemic. *International Journal of Infectious Diseases*. 2020;102:63-69. doi:10.1016/j.ijid.2020.10.036

151. Racine-Brzostek SE, Yang HS, Chadburn A, et al. COVID-19 Viral and Serology Testing in New York City Health Care Workers. *American Journal of Clinical Pathology*. 2020;154(5):592-595. doi:10.1093/ajcp/aqaa142

152. Hoffmann S, Spallek J, Heinz-Detlef G, Schiebel J, Hufert F. Testing the backbone of the healthcare system: A prospective serological-epidemiological cohort study of healthcare workers in rural Germany. Published online September 2020. doi:10.21203/rs.3.rs-84703/v1

153. Patel MM, Thornburg NJ, Stubblefield WB, et al. Change in Antibodies to SARS-CoV-2 Over 60 Days Among Health Care Personnel in Nashville, Tennessee. *JAMA*. 2020;324(17):1781. doi:10.1001/jama.2020.18796

154. Self WH, Tenforde MW, Stubblefield WB, et al. Seroprevalence of SARS-CoV-2 Among Frontline Health Care Personnel in a Multistate Hospital Network 13 Academic Medical Centers, AprilJune 2020. *MMWR Morbidity and Mortality Weekly Report*. 2020;69(35):1221-1226. doi:10.15585/mmwr.mm6935e2

155. Shah VP, Hainy CM, Swift MD, Breeher LE, Theel ES, Sampathkumar P. Unrecognized severe acute respiratory coronavirus virus 2 (SARS-CoV-2) seroprevalence among healthcare personnel in a low-prevalence area. *Infection Control & Hospital Epidemiology*. Published online November 2020:1-3. doi:10.1017/ice.2020.1341

156. Bampoe S, Lucas DN, Neall G, et al. A cross-sectional study of immune seroconversion to SARS-CoV-2 in front-line maternity health professionals. *medRxiv*. Published online June 2020. doi:10.1101/2020.06.24.20139352

157. Dimcheff DE, Schildhouse RJ, Hausman MS, et al. Seroprevalence of severe acute respiratory syndrome coronavirus-2 (SARS-CoV-2) infection among Veterans Affairs healthcare system employees suggests higher risk of infection when exposed to SARS-CoV-2 outside the work environment. *Infection Control & Hospital Epidemiology*.:1-7. doi:10.1017/ice.2020.1220

158. Finkenzeller T, Faltlhauser A, Dietl K-H, et al. SARS-CoV-2-Antikörper bei Intensiv- und Klinikpersonal. *Medizinische Klinik - Intensivmedizin und Notfallmedizin*. 2020;115(3):139-145. doi:10.1007/s00063-020-00761-5

159. Grant JJ, Wilmore SMS, McCann NS, et al. Seroprevalence of SARS-CoV-2 antibodies in healthcare workers at a London NHS Trust. *Infection Control & Hospital Epidemiology*. Published online August 2020:1-3. doi:10.1017/ice.2020.402

160. Mansour M, Leven E, Muellers K, Stone K, Mendu DR, Wajnberg A. Prevalence of SARS-CoV-2 Antibodies Among Healthcare Workers at a Tertiary Academic Hospital in New York City. *Journal of General Internal Medicine*. 2020;35(8):2485-2486. doi:10.1007/s11606-020-05926-8

161. Martín V, Fernández-Villa T, Lamuedra Gil de Gomez M, et al. Prevalence of SARS-CoV-2 infection in general practitioners and nurses in primary care and nursing homes in the Healthcare Area of León and associated factors. *COVID19 en Atención Primaria*. 2020;46:35-39. doi:10.1016/j.semerg.2020.05.014

162. Meissner EG, Litwin C, Crocker T, Mack E, Card L. 460. Point-of-Care, In-Home SARS-CoV-2 IgG Antibody Testing to Assess Seroprevalence in At-Risk Health Care Workers. *Open Forum Infectious Diseases*. 2020;7(Supplement_1):S297-S297. doi:10.1093/ofid/ofaa439.653

163. Mostafa A, Kandil S, El-Sayed MH, et al. Universal COVID-19 screening of 4040 health care workers in a resource-limited setting: An Egyptian pilot model in a university with 12 public hospitals and medical centers. *International Journal of Epidemiology*. 2020;(dyaa173). doi:10.1093/ije/dyaa173

164. Paradiso AV, Summa simona D, Silvestris N, et al. COVID-19 SCREENING AND MONITORING OF ASYMPTOMATIC HEALTH WORKERS WITH A RAPID SEROLOGICAL TEST. *medRxiv*. Published online May 2020:2020.05.05.20086017. doi:10.1101/2020.05.05.20086017

165. Sydney ER, Kishore P, Laniado I, Rucker LM, Bajaj K, Zinaman MJ. Antibody evidence of SARS-CoV-2 infection in healthcare workers in the Bronx. *Infection Control & Hospital Epidemiology*. 2020;41(11):1348-1349. doi:10.1017/ice.2020.437

166. Zhou F, Li J, Lu M, et al. Tracing asymptomatic SARS-CoV-2 carriers among 3674 hospital staff:A cross-sectional survey. *EClinicalMedicine*. 2020;26. doi:10.1016/j.eclinm.2020.100510

167. Buntinx F, Claes P, Gulikers M, et al. Added value of anti-SARS-CoV-2 antibody testing in a Flemish nursing home during an acute COVID-19 outbreak in April 2020. *Acta Clinica Belgica*. 2020;0(0):1-6. doi:10.1080/17843286.2020.1834285

168. Reuben J, Sherman A, Ellison JA, et al. SARS-CoV-2 Seroprevalence among First Responders in the District of Columbia, May July 2020. *medRxiv*. Published online November 2020:2020.11.25.20225490. doi:10.1101/2020.11.25.20225490

169. Saberian P, Mireskandari SM, Baratloo A, et al. Antibody Rapid Test Results in Emergency Medical Services Personnel during COVID-19 Pandemic; a Cross Sectional study. *Archives of Academic Emergency Medicine*. 2020;9(1).

170. Tarabichi Y, Watts B, Collins T, et al. SARS-CoV-2 Infection among Serially Tested Emergency Medical Services Workers. *Prehospital Emergency Care*. 2020;0(0):1-7. doi:10.1080/10903127.2020.1831668

171. Vijh R, Ghafari C, Hayden A, et al. Serological survey following SARS-COV-2 outbreaks at long-term care facilities in metro Vancouver, British Columbia: Implications for outbreak management and infection control policies. *American Journal of Infection Control*. Published online October 2020. doi:10.1016/j.ajic.2020.10.009

172. Bhattacharya D, Winnett A, Fulcher JA, et al. 70. Lack of SARS-CoV-2 Antibody Seroconversion After Prompt Identification and Cohorting of Sentinel sars-cov-2-positive Residents in a Skilled Nursing Facility. *Open Forum Infectious Diseases*. 2020;7(Supplement_1):S165-S166. doi:10.1093/ofid/ofaa439.380

173. Pérez-García F, Pérez-Zapata A, Arcos N, et al. Severe acute respiratory coronavirus virus 2 (SARS-CoV-2) infection among hospital workers in a severely affected institution in Madrid, Spain: A surveillance cross-sectional study. *Infection Control & Hospital Epidemiology*. Published online October 2020:1-7. doi:10.1017/ice.2020.1303

174. Pérez-García F, Pérez-Zapata A, Arcos N, et al. Severe acute respiratory coronavirus virus 2 (SARS-CoV-2) infection among hospital workers in a severely affected institution in Madrid, Spain: A surveillance cross-sectional study. *Infection Control & Hospital Epidemiology*. 2021;42(7):803-809. doi:10.1017/ice.2020.1303

175. Mughal MS, Kaur IP, Patton CD, Mikhail NH, Vareechon C, Granet KM. The prevalence of severe acute respiratory coronavirus virus 2 (SARS-CoV-2) IgG antibodies in intensive care unit (ICU) healthcare personnel (HCP) and its implicationsa single-center, prospective, pilot study. *Infection Control & Hospital Epidemiology*. Published online June 2020:1-2. doi:10.1017/ice.2020.298

176. Rao S. Covid-19: Jayadeva says its survey hints at herd immunity. *The Times of India*. Published online June 2020.

177. Shukla V, Lau CSM, Towns M, et al. COVID-19 Exposure Among First Responders in Arizona. *Journal of Occupational and Environmental Medicine*. 2020;62(12).

178. Gray A. Prevalence Of COVID-19 Antibodies In Washoe Co. Expected To Be Low. *KUNR*. Published online June 2020.

179. Chughtai O, Batool H, Khan M, Chughtai A. Frequency of COVID-19 IgG Antibodies among Special Police Squad Lahore, Pakistan. *Journal of the College of Physicians and Surgeons Pakistan*. 2020;30(7):735-739. doi:10.29271/jcpsp.2020.07.735

180. Gujski M, Jankowski M, Pinkas J, et al. Prevalence of Current and Past SARS-CoV-2 Infections among Police Employees in Poland, JuneJuly 2020. *Journal of Clinical Medicine*. 2020;9(10):3245. doi:10.3390/jcm9103245

181. Jerónimo Langa. *Inquérito Sero-Epidemiológico de SARS-CoV-2 Na Cidade de Lichinga (InCOVID 2020)*. República de Moçambique Ministério da Saúde; 2020.

182. Ivalda Macicame. *Inquérito Sero-Epidemiológico de SARS-CoV-2 Na Província de Maputo (InCOVID 2020)*. República de Moçambique Ministério da Saúde; 2020.

183. Al-Thani MH, Farag E, Bertollini R, et al. Seroprevalence of SARS-CoV-2 infection in the craft and manual worker population of Qatar. *medRxiv*. Published online November 2020:2020.11.24.20237719. doi:10.1101/2020.11.24.20237719

184. Epstude J, Harsch IA. Seroprevalence of COVID-19 antibodies in the cleaning and oncological staff of a municipal clinic. *GMS Hygiene and Infection Control; 15:Doc18*. Published online July 2020. doi:10.3205/DGKH000353

185. Hassan SS, Seigerud Å, Mühr LSA, et al. SARS-CoV-2 infections among personnel providing home care services for the elderly in Stockholm, Sweden. *medRxiv*. Published online December 2020. doi:10.1101/2020.12.18.20248511

186. Ladhani SN, Jeffery-Smith A, Patel M, et al. High prevalence of SARS-CoV-2 antibodies in care homes affected by COVID-19: Prospective cohort study, England. *EClinicalMedicine*. 2020;28. doi:10.1016/j.eclinm.2020.100597

187. Lindahl JF, Hoffman T, Esmaeilzadeh M, et al. High seroprevalence of SARS-CoV-2 in elderly care employees in Sweden. *Infection Ecology & Epidemiology*. 2020;10(1):1789036. doi:10.1080/20008686.2020.1789036

188. Regan T. Fellowship Village Benefits from Covid-19 Antibody Tests. *Senior Housing News*. Published online June 2020.

189. Alali WQ, Bastaki H, Longenecker JC, et al. Seroprevalence of SARS-CoV-2 in migrant workers in Kuwait. *Journal of Travel Medicine*. 2020;(taaa223). doi:10.1093/jtm/taaa223

190. Addetia A, Crawford KHD, Dingens A, et al. Neutralizing Antibodies Correlate with Protection from SARS-CoV-2 in Humans during a Fishery Vessel Outbreak with a High Attack Rate. McAdam AJ, ed. *Journal of Clinical Microbiology*. 2020;58(11):e02107-20, /jcm/58/11/JCM.02107-20.atom. doi:10.1128/JCM.02107-20

191. Picon RV, Carreno I, da Silva AA, et al. Coronavirus disease 2019 population-based prevalence, risk factors, hospitalization, and fatality rates in southern Brazil. *International Journal of Infectious Diseases*. 2020;100:402-410. doi:10.1016/j.ijid.2020.09.028

192. D B, L B, P T, Pa P, A B, U L. Effectiveness of the measures aimed at containing Sars-cov-2 virus spreading in work settings: A survey in companies based in the Veneto region of Italy. *La Medicina del lavoro*. Published online October 2020. doi:10.23749/mdl.v111i5.10037

193. Xu X, Sun J, Nie S, et al. Seroprevalence of immunoglobulin M and G antibodies against SARS-CoV-2 in China. *Nature Medicine*. 2020;26(8):1193-1195. doi:10.1038/s41591-020-0949-6

194. Chamie G, Marquez C, Crawford E, et al. Community Transmission of Severe Acute Respiratory Syndrome Coronavirus 2 Disproportionately Affects the Latinx Population During Shelter-in-Place in San Francisco. *Clinical Infectious Diseases*. Published online August 2020:ciaa1234. doi:10.1093/cid/ciaa1234

195. McLaughlin C, Doll MK, Morrison KT, et al. High Community SARS-CoV-2 Antibody Seroprevalence in a Ski Resort Community, Blaine County, Idaho, US. Preliminary Results. *medRxiv*. Published online July 2020. doi:10.1101/2020.07.19.20157198

196. Muñoz L, Pífano M, Bolzán A, et al. *Surveillance and Seroprevalence: Evaluation of IgG Antibodies for SARS-Cov2 by ELISA in the Popular Neighborhood Villa Azul, Quilmes, Province of Buenos Aires, Argentina*.; 2020. doi:10.1590/SciELOPreprints.1147
