# Supplementary material for: Occupation and SARS-CoV-2 seroprevalence studies: a systematic review
Source: BMJ Open. 2023 Feb 28;13(2):e063771. doi: 10.1136/bmjopen-2022-063771 (PMC9979591; doi:10.1136/bmjopen-2022-063771)
Supplement: Author's manuscript [file bmjopen-2022-063771.draft_revisions.pdf]

BMJ Open is committed to open peer review. As part of this commitment we make the peer review history of every article we publish publicly available.

When an article is published we post the peer reviewers' comments and the authors' responses online. We also post the versions of the paper that were used during peer review. These are the versions that the peer review comments apply to.

The versions of the paper that follow are the versions that were submitted during the peer review process. They are not the versions of record or the final published versions. They should not be cited or distributed as the published version of this manuscript.

BMJ Open is an open access journal and the full, final, typeset and author-corrected version of record of the manuscript is available on our site with no access controls, subscription charges or pay-per-view fees (http://bmjopen.bmj.com).

If you have any questions on BMJ Open's open peer review process please email info.bmjopen@bmj.com

BMJ Open

Occupation and SARS-CoV-2 seroprevalence studies: a systematic review

Journal:	*BMJ Open*
Manuscript ID	bmjopen-2022-063771
Article Type:	Original research
Date Submitted by the Author:	20-Apr-2022

Complete List of Authors:	Boucher, Emily; University of Calgary Cumming School of Medicine,<br>Cao, Christian; University of Calgary, Cumming School of Medicine<br>D'Mello, Sean; University of Waterloo<br>Duarte, Nathan; McGill University, Faculty of Engineering<br>Donnici, Claire; University of Calgary, Cumming School of Medicine<br>Duarte, Natalie; University of Toronto, Faculty of Arts and Science<br>Bennett, Graham; McGill University, Department of Economics<br>Consortium, SeroTracker ; University of Calgary<br>Adisesh, Anil; Unity Health Toronto, St. Michael's Hospital; University of Toronto, Division of Occupational Medicine<br>Arora, Rahul; Oxford University, Institute of Biomedical Engineering<br>Kodama, David; Unity Health Toronto, St. Michael's Hospital; University of Toronto Department of Medicine, Division of Emergency Medicine<br>Bobrovitz, Niklas; University of Toronto Temerty Faculty of Medicine; University of Calgary, Department of Critical Care Medicine

Keywords:	COVID-19, Public health < INFECTIOUS DISEASES, OCCUPATIONAL & INDUSTRIAL MEDICINE

SCHOLARONE™
Manuscripts

BMJ

I, the Submitting Author has the right to grant and does grant on behalf of all authors of the Work (as defined in the below author licence), an exclusive licence and/or a non-exclusive licence for contributions from authors who are: i) UK Crown employees; ii) where BMJ has agreed a CC-BY licence shall apply, and/or iii) in accordance with the terms applicable for US Federal Government officers or employees acting as part of their official duties; on a worldwide, perpetual, irrevocable, royalty-free basis to BMJ Publishing Group Ltd ("BMJ") its licensees and where the relevant Journal is co-owned by BMJ to the co-owners of the Journal, to publish the Work in this journal and any other BMJ products and to exploit all rights, as set out in our licence.

The Submitting Author accepts and understands that any supply made under these terms is made by BMJ to the Submitting Author unless you are acting as an employee on behalf of your employer or a postgraduate student of an affiliated institution which is paying any applicable article publishing charge ("APC") for Open Access articles. Where the Submitting Author wishes to make the Work available on an Open Access basis (and intends to pay the relevant APC), the terms of reuse of such Open Access shall be governed by a Creative Commons licence – details of these licences and which Creative Commons licence will apply to this Work are set out in our licence referred to above.

Other than as permitted in any relevant BMJ Author's Self Archiving Policies, I confirm this Work has not been accepted for publication elsewhere, is not being considered for publication elsewhere and does not duplicate material already published. I confirm all authors consent to publication of this Work and authorise the granting of this licence.

For peer review only

Occupation and SARS-CoV-2 seroprevalence studies: a systematic review

Emily Boucher,[1] Christian Cao[1], Sean D'Mello,[2] Nathan Duarte,[3] Claire Donnici[1], Natalie Duarte,[4] Graham Bennett,[5] SeroTracker Consortium, Anil Adisesh,[6-8] Rahul K. Arora,[1,9] David Kodama,[6,10] Niklas Bobrovitz[11,12]

1. Cumming School of Medicine, University of Calgary, Calgary, AB, Canada
2. Faculty of Engineering, University of Waterloo, Waterloo, ON, Canada
3. Faculty of Engineering, McGill University, Montreal, QC, Canada
4. Faculty of Arts and Science, University of Toronto, ON, Canada
5. Department of Economics, Faculty of Arts, McGill University, Montreal, QC, Canada
6. St. Michael's Hospital, Unity Health Toronto, Toronto, ON, Canada
7. Division of Occupational Medicine, Department of Medicine, University of Toronto, Toronto, ON, Canada
8. Canadian Health Solutions, Saint John, NB, Canada
9. Institute of Biomedical Engineering, University of Oxford, Oxford, UK
10. Division of Emergency Medicine, Department of Medicine, University of Toronto, Toronto, ON, Canada
11. Temerty Faculty of Medicine, University of Toronto, Toronto, ON, Canada
12. Department of Critical Care Medicine, University of Calgary, Calgary, AB, Canada

*Correspondence to Dr. Niklas Bobrovitz, Temerty Faculty of Medicine, University of Toronto, Toronto, ON, Canada; niklas.bobrovitz@mail.utoronto.ca

For peer review only

Word Count 1179

Key Words Covid-19, Infectious diseases**,** Occupational & industrial medicine

Key Messages

1. **What is already known about this subject?**

   Worldwide, workplace outbreaks of COVID-19 have been frequent, and some of the largest reported. Accurate data on the risks of infection with SARS-CoV-2 infection and other respiratory infections across a variety of occupations are needed to inform public health interventions.

2. **What are the new findings?**

   During the first year of the pandemic, a large number of seroprevalence studies covering a broad range of occupations globally were published. Rsults suggest considerable differences in the risk of SARS-CoV-2 infection between occupations.

3. **How might this impact on policy or clinical practice in the foreseeable future?**

   Occupation appears to be an important correlate of SARS-CoV-2 infection. Additional high-quality, well-powered serosurveys would improve our understanding of the occupational risks of SARS-CoV-2 and other respiratory infections and should be considered an essential component of the pandemic response.

Key Messages

For peer review only

For peer review only - http://bmjopen.bmj.com/site/about/guidelines.xhtml

ABSTRACT

Objective. To describe and synthesize studies of SARS-CoV-2 seroprevalence by occupation prior to the widespread vaccine rollout.

Methods. We identified studies of occupational seroprevalence from a living systematic review (PROSPERO CRD42020183634). Electronic databases, gray literature, and news media were searched for studies published January-December 2020. Seroprevalence estimates and a free text description of the occupation were extracted and classified according to the Standard Occupational Classification (SOC) 2010 system using a machine-learning algorithm. Due to heterogeneity, results were synthesized narratively.

Results. We identified 196 studies including 591,940 participants from 38 countries. Most studies (n=162; 83%) were conducted locally vs regionally or nationally. Sample sizes were generally small (median=220 participants per occupation) and 135 studies (69%) were at a high risk of bias. One or more estimates were available for 21/23 major SOC occupation groups, but over half of the estimates identified (n=359/600) were for healthcare-related occupations. 'Personal Care and Service Occupations' (median 22% [IQR 9-28%]; n=14) had the highest median seroprevalence.

Conclusions. Many seroprevalence studies covering a broad range of occupations were published in the first year of the pandemic. Results suggest considerable differences in seroprevalence between occupations, although few large, high-quality studies were done. Well-designed studies are required to improve our understanding of the occupational risk of SARS-CoV-2 and should be considered as an element of pandemic preparedness for future respiratory pathogens.

For peer review only

INTRODUCTION

Occupation is a social determinant of health and an important risk factor for SARS-CoV-2 infection. Essential workers in health and social care occupations have an increased risk of COVID-19 compared to non-essential workers, but the risks for other occupations are not well defined.[1-3] Studies using diagnostic or rapid testing results from health system and administrative data to examine occupational COVID-19 risk are affected by variable testing rates and access (e.g. due to workplace testing, paid sick leave, geographic, socio-economic factors). Few high-quality, prospective studies using frequent, serial diagnostic or rapid testing covering a broad range of occupations having been conducted.[4]

Serologic testing for SARS-CoV-2 antibodies provides evidence of previous infection and/or vaccination depending on vaccination status and the specific antigens targeted and can be used to obtain more accurate estimates of the cumulative incidence of infection.[5] Accurate data on the occupational risks of COVID-19 and other respiratory infections are essential for informing compliance with workplace safety regulations, transmission control measures and resource allocation (testing, personal protective equipment (PPE), etc.). The objectives of this review were to describe and synthesize studies of SARS-CoV-2 seroprevalence across a broad range of occupations globally prior to the widespread rollout of vaccines.

METHODS

We identified studies of occupational seroprevalence from a living systematic review (PROSPERO CRD42020183634) of >1000 seroprevalence studies.[6] In brief, electronic databases, grey literature, and news media were searched for cohort or cross-sectional studies

For peer review only

reporting antibody testing for SARS-CoV-2. Records were screened independently, in duplicate. We restricted eligibility to studies that in English, French or that were machine-translatable and published January-December 2020 before vaccines were rolled-out, because differential vaccination rates by occupation would obscure results. We extracted seroprevalence estimates with a free-text description for each occupation. If multiple estimates were reported, the most recent estimate using laboratory-based methods (e.g. ELISA), and anti-spike and/or IgG antibodies were prioritized, because non-IgG and anti-nucleocapsid antibodies may decline more rapidly.[7] Study-level risk of bias was assessed with a modified Joanna Briggs Institute Checklist for Prevalence Studies **(Table S1)**.[8]

For each seroprevalence estimate, we identified the relevant Standard Occupational Classification (SOC) 2010 codes by applying the National Institute for Occupational Safety & Health (NIOSH) Industry and Occupation Computerized Coding System (NIOCCS) to occupation descriptions.[9] NIOCCS was chosen, because most studies were conducted in the USA. Coding was manually verified if there was insufficient information for classification or the probability of correct classification was <0.8. Anticipating substantial heterogeneity and an insufficient number of estimates relative to covariates for meta-regression, we planned to summarize data using the median/IQR.

Patient and Public Involvement: It was not possible or appropriate to involve patients or the public in this study.

For peer review only

RESULTS

We identified 196 studies of occupational seroprevalence conducted in 2020 during the first and second waves of the pandemic. There were 591,940 participants from 38 countries, including the USA (n=44 studies), UK (n=16) and Italy (n=15). Most studies (n=162; 83%) were conducted locally (e.g. city, county) as opposed to regionally (e.g. state; n=20; 10%) or nationally (n=14; 7%). Most were restricted to one occupational group (n=103), limiting direct comparisons (i.e. using the same reference group). Sample sizes were often small (median=220, IQR 64-568 participants). Overall, 135 studies (69%) were at a high risk of bias, 47 moderate (24%), 2 low (1%) and 12 unclear (6%). Common reasons for bias were inadequate statistical analysis (i.e. no adjustment for test or sample characteristics; 92%), non-probability sampling (74%), and small sample-size (46%).

At least one estimate was available for all 23 major SOC occupation groups, except for 'Legal' and 'Military-Specific' occupations (**Figure 1**; all studies). Over half of the 600 estimates identified (n=359) were for healthcare-related occupations. For SOC groups with three or more estimates, the highest median seroprevalence was reported for 'Personal Care and Service Occupations' (median 22% [IQR 9-28%]; n=14, e.g. 'Personal Care Aids'). The next highest was reported for 'Building and Grounds Cleaning and Maintenance' occupations (11% [3-22%]; n=17, e.g. 'Maids and Housekeeping Cleaners'), and 'Healthcare Support' (11% [2-20%]; n=39, e.g. 'Nursing Assistants') occupations. The lowest median seroprevalence was 1% (0-11%; n=6, e.g. 'Athletes') for 'Arts, Design, Entertainment, Sports, and Media Occupations.' Individual estimates are listed in **Table S2**.

For peer review only - http://bmjopen.bmj.com/site/about/guidelines.xhtml

DISCUSSION

This review is the first comprehensive synthesis of occupational COVID-19 seroprevalence studies world-wide. We identified 196 studies representing 21 out of 23 major SOC groups conducted during the first and second waves of the SARS-CoV-2 pandemic in 2020, prior to the widespread rollout of vaccines, and described occupational groups with high seroprevalence.

Seroprevalence studies may estimate the cumulative incidence of infection more accurately than diagnostic testing studies when access to testing is variable.[2,4] The data identified suggest considerable differences in seroprevalence by occupation, though we did not statistically test for differences due to considerable variation in geography, study dates and workplace determinants of infection (e.g. PPE, ventilation). 'Caring and Personal Service' occupations had the highest median seroprevalence (22%), which was four-times higher than the unemployed (5%) and median seroprevalence across all occupational groups (5%). The UK Office for National Statistics reported a slightly lower mean risk of a positive diagnostic or rapid test for COVID-19 across 25 occupational groups of 4%,[10] but the discrepancy between the true cumulative incidence and confirmed infections is likely greater in regions with less access to testing: national, population-based serosurveys have estimated there are 10-20 serologically identifiable cases per one confirmed case.[6]

In future pandemics, large, well-reported, high-quality seroprevalence studies across a broad range of occupations are needed at an early stage to inform appropriate workplace policy. It has been suggested that 20% of the US workforce was exposed to disease or infection at work at

For peer review only - http://bmjopen.bmj.com/site/about/guidelines.xhtml

least once a month prior to the pandemic.[11] Public health agencies require accurate data on the occupational risks of respiratory infections, including SARS-CoV-2, to inform compliance with workplace safety regulations, transmission control measures and allocate limited resources (e.g. testing, personal protective equipment and vaccines) during outbreaks and pandemics. For governments, there are also issues of occupational disease recognition and compensation to be considered. As such, public health agencies and governments may be best positioned to coordinate these types of studies, as opposed to academic institutions,[6] which led the majority of serosurveys in the first year of the pandemic.

Strengths and Limitations

Despite the large number of studies of occupational seroprevalence conducted, many studies had methodological limitations. Only two studies were at a low risk of bias and most occupational subgroups had small sample sizes (median 220 participants). Many were limited to one major SOC group (n=103 studies), which precluded comparisons. Detailed descriptions of occupations were often lacking, potentially contributing to coding errors, and workplace determinants of infection (e.g. use of PPE) were poorly reported.

In conclusion, our review shows that a large number of seroprevalence studies covering a broad range of occupations were published in the first year of the pandemic. Results suggest considerable differences in seroprevalence between occupations, although few large, well-reported, high-quality studies were done. Carefully-designed, adequately powered seroprevalence studies with coverage of a broad range of occupations could improve our

For peer review only

For peer review only - http://bmjopen.bmj.com/site/about/guidelines.xhtml

understanding of the occupational risk of SARS-CoV-2 and other respiratory infections and should be considered an element of pandemic preparedness.

For peer review only

For peer review only - http://bmjopen.bmj.com/site/about/guidelines.xhtml

Acknowledgements and funding disclosure

SeroTracker receives funding for SARS-CoV-2 seroprevalence study evidence synthesis from the Public Health Agency of Canada through Canada's COVID-19 Immunity Task Force, the World Health Organization Health Emergencies Programme, the Robert Koch Institute, and the Canadian Medical Association Joule Innovation Fund. No funding source had any role in the design of this study, its execution, analyses, interpretation of the data, or decision to submit results. This manuscript does not necessarily reflect the views of the World Health Organization or any other funder.

Statement of author's contributions

This secondary analysis of the SeroTracker database was conceived by NB, EB, DK and AA. Senior authors on this paper were NB, DK, RA and AA. The protocol was developed by EB, NB and DK. Data cleaning was performed by CC, CD, ND, SD and EB and verification by EB, SD, ND and GB. Analysis was performed by EB and RA. The first draft of the manuscript was written by EB and revised by EB, RA, NB, ND, GB, SD, CC, AA, DK. The SeroTracker Consortium maintained the living systematic review database used in the study. All authors reviewed and agreed to the findings, and also provided critical revisions to the paper.

Disclosure of potential and actual conflicts of interest

RKA was previously a Technical Consultant for the Bill and Melinda Gates Foundation Strategic Investment Fund, is a minority shareholder of Alethea Medical, and was a former Senior Policy Advisor at Health Canada. Each of these relationships is unrelated to the present work.

For peer review only

JP reports grants to his institution from MedImmune, Sanofi Pasteur, Merck and AbbVie, and personal fees for lectures from AbbVie and Astra-Zeneca, all outside of the submitted work.

MPC reports grants from McGill Interdisciplinary Initiative in Infection and Immunity, grants from Canadian Institutes of Health Research, during the conduct of the study; personal fees from GEn1E Lifesciences, personal fees from nplex biosciences, personal fees from Kanvas biosciences, personal fees from AstraZeneca, non-financial support from Cidara therapeutics, non-financial support from Scynexis, Inc., non-financial support from Amplyx Pharmaceutics, outside the submitted work. In addition, MPC has a patent for methods detecting tissue damage, graft versus host disease, and infections using cell-free DNA profiling pending, a patent for methods assessing the severity and progression of SARS-CoV-2 infections using cell-free DNA pending, a patent for rapid identification of antimicrobial resistance and other microbial phenotypes using highly-multiplexed fluorescence in situ hybridization pending, and a patent highly multiplexed detection of gene expression with hybridization chain reaction pending, all outside the submitted work.

Ethics approval: Not applicable. This study did not involve human participants or animals.

Dating sharing: Data included in the analysis is available in Table S2 or from https://serotracker.com.

For peer review only

REFERENCES

1. Magnusson K, Nygard KM, Vold L, Telle KE. Occupational risk of COVID-19 in the 1st vs 2nd wave of infection. medRxiv. 2020 Jan 1.

2. Mutambudzi M, Niedwiedz C, Macdonald EB, Leyland A, Mair F, Anderson J, Celis-Morales C, Cleland J, Forbes J, Gill J, Hastie C. Occupation and risk of severe COVID-19: prospective cohort study of 120 075 UK Biobank participants. Occupational and Environmental Medicine. 2021 May 1;78(5):307-14.

3. Nguyen LH, Drew DA, Graham MS, Joshi AD, Guo CG, Ma W, Mehta RS, Warner ET, Sikavi DR, Lo CH, Kwon S. Risk of COVID-19 among front-line health-care workers and the general community: a prospective cohort study. The Lancet Public Health. 2020 Sep 1;5(9):e475-83.

4. Duarte N, D'Mello S, Duarte NA, Rocco S, Van Wyk J, Pillai AA, Liu M, Williamson T, Arora RK. Uptake of SARS-CoV-2 workplace testing programs, March 2020 to March 2021. medRxiv. 2021 Jan 1.

5. Duarte N, Yanes-Lane M, Arora RK, Bobrovitz N, Liu M, Bego MG, Yan T, Cao C, Gurry C, Hankins CA, Cheng MP. Adapting Serosurveys for the SARS-CoV-2 Vaccine Era. Open Forum Infect Dis. 2021 Dec 23;9(2):ofab632.

6. Bobrovitz N, Arora RK, Cao C, Boucher E, Liu M, Donnici C, Yanes-Lane M, Whelan M, Perlman-Arrow S, Chen J, Rahim H. Global seroprevalence of SARS-CoV-2 antibodies: a systematic review and meta-analysis. PloS one. 2021 Jun 23;16(6):e0252617.

7. Isho B, Abe KT, Zuo M, Jamal AJ, Rathod B, Wang JH, et al. Persistence of serum and saliva antibody responses to SARS-CoV-2 spike antigens in COVID-19 patients. Sci Immunol. 2020 Oct 8;5(52):eabe5511.

8. Munn Z, Moola S, Lisy K, Riitano D, Tufanaru C. Methodological guidance for systematic reviews of observational epidemiological studies reporting prevalence and incidence data. Int J Evid Based Healthc. 2015;13(3):147–153.

9. NIOSH (2021). NIOSH Industry and Occupation Computerized Coding System (NIOCCS). U.S. Department of Health and Human Services, Public Health Service, Centers for Disease Control and Prevention, National Institute for Occupational Safety

For peer review only - http://bmjopen.bmj.com/site/about/guidelines.xhtml

and Health, Division of Field Studies & Engineering, Health Informatics Branch. https://csams.cdc.gov/nioccs/About.aspx. Date accessed Sept 1, 2021.

10. Office for National Statistics. Coronavirus (COVID-19) Infection Survey: characteristics of people testing positive for COVID-19 in England. 2021 Feb 22. Available from: https://www.ons.gov.uk/peoplepopulationandcommunity/healthandsocialcare/conditionsanddiseases/articles/coronaviruscovid19infectionsinthecommunityinengland/characteristicsofpeopletestingpositiveforcovid19inengland22february2021

11. Baker MG, Peckham TK, Seixas NS. Estimating the burden of United States workers exposed to infection or disease: a key factor in containing risk of COVID-19 infection. PloS one. 2020 Apr 28;15(4):e0232452.

For peer review only

Figure Legends

Figure 1. Seroprevalence by SOC 2010 major occupation group. *Estimates are a mix of 'Healthcare Practitioners and Technical Occupations' and 'Healthcare Support Occupations' (see next page)

Table S1. Modified Joanna Briggs Institute Risk of Bias Tool (supplementary files)

Table S2. Summary of included studies (supplementary files)

For peer review only

SOC 2010 Major Occupation Group	Total Estimates	Total Countries	Median, IQR Study dates, midpoint	Median, IQR Sample size	Seroprevalence % (Median, IQR)	Seroprevalence % (Scale 0-75%)	N, % Low-Moderate RoB
Architecture and Engineering Occupations (17-0000)	1	1	15/08 (15/08-15/08)	21 (21-21)	42.9 (42.9-42.9)		0 (0%)
Personal Care and Service Occupations (39-0000)	14	7	03/05 (02/04-02/06)	127 (54-302)	21.5 (9.32-27.76)		3 (21%)
Installation, Maintenance, and Repair Occupations (49-0000)	1	1	19/06 (19/06-19/06)	134 (134-134)	16.4 (16.4-16.4)		0 (0%)
Building and Grounds Cleaning and Maintenance Occupations (37-0000)	17	8	13/07 (09/06-16/08)	102 (42-226)	10.8 (3.3-21.7)		6 (35%)
Healthcare Support Occupations (31-0000)	39	12	05/06 (19/05-21/06)	263 (122-562)	10.7 (2-20.05)		12 (31%)
Business and Financial Operations Occupations (13-0000)	2	2	05/07 (18/06-22/07)	462 (252-671)	8.27 (5.3-11.23)		2 (100%)
Management Occupations (11-0000)	10	6	17/06 (01/05-02/08)	44 (23-145)	8.17 (6.7-19.93)		3 (30%)
Food Preparation and Serving Related Occupations (35-0000)	6	4	17/06 (11/05-23/07)	58 (12-108)	6.35 (2.37-24.03)		2 (33%)
Healthcare Practitioners and Technical Occupations (29-0000)	222	23	13/06 (13/05-13/07)	215 (64-482)	5.91 (1.83-11.71)		84 (38%)
*Healthcare Practitioners and Technical Occupations, 5-digit codes***							
Miscellaneous Health Technologists and Technicians	4	3	26/08 (09/08-12/09)	60 (20-121)	12.96 (9.09-27.54)		1 (25%)
Registered Nurses	78	18	05/06 (05/05-05/07)	329 (71-1000)	8.44 (3.68-15.5)		22 (28%)
Clinical Laboratory Technologists and Technicians	18	12	15/06 (19/05-11/07)	204 (86-284)	6.22 (2.07-11.94)		12 (67%)
Physicians and Surgeons	65	21	09/06 (10/05-09/07)	214 (59-564)	5.88 (1.85-11.8)		23 (35%)
Emergency Medical Technicians and Paramedics	9	6	13/06 (27/05-30/06)	157 (56-243)	5.41 (5.2-11)		4 (44%)
Therapists	15	4	08/06 (19/05-28/06)	121 (61-235)	3.75 (0-9.45)		7 (47%)
Physician Assistants	9	2	27/06 (26/05-28/07)	230 (156-320)	3.48 (0.64-9.43)		3 (33%)
Pharmacists	9	7	29/06 (14/06-14/07)	113 (29-213)	0.5 (0-3.45)		4 (44%)
Healthcare Occupations (mixed)*	94	25	05/06 (29/04-12/07)	375 (110-1012)	5.66 (2.35-11.6)		23 (24%)
Sales and Related Occupations (41-0000)	23	8	21/08 (22/06-19/10)	643 (236-1184)	5.3 (1.2-8.8)		6 (26%)
Education, Training, and Library Occupations (25-0000)	6	5	05/07 (12/06-27/07)	238 (73-1305)	5.07 (2.71-17.22)		3 (50%)
Farming, Fishing, and Forestry Occupations (45-0000)	3	3	13/07 (25/06-30/07)	80 (66-100)	5 (2.5-5)		1 (33%)
Not employed (mixed)*	37	14	23/06 (12/05-04/08)	382 (116-905)	4.9 (2.7-14.97)		28 (76%)
Office and Administrative Support Occupations (43-0000)	39	18	14/06 (18/05-11/07)	120 (32-522)	4.88 (1.36-13.36)		20 (51%)
First responders (mixed)*	6	1	18/05 (13/05-22/05)	219 (72-599)	4.67 (1.6-7.34)		1 (17%)
Community and Social Service Occupations (21-0000)	6	2	30/05 (18/05-11/06)	104 (49-188)	4.45 (2.13-6.1)		1 (17%)
Protective Service Occupations (33-0000)	28	9	04/07 (21/05-16/08)	190 (46-555)	4.29 (2.17-7.47)		6 (21%)
Transportation and Material Moving Occupations (53-0000)	23	7	08/08 (08/06-08/10)	230 (80-364)	3.5 (1.8-11.8)		8 (35%)
Life, Physical, and Social Science Occupations (19-0000)	11	7	06/07 (11/06-30/07)	343 (174-570)	2.6 (1.66-6.46)		4 (36%)
Production Occupations (51-0000)	4	3	23/05 (26/04-19/06)	764 (342-1132)	1.52 (1.45-4.93)		2 (50%)
Arts, Design, Entertainment, Sports, and Media Occupations (27-0000)	6	5	07/07 (04/06-09/08)	164 (47-823)	1.39 (0.18-11.02)		3 (50%)
Computer and Mathematical Occupations (15-0000)	1	1	03/05 (03/05-03/05)	47 (47-47)	0 (0-0)		1 (100%)
Construction and Extraction Occupations (47-0000)	1	1	03/05 (03/05-03/05)	42 (42-42)	0 (0-0)		1 (100%)

Supplementary File I. List of all estimates, included studies and references

SOC 2010 Major Group	Study	N	SOC 2010 Occupation Title	Study Type	Study Dates	Country	Serum positive prevalence (95% CIs)	Overall Risk of Bias (JBI)
Not employed (mixed)*	Merkely et al., 2020[1]	n=209	Homemaker (Unpaid)	Cross-sectional survey	05/01 - 05/16	Hungary	0.73% (0-1.74%)	Moderate
Not employed (mixed)*	Siddiqui et al., 2020[2]	n=37	Homemaker (Unpaid)	Prospective cohort	04/15 - 08/15	India	18.9%	High
Not employed (mixed)*	Biggs et al., 2020[3]	n=157	Retired (Unpaid)	Cross-sectional survey	04/28 - 05/03	United States of America	1.91%	Moderate
Not employed (mixed)*	Carrat et al., 2020[4]	n=5381	Retired (Unpaid)	Prospective cohort	05/04 - 06/23	France	4.3% (3.5-5%)	Moderate
Not employed (mixed)*	Merkely et al., 2020[1]	n=2767	Retired (Unpaid)	Cross-sectional survey	05/01 - 05/16	Hungary	1.09% (0.66-1.52%)	Moderate
Not employed (mixed)*	Richard et al., 2020[5]	n=1635	Retired (Unpaid)	Cross-sectional survey	04/06 - 06/30	Switzerland	4.3%	Low
Not employed (mixed)*	Siddiqui et al., 2020[2]	n=10	Retired (Unpaid)	Prospective cohort	04/15 - 08/15	India	20%	High
Not employed (mixed)*	Alemu et al., 2020[6]	n=32	Student (Unpaid)	Cross-sectional survey	04/23 - 04/28	Ethiopia	15.6%	Moderate
Not employed (mixed)*	Biggs et al., 2020[3]	n=16	Student (Unpaid)	Cross-sectional survey	04/28 - 05/03	United States of America	12.5%	Moderate
Not employed (mixed)*	Brehm et al., 2020[7]	n=73	Student (Unpaid)	Cross sectional study with prospective cohort follow up of a subset of the sample	03/20 - 07/17	Germany	2.7%	Moderate
Not employed (mixed)*	Carrat et al., 2020[4]	n=81	Student (Unpaid)	Prospective cohort	05/04 - 06/23	France	7.2% (0.1-12.6%)	Moderate

For peer review only

Not employed (mixed)*	Iversen et al., 2020[8]	n=688	Student (Unpaid)	Cross-sectional survey	04/15 - 04/22	Denmark	14.97%	Low
Not employed (mixed)*	Lumley et al., 2020[9]	n=620	Student (Unpaid)	Prospective cohort	04/23 - 11/30	The United Kingdom	6.77%	Moderate
Not employed (mixed)*	Merkely et al., 2020[1]	n=774	Student (Unpaid)	Cross-sectional survey	05/01 - 05/16	Hungary	0.69% (0-1.49%)	Moderate
Not employed (mixed)*	Richard et al., 2020[5]	n=666	Student (Unpaid)	Cross-sectional survey	04/06 - 06/30	Switzerland	10.5%	Low
Not employed (mixed)*	Shakiba et al., 2020[10]	n=114	Student (Unpaid)	Cross-sectional survey	04/11 - 04/19	Iran (Islamic Republic of)	17.5% (11.3-23.7%)	Moderate
Not employed (mixed)*	Siddiqui et al., 2020[2]	n=14	Student (Unpaid)	Prospective cohort	04/15 - 08/15	India	21.4%	High
Not employed (mixed)*	Tilley et al., 2020[11]	n=790	Student (Unpaid)	Cross-sectional survey	04/29 - 05/08	United States of America	4% (3-5.1%)	Moderate
Not employed (mixed)*	Tsitsilonis et al., 2020[12]	n=1395	Student (Unpaid)	Cross-sectional survey	06/15 - 07/15	Greece	0.42% (0.03-1.5%)	Moderate
Not employed (mixed)*	Arnaldo et al., 2020[13]	n=513	Military, Rank Not Specified	Cross-sectional survey	07/06 - 07/13	Mozambique	3.7%	High
Not employed (mixed)*	Arnaldo et al., 2020[14]	n=116	Military, Rank Not Specified	Cross-sectional survey	11/02 - 11/12	Mozambique	1.7%	High
Not employed (mixed)*	Mabunda et al., 2020[15]	n=324	Military, Rank Not Specified	Cross-sectional survey	09/21 - 10/02	Mozambique	2.8%	High
Not employed (mixed)*	Mahomed et al., 2020[16]	n=116	Military, Rank Not Specified	Cross-sectional survey	11/26 - 12/03	Mozambique	18.1%	High
Not employed (mixed)*	Payne et al., 2020[17]	n=382	Military, Rank Not Specified	Cross-sectional survey	04/20 - 04/24	United States of America	59.7%	High
Not employed (mixed)*	World et al., 2020[18]	n=6900	Military, Rank Not Specified	Cross-sectional survey	08/15 - 10/15	Republic of Korea	0.36%	Unclear
Management Occupations (11-0000)	Shakiba et al., 2020[10]	n=16	Farmers, Ranchers, and Other Agricultural Managers	Cross-sectional survey	04/11 - 04/19	Iran (Islamic Republic of)	19.7% (9.1-31%)	Moderate
Management Occupations (11-	Favara et al., 2020[19]	n=43	Medical and Health Services Managers	Cross-sectional survey	07/13 - 07/13	The United Kingdom	9.3%	High

For peer review only

0000)								
Management Occupations (11-0000)	Galan et al., 2020[20]	n=170	Medical and Health Services Managers	Cross-sectional survey	04/14 - 04/27	Spain	27.6%	High
Management Occupations (11-0000)	Hunter et al., 2020[21]	n=44	Medical and Health Services Managers	Cross-sectional survey	04/29 - 05/08	United States of America	4.55%	High
Management Occupations (11-0000)	Leidner et al., 2020[22]	n=257	Medical and Health Services Managers	Cross sectional study with prospective cohort follow up of a subset of the sample	04/08 - 05/22	United States of America	3.11%	High
Management Occupations (11-0000)	Martin et al., 2020[23]	n=2078	Medical and Health Services Managers	Cross-sectional survey	05/29 - 07/13	The United Kingdom	6.79%	Moderate
Management Occupations (11-0000)	Siddiqui et al., 2020[2]	n=15	Medical and Health Services Managers	Prospective cohort	04/15 - 08/15	India	20%	High
Management Occupations (11-0000)	Baracco et al., 2020[24]	n=45	Managers, All Other	Cross-sectional survey	04/23 - 05/05	Italy	6.67%	High
Management Occupations (11-0000)	Goenka et al., 2020[25]	n=71	Managers, All Other	Cross-sectional survey	07/12 - 08/23	India	7.04%	Moderate
Management Occupations (11-0000)	Goenka et al., 2020[26]	n=13	Managers, All Other	Cross-sectional survey	08/01 - 08/31	India	38.46%	High
Business and Financial Operations Occupations (13-0000)	Satpati et al., 2020[27]	n=43	Management Analysts	Cross-sectional survey	07/26 - 08/08	India	2.33%	Moderate
Business and Financial	Poustchi et al., 2020[28]	n=880	Financial Specialists	Cross-sectional survey	04/17 - 06/02	Iran (Islamic Republic of)	14.2% (12.1-16.5%)	Moderate

For peer review only

Operations Occupations (13-0000)								
Computer and Mathematical Occupations (15-0000)	Biggs et al., 2020[3]	n=47	Computer User Support Specialists	Cross-sectional survey	04/28 - 05/03	United States of America	0%	Moderate
Architecture and Engineering Occupations (17-0000)	Siddiqui et al., 2020[2]	n=21	Engineers	Prospective cohort	04/15 - 08/15	India	42.9%	High
Life, Physical, and Social Science Occupations (19-0000)	Jones et al., 2020[29]	n=245	Medical Scientists	Cross-sectional survey	01/15 - 06/15	The United Kingdom	1.9%	High
Life, Physical, and Social Science Occupations (19-0000)	Anna et al., 2020[30]	n=505	Medical Scientists, Except Epidemiologists	Prospective cohort	04/28 - 07/31	France	8.71%	Moderate
Life, Physical, and Social Science Occupations (19-0000)	Erber et al., 2020[31]	n=635	Medical Scientists, Except Epidemiologists	Cross-sectional survey	04/14 - 05/29	Germany	1.24%	High
Life, Physical, and Social Science Occupations (19-0000)	Favara et al., 2020[19]	n=38	Medical Scientists, Except Epidemiologists	Cross-sectional survey	07/13 - 07/13	The United Kingdom	2.6%	High
Life, Physical, and Social Science Occupations (19-0000)	Hanrath et al., 2020[32]	n=468	Medical Scientists, Except Epidemiologists	Cross-sectional survey	05/29 - 07/06	The United Kingdom	6.2%	High
Life, Physical, and Social Science Occupations (19-0000)	Leidner et al., 2020[22]	n=2654	Medical Scientists, Except Epidemiologists	Cross sectional study with prospective cohort follow up of a subset of the sample	04/08 - 05/22	United States of America	2.22%	High

For peer review only

Life, Physical, and Social Science Occupations (19-0000)	Martin et al., 2020[23]	n=1154	Medical Scientists, Except Epidemiologists	Cross-sectional survey	05/29 - 07/13	The United Kingdom	9.71%	Moderate
Life, Physical, and Social Science Occupations (19-0000)	Rosser et al., 2020[33]	n=102	Medical Scientists, Except Epidemiologists	Cross-sectional survey	04/20 - 05/20	United States of America	0.98%	High
Life, Physical, and Social Science Occupations (19-0000)	Silva et al., 2020[34]	n=69	Chemists	Cross-sectional survey	06/05 - 07/31	Brazil	4%	High
Life, Physical, and Social Science Occupations (19-0000)	Tsitsilonis et al., 2020[12]	n=250	Physical Scientists, All Other	Cross-sectional survey	06/15 - 07/15	Greece	1.42% (0-7.24%)	Moderate
Community and Social Service Occupations (21-0000)	Jones et al., 2020[29]	n=211	Healthcare Social Workers	Cross-sectional survey	01/15 - 06/15	The United Kingdom	6.3%	High
Community and Social Service Occupations (21-0000)	Leidner et al., 2020[22]	n=235	Social Workers, All Other	Cross sectional study with prospective cohort follow up of a subset of the sample	04/08 - 05/22	United States of America	3.4%	High
Community and Social Service Occupations (21-0000)	Rosser et al., 2020[33]	n=117	Social Workers, All Other	Cross-sectional survey	04/20 - 05/20	United States of America	1.71%	High
Community and Social Service Occupations (21-0000)	Sabourin et al., 2020[35]	n=91	Social Workers, All Other	Cross-sectional survey	07/15 - 08/15	United States of America	5.49%	High
Community and Social Service	Yogo et al., 2020[36]	n=35	Social Workers, All Other	Cross-sectional survey	05/20 - 06/08	United States of America	0%	High

Occupations (21-0000)								
Community and Social Service Occupations (21-0000)	Biggs et al., 2020[3]	n=6	Religious Workers	Cross-sectional survey	04/28 - 05/03	United States of America	16.67%	Moderate
Education, Training, and Library Occupations (25-0000)	Campos et al., 2020[37]	n=2715	Postsecondary Teachers	Cross-sectional survey	05/13 - 07/10	Portugal	2.6%	High
Education, Training, and Library Occupations (25-0000)	Goncalves et al., 2020[38]	n=1636	Postsecondary Teachers	Cross-sectional survey	06/15 - 06/30	Portugal	3.05%	Moderate
Education, Training, and Library Occupations (25-0000)	Tsitsilonis et al., 2020[12]	n=312	Postsecondary Teachers	Cross-sectional survey	06/15 - 07/15	Greece	1.2% (0.14-3.7%)	Moderate
Education, Training, and Library Occupations (25-0000)	Fontanet et al., 2020[39]	n=42	Elementary and Middle School Teachers	Retrospective cohort	04/28 - 04/30	France	7.1%	Moderate
Education, Training, and Library Occupations (25-0000)	Siddiqui et al., 2020[2]	n=8	Elementary and Middle School Teachers	Prospective cohort	04/15 - 08/15	India	25%	High
Education, Training, and Library Occupations (25-0000)	Torres et al., 2020[40]	n=165	Elementary and Middle School Teachers	Cross-sectional survey	05/04 - 05/19	Chile	20.6% (14.7-27.6%)	High

Arts, Design, Entertainment, Sports, and Media Occupations (27-0000)	Halatoko et al., 2020[41]	n=55	Fine Artists, Including Painters, Sculptors, and Illustrators	Cross-sectional survey	04/23 - 05/08	Togo	0%	High
Arts, Design, Entertainment, Sports, and Media Occupations (27-0000)	Slusser et al., 2020[42]	n=5603	Athletes, Coaches, Umpires, and Related Workers	Cross-sectional survey	04/08 - 04/21	United States of America	0.7% (0.28-1.15%)	Unclear
Arts, Design, Entertainment, Sports, and Media Occupations (27-0000)	Vince et al., 2020[43]	n=272	Athletes, Coaches, Umpires, and Related Workers	Prospective cohort	05/29 - 07/31	Croatia	14%	Moderate
Arts, Design, Entertainment, Sports, and Media Occupations (27-0000)	Vince et al., 2020[43]	n=43	Coaches and Scouts	Prospective cohort	05/29 - 07/31	Croatia	16.3%	Moderate
Arts, Design, Entertainment, Sports, and Media Occupations (27-0000)	Mack et al., 2020[44]	n=1007	Umpires, Referees, and Other Sports Officials	Prospective cohort	06/16 - 06/30	Germany	2.09% (1.37-3.17%)	High
Arts, Design, Entertainment, Sports, and Media Occupations (27-0000)	Khan et al., 2020[45]	n=44	Media and Communication Workers	Cross-sectional survey	07/01 - 07/15	India	0%	Moderate
Healthcare Practitioners and Technical Occupations (29-0000)	Akinbami et al., 2020[46]	n=566	Healthcare Practitioners and Technical Occupations	Cross-sectional survey	05/18 - 06/13	United States of America	4.6% (3-6.7%)	Moderate

For peer review only

Healthcare Practitioners and Technical Occupations (29-0000)	Khan et al., 2020[45]	n=355	Healthcare Practitioners and Technical Occupations	Cross-sectional survey	07/01 - 07/15	India	4.8% (3-7.6%)	Moderate
Healthcare Practitioners and Technical Occupations (29-0000)	Leidner et al., 2020[22]	n=402	Healthcare Practitioners and Technical Occupations	Cross sectional study with prospective cohort follow up of a subset of the sample	04/08 - 05/22	United States of America	1.49%	High
Healthcare Occupations (mixed)*	Hanrath et al., 2020[32]	n=102	Healthcare Practitioners and Technical Occupations and Healthcare Support Occupations*	Cross-sectional survey	05/29 - 07/06	The United Kingdom	6.62%	High
Healthcare Occupations (mixed)*	Jones et al., 2020[29]	n=413	Healthcare Practitioners and Technical Occupations and Healthcare Support Occupations*	Cross-sectional survey	01/15 - 06/15	The United Kingdom	7.8%	High
Healthcare Occupations (mixed)*	Martin et al., 2020[23]	n=550	Healthcare Practitioners and Technical Occupations and Healthcare Support Occupations*	Cross-sectional survey	05/29 - 07/13	The United Kingdom	10.36%	Moderate
Healthcare Occupations (mixed)*	Amendola et al., 2020[47]	n=117	Healthcare Practitioners and Technical Occupations and Healthcare Support Occupations*	Cross-sectional survey	04/15 - 04/15	Italy	4.27%	High
Healthcare Occupations (mixed)*	Arnaldo et al., 2020[48]	n=543	Healthcare Practitioners and Technical Occupations and Healthcare Support Occupations*	Cross-sectional survey	08/10 - 08/21	Mozambique	3.7%	High

For peer review only

Healthcare Occupations (mixed)*	Bal et al., 2020[49]	n=190	Healthcare Practitioners and Technical Occupations and Healthcare Support Occupations*	Cross-sectional survey	04/10 - 05/28	France	3.68%	High
Healthcare Occupations (mixed)*	Barallat et al., 2020[50]	n=429	Healthcare Practitioners and Technical Occupations and Healthcare Support Occupations*	Cross-sectional survey	05/04 - 05/22	Spain	7.69%	High
Healthcare Occupations (mixed)*	Bardai et al., 2020[51]	n=35	Healthcare Practitioners and Technical Occupations and Healthcare Support Occupations*	Cross-sectional survey	06/10 - 07/27	Canada	11%	High
Healthcare Occupations (mixed)*	Bardai et al., 2020[51]	n=20	Healthcare Practitioners and Technical Occupations and Healthcare Support Occupations*	Cross-sectional survey	06/10 - 07/27	Canada	15%	High
Healthcare Occupations (mixed)*	Bardai et al., 2020[51]	n=44	Healthcare Practitioners and Technical Occupations and Healthcare Support Occupations*	Cross-sectional survey	06/10 - 07/27	Canada	11%	High
Healthcare Occupations (mixed)*	Bardai et al., 2020[51]	n=99	Healthcare Practitioners and Technical Occupations and Healthcare Support Occupations*	Cross-sectional survey	06/10 - 07/27	Canada	12%	High
Healthcare Occupations (mixed)*	Biggs et al., 2020[3]	n=59	Healthcare Practitioners and Technical Occupations and Healthcare Support Occupations*	Cross-sectional survey	04/28 - 05/03	United States of America	10.17%	Moderate

For peer review only

Healthcare Occupations (mixed)*	Blairon et al., 2020[52]	n=588	Healthcare Practitioners and Technical Occupations and Healthcare Support Occupations*	Cross-sectional survey	05/25 - 06/19	Belgium	19.2%	High
Healthcare Occupations (mixed)*	Borraz et al., 2020[53]	n=289	Healthcare Practitioners and Technical Occupations and Healthcare Support Occupations*	Prospective cohort	03/20 - 04/21	Spain	5.88%	High
Healthcare Occupations (mixed)*	Brunner et al., 2020[54]	n=762	Healthcare Practitioners and Technical Occupations and Healthcare Support Occupations*	Cross-sectional survey	05/04 - 05/29	United States of America	4.5%	High
Healthcare Occupations (mixed)*	Brunner et al., 2020[54]	n=764	Healthcare Practitioners and Technical Occupations and Healthcare Support Occupations*	Cross-sectional survey	05/04 - 05/29	United States of America	2%	High
Healthcare Occupations (mixed)*	Carozzi et al., 2020[55]	n=17098	Healthcare Practitioners and Technical Occupations and Healthcare Support Occupations*	Cross-sectional survey	04/01 - 04/30	Italy	3.1%	High
Healthcare Occupations (mixed)*	Carrat et al., 2020[4]	n=568	Healthcare Practitioners and Technical Occupations and Healthcare Support Occupations*	Prospective cohort	05/04 - 06/23	France	11.6% (8.3-14.4%)	Moderate
Healthcare Occupations (mixed)*	Cavlek et al., 2020[56]	n=558	Healthcare Practitioners and Technical Occupations and Healthcare Support Occupations*	Cross-sectional survey	04/25 - 05/24	Croatia	1.25%	High

For peer review only

Healthcare Occupations (mixed)*	Chibwana et al., 2020[57]	n=500	Healthcare Practitioners and Technical Occupations and Healthcare Support Occupations*	Prospective cohort	05/22 - 06/19	Malawi	12.3% (8.2-16.5%)	High
Healthcare Occupations (mixed)*	Coffman et al., 2020[58]	n=1100	Healthcare Practitioners and Technical Occupations and Healthcare Support Occupations*	Cross-sectional survey	07/01 - 07/31	United States of America	2.2%	Unclear
Healthcare Occupations (mixed)*	Cooper et al., 2020[59]	n=118	Healthcare Practitioners and Technical Occupations and Healthcare Support Occupations*	Cross-sectional survey	06/10 - 08/07	The United Kingdom	8.47%	Moderate
Healthcare Occupations (mixed)*	Cooper et al., 2020[59]	n=27	Healthcare Practitioners and Technical Occupations and Healthcare Support Occupations*	Cross-sectional survey	06/10 - 08/07	The United Kingdom	14.81%	Moderate
Healthcare Occupations (mixed)*	Cooper et al., 2020[59]	n=24	Healthcare Practitioners and Technical Occupations and Healthcare Support Occupations*	Cross-sectional survey	06/10 - 08/07	The United Kingdom	12.5%	Moderate
Healthcare Occupations (mixed)*	Cooper et al., 2020[59]	n=1068	Healthcare Practitioners and Technical Occupations and Healthcare Support Occupations*	Cross-sectional survey	06/10 - 08/07	The United Kingdom	5.43%	Moderate
Healthcare Occupations (mixed)*	Cooper et al., 2020[59]	n=174	Healthcare Practitioners and Technical Occupations and Healthcare Support Occupations*	Cross-sectional survey	06/10 - 08/07	The United Kingdom	5.75%	Moderate

For peer review only

Healthcare Occupations (mixed)*	Cooper et al., 2020[59]	n=319	Healthcare Practitioners and Technical Occupations and Healthcare Support Occupations*	Cross-sectional survey	06/10 - 08/07	The United Kingdom	11.29%	Moderate
Healthcare Occupations (mixed)*	Cooper et al., 2020[59]	n=5698	Healthcare Practitioners and Technical Occupations and Healthcare Support Occupations*	Cross-sectional survey	06/10 - 08/07	The United Kingdom	7.2%	Moderate
Healthcare Occupations (mixed)*	Cooper et al., 2020[59]	n=412	Healthcare Practitioners and Technical Occupations and Healthcare Support Occupations*	Cross-sectional survey	06/10 - 08/07	The United Kingdom	4.61%	Moderate
Healthcare Occupations (mixed)*	Denyer et al., 2020[60]	n=5850	Healthcare Practitioners and Technical Occupations and Healthcare Support Occupations*	Cross-sectional survey	05/12 - 05/18	Japan	1.79%	Unclear
Healthcare Occupations (mixed)*	Dimeglio et al., 2020[61]	n=8758	Healthcare Practitioners and Technical Occupations and Healthcare Support Occupations*	Cross-sectional survey	06/10 - 07/10	France	3.2% (2.8-3.5%)	High
Healthcare Occupations (mixed)*	Erber et al., 2020[31]	n=603	Healthcare Practitioners and Technical Occupations and Healthcare Support Occupations*	Cross-sectional survey	04/14 - 05/29	Germany	2.8%	High
Healthcare Occupations (mixed)*	Fuereder et al., 2020[62]	n=62	Healthcare Practitioners and Technical Occupations and Healthcare Support Occupations*	Retrospective cohort	04/01 - 06/04	Austria	3.2% (0.4-11.2%)	High

For peer review only

Healthcare Occupations (mixed)*	Fusco et al., 2020[63]	n=115	Healthcare Practitioners and Technical Occupations and Healthcare Support Occupations*	Cross-sectional survey	03/23 - 04/02	Italy	1.74%	High
Healthcare Occupations (mixed)*	Geraci et al., 2020[64]	n=230	Healthcare Practitioners and Technical Occupations and Healthcare Support Occupations*	Cross-sectional survey	03/16 - 05/20	United States of America	2.17%	High
Healthcare Occupations (mixed)*	Gudo et al., 2020[65]	n=1427	Healthcare Practitioners and Technical Occupations and Healthcare Support Occupations*	Cross-sectional survey	06/17 - 06/30	Mozambique	7% (6-9%)	High
Healthcare Occupations (mixed)*	Hackner et al., 2020[66]	n=130	Healthcare Practitioners and Technical Occupations and Healthcare Support Occupations*	Cross-sectional survey	04/01 - 04/30	Austria	2.3%	High
Healthcare Occupations (mixed)*	Halatoko et al., 2020[41]	n=370	Healthcare Practitioners and Technical Occupations and Healthcare Support Occupations*	Cross-sectional survey	04/23 - 05/08	Togo	1.4%	High
Healthcare Occupations (mixed)*	Haq et al., 2020[67]	n=76	Healthcare Practitioners and Technical Occupations and Healthcare Support Occupations*	Cross-sectional survey	06/15 - 06/29	Pakistan	35.5% (24.8-47.3%)	Moderate
Healthcare Occupations (mixed)*	He et al., 2020[68]	n=1059	Healthcare Practitioners and Technical Occupations and Healthcare Support Occupations*	Repeated cross sectional study	05/13 - 06/10	China	9.3%	High

For peer review only

Healthcare Occupations (mixed)*	Herzberg et al., 2020[69]	n=871	Healthcare Practitioners and Technical Occupations and Healthcare Support Occupations*	Prospective cohort	04/14 - 06/16	Germany	2.64%	High
Healthcare Occupations (mixed)*	Jeremias et al., 2020[70]	n=100	Healthcare Practitioners and Technical Occupations and Healthcare Support Occupations*	Cross-sectional survey	03/01 - 04/30	United States of America	12%	High
Healthcare Occupations (mixed)*	Jespersen et al., 2020[71]	n=17948	Healthcare Practitioners and Technical Occupations and Healthcare Support Occupations*	Cross-sectional survey	05/18 - 06/19	Denmark	3.36% (2.38-3.82%)	Moderate
Healthcare Occupations (mixed)*	Kassem et al., 2020[72]	n=74	Healthcare Practitioners and Technical Occupations and Healthcare Support Occupations*	Cross-sectional survey	06/01 - 06/14	Egypt	12.2%	High
Healthcare Occupations (mixed)*	Kern et al., 2020[73]	n=1316	Healthcare Practitioners and Technical Occupations and Healthcare Support Occupations*	Cross-sectional survey	04/09 - 04/16	Germany	1.06% (0.58-1.78%)	High
Healthcare Occupations (mixed)*	Khalil et al., 2020[74]	n=190	Healthcare Practitioners and Technical Occupations and Healthcare Support Occupations*	Cross-sectional survey	05/15 - 05/28	The United Kingdom	22%	High
Healthcare Occupations (mixed)*	Kumar et al., 2020[75]	n=635	Healthcare Practitioners and Technical Occupations and Healthcare Support Occupations*	Repeated cross sectional study	07/11 - 07/24	India	0%	High

For peer review only

Healthcare Occupations (mixed)*	Lackermair et al., 2020[76]	n=151	Healthcare Practitioners and Technical Occupations and Healthcare Support Occupations*	Cross-sectional survey	04/02 - 04/06	Germany	2.6% (0.8-7.1%)	High
Healthcare Occupations (mixed)*	Lahner et al., 2020[77]	n=1084	Healthcare Practitioners and Technical Occupations and Healthcare Support Occupations*	Cross-sectional survey	04/07 - 04/27	Italy	0.7%	High
Healthcare Occupations (mixed)*	Liu et al., 2020[78]	n=116	Healthcare Practitioners and Technical Occupations and Healthcare Support Occupations*	Cross-sectional survey	02/07 - 04/21	China	0%	High
Healthcare Occupations (mixed)*	Liu et al., 2020[78]	n=304	Healthcare Practitioners and Technical Occupations and Healthcare Support Occupations*	Cross-sectional survey	02/07 - 04/21	China	0%	High
Healthcare Occupations (mixed)*	Liu et al., 2020[79]	n=3832	Healthcare Practitioners and Technical Occupations and Healthcare Support Occupations*	Cross-sectional survey	02/29 - 04/29	China	4% (3.4-4.7%)	Moderate
Healthcare Occupations (mixed)*	Lorenzo et al., 2020[80]	n=38	Healthcare Practitioners and Technical Occupations and Healthcare Support Occupations*	Cross-sectional survey	05/02 - 05/31	Italy	5.3%	High
Healthcare Occupations (mixed)*	Mahomed et al., 2020[81]	n=569	Healthcare Practitioners and Technical Occupations and Healthcare Support Occupations*	Cross-sectional survey	08/31 - 10/12	Mozambique	0.7%	High

For peer review only

Healthcare Occupations (mixed)*	Mahumane et al., 2020[82]	n=380	Healthcare Practitioners and Technical Occupations and Healthcare Support Occupations*	Cross-sectional survey	11/02 - 11/17	Mozambique	1.3%	High
Healthcare Occupations (mixed)*	Majdoubi et al., 2020[83]	n=276	Healthcare Practitioners and Technical Occupations and Healthcare Support Occupations*	Cross-sectional survey	05/17 - 06/19	Canada	0.6% (0-2.71%)	High
Healthcare Occupations (mixed)*	Majiya et al., 2020[84]	n=185	Healthcare Practitioners and Technical Occupations and Healthcare Support Occupations*	Cross-sectional survey	06/26 - 06/30	Nigeria	25.41%	Moderate
Healthcare Occupations (mixed)*	Majiya et al., 2020[84]	n=43	Healthcare Practitioners and Technical Occupations and Healthcare Support Occupations*	Cross-sectional survey	06/26 - 06/30	Nigeria	37.21%	Moderate
Healthcare Occupations (mixed)*	Malfertheiner et al., 2020[85]	n=139	Healthcare Practitioners and Technical Occupations and Healthcare Support Occupations*	Prospective cohort	03/15 - 06/07	Germany	0%	High
Healthcare Occupations (mixed)*	Martin et al., 2020[86]	n=326	Healthcare Practitioners and Technical Occupations and Healthcare Support Occupations*	Cross-sectional survey	04/15 - 05/18	Belgium	11%	High
Healthcare Occupations (mixed)*	Martin et al., 2020[23]	n=4631	Healthcare Practitioners and Technical Occupations and Healthcare Support Occupations*	Cross-sectional survey	05/29 - 07/13	The United Kingdom	13.65%	Moderate

For peer review only

Healthcare Occupations (mixed)*	Melo et al., 2020[87]	n=471	Healthcare Practitioners and Technical Occupations and Healthcare Support Occupations*	Cross-sectional survey	06/01 - 06/30	Brazil	13.59%	High
Healthcare Occupations (mixed)*	Morcuende et al., 2020[88]	n=6	Healthcare Practitioners and Technical Occupations and Healthcare Support Occupations*	Cross-sectional survey	03/01 - 04/21	United States of America	0%	High
Healthcare Occupations (mixed)*	Moscola et al., 2020[89]	n=8156	Healthcare Practitioners and Technical Occupations and Healthcare Support Occupations*	Cross-sectional survey	04/20 - 06/23	United States of America	11.6%	High
Healthcare Occupations (mixed)*	Nishida et al., 2020[90]	n=49	Healthcare Practitioners and Technical Occupations and Healthcare Support Occupations*	Cross-sectional survey	06/12 - 06/19	Japan	0%	Moderate
Healthcare Occupations (mixed)*	Olalla et al., 2020[91]	n=498	Healthcare Practitioners and Technical Occupations and Healthcare Support Occupations*	Cross-sectional survey	04/15 - 04/25	Spain	2.2%	High
Healthcare Occupations (mixed)*	Pallett et al., 2020[92]	n=504	Healthcare Practitioners and Technical Occupations and Healthcare Support Occupations*	Prospective cohort	04/08 - 06/12	The United Kingdom	10.6% (7.6-13.6%)	High
Healthcare Occupations (mixed)*	Pere et al., 2020[93]	n=3569	Healthcare Practitioners and Technical Occupations and Healthcare Support Occupations*	Cross-sectional survey	05/02 - 06/26	France	11.9%	High

For peer review only

Healthcare Occupations (mixed)*	Poulikakos et al., 2020[94]	n=281	Healthcare Practitioners and Technical Occupations and Healthcare Support Occupations*	Cross-sectional survey	05/04 - 05/06	The United Kingdom	6%	High
Healthcare Occupations (mixed)*	Psichogiou et al., 2020[95]	n=1495	Healthcare Practitioners and Technical Occupations and Healthcare Support Occupations*	Cross-sectional survey	04/13 - 05/15	Greece	1.26% (0.43-3.26%)	Moderate
Healthcare Occupations (mixed)*	Satpati et al., 2020[27]	n=18	Healthcare Practitioners and Technical Occupations and Healthcare Support Occupations*	Cross-sectional survey	07/26 - 08/08	India	5.56%	Moderate
Healthcare Occupations (mixed)*	Seetharam et al., 2020[96]	n=728	Healthcare Practitioners and Technical Occupations and Healthcare Support Occupations*	Cross-sectional survey	08/16 - 08/29	India	27.3% (24.1-30.6%)	Unclear
Healthcare Occupations (mixed)*	Shakiba et al., 2020[10]	n=43	Healthcare Practitioners and Technical Occupations and Healthcare Support Occupations*	Cross-sectional survey	04/11 - 04/19	Iran (Islamic Republic of)	14.5% (4.5-25%)	Moderate
Healthcare Occupations (mixed)*	Shields et al., 2020[97]	n=516	Healthcare Practitioners and Technical Occupations and Healthcare Support Occupations*	Cross-sectional survey	04/24 - 04/25	The United Kingdom	24.4%	High
Healthcare Occupations (mixed)*	Silva et al., 2020[98]	n=61	Healthcare Practitioners and Technical Occupations and Healthcare Support Occupations*	Cross-sectional survey	04/09 - 04/29	Brazil	4.91%	High

For peer review only

Healthcare Occupations (mixed)*	Solodky et al., 2020[99]	n=85	Healthcare Practitioners and Technical Occupations and Healthcare Support Occupations*	Cross-sectional survey	03/01 - 04/16	France	5.88%	High
Healthcare Occupations (mixed)*	Soriano et al., 2020[100]	n=108	Healthcare Practitioners and Technical Occupations and Healthcare Support Occupations*	Retrospective cohort	04/26 - 05/16	Spain	13%	High
Healthcare Occupations (mixed)*	Statistica et al., 2020[101]	n=64660	Healthcare Practitioners and Technical Occupations and Healthcare Support Occupations*	Cross-sectional survey	05/25 - 07/15	Italy	2.5%	Unclear
Healthcare Occupations (mixed)*	Steensels et al., 2020[102]	n=3056	Healthcare Practitioners and Technical Occupations and Healthcare Support Occupations*	Cross-sectional survey	04/22 - 04/30	Belgium	6.4% (5.5-7.3%)	High
Healthcare Occupations (mixed)*	Stock et al., 2020[103]	n=98	Healthcare Practitioners and Technical Occupations and Healthcare Support Occupations*	Cross-sectional survey	04/04 - 04/20	United States of America	15.3%	High
Healthcare Occupations (mixed)*	Takita et al., 2020[104]	n=175	Healthcare Practitioners and Technical Occupations and Healthcare Support Occupations*	Cross-sectional survey	04/20 - 05/20	Japan	4% (1.62-8.07%)	High
Healthcare Occupations (mixed)*	Tong et al., 2020[105]	n=191	Healthcare Practitioners and Technical Occupations and Healthcare Support Occupations*	Cross-sectional survey	05/12 - 05/15	China	0%	High

For peer review only

Healthcare Occupations (mixed)*	Trieu et al., 2020[106]	n=607	Healthcare Practitioners and Technical Occupations and Healthcare Support Occupations*	Prospective cohort	03/06 - 04/09	Norway	5.27%	High
Healthcare Occupations (mixed)*	Tu et al., 2020[107]	n=325	Healthcare Practitioners and Technical Occupations and Healthcare Support Occupations*	Cross sectional study with prospective cohort follow up of a subset of the sample	03/19 - 03/20	China	43.08%	High
Healthcare Occupations (mixed)*	Valdivia et al., 2020[108]	n=1153	Healthcare Practitioners and Technical Occupations and Healthcare Support Occupations*	Cross-sectional survey	04/13 - 04/30	Spain	3.5%	High
Healthcare Occupations (mixed)*	Vasquez et al., 2020[109]	n=1147	Healthcare Practitioners and Technical Occupations and Healthcare Support Occupations*	Cross-sectional survey	05/19 - 06/06	Peru	58.3%	High
Healthcare Occupations (mixed)*	Viegas et al., 2020[110]	n=1443	Healthcare Practitioners and Technical Occupations and Healthcare Support Occupations*	Cross-sectional survey	08/03 - 08/21	Mozambique	2.63%	High
Healthcare Occupations (mixed)*	Vlachoyiannopoulosa et al., 2020[111]	n=321	Healthcare Practitioners and Technical Occupations and Healthcare Support Occupations*	Cross-sectional survey	04/25 - 05/10	Greece	2.18%	High
Healthcare Occupations (mixed)*	Volta et al., 2020[112]	n=76	Healthcare Practitioners and Technical Occupations and Healthcare Support Occupations*	Cross-sectional survey	04/27 - 04/27	Italy	11.8%	High

For peer review only

Healthcare Occupations (mixed)*	Ward et al., 2020[113]	n=5416	Healthcare Practitioners and Technical Occupations and Healthcare Support Occupations*	Cross-sectional survey	09/15 - 09/28	The United Kingdom	10.67%	Moderate
Healthcare Occupations (mixed)*	Ward et al., 2020[113]	n=1692	Healthcare Practitioners and Technical Occupations and Healthcare Support Occupations*	Cross-sectional survey	09/15 - 09/28	The United Kingdom	6.68%	Moderate
Healthcare Occupations (mixed)*	Xiong et al., 2020[114]	n=797	Healthcare Practitioners and Technical Occupations and Healthcare Support Occupations*	Cross-sectional survey	02/12 - 03/17	China	4.39%	Unclear
Healthcare Occupations (mixed)*	Zhang et al., 2020[115]	n=63	Healthcare Practitioners and Technical Occupations and Healthcare Support Occupations*	Cross-sectional survey	01/21 - 02/16	China	0%	High
Healthcare Occupations (mixed)*	Zhao et al., 2020[116]	n=1060	Healthcare Practitioners and Technical Occupations and Healthcare Support Occupations*	Cross-sectional survey	01/14 - 02/21	China	8.3%	High
First responders (mixed)*	Ahmad et al., 2020[117]	n=40	Healthcare Practitioners and Technical Occupations and Protective Service Occupations (i.e. first responders)*	Cross-sectional survey	04/21 - 05/22	United States of America	20%	High
First responders (mixed)*	Halbrook et al., 2020[118]	n=679	Healthcare Practitioners and Technical Occupations and Protective Service Occupations (i.e. first responders)*	Cross-sectional survey	05/19 - 08/31	United States of America	8.1%	Moderate

For peer review only

First responders (mixed)*	Iwuji et al., 2020[119]	n=683	Healthcare Practitioners and Technical Occupations and Protective Service Occupations (i.e. first responders)*	Cross-sectional survey	05/12 - 05/13	United States of America	0.7%	High
First responders (mixed)*	Magyar et al., 2020[120]	n=70	Healthcare Practitioners and Technical Occupations and Protective Service Occupations (i.e. first responders)*	Cross-sectional survey	05/01 - 05/14	United States of America	4.29%	High
First responders (mixed)*	Martinez et al., 2020[121]	n=79	Healthcare Practitioners and Technical Occupations and Protective Service Occupations (i.e. first responders)*	Cross-sectional survey	04/16 - 04/17	United States of America	5.06%	High
First responders (mixed)*	Staletovich et al., 2020[122]	n=359	Healthcare Practitioners and Technical Occupations and Protective Service Occupations (i.e. first responders)*	Cross-sectional survey	05/17 - 05/22	United States of America	0%	Unclear
Healthcare Practitioners and Technical Occupations (29-0000)	Hibino et al., 2020[123]	n=806	Health Diagnosing and Treating Practitioners	Cross-sectional survey	06/01 - 07/30	Japan	0.74% (0.27-1.61%)	Unclear
Healthcare Practitioners and Technical Occupations (29-0000)	Jones et al., 2020[29]	n=856	Dentists, General	Cross-sectional survey	01/15 - 06/15	The United Kingdom	7.9%	High
Life, Physical, and Social Science	Calcagno et al., 2020[124]	n=343	Life, Physical, and Social Science Occupations	Cross-sectional survey	04/17 - 05/20	Italy	6.71%	Moderate

For peer review only

Occupations (19-0000)								
Healthcare Practitioners and Technical Occupations (29-0000)	Goenka et al., 2020[25]	n=49	Dietitians and Nutritionists	Cross-sectional survey	07/12 - 08/23	India	18.37%	Moderate
Healthcare Practitioners and Technical Occupations (29-0000)	Goenka et al., 2020[26]	n=6	Dietitians and Nutritionists	Cross-sectional survey	08/01 - 08/31	India	0%	High
Healthcare Practitioners and Technical Occupations (29-0000)	Akinbami et al., 2020[46]	n=321	Pharmacists	Cross-sectional survey	05/18 - 06/13	United States of America	4.4% (2.4-7.2%)	Moderate
Healthcare Practitioners and Technical Occupations (29-0000)	Alharbi et al., 2020[125]	n=5	Pharmacists	Cross-sectional survey	04/18 - 06/17	Saudi Arabia	0%	High
Healthcare Practitioners and Technical Occupations (29-0000)	Calcagno et al., 2020[124]	n=29	Pharmacists	Cross-sectional survey	04/17 - 05/20	Italy	3.45%	Moderate
Healthcare Practitioners and Technical Occupations (29-0000)	Chau et al., 2020[126]	n=17	Pharmacists	Cross-sectional survey	08/23 - 08/30	Viet Nam	0%	High
Healthcare Practitioners and Technical Occupations (29-0000)	Hanrath et al., 2020[32]	n=189	Pharmacists	Cross-sectional survey	05/29 - 07/06	The United Kingdom	4.76%	High

For peer review only

Healthcare Practitioners and Technical Occupations (29-0000)	Khan et al., 2020[127]	n=109	Pharmacists	Cross-sectional survey	06/15 - 06/29	India	0%	Moderate
Healthcare Practitioners and Technical Occupations (29-0000)	Mahomed et al., 2020[81]	n=404	Pharmacists	Cross-sectional survey	08/31 - 10/12	Mozambique	0.5%	High
Healthcare Practitioners and Technical Occupations (29-0000)	Martin et al., 2020[23]	n=113	Pharmacists	Cross-sectional survey	05/29 - 07/13	The United Kingdom	0%	Moderate
Healthcare Practitioners and Technical Occupations (29-0000)	Rosser et al., 2020[33]	n=213	Pharmacists	Cross-sectional survey	04/20 - 05/20	United States of America	1.88%	High
Healthcare Practitioners and Technical Occupations (29-0000)	Costa et al., 2020[128]	n=652	Physicians and Surgeons	Cross-sectional survey	05/14 - 05/28	Brazil	5.8%	Moderate
Healthcare Practitioners and Technical Occupations (29-0000)	Mohr et al., 2020[129]	n=372	Physicians and Surgeons	Cross-sectional survey	05/13 - 07/08	United States of America	1.61%	Moderate
Healthcare Practitioners and Technical Occupations (29-0000)	Nishida et al., 2020[90]	n=63	Physicians and Surgeons	Cross-sectional survey	06/12 - 06/19	Japan	3.2% (0.88-11%)	Moderate
Healthcare Practitioners and	Noor et al., 2020[130]	n=157	Physicians and Surgeons	Cross-sectional survey	07/13 - 07/15	Pakistan	17.83%	Moderate

For peer review only

Technical Occupations (29-0000)								
Healthcare Practitioners and Technical Occupations (29-0000)	Singhal et al., 2020[131]	n=208	Physicians and Surgeons	Cross-sectional survey	06/01 - 06/30	India	12.5%	High
Healthcare Practitioners and Technical Occupations (29-0000)	Morcuende et al., 2020[88]	n=23	Anesthesiologists	Cross-sectional survey	03/01 - 04/21	United States of America	13.04%	High
Healthcare Practitioners and Technical Occupations (29-0000)	Morcuende et al., 2020[88]	n=3	Obstetricians and Gynecologists	Cross-sectional survey	03/01 - 04/21	United States of America	100%	High
Healthcare Practitioners and Technical Occupations (29-0000)	Urbieta et al., 2020[132]	n=23	Pediatricians, General	Cross-sectional survey	04/14 - 04/16	Spain	4.3%	High
Healthcare Practitioners and Technical Occupations (29-0000)	Iversen et al., 2020[8]	n=1944	Psychiatrists	Cross-sectional survey	04/15 - 04/22	Denmark	1.85%	Low
Healthcare Practitioners and Technical Occupations (29-0000)	Leidner et al., 2020[22]	n=301	Surgeons	Cross sectional study with prospective cohort follow up of a subset of the sample	04/08 - 05/22	United States of America	2.66%	High
Healthcare Practitioners and Technical	Akinbami et al., 2020[46]	n=2297	Physicians and Surgeons, All Other	Cross-sectional survey	05/18 - 06/13	United States of America	6.1% (5.1-7.1%)	Moderate

For peer review only - http://bmjopen.bmj.com/site/about/guidelines.xhtml

Occupations (29-0000)								
Healthcare Practitioners and Technical Occupations (29-0000)	Alharbi et al., 2020[125]	n=18	Physicians and Surgeons, All Other	Cross-sectional survey	04/18 - 06/17	Saudi Arabia	27.78%	High
Healthcare Practitioners and Technical Occupations (29-0000)	Amendola et al., 2020[47]	n=214	Physicians and Surgeons, All Other	Cross-sectional survey	04/15 - 04/15	Italy	4.67%	High
Healthcare Practitioners and Technical Occupations (29-0000)	Baracco et al., 2020[24]	n=417	Physicians and Surgeons, All Other	Cross-sectional survey	04/23 - 05/05	Italy	17%	High
Healthcare Practitioners and Technical Occupations (29-0000)	Barallat et al., 2020[50]	n=1821	Physicians and Surgeons, All Other	Cross-sectional survey	05/04 - 05/22	Spain	11.81%	High
Healthcare Practitioners and Technical Occupations (29-0000)	Bianchi et al., 2020[133]	n=34	Physicians and Surgeons, All Other	Cross-sectional survey	04/15 - 05/15	Italy	5.88%	Unclear
Healthcare Practitioners and Technical Occupations (29-0000)	Blairon et al., 2020[52]	n=323	Physicians and Surgeons, All Other	Cross-sectional survey	05/25 - 06/19	Belgium	11.8%	High
Healthcare Practitioners and Technical Occupations (29-0000)	Brehm et al., 2020[7]	n=275	Physicians and Surgeons, All Other	Cross sectional study with prospective cohort follow up of a	03/20 - 07/17	Germany	3.3%	Moderate

For peer review only

				subset of the sample				
Healthcare Practitioners and Technical Occupations (29-0000)	Brousseau et al., 2020[134]	n=432	Physicians and Surgeons, All Other	Cross-sectional survey	07/06 - 09/24	Canada	7.2%	High
Healthcare Practitioners and Technical Occupations (29-0000)	Calcagno et al., 2020[124]	n=700	Physicians and Surgeons, All Other	Cross-sectional survey	04/17 - 05/20	Italy	7.86%	Moderate
Healthcare Practitioners and Technical Occupations (29-0000)	Chau et al., 2020[126]	n=64	Physicians and Surgeons, All Other	Cross-sectional survey	08/23 - 08/30	Viet Nam	0%	High
Healthcare Practitioners and Technical Occupations (29-0000)	Chen et al., 2020[135]	n=17	Physicians and Surgeons, All Other	Cross-sectional survey	02/19 - 02/19	China	41.18%	High
Healthcare Practitioners and Technical Occupations (29-0000)	Erber et al., 2020[31]	n=860	Physicians and Surgeons, All Other	Cross-sectional survey	04/14 - 05/29	Germany	1.63%	High
Healthcare Practitioners and Technical Occupations (29-0000)	Favara et al., 2020[136]	n=15	Physicians and Surgeons, All Other	Prospective cohort	06/01 - 06/07	The United Kingdom	13.33%	High
Healthcare Practitioners and Technical Occupations (29-0000)	Favara et al., 2020[19]	n=82	Physicians and Surgeons, All Other	Cross-sectional survey	07/13 - 07/13	The United Kingdom	10.9%	High

For peer review only

Healthcare Practitioners and Technical Occupations (29-0000)	Fujita et al., 2020[137]	n=42	Physicians and Surgeons, All Other	Cross-sectional survey	04/10 - 04/20	Japan	4.7%	High
Healthcare Practitioners and Technical Occupations (29-0000)	Galan et al., 2020[20]	n=564	Physicians and Surgeons, All Other	Cross-sectional survey	04/14 - 04/27	Spain	39.36%	High
Healthcare Practitioners and Technical Occupations (29-0000)	Godbout et al., 2020[138]	n=490	Physicians and Surgeons, All Other	Cross-sectional survey	07/27 - 10/02	United States of America	1.43%	High
Healthcare Practitioners and Technical Occupations (29-0000)	Goenka et al., 2020[25]	n=255	Physicians and Surgeons, All Other	Cross-sectional survey	07/12 - 08/23	India	3.92%	Moderate
Healthcare Practitioners and Technical Occupations (29-0000)	Goenka et al., 2020[26]	n=29	Physicians and Surgeons, All Other	Cross-sectional survey	08/01 - 08/31	India	20.69%	High
Healthcare Practitioners and Technical Occupations (29-0000)	Hanrath et al., 2020[32]	n=899	Physicians and Surgeons, All Other	Cross-sectional survey	05/29 - 07/06	The United Kingdom	7.01%	High
Healthcare Practitioners and Technical Occupations (29-0000)	Houlihan et al., 2020[139]	n=72	Physicians and Surgeons, All Other	Cross-sectional survey	03/26 - 04/08	The United Kingdom	22%	High
Healthcare Practitioners and	Hunter et al., 2020[21]	n=279	Physicians and Surgeons, All Other	Cross-sectional survey	04/29 - 05/08	United States of America	1.08%	High

For peer review only

Technical Occupations (29-0000)								
Healthcare Practitioners and Technical Occupations (29-0000)	Insua et al., 2020[140]	n=116	Physicians and Surgeons, All Other	Cross-sectional survey	06/08 - 06/09	Argentina	0.9% (0.1-5.5%)	High
Healthcare Practitioners and Technical Occupations (29-0000)	Iversen et al., 2020[8]	n=4698	Physicians and Surgeons, All Other	Cross-sectional survey	04/15 - 04/22	Denmark	4.07%	Low
Healthcare Practitioners and Technical Occupations (29-0000)	Iversen et al., 2020[8]	n=113	Physicians and Surgeons, All Other	Cross-sectional survey	04/15 - 04/22	Denmark	7.08%	Low
Healthcare Practitioners and Technical Occupations (29-0000)	Jeremias et al., 2020[70]	n=79	Physicians and Surgeons, All Other	Cross-sectional survey	03/01 - 04/30	United States of America	11.4%	High
Healthcare Practitioners and Technical Occupations (29-0000)	Kassem et al., 2020[72]	n=30	Physicians and Surgeons, All Other	Cross-sectional survey	06/01 - 06/14	Egypt	6.66%	High
Healthcare Practitioners and Technical Occupations (29-0000)	Kassem et al., 2020[72]	n=30	Physicians and Surgeons, All Other	Cross-sectional survey	06/01 - 06/14	Egypt	3.33%	High
Healthcare Practitioners and Technical	Kassem et al., 2020[72]	n=30	Physicians and Surgeons, All Other	Cross-sectional survey	06/01 - 06/14	Egypt	0%	High

For peer review only

Occupations (29-0000)								
Healthcare Practitioners and Technical Occupations (29-0000)	Kassem et al., 2020[72]	n=30	Physicians and Surgeons, All Other	Cross-sectional survey	06/01 - 06/14	Egypt	3.33%	High
Healthcare Practitioners and Technical Occupations (29-0000)	Khan et al., 2020[127]	n=980	Physicians and Surgeons, All Other	Cross-sectional survey	06/15 - 06/29	India	2.8% (1.9-4%)	Moderate
Healthcare Practitioners and Technical Occupations (29-0000)	Kohler et al., 2020[141]	n=268	Physicians and Surgeons, All Other	Cross-sectional survey	03/19 - 04/03	Switzerland	1.49%	High
Healthcare Practitioners and Technical Occupations (29-0000)	Kumar et al., 2020[142]	n=201	Physicians and Surgeons, All Other	Cross-sectional survey	06/01 - 06/30	India	7% (4.2-11.4%)	High
Healthcare Practitioners and Technical Occupations (29-0000)	Leidner et al., 2020[22]	n=1081	Physicians and Surgeons, All Other	Cross sectional study with prospective cohort follow up of a subset of the sample	04/08 - 05/22	United States of America	3.33%	High
Healthcare Practitioners and Technical Occupations (29-0000)	Lumley et al., 2020[9]	n=1859	Physicians and Surgeons, All Other	Prospective cohort	04/23 - 11/30	The United Kingdom	10.11%	Moderate
Healthcare Practitioners and Technical	Martin et al., 2020[23]	n=1243	Physicians and Surgeons, All Other	Cross-sectional survey	05/29 - 07/13	The United Kingdom	10.3%	Moderate

For peer review only

Occupations (29-0000)								
Healthcare Practitioners and Technical Occupations (29-0000)	Mesnil et al., 2020[143]	n=111	Physicians and Surgeons, All Other	Cross-sectional survey	06/08 - 06/22	France	11%	High
Healthcare Practitioners and Technical Occupations (29-0000)	Missaglia et al., 2020[144]	n=377	Physicians and Surgeons, All Other	Cross-sectional survey	04/01 - 04/30	Italy	14.9%	High
Healthcare Practitioners and Technical Occupations (29-0000)	Mohr et al., 2020[129]	n=272	Physicians and Surgeons, All Other	Cross-sectional survey	05/13 - 07/08	United States of America	2.94%	Moderate
Healthcare Practitioners and Technical Occupations (29-0000)	Moscola et al., 2020[89]	n=3746	Physicians and Surgeons, All Other	Cross-sectional survey	04/20 - 06/23	United States of America	8.7%	High
Healthcare Practitioners and Technical Occupations (29-0000)	Nishida et al., 2020[90]	n=149	Physicians and Surgeons, All Other	Cross-sectional survey	06/12 - 06/19	Japan	1.3% (0.37-4.8%)	Moderate
Healthcare Practitioners and Technical Occupations (29-0000)	Nishida et al., 2020[90]	n=46	Physicians and Surgeons, All Other	Cross-sectional survey	06/12 - 06/19	Japan	0%	Moderate
Healthcare Practitioners and Technical Occupations (29-0000)	Nishida et al., 2020[90]	n=40	Physicians and Surgeons, All Other	Cross-sectional survey	06/12 - 06/19	Japan	0%	Moderate

For peer review only

Healthcare Practitioners and Technical Occupations (29-0000)	Nishida et al., 2020[90]	n=59	Physicians and Surgeons, All Other	Cross-sectional survey	06/12 - 06/19	Japan	1.7% (0.3-9%)	Moderate
Healthcare Practitioners and Technical Occupations (29-0000)	Nishida et al., 2020[90]	n=925	Physicians and Surgeons, All Other	Cross-sectional survey	06/12 - 06/19	Japan	0.43% (0.17-1.1%)	Moderate
Healthcare Practitioners and Technical Occupations (29-0000)	Noor et al., 2020[130]	n=303	Physicians and Surgeons, All Other	Cross-sectional survey	07/13 - 07/15	Pakistan	19.8%	Moderate
Healthcare Practitioners and Technical Occupations (29-0000)	Orth-Holler et al., 2020[145]	n=377	Physicians and Surgeons, All Other	Cross-sectional survey	03/20 - 03/27	Austria	0.3% (0.01-1.5%)	High
Healthcare Practitioners and Technical Occupations (29-0000)	Plebani et al., 2020[146]	n=2337	Physicians and Surgeons, All Other	Cross-sectional survey	02/22 - 05/29	Italy	3.6% (2.8-4.4%)	High
Healthcare Practitioners and Technical Occupations (29-0000)	Rosser et al., 2020[33]	n=2533	Physicians and Surgeons, All Other	Cross-sectional survey	04/20 - 05/20	United States of America	1.07%	High
Healthcare Practitioners and Technical Occupations (29-0000)	Rudberg et al., 2020[147]	n=439	Physicians and Surgeons, All Other	Cross-sectional survey	04/14 - 05/08	Sweden	19.1%	Moderate
Healthcare Practitioners and	Schmidt et al., 2020[148]	n=34	Physicians and Surgeons, All Other	Cross-sectional survey	04/20 - 04/30	Germany	8.82%	High

For peer review only

Technical Occupations (29-0000)								
Healthcare Practitioners and Technical Occupations (29-0000)	Sotgiu et al., 2020[149]	n=115	Physicians and Surgeons, All Other	Cross-sectional survey	04/02 - 04/16	Italy	6.09%	High
Healthcare Practitioners and Technical Occupations (29-0000)	Venugopal et al., 2020[150]	n=157	Physicians and Surgeons, All Other	Cross-sectional survey	03/01 - 05/01	United States of America	25%	Moderate
Healthcare Practitioners and Technical Occupations (29-0000)	Yogo et al., 2020[36]	n=110	Physicians and Surgeons, All Other	Cross-sectional survey	05/20 - 06/08	United States of America	1.82%	High
Healthcare Practitioners and Technical Occupations (29-0000)	Brzostek et al., 2020[151]	n=998	Physician Assistants	Cross-sectional survey	04/17 - 05/07	United States of America	28.3%	Moderate
Healthcare Practitioners and Technical Occupations (29-0000)	Hoffmann et al., 2020[152]	n=156	Physician Assistants	Prospective cohort	07/01 - 07/31	Germany	1.3%	High
Healthcare Practitioners and Technical Occupations (29-0000)	Mohr et al., 2020[129]	n=156	Physician Assistants	Cross-sectional survey	05/13 - 07/08	United States of America	0.64%	Moderate
Healthcare Practitioners and Technical	Morcuende et al., 2020[88]	n=6	Physician Assistants	Cross-sectional survey	03/01 - 04/21	United States of America	9.43%	High

For peer review only

Occupations (29-0000)								
Healthcare Practitioners and Technical Occupations (29-0000)	Morcuende et al., 2020[88]	n=53	Physician Assistants	Cross-sectional survey	03/01 - 04/21	United States of America	9.43%	High
Healthcare Practitioners and Technical Occupations (29-0000)	Patel et al., 2020[153]	n=230	Physician Assistants	Prospective cohort	06/02 - 06/27	United States of America	3.48%	High
Healthcare Practitioners and Technical Occupations (29-0000)	Self et al., 2020[154]	n=919	Physician Assistants	Cross-sectional survey	04/03 - 06/19	United States of America	5.66%	Moderate
Healthcare Practitioners and Technical Occupations (29-0000)	Shah et al., 2020[155]	n=248	Physician Assistants	Cross-sectional survey	05/25 - 07/09	United States of America	0%	High
Healthcare Practitioners and Technical Occupations (29-0000)	Shah et al., 2020[155]	n=320	Physician Assistants	Cross-sectional survey	05/25 - 07/09	United States of America	0.63%	High
Healthcare Practitioners and Technical Occupations (29-0000)	Lumley et al., 2020[9]	n=386	Occupational Therapists	Prospective cohort	04/23 - 11/30	The United Kingdom	11.4%	Moderate
Healthcare Practitioners and Technical Occupations (29-0000)	Akinbami et al., 2020[46]	n=235	Physical Therapists	Cross-sectional survey	05/18 - 06/13	United States of America	10.6% (7-15.3%)	Moderate

Healthcare Practitioners and Technical Occupations (29-0000)	Brehm et al., 2020[7]	n=15	Physical Therapists	Cross sectional study with prospective cohort follow up of a subset of the sample	03/20 - 07/17	Germany	0%	Moderate
Healthcare Practitioners and Technical Occupations (29-0000)	Cooper et al., 2020[59]	n=84	Physical Therapists	Cross-sectional survey	06/10 - 08/07	The United Kingdom	10.71%	Moderate
Healthcare Practitioners and Technical Occupations (29-0000)	Costa et al., 2020[128]	n=159	Physical Therapists	Cross-sectional survey	05/14 - 05/28	Brazil	10.7%	Moderate
Healthcare Practitioners and Technical Occupations (29-0000)	Akinbami et al., 2020[46]	n=409	Respiratory Therapists	Cross-sectional survey	05/18 - 06/13	United States of America	8.3% (5.8-11.4%)	Moderate
Healthcare Practitioners and Technical Occupations (29-0000)	Brunner et al., 2020[54]	n=42	Respiratory Therapists	Cross-sectional survey	05/04 - 05/29	United States of America	0%	High
Healthcare Practitioners and Technical Occupations (29-0000)	Godbout et al., 2020[138]	n=25	Respiratory Therapists	Cross-sectional survey	07/27 - 10/02	United States of America	0%	High
Healthcare Practitioners and Technical Occupations (29-0000)	Hunter et al., 2020[21]	n=94	Respiratory Therapists	Cross-sectional survey	04/29 - 05/08	United States of America	0%	High

For peer review only

Healthcare Practitioners and Technical Occupations (29-0000)	Rosser et al., 2020[33]	n=135	Respiratory Therapists	Cross-sectional survey	04/20 - 05/20	United States of America	0%	High
Healthcare Practitioners and Technical Occupations (29-0000)	Self et al., 2020[154]	n=235	Respiratory Therapists	Cross-sectional survey	04/03 - 06/19	United States of America	4.26%	Moderate
Healthcare Practitioners and Technical Occupations (29-0000)	Yogo et al., 2020[36]	n=121	Respiratory Therapists	Cross-sectional survey	05/20 - 06/08	United States of America	0%	High
Healthcare Practitioners and Technical Occupations (29-0000)	Rosser et al., 2020[33]	n=253	Therapists, All Other	Cross-sectional survey	04/20 - 05/20	United States of America	1.58%	High
Healthcare Practitioners and Technical Occupations (29-0000)	Schmidt et al., 2020[148]	n=80	Therapists, All Other	Cross-sectional survey	04/20 - 04/30	Germany	3.75%	High
Healthcare Practitioners and Technical Occupations (29-0000)	Yogo et al., 2020[36]	n=22	Therapists, All Other	Cross-sectional survey	05/20 - 06/08	United States of America	4.55%	High
Healthcare Practitioners and Technical Occupations (29-0000)	Calcagno et al., 2020[124]	n=13	Veterinarians	Cross-sectional survey	04/17 - 05/20	Italy	0%	Moderate
Healthcare Practitioners and	Akinbami et al., 2020[46]	n=6426	Registered Nurses	Cross-sectional survey	05/18 - 06/13	United States of America	7.7% (7.1-8.4%)	Moderate

For peer review only

Technical Occupations (29-0000)								
Healthcare Practitioners and Technical Occupations (29-0000)	Alharbi et al., 2020[125]	n=70	Registered Nurses	Cross-sectional survey	04/18 - 06/17	Saudi Arabia	10%	High
Healthcare Practitioners and Technical Occupations (29-0000)	Alharbi et al., 2020[125]	n=9	Registered Nurses	Cross-sectional survey	04/18 - 06/17	Saudi Arabia	33.33%	High
Healthcare Practitioners and Technical Occupations (29-0000)	Alharbi et al., 2020[125]	n=76	Registered Nurses	Cross-sectional survey	04/18 - 06/17	Saudi Arabia	26.32%	High
Healthcare Practitioners and Technical Occupations (29-0000)	Alharbi et al., 2020[125]	n=21	Registered Nurses	Cross-sectional survey	04/18 - 06/17	Saudi Arabia	14.29%	High
Healthcare Practitioners and Technical Occupations (29-0000)	Alharbi et al., 2020[125]	n=43	Registered Nurses	Cross-sectional survey	04/18 - 06/17	Saudi Arabia	27.91%	High
Healthcare Practitioners and Technical Occupations (29-0000)	Amendola et al., 2020[47]	n=216	Registered Nurses	Cross-sectional survey	04/15 - 04/15	Italy	6.02%	High
Healthcare Practitioners and Technical	Bampoe et al., 2020[156]	n=52	Registered Nurses	Cross-sectional survey	05/11 - 06/05	The United Kingdom	13.5% (5.6-25.8%)	High

For peer review only

Occupations (29-0000)								
Healthcare Practitioners and Technical Occupations (29-0000)	Bampoe et al., 2020[156]	n=40	Registered Nurses	Cross-sectional survey	05/11 - 06/05	The United Kingdom	12.5% (4.2-26.8%)	High
Healthcare Practitioners and Technical Occupations (29-0000)	Baracco et al., 2020[24]	n=1014	Registered Nurses	Cross-sectional survey	04/23 - 05/05	Italy	17.9%	High
Healthcare Practitioners and Technical Occupations (29-0000)	Barallat et al., 2020[50]	n=2243	Registered Nurses	Cross-sectional survey	05/04 - 05/22	Spain	10.64%	High
Healthcare Practitioners and Technical Occupations (29-0000)	Brehm et al., 2020[7]	n=444	Registered Nurses	Cross sectional study with prospective cohort follow up of a subset of the sample	03/20 - 07/17	Germany	2.3%	Moderate
Healthcare Practitioners and Technical Occupations (29-0000)	Brousseau et al., 2020[134]	n=1189	Registered Nurses	Cross-sectional survey	07/06 - 09/24	Canada	11.9%	High
Healthcare Practitioners and Technical Occupations (29-0000)	Calcagno et al., 2020[124]	n=1833	Registered Nurses	Cross-sectional survey	04/17 - 05/20	Italy	8.18%	Moderate
Healthcare Practitioners and Technical	Chau et al., 2020[126]	n=144	Registered Nurses	Cross-sectional survey	08/23 - 08/30	Viet Nam	0%	High

For peer review only

Occupations (29-0000)								
Healthcare Practitioners and Technical Occupations (29-0000)	Chen et al., 2020[135]	n=25	Registered Nurses	Cross-sectional survey	02/19 - 02/19	China	8%	High
Healthcare Practitioners and Technical Occupations (29-0000)	Cooper et al., 2020[59]	n=3471	Registered Nurses	Cross-sectional survey	06/10 - 08/07	The United Kingdom	7.52%	Moderate
Healthcare Practitioners and Technical Occupations (29-0000)	Costa et al., 2020[128]	n=370	Registered Nurses	Cross-sectional survey	05/14 - 05/28	Brazil	11.4%	Moderate
Healthcare Practitioners and Technical Occupations (29-0000)	Dimcheff et al., 2020[157]	n=412	Registered Nurses	Cross-sectional survey	06/08 - 07/08	United States of America	7%	Moderate
Healthcare Practitioners and Technical Occupations (29-0000)	Erber et al., 2020[31]	n=958	Registered Nurses	Cross-sectional survey	04/14 - 05/29	Germany	2.5%	High
Healthcare Practitioners and Technical Occupations (29-0000)	Favara et al., 2020[136]	n=45	Registered Nurses	Prospective cohort	06/01 - 06/07	The United Kingdom	28.89%	High
Healthcare Practitioners and Technical Occupations (29-0000)	Favara et al., 2020[19]	n=237	Registered Nurses	Cross-sectional survey	07/13 - 07/13	The United Kingdom	16.5%	High

For peer review only

Healthcare Practitioners and Technical Occupations (29-0000)	Finkenzeller et al., 2020[158]	n=251	Registered Nurses	Prospective cohort	06/29 - 07/29	Germany	12%	Moderate
Healthcare Practitioners and Technical Occupations (29-0000)	Finkenzeller et al., 2020[158]	n=887	Registered Nurses	Prospective cohort	06/29 - 07/29	Germany	20%	Moderate
Healthcare Practitioners and Technical Occupations (29-0000)	Fujita et al., 2020[137]	n=50	Registered Nurses	Cross-sectional survey	04/10 - 04/20	Japan	6%	High
Healthcare Practitioners and Technical Occupations (29-0000)	Galan et al., 2020[20]	n=687	Registered Nurses	Cross-sectional survey	04/14 - 04/27	Spain	30.71%	High
Healthcare Practitioners and Technical Occupations (29-0000)	Godbout et al., 2020[138]	n=937	Registered Nurses	Cross-sectional survey	07/27 - 10/02	United States of America	1.39%	High
Healthcare Practitioners and Technical Occupations (29-0000)	Goenka et al., 2020[25]	n=224	Registered Nurses	Cross-sectional survey	07/12 - 08/23	India	9.38%	Moderate
Healthcare Practitioners and Technical Occupations (29-0000)	Goenka et al., 2020[26]	n=43	Registered Nurses	Cross-sectional survey	08/01 - 08/31	India	34.88%	High
Healthcare Practitioners and	Grant et al., 2020[159]	n=1345	Registered Nurses	Cross-sectional survey	05/15 - 06/05	The United Kingdom	34.7%	High

For peer review only

Technical Occupations (29-0000)								
Healthcare Practitioners and Technical Occupations (29-0000)	Grant et al., 2020[159]	n=108	Registered Nurses	Cross-sectional survey	05/15 - 06/05	The United Kingdom	25%	High
Healthcare Practitioners and Technical Occupations (29-0000)	Hanrath et al., 2020[32]	n=749	Registered Nurses	Cross-sectional survey	05/29 - 07/06	The United Kingdom	8.99%	High
Healthcare Practitioners and Technical Occupations (29-0000)	Haq et al., 2020[67]	n=209	Registered Nurses	Cross-sectional survey	06/15 - 06/29	Pakistan	38.8% (32.1-45.7%)	Moderate
Healthcare Practitioners and Technical Occupations (29-0000)	Houlihan et al., 2020[139]	n=106	Registered Nurses	Cross-sectional survey	03/26 - 04/08	The United Kingdom	24%	High
Healthcare Practitioners and Technical Occupations (29-0000)	Houlihan et al., 2020[139]	n=22	Registered Nurses	Cross-sectional survey	03/26 - 04/08	The United Kingdom	23%	High
Healthcare Practitioners and Technical Occupations (29-0000)	Hunter et al., 2020[21]	n=317	Registered Nurses	Cross-sectional survey	04/29 - 05/08	United States of America	2.2%	High
Healthcare Practitioners and Technical	Iversen et al., 2020[8]	n=9963	Registered Nurses	Cross-sectional survey	04/15 - 04/22	Denmark	4.03%	Low

For peer review only

Occupations (29-0000)								
Healthcare Practitioners and Technical Occupations (29-0000)	Iversen et al., 2020[8]	n=1786	Registered Nurses	Cross-sectional survey	04/15 - 04/22	Denmark	4.65%	Low
Healthcare Practitioners and Technical Occupations (29-0000)	Jeremias et al., 2020[70]	n=1043	Registered Nurses	Cross-sectional survey	03/01 - 04/30	United States of America	9.5%	High
Healthcare Practitioners and Technical Occupations (29-0000)	Jones et al., 2020[29]	n=1962	Registered Nurses	Cross-sectional survey	01/15 - 06/15	The United Kingdom	10.5%	High
Healthcare Practitioners and Technical Occupations (29-0000)	Kassem et al., 2020[72]	n=28	Registered Nurses	Cross-sectional survey	06/01 - 06/14	Egypt	10.71%	High
Healthcare Practitioners and Technical Occupations (29-0000)	Kassem et al., 2020[72]	n=28	Registered Nurses	Cross-sectional survey	06/01 - 06/14	Egypt	7.14%	High
Healthcare Practitioners and Technical Occupations (29-0000)	Kassem et al., 2020[72]	n=28	Registered Nurses	Cross-sectional survey	06/01 - 06/14	Egypt	3.57%	High
Healthcare Practitioners and Technical Occupations (29-0000)	Kassem et al., 2020[72]	n=28	Registered Nurses	Cross-sectional survey	06/01 - 06/14	Egypt	0%	High

For peer review only

Healthcare Practitioners and Technical Occupations (29-0000)	Khan et al., 2020[127]	n=321	Registered Nurses	Cross-sectional survey	06/15 - 06/29	India	2.8% (1.5-5.3%)	Moderate
Healthcare Practitioners and Technical Occupations (29-0000)	Kohler et al., 2020[141]	n=398	Registered Nurses	Cross-sectional survey	03/19 - 04/03	Switzerland	0.75%	High
Healthcare Practitioners and Technical Occupations (29-0000)	Kumar et al., 2020[142]	n=308	Registered Nurses	Cross-sectional survey	06/01 - 06/30	India	6.8% (4.5-10.2%)	High
Healthcare Practitioners and Technical Occupations (29-0000)	Leidner et al., 2020[22]	n=110	Registered Nurses	Cross sectional study with prospective cohort follow up of a subset of the sample	04/08 - 05/22	United States of America	0%	High
Healthcare Practitioners and Technical Occupations (29-0000)	Leidner et al., 2020[22]	n=3504	Registered Nurses	Cross sectional study with prospective cohort follow up of a subset of the sample	04/08 - 05/22	United States of America	2.34%	High
Healthcare Practitioners and Technical Occupations (29-0000)	Lumley et al., 2020[9]	n=4528	Registered Nurses	Prospective cohort	04/23 - 11/30	The United Kingdom	13.21%	Moderate
Healthcare Practitioners and Technical Occupations (29-0000)	Mansour et al., 2020[160]	n=285	Registered Nurses	Cross-sectional survey	03/24 - 04/04	United States of America	32.63%	High

For peer review only

Healthcare Practitioners and Technical Occupations (29-0000)	Martin et al., 2020[161]	n=580	Registered Nurses	Cross-sectional survey	04/01 - 04/15	Spain	5.52%	High
Healthcare Practitioners and Technical Occupations (29-0000)	Martin et al., 2020[161]	n=74	Registered Nurses	Cross-sectional survey	04/01 - 04/15	Spain	9.46%	High
Healthcare Practitioners and Technical Occupations (29-0000)	Martin et al., 2020[161]	n=676	Registered Nurses	Cross-sectional survey	04/01 - 04/15	Spain	5.92%	High
Healthcare Practitioners and Technical Occupations (29-0000)	Martin et al., 2020[161]	n=337	Registered Nurses	Cross-sectional survey	04/01 - 04/15	Spain	5.93%	High
Healthcare Practitioners and Technical Occupations (29-0000)	Martin et al., 2020[161]	n=339	Registered Nurses	Cross-sectional survey	04/01 - 04/15	Spain	5.9%	High
Healthcare Practitioners and Technical Occupations (29-0000)	Meissner et al., 2020[162]	n=439	Registered Nurses	Cross-sectional survey	04/14 - 05/06	United States of America	1.37%	High
Healthcare Practitioners and Technical Occupations (29-0000)	Mohr et al., 2020[129]	n=410	Registered Nurses	Cross-sectional survey	05/13 - 07/08	United States of America	1.46%	Moderate
Healthcare Practitioners and	Moscola et al., 2020[89]	n=11468	Registered Nurses	Cross-sectional survey	04/20 - 06/23	United States of America	13.1%	High

For peer review only

Technical Occupations (29-0000)								
Healthcare Practitioners and Technical Occupations (29-0000)	Mostafa et al., 2020[163]	n=4040	Registered Nurses	Cross-sectional survey	04/22 - 05/14	Egypt	1.31%	High
Healthcare Practitioners and Technical Occupations (29-0000)	Nishida et al., 2020[90]	n=489	Registered Nurses	Cross-sectional survey	06/12 - 06/19	Japan	0.2% (0.04-1.1%)	Moderate
Healthcare Practitioners and Technical Occupations (29-0000)	Noor et al., 2020[130]	n=460	Registered Nurses	Cross-sectional survey	07/13 - 07/15	Pakistan	39.78%	Moderate
Healthcare Practitioners and Technical Occupations (29-0000)	Paradiso et al., 2020[164]	n=606	Registered Nurses	Cross sectional study with prospective cohort follow up of a subset of the sample	03/26 - 04/17	Italy	0.33%	High
Healthcare Practitioners and Technical Occupations (29-0000)	Plebani et al., 2020[146]	n=3230	Registered Nurses	Cross-sectional survey	02/22 - 05/29	Italy	4.7% (4-5.5%)	High
Healthcare Practitioners and Technical Occupations (29-0000)	Poustchi et al., 2020[28]	n=1245	Registered Nurses	Cross-sectional survey	04/17 - 06/02	Iran (Islamic Republic of)	15.9% (13.9-18%)	Moderate
Healthcare Practitioners and Technical	Rudberg et al., 2020[147]	n=636	Registered Nurses	Cross-sectional survey	04/14 - 05/08	Sweden	21.9%	Moderate

Occupations (29-0000)								
Healthcare Practitioners and Technical Occupations (29-0000)	Schmidt et al., 2020[148]	n=154	Registered Nurses	Cross-sectional survey	04/20 - 04/30	Germany	0%	High
Healthcare Practitioners and Technical Occupations (29-0000)	Self et al., 2020[154]	n=1445	Registered Nurses	Cross-sectional survey	04/03 - 06/19	United States of America	5.05%	Moderate
Healthcare Practitioners and Technical Occupations (29-0000)	Siddiqui et al., 2020[2]	n=59	Registered Nurses	Prospective cohort	04/15 - 08/15	India	10.2%	High
Healthcare Practitioners and Technical Occupations (29-0000)	Siddiqui et al., 2020[2]	n=70	Registered Nurses	Prospective cohort	04/15 - 08/15	India	10%	High
Healthcare Practitioners and Technical Occupations (29-0000)	Sotgiu et al., 2020[149]	n=64	Registered Nurses	Cross-sectional survey	04/02 - 04/16	Italy	7.8% (1.2-14.4%)	High
Healthcare Practitioners and Technical Occupations (29-0000)	Sydney et al., 2020[165]	n=81	Registered Nurses	Cross-sectional survey	04/28 - 05/04	United States of America	18.52%	High
Healthcare Practitioners and Technical Occupations (29-0000)	Urbieta et al., 2020[132]	n=83	Registered Nurses	Cross-sectional survey	04/14 - 04/16	Spain	4.8%	High

For peer review only

Healthcare Practitioners and Technical Occupations (29-0000)	Urbieta et al., 2020[132]	n=23	Registered Nurses	Cross-sectional survey	04/14 - 04/16	Spain	8.7%	High
Healthcare Practitioners and Technical Occupations (29-0000)	Venugopal et al., 2020[150]	n=142	Registered Nurses	Cross-sectional survey	03/01 - 05/01	United States of America	28%	Moderate
Healthcare Practitioners and Technical Occupations (29-0000)	Yogo et al., 2020[36]	n=1129	Registered Nurses	Cross-sectional survey	05/20 - 06/08	United States of America	2.48%	High
Healthcare Practitioners and Technical Occupations (29-0000)	Yogo et al., 2020[36]	n=12	Registered Nurses	Cross-sectional survey	05/20 - 06/08	United States of America	0%	High
Healthcare Practitioners and Technical Occupations (29-0000)	Zhou et al., 2020[166]	n=2406	Registered Nurses	Cross-sectional survey	03/16 - 03/25	China	1.37%	Moderate
Healthcare Practitioners and Technical Occupations (29-0000)	Godbout et al., 2020[138]	n=141	Nurse Practitioners	Cross-sectional survey	07/27 - 10/02	United States of America	1.42%	High
Healthcare Practitioners and Technical Occupations (29-0000)	Dimcheff et al., 2020[157]	n=214	Nurse Practitioners	Cross-sectional survey	06/08 - 07/08	United States of America	3.7%	Moderate
Healthcare Practitioners and	Akinbami et al., 2020[46]	n=719	Health Technologists and Technicians	Cross-sectional survey	05/18 - 06/13	United States of America	4.2% (2.8-5.9%)	Moderate

For peer review only

Technical Occupations (29-0000)								
Healthcare Practitioners and Technical Occupations (29-0000)	Blairon et al., 2020[52]	n=61	Health Technologists and Technicians	Cross-sectional survey	05/25 - 06/19	Belgium	6.6%	High
Healthcare Practitioners and Technical Occupations (29-0000)	Yogo et al., 2020[36]	n=65	Health Technologists and Technicians	Cross-sectional survey	05/20 - 06/08	United States of America	4.62%	High
Healthcare Practitioners and Technical Occupations (29-0000)	Silva et al., 2020[34]	n=224	Clinical Laboratory Technologists and Technicians	Cross-sectional survey	06/05 - 07/31	Brazil	7.59%	High
Healthcare Practitioners and Technical Occupations (29-0000)	Costa et al., 2020[128]	n=66	Medical and Clinical Laboratory Technologists	Cross-sectional survey	05/14 - 05/28	Brazil	3%	Moderate
Healthcare Practitioners and Technical Occupations (29-0000)	Akinbami et al., 2020[46]	n=293	Medical and Clinical Laboratory Technicians	Cross-sectional survey	05/18 - 06/13	United States of America	3.4% (1.7-6.2%)	Moderate
Healthcare Practitioners and Technical Occupations (29-0000)	Akinbami et al., 2020[46]	n=365	Medical and Clinical Laboratory Technicians	Cross-sectional survey	05/18 - 06/13	United States of America	5.5% (3.4-8.3%)	Moderate
Healthcare Practitioners and Technical	Alharbi et al., 2020[125]	n=80	Medical and Clinical Laboratory Technicians	Cross-sectional survey	04/18 - 06/17	Saudi Arabia	20%	High

For peer review only

Occupations (29-0000)								
Healthcare Practitioners and Technical Occupations (29-0000)	Baracco et al., 2020[24]	n=256	Medical and Clinical Laboratory Technicians	Cross-sectional survey	04/23 - 05/05	Italy	12.1%	High
Healthcare Practitioners and Technical Occupations (29-0000)	Brehm et al., 2020[7]	n=105	Medical and Clinical Laboratory Technicians	Cross sectional study with prospective cohort follow up of a subset of the sample	03/20 - 07/17	Germany	0%	Moderate
Healthcare Practitioners and Technical Occupations (29-0000)	Calcagno et al., 2020[124]	n=216	Medical and Clinical Laboratory Technicians	Cross-sectional survey	04/17 - 05/20	Italy	6.94%	Moderate
Healthcare Practitioners and Technical Occupations (29-0000)	Calcagno et al., 2020[124]	n=157	Medical and Clinical Laboratory Technicians	Cross-sectional survey	04/17 - 05/20	Italy	11.46%	Moderate
Healthcare Practitioners and Technical Occupations (29-0000)	Chau et al., 2020[126]	n=33	Medical and Clinical Laboratory Technicians	Cross-sectional survey	08/23 - 08/30	Viet Nam	0%	High
Healthcare Practitioners and Technical Occupations (29-0000)	Galan et al., 2020[20]	n=192	Medical and Clinical Laboratory Technicians	Cross-sectional survey	04/14 - 04/27	Spain	21.35%	High
Healthcare Practitioners and Technical	Goenka et al., 2020[25]	n=72	Medical and Clinical Laboratory Technicians	Cross-sectional survey	07/12 - 08/23	India	15.28%	Moderate

For peer review only

Occupations (29-0000)								
Healthcare Practitioners and Technical Occupations (29-0000)	Haq et al., 2020[67]	n=32	Medical and Clinical Laboratory Technicians	Cross-sectional survey	06/15 - 06/29	Pakistan	50% (31.8-68.1%)	Moderate
Healthcare Practitioners and Technical Occupations (29-0000)	Iversen et al., 2020[8]	n=1292	Medical and Clinical Laboratory Technicians	Cross-sectional survey	04/15 - 04/22	Denmark	1.93%	Low
Healthcare Practitioners and Technical Occupations (29-0000)	Khan et al., 2020[127]	n=397	Medical and Clinical Laboratory Technicians	Cross-sectional survey	06/15 - 06/29	India	2.5% (1.4-4.6%)	Moderate
Healthcare Practitioners and Technical Occupations (29-0000)	Lumley et al., 2020[9]	n=452	Medical and Clinical Laboratory Technicians	Prospective cohort	04/23 - 11/30	The United Kingdom	8.63%	Moderate
Healthcare Practitioners and Technical Occupations (29-0000)	Nishida et al., 2020[90]	n=140	Medical and Clinical Laboratory Technicians	Cross-sectional survey	06/12 - 06/19	Japan	0%	Moderate
Healthcare Practitioners and Technical Occupations (29-0000)	Rosser et al., 2020[33]	n=225	Medical and Clinical Laboratory Technicians	Cross-sectional survey	04/20 - 05/20	United States of America	0.44%	High
Healthcare Practitioners and Technical Occupations (29-0000)	Iversen et al., 2020[8]	n=342	Radiologic Technologists	Cross-sectional survey	04/15 - 04/22	Denmark	3.51%	Low

For peer review only

Healthcare Practitioners and Technical Occupations (29-0000)	Martin et al., 2020[23]	n=241	Radiologic Technologists	Cross-sectional survey	05/29 - 07/13	The United Kingdom	9.96%	Moderate
Healthcare Practitioners and Technical Occupations (29-0000)	Akinbami et al., 2020[46]	n=1158	Emergency Medical Technicians and Paramedics	Cross-sectional survey	05/18 - 06/13	United States of America	5.2% (4-6.6%)	Moderate
Healthcare Practitioners and Technical Occupations (29-0000)	Buntinx et al., 2020[167]	n=10	Emergency Medical Technicians and Paramedics	Cross-sectional survey	04/14 - 04/16	Belgium	10%	High
Healthcare Practitioners and Technical Occupations (29-0000)	Haq et al., 2020[67]	n=157	Emergency Medical Technicians and Paramedics	Cross-sectional survey	06/15 - 06/29	Pakistan	42% (34.2-50.1%)	Moderate
Healthcare Practitioners and Technical Occupations (29-0000)	Iversen et al., 2020[8]	n=323	Emergency Medical Technicians and Paramedics	Cross-sectional survey	04/15 - 04/22	Denmark	4.95%	Low
Healthcare Practitioners and Technical Occupations (29-0000)	Mesnil et al., 2020[143]	n=212	Emergency Medical Technicians and Paramedics	Cross-sectional survey	06/08 - 06/22	France	11%	High
Healthcare Practitioners and Technical Occupations (29-0000)	Reuben et al., 2020[168]	n=10	Emergency Medical Technicians and Paramedics	Cross-sectional survey	05/28 - 07/15	United States of America	0%	High

For peer review only

Healthcare Practitioners and Technical Occupations (29-0000)	Saberian et al., 2020[169]	n=243	Emergency Medical Technicians and Paramedics	Cross-sectional survey	03/20 - 05/20	Iran (Islamic Republic of)	41.56%	High
Healthcare Practitioners and Technical Occupations (29-0000)	Self et al., 2020[154]	n=56	Emergency Medical Technicians and Paramedics	Cross-sectional survey	04/03 - 06/19	United States of America	5.36%	Moderate
Healthcare Practitioners and Technical Occupations (29-0000)	Tarabichi et al., 2020[170]	n=111	Emergency Medical Technicians and Paramedics	Cross-sectional survey	04/20 - 05/19	United States of America	5.41%	High
Healthcare Practitioners and Technical Occupations (29-0000)	Baracco et al., 2020[24]	n=188	Health Technologists and Technicians, All Other	Cross-sectional survey	04/23 - 05/05	Italy	13.8%	High
Healthcare Practitioners and Technical Occupations (29-0000)	Chau et al., 2020[126]	n=22	Health Technologists and Technicians, All Other	Cross-sectional survey	08/23 - 08/30	Viet Nam	0%	High
Healthcare Practitioners and Technical Occupations (29-0000)	Goenka et al., 2020[25]	n=99	Health Technologists and Technicians, All Other	Cross-sectional survey	07/12 - 08/23	India	12.12%	Moderate
Healthcare Practitioners and Technical Occupations (29-0000)	Goenka et al., 2020[26]	n=16	Health Technologists and Technicians, All Other	Cross-sectional survey	08/01 - 08/31	India	68.75%	High
Healthcare Support	Jeremias et al., 2020[70]	n=155	Healthcare Support Occupations	Cross-sectional survey	03/01 - 04/30	United States of America	5.8%	High

Occupations (31-0000)								
Healthcare Support Occupations (31-0000)	Ward et al., 2020[113]	n=979	Nursing, Psychiatric, and Home Health Aides	Cross-sectional survey	09/15 - 09/28	The United Kingdom	11.09% (8.96-13.59%)	Moderate
Healthcare Support Occupations (31-0000)	Ward et al., 2020[113]	n=257	Nursing, Psychiatric, and Home Health Aides	Cross-sectional survey	09/15 - 09/28	The United Kingdom	8.95%	Moderate
Healthcare Support Occupations (31-0000)	Vijh et al., 2020[171]	n=169	Nursing, Psychiatric, and Home Health Aides	Cross-sectional survey	05/04 - 05/14	Canada	26.63%	High
Healthcare Support Occupations (31-0000)	Akinbami et al., 2020[46]	n=641	Nursing Assistants	Cross-sectional survey	05/18 - 06/13	United States of America	12.8% (10.3-15.6%)	Moderate
Healthcare Support Occupations (31-0000)	Bampoe et al., 2020[156]	n=108	Nursing Assistants	Cross-sectional survey	05/11 - 06/05	The United Kingdom	15.7% (9.5-24%)	High
Healthcare Support Occupations (31-0000)	Baracco et al., 2020[24]	n=257	Nursing Assistants	Cross-sectional survey	04/23 - 05/05	Italy	22.2%	High
Healthcare Support Occupations (31-0000)	Barallat et al., 2020[50]	n=832	Nursing Assistants	Cross-sectional survey	05/04 - 05/22	Spain	13.94%	High
Healthcare Support Occupations (31-0000)	Bhattacharya et al., 2020[172]	n=121	Nursing Assistants	Cross-sectional survey	06/01 - 06/15	United States of America	1.65%	High
Healthcare Support	Brousseau et al., 2020[134]	n=132	Nursing Assistants	Cross-sectional survey	07/06 - 09/24	Canada	16.7%	High

For peer review only

Occupations (31-0000)								
Healthcare Support Occupations (31-0000)	Brunner et al., 2020[54]	n=95	Nursing Assistants	Cross-sectional survey	05/04 - 05/29	United States of America	1.05%	High
Healthcare Support Occupations (31-0000)	Brzostek et al., 2020[151]	n=570	Nursing Assistants	Cross-sectional survey	04/17 - 05/07	United States of America	39.5%	Moderate
Healthcare Support Occupations (31-0000)	Brzostek et al., 2020[151]	n=263	Nursing Assistants	Cross-sectional survey	04/17 - 05/07	United States of America	45.6%	Moderate
Healthcare Support Occupations (31-0000)	Calcagno et al., 2020[124]	n=476	Nursing Assistants	Cross-sectional survey	04/17 - 05/20	Italy	9.24%	Moderate
Healthcare Support Occupations (31-0000)	Costa et al., 2020[128]	n=553	Nursing Assistants	Cross-sectional survey	05/14 - 05/28	Brazil	10.5%	Moderate
Healthcare Support Occupations (31-0000)	Galan et al., 2020[20]	n=472	Nursing Assistants	Cross-sectional survey	04/14 - 04/27	Spain	33.26%	High
Healthcare Support Occupations (31-0000)	Garcia et al., 2020[173]	n=2424	Nursing Assistants	Cross-sectional survey	05/01 - 05/30	Spain	22.4%	High
Healthcare Support Occupations (31-0000)	Garcia et al., 2020[174]	n=2424	Nursing Assistants	Cross-sectional survey	05/01 - 05/30	Spain	22.4%	High
Healthcare Support	Hanrath et al., 2020[32]	n=1434	Nursing Assistants	Cross-sectional survey	05/29 - 07/06	The United Kingdom	11.44%	High

Occupations (31-0000)								
Healthcare Support Occupations (31-0000)	Iversen et al., 2020[8]	n=501	Nursing Assistants	Cross-sectional survey	04/15 - 04/22	Denmark	1.2%	Low
Healthcare Support Occupations (31-0000)	Khan et al., 2020[127]	n=624	Nursing Assistants	Cross-sectional survey	06/15 - 06/29	India	2.4% (1.5-4%)	Moderate
Healthcare Support Occupations (31-0000)	Mughal et al., 2020[175]	n=121	Nursing Assistants	Cross-sectional survey	05/14 - 05/19	United States of America	0.83%	High
Healthcare Support Occupations (31-0000)	Rao et al., 2020[176]	n=1000	Nursing Assistants	Cross-sectional survey	05/23 - 06/06	India	1%	Unclear
Healthcare Support Occupations (31-0000)	Rudberg et al., 2020[147]	n=428	Nursing Assistants	Cross-sectional survey	04/14 - 05/08	Sweden	25.5%	Moderate
Healthcare Support Occupations (31-0000)	Siddiqui et al., 2020[2]	n=28	Nursing Assistants	Prospective cohort	04/15 - 08/15	India	10.7%	High
Healthcare Support Occupations (31-0000)	Yogo et al., 2020[36]	n=154	Nursing Assistants	Cross-sectional survey	05/20 - 06/08	United States of America	3.24%	High
Healthcare Support Occupations (31-0000)	Brousseau et al., 2020[134]	n=201	Orderlies	Cross-sectional survey	07/06 - 09/24	Canada	17.9%	High
Healthcare Support	Kassem et al., 2020[72]	n=9	Orderlies	Cross-sectional survey	06/01 - 06/14	Egypt	0%	High

For peer review only

Occupations (31-0000)								
Healthcare Support Occupations (31-0000)	Kassem et al., 2020[72]	n=9	Orderlies	Cross-sectional survey	06/01 - 06/14	Egypt	33.33%	High
Healthcare Support Occupations (31-0000)	Kassem et al., 2020[72]	n=9	Orderlies	Cross-sectional survey	06/01 - 06/14	Egypt	11.11%	High
Healthcare Support Occupations (31-0000)	Kassem et al., 2020[72]	n=9	Orderlies	Cross-sectional survey	06/01 - 06/14	Egypt	22.22%	High
Healthcare Support Occupations (31-0000)	Hanrath et al., 2020[32]	n=122	Orderlies	Cross-sectional survey	05/29 - 07/06	The United Kingdom	9.02%	High
Healthcare Support Occupations (31-0000)	Lumley et al., 2020[9]	n=377	Orderlies	Prospective cohort	04/23 - 11/30	The United Kingdom	15.38%	Moderate
Healthcare Support Occupations (31-0000)	Rosser et al., 2020[33]	n=3959	Medical Assistants	Cross-sectional survey	04/20 - 05/20	United States of America	1.39%	High
Healthcare Support Occupations (31-0000)	Yogo et al., 2020[36]	n=106	Phlebotomists	Cross-sectional survey	05/20 - 06/08	United States of America	0%	High
Healthcare Support Occupations (31-0000)	Cavlek et al., 2020[56]	n=300	Healthcare Support Workers, All Other	Cross-sectional survey	04/25 - 05/24	Croatia	0.67%	High
Healthcare Support	Erber et al., 2020[31]	n=383	Healthcare Support Workers, All Other	Cross-sectional survey	04/14 - 05/29	Germany	2.34%	High

For peer review only

Occupations (31-0000)								
Healthcare Support Occupations (31-0000)	Khan et al., 2020[127]	n=141	Healthcare Support Workers, All Other	Cross-sectional survey	06/15 - 06/29	India	0%	Moderate
Protective Service Occupations (33-0000)	Shukla et al., 2020[177]	n=1713	Protective Service Occupations	Cross-sectional survey	04/24 - 05/21	United States of America	1.46%	Moderate
Protective Service Occupations (33-0000)	Martinez et al., 2020[121]	n=18	First-Line Supervisors of Fire Fighting and Prevention Workers	Cross-sectional survey	04/16 - 04/17	United States of America	0%	High
Protective Service Occupations (33-0000)	Martinez et al., 2020[121]	n=47	First-Line Supervisors of Fire Fighting and Prevention Workers	Cross-sectional survey	04/16 - 04/17	United States of America	14.89%	High
Protective Service Occupations (33-0000)	Martinez et al., 2020[121]	n=13	First-Line Supervisors of Fire Fighting and Prevention Workers	Cross-sectional survey	04/16 - 04/17	United States of America	7.69%	High
Protective Service Occupations (33-0000)	Akinbami et al., 2020[46]	n=330	Firefighters	Cross-sectional survey	05/18 - 06/13	United States of America	6.7% (4.2-9.9%)	Moderate
Protective Service Occupations (33-0000)	Gray et al., 2020[178]	n=132	Firefighters	Cross-sectional survey	05/01 - 05/31	United States of America	14%	High
Protective Service Occupations (33-0000)	Reuben et al., 2020[168]	n=62	Firefighters	Cross-sectional survey	05/28 - 07/15	United States of America	4.84%	High
Protective Service Occupations (33-0000)	Sabourin et al., 2020[35]	n=42	Firefighters	Cross-sectional survey	07/15 - 08/15	United States of America	2.38%	High
Protective Service Occupations (33-0000)	Tarabichi et al., 2020[170]	n=185	Firefighters	Cross-sectional survey	04/20 - 05/19	United States of America	5.41%	High

For peer review only

Protective Service Occupations (33-0000)	Martinez et al., 2020[121]	n=7	Fire Inspectors and Investigators	Cross-sectional survey	04/16 - 04/17	United States of America	14.29%	High
Protective Service Occupations (33-0000)	Akinbami et al., 2020[46]	n=785	Police and Sheriff's Patrol Officers	Cross-sectional survey	05/18 - 06/13	United States of America	4% (2.7-5.6%)	Moderate
Protective Service Occupations (33-0000)	Chughtai et al., 2020[179]	n=154	Police and Sheriff's Patrol Officers	Cross-sectional survey	05/20 - 05/30	Pakistan	15.6%	High
Protective Service Occupations (33-0000)	Gudo et al., 2020[65]	n=564	Police and Sheriff's Patrol Officers	Cross-sectional survey	06/17 - 06/30	Mozambique	6% (4-8%)	High
Protective Service Occupations (33-0000)	Gujski et al., 2020[180]	n=4026	Police and Sheriff's Patrol Officers	Cross-sectional survey	06/22 - 07/08	Poland	4.2%	Moderate
Protective Service Occupations (33-0000)	Halatoko et al., 2020[41]	n=196	Police and Sheriff's Patrol Officers	Cross-sectional survey	04/23 - 05/08	Togo	0%	High
Protective Service Occupations (33-0000)	Langa et al., 2020[181]	n=471	Police and Sheriff's Patrol Officers	Cross-sectional survey	09/28 - 10/09	Mozambique	1.5%	High
Protective Service Occupations (33-0000)	Macicame et al., 2020[182]	n=456	Police and Sheriff's Patrol Officers	Cross-sectional survey	09/14 - 09/30	Mozambique	4.39%	High
Protective Service Occupations (33-0000)	Mahomed et al., 2020[81]	n=554	Police and Sheriff's Patrol Officers	Cross-sectional survey	08/31 - 10/12	Mozambique	2.9%	High
Protective Service Occupations (33-0000)	Reuben et al., 2020[168]	n=220	Police and Sheriff's Patrol Officers	Cross-sectional survey	05/28 - 07/15	United States of America	3.64%	High
Protective Service Occupations (33-0000)	Sabourin et al., 2020[35]	n=125	Police and Sheriff's Patrol Officers	Cross-sectional survey	07/15 - 08/15	United States of America	4%	High

For peer review only

Protective Service Occupations (33-0000)	Shukla et al., 2020[177]	n=1643	Police and Sheriff's Patrol Officers	Cross-sectional survey	04/24 - 05/21	United States of America	1.52%	Moderate
Protective Service Occupations (33-0000)	Siddiqui et al., 2020[2]	n=27	Police and Sheriff's Patrol Officers	Prospective cohort	04/15 - 08/15	India	7.4%	High
Protective Service Occupations (33-0000)	Viegas et al., 2020[110]	n=559	Police and Sheriff's Patrol Officers	Cross-sectional survey	08/03 - 08/21	Mozambique	3.94%	High
Protective Service Occupations (33-0000)	Denyer et al., 2020[60]	n=38216	Security Guards	Cross-sectional survey	05/12 - 05/18	Japan	0.23%	Unclear
Protective Service Occupations (33-0000)	Mahumane et al., 2020[82]	n=407	Security Guards	Cross-sectional survey	11/02 - 11/17	Mozambique	4.9%	High
Protective Service Occupations (33-0000)	Siddiqui et al., 2020[2]	n=9	Security Guards	Prospective cohort	04/15 - 08/15	India	0%	High
Protective Service Occupations (33-0000)	Silva et al., 2020[34]	n=32	Security Guards	Cross-sectional survey	06/05 - 07/31	Brazil	34%	High
Protective Service Occupations (33-0000)	Thani et al., 2020[183]	n=61	Security Guards	Cross-sectional survey	07/26 - 09/09	Qatar	60.1%	Moderate
Food Preparation and Serving Related Occupations (35-0000)	Thani et al., 2020[183]	n=93	Food Preparation and Serving Related Occupations	Cross-sectional survey	07/26 - 09/09	Qatar	29.2%	Moderate
Food Preparation and Serving Related Occupations (35-0000)	Siddiqui et al., 2020[2]	n=8	Cooks, All Other	Prospective cohort	04/15 - 08/15	India	37.5%	High
Food Preparation and Serving	Brunner et al., 2020[54]	n=8	Food Preparation Workers	Cross-sectional survey	05/04 - 05/29	United States of America	0%	High

For peer review only

Related Occupations (35-0000)								
Healthcare Support Occupations (31-0000)	Rosser et al., 2020[33]	n=335	Healthcare Support Occupations	Cross-sectional survey	04/20 - 05/20	United States of America	3.58%	High
Food Preparation and Serving Related Occupations (35-0000)	Biggs et al., 2020[3]	n=24	Food Servers, Nonrestaurant	Cross-sectional survey	04/28 - 05/03	United States of America	4.17%	Moderate
Food Preparation and Serving Related Occupations (35-0000)	Leidner et al., 2020[22]	n=113	Food Servers, Nonrestaurant	Cross sectional study with prospective cohort follow up of a subset of the sample	04/08 - 05/22	United States of America	1.77%	High
Food Preparation and Serving Related Occupations (35-0000)	Hanrath et al., 2020[32]	n=340	Other Food Preparation and Serving Related Workers	Cross-sectional survey	05/29 - 07/06	The United Kingdom	8.53%	High
Building and Grounds Cleaning and Maintenance Occupations (37-0000)	Martin et al., 2020[23]	n=528	Building and Grounds Cleaning and Maintenance Occupations	Cross-sectional survey	05/29 - 07/13	The United Kingdom	8.14%	Moderate
Building and Grounds Cleaning and Maintenance Occupations (37-0000)	Brousseau et al., 2020[134]	n=102	Building Cleaning and Pest Control Workers	Cross-sectional survey	07/06 - 09/24	Canada	10.8%	High
Building and Grounds Cleaning and Maintenance	Chau et al., 2020[126]	n=42	Building Cleaning and Pest Control Workers	Cross-sectional survey	08/23 - 08/30	Viet Nam	0%	High

For peer review only

Occupations (37-0000)								
Building and Grounds Cleaning and Maintenance Occupations (37-0000)	Finkenzeller et al., 2020[158]	n=57	Building Cleaning and Pest Control Workers	Prospective cohort	06/29 - 07/29	Germany	19.3%	Moderate
Building and Grounds Cleaning and Maintenance Occupations (37-0000)	Chau et al., 2020[126]	n=6	Janitors and Cleaners, Except Maids and Housekeeping Cleaners	Cross-sectional survey	08/23 - 08/30	Viet Nam	0%	High
Building and Grounds Cleaning and Maintenance Occupations (37-0000)	Epstude et al., 2020[184]	n=45	Janitors and Cleaners, Except Maids and Housekeeping Cleaners	Cross-sectional survey	06/15 - 06/30	Germany	0%	High
Building and Grounds Cleaning and Maintenance Occupations (37-0000)	Thani et al., 2020[183]	n=105	Janitors and Cleaners, Except Maids and Housekeeping Cleaners	Cross-sectional survey	07/26 - 09/09	Qatar	54.5%	Moderate
Building and Grounds Cleaning and Maintenance Occupations (37-0000)	Brunner et al., 2020[54]	n=23	Maids and Housekeeping Cleaners	Cross-sectional survey	05/04 - 05/29	United States of America	0%	High
Building and Grounds Cleaning and Maintenance Occupations (37-0000)	Goenka et al., 2020[25]	n=226	Maids and Housekeeping Cleaners	Cross-sectional survey	07/12 - 08/23	India	26.11%	Moderate
Building and Grounds Cleaning and Maintenance Occupations (37-0000)	Goenka et al., 2020[26]	n=10	Maids and Housekeeping Cleaners	Cross-sectional survey	08/01 - 08/31	India	10%	High

For peer review only

Building and Grounds Cleaning and Maintenance Occupations (37-0000)	Hanrath et al., 2020[32]	n=515	Maids and Housekeeping Cleaners	Cross-sectional survey	05/29 - 07/06	The United Kingdom	13.2%	High
Building and Grounds Cleaning and Maintenance Occupations (37-0000)	Khan et al., 2020[127]	n=276	Maids and Housekeeping Cleaners	Cross-sectional survey	06/15 - 06/29	India	3.3% (1.7-6.2%)	Moderate
Building and Grounds Cleaning and Maintenance Occupations (37-0000)	Leidner et al., 2020[22]	n=137	Maids and Housekeeping Cleaners	Cross sectional study with prospective cohort follow up of a subset of the sample	04/08 - 05/22	United States of America	8.03%	High
Building and Grounds Cleaning and Maintenance Occupations (37-0000)	Moscola et al., 2020[89]	n=7314	Maids and Housekeeping Cleaners	Cross-sectional survey	04/20 - 06/23	United States of America	20.9%	High
Building and Grounds Cleaning and Maintenance Occupations (37-0000)	Shakiba et al., 2020[10]	n=159	Maids and Housekeeping Cleaners	Cross-sectional survey	04/11 - 04/19	Iran (Islamic Republic of)	25% (13.6-37.5%)	Moderate
Building and Grounds Cleaning and Maintenance Occupations (37-0000)	Shields et al., 2020[97]	n=29	Maids and Housekeeping Cleaners	Cross-sectional survey	04/24 - 04/25	The United Kingdom	34.5%	High
Building and Grounds Cleaning and Maintenance Occupations (37-0000)	Siddiqui et al., 2020[2]	n=46	Maids and Housekeeping Cleaners	Prospective cohort	04/15 - 08/15	India	21.7%	High

For peer review only

Personal Care and Service Occupations (39-0000)	Biggs et al., 2020[3]	n=10	Hairdressers, Hairstylists, and Cosmetologists	Cross-sectional survey	04/28 - 05/03	United States of America	10%	Moderate
Personal Care and Service Occupations (39-0000)	Biggs et al., 2020[3]	n=48	Childcare Workers	Cross-sectional survey	04/28 - 05/03	United States of America	0%	Moderate
Personal Care and Service Occupations (39-0000)	Chen et al., 2020[135]	n=11	Personal Care Aides	Cross-sectional survey	02/19 - 02/19	China	9.09%	High
Personal Care and Service Occupations (39-0000)	Galan et al., 2020[20]	n=337	Personal Care Aides	Cross-sectional survey	04/14 - 04/27	Spain	27.89%	High
Personal Care and Service Occupations (39-0000)	Galan et al., 2020[20]	n=168	Personal Care Aides	Cross-sectional survey	04/14 - 04/27	Spain	27.38%	High
Personal Care and Service Occupations (39-0000)	Godbout et al., 2020[138]	n=86	Personal Care Aides	Cross-sectional survey	07/27 - 10/02	United States of America	2.32%	High
Personal Care and Service Occupations (39-0000)	Hassan et al., 2020[185]	n=403	Personal Care Aides	Cross-sectional survey	05/11 - 06/17	Sweden	20.1%	High
Personal Care and Service Occupations (39-0000)	Kumar et al., 2020[142]	n=292	Personal Care Aides	Cross-sectional survey	06/01 - 06/30	India	18.5% (14.5-23.3%)	High
Personal Care and Service Occupations (39-0000)	Ladhani et al., 2020[186]	n=208	Personal Care Aides	Prospective cohort	04/10 - 04/13	The United Kingdom	75% (68.7-80.4%)	High

For peer review only

Personal Care and Service Occupations (39-0000)	Lindahl et al., 2020[187]	n=1005	Personal Care Aides	Cross-sectional survey	04/01 - 04/20	Sweden	22.9% (20.4-25.7%)	High
Personal Care and Service Occupations (39-0000)	Regan et al., 2020[188]	n=305	Personal Care Aides	Cross-sectional survey	04/15 - 05/06	United States of America	23.6%	Unclear
Personal Care and Service Occupations (39-0000)	Siddiqui et al., 2020[2]	n=5	Personal Care Aides	Prospective cohort	04/15 - 08/15	India	40%	High
Personal Care and Service Occupations (39-0000)	Venugopal et al., 2020[150]	n=72	Personal Care Aides	Cross-sectional survey	03/01 - 05/01	United States of America	28%	Moderate
Personal Care and Service Occupations (39-0000)	Viegas et al., 2020[110]	n=85	Personal Care Aides	Cross-sectional survey	08/03 - 08/21	Mozambique	1.18%	High
Sales and Related Occupations (41-0000)	Arnaldo et al., 2020[13]	n=928	Sales and Related Occupations	Cross-sectional survey	07/06 - 07/13	Mozambique	6.5%	High
Sales and Related Occupations (41-0000)	Arnaldo et al., 2020[48]	n=1123	Sales and Related Occupations	Cross-sectional survey	08/10 - 08/21	Mozambique	1.6%	High
Sales and Related Occupations (41-0000)	Langa et al., 2020[181]	n=871	Sales and Related Occupations	Cross-sectional survey	09/28 - 10/09	Mozambique	0.2%	High
Sales and Related Occupations (41-0000)	Mabunda et al., 2020[15]	n=1585	Sales and Related Occupations	Cross-sectional survey	09/21 - 10/02	Mozambique	8.3%	High
Sales and Related Occupations (41-0000)	Macicame et al., 2020[182]	n=1288	Sales and Related Occupations	Cross-sectional survey	09/14 - 09/30	Mozambique	4.97%	High

For peer review only

Sales and Related Occupations (41-0000)	Mahomed et al., 2020[81]	n=1556	Sales and Related Occupations	Cross-sectional survey	08/31 - 10/12	Mozambique	0.8%	High
Sales and Related Occupations (41-0000)	Mahumane et al., 2020[82]	n=643	Sales and Related Occupations	Cross-sectional survey	11/02 - 11/17	Mozambique	1.9%	High
Sales and Related Occupations (41-0000)	Arnaldo et al., 2020[14]	n=472	Sales and Related Occupations	Cross-sectional survey	11/16 - 11/21	Mozambique	6.8%	High
Sales and Related Occupations (41-0000)	Arnaldo et al., 2020[14]	n=460	Sales and Related Occupations	Cross-sectional survey	11/02 - 11/12	Mozambique	5.9%	High
Sales and Related Occupations (41-0000)	Mahomed et al., 2020[16]	n=517	Sales and Related Occupations	Cross-sectional survey	11/26 - 12/03	Mozambique	8.9%	High
Sales and Related Occupations (41-0000)	Mahomed et al., 2020[16]	n=1001	Sales and Related Occupations	Cross-sectional survey	11/07 - 11/21	Mozambique	4.5%	High
Sales and Related Occupations (41-0000)	Biggs et al., 2020[3]	n=19	Retail Sales Workers	Cross-sectional survey	04/28 - 05/03	United States of America	0%	Moderate
Sales and Related Occupations (41-0000)	Poustchi et al., 2020[28]	n=753	Cashiers	Cross-sectional survey	04/17 - 06/02	Iran (Islamic Republic of)	16.1% (12.9-19.2%)	Moderate
Sales and Related Occupations (41-0000)	Alali et al., 2020[189]	n=525	Cashiers	Cross-sectional survey	05/23 - 06/26	Kuwait	38.1% (34-42.3%)	High
Sales and Related Occupations (41-0000)	Denyer et al., 2020[60]	n=19075	Retail Salespersons	Cross-sectional survey	05/12 - 05/18	Japan	0.04%	Unclear
Sales and Related Occupations (41-0000)	Kern et al., 2020[73]	n=300	Retail Salespersons	Cross-sectional survey	04/09 - 04/16	Germany	0.33% (0.01-1.84%)	High

For peer review only

Sales and Related Occupations (41-0000)	Khan et al., 2020[45]	n=132	Retail Salespersons	Cross-sectional survey	07/01 - 07/15	India	5.3% (2.5-10.7%)	Moderate
Sales and Related Occupations (41-0000)	Thani et al., 2020[183]	n=171	Retail Salespersons	Cross-sectional survey	07/26 - 09/09	Qatar	40.3%	Moderate
Sales and Related Occupations (41-0000)	Siddiqui et al., 2020[2]	n=4	Sales Representatives, Wholesale and Manufacturing, Except Technical and Scientific Products	Prospective cohort	04/15 - 08/15	India	25%	High
Sales and Related Occupations (41-0000)	Biggs et al., 2020[3]	n=34	Real Estate Sales Agents	Cross-sectional survey	04/28 - 05/03	United States of America	0%	Moderate
Sales and Related Occupations (41-0000)	Gudo et al., 2020[65]	n=1493	Door-to-Door Sales Workers, News and Street Vendors, and Related Workers	Cross-sectional survey	06/17 - 06/30	Mozambique	10% (8-11%)	High
Sales and Related Occupations (41-0000)	Viegas et al., 2020[110]	n=1246	Door-to-Door Sales Workers, News and Street Vendors, and Related Workers	Cross-sectional survey	08/03 - 08/21	Mozambique	5.22%	High
Sales and Related Occupations (41-0000)	Shakiba et al., 2020[10]	n=46	Sales and Related Workers, All Other	Cross-sectional survey	04/11 - 04/19	Iran (Islamic Republic of)	8.7% (0.8-20%)	Moderate
Office and Administrative Support Occupations (43-0000)	Calcagno et al., 2020[124]	n=539	Office and Administrative Support Occupations	Cross-sectional survey	04/17 - 05/20	Italy	3.34%	Moderate
Office and Administrative Support Occupations (43-0000)	Costa et al., 2020[128]	n=120	Office and Administrative Support Occupations	Cross-sectional survey	05/14 - 05/28	Brazil	14.2%	Moderate

For peer review only

Office and Administrative Support Occupations (43-0000)	Rosser et al., 2020[33]	n=972	Office and Administrative Support Occupations	Cross-sectional survey	04/20 - 05/20	United States of America	1.34%	High
Office and Administrative Support Occupations (43-0000)	Tsitsilonis et al., 2020[12]	n=504	Office and Administrative Support Occupations	Cross-sectional survey	06/15 - 07/15	Greece	0.48% (0-2.37%)	Moderate
Office and Administrative Support Occupations (43-0000)	Khan et al., 2020[45]	n=37	Hotel, Motel, and Resort Desk Clerks	Cross-sectional survey	07/01 - 07/15	India	10.8% (4.1-25.5%)	Moderate
Office and Administrative Support Occupations (43-0000)	Brunner et al., 2020[54]	n=26	Receptionists and Information Clerks	Cross-sectional survey	05/04 - 05/29	United States of America	0%	High
Office and Administrative Support Occupations (43-0000)	Favara et al., 2020[136]	n=10	Receptionists and Information Clerks	Prospective cohort	06/01 - 06/07	The United Kingdom	0%	High
Office and Administrative Support Occupations (43-0000)	Moscola et al., 2020[89]	n=9645	Receptionists and Information Clerks	Cross-sectional survey	04/20 - 06/23	United States of America	12.6%	High
Office and Administrative Support Occupations (43-0000)	Biggs et al., 2020[3]	n=11	Shipping, Receiving, and Traffic Clerks	Cross-sectional survey	04/28 - 05/03	United States of America	18.18%	Moderate
Office and Administrative	Silva et al., 2020[34]	n=82	Stock Clerks and Order Fillers	Cross-sectional survey	06/05 - 07/31	Brazil	4.88%	High

For peer review only

Support Occupations (43-0000)								
Office and Administrative Support Occupations (43-0000)	Khan et al., 2020[45]	n=186	Secretaries and Administrative Assistants	Cross-sectional survey	07/01 - 07/15	India	3.8% (1.8-7.7%)	Moderate
Office and Administrative Support Occupations (43-0000)	Alemu et al., 2020[6]	n=48	Executive Secretaries and Executive Administrative Assistants	Cross-sectional survey	04/23 - 04/28	Ethiopia	2.1%	Moderate
Office and Administrative Support Occupations (43-0000)	Barallat et al., 2020[50]	n=1181	Executive Secretaries and Executive Administrative Assistants	Cross-sectional survey	05/04 - 05/22	Spain	6.52%	High
Office and Administrative Support Occupations (43-0000)	Lumley et al., 2020[9]	n=1557	Executive Secretaries and Executive Administrative Assistants	Prospective cohort	04/23 - 11/30	The United Kingdom	6.74%	Moderate
Office and Administrative Support Occupations (43-0000)	Reuben et al., 2020[168]	n=18	Executive Secretaries and Executive Administrative Assistants	Cross-sectional survey	05/28 - 07/15	United States of America	0%	High
Office and Administrative Support Occupations (43-0000)	Akinbami et al., 2020[46]	n=964	Medical Secretaries	Cross-sectional survey	05/18 - 06/13	United States of America	8% (6.4-9.9%)	Moderate
Office and Administrative Support	Alharbi et al., 2020[125]	n=8	Medical Secretaries	Cross-sectional survey	04/18 - 06/17	Saudi Arabia	25%	High

For peer review only

Occupations (43-0000)								
Office and Administrative Support Occupations (43-0000)	Dimcheff et al., 2020[157]	n=357	Medical Secretaries	Cross-sectional survey	06/08 - 07/08	United States of America	4.2%	Moderate
Office and Administrative Support Occupations (43-0000)	Erber et al., 2020[31]	n=557	Medical Secretaries	Cross-sectional survey	04/14 - 05/29	Germany	3.78%	High
Office and Administrative Support Occupations (43-0000)	Finkenzeller et al., 2020[158]	n=240	Medical Secretaries	Prospective cohort	06/29 - 07/29	Germany	7.1%	Moderate
Office and Administrative Support Occupations (43-0000)	Goenka et al., 2020[25]	n=75	Medical Secretaries	Cross-sectional survey	07/12 - 08/23	India	8%	Moderate
Office and Administrative Support Occupations (43-0000)	Goenka et al., 2020[25]	n=75	Medical Secretaries	Cross-sectional survey	07/12 - 08/23	India	8%	Moderate
Office and Administrative Support Occupations (43-0000)	Iversen et al., 2020[8]	n=2631	Medical Secretaries	Cross-sectional survey	04/15 - 04/22	Denmark	2.7%	Low
Office and Administrative Support Occupations (43-0000)	Leidner et al., 2020[22]	n=793	Medical Secretaries	Cross sectional study with prospective cohort follow up of a	04/08 - 05/22	United States of America	3.15%	High

For peer review only

				subset of the sample				
Office and Administrative Support Occupations (43-0000)	Mesnil et al., 2020[143]	n=184	Medical Secretaries	Cross-sectional survey	06/08 - 06/22	France	14.13%	High
Office and Administrative Support Occupations (43-0000)	Nishida et al., 2020[90]	n=98	Medical Secretaries	Cross-sectional survey	06/12 - 06/19	Japan	1% (0.18-5.6%)	Moderate
Office and Administrative Support Occupations (43-0000)	Noor et al., 2020[130]	n=91	Medical Secretaries	Cross-sectional survey	07/13 - 07/15	Pakistan	43.96%	Moderate
Office and Administrative Support Occupations (43-0000)	Thani et al., 2020[183]	n=82	Medical Secretaries	Cross-sectional survey	07/26 - 09/09	Qatar	31.6%	Moderate
Office and Administrative Support Occupations (43-0000)	Zhou et al., 2020[166]	n=505	Medical Secretaries	Cross-sectional survey	03/16 - 03/25	China	1.39%	Moderate
Office and Administrative Support Occupations (43-0000)	Chau et al., 2020[126]	n=20	Data Entry Keyers	Cross-sectional survey	08/23 - 08/30	Viet Nam	0%	High
Office and Administrative Support Occupations (43-0000)	Jones et al., 2020[29]	n=1233	Office Clerks, General	Cross-sectional survey	01/15 - 06/15	The United Kingdom	6.1%	High

For peer review only

Office and Administrative Support Occupations (43-0000)	Rosser et al., 2020[33]	n=218	Office Clerks, General	Cross-sectional survey	04/20 - 05/20	United States of America	0%	High
Office and Administrative Support Occupations (43-0000)	Satpati et al., 2020[27]	n=47	Office Clerks, General	Cross-sectional survey	07/26 - 08/08	India	4.26%	Moderate
Office and Administrative Support Occupations (43-0000)	Baracco et al., 2020[24]	n=194	Office and Administrative Support Workers, All Other	Cross-sectional survey	04/23 - 05/05	Italy	14.4%	High
Office and Administrative Support Occupations (43-0000)	Brzostek et al., 2020[151]	n=286	Office and Administrative Support Workers, All Other	Cross-sectional survey	04/17 - 05/07	United States of America	45.5%	Moderate
Office and Administrative Support Occupations (43-0000)	Kassem et al., 2020[72]	n=7	Office and Administrative Support Workers, All Other	Cross-sectional survey	06/01 - 06/14	Egypt	14.28%	High
Office and Administrative Support Occupations (43-0000)	Kassem et al., 2020[72]	n=7	Office and Administrative Support Workers, All Other	Cross-sectional survey	06/01 - 06/14	Egypt	0%	High
Office and Administrative Support Occupations (43-0000)	Kassem et al., 2020[72]	n=7	Office and Administrative Support Workers, All Other	Cross-sectional survey	06/01 - 06/14	Egypt	0%	High

For peer review only

Office and Administrative Support Occupations (43-0000)	Kassem et al., 2020[72]	n=7	Office and Administrative Support Workers, All Other	Cross-sectional survey	06/01 - 06/14	Egypt	14.28%	High
Farming, Fishing, and Forestry Occupations (45-0000)	Satpati et al., 2020[27]	n=53	Agricultural Workers	Cross-sectional survey	07/26 - 08/08	India	0%	Moderate
Farming, Fishing, and Forestry Occupations (45-0000)	Addetia et al., 2020[190]	n=120	Fishers and Related Fishing Workers	Retrospective cohort	05/01 - 05/31	United States of America	5%	High
Farming, Fishing, and Forestry Occupations (45-0000)	Arnaldo et al., 2020[13]	n=80	Fishers and Related Fishing Workers	Cross-sectional survey	07/06 - 07/13	Mozambique	5%	High
Construction and Extraction Occupations (47-0000)	Biggs et al., 2020[3]	n=42	Construction Trades Workers	Cross-sectional survey	04/28 - 05/03	United States of America	0%	Moderate
Installation, Maintenance, and Repair Occupations (49-0000)	Blairon et al., 2020[52]	n=134	Other Installation, Maintenance, and Repair Occupations	Cross-sectional survey	05/25 - 06/19	Belgium	16.4%	High
Production Occupations (51-0000)	Picon et al., 2020[191]	n=40	Butchers and Other Meat, Poultry, and Fish Processing Workers	Cross-sectional survey	06/13 - 06/17	Brazil	15%	Moderate
Production Occupations (51-0000)	Picon et al., 2020[191]	n=1087	Miscellaneous Food Processing Workers	Cross-sectional survey	06/13 - 06/17	Brazil	1.47%	Moderate
Production Occupations (51-0000)	Bontadi et al., 2020[192]	n=1267	Production Workers, All Other	Cross-sectional survey	04/11 - 04/29	Italy	1.58%	High

For peer review only

Production Occupations (51-0000)	Xu et al., 2020[193]	n=442	Production Workers, All Other	Cross-sectional survey	03/09 - 04/10	China	1.4% (0.6-2.9%)	High
Transportation and Material Moving Occupations (53-0000)	Arnaldo et al., 2020[13]	n=248	Transportation and Material Moving Occupations	Cross-sectional survey	07/06 - 07/13	Mozambique	4.8%	High
Transportation and Material Moving Occupations (53-0000)	Arnaldo et al., 2020[48]	n=367	Transportation and Material Moving Occupations	Cross-sectional survey	08/10 - 08/21	Mozambique	7.4%	High
Transportation and Material Moving Occupations (53-0000)	Arnaldo et al., 2020[14]	n=112	Transportation and Material Moving Occupations	Cross-sectional survey	11/16 - 11/21	Mozambique	16.1%	High
Transportation and Material Moving Occupations (53-0000)	Biggs et al., 2020[3]	n=14	Transportation and Material Moving Occupations	Cross-sectional survey	04/28 - 05/03	United States of America	0%	Moderate
Transportation and Material Moving Occupations (53-0000)	Gudo et al., 2020[65]	n=554	Transportation and Material Moving Occupations	Cross-sectional survey	06/17 - 06/30	Mozambique	3% (1-4%)	High
Transportation and Material Moving Occupations (53-0000)	Langa et al., 2020[181]	n=230	Transportation and Material Moving Occupations	Cross-sectional survey	09/28 - 10/09	Mozambique	0.4%	High
Transportation and Material Moving Occupations (53-0000)	Mabunda et al., 2020[15]	n=473	Transportation and Material Moving Occupations	Cross-sectional survey	09/21 - 10/02	Mozambique	8.7%	High
Transportation and Material Moving Occupations (53-0000)	Macicame et al., 2020[182]	n=282	Transportation and Material Moving Occupations	Cross-sectional survey	09/14 - 09/30	Mozambique	3.19%	High

For peer review only

Transportation and Material Moving Occupations (53-0000)	Mahomed et al., 2020[81]	n=334	Transportation and Material Moving Occupations	Cross-sectional survey	08/31 - 10/12	Mozambique	1.5%	High
Transportation and Material Moving Occupations (53-0000)	Mahumane et al., 2020[82]	n=287	Transportation and Material Moving Occupations	Cross-sectional survey	11/02 - 11/17	Mozambique	1%	High
Transportation and Material Moving Occupations (53-0000)	Thani et al., 2020[183]	n=435	Transportation and Material Moving Occupations	Cross-sectional survey	07/26 - 09/09	Qatar	53.4%	Moderate
Transportation and Material Moving Occupations (53-0000)	Halatoko et al., 2020[41]	n=212	Air Transportation Workers	Cross-sectional survey	04/23 - 05/08	Togo	0.9%	High
Transportation and Material Moving Occupations (53-0000)	Viegas et al., 2020[110]	n=623	Air Transportation Workers	Cross-sectional survey	08/03 - 08/21	Mozambique	2.25%	High
Transportation and Material Moving Occupations (53-0000)	Viegas et al., 2020[110]	n=362	Air Transportation Workers	Cross-sectional survey	08/03 - 08/21	Mozambique	3.31%	High
Transportation and Material Moving Occupations (53-0000)	Khan et al., 2020[127]	n=57	Ambulance Drivers and Attendants, Except Emergency Medical Technicians	Cross-sectional survey	06/15 - 06/29	India	3.5% (0.9-13.3%)	Moderate
Transportation and Material Moving Occupations (53-0000)	Martinez et al., 2020[121]	n=30	Heavy and Tractor-Trailer Truck Drivers	Cross-sectional survey	04/16 - 04/17	United States of America	16.67%	High
Transportation and Material Moving Occupations (53-0000)	Siddiqui et al., 2020[2]	n=9	Heavy and Tractor-Trailer Truck Drivers	Prospective cohort	04/15 - 08/15	India	11.1%	High

For peer review only

Transportation and Material Moving Occupations (53-0000)	Halatoko et al., 2020[41]	n=122	Taxi Drivers and Chauffeurs	Cross-sectional survey	04/23 - 05/08	Togo	0.8%	High
Transportation and Material Moving Occupations (53-0000)	Poustchi et al., 2020[28]	n=718	Taxi Drivers and Chauffeurs	Cross-sectional survey	04/17 - 06/02	Iran (Islamic Republic of)	14.1% (11.4-16.9%)	Moderate
Transportation and Material Moving Occupations (53-0000)	Alemu et al., 2020[6]	n=8	Parking Lot Attendants	Cross-sectional survey	04/23 - 04/28	Ethiopia	12.5%	Moderate
Transportation and Material Moving Occupations (53-0000)	Alemu et al., 2020[6]	n=110	Laborers and Freight, Stock, and Material Movers, Hand	Cross-sectional survey	04/23 - 04/28	Ethiopia	10%	Moderate
Transportation and Material Moving Occupations (53-0000)	Khan et al., 2020[45]	n=97	Laborers and Freight, Stock, and Material Movers, Hand	Cross-sectional survey	07/01 - 07/15	India	2.1% (0.5-7.9%)	Moderate
Transportation and Material Moving Occupations (53-0000)	Satpati et al., 2020[27]	n=63	Laborers and Freight, Stock, and Material Movers, Hand	Cross-sectional survey	07/26 - 08/08	India	12.7%	Moderate
Not employed (mixed)*	Carrat et al., 2020[4]	n=6295	Unemployed	Prospective cohort	05/04 - 06/23	France	4.9% (4.1-5.6%)	Moderate
Not employed (mixed)*	Carrat et al., 2020[4]	n=1457	Unemployed	Prospective cohort	05/04 - 06/23	France	8.3% (6.4-10%)	Moderate
Not employed (mixed)*	Carrat et al., 2020[4]	n=306	Unemployed	Prospective cohort	05/04 - 06/23	France	7.2% (2.3-11.1%)	Moderate
Not employed (mixed)*	Carrat et al., 2020[4]	n=125	Unemployed	Prospective cohort	05/04 - 06/23	France	3.8% (0.5-6.3%)	Moderate
Not employed (mixed)*	Carrat et al., 2020[4]	n=402	Unemployed	Prospective cohort	05/04 - 06/23	France	7.8% (4.7-10.4%)	Moderate

For peer review only

Not employed (mixed)*	Chamie et al., 2020[194]	n=230	Unemployed	Cross-sectional survey	04/25 - 04/28	United States of America	4.3%	Moderate
Not employed (mixed)*	McLaughlin et al., 2020[195]	n=241	Unemployed	Cross-sectional survey	05/04 - 05/19	United States of America	19.3% (14.6-24.5%)	Moderate
Not employed (mixed)*	Merkely et al., 2020[1]	n=1095	Unemployed	Cross-sectional survey	05/01 - 05/16	Hungary	0.43% (0.16-0.84%)	Moderate
Not employed (mixed)*	Munoz et al., 2020[196]	n=905	Unemployed	Cross-sectional survey	07/15 - 07/16	Argentina	20%	Moderate
Not employed (mixed)*	Richard et al., 2020[5]	n=549	Unemployed	Cross-sectional survey	04/06 - 06/30	Switzerland	6%	Low
Not employed (mixed)*	Satpati et al., 2020[27]	n=47	Unemployed	Cross-sectional survey	07/26 - 08/08	India	2.13%	Moderate
Not employed (mixed)*	Ward et al., 2020[113]	n=59369	Unemployed	Cross-sectional survey	09/15 - 09/28	The United Kingdom	3.35%	Moderate

1. Merkely B, Szabó AJ, Kosztin A, et al. Novel coronavirus epidemic in the Hungarian population, a cross-sectional nationwide survey to support the exit policy in Hungary. *GeroScience*. 2020;42(4):1063-1074. doi:10.1007/s11357-020-00226-9

2. Siddiqui S, Naushin S, Pradhan S, et al. SARS-CoV-2 antibody seroprevalence and stability in a tertiary care hospital-setting. *medRxiv*. Published online September 2020. doi:10.1101/2020.09.02.20186486

3. Biggs HM, Harris JB, Breakwell L, et al. Estimated Community Seroprevalence of SARS-CoV-2 Antibodies Two Georgia Counties, April 28May 3, 2020. *MMWR Morbidity and Mortality Weekly Report*. 2020;69(29):965-970. doi:10.15585/mmwr.mm6929e2

4. Carrat F, Lamballerie X de, Rahib D, et al. Seroprevalence of SARS-CoV-2 among adults in three regions of France following the lockdown and associated risk factors: A multicohort study. *medRxiv*. Published online September 2020:2020.09.16.20195693. doi:10.1101/2020.09.16.20195693

5. Richard A, Wisniak A, Perez-Saez J, et al. Seroprevalence of anti-SARS-CoV-2 IgG antibodies, risk factors for infection and associated symptoms in Geneva, Switzerland: A population-based study. *medRxiv*. Published online December 2020. doi:10.1101/2020.12.16.20248180

6. Alemu BN, Addissie A, Mamo G, et al. *Sero-Prevalence of Anti-SARS-CoV-2 Antibodies in Addis Ababa, Ethiopia*. Microbiology; 2020. doi:10.1101/2020.10.13.337287

7. Brehm T, Schwinge D, Lampalzer S, et al. Seroprevalence of SARS-CoV-2 antibodies among hospital workers in a German tertiary care center: A sequential follow-up study. *International Journal of Hygiene and Environmental Health*. 2021;232:113671. doi:10.1016/j.ijheh.2020.113671

8. Iversen K, Bundgaard H, Hasselbalch RB, et al. Risk of COVID-19 in health-care workers in Denmark: An observational cohort study. *The Lancet Infectious diseases*. Published online August 2020. doi:10.1016/S1473-3099(20)30589-2

For peer review only

9. Lumley SF, O’Donnell D, Stoesser NE, et al. Antibody Status and Incidence of SARS-CoV-2 Infection in Health Care Workers. *New England Journal of Medicine*. Published online December 2020:NEJMoa2034545. doi:10.1056/NEJMoa2034545

10. Shakiba M, Nazemipour M, Salari A, et al. Seroprevalence of SARS-CoV-2 in Guilan Province, Iran, April 2020. *Emerging Infectious Disease journal*. 2021;27(2). doi:10.3201/eid2702.201960

11. Tilley K, Ayvazyan V, Martinez L, et al. A Cross-Sectional Study Examining the Seroprevalence of Severe Acute Respiratory Syndrome Coronavirus 2 Antibodies in a University Student Population. *Journal of Adolescent Health*. 2020;67(6):763-768. doi:10.1016/j.jadohealth.2020.09.001

12. Tsitsilonis OE, Paraskevis D, Lianidou E, et al. Seroprevalence of Antibodies against SARS-CoV-2 among the Personnel and Students of the National and Kapodistrian University of Athens, Greece: A Preliminary Report. *Life*. 2020;10(9):214. doi:10.3390/life10090214

13. Paulo Arnaldo. *Inquérito Sero-Epidemiológico de SARS-CoV-2 Na Cidade de Pemba (InCOVID 2020)*. República de Moçambique Ministério da Saúde; 2020.

14. Paulo Arnaldo. *Inquérito Sero-Epidemiológico de SARS-CoV-2 Nas Cidades de Xai-Xai E Chókwè (InCOVID 2020)*. República de Moçambique Ministério da Saúde; 2020.

15. Nedio Mabunda. *Inquérito Sero-Epidemiológico de SARS-CoV-2 Na Cidade de Beira (InCOVID 2020)*. República de Moçambique Ministério da Saúde; 2020.

16. Mussagy Mahomed. *Inquérito Sero-Epidemiológico de SARS-CoV-2 Na Cidade de Maxixe E Vila de Massinga (InCOVID 2020)*. República de Moçambique Ministério da Saúde; 2020.

17. Payne DC, Smith-Jeffcoat SE, Nowak G, et al. SARS-CoV-2 Infections and Serologic Responses from a Sample of U.S. Navy Service Members - USS Theodore Roosevelt, April 2020. *MMWR Morbidity and mortality weekly report*. 2020;69(23):714-721. doi:10.15585/mmwr.mm6923e4

18. COVID-19 Serology Tests Still Show Low Antibody Rate of 0.07%. *KBS World Radio*.

19. Favara DM, McAdam K, Cooke A, et al. SARS-CoV-2 antigen and antibody prevalence among UK staff working with cancer patients during the COVID-19 pandemic. *medRxiv*. Published online September 2020:2020.09.18.20197590. doi:10.1101/2020.09.18.20197590

20. Galán MI, Velasco M, Casas ML, et al. Hospital-Wide SARS-CoV-2 seroprevalence in health care workers in a Spanish teaching hospital. *Enfermedades Infecciosas y Microbiología Clínica*. Published online December 2020:S0213005X20304183. doi:10.1016/j.eimc.2020.11.015

21. Hunter BR, Dbeibo L, Weaver CS, et al. Seroprevalence of severe acute respiratory coronavirus virus 2 (SARS-CoV-2) antibodies among healthcare workers with differing levels of coronavirus disease 2019 (COVID-19) patient exposure. *Infection Control & Hospital Epidemiology*. Published online August 2020:1-2. doi:10.1017/ice.2020.390

22. Leidner R, Frary A, Cramer J, et al. Longitudinal SARS-CoV-2 serosurveillance of over ten thousand health care workers in the Providence Oregon cohort. *medRxiv*. Published online August 2020:2020.08.16.20176107. doi:10.1101/2020.08.16.20176107

23. Martin CA, Patel P, Goss C, et al. Demographic and occupational determinants of anti-SARS-CoV-2 IgG seropositivity in hospital staff. *Journal of Public Health*. 2020;(fdaa199). doi:10.1093/pubmed/fdaa199

For peer review only

24. Baracco A, Perotti G, Filippin A, et al. *SARS-CoV-2 Antibody Prevalence in Health Care Workers of Lodi Hospital, the COVID-19 Italian Epicentre*. Social Science Research Network; 2020.

25. Goenka M, Afzalpurkar S, Goenka U, et al. Seroprevalence of COVID-19 Amongst Health Care Workers in a Tertiary Care Hospital of a Metropolitan City from India. *The Journal of the Association of Physicians of India*. 2020;68(11):14-19.

26. Goenka MK, Shah BB, Goenka U, et al. COVID-19 prevalence among health-care workers of Gastroenterology department: An audit from a tertiary-care hospital in India. *JGH Open*. 2021;5(1):56-63. doi:10.1002/jgh3.12447

27. Satpati P, Sarangi S, Gantait K, et al. *Sero-Surveillance (IgG) of SARS-CoV-2 Among Asymptomatic General Population of Paschim Medinipur, West Bengal, India*. Infectious Diseases (except HIV/AIDS); 2020. doi:10.1101/2020.09.12.20193219

28. Poustchi H, Darvishian M, Mohammadi Z, et al. SARS-CoV-2 antibody seroprevalence in the general population and high-risk occupational groups across 18 cities in Iran: A population-based cross-sectional study. *The Lancet Infectious Diseases*. 2020;0(0). doi:10.1016/S1473-3099(20)30858-6

29. Jones CR, Hamilton FW, Thompson A, Morris TT, Moran E. SARS-CoV-2 IgG seroprevalence in healthcare workers and other staff at North Bristol NHS Trust: A sociodemographic analysis. *Journal of Infection*. 2020;0(0). doi:10.1016/j.jinf.2020.11.036

30. Anna F, Goyard S, Lalanne AI, et al. High seroprevalence but short-lived immune response to SARS-CoV-2 infection in Paris. *medRxiv*. Published online November 2020:2020.10.25.20219030. doi:10.1101/2020.10.25.20219030

31. Erber J, Kappler V, Haller B, et al. Strategies for infection control and prevalence of anti-SARS-CoV-2 IgG in 4,554 employees of a university hospital in Munich, Germany. Published online October 2020. doi:10.1101/2020.10.04.20206136

32. Hanrath AT, Loeff IS van der, Lendrem DW, et al. SARS-CoV-2 testing of 11,884 healthcare workers at an acute NHS hospital trust in England: A retrospective analysis. *medRxiv*. Published online December 2020:2020.12.22.20242362. doi:10.1101/2020.12.22.20242362

33. Rosser JI, Röltgen K, Dymock M, et al. Severe acute respiratory coronavirus virus 2 (SARS-CoV-2) seroprevalence in healthcare personnel in northern California early in the coronavirus disease 2019 (COVID-19) pandemic. *Infection Control & Hospital Epidemiology*. Published online December 2020:1-7. doi:10.1017/ice.2020.1358

34. Silva VO, de Oliveira EL, Castejon MJ, et al. Prevalence of antibodies against sars-cov-2 in professionals of a public health laboratory at são paulo, sp, brazil. *medRxiv*. Published online October 2020. doi:10.1101/2020.10.19.20213421

35. Sabourin KR, Schultz J, Romero J, et al. Risk Factors of SARS-CoV-2 Antibodies in Arapahoe County First Responders - the COVID-19 Arapahoe SErosurveillance Study (CASES) Project. *Journal of Occupational and Environmental Medicine*. Published online December 2020. doi:10.1097/JOM.0000000000002099

36. Yogo N, Greenwood KL, Thompson L, et al. Point prevalence survey to evaluate the seropositivity for coronavirus disease 2019 (COVID-19) among high-risk healthcare workers. *Infection Control and Hospital Epidemiology*. Published online December 2020:1-6. doi:10.1017/ice.2020.1370

37. Figueiredo-Campos P, Blankenhaus B, Mota C, et al. Seroprevalence of anti-SARS-CoV-2 antibodies in COVID-19 patients and healthy volunteers up to 6 months post disease onset. *European Journal of Immunology*. 2020;50(12):2025-2040. doi:10.1002/eji.202048970

For peer review only

38. Gonçalves J, Sousa RL, Jacinto MJ, et al. Evaluating SARS-CoV-2 Seroconversion Following Relieve of Confinement Measures. *Frontiers in Medicine*. 2020;7. doi:10.3389/fmed.2020.603996

39. Fontanet A, Grant R, Tondeur L, et al. SARS-CoV-2 infection in primary schools in northern France: A retrospective cohort study in an area of high transmission. *medRxiv*. Published online June 2020:2020.06.25.20140178. doi:10.1101/2020.06.25.20140178

40. Torres JP, Piñera C, De La Maza V, et al. Severe Acute Respiratory Syndrome Coronavirus 2 Antibody Prevalence in Blood in a Large School Community Subject to a Coronavirus Disease 2019 Outbreak: A Cross-sectional Study. *Clinical Infectious Diseases*. Published online July 2020:ciaa955. doi:10.1093/cid/ciaa955

41. Halatoko WA, KONU YR, Gbeasor-Komlanvi FA, et al. Prevalence of SARS-CoV-2 among high-risk populations in Lom&Eacute (Togo) in 2020. *medRxiv*. Published online August 2020:2020.08.07.20163840. doi:10.1101/2020.08.07.20163840

42. Slusser S. MLB antibody study: 0.7% of those tested had been exposed to coronavirus. *San Francisco Chronicle*. Published online May 2020.

43. Vince A, Zadro R, Šostar Z, et al. SARS-CoV-2 Seroprevalence in a Cohort of Asymptomatic, RT-PCR Negative Croatian First League Football Players. *medRxiv*. Published online November 2020:2020.10.30.20223230. doi:10.1101/2020.10.30.20223230

44. Mack D, Gärtner BC, Rössler A, et al. Prevalence of SARS-CoV-2 IgG antibodies in a large prospective cohort study of elite football players in Germany (MayJune 2020): Implications for a testing protocol in asymptomatic individuals and estimation of the rate of undetected cases. *Clinical Microbiology and Infection*. 2020;27(3):473.e1-473.e4. doi:10.1016/j.cmi.2020.11.033

45. Khan SMS, Qurieshi MA, Haq I, et al. Seroprevalence of SARS-CoV-2 specific IgG antibodies in District Srinagar, northern India A cross-sectional study. *PLOS ONE*. 2020;15(11):e0239303. doi:10.1371/journal.pone.0239303

46. Akinbami LJ, Vuong N, Petersen LR, et al. SARS-CoV-2 Seroprevalence among Healthcare, First Response, and Public Safety Personnel, Detroit Metropolitan Area, Michigan, USA, MayJune 2020 - Volume 26, Number 12December 2020 - Emerging Infectious Diseases journal - CDC. Published online December 2020. doi:10.3201/eid2612.203764

47. Amendola A, Tanzi E, Folgori L, et al. Low seroprevalence of SARS-CoV-2 infection among healthcare workers of the largest children hospital in Milan during the pandemic wave. *Infection Control & Hospital Epidemiology*. Published online August 2020:1-2. doi:10.1017/ice.2020.401

48. Paulo Arnaldo. *Inquérito Sero-Epidemiológico de SARS-CoV-2 Na Cidade de Quelimane (InCOVID 2020)*. República de Moçambique Ministério da Saúde; 2020.

49. Bal A, Brengel-Pesce K, Gaymard A, et al. Clinical and microbiological assessments of COVID-19 in healthcare workers: A prospective longitudinal study. *medRxiv*. Published online November 2020:2020.11.04.20225862. doi:10.1101/2020.11.04.20225862

50. Fernández-Rivas G, Quirant-Sánchez B, González V, et al. Seroprevalence of SARS-CoV-2 IgG Specific Antibodies among Healthcare Workers in the Northern Metropolitan Area of Barcelona, Spain, after the first pandemic wave. *medRxiv*. Published online June 2020:2020.06.24.20135673. doi:10.1101/2020.06.24.20135673

51. Bardai G, Ouellet J, Engelhardt T, Bertolizio G, Wu Z, Rauch F. Prevalence of SARS-CoV-2 infections in a pediatric orthopedic hospital. von Ungern-Sternberg B, ed. *Pediatric Anesthesia*. 2021;31(2):247-248. doi:10.1111/pan.14047

For peer review only

52. Blairon L, Mokrane S, Wilmet A, et al. Large-scale, molecular and serological SARS-CoV-2 screening of healthcare workers in a 4-site public hospital in Belgium after COVID-19 outbreak. *Journal of Infection*. Published online July 2020:S0163445320305144. doi:10.1016/j.jinf.2020.07.033

53. Moreno Borraz LA, Giménez López M, Carrera Lasfuentes P, et al. Prevalencia de infección por coronavirus SARS-CoV-2 en pacientes y profesionales de un hospital de media y larga estancia en España. *Revista Española de Geriatría y Gerontología*. 2020;56(2):75-80. doi:10.1016/j.regg.2020.10.005

54. Brunner WM, Hirabayashi L, Krupa NL, et al. Severe acute respiratory coronavirus virus 2 (SARS-CoV-2) IgG results among healthcare workers in a rural upstate New York hospital system. *Infection Control & Hospital Epidemiology*. Published online October 2020:1-4. doi:10.1017/ice.2020.1296

55. Carozzi FM, Cusi MG, Pistello M, et al. Detection of asymptomatic SARS-CoV-2 infections among healthcare workers: Results from a large-scale screening program based on rapid serological testing. *medRxiv*. Published online August 2020. doi:10.1101/2020.07.30.20149567

56. Vilibic-Cavlek T, Stevanovic V, Tabain I, et al. Severe acute respiratory syndrome coronavirus 2 seroprevalence among personnel in the healthcare facilities of Croatia, 2020. *Revista da Sociedade Brasileira de Medicina Tropical*. 2020;53. doi:10.1590/0037-8682-0458-2020

57. Chibwana MG, Jere KC, kamng'ona R, et al. High SARS-CoV-2 seroprevalence in Health Care Workers but relatively low numbers of deaths in urban Malawi. *medRxiv*. Published online August 2020:2020.07.30.20164970. doi:10.1101/2020.07.30.20164970

58. Coffman B. New Co-Immunity Project data show COVID-19 infection among health care workers may be lower than the general population | UofL News. *UofLNews*. Published online August 2020.

59. Cooper DJ, Lear S, Watson L, et al. A prospective study of risk factors associated with seroprevalence of SARS-CoV-2 antibodies in healthcare workers at a large UK teaching hospital. *medRxiv*. Published online November 2020:2020.11.03.20220699. doi:10.1101/2020.11.03.20220699

60. Denyer S. Japanese firm's blanket testing of employees could serve as model. *LMT Online*. Published online June 2020.

61. Dimeglio C, Herin F, Miedougé M, et al. Screening for SARS-CoV-2 antibodies among healthcare workers in a university hospital in southern France. *Journal of Infection*. 2020;0(0). doi:10.1016/j.jinf.2020.09.035

62. Fuereder T, Berghoff AS, Heller G, et al. SARS-CoV-2 seroprevalence in oncology healthcare professionals and patients with cancer at a tertiary care centre during the COVID-19 pandemic. *ESMO Open*. 2020;5(5). doi:10.1136/esmoopen-2020-000889

63. Fusco FM, Pisaturo M, Iodice V, et al. COVID-19 among healthcare workers in a specialist infectious diseases setting in Naples, Southern Italy: Results of a cross-sectional surveillance study. *Journal of Hospital Infection*. 2020;105(4):596-600. doi:10.1016/j.jhin.2020.06.021

64. Geraci L. Antibody tests show just 2% exposure rate to COVID-19. *The Lancaster News*. Published online May 2020.

65. Eduardo Samo Gudo. *Inquérito Sero-epidemiológico de SARS-CoV-2 na Cidade de Nampula*. República de Moçambique Ministério da Saúde; 2020:19.

66. Hackner K, Errhalt P, Willheim M, et al. Diagnostic accuracy of two commercially available rapid assays for detection of IgG and IgM antibodies to SARS-CoV-2 compared to ELISA in a low-prevalence population. *Research Square*. Published online August 2020. doi:10.21203/rs.3.rs-50887/v1

For peer review only

67. Haq M, Rehman A, Noor M, et al. Seroprevalence and Risk Factors of SARS CoV-2 in Health Care Workers of Tertiary-Care Hospitals in the Province of Khyber Pakhtunkhwa, Pakistan. *medRxiv*. Published online September 2020:2020.09.29.20203125. doi:10.1101/2020.09.29.20203125

68. He L, Zeng Y, Zeng C, et al. Positive Rate of Serology and RT-PCR for COVID-19 among healthcare workers during different periods in Wuhan, China. *Journal of Infection*. Published online August 2020. doi:10.1016/j.jinf.2020.08.027

69. Herzberg J, Vollmer T, Fischer B, et al. Prospective Sero-epidemiological Evaluation of SARS-CoV-2 among Health Care Workers in a German Secondary Care Hospital. *International Journal of Infectious Diseases*. 2021;102:136-143. doi:10.1016/j.ijid.2020.10.026

70. Jeremias A, Nguyen J, Levine J, et al. Prevalence of SARS-CoV-2 Infection Among Health Care Workers in a Tertiary Community Hospital. *JAMA Internal Medicine*. Published online August 2020. doi:10.1001/jamainternmed.2020.4214

71. Jespersen S, Mikkelsen S, Greve T, et al. Severe Acute Respiratory Syndrome Coronavirus 2 Seroprevalence Survey Among 17 971 Healthcare and Administrative Personnel at Hospitals, Prehospital Services, and Specialist Practitioners in the Central Denmark Region. *Clinical Infectious Diseases*. Published online October 2020:ciaa1471. doi:10.1093/cid/ciaa1471

72. Kassem AM, Talaat H, Shawky S, et al. SARS-CoV-2 infection among healthcare workers of a gastroenterological service in a tertiary care facility. *Arab Journal of Gastroenterology*. 2020;21(3):151-155. doi:10.1016/j.ajg.2020.07.005

73. Kern PM, Müller H-H, Menzel T, Weisser H. Studie zur Immunität gegen SARS-CoV-2: Keine signifikante humorale Immunität gegen SARS-CoV-2 im medizinischen Personal eines Klinikums der Maximalversorgung und in der Stadtregion Fulda. *Der Klinikarzt*. 2020;49(06):268-273. doi:10.1055/a-1198-1243

74. Khalil A, Hill R, Wright A, Ladhani S, O'Brien P. SARS-CoV-2-Specific Antibody Detection in Healthcare Workers in a UK Maternity Hospital: Correlation With SARS-CoV-2 RT-PCR Results. *Clinical Infectious Diseases*. 2020;(ciaa893). doi:10.1093/cid/ciaa893

75. Kumar A, Sathyapalan D, Ramachandran A, Subhash K, Biswas L, Beena KV. SARS-CoV-2 antibodies in healthcare workers in a large university hospital, Kerala, India. *Clinical Microbiology and Infection*. 2021;27(3):481-483. doi:10.1016/j.cmi.2020.09.013

76. Lackermair K, William F, Grzanna N, et al. Infection with SARS-CoV-2 in primary care health care workers assessed by antibody testing. *Family Practice*. Published online August 2020:cmaa078. doi:10.1093/fampra/cmaa078

77. Lahner E, Dilaghi E, Prestigiacomo C, et al. Prevalence of Sars-Cov-2 Infection in Health Workers (HWs) and Diagnostic Test Performance: The Experience of a Teaching Hospital in Central Italy. *International Journal of Environmental Research and Public Health*. 2020;17(12). doi:10.3390/ijerph17124417

78. Liu M, Cheng S-Z, Xu K-W, et al. Use of personal protective equipment against coronavirus disease 2019 by healthcare professionals in Wuhan, China: Cross sectional study. *BMJ*. 2020;369. doi:10.1136/bmj.m2195

79. Liu T, Wu S, Tao H, Zeng G, Zhou F, Wang X. Prevalence of IgG Antibodies to SARS-CoV-2 in Wuhan Implications for the Longevity of Antibodies Against SARS-CoV-2. *Research Square*. Published online November 2020. doi:10.21203/rs.3.rs-99748/v1

80. Lorenzo D, Carrisi C. COVID-19 exposure risk for family members of healthcare workers: An observational study. *International Journal of Infectious Diseases*. 2020;98:287-289. doi:10.1016/j.ijid.2020.06.106

For peer review only

81. Mussagy Mahomed. *Inquérito Sero-Epidemiológico de SARS-CoV-2 Na Cidade de Tete (InCOVID 2020)*. República de Moçambique Ministério da Saúde; 2020.

82. Arlete Mahumane. *Inquérito Sero-Epidemiológico de SARS-CoV-2 Na Cidade de Chimoio (InCOVID 2020)*. República de Moçambique Ministério da Saúde; 2020.

83. Majdoubi A, Michalski C, O'Connell SE, et al. Antibody reactivity to SARS-CoV-2 is common in unexposed adults and infants under 6 months. *medRxiv*. Published online November 2020:2020.10.05.20206664. doi:10.1101/2020.10.05.20206664

84. Majiya H, Aliyu-Paiko M, Balogu VT, et al. Seroprevalence of COVID-19 in Niger State. *medRxiv*. Published online August 2020. doi:10.1101/2020.08.04.20168112

85. Fill Malfertheiner S, Brandstetter S, Roth S, et al. Immune response to SARS-CoV-2 in health care workers following a COVID-19 outbreak: A prospective longitudinal study. *Journal of Clinical Virology*. 2020;130:104575. doi:10.1016/j.jcv.2020.104575

86. Martin C, Montesinos I, Dauby N, et al. Dynamics of SARS-CoV-2 RT-PCR positivity and seroprevalence among high-risk healthcare workers and hospital staff. *Journal of Hospital Infection*. 2020;106(1):102-106. doi:10.1016/j.jhin.2020.06.028

87. de Melo MS, Borges LP, de Souza DRV, et al. *Anti-SARS-CoV-2 IgM and IgG Antibodies in Health Workers in Sergipe, Brazil*. Infectious Diseases (except HIV/AIDS); 2020. doi:10.1101/2020.09.24.20200873

88. Morcuende M, Guglielminotti J, Landau R. Anesthesiologists' and Intensive Care Providers' Exposure to COVID-19 Infection in a New York City Academic Center: A Prospective Cohort Study Assessing Symptoms and COVID-19 Antibody Testing. *Anesthesia and analgesia*. 2020;131(3):669-676. doi:10.1213/ANE.0000000000005056

89. Moscola J, Sembajwe G, Jarrett M, et al. Prevalence of SARS-CoV-2 Antibodies in Health Care Personnel in the New York City Area. *JAMA*. 2020;324(9):893-895. doi:10.1001/jama.2020.14765

90. Nishida T, Iwahashi H, Yamauchi K, et al. Seroprevalence of SARS-CoV-2 Antibodies Among 925 Staff Members in an Urban Hospital Accepting COVID-19 Patients in Osaka Prefecture, Japan. *medRxiv*. Published online January 2020:2020.09.10.20191866. doi:10.1101/2020.09.10.20191866

91. Olalla J, Correa AM, Martín-Escalante MD, et al. Search for asymptomatic carriers of SARS-CoV-2 in healthcare workers during the pandemic: A Spanish experience. *QJM: An International Journal of Medicine*. 2020;(hcaa238). doi:10.1093/qjmed/hcaa238

92. Pallett SJC, Rayment M, Patel A, et al. Point-of-care serological assays for delayed SARS-CoV-2 case identification among health-care workers in the UK: A prospective multicentre cohort study. *The Lancet Respiratory Medicine*. 2020;8(9):885-894. doi:10.1016/S2213-2600(20)30315-5

93. Péré H, Wack M, Védie B, et al. Sequential SARS-CoV-2 IgG assays as confirmatory strategy to confirm equivocal results: Hospital-wide antibody screening in 3,569 staff health care workers in Paris. *Journal of Clinical Virology*. 2020;132:104617. doi:10.1016/j.jcv.2020.104617

94. Poulikakos D, Sinha S, Kalra PA. SARS-CoV-2 antibody screening in healthcare workers in a tertiary centre in North West England. *Journal of clinical virology : the official publication of the Pan American Society for Clinical Virology*. 2020;129:104545-104545. doi:10.1016/j.jcv.2020.104545

95. Psichogiou M, Karabinis A, Pavlopoulou I, et al. Antibodies against SARS-CoV-2 among health care workers in a country with low burden of COVID-19. *medRxiv*. Published online June 2020. doi:10.1101/2020.06.23.20137620

For peer review only

96. Kolthur-Seetharam U, Shah D, Shastri J, et al. *SARS-CoV2 Serological Survey in Mumbai by NITI-BMC-TIFR: Preliminary Report of Round-2*. NITI-BMC-TIFR; 2020.

97. Shields AM, Faustini SE, Perez-Toledo M, et al. SARS-CoV-2 seroconversion in health care workers. *medRxiv*. Published online May 2020:2020.05.18.20105197. doi:10.1101/2020.05.18.20105197

98. Ismael Amaral Silva PA, Ismael C, Marchon da Silva C, Domenge C. 1761P Universal screening of SARS-CoV-2 of oncology healthcare workers a Brazilian experience. *Annals of Oncology*. 2020;31:S1024. doi:10.1016/j.annonc.2020.08.1825

99. Solodky ML, Galvez C, Russias B, et al. Lower detection rates of SARS-COV2 antibodies in cancer patients versus health care workers after symptomatic COVID-19. *Annals of Oncology*. 2020;31(8):1087-1088. doi:10.1016/j.annonc.2020.04.475

100. Soriano V, Meiriño R, Corral O, Guallar MP. Severe Acute Respiratory Syndrome Coronavirus 2 Antibodies in Adults in Madrid, Spain. *Clinical Infectious Diseases*. 2020;(ciaa769). doi:10.1093/cid/ciaa769

101. Instituto Nazionale di Statistica. *PRIMI RISULTATI DELL'INDAGINE DI SIEROPREVALENZA SUL SARS-CoV-2*. Instituto Nazionale di Statistica; 2020.

102. Steensels D, Oris E, Coninx L, et al. Hospital-Wide SARS-CoV-2 Antibody Screening in 3056 Staff in a Tertiary Center in Belgium. *JAMA*. 2020;(7501160). doi:10.1001/jama.2020.11160

103. Stock AD, Bader ER, Cezayirli P, et al. COVID-19 Infection Among Healthcare Workers: Serological Findings Supporting Routine Testing. *Frontiers in Medicine*. 2020;7. doi:10.3389/fmed.2020.00471

104. Takita M, Matsumura T, Yamamoto K, et al. Geographical Profiles of COVID-19 Outbreak in Tokyo: An Analysis of the Primary Care ClinicBased Point-of-Care Antibody Testing. *Journal of Primary Care & Community Health*. 2020;11:215013272094269. doi:10.1177/2150132720942695

105. Tong X, Ning M, Huang R, et al. Surveillance of SARS-CoV-2 infection among frontline health care workers in Wuhan during COVID-19 outbreak. *Immunity, Inflammation and Disease*. 2020;8(4):840-843. doi:10.1002/iid3.340

106. Trieu M-C, Bansal A, Madsen A, et al. SARS-CoV-2Specific Neutralizing Antibody Responses in Norwegian Health Care Workers After the First Wave of COVID-19 Pandemic: A Prospective Cohort Study. *The Journal of Infectious Diseases*. 2020;2021-(jiaa737). doi:10.1093/infdis/jiaa737

107. Tu D, Shu J, Wu X, et al. Immunological detection of serum antibodies in pediatric medical workers exposed to varying levels of SARS-CoV-2. *The Journal of Infection*. 2021;82(1):159-198. doi:10.1016/j.jinf.2020.07.023

108. Valdivia A, Torres I, Huntley D, et al. Caveats in interpreting SARS-CoV-2 IgM+/IgG- antibody profile in asymptomatic health care workers. *Journal of Medical Virology*. 2020;n/a(n/a). doi:10.1002/jmv.26400

109. Chafloque-Vasquez RA, Pampa-Espinoza L, Salinas JCC. Seroprevalence of COVID-19 in workers in a hospital in the Peruvian Amazon. *ACTA MEDICA PERUANA*. 2020;37(3). doi:10.35663/amp.2020.373.1050

110. Edna Viegas. *Inquérito Sero-Epidemiológico de SARS-CoV-2 Na Cidade de Maputo (InCOVID 2020)*. República de Moçambique Ministério da Saúde; 2020.

For peer review only

111. Vlachoyiannopoulos P, Alexopoulos H, Apostolidi I, et al. Anti-SARS-CoV-2 antibody detection in healthcare workers of two tertiary hospitals in Athens, Greece. *Clinical Immunology*. 2020;221:108619. doi:10.1016/j.clim.2020.108619

112. Dalla Volta A, Valcamonico F, Pedersini R, et al. The Spread of SARS-CoV-2 Infection Among the Medical Oncology Staff of ASST Spedali Civili of Brescia: Efficacy of Preventive Measures. *Frontiers in Oncology*. 2020;10:1574. doi:10.3389/fonc.2020.01574

113. Ward H, Cooke G, Atchison C, et al. Declining prevalence of antibody positivity to SARS-CoV-2: A community study of 365,000 adults. *medRxiv*. Published online October 2020:2020.10.26.20219725. doi:10.1101/2020.10.26.20219725

114. Xiong S, Guo C, Dittmer U, Zheng X, Wang B. The prevalence of antibodies to SARS-CoV-2 in asymptomatic healthcare workers with intensive exposure to COVID-19. *medRxiv*. Published online June 2020:2020.05.28.20110767. doi:10.1101/2020.05.28.20110767

115. Zhang J, Liu J, Li N, et al. Serological detection of 2019-nCoV respond to the epidemic: A useful complement to nucleic acid testing. *medRxiv*. Published online March 2020:2020.03.04.20030916. doi:10.1101/2020.03.04.20030916

116. Zhao D, Wang M, Wang M, et al. Asymptomatic infection by SARS-CoV-2 in healthcare workers: A study in a large teaching hospital in Wuhan, China. *International Journal of Infectious Diseases*. 2020;99:219-225. doi:10.1016/j.ijid.2020.07.082

117. Ahmad K, Rezvanizadeh V, Dahal S, et al. COVID-19 IgG/IgM antibody testing in Los Angeles County, California. *European Journal of Clinical Microbiology & Infectious Diseases*. Published online November 2020. doi:10.1007/s10096-020-04111-3

118. Halbrook M, Gadoth A, Martin-Blais R, et al. Incidence of SARS-CoV-2 infection among asymptomatic frontline health workers in Los Angeles County, California. *medRxiv*. Published online November 2020:2020.11.18.20234211. doi:10.1101/2020.11.18.20234211

119. Iwuji K, Islam E, Berdine G, Nugent K, Test V, Tijerina A. Prevalence of Coronavirus Antibody Among First Responders in Lubbock, Texas. *Journal of Primary Care & Community Health*. 11:2150132720971390. doi:10.1177/2150132720971390

120. Parker-Magyar A. Few among Long Hill first responders test positive for COVID-19 antibodies. *Echoes Sentinel*. Published online June 2020.

121. Caban-Martinez AJ, Schaefer-Solle N, Santiago K, et al. Epidemiology of SARS-CoV-2 antibodies among firefighters/paramedics of a US fire department: A cross-sectional study. *Occupational and Environmental Medicine*. 2020;77(12):857-861. doi:10.1136/oemed-2020-106676

122. Staletovich J. South Florida Cities Begin Testing Employees For COVID-19 Antibodies. *WLRN*. Published online May 2020.

123. Hibino M, Iwabuchi S, Munakata H. SARS-CoV-2 IgG seroprevalence among medical staff in a general hospital that treated patients with COVID-19 in Japan: Retrospective evaluation of nosocomial infection control. *Journal of Hospital Infection*. 2020;107:103-104. doi:10.1016/j.jhin.2020.10.001

124. Calcagno A, Ghisetti V, Emanuele T, et al. Risk for SARS-CoV-2 Infection in Healthcare Workers, Turin, Italy. *Emerging Infectious Diseases*. 2021;27(1):303-305. doi:10.3201/eid2701.203027

125. Alharbi SA, Almutairi AZ, Jan AA, Alkhalify AM. Enzyme-Linked Immunosorbent Assay for the Detection of Severe Acute Respiratory Syndrome Coronavirus 2 (SARS-CoV-2) IgM/IgA and IgG Antibodies Among Healthcare Workers. *Cureus*. Published online September 2020. doi:10.7759/cureus.10285

For peer review only

126. Chau NVV, Toan LM, Man DNH, et al. Absence of SARS-CoV-2 antibodies in health care workers of a tertiary referral hospital for COVID-19 in southern Vietnam. *Journal of Infection*. 2020;82(1):e36-e37. doi:10.1016/j.jinf.2020.11.018

127. Khan MS, Haq I, Qurieshi MA, et al. SARS-CoV-2 seroprevalence in healthcare workers of dedicated-COVID hospitals and nonCOVID hospitals of District Srinagar, Kashmir. *medRxiv*. Published online October 2020:2020.10.23.20218164. doi:10.1101/2020.10.23.20218164

128. Costa SF, Giavina-Bianchi P, Buss L, et al. SARS-CoV-2 seroprevalence and risk factors among oligo/asymptomatic healthcare workers(HCW): Estimating the impact of community transmission. *Clinical Infectious Diseases*. 2020;(ciaa1845). doi:10.1093/cid/ciaa1845

129. Mohr N, Harland K, Krishnadasan A, Santibanez S, Talan D. Diagnosed and Undiagnosed COVID-19 in US Emergency Department Health Care Personnel: A Cross-sectional Analysis. *Annals of Emergency Medicine*. Published online December 2020. doi:10.1016/j.annemergmed.2020.12.007

130. Noor M, Haq M, Ul Haq N, et al. Does Working in a COVID-19 Receiving Health Facility Influence Seroprevalence to SARS-CoV-2? *Cureus*. Published online November 2020. doi:10.7759/cureus.11389

131. Singhal T, Shah S, Naik R, Kazi A, Thakkar P. Prevalence of COVID-19 Antibodies in Healthcare Workers at the Peak of the Pandemic in Mumbai, India: A Preliminary Study. *Indian Journal of Medical Microbiology*. 2020;38(3):461-463. doi:10.4103/ijmm.IJMM_20_308

132. Dacosta-Urbieta A, Rivero-Calle I, Pardo-Seco J, et al. Seroprevalence of SARS-CoV-2 Among Pediatric Healthcare Workers in Spain. *Frontiers in Pediatrics*. 2020;8. doi:10.3389/fped.2020.00547

133. Sartore-Bianchi A, Patelli G, Tosi F, et al. INCIDENCE OF SARS-COV-2 INFECTION IN PATIENTS WITH ACTIVE CANCER: MONO-INSTITUTIONAL SERIES OF A COMPREHENSIVE CANCER INSTITUTION IN LOMBARDY DURING THE COVID-19 PANDEMIC (NIGUARDA CANCER CENTER, MILANO, ITALY). In: *Tumori Journal*. Vol 106. AIOM Abstracts.; 2020:1-215. doi:10.1177/0300891620953388

134. Brousseau N, Morin L, Ouakki M, et al. *COVID-19 : Étude de séroprévalence chez des travailleurs de la santé de centres hospitaliers au Québec*. Institut National de Sante Publique du Quebec; 2020:20.

135. Chen Y, Tong X, Wang J, et al. High SARS-CoV-2 antibody prevalence among healthcare workers exposed to COVID-19 patients. *The Journal of Infection*. 2020;81(3):420-426. doi:10.1016/j.jinf.2020.05.067

136. Favara DM, Cooke A, Doffinger R, McAdam K, Corrie P, Ainsworth NL. COVID-19 Serology in Oncology Staff Study: Understanding SARS-CoV-2 in the Oncology Workforce. *Clinical Oncology (Royal College of Radiologists (Great Britain)*. 2021;33(1):e61-e63. doi:10.1016/j.clon.2020.07.015

137. Fujita K, Shinpei Kada, Osamu Kanai, et al. Quantitative SARS-CoV-2 antibody screening of healthcare workers in the southern part of Kyoto city during the COVID-19 peri-pandemic period. *medRxiv*. Published online May 2020.

138. Godbout EJ, Pryor R, Harmon M, et al. Severe acute respiratory coronavirus virus 2 (SARS-CoV-2) seroprevalence among healthcare workers in a low prevalence region. *Infection Control & Hospital Epidemiology*. Published online December 2020:1-3. doi:10.1017/ice.2020.1374

139. Houlihan CF, Vora N, Byrne T, et al. Pandemic peak SARS-CoV-2 infection and seroconversion rates in London frontline health-care workers. *The Lancet*. 2020;396(10246):e6-e7. doi:10.1016/S0140-6736(20)31484-7

For peer review only

140. Insúa C, Stedile G, Figueroa V, et al. Seroprevalence of SARS-CoV-2 antibodies among physicians from a children's hospital. *Archivos Argentinos De Pediatria*. 2020;118(6):381-385. doi:10.5546/aap.2020.eng.381

141. Kohler PP, Kahlert CR, Sumer J, et al. Prevalence of SARS-CoV-2 antibodies among Swiss hospital workers: Results of a prospective cohort study. *Infection Control & Hospital Epidemiology*. Published online October 2020:1-5. doi:10.1017/ice.2020.1244

142. Kumar N, Bhartiya S, Singh T. Duration of anti-SARS-CoV-2 antibodies much shorter in India. *Vaccine*. 2021;39(6):886-888. doi:10.1016/j.vaccine.2020.10.094

143. Mesnil M, Joubel K, Yavchitz A, Miklaszewski N, Devys J-M. Seroprevalence of SARS-Cov-2 in 646 professionals at the Rothschild Foundation Hospital (ProSeCoV study). *Anaesthesia Critical Care & Pain Medicine*. 2020;39(5):595-596. doi:10.1016/j.accpm.2020.08.003

144. Missaglia R, Belingheri M, Antolini L, et al. SARS-CoV-2 pandemia in Lombardy: The impact on family Paediatricians. *Italian Journal of Pediatrics*. 2020;46(1):184. doi:10.1186/s13052-020-00950-0

145. Orth-Höller D, Eigentler A, Weseslindtner L, Möst J. Antibody kinetics in primary- and secondary-care physicians with mild to moderate SARS-CoV-2 infection. *Emerging Microbes & Infections*. 2020;9(1):1692-1694. doi:10.1080/22221751.2020.1793690

146. Plebani M, Padoan A, Fedeli U, et al. SARS-CoV-2 serosurvey in health care workers of the Veneto Region. *Clinical Chemistry and Laboratory Medicine (CCLM)*. 2020;58(12):2107-2111. doi:10.1515/cclm-2020-1236

147. Rudberg A-S, Havervall S, Månberg A, et al. SARS-CoV-2 exposure, symptoms and seroprevalence in healthcare workers in Sweden. *Nature Communications*. 2020;11(1):5064. doi:10.1038/s41467-020-18848-0

148. Schmidt SB, Grüter L, Boltzmann M, Rollnik JD. Prevalence of serum IgG antibodies against SARS-CoV-2 among clinic staff. Adrish M, ed. *PLOS ONE*. 2020;15(6):e0235417. doi:10.1371/journal.pone.0235417

149. Sotgiu G, Barassi A, Miozzo M, et al. SARS-CoV-2 specific serological pattern in healthcare workers of an Italian COVID-19 forefront hospital. *BMC Pulmonary Medicine*. 2020;20(1):203. doi:10.1186/s12890-020-01237-0

150. Venugopal U, Jilani N, Rabah S, et al. SARS-CoV-2 seroprevalence among health care workers in a New York City hospital: A cross-sectional analysis during the COVID-19 pandemic. *International Journal of Infectious Diseases*. 2020;102:63-69. doi:10.1016/j.ijid.2020.10.036

151. Racine-Brzostek SE, Yang HS, Chadburn A, et al. COVID-19 Viral and Serology Testing in New York City Health Care Workers. *American Journal of Clinical Pathology*. 2020;154(5):592-595. doi:10.1093/ajcp/aqaa142

152. Hoffmann S, Spallek J, Heinz-Detlef G, Schiebel J, Hufert F. Testing the backbone of the healthcare system: A prospective serological-epidemiological cohort study of healthcare workers in rural Germany. Published online September 2020. doi:10.21203/rs.3.rs-84703/v1

153. Patel MM, Thornburg NJ, Stubblefield WB, et al. Change in Antibodies to SARS-CoV-2 Over 60 Days Among Health Care Personnel in Nashville, Tennessee. *JAMA*. 2020;324(17):1781. doi:10.1001/jama.2020.18796

For peer review only

154. Self WH, Tenforde MW, Stubblefield WB, et al. Seroprevalence of SARS-CoV-2 Among Frontline Health Care Personnel in a Multistate Hospital Network 13 Academic Medical Centers, AprilJune 2020. *MMWR Morbidity and Mortality Weekly Report*. 2020;69(35):1221-1226. doi:10.15585/mmwr.mm6935e2

155. Shah VP, Hainy CM, Swift MD, Breeher LE, Theel ES, Sampathkumar P. Unrecognized severe acute respiratory coronavirus virus 2 (SARS-CoV-2) seroprevalence among healthcare personnel in a low-prevalence area. *Infection Control & Hospital Epidemiology*. Published online November 2020:1-3. doi:10.1017/ice.2020.1341

156. Bampoe S, Lucas DN, Neall G, et al. A cross-sectional study of immune seroconversion to SARS-CoV-2 in front-line maternity health professionals. *medRxiv*. Published online June 2020. doi:10.1101/2020.06.24.20139352

157. Dimcheff DE, Schildhouse RJ, Hausman MS, et al. Seroprevalence of severe acute respiratory syndrome coronavirus-2 (SARS-CoV-2) infection among Veterans Affairs healthcare system employees suggests higher risk of infection when exposed to SARS-CoV-2 outside the work environment. *Infection Control & Hospital Epidemiology*.:1-7. doi:10.1017/ice.2020.1220

158. Finkenzeller T, Faltlhauser A, Dietl K-H, et al. SARS-CoV-2-Antikörper bei Intensiv- und Klinikpersonal. *Medizinische Klinik - Intensivmedizin und Notfallmedizin*. 2020;115(3):139-145. doi:10.1007/s00063-020-00761-5

159. Grant JJ, Wilmore SMS, McCann NS, et al. Seroprevalence of SARS-CoV-2 antibodies in healthcare workers at a London NHS Trust. *Infection Control & Hospital Epidemiology*. Published online August 2020:1-3. doi:10.1017/ice.2020.402

160. Mansour M, Leven E, Muellers K, Stone K, Mendu DR, Wajnberg A. Prevalence of SARS-CoV-2 Antibodies Among Healthcare Workers at a Tertiary Academic Hospital in New York City. *Journal of General Internal Medicine*. 2020;35(8):2485-2486. doi:10.1007/s11606-020-05926-8

161. Martín V, Fernández-Villa T, Lamuedra Gil de Gomez M, et al. Prevalence of SARS-CoV-2 infection in general practitioners and nurses in primary care and nursing homes in the Healthcare Area of León and associated factors. *COVID19 en Atención Primaria*. 2020;46:35-39. doi:10.1016/j.semerg.2020.05.014

162. Meissner EG, Litwin C, Crocker T, Mack E, Card L. 460. Point-of-Care, In-Home SARS-CoV-2 IgG Antibody Testing to Assess Seroprevalence in At-Risk Health Care Workers. *Open Forum Infectious Diseases*. 2020;7(Supplement_1):S297-S297. doi:10.1093/ofid/ofaa439.653

163. Mostafa A, Kandil S, El-Sayed MH, et al. Universal COVID-19 screening of 4040 health care workers in a resource-limited setting: An Egyptian pilot model in a university with 12 public hospitals and medical centers. *International Journal of Epidemiology*. 2020;(dyaa173). doi:10.1093/ije/dyaa173

164. Paradiso AV, Summa simona D, Silvestris N, et al. COVID-19 SCREENING AND MONITORING OF ASYMPTOMATIC HEALTH WORKERS WITH A RAPID SEROLOGICAL TEST. *medRxiv*. Published online May 2020:2020.05.05.20086017. doi:10.1101/2020.05.05.20086017

165. Sydney ER, Kishore P, Laniado I, Rucker LM, Bajaj K, Zinaman MJ. Antibody evidence of SARS-CoV-2 infection in healthcare workers in the Bronx. *Infection Control & Hospital Epidemiology*. 2020;41(11):1348-1349. doi:10.1017/ice.2020.437

166. Zhou F, Li J, Lu M, et al. Tracing asymptomatic SARS-CoV-2 carriers among 3674 hospital staff:A cross-sectional survey. *EClinicalMedicine*. 2020;26. doi:10.1016/j.eclinm.2020.100510

167. Buntinx F, Claes P, Gulikers M, et al. Added value of anti-SARS-CoV-2 antibody testing in a Flemish nursing home during an acute COVID-19 outbreak in April 2020. *Acta Clinica Belgica*. 2020;0(0):1-6. doi:10.1080/17843286.2020.1834285

For peer review only

168. Reuben J, Sherman A, Ellison JA, et al. SARS-CoV-2 Seroprevalence among First Responders in the District of Columbia, May July 2020. *medRxiv*. Published online November 2020:2020.11.25.20225490. doi:10.1101/2020.11.25.20225490

169. Saberian P, Mireskandari SM, Baratloo A, et al. Antibody Rapid Test Results in Emergency Medical Services Personnel during COVID-19 Pandemic; a Cross Sectional study. *Archives of Academic Emergency Medicine*. 2020;9(1).

170. Tarabichi Y, Watts B, Collins T, et al. SARS-CoV-2 Infection among Serially Tested Emergency Medical Services Workers. *Prehospital Emergency Care*. 2020;0(0):1-7. doi:10.1080/10903127.2020.1831668

171. Vijh R, Ghafari C, Hayden A, et al. Serological survey following SARS-COV-2 outbreaks at long-term care facilities in metro Vancouver, British Columbia: Implications for outbreak management and infection control policies. *American Journal of Infection Control*. Published online October 2020. doi:10.1016/j.ajic.2020.10.009

172. Bhattacharya D, Winnett A, Fulcher JA, et al. 70. Lack of SARS-CoV-2 Antibody Seroconversion After Prompt Identification and Cohorting of Sentinel sars-cov-2-positive Residents in a Skilled Nursing Facility. *Open Forum Infectious Diseases*. 2020;7(Supplement_1):S165-S166. doi:10.1093/ofid/ofaa439.380

173. Pérez-García F, Pérez-Zapata A, Arcos N, et al. Severe acute respiratory coronavirus virus 2 (SARS-CoV-2) infection among hospital workers in a severely affected institution in Madrid, Spain: A surveillance cross-sectional study. *Infection Control & Hospital Epidemiology*. Published online October 2020:1-7. doi:10.1017/ice.2020.1303

174. Pérez-García F, Pérez-Zapata A, Arcos N, et al. Severe acute respiratory coronavirus virus 2 (SARS-CoV-2) infection among hospital workers in a severely affected institution in Madrid, Spain: A surveillance cross-sectional study. *Infection Control & Hospital Epidemiology*. 2021;42(7):803-809. doi:10.1017/ice.2020.1303

175. Mughal MS, Kaur IP, Patton CD, Mikhail NH, Vareechon C, Granet KM. The prevalence of severe acute respiratory coronavirus virus 2 (SARS-CoV-2) IgG antibodies in intensive care unit (ICU) healthcare personnel (HCP) and its implicationsa single-center, prospective, pilot study. *Infection Control & Hospital Epidemiology*. Published online June 2020:1-2. doi:10.1017/ice.2020.298

176. Rao S. Covid-19: Jayadeva says its survey hints at herd immunity. *The Times of India*. Published online June 2020.

177. Shukla V, Lau CSM, Towns M, et al. COVID-19 Exposure Among First Responders in Arizona. *Journal of Occupational and Environmental Medicine*. 2020;62(12).

178. Gray A. Prevalence Of COVID-19 Antibodies In Washoe Co. Expected To Be Low. *KUNR*. Published online June 2020.

179. Chughtai O, Batool H, Khan M, Chughtai A. Frequency of COVID-19 IgG Antibodies among Special Police Squad Lahore, Pakistan. *Journal of the College of Physicians and Surgeons Pakistan*. 2020;30(7):735-739. doi:10.29271/jcpsp.2020.07.735

180. Gujski M, Jankowski M, Pinkas J, et al. Prevalence of Current and Past SARS-CoV-2 Infections among Police Employees in Poland, JuneJuly 2020. *Journal of Clinical Medicine*. 2020;9(10):3245. doi:10.3390/jcm9103245

181. Jerónimo Langa. *Inquérito Sero-Epidemiológico de SARS-CoV-2 Na Cidade de Lichinga (InCOVID 2020)*. República de Moçambique Ministério da Saúde; 2020.

For peer review only

182. Ivalda Macicame. *Inquérito Sero-Epidemiológico de SARS-CoV-2 Na Província de Maputo (InCOVID 2020)*. República de Moçambique Ministério da Saúde; 2020.

183. Al-Thani MH, Farag E, Bertollini R, et al. Seroprevalence of SARS-CoV-2 infection in the craft and manual worker population of Qatar. *medRxiv*. Published online November 2020:2020.11.24.20237719. doi:10.1101/2020.11.24.20237719

184. Epstude J, Harsch IA. Seroprevalence of COVID-19 antibodies in the cleaning and oncological staff of a municipal clinic. *GMS Hygiene and Infection Control; 15:Doc18*. Published online July 2020. doi:10.3205/DGKH000353

185. Hassan SS, Seigerud Å, Mühr LSA, et al. SARS-CoV-2 infections among personnel providing home care services for the elderly in Stockholm, Sweden. *medRxiv*. Published online December 2020. doi:10.1101/2020.12.18.20248511

186. Ladhani SN, Jeffery-Smith A, Patel M, et al. High prevalence of SARS-CoV-2 antibodies in care homes affected by COVID-19: Prospective cohort study, England. *EClinicalMedicine*. 2020;28. doi:10.1016/j.eclinm.2020.100597

187. Lindahl JF, Hoffman T, Esmaeilzadeh M, et al. High seroprevalence of SARS-CoV-2 in elderly care employees in Sweden. *Infection Ecology & Epidemiology*. 2020;10(1):1789036. doi:10.1080/20008686.2020.1789036

188. Regan T. Fellowship Village Benefits from Covid-19 Antibody Tests. *Senior Housing News*. Published online June 2020.

189. Alali WQ, Bastaki H, Longenecker JC, et al. Seroprevalence of SARS-CoV-2 in migrant workers in Kuwait. *Journal of Travel Medicine*. 2020;(taaa223). doi:10.1093/jtm/taaa223

190. Addetia A, Crawford KHD, Dingens A, et al. Neutralizing Antibodies Correlate with Protection from SARS-CoV-2 in Humans during a Fishery Vessel Outbreak with a High Attack Rate. McAdam AJ, ed. *Journal of Clinical Microbiology*. 2020;58(11):e02107-20, /jcm/58/11/JCM.02107-20.atom. doi:10.1128/JCM.02107-20

191. Picon RV, Carreno I, da Silva AA, et al. Coronavirus disease 2019 population-based prevalence, risk factors, hospitalization, and fatality rates in southern Brazil. *International Journal of Infectious Diseases*. 2020;100:402-410. doi:10.1016/j.ijid.2020.09.028

192. D B, L B, P T, Pa P, A B, U L. Effectiveness of the measures aimed at containing Sars-cov-2 virus spreading in work settings: A survey in companies based in the Veneto region of Italy. *La Medicina del lavoro*. Published online October 2020. doi:10.23749/mdl.v111i5.10037

193. Xu X, Sun J, Nie S, et al. Seroprevalence of immunoglobulin M and G antibodies against SARS-CoV-2 in China. *Nature Medicine*. 2020;26(8):1193-1195. doi:10.1038/s41591-020-0949-6

194. Chamie G, Marquez C, Crawford E, et al. Community Transmission of Severe Acute Respiratory Syndrome Coronavirus 2 Disproportionately Affects the Latinx Population During Shelter-in-Place in San Francisco. *Clinical Infectious Diseases*. Published online August 2020:ciaa1234. doi:10.1093/cid/ciaa1234

195. McLaughlin C, Doll MK, Morrison KT, et al. High Community SARS-CoV-2 Antibody Seroprevalence in a Ski Resort Community, Blaine County, Idaho, US. Preliminary Results. *medRxiv*. Published online July 2020. doi:10.1101/2020.07.19.20157198

196. Muñoz L, Pífano M, Bolzán A, et al. *Surveillance and Seroprevalence: Evaluation of IgG Antibodies for SARS-Cov2 by ELISA in the Popular Neighborhood Villa Azul, Quilmes, Province of Buenos Aires, Argentina*.; 2020. doi:10.1590/SciELOPreprints.1147

For peer review only

Supplementary File 2.

Modified Joanna Briggs Institute Tool for assessing study risk of bias[1]

**Item 1: Was the sample frame appropriate to address the target population?**	
Yes	Sample frame described and it approximated the target population
No	Sample frame did not approximate the target population (e.g., blood donors do not represent general population, doctors do not represent all health care providers)
Exclude	Sample frame not described
*Notes	The term "target population" should not be taken to infer every individual from everywhere or with similar disease or exposure characteristics. Instead, give consideration to specific population characteristics in the study, including age range, gender, morbidities, medications, and other potentially influential factors. For example, a sample frame may not be appropriate to address the target population if a certain group has been used (such as those working for one organisation, or one profession) and the results then inferred to the target population (i.e. working adults). A sample frame may be appropriate when it includes almost all the members of the target population (i.e. a census, or a complete list of participants or complete registry data).

**Item 2: Were study participants recruited in an appropriate way?**	
Yes	Probability sampling method (simple or stratified random) or entire sample (e.g., an entire town) was used
No	Non-probability sampling
Exclude	Sampling method not reported

**Item 3: Was the sample size adequate?**	
Yes	≥599
No	<599

For peer review only

Exclude	Sample size not reported
*Notes	To calculate the required sample size we used an assumed prevalence of 2.5%, which was the global average estimated by the WHO in April, 2020.[2] Based on guidance by the Joanna Briggs Institute and published medical statistical recommendations we selected a precision value that was half the assumed prevalence (1.25%).[1,3] We calculated a minimum sample size of 599 using these inputs:<br>Sample size calculation:<br><br>$n= Z^2 P(1-P)/d^2$<br><br>Where n = sample size;<br>Z = Z statistic for level of confidence (95%);<br>P = expected prevalence (2.5% WHO global estimate);<br>d = precision (1.25%)<br><br>In cases where the sample size calculation was provided and the required sample for 80% power was below our threshold (n<599), this item was marked as yes.

**Item 4: Were the study subjects and setting described in detail?**	
Yes	Average age and distribution of gender/sex provided
No	Neither age or gender/sex is provided, or only one of age and gender/sex is provided

**Item 5: Was data analysis conducted with sufficient coverage of the identified sample?**	
Yes	The demographic characteristics (gender/sex, age, and ethnicity) of the sample is at least somewhat representative of the population
No	The demographic characteristics (gender/sex, age, and ethnicity) of the sample is not representative of the population
Unclear	Information is not provided about demographic characteristics of the sample (gender/sex, age, and ethnicity)

For peer review only

Item 6: Were valid methods used for the identification of the condition?	
Yes	The test used met the FDA standards for Emergency Use Authorizations for COVID-19 serological tests: sensitivity minimum 90%, specificity minimum 95%, as reported in the study.[4]
No	The test used did not meet the FDA standards for Emergency Use Authorizations for COVID-19 serological tests: sensitivity minimum 90%, specificity minimum 95%.
Exclude	Test sensitivity and specificity not reported

Item 7: Was the condition measured in a standard, reliable way for all participants?	
Yes	The same serology test was used for all participants
No	Different serology tests were used for participants
Unclear	No details were provided about which participants received which serology tests

Item 8: Was there appropriate statistical analysis?	
Yes	Does all of the following: corrects for population characteristics or the sample is somewhat representative of the population (probability sampling), corrects for test characteristics), and provides the information necessary to determine the numerator, denominator, prevalence estimate, and confidence interval.
No	Does not correct for population characteristics and the sample is not likely representative of the population (non-probability sampling), does not correct for test or provide the information necessary to correct for test characteristics, or does not provide the information necessary to determine the numerator, denominator, prevalence estimate, and confidence interval.

Item 9: Was the response rate adequate, and if not, was the low response rate managed appropriately?	
Yes	Response rate > 60% or the demographics of the sample were a reasonable match to those of the target population[5]

No	Response rate < 60% and the demographics of the sample were not a reasonable match to those of the target population
Unclear	Response rate not provided and it was unclear if the demographics of the sample differed from the target population

For

**Item 10: Overall risk of bias**	
Low	The estimates are very likely correct for the target population. To obtain a low risk of bias classification, all criteria must be met or departures from the criteria must be minimal and unlikely to impact on the validity and reliability of the prevalence estimate. These include sample sizes that are just below the threshold when all other criteria are met, reporting only some of characteristics of the sample, test characteristics below the threshold but corrections for the test performance, and response rates that are just below the threshold in the context of probability based sampling of an appropriate sampling frame with population weighted seroprevalence estimates.
Moderate	The estimates are likely correct for the target population. To obtain a moderate risk of bias classification, most criteria must be met and departures from the criteria are likely to have only a small impact on the validity and reliability of the prevalence estimates.
High	The estimates are not likely correct for the target population. To obtain a high risk of bias, many criteria must not be met or departures from criteria are likely to have a major impact on the validity and reliability of the prevalence estimates.
Unclear	There was insufficient information to assess the risk of bias.

References

1. Munn Z, Moola S, Lisy K, Riitano D, Tufanaru C. Methodological guidance for systematic reviews of observational epidemiological studies reporting prevalence and cumulative incidence data. Int J Evid Based Healthc. 2015 Sep;13(3):147–53.

2. Boseley S. WHO warns that few have developed antibodies to Covid-19. The Guardian [Internet]. 2020 Apr 20; Available from: https://www.theguardian.com/society/2020/apr/20/studies-suggest-very-few-have-had-covid-19-without-symptoms

3. Naing L, Winn T, Ruslil B. Practical issues in calculating the sample size for prevalence studies. Arch Orofac Sci. 2006;1:9–14.

4. U.S. Food & Drug Administration. Emergency Use Authorization for SARS-CoV-2 Antibody Tests [Internet]. 2020 [cited 2020 May 5]. Available from: https://www.fda.gov/media/137470/download.

5. Morton MBS, Bandara DK, Robinson EM, Carr PEA. In the 21st century, what is an acceptable response rate? Aust N Z J Public Health. 2012 April; 36 (2): 106-8.

For peer review only

BMJ Open

Occupation and SARS-CoV-2 seroprevalence studies: a systematic review

Journal:	*BMJ Open*
Manuscript ID	bmjopen-2022-063771.R1
Article Type:	Original research
Date Submitted by the Author:	04-Nov-2022
Complete List of Authors:	Boucher, Emily; University of Calgary Cumming School of Medicine,<br>Cao, Christian; University of Calgary, Cumming School of Medicine<br>D'Mello, Sean; University of Waterloo<br>Duarte, Nathan; McGill University, Faculty of Engineering<br>Donnici, Claire; University of Calgary, Cumming School of Medicine<br>Duarte, Natalie; University of Toronto, Faculty of Arts and Science<br>Bennett, Graham; McGill University, Department of Economics<br>Consortium, SeroTracker ; University of Calgary<br>Adisesh, Anil; Unity Health Toronto, St. Michael's Hospital; University of Toronto, Division of Occupational Medicine<br>Arora, Rahul; Oxford University, Institute of Biomedical Engineering<br>Kodama, David; Unity Health Toronto, St. Michael's Hospital; University of Toronto Department of Medicine, Division of Emergency Medicine<br>Bobrovitz, Niklas; University of Toronto Temerty Faculty of Medicine; University of Calgary, Department of Critical Care Medicine
**Primary Subject Heading**:	Occupational and environmental medicine
Secondary Subject Heading:	Infectious diseases, Public health
Keywords:	COVID-19, Public health < INFECTIOUS DISEASES, OCCUPATIONAL & INDUSTRIAL MEDICINE

SCHOLARONE™
Manuscripts

BMJ

I, the Submitting Author has the right to grant and does grant on behalf of all authors of the Work (as defined in the below author licence), an exclusive licence and/or a non-exclusive licence for contributions from authors who are: i) UK Crown employees; ii) where BMJ has agreed a CC-BY licence shall apply, and/or iii) in accordance with the terms applicable for US Federal Government officers or employees acting as part of their official duties; on a worldwide, perpetual, irrevocable, royalty-free basis to BMJ Publishing Group Ltd ("BMJ") its licensees and where the relevant Journal is co-owned by BMJ to the co-owners of the Journal, to publish the Work in this journal and any other BMJ products and to exploit all rights, as set out in our licence.

The Submitting Author accepts and understands that any supply made under these terms is made by BMJ to the Submitting Author unless you are acting as an employee on behalf of your employer or a postgraduate student of an affiliated institution which is paying any applicable article publishing charge ("APC") for Open Access articles. Where the Submitting Author wishes to make the Work available on an Open Access basis (and intends to pay the relevant APC), the terms of reuse of such Open Access shall be governed by a Creative Commons licence – details of these licences and which Creative Commons licence will apply to this Work are set out in our licence referred to above.

Other than as permitted in any relevant BMJ Author's Self Archiving Policies, I confirm this Work has not been accepted for publication elsewhere, is not being considered for publication elsewhere and does not duplicate material already published. I confirm all authors consent to publication of this Work and authorise the granting of this licence.

For peer review only

Occupation and SARS-CoV-2 seroprevalence studies: a systematic review

Emily Boucher,[1] Christian Cao[1], Sean D'Mello,[2] Nathan Duarte,[3] Claire Donnici[1], Natalie Duarte,[4] Graham Bennett,[5] SeroTracker Consortium, Anil Adisesh,[6-8] Rahul K. Arora,[1,9] David Kodama,[6,10] Niklas Bobrovitz[11,12]

1. Cumming School of Medicine, University of Calgary, Calgary, AB, Canada
2. Faculty of Engineering, University of Waterloo, Waterloo, ON, Canada
3. Faculty of Engineering, McGill University, Montreal, QC, Canada
4. Faculty of Arts and Science, University of Toronto, ON, Canada
5. Department of Economics, Faculty of Arts, McGill University, Montreal, QC, Canada
6. St. Michael's Hospital, Unity Health Toronto, Toronto, ON, Canada
7. Division of Occupational Medicine, Department of Medicine, University of Toronto, Toronto, ON, Canada
8. Canadian Health Solutions, Saint John, NB, Canada
9. Institute of Biomedical Engineering, University of Oxford, Oxford, UK
10. Division of Emergency Medicine, Department of Medicine, University of Toronto, Toronto, ON, Canada
11. Temerty Faculty of Medicine, University of Toronto, Toronto, ON, Canada
12. Department of Critical Care Medicine, University of Calgary, Calgary, AB, Canada

*Correspondence to Emily Boucher, Cumming School of Medicine, University of Calgary, Calgary, AB, Canada; emily.boucher@ucalgry.ca

Word Count 1179

Key Words Covid-19, Infectious diseases**,** Occupational & industrial medicine

For peer review only

For peer review only - http://bmjopen.bmj.com/site/about/guidelines.xhtml

ABSTRACT

Objective. To describe and synthesize studies of SARS-CoV-2 seroprevalence by occupation prior to the widespread vaccine rollout.

Methods. We identified studies of occupational seroprevalence from a living systematic review (PROSPERO CRD42020183634). Electronic databases, gray literature, and news media were searched for studies published January-December 2020. Seroprevalence estimates and a free text description of the occupation were extracted and classified according to the Standard Occupational Classification (SOC) 2010 system using a machine-learning algorithm. Due to heterogeneity, results were synthesized narratively.

Results. We identified 196 studies including 591,940 participants from 38 countries. Most studies (n=162; 83%) were conducted locally vs regionally or nationally. Sample sizes were generally small (median=220 participants per occupation) and 135 studies (69%) were at a high risk of bias. One or more estimates were available for 21/23 major SOC occupation groups, but over half of the estimates identified (n=359/600) were for healthcare-related occupations. 'Personal Care and Service Occupations' (median 22% [IQR 9-28%]; n=14) had the highest median seroprevalence.

Conclusions. Many seroprevalence studies covering a broad range of occupations were published in the first year of the pandemic. Results suggest considerable differences in seroprevalence between occupations, although few large, high-quality studies were done. Well-designed studies are required to improve our understanding of the occupational risk of SARS-CoV-2 and should be considered as an element of pandemic preparedness for future respiratory pathogens.

For peer review only

Strengths and limitations

- We conducted a comprehensive search of the COVID-19 seroprevalence literature, including non-English articles, government reports, unpublished data.
- Occupations were classified using the Standard Occupational Classification (SOC) 2010 coding system to improve interpretability and facilitate comparison with other datasets.
- Seroprevalence may underestimate the true prevalence of infection because antibody titres decline over time, but where possible we prioritized prevalence estimates for IgG antibodies, which appear to be more robust than other immunoglobulin types.
- We did not adjust for differences in serologic test performance.

For peer review only

For peer review only - http://bmjopen.bmj.com/site/about/guidelines.xhtml

INTRODUCTION

Occupation is a social determinant of health and an important risk factor for SARS-CoV-2 infection. Essential workers in health and social care occupations have an increased risk of COVID-19 compared to non-essential workers, but the risks for other occupations are not well defined.[1-3] Studies examining confirmed COVID-19 cases to examine occupational COVID-19 risk are affected by variable testing rates. For example, testing rates may be higher in workplaces offering testing or paid sick leave, and are impacted by geographic (e.g., urban versus rural) and socio-economic factors (e.g., deprivation), potentially biasing results.[4-6] Few high-quality, prospective studies using frequent, serial PCR or antigen testing covering a broad range of occupations having been conducted, in part due to the costs and administrative burden of such studies.[7,8]

Serologic testing for SARS-CoV-2 antibodies provides evidence of previous infection and/or vaccination depending on vaccination status and the specific antigens targeted and can be used to obtain more accurate estimates of the cumulative incidence of infection.[9] Accurate data on the occupational risks of COVID-19 and other respiratory infections are essential for informing the development of occupational safety guidelines and regulations, transmission control measures and resource allocation (testing, personal protective equipment (PPE), etc.). The objectives of this review were to describe and synthesize studies of SARS-CoV-2 seroprevalence across a broad range of occupations globally prior to the widespread rollout of vaccines.

For peer review only - http://bmjopen.bmj.com/site/about/guidelines.xhtml

METHODS

We identified studies of occupational seroprevalence from a living systematic review (PROSPERO CRD42020183634) of >1000 seroprevalence studies.[10-12] In brief, electronic databases, grey literature, and news media were searched for cohort or cross-sectional studies reporting antibody testing for SARS-CoV-2. Records were screened independently, in duplicate. We restricted eligibility to studies in English, French or that were machine-translatable using Google Translate and published January-December 2020 before vaccines were rolled-out, because differential vaccination rates by occupation would obscure results. We extracted seroprevalence estimates with a free-text description for each occupation. If multiple estimates were reported, the most recent estimate using laboratory-based methods (e.g. ELISA), and anti-spike and/or IgG antibodies were prioritized, because non-IgG and anti-nucleocapsid antibodies may decline more rapidly.[13] Study-level risk of bias was assessed with a modified Joanna Briggs Institute Checklist for Prevalence Studies **(Supplementary File 1)**.[14]

For each seroprevalence estimate, we identified the relevant Standard Occupational Classification (SOC) 2010 codes by applying the National Institute for Occupational Safety & Health (NIOSH) Industry and Occupation Computerized Coding System (NIOCCS) to occupation descriptions.[15] NIOCCS was chosen, because many studies were conducted in the USA. Coding was manually verified if there was insufficient information for NIOCCS classification, or if the probability of correct classification to the six-digit level was <0.8 based on our review of a subset of the NIOCCS coded data (**Supplementary File 1**). Anticipating substantial heterogeneity and an insufficient number of estimates relative to covariates for meta-regression, we planned to summarize data using the median/IQR.

For peer review only - http://bmjopen.bmj.com/site/about/guidelines.xhtml

Patient and Public Involvement: It was not possible or appropriate to involve patients or the public in this study.

RESULTS

We identified 196 studies of occupational seroprevalence conducted in 2020 during the first and second waves of the pandemic **(Figure 1)**. There were 591,940 participants from 38 countries, including the USA (n=44 studies), UK (n=16) and Italy (n=15). Most studies (n=162; 83%) were conducted locally (e.g. city, county) as opposed to regionally (e.g. state; n=20; 10%) or nationally (n=14; 7%). Most were restricted to one occupational group (n=103), limiting direct comparisons (i.e. using the same reference group). Sample sizes were often small (median=220, IQR 64-568 participants). Overall, 135 studies (69%) were at a high risk of bias, 47 moderate (24%), 2 low (1%) and 12 unclear (6%).. Common reasons for bias were inadequate statistical analysis (i.e. no adjustment for test or sample characteristics; 92%), non-probability sampling (74%), and small sample-size (46%).

At least one estimate was available for all 23 major SOC occupation groups, except for 'Legal' and 'Military-Specific' occupations **(Figure 2**; all studies**)**. Over half of the 600 estimates identified (n=359) were for healthcare-related occupations. For SOC groups with three or more estimates, the highest median seroprevalence was reported for 'Personal Care and Service Occupations' (median 22% [IQR 9-28%]; n=14, e.g. 'Personal Care Aids'). The next highest was reported for 'Building and Grounds Cleaning and Maintenance' occupations (11% [3-22%]; n=17, e.g. 'Maids and Housekeeping Cleaners'), and 'Healthcare Support' (11% [2-20%]; n=39, e.g. 'Nursing Assistants') occupations. The lowest median seroprevalence was 1% (0-11%; n=6,

For peer review only - http://bmjopen.bmj.com/site/about/guidelines.xhtml

e.g. 'Athletes') for 'Arts, Design, Entertainment, Sports, and Media Occupations.' Individual estimates are listed in **Supplementary File 2**.

DISCUSSION

This review is the first comprehensive synthesis of occupational COVID-19 seroprevalence studies world-wide. We identified 196 studies representing 21 out of 23 major SOC groups conducted during the first and second waves of the SARS-CoV-2 pandemic in 2020, prior to the widespread rollout of vaccines, and described occupational groups with high seroprevalence.

Seroprevalence studies may estimate the cumulative incidence of infection more accurately than diagnostic testing studies when access to testing and test performance are poor, and also can identify asymptomatic infections.[6,8] The data identified suggest considerable differences in seroprevalence by occupation, though we did not statistically test for differences due to considerable variation in geography, study dates and workplace determinants of infection (e.g. PPE, ventilation). 'Caring and Personal Service' occupations had the highest median seroprevalence (22%), which was four-times higher than the unemployed (5%) and median seroprevalence across all occupational groups (5%). The UK Office for National Statistics reported a slightly lower cumulative incidence for positive diagnostic or rapid tests for COVID-19 across 25 occupational groups of 4% (mean),[4] but the discrepancy between the true cumulative incidence and confirmed infections is likely greater in regions with less access to testing: national, population-based serosurveys have estimated there are 10-20 serologically identifiable cases per one confirmed case.[12]

For peer review only - http://bmjopen.bmj.com/site/about/guidelines.xhtml

In future pandemics, large, well-reported, high-quality seroprevalence studies across a broad range of occupations are needed at an early stage to inform appropriate workplace policy. It has been suggested that 20% of the US workforce was exposed to disease or infection at work at least once a month prior to the pandemic.[16] Accurate data on the occupational risks of respiratory infections, including SARS-CoV-2 are needed to inform understanding of transmission, occupational health and safety agency guidelines and allocation of resources (e.g., personal protective equipment and vaccines) during outbreaks and pandemics. For governments, there are also issues of occupational disease recognition and compensation to be considered.

As such, future population-based studies on respiratory infections should collect data on occupation. In the case of epidemic infection, collaboration between academic centres with the capacity to conduct large-scale studies and government agencies with expertise in disease surveillance and access to workplace data (e.g., public health, occupational health and safety) may be beneficial.[12] Other authors have suggested the utility of occupational surveillance systems.[17] However, the routine completion of the occupation field in electronic health records would also serve this purpose as well as informing patient reported outcome measures.

Strengths and Limitations

Despite the large number of studies of occupational seroprevalence conducted, many studies had methodological limitations. Only two studies were at a low risk of bias and most occupational subgroups had small sample sizes (median 220 participants). Many were limited to one major SOC group (n=103 studies), which precluded comparisons. Detailed descriptions of occupations were often lacking, potentially contributing to coding errors and misclassification, and workplace determinants of infection (e.g. use of PPE) were poorly reported.

For peer review only - http://bmjopen.bmj.com/site/about/guidelines.xhtml

134

135 In conclusion, our review shows that a large number of seroprevalence studies covering a broad
136 range of occupations were published in the first year of the pandemic. Results suggest
137 considerable differences in seroprevalence between occupations, although few large, well-
138 reported, high-quality studies were done. Carefully-designed, adequately powered
139 seroprevalence studies with coverage of a broad range of occupations could improve our
140 understanding of the occupational risk of SARS-CoV-2 and other respiratory infections and
141 should be considered an element of pandemic preparedness and response.

142

143

For peer review only

Funding Statement

SeroTracker receives funding for SARS-CoV-2 seroprevalence study evidence synthesis from the Public Health Agency of Canada through Canada's COVID-19 Immunity Task Force (Grant Number 2021-HQ-000056), the World Health Organization Health Emergencies Programme, the Robert Koch Institute, and the Canadian Medical Association Joule Innovation Fund. No funding source had any role in the design of this study, its execution, analyses, interpretation of the data, or decision to submit results. This manuscript does not necessarily reflect the views of the World Health Organization or any other funder.

Statement of author's contributions

This secondary analysis of the SeroTracker database was conceived by NB, EB, DK and AA. Senior authors on this paper were NB, DK, RA and AA. The protocol was developed by EB, NB and DK. Data cleaning was performed by CC, CD, NaD, SD and EB and verification by EB, SD, ND and GB. Analysis was performed by EB and RA. The first draft of the manuscript was written by EB and revised by EB, RA, NB, ND, GB, SD, CC, AA, DK. The SeroTracker Consortium maintained the living systematic review database used in the study. All authors reviewed and agreed to the findings, and also provided critical revisions to the paper.

Disclosure of potential and actual conflicts of interest

RKA was previously a Technical Consultant for the Bill and Melinda Gates Foundation Strategic Investment Fund, is a minority shareholder of Alethea Medical, and was a former Senior Policy Advisor at Health Canada. Each of these relationships is unrelated to the present work.

For peer review only - http://bmjopen.bmj.com/site/about/guidelines.xhtml

JP reports grants to his institution from MedImmune, Sanofi Pasteur, Merck and AbbVie, and personal fees for lectures from AbbVie and Astra-Zeneca, all outside of the submitted work.

MPC reports grants from McGill Interdisciplinary Initiative in Infection and Immunity, grants from Canadian Institutes of Health Research, during the conduct of the study; personal fees from GEn1E Lifesciences, personal fees from nplex biosciences, personal fees from Kanvas biosciences, personal fees from AstraZeneca, non-financial support from Cidara therapeutics, non-financial support from Scynexis, Inc., non-financial support from Amplyx Pharmaceutics, outside the submitted work. In addition, MPC has a patent for methods detecting tissue damage, graft versus host disease, and infections using cell-free DNA profiling pending, a patent for methods assessing the severity and progression of SARS-CoV-2 infections using cell-free DNA pending, a patent for rapid identification of antimicrobial resistance and other microbial phenotypes using highly-multiplexed fluorescence in situ hybridization pending, and a patent highly multiplexed detection of gene expression with hybridization chain reaction pending, all outside the submitted work.

Ethics approval: Not applicable. This study did not involve human participants or animals.

Dating sharing: Seroprevalence data can be downloaded (or requested) from https://serotracker.com.

For peer review only

REFERENCES

1. Magnusson K, Nygard KM, Vold L, Telle KE. Occupational risk of COVID-19 in the 1st vs 2nd wave of infection. medRxiv. 2020 Jan 1.

2. Mutambudzi M, Niedwiedz C, Macdonald EB, Leyland A, Mair F, Anderson J, Celis-Morales C, Cleland J, Forbes J, Gill J, Hastie C. Occupation and risk of severe COVID-19: prospective cohort study of 120 075 UK Biobank participants. Occupational and Environmental Medicine. 2021 May 1;78(5):307-14.

3. Nguyen LH, Drew DA, Graham MS, Joshi AD, Guo CG, Ma W, Mehta RS, Warner ET, Sikavi DR, Lo CH, Kwon S. Risk of COVID-19 among front-line health-care workers and the general community: a prospective cohort study. The Lancet Public Health. 2020 Sep 1;5(9):e475-83.

4. Seo E, Mun E, Kim W, Lee C. Fighting the COVID-19 pandemic: onsite mass workplace testing for COVID-19 in the Republic of Korea. Annals of occupational and environmental medicine. 2020;32.

5. Tan TQ, Kullar R, Swartz TH, Mathew TA, Piggott DA, Berthaud V. Location matters: geographic disparities and impact of coronavirus disease 2019. The Journal of infectious diseases. 2020 Dec 15;222(12):1951-4.

6. Duarte N, D'Mello S, Duarte NA, Rocco S, Van Wyk J, Pillai AA, Liu M, Williamson T, Arora RK. Uptake of SARS-CoV-2 workplace testing programs, March 2020 to March 2021. medRxiv. 2021 Jan 1.

7. Office for National Statistics. Coronavirus (COVID-19) Infection Survey: characteristics of people testing positive for COVID-19 in England. 2021 Feb 22. Available from: https://www.ons.gov.uk/peoplepopulationandcommunity/healthandsocialcare/conditionsanddiseases/articles/coronaviruscovid19infectionsinthecommunityinengland/characteristicsofpeopletestingpositiveforcovid19inengland22february2021

8. Pearce N, Rhodes S, Stocking K, Pembrey L, van Veldhoven K, Brickley EB, Robertson S, Davoren D, Nafilyan V, Windsor-Shellard B, Fletcher T. Occupational differences in COVID-19 incidence, severity, and mortality in the United Kingdom: Available data and framework for analyses. Wellcome open research. 2021;6.

9. Duarte N, Yanes-Lane M, Arora RK, Bobrovitz N, Liu M, Bego MG, Yan T, Cao C, Gurry C, Hankins CA, Cheng MP. Adapting Serosurveys for the SARS-CoV-2 Vaccine Era. Open Forum Infect Dis. 2021 Dec 23;9(2):ofab632.

For peer review only - http://bmjopen.bmj.com/site/about/guidelines.xhtml

10. Arora RK, Joseph A, Van Wyk J, Rocco S, Atmaja A, May E, et al.. SeroTracker: a global SARS-CoV-2 seroprevalence dashboard. The Lancet Infectious Diseases. The Lancet Infectious Diseases; 2021;21(4):e75–6.

11. [dataset] SeroTracker Consortium. Data from: Our Data. November 7, 2021. https://serotracker.com/data

12. Bobrovitz N, Arora RK, Cao C, Boucher E, Liu M, Donnici C, Yanes-Lane M, Whelan M, Perlman-Arrow S, Chen J, Rahim H. Global seroprevalence of SARS-CoV-2 antibodies: a systematic review and meta-analysis. PloS one. 2021 Jun 23;16(6):e0252617.

13. Isho B, Abe KT, Zuo M, Jamal AJ, Rathod B, Wang JH, et al. Persistence of serum and saliva antibody responses to SARS-CoV-2 spike antigens in COVID-19 patients. Sci Immunol. 2020 Oct 8;5(52):eabe5511.

14. Munn Z, Moola S, Lisy K, Riitano D, Tufanaru C. Methodological guidance for systematic reviews of observational epidemiological studies reporting prevalence and incidence data. Int J Evid Based Healthc. 2015;13(3):147–153.

15. NIOSH (2021). NIOSH Industry and Occupation Computerized Coding System (NIOCCS). U.S. Department of Health and Human Services, Public Health Service, Centers for Disease Control and Prevention, National Institute for Occupational Safety and Health, Division of Field Studies & Engineering, Health Informatics Branch. https://csams.cdc.gov/nioccs/About.aspx. Date accessed Sept 1, 2021.

16. Baker MG, Peckham TK, Seixas NS. Estimating the burden of United States workers exposed to infection or disease: a key factor in containing risk of COVID-19 infection. PloS one. 2020 Apr 28;15(4):e0232452.

17. Marinaccio A, Boccuni F, Rondinone BM, Brusco A, D'Amario S, Iavicoli S. Occupational factors in the COVID-19 pandemic in Italy: compensation claims applications support establishing an occupational surveillance system. Occupational and Environmental Medicine. 2020 Dec 1;77(12):818-21.

For peer review only - http://bmjopen.bmj.com/site/about/guidelines.xhtml

Figure Legends

Figure 1. PRISMA flow diagram

Figure 2. Seroprevalence by SOC 2010 major occupation group. *Estimates are a mix of 'Healthcare Practitioners and Technical Occupations' and 'Healthcare Support Occupations' (see next page)

Supplementary File 1. Supplementary methods

Supplementary File 2. Summary of included studies and references

For peer review only

For peer review only - http://bmjopen.bmj.com/site/about/guidelines.xhtml

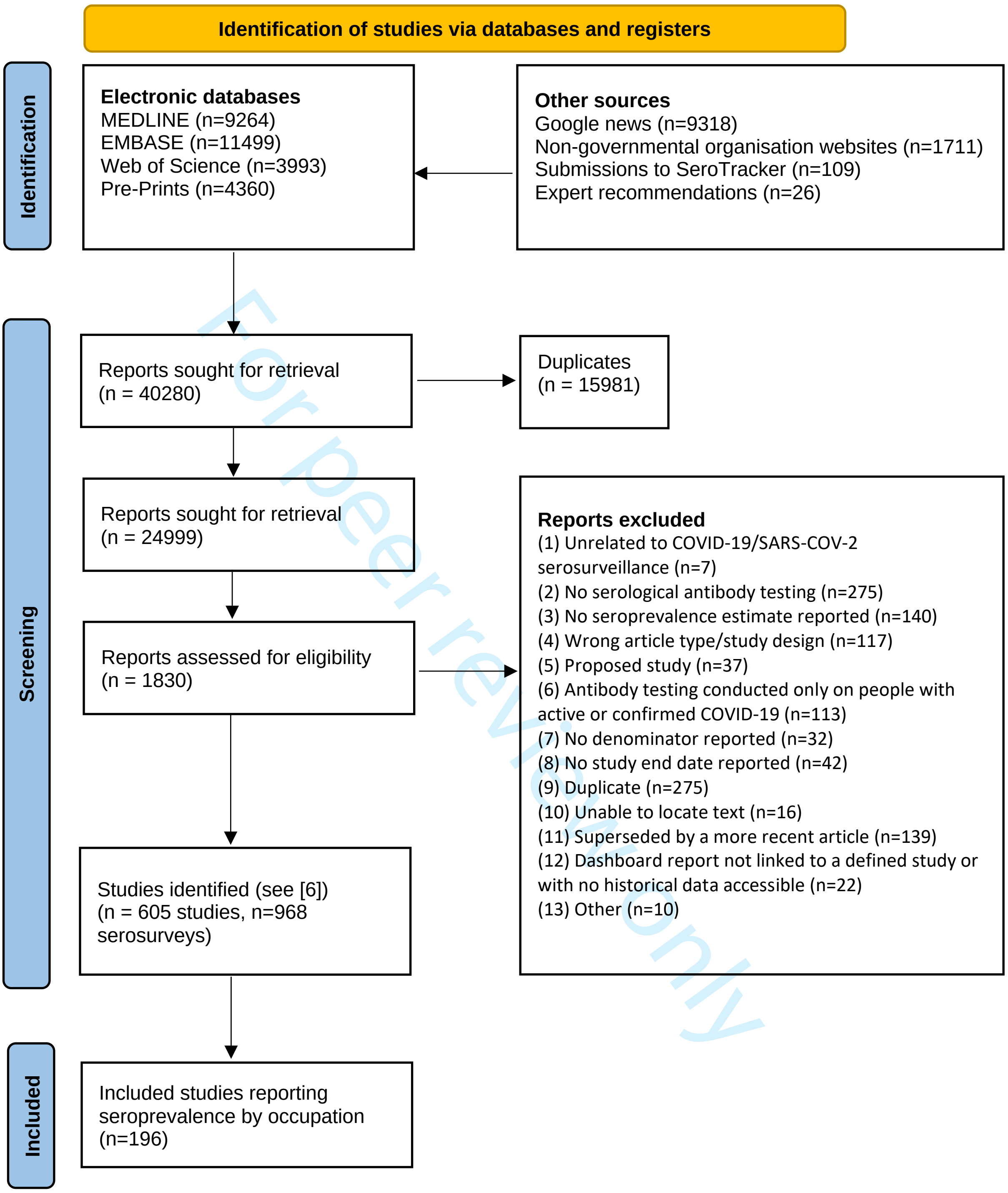

From: Page MJ, McKenzie JE, Bossuyt PM, Boutron I, Hoffmann TC, Mulrow CD, et al. The PRISMA 2020 statement: an updated guideline for reporting systematic reviews. BMJ 2021;372:n71. doi: 10.1136/bmj.n71

For more information, visit: http://www.prisma-statement.org/

For peer review only

SOC 2010 Major Occupation Group	Total Estimates	Total Countries	Median, IQR Study dates, midpoint	Median, IQR Sample size	Seroprevalence % (Median, IQR)	Seroprevalence % (Scale 0-75%)	N, % Low-Moderate RoB
Architecture and Engineering Occupations (17-0000)	1	1	15/08 (15/08-15/08)	21 (21-21)	42.9 (42.9-42.9)		0 (0%)
Personal Care and Service Occupations (39-0000)	14	7	03/05 (02/04-02/06)	127 (54-302)	21.5 (9.32-27.76)		3 (21%)
Installation, Maintenance, and Repair Occupations (49-0000)	1	1	19/06 (19/06-19/06)	134 (134-134)	16.4 (16.4-16.4)		0 (0%)
Building and Grounds Cleaning and Maintenance Occupations (37-0000)	17	8	13/07 (09/06-16/08)	102 (42-226)	10.8 (3.3-21.7)		6 (35%)
Healthcare Support Occupations (31-0000)	39	12	05/06 (19/05-21/06)	263 (122-562)	10.7 (2-20.05)		12 (31%)
Business and Financial Operations Occupations (13-0000)	2	2	05/07 (18/06-22/07)	462 (252-671)	8.27 (5.3-11.23)		2 (100%)
Management Occupations (11-0000)	10	6	17/06 (01/05-02/08)	44 (23-145)	8.17 (6.7-19.93)		3 (30%)
Food Preparation and Serving Related Occupations (35-0000)	6	4	17/06 (11/05-23/07)	58 (12-108)	6.35 (2.37-24.03)		2 (33%)
Healthcare Practitioners and Technical Occupations (29-0000)	222	23	13/06 (13/05-13/07)	215 (64-482)	5.91 (1.83-11.71)		84 (38%)
*Healthcare Practitioners and Technical Occupations, 5-digit codes***							
Miscellaneous Health Technologists and Technicians	4	3	26/08 (09/08-12/09)	60 (20-121)	12.96 (9.09-27.54)		1 (25%)
Registered Nurses	78	18	05/06 (05/05-05/07)	329 (71-1000)	8.44 (3.68-15.5)		22 (28%)
Clinical Laboratory Technologists and Technicians	18	12	15/06 (19/05-11/07)	204 (86-284)	6.22 (2.07-11.94)		12 (67%)
Physicians and Surgeons	65	21	09/06 (10/05-09/07)	214 (59-564)	5.88 (1.85-11.8)		23 (35%)
Emergency Medical Technicians and Paramedics	9	6	13/06 (27/05-30/06)	157 (56-243)	5.41 (5.2-11)		4 (44%)
Therapists	15	4	08/06 (19/05-28/06)	121 (61-235)	3.75 (0-9.45)		7 (47%)
Physician Assistants	9	2	27/06 (26/05-28/07)	230 (156-320)	3.48 (0.64-9.43)		3 (33%)
Pharmacists	9	7	29/06 (14/06-14/07)	113 (29-213)	0.5 (0-3.45)		4 (44%)
Healthcare Occupations (mixed)*	94	25	05/06 (29/04-12/07)	375 (110-1012)	5.66 (2.35-11.6)		23 (24%)
Sales and Related Occupations (41-0000)	23	8	21/08 (22/06-19/10)	643 (236-1184)	5.3 (1.2-8.8)		6 (26%)
Education, Training, and Library Occupations (25-0000)	6	5	05/07 (12/06-27/07)	238 (73-1305)	5.07 (2.71-17.22)		3 (50%)
Farming, Fishing, and Forestry Occupations (45-0000)	3	3	13/07 (25/06-30/07)	80 (66-100)	5 (2.5-5)		1 (33%)
Not employed (mixed)*	37	14	23/06 (12/05-04/08)	382 (116-905)	4.9 (2.7-14.97)		28 (76%)
Office and Administrative Support Occupations (43-0000)	39	18	14/06 (18/05-11/07)	120 (32-522)	4.88 (1.36-13.36)		20 (51%)
First responders (mixed)*	6	1	18/05 (13/05-22/05)	219 (72-599)	4.67 (1.6-7.34)		1 (17%)
Community and Social Service Occupations (21-0000)	6	2	30/05 (18/05-11/06)	104 (49-188)	4.45 (2.13-6.1)		1 (17%)
Protective Service Occupations (33-0000)	28	9	04/07 (21/05-16/08)	190 (46-555)	4.29 (2.17-7.47)		6 (21%)
Transportation and Material Moving Occupations (53-0000)	23	7	08/08 (08/06-08/10)	230 (80-364)	3.5 (1.8-11.8)		8 (35%)
Life, Physical, and Social Science Occupations (19-0000)	11	7	06/07 (11/06-30/07)	343 (174-570)	2.6 (1.66-6.46)		4 (36%)
Production Occupations (51-0000)	4	3	23/05 (26/04-19/06)	764 (342-1132)	1.52 (1.45-4.93)		2 (50%)
Arts, Design, Entertainment, Sports, and Media Occupations (27-0000)	6	5	07/07 (04/06-09/08)	164 (47-823)	1.39 (0.18-11.02)		3 (50%)
Computer and Mathematical Occupations (15-0000)	1	1	03/05 (03/05-03/05)	47 (47-47)	0 (0-0)		1 (100%)
Construction and Extraction Occupations (47-0000)	1	1	03/05 (03/05-03/05)	42 (42-42)	0 (0-0)		1 (100%)

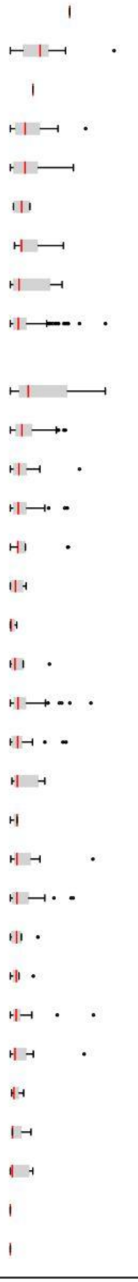

S1 Materials

Table of Contents

Supplementary files **2**
S1 File. PRISMA checklist 2
S2 File. Search strategy 4
S3 File. Tool for assessing study risk of bias 8
S4 File. Details of occupational coding 11
References for supplementary files **13**

For peer review only

Supplementary files

S1 File. PRISMA checklist

Section/topic	#	Checklist item	Reported on page #
**TITLE**			
Title	1	Identify the report as a systematic review, meta-analysis, or both.	0
**ABSTRACT**			
Structured summary	2	Provide a structured summary including, as applicable: background; objectives; data sources; study eligibility criteria, participants, and interventions; study appraisal and synthesis methods; results; limitations; conclusions and implications of key findings; systematic review registration number.	1
**INTRODUCTION**			
Rationale	3	Describe the rationale for the review in the context of what is already known.	3, lines 14-30
Objectives	4	Provide an explicit statement of questions being addressed with reference to participants, interventions, comparisons, outcomes, and study design (PICOS).	3, line 30-32
**METHODS**			
Protocol and registration	5	Indicate if a review protocol exists, if and where it can be accessed (e.g., Web address), and, if available, provide registration information including registration number.	3, line 39
Eligibility criteria	6	Specify study characteristics (e.g., PICOS, length of follow-up) and report characteristics (e.g., years considered, language, publication status) used as criteria for eligibility, giving rationale.	4, lines 39-45
Information sources	7	Describe all information sources (e.g., databases with dates of coverage, contact with study authors to identify additional studies) in the search and date last searched.	4, lines 39-40
Search	8	Present full electronic search strategy for at least one database, including any limits used, such that it could be repeated.	Suppl. File 2
Study selection	9	State the process for selecting studies (i.e., screening, eligibility, included in systematic review, and, if applicable, included in the meta-analysis).	4, lines 41-43
Data collection process	10	Describe method of data extraction from reports (e.g., piloted forms, independently, in duplicate) and any processes for obtaining and confirming data from investigators.	4, lines 41-49, 57-58
Data items	11	List and define all variables for which data were sought (e.g., PICOS, funding sources) and any assumptions and simplifications made.	4, lines 44-45 (see reference to previous study)
Risk of bias in individual studies	12	Describe methods used for assessing risk of bias of individual studies (including specification of whether this was done at the study or outcome level), and how this information is to be used in any data synthesis.	4, see reference and Suppl. File 1
Summary measures	13	State the principal summary measures (e.g., risk ratio, difference in means).	4, lines 57-78
Synthesis of results	14	Describe the methods of handling data and combining results of studies, if done, including measures of consistency (e.g., I^2) for each meta-analysis.	4, lines 57-58
Risk of bias across studies	15	Specify any assessment of risk of bias that may affect the cumulative evidence (e.g., publication bias, selective reporting within studies).	4, lines 47-48
Additional analyses	16	Describe methods of additional analyses (e.g., sensitivity or subgroup analyses, meta-regression), if done, indicating which were pre-specified.	NA
**RESULTS**			
Study selection	17	Give numbers of studies screened, assessed for eligibility, and included in the review, with reasons for exclusions at each stage, ideally with a flow diagram.	Suppl File 1
Study characteristics	18	For each study, present characteristics for which data were extracted (e.g., study size, PICOS, follow-up period) and provide the citations.	Suppl. File 2
Risk of bias within studies	19	Present data on risk of bias of each study and, if available, any outcome level assessment (see item 12).	Suppl. File 2
Results of individual studies	20	For all outcomes considered (benefits or harms), present, for each study: (a) simple summary data for each intervention group (b) effect estimates and confidence intervals, ideally with a forest plot.	Suppl. File 2
Synthesis of results	21	Present results of each meta-analysis done, including confidence intervals and measures of consistency.	NA – see narrative synthesis on page 5 & Figure 1
Risk of bias across studies	22	Present results of any assessment of risk of bias across studies (see Item 15).	5, lines 72-75 Figure 1
Additional analysis	23	Give results of additional analyses, if done (e.g., sensitivity or subgroup analyses, meta-regression [see Item 16]).	NA
**DISCUSSION**			
Summary of evidence	24	Summarize the main findings including the strength of evidence for each main outcome; consider their relevance to key groups (e.g., healthcare providers, users, and policy makers).	6, lines 110-118

For peer review only - http://bmjopen.bmj.com/site/about/guidelines.xhtml

Limitations	25	Discuss limitations at study and outcome level (e.g., risk of bias), and at review-level (e.g., incomplete retrieval of identified research, reporting bias).	6, lines 131-136
Conclusions	26	Provide a general interpretation of the results in the context of other evidence, and implications for future research.	6, lines 119-120
**FUNDING**			
Funding	27	Describe sources of funding for the systematic review and other support (e.g., supply of data); role of funders for the systematic review.	9

14

For peer review only

S2 File. Search strategy

Database: Ovid MEDLINE(R) and Epub Ahead of Print, In-Process & Other Non-Indexed Citations and Daily
Dates: January 1, 2020 to December 31, 2020
Notes: Covid-19 search terms were adapted from Ovid Expert Searches

#	Search terms
1	exp Coronavirus/
2	exp Coronavirus Infections/
3	(coronavirus* or corona virus* or OC43 or NL63 or 229E or HKU1 or HCoV* or ncov* or covid* or sars-cov* or sarscov* or Sars-coronavirus* or Severe Acute Respiratory Syndrome Coronavirus*).tw,kf.[EB2]
4	or/1-3
5	4 not ((MERS or MERS-CoV or Middle East respiratory syndrome or camel* or dromedar* or equine or coronary or coronal or covidence* or covidien or influenza virus or HIV or bovine or calves or TGEV or feline or porcine or BCoV or PED or PEDV or PDCoV or FIPV or FCoV or SADS-CoV or canine or CCov or zoonotic or avian influenza or H1N1 or H5N1 or H5N6 or IBV).mp. or (animals/ not humans/))
6	((pneumonia or covid* or coronavirus* or corona virus* or ncov* or 2019-ncov or sars* or virus).tw,kf. or exp pneumonia/) and Wuhan.tw,kf.
7	(2019-ncov* or 2019nCov* or ncov19 or ncov-19 or 2019-novel CoV or sars-cov2* or sars-cov-2* or sarscov2* or sarscov-2* or Sars-coronavirus2 or Sars-coronavirus-2 or SARS-like coronavirus* or coronavirus 2 or coronavirus2* or corona or coronavirus-19 or covid19 or covid-19 or covid 2019 or ((novel or new or nouveau) adj2 (CoV or nCoV or covid or coronavirus* or corona virus or Pandemi*2)) or ((covid or covid19* or covid-19) and pandemic*2) or (coronavirus* and pneumonia)).tw,kf.
8	COVID-19.rx,px,ox. or severe acute respiratory syndrome coronavirus 2.os.
9	or/6-8
10	5 or 9
11	immunoglobulins/ or antibodies/ or antibodies, blocking/ or exp antibodies, neutralizing/ or antibodies, viral/ or antigen-antibody complex/ or immune sera/ or exp immunoglobulin isotypes/ or immunoglobulin a/ or immunoglobulin d/ or immunoglobulin e/ or immunoglobulin g/ or immunoglobulin m/
12	serologic tests/ or complement fixation tests/ or hemagglutination inhibition tests/ or neutralization tests/
13	immunoassay/ or fluoroimmunoassay/ or exp immunoblotting/ or immunoenzyme techniques/ or exp enzyme-linked immunosorbent assay/ or exp enzyme-linked immunospot assay/ or immunosorbent techniques/ or serologic tests/ or complement fixation tests/ or hemagglutination inhibition tests/ or neutralization tests/ or Serology/di
14	(enzyme linked immunosorbent or enzyme-linked immunosorbent or ELISA or immunofluorescence or complement fixation or hemagglutination inhibition or immunoblot or western blot or neutrali*).tw,kf.
15	(antibod* or immunoglobulin* or immune globulin* or titer* or isotype* or IgG or IgM or IgA or neutrali* or sera or serum or serolog* or saliva).tw,kf.
16	or/11-14
17	seroepidemiologic studies/
18	incidence/ or prevalence/
19	(seroconver* or seroprevalence or sero-prevalence or seroincidence or sero-incidence or seroepidemiolog* or sero-epidemiolog*).mp.
20	(inciden* or prevalen* or count* or rate*).mp.
21	(serosurvey or sero-survey or screen* or diagnostic).mp.
22	(seroconver* or seroprevalence or sero-prevalence or seroincidence or sero-incidence or seroepidemiolog* or sero-epidemiolog* or inciden* or prevalen* or silent or asymptomatic or serosurvey or sero-survey).tw,kf.
23	or/17-21
24	10 and (16 and 23)
25	10 and 15
26	10 and 22
27	or/24-26
28	limit 27 to yr="2020-Current"
29	remove duplicates from 28

For peer review only - http://bmjopen.bmj.com/site/about/guidelines.xhtml

Database: Embase
Dates: January 1, 2020 to December 31, 2020
Notes: Covid-19 search terms were adapted from Ovid Expert Searches

#	Searches
1	exp Coronavirus/
2	exp Coronavirus Infections/
3	(coronavirus* or corona virus* or OC43 or NL63 or 229E or HKU1 or HCoV* or ncov* or covid* or sars-cov* or sarscov* or Sars-coronavirus* or Severe Acute Respiratory Syndrome Coronavirus*).tw,kw.
4	or/1-3
5	4 not ((MERS or MERS-CoV or Middle East respiratory syndrome or camel* or dromedar* or equine or coronary or coronal or covidence* or covidien or influenza virus or HIV or bovine or calves or TGEV or feline or porcine or BCoV or PED or PEDV or PDCoV or FIPV or FCoV or SADS-CoV or canine or CCov or zoonotic or avian influenza or H1N1 or H5N1 or H5N6 or IBV).mp. or (animals/ not humans/))
6	((pneumonia or covid* or coronavirus* or corona virus* or ncov* or 2019-ncov or sars*).tw,kw. or exp pneumonia/) and Wuhan.tw,kw.
7	(2019-ncov or ncov19 or ncov-19 or 2019-novel CoV or sars-cov2 or sars-cov-2 or sarscov2 or sarscov-2 or Sars-coronavirus2 or Sars-coronavirus-2 or SARS-like coronavirus* or coronavirus-19 or covid19 or covid-19 or covid 2019 or ((novel or new or nouveau) adj2 (CoV or nCoV or covid or coronavirus* or corona virus or Pandemi*2)) or ((covid or covid19 or covid-19) and pandemic*2) or (coronavirus* and pneumonia)).tw,kw.
8	(coronavirus disease 2019 or severe acute respiratory syndrome coronavirus 2).sh,dj.
9	6 or 7 or 8
10	5 or 9
11	virus antibody/ec [Endogenous Compound]
12	neutralizing antibody/ec [Endogenous Compound]
13	exp immunoglobulin/ or exp immunoglobulin A antibody/ or exp immunoglobulin class/ or exp immunoglobulin M antibody/ or exp immunoglobulin G antibody/ or exp immunoglobulin antibody/
14	11 or 12 or 13
15	serology/
16	serodiagnosis/ or complement fixation test/ or hemagglutination inhibition test/ or hemolytic plaque assay/
17	fluorescent antibody technique/
18	immunofluorescence test/ or viral disease immunofluorescence assay/
19	enzyme linked immunosorbent assay/
20	western blotting/
21	(enzyme linked immunosorbent or enzyme-linked immunosorbent or ELISA or immunoassay or immunofluorescence or fluorescent antibody or complement fixation or hemagglutination inhibition or hemolytic plaque assay or immunoblot or western blot or neutrali*).tw,kw.
22	(antibod* or immunoglobulin* or immune globulin* or titer* or isotype* or IgG or IgM or IgA or neutrali* or sera or serolog* or serum or saliva).tw,kw.
23	15 or 16 or 17 or 18 or 19 or 20 or 21
24	14 or 23
25	exp seroepidemiology/
26	*prevalence/
27	*incidence/
28	(seroconver* or seroprevalence or sero-prevalence or seroincidence or sero-incidence or seroepidemiolog* or sero-epidemiolog* or inciden* or prevalen* or count* or rate* or serosurvey or sero-survey or screen* or diagnostic).mp.
29	(seroconver* or seroprevalence or sero-prevalence or seroincidence or sero-incidence or seroepidemiolog* or sero-epidemiolog* or inciden* or prevalen* or silent or asymptomatic or serosurvey or sero-survey).tw,kw.
30	25 or 26 or 27 or 28
31	10 and (24 and 30)
32	10 and 22
33	10 and 29
34	31 or 32 or 33
35	limit 34 to yr="2020-Current"
36	remove duplicates from 35

For peer review only

Database: Web of Science Core Collection
Date: January 1, 2020 to December 31, 2020

#	Searches
1	TS=(coronavirus* or corona virus* or OC43 or NL63 or 229E or HKU1 or HCoV* or ncov* or covid* or sars-cov* or sarscov* or Sars-coronavirus* or Severe Acute Respiratory Syndrome Coronavirus*)
2	TS=(MERS or MERS-CoV or Middle East respiratory syndrome or camel* or dromedar* or equine or coronary or coronal or covidence* or covidien or influenza virus or HIV or bovine or calves or TGEV or feline or porcine or BCoV or PED or PEDV or PDCoV or FIPV or FCoV or SADS-CoV or canine or CCov or zoonotic or avian influenza or H1N1 or H5N1 or H5N6 or IBV)
3	#1 NOT #2
4	TS=((pneumonia or covid* or coronavirus* or corona virus* or ncov* or 2019-ncov or sars* or virus) AND Wuhan)
5	TS=(2019-ncov* or 2019nCov* or ncov19 or ncov-19 or 2019-novel CoV or sars-cov2* or sars-cov-2* or sarscov2* or sarscov-2* or Sars-coronavirus2 or Sars-coronavirus-2 or SARS-like coronavirus* or corona or coronavirus-19 or covid19 or covid-19 or covid 2019 or ((novel or new or nouveau) adj2 (CoV or nCoV or covid or coronavirus*)) or (coronavirus* and pneumonia)).
6	TS=(COVID-19 or "severe acute respiratory syndrome coronavirus")
7	#6 OR #5 OR #4 OR #3
8	TS=(antibod* or immunoglobulin* or immune globulin* or titer* or isotype* or IgG or IgM or IgA or neutralization or sera or serolog* or saliva or serum).
9	TS=("enzyme linked immunosorbent assay" or "enzyme-linked immunosorbent assay" or "immunoenzyme" or ELISA or "lateral flow immunoassay" or LFIA or "immunofluorescence assay" or immunochromatography or "complement fixation test" or "hemagglutination inhibition" or immunoblot or "western blot" or "neutralization assay")
10	#9 OR #8
11	TI=(seroconversion or seroprevalence or seroincidence or seroepidemiolog* or incidence or prevalence or asymptomatic or sero-survey*) or AK=(seroconversion or seroprevalence or seroincidence or seroepidemiolog* or incidence or prevalence or asymptomatic or sero-survey*)
12	ALL=(prevalence or incidence or seroconversion or seroconvert or seroprevalence or seroincidence or seroepidemiolog* or serosurvey or sero-survey or survey or screen* or diagnostic test)
13	#12 AND #10 AND #7
14	#11 AND #7
15	TI=(antibod* or immunoglobulin* or immune globulin* or titer* or isotype* or IgG or IgM or IgA or neutralization or sera or serolog* or saliva or serum).
16	#15 AND #7
17	#16 OR #14 OR #13

Database: Europe PMC [Secondary search for pre-prints]
Dates: January 1, 2020 to December 31, 2020

#	Searches
	("2019-nCoV" OR "2019nCoV" OR "COVID-19" OR "SARS-CoV-2" OR "COVID19" OR "COVID" OR "SARS-nCoV" OR ("wuhan" AND "coronavirus") OR "Coronavirus" OR "Corona virus" OR "corona-virus" OR "corona viruses" OR "coronaviruses" OR "SARS-CoV" OR "Severe Acute Respiratory Syndrome Coronavirus" OR ("SARS" AND "coronavirus")) AND ABSTRACT:(sera* OR sero* OR immun* OR Ig* OR “enzyme-linked immunosorbent assay” OR ELISA OR “neutralization assay" OR seroprevalence) AND (SRC:"PPR")

For peer review only - http://bmjopen.bmj.com/site/about/guidelines.xhtml

Sources: Health organizations
Dates: January 1, 2020 to December 31, 2020

Source	Search strategy	
WHO Situation Reports	1	“antibod”, “sero”, “immun”, “ELISA”
National Institutes of Health	1	("COVID" OR "SARS-CoV-2")
	2	("sero*" OR "antibod*" OR "immun*" OR "RDT" OR "ELISA" OR "LFIA")
	3	allintext:(1 AND 2) site:nih.gov -site:ncbi.nlm.nih.gov
	3	2 AND 3
United States Centres for Disease Control and Prevention	1	("COVID" OR "SARS-CoV-2")
	2	("sero*" OR "antibod*" OR "immun*" OR "RDT" OR "ELISA" OR "LFIA")
	3	allintext:(1 AND 2) site:cdc.gov
	5	2 AND 3
European Centres for Disease Control and Prevention	1	("COVID" OR "SARS-CoV-2")
	2	("sero*" OR "antibod*" OR "immun*" OR "RDT" OR "ELISA" OR "LFIA")
	3	allintext:(1 AND 2) site:ecdc.europa.eu
	5	2 AND 3

Sources: Google News
Dates: January 1, 2020 to December 31, 2020

Source	Search strategy	
Google news	1	(antibody OR antibodies OR surveillance OR screen OR serology OR serological OR serosurvey OR ELISA OR LFIA OR assay OR blood OR serum OR immune OR immunity OR herd immunity OR random test)

For peer review only

S3 File. Tool for assessing study risk of bias

**Item 1: Was the sample frame appropriate to address the target population?**	
Yes	Sample frame described and it approximated the target population
No	Sample frame did not approximate the target population (e.g., blood donors do not represent general population, doctors do not represent all health care providers)
Exclude	Sample frame not described
*Notes	The term "target population" should not be taken to infer every individual from everywhere or with similar disease or exposure characteristics. Instead, give consideration to specific population characteristics in the study, including age range, gender, morbidities, medications, and other potentially influential factors. For example, a sample frame may not be appropriate to address the target population if a certain group has been used (such as those working for one organisation, or one profession) and the results then inferred to the target population (i.e. working adults). A sample frame may be appropriate when it includes almost all the members of the target population (i.e. a census, or a complete list of participants or complete registry data).

**Item 2: Were study participants recruited in an appropriate way?**	
Yes	Probability sampling method (simple or stratified random) or entire sample (e.g., an entire town) was used
No	Non-probability sampling
Exclude	Sampling method not reported

**Item 3: Was the sample size adequate?**	
Yes	$\geq$599
No	<599
Exclude	Sample size not reported
*Notes	To calculate the required sample size we used an assumed prevalence of 2.5%, which was the global average estimated by the WHO in April, 2020.[1] Based on guidance by the Joanna Briggs Institute and published medical statistical recommendations we selected a precision value that was half the assumed prevalence (1.25%) [2,3]. We calculated a minimum sample size of 599 using these inputs:<br>Sample size calculation: $n = \frac{Z^2 P(1-P)}{d^2}$<br>Where n = sample size;<br>Z = Z statistic for level of confidence (95%);<br>P = expected prevalence (2.5% WHO global estimate);<br>d = precision (1.25%)<br><br>In cases where the sample size calculation was provided and the required sample for 80% power was below our threshold (n<599), this item was marked as yes.

**Item 4: Were the study subjects and setting described in detail?**	
Yes	Average age and distribution of gender/sex provided
No	Neither age or gender/sex is provided, or only one of age and gender/sex is provided

For peer review only

Item 5: Was data analysis conducted with sufficient coverage of the identified sample?	
Yes	The demographic characteristics (gender/sex, age, and ethnicity) of the sample is at least somewhat representative of the population
No	The demographic characteristics (gender/sex, age, and ethnicity) of the sample is not representative of the population
Unclear	Information is not provided about demographic characteristics of the sample (gender/sex, age, and ethnicity)

Item 6: Were valid methods used for the identification of the condition?	
Yes	The test used met the FDA standards for Emergency Use Authorizations for COVID-19 serological tests: sensitivity minimum 90%, specificity minimum 95%, as reported in the study [4].
No	The test used did not meet the FDA standards for Emergency Use Authorizations for COVID-19 serological tests: sensitivity minimum 90%, specificity minimum 95%.
Exclude	Test sensitivity and specificity not reported

Item 7: Was the condition measured in a standard, reliable way for all participants?	
Yes	The same serology test was used for all participants
No	Different serology tests were used for participants
Unclear	No details were provided about which participants received which serology tests

Item 8: Was there appropriate statistical analysis?	
Yes	Does all of the following: corrects for population characteristics or the sample is somewhat representative of the population (probability sampling), corrects for test characteristics), and provides the information necessary to determine the numerator, denominator, prevalence estimate, and confidence interval.
No	Does not correct for population characteristics and the sample is not likely representative of the population (non-probability sampling), does not correct for test or provide the information necessary to correct for test characteristics, or does not provide the information necessary to determine the numerator, denominator, prevalence estimate, and confidence interval.

Item 9: Was the response rate adequate, and if not, was the low response rate managed appropriately?	
Yes	Response rate > 60% or the demographics of the sample were a reasonable match to those of the target population [5]
No	Response rate < 60% and the demographics of the sample were not a reasonable match to those of the target population
Unclear	Response rate not provided and it was unclear if the demographics of the sample differed from the target population

Item 10: Overall risk of bias	
Low	The estimates are very likely correct for the target population. To obtain a low risk of bias classification, all criteria must be met or departures from the criteria must be minimal and unlikely to impact on the validity and reliability of the prevalence estimate. These include sample sizes that are just below the threshold when all other criteria are met,

For peer review only

	reporting only some of characteristics of the sample, test characteristics below the threshold but corrections for the test performance, and response rates that are just below the threshold in the context of probability based sampling of an appropriate sampling frame with population weighted seroprevalence estimates.
Moderate	The estimates are likely correct for the target population. To obtain a moderate risk of bias classification, most criteria must be met and departures from the criteria are likely to have only a small impact on the validity and reliability of the prevalence estimates.
High	The estimates are not likely correct for the target population. To obtain a high risk of bias, many criteria must not be met or departures from criteria are likely to have a major impact on the validity and reliability of the prevalence estimates.
Unclear	There was insufficient information to assess the risk of bias.

For peer review only

For peer review only - http://bmjopen.bmj.com/site/about/guidelines.xhtml

S5 File. Details of occupational coding

For each seroprevalence estimate, we identified the relevant Standard Occupational Classification (SOC) 2010 codes. This was done by applying the National Institute for Occupational Safety & Health (NIOSH) Industry and Occupation Computerized Coding System (NIOCCS) to text occupation descriptions extracted by members of the research team. There is no standard cut-off for manually verifying results from the National Institute for Occupational Safety & Health (NIOSH) Industry and Occupation Computerized Coding System (NIOCCS). However, NIOCCS reports the probability of correct classification to the six-digit level. After manually verifying a subset of records from the first round of classification, we decided to manual perform a second round of classification for any observations for which the probability of correct classification was <0.8. This cut-off was chosen based on the observation that that most codes with a probability of correct classification to of ≥0.8 to the six-digit level were correctly coded at the two- and three-digit level, which we used in our main analyses and are more likely to be coded correctly than the more granular, 6-digit codes and consideration of the number of records that could feasibly be verified manually

For peer review only

References for supplementary files

1. Boseley S. WHO warns that few have developed antibodies to Covid-19. The Guardian [Internet]. 2020 Apr 20; Available from: https://www.theguardian.com/society/2020/apr/20/studies-suggest-very-few-have-had-covid-19-without-symptoms
2. Munn Z, Moola S, Lisy K, Riitano D, Tufanaru C. Methodological guidance for systematic reviews of observational epidemiological studies reporting prevalence and cumulative incidence data. Int J Evid Based Healthc. 2015 Sep;13(3):147–53.
3. Naing L, Winn T, Ruslil B. Practical issues in calculating the sample size for prevalence studies. Arch Orofac Sci. 2006;1:9–14.
4. U.S. Food & Drug Administration. Emergency Use Authorization for SARS-CoV-2 Antibody Tests [Internet]. 2020 [cited 2020 May 5]. Available from: https://www.fda.gov/media/137470/download.
5. Morton MBS, Bandara DK, Robinson EM, Carr PEA. In the 21st century, what is an acceptable response rate? Aust N Z J Public Health. 2012 April; 36 (2): 106-8.

6 Bobrovitz N, Arora RK, Cao C, Boucher E, Liu M, Donnici C, Yanes-Lane M, Whelan M, Perlman-Arrow S, Chen J, Rahim H. Global seroprevalence of SARS-CoV-2 antibodies: A systematic review and meta-analysis. PloS one. 2021 Jun 23;16(6):e0252617.

For peer review only

33

For peer review only - http://bmjopen.bmj.com/site/about/guidelines.xhtml

Supplementary File I. List of all estimates, included studies and references

SOC 2010 Major Group	Study	N	SOC 2010 Occupation Title	Study Type	Study Dates	Country	Serum positive prevalence (95% CIs)	Overall Risk of Bias (JBI)
Not employed (mixed)*	Merkely et al., 2020[1]	n=209	Homemaker (Unpaid)	Cross-sectional survey	05/01 - 05/16	Hungary	0.73% (0-1.74%)	Moderate
Not employed (mixed)*	Siddiqui et al., 2020[2]	n=37	Homemaker (Unpaid)	Prospective cohort	04/15 - 08/15	India	18.9%	High
Not employed (mixed)*	Biggs et al., 2020[3]	n=157	Retired (Unpaid)	Cross-sectional survey	04/28 - 05/03	United States of America	1.91%	Moderate
Not employed (mixed)*	Carrat et al., 2020[4]	n=5381	Retired (Unpaid)	Prospective cohort	05/04 - 06/23	France	4.3% (3.5-5%)	Moderate
Not employed (mixed)*	Merkely et al., 2020[1]	n=2767	Retired (Unpaid)	Cross-sectional survey	05/01 - 05/16	Hungary	1.09% (0.66-1.52%)	Moderate
Not employed (mixed)*	Richard et al., 2020[5]	n=1635	Retired (Unpaid)	Cross-sectional survey	04/06 - 06/30	Switzerland	4.3%	Low
Not employed (mixed)*	Siddiqui et al., 2020[2]	n=10	Retired (Unpaid)	Prospective cohort	04/15 - 08/15	India	20%	High
Not employed (mixed)*	Alemu et al., 2020[6]	n=32	Student (Unpaid)	Cross-sectional survey	04/23 - 04/28	Ethiopia	15.6%	Moderate
Not employed (mixed)*	Biggs et al., 2020[3]	n=16	Student (Unpaid)	Cross-sectional survey	04/28 - 05/03	United States of America	12.5%	Moderate
Not employed (mixed)*	Brehm et al., 2020[7]	n=73	Student (Unpaid)	Cross sectional study with prospective cohort follow up of a subset of the sample	03/20 - 07/17	Germany	2.7%	Moderate
Not employed (mixed)*	Carrat et al., 2020[4]	n=81	Student (Unpaid)	Prospective cohort	05/04 - 06/23	France	7.2% (0.1-12.6%)	Moderate

For peer review only

Not employed (mixed)*	Iversen et al., 2020[8]	n=688	Student (Unpaid)	Cross-sectional survey	04/15 - 04/22	Denmark	14.97%	Low
Not employed (mixed)*	Lumley et al., 2020[9]	n=620	Student (Unpaid)	Prospective cohort	04/23 - 11/30	The United Kingdom	6.77%	Moderate
Not employed (mixed)*	Merkely et al., 2020[1]	n=774	Student (Unpaid)	Cross-sectional survey	05/01 - 05/16	Hungary	0.69% (0-1.49%)	Moderate
Not employed (mixed)*	Richard et al., 2020[5]	n=666	Student (Unpaid)	Cross-sectional survey	04/06 - 06/30	Switzerland	10.5%	Low
Not employed (mixed)*	Shakiba et al., 2020[10]	n=114	Student (Unpaid)	Cross-sectional survey	04/11 - 04/19	Iran (Islamic Republic of)	17.5% (11.3-23.7%)	Moderate
Not employed (mixed)*	Siddiqui et al., 2020[2]	n=14	Student (Unpaid)	Prospective cohort	04/15 - 08/15	India	21.4%	High
Not employed (mixed)*	Tilley et al., 2020[11]	n=790	Student (Unpaid)	Cross-sectional survey	04/29 - 05/08	United States of America	4% (3-5.1%)	Moderate
Not employed (mixed)*	Tsitsilonis et al., 2020[12]	n=1395	Student (Unpaid)	Cross-sectional survey	06/15 - 07/15	Greece	0.42% (0.03-1.5%)	Moderate
Not employed (mixed)*	Arnaldo et al., 2020[13]	n=513	Military, Rank Not Specified	Cross-sectional survey	07/06 - 07/13	Mozambique	3.7%	High
Not employed (mixed)*	Arnaldo et al., 2020[14]	n=116	Military, Rank Not Specified	Cross-sectional survey	11/02 - 11/12	Mozambique	1.7%	High
Not employed (mixed)*	Mabunda et al., 2020[15]	n=324	Military, Rank Not Specified	Cross-sectional survey	09/21 - 10/02	Mozambique	2.8%	High
Not employed (mixed)*	Mahomed et al., 2020[16]	n=116	Military, Rank Not Specified	Cross-sectional survey	11/26 - 12/03	Mozambique	18.1%	High
Not employed (mixed)*	Payne et al., 2020[17]	n=382	Military, Rank Not Specified	Cross-sectional survey	04/20 - 04/24	United States of America	59.7%	High
Not employed (mixed)*	World et al., 2020[18]	n=6900	Military, Rank Not Specified	Cross-sectional survey	08/15 - 10/15	Republic of Korea	0.36%	Unclear
Management Occupations (11-0000)	Shakiba et al., 2020[10]	n=16	Farmers, Ranchers, and Other Agricultural Managers	Cross-sectional survey	04/11 - 04/19	Iran (Islamic Republic of)	19.7% (9.1-31%)	Moderate
Management Occupations (11-	Favara et al., 2020[19]	n=43	Medical and Health Services Managers	Cross-sectional survey	07/13 - 07/13	The United Kingdom	9.3%	High

0000)								
Management Occupations (11-0000)	Galan et al., 2020[20]	n=170	Medical and Health Services Managers	Cross-sectional survey	04/14 - 04/27	Spain	27.6%	High
Management Occupations (11-0000)	Hunter et al., 2020[21]	n=44	Medical and Health Services Managers	Cross-sectional survey	04/29 - 05/08	United States of America	4.55%	High
Management Occupations (11-0000)	Leidner et al., 2020[22]	n=257	Medical and Health Services Managers	Cross sectional study with prospective cohort follow up of a subset of the sample	04/08 - 05/22	United States of America	3.11%	High
Management Occupations (11-0000)	Martin et al., 2020[23]	n=2078	Medical and Health Services Managers	Cross-sectional survey	05/29 - 07/13	The United Kingdom	6.79%	Moderate
Management Occupations (11-0000)	Siddiqui et al., 2020[2]	n=15	Medical and Health Services Managers	Prospective cohort	04/15 - 08/15	India	20%	High
Management Occupations (11-0000)	Baracco et al., 2020[24]	n=45	Managers, All Other	Cross-sectional survey	04/23 - 05/05	Italy	6.67%	High
Management Occupations (11-0000)	Goenka et al., 2020[25]	n=71	Managers, All Other	Cross-sectional survey	07/12 - 08/23	India	7.04%	Moderate
Management Occupations (11-0000)	Goenka et al., 2020[26]	n=13	Managers, All Other	Cross-sectional survey	08/01 - 08/31	India	38.46%	High
Business and Financial Operations Occupations (13-0000)	Satpati et al., 2020[27]	n=43	Management Analysts	Cross-sectional survey	07/26 - 08/08	India	2.33%	Moderate
Business and Financial	Poustchi et al., 2020[28]	n=880	Financial Specialists	Cross-sectional survey	04/17 - 06/02	Iran (Islamic Republic of)	14.2% (12.1-16.5%)	Moderate

For peer review only

Operations Occupations (13-0000)								
Computer and Mathematical Occupations (15-0000)	Biggs et al., 2020[3]	n=47	Computer User Support Specialists	Cross-sectional survey	04/28 - 05/03	United States of America	0%	Moderate
Architecture and Engineering Occupations (17-0000)	Siddiqui et al., 2020[2]	n=21	Engineers	Prospective cohort	04/15 - 08/15	India	42.9%	High
Life, Physical, and Social Science Occupations (19-0000)	Jones et al., 2020[29]	n=245	Medical Scientists	Cross-sectional survey	01/15 - 06/15	The United Kingdom	1.9%	High
Life, Physical, and Social Science Occupations (19-0000)	Anna et al., 2020[30]	n=505	Medical Scientists, Except Epidemiologists	Prospective cohort	04/28 - 07/31	France	8.71%	Moderate
Life, Physical, and Social Science Occupations (19-0000)	Erber et al., 2020[31]	n=635	Medical Scientists, Except Epidemiologists	Cross-sectional survey	04/14 - 05/29	Germany	1.24%	High
Life, Physical, and Social Science Occupations (19-0000)	Favara et al., 2020[19]	n=38	Medical Scientists, Except Epidemiologists	Cross-sectional survey	07/13 - 07/13	The United Kingdom	2.6%	High
Life, Physical, and Social Science Occupations (19-0000)	Hanrath et al., 2020[32]	n=468	Medical Scientists, Except Epidemiologists	Cross-sectional survey	05/29 - 07/06	The United Kingdom	6.2%	High
Life, Physical, and Social Science Occupations (19-0000)	Leidner et al., 2020[22]	n=2654	Medical Scientists, Except Epidemiologists	Cross sectional study with prospective cohort follow up of a subset of the sample	04/08 - 05/22	United States of America	2.22%	High

For peer review only

Life, Physical, and Social Science Occupations (19-0000)	Martin et al., 2020[23]	n=1154	Medical Scientists, Except Epidemiologists	Cross-sectional survey	05/29 - 07/13	The United Kingdom	9.71%	Moderate
Life, Physical, and Social Science Occupations (19-0000)	Rosser et al., 2020[33]	n=102	Medical Scientists, Except Epidemiologists	Cross-sectional survey	04/20 - 05/20	United States of America	0.98%	High
Life, Physical, and Social Science Occupations (19-0000)	Silva et al., 2020[34]	n=69	Chemists	Cross-sectional survey	06/05 - 07/31	Brazil	4%	High
Life, Physical, and Social Science Occupations (19-0000)	Tsitsilonis et al., 2020[12]	n=250	Physical Scientists, All Other	Cross-sectional survey	06/15 - 07/15	Greece	1.42% (0-7.24%)	Moderate
Community and Social Service Occupations (21-0000)	Jones et al., 2020[29]	n=211	Healthcare Social Workers	Cross-sectional survey	01/15 - 06/15	The United Kingdom	6.3%	High
Community and Social Service Occupations (21-0000)	Leidner et al., 2020[22]	n=235	Social Workers, All Other	Cross sectional study with prospective cohort follow up of a subset of the sample	04/08 - 05/22	United States of America	3.4%	High
Community and Social Service Occupations (21-0000)	Rosser et al., 2020[33]	n=117	Social Workers, All Other	Cross-sectional survey	04/20 - 05/20	United States of America	1.71%	High
Community and Social Service Occupations (21-0000)	Sabourin et al., 2020[35]	n=91	Social Workers, All Other	Cross-sectional survey	07/15 - 08/15	United States of America	5.49%	High
Community and Social Service	Yogo et al., 2020[36]	n=35	Social Workers, All Other	Cross-sectional survey	05/20 - 06/08	United States of America	0%	High

For peer review only

Occupations (21-0000)								
Community and Social Service Occupations (21-0000)	Biggs et al., 2020[3]	n=6	Religious Workers	Cross-sectional survey	04/28 - 05/03	United States of America	16.67%	Moderate
Education, Training, and Library Occupations (25-0000)	Campos et al., 2020[37]	n=2715	Postsecondary Teachers	Cross-sectional survey	05/13 - 07/10	Portugal	2.6%	High
Education, Training, and Library Occupations (25-0000)	Goncalves et al., 2020[38]	n=1636	Postsecondary Teachers	Cross-sectional survey	06/15 - 06/30	Portugal	3.05%	Moderate
Education, Training, and Library Occupations (25-0000)	Tsitsilonis et al., 2020[12]	n=312	Postsecondary Teachers	Cross-sectional survey	06/15 - 07/15	Greece	1.2% (0.14-3.7%)	Moderate
Education, Training, and Library Occupations (25-0000)	Fontanet et al., 2020[39]	n=42	Elementary and Middle School Teachers	Retrospective cohort	04/28 - 04/30	France	7.1%	Moderate
Education, Training, and Library Occupations (25-0000)	Siddiqui et al., 2020[2]	n=8	Elementary and Middle School Teachers	Prospective cohort	04/15 - 08/15	India	25%	High
Education, Training, and Library Occupations (25-0000)	Torres et al., 2020[40]	n=165	Elementary and Middle School Teachers	Cross-sectional survey	05/04 - 05/19	Chile	20.6% (14.7-27.6%)	High

For peer review only

Arts, Design, Entertainment, Sports, and Media Occupations (27-0000)	Halatoko et al., 2020[41]	n=55	Fine Artists, Including Painters, Sculptors, and Illustrators	Cross-sectional survey	04/23 - 05/08	Togo	0%	High
Arts, Design, Entertainment, Sports, and Media Occupations (27-0000)	Slusser et al., 2020[42]	n=5603	Athletes, Coaches, Umpires, and Related Workers	Cross-sectional survey	04/08 - 04/21	United States of America	0.7% (0.28-1.15%)	Unclear
Arts, Design, Entertainment, Sports, and Media Occupations (27-0000)	Vince et al., 2020[43]	n=272	Athletes, Coaches, Umpires, and Related Workers	Prospective cohort	05/29 - 07/31	Croatia	14%	Moderate
Arts, Design, Entertainment, Sports, and Media Occupations (27-0000)	Vince et al., 2020[43]	n=43	Coaches and Scouts	Prospective cohort	05/29 - 07/31	Croatia	16.3%	Moderate
Arts, Design, Entertainment, Sports, and Media Occupations (27-0000)	Mack et al., 2020[44]	n=1007	Umpires, Referees, and Other Sports Officials	Prospective cohort	06/16 - 06/30	Germany	2.09% (1.37-3.17%)	High
Arts, Design, Entertainment, Sports, and Media Occupations (27-0000)	Khan et al., 2020[45]	n=44	Media and Communication Workers	Cross-sectional survey	07/01 - 07/15	India	0%	Moderate
Healthcare Practitioners and Technical Occupations (29-0000)	Akinbami et al., 2020[46]	n=566	Healthcare Practitioners and Technical Occupations	Cross-sectional survey	05/18 - 06/13	United States of America	4.6% (3-6.7%)	Moderate

For peer review only

Healthcare Practitioners and Technical Occupations (29-0000)	Khan et al., 2020[45]	n=355	Healthcare Practitioners and Technical Occupations	Cross-sectional survey	07/01 - 07/15	India	4.8% (3-7.6%)	Moderate
Healthcare Practitioners and Technical Occupations (29-0000)	Leidner et al., 2020[22]	n=402	Healthcare Practitioners and Technical Occupations	Cross sectional study with prospective cohort follow up of a subset of the sample	04/08 - 05/22	United States of America	1.49%	High
Healthcare Occupations (mixed)*	Hanrath et al., 2020[32]	n=102	Healthcare Practitioners and Technical Occupations and Healthcare Support Occupations*	Cross-sectional survey	05/29 - 07/06	The United Kingdom	6.62%	High
Healthcare Occupations (mixed)*	Jones et al., 2020[29]	n=413	Healthcare Practitioners and Technical Occupations and Healthcare Support Occupations*	Cross-sectional survey	01/15 - 06/15	The United Kingdom	7.8%	High
Healthcare Occupations (mixed)*	Martin et al., 2020[23]	n=550	Healthcare Practitioners and Technical Occupations and Healthcare Support Occupations*	Cross-sectional survey	05/29 - 07/13	The United Kingdom	10.36%	Moderate
Healthcare Occupations (mixed)*	Amendola et al., 2020[47]	n=117	Healthcare Practitioners and Technical Occupations and Healthcare Support Occupations*	Cross-sectional survey	04/15 - 04/15	Italy	4.27%	High
Healthcare Occupations (mixed)*	Arnaldo et al., 2020[48]	n=543	Healthcare Practitioners and Technical Occupations and Healthcare Support Occupations*	Cross-sectional survey	08/10 - 08/21	Mozambique	3.7%	High

For peer review only

Healthcare Occupations (mixed)*	Bal et al., 2020[49]	n=190	Healthcare Practitioners and Technical Occupations and Healthcare Support Occupations*	Cross-sectional survey	04/10 - 05/28	France	3.68%	High
Healthcare Occupations (mixed)*	Barallat et al., 2020[50]	n=429	Healthcare Practitioners and Technical Occupations and Healthcare Support Occupations*	Cross-sectional survey	05/04 - 05/22	Spain	7.69%	High
Healthcare Occupations (mixed)*	Bardai et al., 2020[51]	n=35	Healthcare Practitioners and Technical Occupations and Healthcare Support Occupations*	Cross-sectional survey	06/10 - 07/27	Canada	11%	High
Healthcare Occupations (mixed)*	Bardai et al., 2020[51]	n=20	Healthcare Practitioners and Technical Occupations and Healthcare Support Occupations*	Cross-sectional survey	06/10 - 07/27	Canada	15%	High
Healthcare Occupations (mixed)*	Bardai et al., 2020[51]	n=44	Healthcare Practitioners and Technical Occupations and Healthcare Support Occupations*	Cross-sectional survey	06/10 - 07/27	Canada	11%	High
Healthcare Occupations (mixed)*	Bardai et al., 2020[51]	n=99	Healthcare Practitioners and Technical Occupations and Healthcare Support Occupations*	Cross-sectional survey	06/10 - 07/27	Canada	12%	High
Healthcare Occupations (mixed)*	Biggs et al., 2020[3]	n=59	Healthcare Practitioners and Technical Occupations and Healthcare Support Occupations*	Cross-sectional survey	04/28 - 05/03	United States of America	10.17%	Moderate

For peer review only

Healthcare Occupations (mixed)*	Blairon et al., 2020[52]	n=588	Healthcare Practitioners and Technical Occupations and Healthcare Support Occupations*	Cross-sectional survey	05/25 - 06/19	Belgium	19.2%	High
Healthcare Occupations (mixed)*	Borraz et al., 2020[53]	n=289	Healthcare Practitioners and Technical Occupations and Healthcare Support Occupations*	Prospective cohort	03/20 - 04/21	Spain	5.88%	High
Healthcare Occupations (mixed)*	Brunner et al., 2020[54]	n=762	Healthcare Practitioners and Technical Occupations and Healthcare Support Occupations*	Cross-sectional survey	05/04 - 05/29	United States of America	4.5%	High
Healthcare Occupations (mixed)*	Brunner et al., 2020[54]	n=764	Healthcare Practitioners and Technical Occupations and Healthcare Support Occupations*	Cross-sectional survey	05/04 - 05/29	United States of America	2%	High
Healthcare Occupations (mixed)*	Carozzi et al., 2020[55]	n=17098	Healthcare Practitioners and Technical Occupations and Healthcare Support Occupations*	Cross-sectional survey	04/01 - 04/30	Italy	3.1%	High
Healthcare Occupations (mixed)*	Carrat et al., 2020[4]	n=568	Healthcare Practitioners and Technical Occupations and Healthcare Support Occupations*	Prospective cohort	05/04 - 06/23	France	11.6% (8.3-14.4%)	Moderate
Healthcare Occupations (mixed)*	Cavlek et al., 2020[56]	n=558	Healthcare Practitioners and Technical Occupations and Healthcare Support Occupations*	Cross-sectional survey	04/25 - 05/24	Croatia	1.25%	High

For peer review only

Healthcare Occupations (mixed)*	Chibwana et al., 2020[57]	n=500	Healthcare Practitioners and Technical Occupations and Healthcare Support Occupations*	Prospective cohort	05/22 - 06/19	Malawi	12.3% (8.2-16.5%)	High
Healthcare Occupations (mixed)*	Coffman et al., 2020[58]	n=1100	Healthcare Practitioners and Technical Occupations and Healthcare Support Occupations*	Cross-sectional survey	07/01 - 07/31	United States of America	2.2%	Unclear
Healthcare Occupations (mixed)*	Cooper et al., 2020[59]	n=118	Healthcare Practitioners and Technical Occupations and Healthcare Support Occupations*	Cross-sectional survey	06/10 - 08/07	The United Kingdom	8.47%	Moderate
Healthcare Occupations (mixed)*	Cooper et al., 2020[59]	n=27	Healthcare Practitioners and Technical Occupations and Healthcare Support Occupations*	Cross-sectional survey	06/10 - 08/07	The United Kingdom	14.81%	Moderate
Healthcare Occupations (mixed)*	Cooper et al., 2020[59]	n=24	Healthcare Practitioners and Technical Occupations and Healthcare Support Occupations*	Cross-sectional survey	06/10 - 08/07	The United Kingdom	12.5%	Moderate
Healthcare Occupations (mixed)*	Cooper et al., 2020[59]	n=1068	Healthcare Practitioners and Technical Occupations and Healthcare Support Occupations*	Cross-sectional survey	06/10 - 08/07	The United Kingdom	5.43%	Moderate
Healthcare Occupations (mixed)*	Cooper et al., 2020[59]	n=174	Healthcare Practitioners and Technical Occupations and Healthcare Support Occupations*	Cross-sectional survey	06/10 - 08/07	The United Kingdom	5.75%	Moderate

For peer review only

Healthcare Occupations (mixed)*	Cooper et al., 2020[59]	n=319	Healthcare Practitioners and Technical Occupations and Healthcare Support Occupations*	Cross-sectional survey	06/10 - 08/07	The United Kingdom	11.29%	Moderate
Healthcare Occupations (mixed)*	Cooper et al., 2020[59]	n=5698	Healthcare Practitioners and Technical Occupations and Healthcare Support Occupations*	Cross-sectional survey	06/10 - 08/07	The United Kingdom	7.2%	Moderate
Healthcare Occupations (mixed)*	Cooper et al., 2020[59]	n=412	Healthcare Practitioners and Technical Occupations and Healthcare Support Occupations*	Cross-sectional survey	06/10 - 08/07	The United Kingdom	4.61%	Moderate
Healthcare Occupations (mixed)*	Denyer et al., 2020[60]	n=5850	Healthcare Practitioners and Technical Occupations and Healthcare Support Occupations*	Cross-sectional survey	05/12 - 05/18	Japan	1.79%	Unclear
Healthcare Occupations (mixed)*	Dimeglio et al., 2020[61]	n=8758	Healthcare Practitioners and Technical Occupations and Healthcare Support Occupations*	Cross-sectional survey	06/10 - 07/10	France	3.2% (2.8-3.5%)	High
Healthcare Occupations (mixed)*	Erber et al., 2020[31]	n=603	Healthcare Practitioners and Technical Occupations and Healthcare Support Occupations*	Cross-sectional survey	04/14 - 05/29	Germany	2.8%	High
Healthcare Occupations (mixed)*	Fuereder et al., 2020[62]	n=62	Healthcare Practitioners and Technical Occupations and Healthcare Support Occupations*	Retrospective cohort	04/01 - 06/04	Austria	3.2% (0.4-11.2%)	High

For peer review only

Healthcare Occupations (mixed)*	Fusco et al., 2020[63]	n=115	Healthcare Practitioners and Technical Occupations and Healthcare Support Occupations*	Cross-sectional survey	03/23 - 04/02	Italy	1.74%	High
Healthcare Occupations (mixed)*	Geraci et al., 2020[64]	n=230	Healthcare Practitioners and Technical Occupations and Healthcare Support Occupations*	Cross-sectional survey	03/16 - 05/20	United States of America	2.17%	High
Healthcare Occupations (mixed)*	Gudo et al., 2020[65]	n=1427	Healthcare Practitioners and Technical Occupations and Healthcare Support Occupations*	Cross-sectional survey	06/17 - 06/30	Mozambique	7% (6-9%)	High
Healthcare Occupations (mixed)*	Hackner et al., 2020[66]	n=130	Healthcare Practitioners and Technical Occupations and Healthcare Support Occupations*	Cross-sectional survey	04/01 - 04/30	Austria	2.3%	High
Healthcare Occupations (mixed)*	Halatoko et al., 2020[41]	n=370	Healthcare Practitioners and Technical Occupations and Healthcare Support Occupations*	Cross-sectional survey	04/23 - 05/08	Togo	1.4%	High
Healthcare Occupations (mixed)*	Haq et al., 2020[67]	n=76	Healthcare Practitioners and Technical Occupations and Healthcare Support Occupations*	Cross-sectional survey	06/15 - 06/29	Pakistan	35.5% (24.8-47.3%)	Moderate
Healthcare Occupations (mixed)*	He et al., 2020[68]	n=1059	Healthcare Practitioners and Technical Occupations and Healthcare Support Occupations*	Repeated cross sectional study	05/13 - 06/10	China	9.3%	High

For peer review only

Healthcare Occupations (mixed)*	Herzberg et al., 2020[69]	n=871	Healthcare Practitioners and Technical Occupations and Healthcare Support Occupations*	Prospective cohort	04/14 - 06/16	Germany	2.64%	High
Healthcare Occupations (mixed)*	Jeremias et al., 2020[70]	n=100	Healthcare Practitioners and Technical Occupations and Healthcare Support Occupations*	Cross-sectional survey	03/01 - 04/30	United States of America	12%	High
Healthcare Occupations (mixed)*	Jespersen et al., 2020[71]	n=17948	Healthcare Practitioners and Technical Occupations and Healthcare Support Occupations*	Cross-sectional survey	05/18 - 06/19	Denmark	3.36% (2.38-3.82%)	Moderate
Healthcare Occupations (mixed)*	Kassem et al., 2020[72]	n=74	Healthcare Practitioners and Technical Occupations and Healthcare Support Occupations*	Cross-sectional survey	06/01 - 06/14	Egypt	12.2%	High
Healthcare Occupations (mixed)*	Kern et al., 2020[73]	n=1316	Healthcare Practitioners and Technical Occupations and Healthcare Support Occupations*	Cross-sectional survey	04/09 - 04/16	Germany	1.06% (0.58-1.78%)	High
Healthcare Occupations (mixed)*	Khalil et al., 2020[74]	n=190	Healthcare Practitioners and Technical Occupations and Healthcare Support Occupations*	Cross-sectional survey	05/15 - 05/28	The United Kingdom	22%	High
Healthcare Occupations (mixed)*	Kumar et al., 2020[75]	n=635	Healthcare Practitioners and Technical Occupations and Healthcare Support Occupations*	Repeated cross sectional study	07/11 - 07/24	India	0%	High

For peer review only

Healthcare Occupations (mixed)*	Lackermair et al., 2020[76]	n=151	Healthcare Practitioners and Technical Occupations and Healthcare Support Occupations*	Cross-sectional survey	04/02 - 04/06	Germany	2.6% (0.8-7.1%)	High
Healthcare Occupations (mixed)*	Lahner et al., 2020[77]	n=1084	Healthcare Practitioners and Technical Occupations and Healthcare Support Occupations*	Cross-sectional survey	04/07 - 04/27	Italy	0.7%	High
Healthcare Occupations (mixed)*	Liu et al., 2020[78]	n=116	Healthcare Practitioners and Technical Occupations and Healthcare Support Occupations*	Cross-sectional survey	02/07 - 04/21	China	0%	High
Healthcare Occupations (mixed)*	Liu et al., 2020[78]	n=304	Healthcare Practitioners and Technical Occupations and Healthcare Support Occupations*	Cross-sectional survey	02/07 - 04/21	China	0%	High
Healthcare Occupations (mixed)*	Liu et al., 2020[79]	n=3832	Healthcare Practitioners and Technical Occupations and Healthcare Support Occupations*	Cross-sectional survey	02/29 - 04/29	China	4% (3.4-4.7%)	Moderate
Healthcare Occupations (mixed)*	Lorenzo et al., 2020[80]	n=38	Healthcare Practitioners and Technical Occupations and Healthcare Support Occupations*	Cross-sectional survey	05/02 - 05/31	Italy	5.3%	High
Healthcare Occupations (mixed)*	Mahomed et al., 2020[81]	n=569	Healthcare Practitioners and Technical Occupations and Healthcare Support Occupations*	Cross-sectional survey	08/31 - 10/12	Mozambique	0.7%	High

For peer review only

Healthcare Occupations (mixed)*	Mahumane et al., 2020[82]	n=380	Healthcare Practitioners and Technical Occupations and Healthcare Support Occupations*	Cross-sectional survey	11/02 - 11/17	Mozambique	1.3%	High
Healthcare Occupations (mixed)*	Majdoubi et al., 2020[83]	n=276	Healthcare Practitioners and Technical Occupations and Healthcare Support Occupations*	Cross-sectional survey	05/17 - 06/19	Canada	0.6% (0-2.71%)	High
Healthcare Occupations (mixed)*	Majiya et al., 2020[84]	n=185	Healthcare Practitioners and Technical Occupations and Healthcare Support Occupations*	Cross-sectional survey	06/26 - 06/30	Nigeria	25.41%	Moderate
Healthcare Occupations (mixed)*	Majiya et al., 2020[84]	n=43	Healthcare Practitioners and Technical Occupations and Healthcare Support Occupations*	Cross-sectional survey	06/26 - 06/30	Nigeria	37.21%	Moderate
Healthcare Occupations (mixed)*	Malfertheiner et al., 2020[85]	n=139	Healthcare Practitioners and Technical Occupations and Healthcare Support Occupations*	Prospective cohort	03/15 - 06/07	Germany	0%	High
Healthcare Occupations (mixed)*	Martin et al., 2020[86]	n=326	Healthcare Practitioners and Technical Occupations and Healthcare Support Occupations*	Cross-sectional survey	04/15 - 05/18	Belgium	11%	High
Healthcare Occupations (mixed)*	Martin et al., 2020[23]	n=4631	Healthcare Practitioners and Technical Occupations and Healthcare Support Occupations*	Cross-sectional survey	05/29 - 07/13	The United Kingdom	13.65%	Moderate

For peer review only

Healthcare Occupations (mixed)*	Melo et al., 2020[87]	n=471	Healthcare Practitioners and Technical Occupations and Healthcare Support Occupations*	Cross-sectional survey	06/01 - 06/30	Brazil	13.59%	High
Healthcare Occupations (mixed)*	Morcuende et al., 2020[88]	n=6	Healthcare Practitioners and Technical Occupations and Healthcare Support Occupations*	Cross-sectional survey	03/01 - 04/21	United States of America	0%	High
Healthcare Occupations (mixed)*	Moscola et al., 2020[89]	n=8156	Healthcare Practitioners and Technical Occupations and Healthcare Support Occupations*	Cross-sectional survey	04/20 - 06/23	United States of America	11.6%	High
Healthcare Occupations (mixed)*	Nishida et al., 2020[90]	n=49	Healthcare Practitioners and Technical Occupations and Healthcare Support Occupations*	Cross-sectional survey	06/12 - 06/19	Japan	0%	Moderate
Healthcare Occupations (mixed)*	Olalla et al., 2020[91]	n=498	Healthcare Practitioners and Technical Occupations and Healthcare Support Occupations*	Cross-sectional survey	04/15 - 04/25	Spain	2.2%	High
Healthcare Occupations (mixed)*	Pallett et al., 2020[92]	n=504	Healthcare Practitioners and Technical Occupations and Healthcare Support Occupations*	Prospective cohort	04/08 - 06/12	The United Kingdom	10.6% (7.6-13.6%)	High
Healthcare Occupations (mixed)*	Pere et al., 2020[93]	n=3569	Healthcare Practitioners and Technical Occupations and Healthcare Support Occupations*	Cross-sectional survey	05/02 - 06/26	France	11.9%	High

For peer review only

Healthcare Occupations (mixed)*	Poulikakos et al., 2020[94]	n=281	Healthcare Practitioners and Technical Occupations and Healthcare Support Occupations*	Cross-sectional survey	05/04 - 05/06	The United Kingdom	6%	High
Healthcare Occupations (mixed)*	Psichogiou et al., 2020[95]	n=1495	Healthcare Practitioners and Technical Occupations and Healthcare Support Occupations*	Cross-sectional survey	04/13 - 05/15	Greece	1.26% (0.43-3.26%)	Moderate
Healthcare Occupations (mixed)*	Satpati et al., 2020[27]	n=18	Healthcare Practitioners and Technical Occupations and Healthcare Support Occupations*	Cross-sectional survey	07/26 - 08/08	India	5.56%	Moderate
Healthcare Occupations (mixed)*	Seetharam et al., 2020[96]	n=728	Healthcare Practitioners and Technical Occupations and Healthcare Support Occupations*	Cross-sectional survey	08/16 - 08/29	India	27.3% (24.1-30.6%)	Unclear
Healthcare Occupations (mixed)*	Shakiba et al., 2020[10]	n=43	Healthcare Practitioners and Technical Occupations and Healthcare Support Occupations*	Cross-sectional survey	04/11 - 04/19	Iran (Islamic Republic of)	14.5% (4.5-25%)	Moderate
Healthcare Occupations (mixed)*	Shields et al., 2020[97]	n=516	Healthcare Practitioners and Technical Occupations and Healthcare Support Occupations*	Cross-sectional survey	04/24 - 04/25	The United Kingdom	24.4%	High
Healthcare Occupations (mixed)*	Silva et al., 2020[98]	n=61	Healthcare Practitioners and Technical Occupations and Healthcare Support Occupations*	Cross-sectional survey	04/09 - 04/29	Brazil	4.91%	High

For peer review only

Healthcare Occupations (mixed)*	Solodky et al., 2020[99]	n=85	Healthcare Practitioners and Technical Occupations and Healthcare Support Occupations*	Cross-sectional survey	03/01 - 04/16	France	5.88%	High
Healthcare Occupations (mixed)*	Soriano et al., 2020[100]	n=108	Healthcare Practitioners and Technical Occupations and Healthcare Support Occupations*	Retrospective cohort	04/26 - 05/16	Spain	13%	High
Healthcare Occupations (mixed)*	Statistica et al., 2020[101]	n=64660	Healthcare Practitioners and Technical Occupations and Healthcare Support Occupations*	Cross-sectional survey	05/25 - 07/15	Italy	2.5%	Unclear
Healthcare Occupations (mixed)*	Steensels et al., 2020[102]	n=3056	Healthcare Practitioners and Technical Occupations and Healthcare Support Occupations*	Cross-sectional survey	04/22 - 04/30	Belgium	6.4% (5.5-7.3%)	High
Healthcare Occupations (mixed)*	Stock et al., 2020[103]	n=98	Healthcare Practitioners and Technical Occupations and Healthcare Support Occupations*	Cross-sectional survey	04/04 - 04/20	United States of America	15.3%	High
Healthcare Occupations (mixed)*	Takita et al., 2020[104]	n=175	Healthcare Practitioners and Technical Occupations and Healthcare Support Occupations*	Cross-sectional survey	04/20 - 05/20	Japan	4% (1.62-8.07%)	High
Healthcare Occupations (mixed)*	Tong et al., 2020[105]	n=191	Healthcare Practitioners and Technical Occupations and Healthcare Support Occupations*	Cross-sectional survey	05/12 - 05/15	China	0%	High

For peer review only

Healthcare Occupations (mixed)*	Trieu et al., 2020[106]	n=607	Healthcare Practitioners and Technical Occupations and Healthcare Support Occupations*	Prospective cohort	03/06 - 04/09	Norway	5.27%	High
Healthcare Occupations (mixed)*	Tu et al., 2020[107]	n=325	Healthcare Practitioners and Technical Occupations and Healthcare Support Occupations*	Cross sectional study with prospective cohort follow up of a subset of the sample	03/19 - 03/20	China	43.08%	High
Healthcare Occupations (mixed)*	Valdivia et al., 2020[108]	n=1153	Healthcare Practitioners and Technical Occupations and Healthcare Support Occupations*	Cross-sectional survey	04/13 - 04/30	Spain	3.5%	High
Healthcare Occupations (mixed)*	Vasquez et al., 2020[109]	n=1147	Healthcare Practitioners and Technical Occupations and Healthcare Support Occupations*	Cross-sectional survey	05/19 - 06/06	Peru	58.3%	High
Healthcare Occupations (mixed)*	Viegas et al., 2020[110]	n=1443	Healthcare Practitioners and Technical Occupations and Healthcare Support Occupations*	Cross-sectional survey	08/03 - 08/21	Mozambique	2.63%	High
Healthcare Occupations (mixed)*	Vlachoyiannopoulosa et al., 2020[111]	n=321	Healthcare Practitioners and Technical Occupations and Healthcare Support Occupations*	Cross-sectional survey	04/25 - 05/10	Greece	2.18%	High
Healthcare Occupations (mixed)*	Volta et al., 2020[112]	n=76	Healthcare Practitioners and Technical Occupations and Healthcare Support Occupations*	Cross-sectional survey	04/27 - 04/27	Italy	11.8%	High

For peer review only

Healthcare Occupations (mixed)*	Ward et al., 2020[113]	n=5416	Healthcare Practitioners and Technical Occupations and Healthcare Support Occupations*	Cross-sectional survey	09/15 - 09/28	The United Kingdom	10.67%	Moderate
Healthcare Occupations (mixed)*	Ward et al., 2020[113]	n=1692	Healthcare Practitioners and Technical Occupations and Healthcare Support Occupations*	Cross-sectional survey	09/15 - 09/28	The United Kingdom	6.68%	Moderate
Healthcare Occupations (mixed)*	Xiong et al., 2020[114]	n=797	Healthcare Practitioners and Technical Occupations and Healthcare Support Occupations*	Cross-sectional survey	02/12 - 03/17	China	4.39%	Unclear
Healthcare Occupations (mixed)*	Zhang et al., 2020[115]	n=63	Healthcare Practitioners and Technical Occupations and Healthcare Support Occupations*	Cross-sectional survey	01/21 - 02/16	China	0%	High
Healthcare Occupations (mixed)*	Zhao et al., 2020[116]	n=1060	Healthcare Practitioners and Technical Occupations and Healthcare Support Occupations*	Cross-sectional survey	01/14 - 02/21	China	8.3%	High
First responders (mixed)*	Ahmad et al., 2020[117]	n=40	Healthcare Practitioners and Technical Occupations and Protective Service Occupations (i.e. first responders)*	Cross-sectional survey	04/21 - 05/22	United States of America	20%	High
First responders (mixed)*	Halbrook et al., 2020[118]	n=679	Healthcare Practitioners and Technical Occupations and Protective Service Occupations (i.e. first responders)*	Cross-sectional survey	05/19 - 08/31	United States of America	8.1%	Moderate

For peer review only

First responders (mixed)*	Iwuji et al., 2020[119]	n=683	Healthcare Practitioners and Technical Occupations and Protective Service Occupations (i.e. first responders)*	Cross-sectional survey	05/12 - 05/13	United States of America	0.7%	High
First responders (mixed)*	Magyar et al., 2020[120]	n=70	Healthcare Practitioners and Technical Occupations and Protective Service Occupations (i.e. first responders)*	Cross-sectional survey	05/01 - 05/14	United States of America	4.29%	High
First responders (mixed)*	Martinez et al., 2020[121]	n=79	Healthcare Practitioners and Technical Occupations and Protective Service Occupations (i.e. first responders)*	Cross-sectional survey	04/16 - 04/17	United States of America	5.06%	High
First responders (mixed)*	Staletovich et al., 2020[122]	n=359	Healthcare Practitioners and Technical Occupations and Protective Service Occupations (i.e. first responders)*	Cross-sectional survey	05/17 - 05/22	United States of America	0%	Unclear
Healthcare Practitioners and Technical Occupations (29-0000)	Hibino et al., 2020[123]	n=806	Health Diagnosing and Treating Practitioners	Cross-sectional survey	06/01 - 07/30	Japan	0.74% (0.27-1.61%)	Unclear
Healthcare Practitioners and Technical Occupations (29-0000)	Jones et al., 2020[29]	n=856	Dentists, General	Cross-sectional survey	01/15 - 06/15	The United Kingdom	7.9%	High
Life, Physical, and Social Science	Calcagno et al., 2020[124]	n=343	Life, Physical, and Social Science Occupations	Cross-sectional survey	04/17 - 05/20	Italy	6.71%	Moderate

For peer review only

Occupations (19-0000)								
Healthcare Practitioners and Technical Occupations (29-0000)	Goenka et al., 2020[25]	n=49	Dietitians and Nutritionists	Cross-sectional survey	07/12 - 08/23	India	18.37%	Moderate
Healthcare Practitioners and Technical Occupations (29-0000)	Goenka et al., 2020[26]	n=6	Dietitians and Nutritionists	Cross-sectional survey	08/01 - 08/31	India	0%	High
Healthcare Practitioners and Technical Occupations (29-0000)	Akinbami et al., 2020[46]	n=321	Pharmacists	Cross-sectional survey	05/18 - 06/13	United States of America	4.4% (2.4-7.2%)	Moderate
Healthcare Practitioners and Technical Occupations (29-0000)	Alharbi et al., 2020[125]	n=5	Pharmacists	Cross-sectional survey	04/18 - 06/17	Saudi Arabia	0%	High
Healthcare Practitioners and Technical Occupations (29-0000)	Calcagno et al., 2020[124]	n=29	Pharmacists	Cross-sectional survey	04/17 - 05/20	Italy	3.45%	Moderate
Healthcare Practitioners and Technical Occupations (29-0000)	Chau et al., 2020[126]	n=17	Pharmacists	Cross-sectional survey	08/23 - 08/30	Viet Nam	0%	High
Healthcare Practitioners and Technical Occupations (29-0000)	Hanrath et al., 2020[32]	n=189	Pharmacists	Cross-sectional survey	05/29 - 07/06	The United Kingdom	4.76%	High

For peer review only

Healthcare Practitioners and Technical Occupations (29-0000)	Khan et al., 2020[127]	n=109	Pharmacists	Cross-sectional survey	06/15 - 06/29	India	0%	Moderate
Healthcare Practitioners and Technical Occupations (29-0000)	Mahomed et al., 2020[81]	n=404	Pharmacists	Cross-sectional survey	08/31 - 10/12	Mozambique	0.5%	High
Healthcare Practitioners and Technical Occupations (29-0000)	Martin et al., 2020[23]	n=113	Pharmacists	Cross-sectional survey	05/29 - 07/13	The United Kingdom	0%	Moderate
Healthcare Practitioners and Technical Occupations (29-0000)	Rosser et al., 2020[33]	n=213	Pharmacists	Cross-sectional survey	04/20 - 05/20	United States of America	1.88%	High
Healthcare Practitioners and Technical Occupations (29-0000)	Costa et al., 2020[128]	n=652	Physicians and Surgeons	Cross-sectional survey	05/14 - 05/28	Brazil	5.8%	Moderate
Healthcare Practitioners and Technical Occupations (29-0000)	Mohr et al., 2020[129]	n=372	Physicians and Surgeons	Cross-sectional survey	05/13 - 07/08	United States of America	1.61%	Moderate
Healthcare Practitioners and Technical Occupations (29-0000)	Nishida et al., 2020[90]	n=63	Physicians and Surgeons	Cross-sectional survey	06/12 - 06/19	Japan	3.2% (0.88-11%)	Moderate
Healthcare Practitioners and	Noor et al., 2020[130]	n=157	Physicians and Surgeons	Cross-sectional survey	07/13 - 07/15	Pakistan	17.83%	Moderate

For peer review only

Technical Occupations (29-0000)								
Healthcare Practitioners and Technical Occupations (29-0000)	Singhal et al., 2020[131]	n=208	Physicians and Surgeons	Cross-sectional survey	06/01 - 06/30	India	12.5%	High
Healthcare Practitioners and Technical Occupations (29-0000)	Morcuende et al., 2020[88]	n=23	Anesthesiologists	Cross-sectional survey	03/01 - 04/21	United States of America	13.04%	High
Healthcare Practitioners and Technical Occupations (29-0000)	Morcuende et al., 2020[88]	n=3	Obstetricians and Gynecologists	Cross-sectional survey	03/01 - 04/21	United States of America	100%	High
Healthcare Practitioners and Technical Occupations (29-0000)	Urbieta et al., 2020[132]	n=23	Pediatricians, General	Cross-sectional survey	04/14 - 04/16	Spain	4.3%	High
Healthcare Practitioners and Technical Occupations (29-0000)	Iversen et al., 2020[8]	n=1944	Psychiatrists	Cross-sectional survey	04/15 - 04/22	Denmark	1.85%	Low
Healthcare Practitioners and Technical Occupations (29-0000)	Leidner et al., 2020[22]	n=301	Surgeons	Cross sectional study with prospective cohort follow up of a subset of the sample	04/08 - 05/22	United States of America	2.66%	High
Healthcare Practitioners and Technical	Akinbami et al., 2020[46]	n=2297	Physicians and Surgeons, All Other	Cross-sectional survey	05/18 - 06/13	United States of America	6.1% (5.1-7.1%)	Moderate

For peer review only

Occupations (29-0000)								
Healthcare Practitioners and Technical Occupations (29-0000)	Alharbi et al., 2020[125]	n=18	Physicians and Surgeons, All Other	Cross-sectional survey	04/18 - 06/17	Saudi Arabia	27.78%	High
Healthcare Practitioners and Technical Occupations (29-0000)	Amendola et al., 2020[47]	n=214	Physicians and Surgeons, All Other	Cross-sectional survey	04/15 - 04/15	Italy	4.67%	High
Healthcare Practitioners and Technical Occupations (29-0000)	Baracco et al., 2020[24]	n=417	Physicians and Surgeons, All Other	Cross-sectional survey	04/23 - 05/05	Italy	17%	High
Healthcare Practitioners and Technical Occupations (29-0000)	Barallat et al., 2020[50]	n=1821	Physicians and Surgeons, All Other	Cross-sectional survey	05/04 - 05/22	Spain	11.81%	High
Healthcare Practitioners and Technical Occupations (29-0000)	Bianchi et al., 2020[133]	n=34	Physicians and Surgeons, All Other	Cross-sectional survey	04/15 - 05/15	Italy	5.88%	Unclear
Healthcare Practitioners and Technical Occupations (29-0000)	Blairon et al., 2020[52]	n=323	Physicians and Surgeons, All Other	Cross-sectional survey	05/25 - 06/19	Belgium	11.8%	High
Healthcare Practitioners and Technical Occupations (29-0000)	Brehm et al., 2020[7]	n=275	Physicians and Surgeons, All Other	Cross sectional study with prospective cohort follow up of a	03/20 - 07/17	Germany	3.3%	Moderate

For peer review only

				subset of the sample				
Healthcare Practitioners and Technical Occupations (29-0000)	Brousseau et al., 2020[134]	n=432	Physicians and Surgeons, All Other	Cross-sectional survey	07/06 - 09/24	Canada	7.2%	High
Healthcare Practitioners and Technical Occupations (29-0000)	Calcagno et al., 2020[124]	n=700	Physicians and Surgeons, All Other	Cross-sectional survey	04/17 - 05/20	Italy	7.86%	Moderate
Healthcare Practitioners and Technical Occupations (29-0000)	Chau et al., 2020[126]	n=64	Physicians and Surgeons, All Other	Cross-sectional survey	08/23 - 08/30	Viet Nam	0%	High
Healthcare Practitioners and Technical Occupations (29-0000)	Chen et al., 2020[135]	n=17	Physicians and Surgeons, All Other	Cross-sectional survey	02/19 - 02/19	China	41.18%	High
Healthcare Practitioners and Technical Occupations (29-0000)	Erber et al., 2020[31]	n=860	Physicians and Surgeons, All Other	Cross-sectional survey	04/14 - 05/29	Germany	1.63%	High
Healthcare Practitioners and Technical Occupations (29-0000)	Favara et al., 2020[136]	n=15	Physicians and Surgeons, All Other	Prospective cohort	06/01 - 06/07	The United Kingdom	13.33%	High
Healthcare Practitioners and Technical Occupations (29-0000)	Favara et al., 2020[19]	n=82	Physicians and Surgeons, All Other	Cross-sectional survey	07/13 - 07/13	The United Kingdom	10.9%	High

For peer review only

Healthcare Practitioners and Technical Occupations (29-0000)	Fujita et al., 2020[137]	n=42	Physicians and Surgeons, All Other	Cross-sectional survey	04/10 - 04/20	Japan	4.7%	High
Healthcare Practitioners and Technical Occupations (29-0000)	Galan et al., 2020[20]	n=564	Physicians and Surgeons, All Other	Cross-sectional survey	04/14 - 04/27	Spain	39.36%	High
Healthcare Practitioners and Technical Occupations (29-0000)	Godbout et al., 2020[138]	n=490	Physicians and Surgeons, All Other	Cross-sectional survey	07/27 - 10/02	United States of America	1.43%	High
Healthcare Practitioners and Technical Occupations (29-0000)	Goenka et al., 2020[25]	n=255	Physicians and Surgeons, All Other	Cross-sectional survey	07/12 - 08/23	India	3.92%	Moderate
Healthcare Practitioners and Technical Occupations (29-0000)	Goenka et al., 2020[26]	n=29	Physicians and Surgeons, All Other	Cross-sectional survey	08/01 - 08/31	India	20.69%	High
Healthcare Practitioners and Technical Occupations (29-0000)	Hanrath et al., 2020[32]	n=899	Physicians and Surgeons, All Other	Cross-sectional survey	05/29 - 07/06	The United Kingdom	7.01%	High
Healthcare Practitioners and Technical Occupations (29-0000)	Houlihan et al., 2020[139]	n=72	Physicians and Surgeons, All Other	Cross-sectional survey	03/26 - 04/08	The United Kingdom	22%	High
Healthcare Practitioners and	Hunter et al., 2020[21]	n=279	Physicians and Surgeons, All Other	Cross-sectional survey	04/29 - 05/08	United States of America	1.08%	High

For peer review only

Technical Occupations (29-0000)								
Healthcare Practitioners and Technical Occupations (29-0000)	Insua et al., 2020[140]	n=116	Physicians and Surgeons, All Other	Cross-sectional survey	06/08 - 06/09	Argentina	0.9% (0.1-5.5%)	High
Healthcare Practitioners and Technical Occupations (29-0000)	Iversen et al., 2020[8]	n=4698	Physicians and Surgeons, All Other	Cross-sectional survey	04/15 - 04/22	Denmark	4.07%	Low
Healthcare Practitioners and Technical Occupations (29-0000)	Iversen et al., 2020[8]	n=113	Physicians and Surgeons, All Other	Cross-sectional survey	04/15 - 04/22	Denmark	7.08%	Low
Healthcare Practitioners and Technical Occupations (29-0000)	Jeremias et al., 2020[70]	n=79	Physicians and Surgeons, All Other	Cross-sectional survey	03/01 - 04/30	United States of America	11.4%	High
Healthcare Practitioners and Technical Occupations (29-0000)	Kassem et al., 2020[72]	n=30	Physicians and Surgeons, All Other	Cross-sectional survey	06/01 - 06/14	Egypt	6.66%	High
Healthcare Practitioners and Technical Occupations (29-0000)	Kassem et al., 2020[72]	n=30	Physicians and Surgeons, All Other	Cross-sectional survey	06/01 - 06/14	Egypt	3.33%	High
Healthcare Practitioners and Technical	Kassem et al., 2020[72]	n=30	Physicians and Surgeons, All Other	Cross-sectional survey	06/01 - 06/14	Egypt	0%	High

For peer review only

Occupations (29-0000)								
Healthcare Practitioners and Technical Occupations (29-0000)	Kassem et al., 2020[72]	n=30	Physicians and Surgeons, All Other	Cross-sectional survey	06/01 - 06/14	Egypt	3.33%	High
Healthcare Practitioners and Technical Occupations (29-0000)	Khan et al., 2020[127]	n=980	Physicians and Surgeons, All Other	Cross-sectional survey	06/15 - 06/29	India	2.8% (1.9-4%)	Moderate
Healthcare Practitioners and Technical Occupations (29-0000)	Kohler et al., 2020[141]	n=268	Physicians and Surgeons, All Other	Cross-sectional survey	03/19 - 04/03	Switzerland	1.49%	High
Healthcare Practitioners and Technical Occupations (29-0000)	Kumar et al., 2020[142]	n=201	Physicians and Surgeons, All Other	Cross-sectional survey	06/01 - 06/30	India	7% (4.2-11.4%)	High
Healthcare Practitioners and Technical Occupations (29-0000)	Leidner et al., 2020[22]	n=1081	Physicians and Surgeons, All Other	Cross sectional study with prospective cohort follow up of a subset of the sample	04/08 - 05/22	United States of America	3.33%	High
Healthcare Practitioners and Technical Occupations (29-0000)	Lumley et al., 2020[9]	n=1859	Physicians and Surgeons, All Other	Prospective cohort	04/23 - 11/30	The United Kingdom	10.11%	Moderate
Healthcare Practitioners and Technical	Martin et al., 2020[23]	n=1243	Physicians and Surgeons, All Other	Cross-sectional survey	05/29 - 07/13	The United Kingdom	10.3%	Moderate

For peer review only

Occupations (29-0000)								
Healthcare Practitioners and Technical Occupations (29-0000)	Mesnil et al., 2020[143]	n=111	Physicians and Surgeons, All Other	Cross-sectional survey	06/08 - 06/22	France	11%	High
Healthcare Practitioners and Technical Occupations (29-0000)	Missaglia et al., 2020[144]	n=377	Physicians and Surgeons, All Other	Cross-sectional survey	04/01 - 04/30	Italy	14.9%	High
Healthcare Practitioners and Technical Occupations (29-0000)	Mohr et al., 2020[129]	n=272	Physicians and Surgeons, All Other	Cross-sectional survey	05/13 - 07/08	United States of America	2.94%	Moderate
Healthcare Practitioners and Technical Occupations (29-0000)	Moscola et al., 2020[89]	n=3746	Physicians and Surgeons, All Other	Cross-sectional survey	04/20 - 06/23	United States of America	8.7%	High
Healthcare Practitioners and Technical Occupations (29-0000)	Nishida et al., 2020[90]	n=149	Physicians and Surgeons, All Other	Cross-sectional survey	06/12 - 06/19	Japan	1.3% (0.37-4.8%)	Moderate
Healthcare Practitioners and Technical Occupations (29-0000)	Nishida et al., 2020[90]	n=46	Physicians and Surgeons, All Other	Cross-sectional survey	06/12 - 06/19	Japan	0%	Moderate
Healthcare Practitioners and Technical Occupations (29-0000)	Nishida et al., 2020[90]	n=40	Physicians and Surgeons, All Other	Cross-sectional survey	06/12 - 06/19	Japan	0%	Moderate

For peer review only

Healthcare Practitioners and Technical Occupations (29-0000)	Nishida et al., 2020[90]	n=59	Physicians and Surgeons, All Other	Cross-sectional survey	06/12 - 06/19	Japan	1.7% (0.3-9%)	Moderate
Healthcare Practitioners and Technical Occupations (29-0000)	Nishida et al., 2020[90]	n=925	Physicians and Surgeons, All Other	Cross-sectional survey	06/12 - 06/19	Japan	0.43% (0.17-1.1%)	Moderate
Healthcare Practitioners and Technical Occupations (29-0000)	Noor et al., 2020[130]	n=303	Physicians and Surgeons, All Other	Cross-sectional survey	07/13 - 07/15	Pakistan	19.8%	Moderate
Healthcare Practitioners and Technical Occupations (29-0000)	Orth-Holler et al., 2020[145]	n=377	Physicians and Surgeons, All Other	Cross-sectional survey	03/20 - 03/27	Austria	0.3% (0.01-1.5%)	High
Healthcare Practitioners and Technical Occupations (29-0000)	Plebani et al., 2020[146]	n=2337	Physicians and Surgeons, All Other	Cross-sectional survey	02/22 - 05/29	Italy	3.6% (2.8-4.4%)	High
Healthcare Practitioners and Technical Occupations (29-0000)	Rosser et al., 2020[33]	n=2533	Physicians and Surgeons, All Other	Cross-sectional survey	04/20 - 05/20	United States of America	1.07%	High
Healthcare Practitioners and Technical Occupations (29-0000)	Rudberg et al., 2020[147]	n=439	Physicians and Surgeons, All Other	Cross-sectional survey	04/14 - 05/08	Sweden	19.1%	Moderate
Healthcare Practitioners and	Schmidt et al., 2020[148]	n=34	Physicians and Surgeons, All Other	Cross-sectional survey	04/20 - 04/30	Germany	8.82%	High

For peer review only

Technical Occupations (29-0000)								
Healthcare Practitioners and Technical Occupations (29-0000)	Sotgiu et al., 2020[149]	n=115	Physicians and Surgeons, All Other	Cross-sectional survey	04/02 - 04/16	Italy	6.09%	High
Healthcare Practitioners and Technical Occupations (29-0000)	Venugopal et al., 2020[150]	n=157	Physicians and Surgeons, All Other	Cross-sectional survey	03/01 - 05/01	United States of America	25%	Moderate
Healthcare Practitioners and Technical Occupations (29-0000)	Yogo et al., 2020[36]	n=110	Physicians and Surgeons, All Other	Cross-sectional survey	05/20 - 06/08	United States of America	1.82%	High
Healthcare Practitioners and Technical Occupations (29-0000)	Brzostek et al., 2020[151]	n=998	Physician Assistants	Cross-sectional survey	04/17 - 05/07	United States of America	28.3%	Moderate
Healthcare Practitioners and Technical Occupations (29-0000)	Hoffmann et al., 2020[152]	n=156	Physician Assistants	Prospective cohort	07/01 - 07/31	Germany	1.3%	High
Healthcare Practitioners and Technical Occupations (29-0000)	Mohr et al., 2020[129]	n=156	Physician Assistants	Cross-sectional survey	05/13 - 07/08	United States of America	0.64%	Moderate
Healthcare Practitioners and Technical	Morcuende et al., 2020[88]	n=6	Physician Assistants	Cross-sectional survey	03/01 - 04/21	United States of America	9.43%	High

For peer review only

Occupations (29-0000)								
Healthcare Practitioners and Technical Occupations (29-0000)	Morcuende et al., 2020[88]	n=53	Physician Assistants	Cross-sectional survey	03/01 - 04/21	United States of America	9.43%	High
Healthcare Practitioners and Technical Occupations (29-0000)	Patel et al., 2020[153]	n=230	Physician Assistants	Prospective cohort	06/02 - 06/27	United States of America	3.48%	High
Healthcare Practitioners and Technical Occupations (29-0000)	Self et al., 2020[154]	n=919	Physician Assistants	Cross-sectional survey	04/03 - 06/19	United States of America	5.66%	Moderate
Healthcare Practitioners and Technical Occupations (29-0000)	Shah et al., 2020[155]	n=248	Physician Assistants	Cross-sectional survey	05/25 - 07/09	United States of America	0%	High
Healthcare Practitioners and Technical Occupations (29-0000)	Shah et al., 2020[155]	n=320	Physician Assistants	Cross-sectional survey	05/25 - 07/09	United States of America	0.63%	High
Healthcare Practitioners and Technical Occupations (29-0000)	Lumley et al., 2020[9]	n=386	Occupational Therapists	Prospective cohort	04/23 - 11/30	The United Kingdom	11.4%	Moderate
Healthcare Practitioners and Technical Occupations (29-0000)	Akinbami et al., 2020[46]	n=235	Physical Therapists	Cross-sectional survey	05/18 - 06/13	United States of America	10.6% (7-15.3%)	Moderate

For peer review only

Healthcare Practitioners and Technical Occupations (29-0000)	Brehm et al., 2020[7]	n=15	Physical Therapists	Cross sectional study with prospective cohort follow up of a subset of the sample	03/20 - 07/17	Germany	0%	Moderate
Healthcare Practitioners and Technical Occupations (29-0000)	Cooper et al., 2020[59]	n=84	Physical Therapists	Cross-sectional survey	06/10 - 08/07	The United Kingdom	10.71%	Moderate
Healthcare Practitioners and Technical Occupations (29-0000)	Costa et al., 2020[128]	n=159	Physical Therapists	Cross-sectional survey	05/14 - 05/28	Brazil	10.7%	Moderate
Healthcare Practitioners and Technical Occupations (29-0000)	Akinbami et al., 2020[46]	n=409	Respiratory Therapists	Cross-sectional survey	05/18 - 06/13	United States of America	8.3% (5.8-11.4%)	Moderate
Healthcare Practitioners and Technical Occupations (29-0000)	Brunner et al., 2020[54]	n=42	Respiratory Therapists	Cross-sectional survey	05/04 - 05/29	United States of America	0%	High
Healthcare Practitioners and Technical Occupations (29-0000)	Godbout et al., 2020[138]	n=25	Respiratory Therapists	Cross-sectional survey	07/27 - 10/02	United States of America	0%	High
Healthcare Practitioners and Technical Occupations (29-0000)	Hunter et al., 2020[21]	n=94	Respiratory Therapists	Cross-sectional survey	04/29 - 05/08	United States of America	0%	High

For peer review only

Healthcare Practitioners and Technical Occupations (29-0000)	Rosser et al., 2020[33]	n=135	Respiratory Therapists	Cross-sectional survey	04/20 - 05/20	United States of America	0%	High
Healthcare Practitioners and Technical Occupations (29-0000)	Self et al., 2020[154]	n=235	Respiratory Therapists	Cross-sectional survey	04/03 - 06/19	United States of America	4.26%	Moderate
Healthcare Practitioners and Technical Occupations (29-0000)	Yogo et al., 2020[36]	n=121	Respiratory Therapists	Cross-sectional survey	05/20 - 06/08	United States of America	0%	High
Healthcare Practitioners and Technical Occupations (29-0000)	Rosser et al., 2020[33]	n=253	Therapists, All Other	Cross-sectional survey	04/20 - 05/20	United States of America	1.58%	High
Healthcare Practitioners and Technical Occupations (29-0000)	Schmidt et al., 2020[148]	n=80	Therapists, All Other	Cross-sectional survey	04/20 - 04/30	Germany	3.75%	High
Healthcare Practitioners and Technical Occupations (29-0000)	Yogo et al., 2020[36]	n=22	Therapists, All Other	Cross-sectional survey	05/20 - 06/08	United States of America	4.55%	High
Healthcare Practitioners and Technical Occupations (29-0000)	Calcagno et al., 2020[124]	n=13	Veterinarians	Cross-sectional survey	04/17 - 05/20	Italy	0%	Moderate
Healthcare Practitioners and	Akinbami et al., 2020[46]	n=6426	Registered Nurses	Cross-sectional survey	05/18 - 06/13	United States of America	7.7% (7.1-8.4%)	Moderate

For peer review only

Technical Occupations (29-0000)								
Healthcare Practitioners and Technical Occupations (29-0000)	Alharbi et al., 2020[125]	n=70	Registered Nurses	Cross-sectional survey	04/18 - 06/17	Saudi Arabia	10%	High
Healthcare Practitioners and Technical Occupations (29-0000)	Alharbi et al., 2020[125]	n=9	Registered Nurses	Cross-sectional survey	04/18 - 06/17	Saudi Arabia	33.33%	High
Healthcare Practitioners and Technical Occupations (29-0000)	Alharbi et al., 2020[125]	n=76	Registered Nurses	Cross-sectional survey	04/18 - 06/17	Saudi Arabia	26.32%	High
Healthcare Practitioners and Technical Occupations (29-0000)	Alharbi et al., 2020[125]	n=21	Registered Nurses	Cross-sectional survey	04/18 - 06/17	Saudi Arabia	14.29%	High
Healthcare Practitioners and Technical Occupations (29-0000)	Alharbi et al., 2020[125]	n=43	Registered Nurses	Cross-sectional survey	04/18 - 06/17	Saudi Arabia	27.91%	High
Healthcare Practitioners and Technical Occupations (29-0000)	Amendola et al., 2020[47]	n=216	Registered Nurses	Cross-sectional survey	04/15 - 04/15	Italy	6.02%	High
Healthcare Practitioners and Technical	Bampoe et al., 2020[156]	n=52	Registered Nurses	Cross-sectional survey	05/11 - 06/05	The United Kingdom	13.5% (5.6-25.8%)	High

For peer review only

Occupations (29-0000)								
Healthcare Practitioners and Technical Occupations (29-0000)	Bampoe et al., 2020[156]	n=40	Registered Nurses	Cross-sectional survey	05/11 - 06/05	The United Kingdom	12.5% (4.2-26.8%)	High
Healthcare Practitioners and Technical Occupations (29-0000)	Baracco et al., 2020[24]	n=1014	Registered Nurses	Cross-sectional survey	04/23 - 05/05	Italy	17.9%	High
Healthcare Practitioners and Technical Occupations (29-0000)	Barallat et al., 2020[50]	n=2243	Registered Nurses	Cross-sectional survey	05/04 - 05/22	Spain	10.64%	High
Healthcare Practitioners and Technical Occupations (29-0000)	Brehm et al., 2020[7]	n=444	Registered Nurses	Cross sectional study with prospective cohort follow up of a subset of the sample	03/20 - 07/17	Germany	2.3%	Moderate
Healthcare Practitioners and Technical Occupations (29-0000)	Brousseau et al., 2020[134]	n=1189	Registered Nurses	Cross-sectional survey	07/06 - 09/24	Canada	11.9%	High
Healthcare Practitioners and Technical Occupations (29-0000)	Calcagno et al., 2020[124]	n=1833	Registered Nurses	Cross-sectional survey	04/17 - 05/20	Italy	8.18%	Moderate
Healthcare Practitioners and Technical	Chau et al., 2020[126]	n=144	Registered Nurses	Cross-sectional survey	08/23 - 08/30	Viet Nam	0%	High

For peer review only

Occupations (29-0000)								
Healthcare Practitioners and Technical Occupations (29-0000)	Chen et al., 2020[135]	n=25	Registered Nurses	Cross-sectional survey	02/19 - 02/19	China	8%	High
Healthcare Practitioners and Technical Occupations (29-0000)	Cooper et al., 2020[59]	n=3471	Registered Nurses	Cross-sectional survey	06/10 - 08/07	The United Kingdom	7.52%	Moderate
Healthcare Practitioners and Technical Occupations (29-0000)	Costa et al., 2020[128]	n=370	Registered Nurses	Cross-sectional survey	05/14 - 05/28	Brazil	11.4%	Moderate
Healthcare Practitioners and Technical Occupations (29-0000)	Dimcheff et al., 2020[157]	n=412	Registered Nurses	Cross-sectional survey	06/08 - 07/08	United States of America	7%	Moderate
Healthcare Practitioners and Technical Occupations (29-0000)	Erber et al., 2020[31]	n=958	Registered Nurses	Cross-sectional survey	04/14 - 05/29	Germany	2.5%	High
Healthcare Practitioners and Technical Occupations (29-0000)	Favara et al., 2020[136]	n=45	Registered Nurses	Prospective cohort	06/01 - 06/07	The United Kingdom	28.89%	High
Healthcare Practitioners and Technical Occupations (29-0000)	Favara et al., 2020[19]	n=237	Registered Nurses	Cross-sectional survey	07/13 - 07/13	The United Kingdom	16.5%	High

For peer review only

Healthcare Practitioners and Technical Occupations (29-0000)	Finkenzeller et al., 2020[158]	n=251	Registered Nurses	Prospective cohort	06/29 - 07/29	Germany	12%	Moderate
Healthcare Practitioners and Technical Occupations (29-0000)	Finkenzeller et al., 2020[158]	n=887	Registered Nurses	Prospective cohort	06/29 - 07/29	Germany	20%	Moderate
Healthcare Practitioners and Technical Occupations (29-0000)	Fujita et al., 2020[137]	n=50	Registered Nurses	Cross-sectional survey	04/10 - 04/20	Japan	6%	High
Healthcare Practitioners and Technical Occupations (29-0000)	Galan et al., 2020[20]	n=687	Registered Nurses	Cross-sectional survey	04/14 - 04/27	Spain	30.71%	High
Healthcare Practitioners and Technical Occupations (29-0000)	Godbout et al., 2020[138]	n=937	Registered Nurses	Cross-sectional survey	07/27 - 10/02	United States of America	1.39%	High
Healthcare Practitioners and Technical Occupations (29-0000)	Goenka et al., 2020[25]	n=224	Registered Nurses	Cross-sectional survey	07/12 - 08/23	India	9.38%	Moderate
Healthcare Practitioners and Technical Occupations (29-0000)	Goenka et al., 2020[26]	n=43	Registered Nurses	Cross-sectional survey	08/01 - 08/31	India	34.88%	High
Healthcare Practitioners and	Grant et al., 2020[159]	n=1345	Registered Nurses	Cross-sectional survey	05/15 - 06/05	The United Kingdom	34.7%	High

For peer review only

Technical Occupations (29-0000)								
Healthcare Practitioners and Technical Occupations (29-0000)	Grant et al., 2020[159]	n=108	Registered Nurses	Cross-sectional survey	05/15 - 06/05	The United Kingdom	25%	High
Healthcare Practitioners and Technical Occupations (29-0000)	Hanrath et al., 2020[32]	n=749	Registered Nurses	Cross-sectional survey	05/29 - 07/06	The United Kingdom	8.99%	High
Healthcare Practitioners and Technical Occupations (29-0000)	Haq et al., 2020[67]	n=209	Registered Nurses	Cross-sectional survey	06/15 - 06/29	Pakistan	38.8% (32.1-45.7%)	Moderate
Healthcare Practitioners and Technical Occupations (29-0000)	Houlihan et al., 2020[139]	n=106	Registered Nurses	Cross-sectional survey	03/26 - 04/08	The United Kingdom	24%	High
Healthcare Practitioners and Technical Occupations (29-0000)	Houlihan et al., 2020[139]	n=22	Registered Nurses	Cross-sectional survey	03/26 - 04/08	The United Kingdom	23%	High
Healthcare Practitioners and Technical Occupations (29-0000)	Hunter et al., 2020[21]	n=317	Registered Nurses	Cross-sectional survey	04/29 - 05/08	United States of America	2.2%	High
Healthcare Practitioners and Technical	Iversen et al., 2020[8]	n=9963	Registered Nurses	Cross-sectional survey	04/15 - 04/22	Denmark	4.03%	Low

For peer review only

Occupations (29-0000)								
Healthcare Practitioners and Technical Occupations (29-0000)	Iversen et al., 2020[8]	n=1786	Registered Nurses	Cross-sectional survey	04/15 - 04/22	Denmark	4.65%	Low
Healthcare Practitioners and Technical Occupations (29-0000)	Jeremias et al., 2020[70]	n=1043	Registered Nurses	Cross-sectional survey	03/01 - 04/30	United States of America	9.5%	High
Healthcare Practitioners and Technical Occupations (29-0000)	Jones et al., 2020[29]	n=1962	Registered Nurses	Cross-sectional survey	01/15 - 06/15	The United Kingdom	10.5%	High
Healthcare Practitioners and Technical Occupations (29-0000)	Kassem et al., 2020[72]	n=28	Registered Nurses	Cross-sectional survey	06/01 - 06/14	Egypt	10.71%	High
Healthcare Practitioners and Technical Occupations (29-0000)	Kassem et al., 2020[72]	n=28	Registered Nurses	Cross-sectional survey	06/01 - 06/14	Egypt	7.14%	High
Healthcare Practitioners and Technical Occupations (29-0000)	Kassem et al., 2020[72]	n=28	Registered Nurses	Cross-sectional survey	06/01 - 06/14	Egypt	3.57%	High
Healthcare Practitioners and Technical Occupations (29-0000)	Kassem et al., 2020[72]	n=28	Registered Nurses	Cross-sectional survey	06/01 - 06/14	Egypt	0%	High

For peer review only

Healthcare Practitioners and Technical Occupations (29-0000)	Khan et al., 2020[127]	n=321	Registered Nurses	Cross-sectional survey	06/15 - 06/29	India	2.8% (1.5-5.3%)	Moderate
Healthcare Practitioners and Technical Occupations (29-0000)	Kohler et al., 2020[141]	n=398	Registered Nurses	Cross-sectional survey	03/19 - 04/03	Switzerland	0.75%	High
Healthcare Practitioners and Technical Occupations (29-0000)	Kumar et al., 2020[142]	n=308	Registered Nurses	Cross-sectional survey	06/01 - 06/30	India	6.8% (4.5-10.2%)	High
Healthcare Practitioners and Technical Occupations (29-0000)	Leidner et al., 2020[22]	n=110	Registered Nurses	Cross sectional study with prospective cohort follow up of a subset of the sample	04/08 - 05/22	United States of America	0%	High
Healthcare Practitioners and Technical Occupations (29-0000)	Leidner et al., 2020[22]	n=3504	Registered Nurses	Cross sectional study with prospective cohort follow up of a subset of the sample	04/08 - 05/22	United States of America	2.34%	High
Healthcare Practitioners and Technical Occupations (29-0000)	Lumley et al., 2020[9]	n=4528	Registered Nurses	Prospective cohort	04/23 - 11/30	The United Kingdom	13.21%	Moderate
Healthcare Practitioners and Technical Occupations (29-0000)	Mansour et al., 2020[160]	n=285	Registered Nurses	Cross-sectional survey	03/24 - 04/04	United States of America	32.63%	High

For peer review only

Healthcare Practitioners and Technical Occupations (29-0000)	Martin et al., 2020[161]	n=580	Registered Nurses	Cross-sectional survey	04/01 - 04/15	Spain	5.52%	High
Healthcare Practitioners and Technical Occupations (29-0000)	Martin et al., 2020[161]	n=74	Registered Nurses	Cross-sectional survey	04/01 - 04/15	Spain	9.46%	High
Healthcare Practitioners and Technical Occupations (29-0000)	Martin et al., 2020[161]	n=676	Registered Nurses	Cross-sectional survey	04/01 - 04/15	Spain	5.92%	High
Healthcare Practitioners and Technical Occupations (29-0000)	Martin et al., 2020[161]	n=337	Registered Nurses	Cross-sectional survey	04/01 - 04/15	Spain	5.93%	High
Healthcare Practitioners and Technical Occupations (29-0000)	Martin et al., 2020[161]	n=339	Registered Nurses	Cross-sectional survey	04/01 - 04/15	Spain	5.9%	High
Healthcare Practitioners and Technical Occupations (29-0000)	Meissner et al., 2020[162]	n=439	Registered Nurses	Cross-sectional survey	04/14 - 05/06	United States of America	1.37%	High
Healthcare Practitioners and Technical Occupations (29-0000)	Mohr et al., 2020[129]	n=410	Registered Nurses	Cross-sectional survey	05/13 - 07/08	United States of America	1.46%	Moderate
Healthcare Practitioners and	Moscola et al., 2020[89]	n=11468	Registered Nurses	Cross-sectional survey	04/20 - 06/23	United States of America	13.1%	High

For peer review only

Technical Occupations (29-0000)								
Healthcare Practitioners and Technical Occupations (29-0000)	Mostafa et al., 2020[163]	n=4040	Registered Nurses	Cross-sectional survey	04/22 - 05/14	Egypt	1.31%	High
Healthcare Practitioners and Technical Occupations (29-0000)	Nishida et al., 2020[90]	n=489	Registered Nurses	Cross-sectional survey	06/12 - 06/19	Japan	0.2% (0.04-1.1%)	Moderate
Healthcare Practitioners and Technical Occupations (29-0000)	Noor et al., 2020[130]	n=460	Registered Nurses	Cross-sectional survey	07/13 - 07/15	Pakistan	39.78%	Moderate
Healthcare Practitioners and Technical Occupations (29-0000)	Paradiso et al., 2020[164]	n=606	Registered Nurses	Cross sectional study with prospective cohort follow up of a subset of the sample	03/26 - 04/17	Italy	0.33%	High
Healthcare Practitioners and Technical Occupations (29-0000)	Plebani et al., 2020[146]	n=3230	Registered Nurses	Cross-sectional survey	02/22 - 05/29	Italy	4.7% (4-5.5%)	High
Healthcare Practitioners and Technical Occupations (29-0000)	Poustchi et al., 2020[28]	n=1245	Registered Nurses	Cross-sectional survey	04/17 - 06/02	Iran (Islamic Republic of)	15.9% (13.9-18%)	Moderate
Healthcare Practitioners and Technical	Rudberg et al., 2020[147]	n=636	Registered Nurses	Cross-sectional survey	04/14 - 05/08	Sweden	21.9%	Moderate

For peer review only

Occupations (29-0000)								
Healthcare Practitioners and Technical Occupations (29-0000)	Schmidt et al., 2020[148]	n=154	Registered Nurses	Cross-sectional survey	04/20 - 04/30	Germany	0%	High
Healthcare Practitioners and Technical Occupations (29-0000)	Self et al., 2020[154]	n=1445	Registered Nurses	Cross-sectional survey	04/03 - 06/19	United States of America	5.05%	Moderate
Healthcare Practitioners and Technical Occupations (29-0000)	Siddiqui et al., 2020[2]	n=59	Registered Nurses	Prospective cohort	04/15 - 08/15	India	10.2%	High
Healthcare Practitioners and Technical Occupations (29-0000)	Siddiqui et al., 2020[2]	n=70	Registered Nurses	Prospective cohort	04/15 - 08/15	India	10%	High
Healthcare Practitioners and Technical Occupations (29-0000)	Sotgiu et al., 2020[149]	n=64	Registered Nurses	Cross-sectional survey	04/02 - 04/16	Italy	7.8% (1.2-14.4%)	High
Healthcare Practitioners and Technical Occupations (29-0000)	Sydney et al., 2020[165]	n=81	Registered Nurses	Cross-sectional survey	04/28 - 05/04	United States of America	18.52%	High
Healthcare Practitioners and Technical Occupations (29-0000)	Urbieta et al., 2020[132]	n=83	Registered Nurses	Cross-sectional survey	04/14 - 04/16	Spain	4.8%	High

Healthcare Practitioners and Technical Occupations (29-0000)	Urbieta et al., 2020[132]	n=23	Registered Nurses	Cross-sectional survey	04/14 - 04/16	Spain	8.7%	High
Healthcare Practitioners and Technical Occupations (29-0000)	Venugopal et al., 2020[150]	n=142	Registered Nurses	Cross-sectional survey	03/01 - 05/01	United States of America	28%	Moderate
Healthcare Practitioners and Technical Occupations (29-0000)	Yogo et al., 2020[36]	n=1129	Registered Nurses	Cross-sectional survey	05/20 - 06/08	United States of America	2.48%	High
Healthcare Practitioners and Technical Occupations (29-0000)	Yogo et al., 2020[36]	n=12	Registered Nurses	Cross-sectional survey	05/20 - 06/08	United States of America	0%	High
Healthcare Practitioners and Technical Occupations (29-0000)	Zhou et al., 2020[166]	n=2406	Registered Nurses	Cross-sectional survey	03/16 - 03/25	China	1.37%	Moderate
Healthcare Practitioners and Technical Occupations (29-0000)	Godbout et al., 2020[138]	n=141	Nurse Practitioners	Cross-sectional survey	07/27 - 10/02	United States of America	1.42%	High
Healthcare Practitioners and Technical Occupations (29-0000)	Dimcheff et al., 2020[157]	n=214	Nurse Practitioners	Cross-sectional survey	06/08 - 07/08	United States of America	3.7%	Moderate
Healthcare Practitioners and	Akinbami et al., 2020[46]	n=719	Health Technologists and Technicians	Cross-sectional survey	05/18 - 06/13	United States of America	4.2% (2.8-5.9%)	Moderate

For peer review only

Technical Occupations (29-0000)								
Healthcare Practitioners and Technical Occupations (29-0000)	Blairon et al., 2020[52]	n=61	Health Technologists and Technicians	Cross-sectional survey	05/25 - 06/19	Belgium	6.6%	High
Healthcare Practitioners and Technical Occupations (29-0000)	Yogo et al., 2020[36]	n=65	Health Technologists and Technicians	Cross-sectional survey	05/20 - 06/08	United States of America	4.62%	High
Healthcare Practitioners and Technical Occupations (29-0000)	Silva et al., 2020[34]	n=224	Clinical Laboratory Technologists and Technicians	Cross-sectional survey	06/05 - 07/31	Brazil	7.59%	High
Healthcare Practitioners and Technical Occupations (29-0000)	Costa et al., 2020[128]	n=66	Medical and Clinical Laboratory Technologists	Cross-sectional survey	05/14 - 05/28	Brazil	3%	Moderate
Healthcare Practitioners and Technical Occupations (29-0000)	Akinbami et al., 2020[46]	n=293	Medical and Clinical Laboratory Technicians	Cross-sectional survey	05/18 - 06/13	United States of America	3.4% (1.7-6.2%)	Moderate
Healthcare Practitioners and Technical Occupations (29-0000)	Akinbami et al., 2020[46]	n=365	Medical and Clinical Laboratory Technicians	Cross-sectional survey	05/18 - 06/13	United States of America	5.5% (3.4-8.3%)	Moderate
Healthcare Practitioners and Technical	Alharbi et al., 2020[125]	n=80	Medical and Clinical Laboratory Technicians	Cross-sectional survey	04/18 - 06/17	Saudi Arabia	20%	High

For peer review only

Occupations (29-0000)								
Healthcare Practitioners and Technical Occupations (29-0000)	Baracco et al., 2020[24]	n=256	Medical and Clinical Laboratory Technicians	Cross-sectional survey	04/23 - 05/05	Italy	12.1%	High
Healthcare Practitioners and Technical Occupations (29-0000)	Brehm et al., 2020[7]	n=105	Medical and Clinical Laboratory Technicians	Cross sectional study with prospective cohort follow up of a subset of the sample	03/20 - 07/17	Germany	0%	Moderate
Healthcare Practitioners and Technical Occupations (29-0000)	Calcagno et al., 2020[124]	n=216	Medical and Clinical Laboratory Technicians	Cross-sectional survey	04/17 - 05/20	Italy	6.94%	Moderate
Healthcare Practitioners and Technical Occupations (29-0000)	Calcagno et al., 2020[124]	n=157	Medical and Clinical Laboratory Technicians	Cross-sectional survey	04/17 - 05/20	Italy	11.46%	Moderate
Healthcare Practitioners and Technical Occupations (29-0000)	Chau et al., 2020[126]	n=33	Medical and Clinical Laboratory Technicians	Cross-sectional survey	08/23 - 08/30	Viet Nam	0%	High
Healthcare Practitioners and Technical Occupations (29-0000)	Galan et al., 2020[20]	n=192	Medical and Clinical Laboratory Technicians	Cross-sectional survey	04/14 - 04/27	Spain	21.35%	High
Healthcare Practitioners and Technical	Goenka et al., 2020[25]	n=72	Medical and Clinical Laboratory Technicians	Cross-sectional survey	07/12 - 08/23	India	15.28%	Moderate

For peer review only

Occupations (29-0000)								
Healthcare Practitioners and Technical Occupations (29-0000)	Haq et al., 2020[67]	n=32	Medical and Clinical Laboratory Technicians	Cross-sectional survey	06/15 - 06/29	Pakistan	50% (31.8-68.1%)	Moderate
Healthcare Practitioners and Technical Occupations (29-0000)	Iversen et al., 2020[8]	n=1292	Medical and Clinical Laboratory Technicians	Cross-sectional survey	04/15 - 04/22	Denmark	1.93%	Low
Healthcare Practitioners and Technical Occupations (29-0000)	Khan et al., 2020[127]	n=397	Medical and Clinical Laboratory Technicians	Cross-sectional survey	06/15 - 06/29	India	2.5% (1.4-4.6%)	Moderate
Healthcare Practitioners and Technical Occupations (29-0000)	Lumley et al., 2020[9]	n=452	Medical and Clinical Laboratory Technicians	Prospective cohort	04/23 - 11/30	The United Kingdom	8.63%	Moderate
Healthcare Practitioners and Technical Occupations (29-0000)	Nishida et al., 2020[90]	n=140	Medical and Clinical Laboratory Technicians	Cross-sectional survey	06/12 - 06/19	Japan	0%	Moderate
Healthcare Practitioners and Technical Occupations (29-0000)	Rosser et al., 2020[33]	n=225	Medical and Clinical Laboratory Technicians	Cross-sectional survey	04/20 - 05/20	United States of America	0.44%	High
Healthcare Practitioners and Technical Occupations (29-0000)	Iversen et al., 2020[8]	n=342	Radiologic Technologists	Cross-sectional survey	04/15 - 04/22	Denmark	3.51%	Low

For peer review only

Healthcare Practitioners and Technical Occupations (29-0000)	Martin et al., 2020[23]	n=241	Radiologic Technologists	Cross-sectional survey	05/29 - 07/13	The United Kingdom	9.96%	Moderate
Healthcare Practitioners and Technical Occupations (29-0000)	Akinbami et al., 2020[46]	n=1158	Emergency Medical Technicians and Paramedics	Cross-sectional survey	05/18 - 06/13	United States of America	5.2% (4-6.6%)	Moderate
Healthcare Practitioners and Technical Occupations (29-0000)	Buntinx et al., 2020[167]	n=10	Emergency Medical Technicians and Paramedics	Cross-sectional survey	04/14 - 04/16	Belgium	10%	High
Healthcare Practitioners and Technical Occupations (29-0000)	Haq et al., 2020[67]	n=157	Emergency Medical Technicians and Paramedics	Cross-sectional survey	06/15 - 06/29	Pakistan	42% (34.2-50.1%)	Moderate
Healthcare Practitioners and Technical Occupations (29-0000)	Iversen et al., 2020[8]	n=323	Emergency Medical Technicians and Paramedics	Cross-sectional survey	04/15 - 04/22	Denmark	4.95%	Low
Healthcare Practitioners and Technical Occupations (29-0000)	Mesnil et al., 2020[143]	n=212	Emergency Medical Technicians and Paramedics	Cross-sectional survey	06/08 - 06/22	France	11%	High
Healthcare Practitioners and Technical Occupations (29-0000)	Reuben et al., 2020[168]	n=10	Emergency Medical Technicians and Paramedics	Cross-sectional survey	05/28 - 07/15	United States of America	0%	High

For peer review only

Healthcare Practitioners and Technical Occupations (29-0000)	Saberian et al., 2020[169]	n=243	Emergency Medical Technicians and Paramedics	Cross-sectional survey	03/20 - 05/20	Iran (Islamic Republic of)	41.56%	High
Healthcare Practitioners and Technical Occupations (29-0000)	Self et al., 2020[154]	n=56	Emergency Medical Technicians and Paramedics	Cross-sectional survey	04/03 - 06/19	United States of America	5.36%	Moderate
Healthcare Practitioners and Technical Occupations (29-0000)	Tarabichi et al., 2020[170]	n=111	Emergency Medical Technicians and Paramedics	Cross-sectional survey	04/20 - 05/19	United States of America	5.41%	High
Healthcare Practitioners and Technical Occupations (29-0000)	Baracco et al., 2020[24]	n=188	Health Technologists and Technicians, All Other	Cross-sectional survey	04/23 - 05/05	Italy	13.8%	High
Healthcare Practitioners and Technical Occupations (29-0000)	Chau et al., 2020[126]	n=22	Health Technologists and Technicians, All Other	Cross-sectional survey	08/23 - 08/30	Viet Nam	0%	High
Healthcare Practitioners and Technical Occupations (29-0000)	Goenka et al., 2020[25]	n=99	Health Technologists and Technicians, All Other	Cross-sectional survey	07/12 - 08/23	India	12.12%	Moderate
Healthcare Practitioners and Technical Occupations (29-0000)	Goenka et al., 2020[26]	n=16	Health Technologists and Technicians, All Other	Cross-sectional survey	08/01 - 08/31	India	68.75%	High
Healthcare Support	Jeremias et al., 2020[70]	n=155	Healthcare Support Occupations	Cross-sectional survey	03/01 - 04/30	United States of America	5.8%	High

For peer review only

Occupations (31-0000)								
Healthcare Support Occupations (31-0000)	Ward et al., 2020[113]	n=979	Nursing, Psychiatric, and Home Health Aides	Cross-sectional survey	09/15 - 09/28	The United Kingdom	11.09% (8.96-13.59%)	Moderate
Healthcare Support Occupations (31-0000)	Ward et al., 2020[113]	n=257	Nursing, Psychiatric, and Home Health Aides	Cross-sectional survey	09/15 - 09/28	The United Kingdom	8.95%	Moderate
Healthcare Support Occupations (31-0000)	Vijh et al., 2020[171]	n=169	Nursing, Psychiatric, and Home Health Aides	Cross-sectional survey	05/04 - 05/14	Canada	26.63%	High
Healthcare Support Occupations (31-0000)	Akinbami et al., 2020[46]	n=641	Nursing Assistants	Cross-sectional survey	05/18 - 06/13	United States of America	12.8% (10.3-15.6%)	Moderate
Healthcare Support Occupations (31-0000)	Bampoe et al., 2020[156]	n=108	Nursing Assistants	Cross-sectional survey	05/11 - 06/05	The United Kingdom	15.7% (9.5-24%)	High
Healthcare Support Occupations (31-0000)	Baracco et al., 2020[24]	n=257	Nursing Assistants	Cross-sectional survey	04/23 - 05/05	Italy	22.2%	High
Healthcare Support Occupations (31-0000)	Barallat et al., 2020[50]	n=832	Nursing Assistants	Cross-sectional survey	05/04 - 05/22	Spain	13.94%	High
Healthcare Support Occupations (31-0000)	Bhattacharya et al., 2020[172]	n=121	Nursing Assistants	Cross-sectional survey	06/01 - 06/15	United States of America	1.65%	High
Healthcare Support	Brousseau et al., 2020[134]	n=132	Nursing Assistants	Cross-sectional survey	07/06 - 09/24	Canada	16.7%	High

For peer review only

Occupations (31-0000)								
Healthcare Support Occupations (31-0000)	Brunner et al., 2020[54]	n=95	Nursing Assistants	Cross-sectional survey	05/04 - 05/29	United States of America	1.05%	High
Healthcare Support Occupations (31-0000)	Brzostek et al., 2020[151]	n=570	Nursing Assistants	Cross-sectional survey	04/17 - 05/07	United States of America	39.5%	Moderate
Healthcare Support Occupations (31-0000)	Brzostek et al., 2020[151]	n=263	Nursing Assistants	Cross-sectional survey	04/17 - 05/07	United States of America	45.6%	Moderate
Healthcare Support Occupations (31-0000)	Calcagno et al., 2020[124]	n=476	Nursing Assistants	Cross-sectional survey	04/17 - 05/20	Italy	9.24%	Moderate
Healthcare Support Occupations (31-0000)	Costa et al., 2020[128]	n=553	Nursing Assistants	Cross-sectional survey	05/14 - 05/28	Brazil	10.5%	Moderate
Healthcare Support Occupations (31-0000)	Galan et al., 2020[20]	n=472	Nursing Assistants	Cross-sectional survey	04/14 - 04/27	Spain	33.26%	High
Healthcare Support Occupations (31-0000)	Garcia et al., 2020[173]	n=2424	Nursing Assistants	Cross-sectional survey	05/01 - 05/30	Spain	22.4%	High
Healthcare Support Occupations (31-0000)	Garcia et al., 2020[174]	n=2424	Nursing Assistants	Cross-sectional survey	05/01 - 05/30	Spain	22.4%	High
Healthcare Support	Hanrath et al., 2020[32]	n=1434	Nursing Assistants	Cross-sectional survey	05/29 - 07/06	The United Kingdom	11.44%	High

For peer review only

Occupations (31-0000)								
Healthcare Support Occupations (31-0000)	Iversen et al., 2020[8]	n=501	Nursing Assistants	Cross-sectional survey	04/15 - 04/22	Denmark	1.2%	Low
Healthcare Support Occupations (31-0000)	Khan et al., 2020[127]	n=624	Nursing Assistants	Cross-sectional survey	06/15 - 06/29	India	2.4% (1.5-4%)	Moderate
Healthcare Support Occupations (31-0000)	Mughal et al., 2020[175]	n=121	Nursing Assistants	Cross-sectional survey	05/14 - 05/19	United States of America	0.83%	High
Healthcare Support Occupations (31-0000)	Rao et al., 2020[176]	n=1000	Nursing Assistants	Cross-sectional survey	05/23 - 06/06	India	1%	Unclear
Healthcare Support Occupations (31-0000)	Rudberg et al., 2020[147]	n=428	Nursing Assistants	Cross-sectional survey	04/14 - 05/08	Sweden	25.5%	Moderate
Healthcare Support Occupations (31-0000)	Siddiqui et al., 2020[2]	n=28	Nursing Assistants	Prospective cohort	04/15 - 08/15	India	10.7%	High
Healthcare Support Occupations (31-0000)	Yogo et al., 2020[36]	n=154	Nursing Assistants	Cross-sectional survey	05/20 - 06/08	United States of America	3.24%	High
Healthcare Support Occupations (31-0000)	Brousseau et al., 2020[134]	n=201	Orderlies	Cross-sectional survey	07/06 - 09/24	Canada	17.9%	High
Healthcare Support	Kassem et al., 2020[72]	n=9	Orderlies	Cross-sectional survey	06/01 - 06/14	Egypt	0%	High

For peer review only

Occupations (31-0000)								
Healthcare Support Occupations (31-0000)	Kassem et al., 2020[72]	n=9	Orderlies	Cross-sectional survey	06/01 - 06/14	Egypt	33.33%	High
Healthcare Support Occupations (31-0000)	Kassem et al., 2020[72]	n=9	Orderlies	Cross-sectional survey	06/01 - 06/14	Egypt	11.11%	High
Healthcare Support Occupations (31-0000)	Kassem et al., 2020[72]	n=9	Orderlies	Cross-sectional survey	06/01 - 06/14	Egypt	22.22%	High
Healthcare Support Occupations (31-0000)	Hanrath et al., 2020[32]	n=122	Orderlies	Cross-sectional survey	05/29 - 07/06	The United Kingdom	9.02%	High
Healthcare Support Occupations (31-0000)	Lumley et al., 2020[9]	n=377	Orderlies	Prospective cohort	04/23 - 11/30	The United Kingdom	15.38%	Moderate
Healthcare Support Occupations (31-0000)	Rosser et al., 2020[33]	n=3959	Medical Assistants	Cross-sectional survey	04/20 - 05/20	United States of America	1.39%	High
Healthcare Support Occupations (31-0000)	Yogo et al., 2020[36]	n=106	Phlebotomists	Cross-sectional survey	05/20 - 06/08	United States of America	0%	High
Healthcare Support Occupations (31-0000)	Cavlek et al., 2020[56]	n=300	Healthcare Support Workers, All Other	Cross-sectional survey	04/25 - 05/24	Croatia	0.67%	High
Healthcare Support	Erber et al., 2020[31]	n=383	Healthcare Support Workers, All Other	Cross-sectional survey	04/14 - 05/29	Germany	2.34%	High

For peer review only

Occupations (31-0000)								
Healthcare Support Occupations (31-0000)	Khan et al., 2020[127]	n=141	Healthcare Support Workers, All Other	Cross-sectional survey	06/15 - 06/29	India	0%	Moderate
Protective Service Occupations (33-0000)	Shukla et al., 2020[177]	n=1713	Protective Service Occupations	Cross-sectional survey	04/24 - 05/21	United States of America	1.46%	Moderate
Protective Service Occupations (33-0000)	Martinez et al., 2020[121]	n=18	First-Line Supervisors of Fire Fighting and Prevention Workers	Cross-sectional survey	04/16 - 04/17	United States of America	0%	High
Protective Service Occupations (33-0000)	Martinez et al., 2020[121]	n=47	First-Line Supervisors of Fire Fighting and Prevention Workers	Cross-sectional survey	04/16 - 04/17	United States of America	14.89%	High
Protective Service Occupations (33-0000)	Martinez et al., 2020[121]	n=13	First-Line Supervisors of Fire Fighting and Prevention Workers	Cross-sectional survey	04/16 - 04/17	United States of America	7.69%	High
Protective Service Occupations (33-0000)	Akinbami et al., 2020[46]	n=330	Firefighters	Cross-sectional survey	05/18 - 06/13	United States of America	6.7% (4.2-9.9%)	Moderate
Protective Service Occupations (33-0000)	Gray et al., 2020[178]	n=132	Firefighters	Cross-sectional survey	05/01 - 05/31	United States of America	14%	High
Protective Service Occupations (33-0000)	Reuben et al., 2020[168]	n=62	Firefighters	Cross-sectional survey	05/28 - 07/15	United States of America	4.84%	High
Protective Service Occupations (33-0000)	Sabourin et al., 2020[35]	n=42	Firefighters	Cross-sectional survey	07/15 - 08/15	United States of America	2.38%	High
Protective Service Occupations (33-0000)	Tarabichi et al., 2020[170]	n=185	Firefighters	Cross-sectional survey	04/20 - 05/19	United States of America	5.41%	High

For peer review only

Protective Service Occupations (33-0000)	Martinez et al., 2020[121]	n=7	Fire Inspectors and Investigators	Cross-sectional survey	04/16 - 04/17	United States of America	14.29%	High
Protective Service Occupations (33-0000)	Akinbami et al., 2020[46]	n=785	Police and Sheriff's Patrol Officers	Cross-sectional survey	05/18 - 06/13	United States of America	4% (2.7-5.6%)	Moderate
Protective Service Occupations (33-0000)	Chughtai et al., 2020[179]	n=154	Police and Sheriff's Patrol Officers	Cross-sectional survey	05/20 - 05/30	Pakistan	15.6%	High
Protective Service Occupations (33-0000)	Gudo et al., 2020[65]	n=564	Police and Sheriff's Patrol Officers	Cross-sectional survey	06/17 - 06/30	Mozambique	6% (4-8%)	High
Protective Service Occupations (33-0000)	Gujski et al., 2020[180]	n=4026	Police and Sheriff's Patrol Officers	Cross-sectional survey	06/22 - 07/08	Poland	4.2%	Moderate
Protective Service Occupations (33-0000)	Halatoko et al., 2020[41]	n=196	Police and Sheriff's Patrol Officers	Cross-sectional survey	04/23 - 05/08	Togo	0%	High
Protective Service Occupations (33-0000)	Langa et al., 2020[181]	n=471	Police and Sheriff's Patrol Officers	Cross-sectional survey	09/28 - 10/09	Mozambique	1.5%	High
Protective Service Occupations (33-0000)	Macicame et al., 2020[182]	n=456	Police and Sheriff's Patrol Officers	Cross-sectional survey	09/14 - 09/30	Mozambique	4.39%	High
Protective Service Occupations (33-0000)	Mahomed et al., 2020[81]	n=554	Police and Sheriff's Patrol Officers	Cross-sectional survey	08/31 - 10/12	Mozambique	2.9%	High
Protective Service Occupations (33-0000)	Reuben et al., 2020[168]	n=220	Police and Sheriff's Patrol Officers	Cross-sectional survey	05/28 - 07/15	United States of America	3.64%	High
Protective Service Occupations (33-0000)	Sabourin et al., 2020[35]	n=125	Police and Sheriff's Patrol Officers	Cross-sectional survey	07/15 - 08/15	United States of America	4%	High

For peer review only

Protective Service Occupations (33-0000)	Shukla et al., 2020[177]	n=1643	Police and Sheriff's Patrol Officers	Cross-sectional survey	04/24 - 05/21	United States of America	1.52%	Moderate
Protective Service Occupations (33-0000)	Siddiqui et al., 2020[2]	n=27	Police and Sheriff's Patrol Officers	Prospective cohort	04/15 - 08/15	India	7.4%	High
Protective Service Occupations (33-0000)	Viegas et al., 2020[110]	n=559	Police and Sheriff's Patrol Officers	Cross-sectional survey	08/03 - 08/21	Mozambique	3.94%	High
Protective Service Occupations (33-0000)	Denyer et al., 2020[60]	n=38216	Security Guards	Cross-sectional survey	05/12 - 05/18	Japan	0.23%	Unclear
Protective Service Occupations (33-0000)	Mahumane et al., 2020[82]	n=407	Security Guards	Cross-sectional survey	11/02 - 11/17	Mozambique	4.9%	High
Protective Service Occupations (33-0000)	Siddiqui et al., 2020[2]	n=9	Security Guards	Prospective cohort	04/15 - 08/15	India	0%	High
Protective Service Occupations (33-0000)	Silva et al., 2020[34]	n=32	Security Guards	Cross-sectional survey	06/05 - 07/31	Brazil	34%	High
Protective Service Occupations (33-0000)	Thani et al., 2020[183]	n=61	Security Guards	Cross-sectional survey	07/26 - 09/09	Qatar	60.1%	Moderate
Food Preparation and Serving Related Occupations (35-0000)	Thani et al., 2020[183]	n=93	Food Preparation and Serving Related Occupations	Cross-sectional survey	07/26 - 09/09	Qatar	29.2%	Moderate
Food Preparation and Serving Related Occupations (35-0000)	Siddiqui et al., 2020[2]	n=8	Cooks, All Other	Prospective cohort	04/15 - 08/15	India	37.5%	High
Food Preparation and Serving	Brunner et al., 2020[54]	n=8	Food Preparation Workers	Cross-sectional survey	05/04 - 05/29	United States of America	0%	High

For peer review only

Related Occupations (35-0000)								
Healthcare Support Occupations (31-0000)	Rosser et al., 2020[33]	n=335	Healthcare Support Occupations	Cross-sectional survey	04/20 - 05/20	United States of America	3.58%	High
Food Preparation and Serving Related Occupations (35-0000)	Biggs et al., 2020[3]	n=24	Food Servers, Nonrestaurant	Cross-sectional survey	04/28 - 05/03	United States of America	4.17%	Moderate
Food Preparation and Serving Related Occupations (35-0000)	Leidner et al., 2020[22]	n=113	Food Servers, Nonrestaurant	Cross sectional study with prospective cohort follow up of a subset of the sample	04/08 - 05/22	United States of America	1.77%	High
Food Preparation and Serving Related Occupations (35-0000)	Hanrath et al., 2020[32]	n=340	Other Food Preparation and Serving Related Workers	Cross-sectional survey	05/29 - 07/06	The United Kingdom	8.53%	High
Building and Grounds Cleaning and Maintenance Occupations (37-0000)	Martin et al., 2020[23]	n=528	Building and Grounds Cleaning and Maintenance Occupations	Cross-sectional survey	05/29 - 07/13	The United Kingdom	8.14%	Moderate
Building and Grounds Cleaning and Maintenance Occupations (37-0000)	Brousseau et al., 2020[134]	n=102	Building Cleaning and Pest Control Workers	Cross-sectional survey	07/06 - 09/24	Canada	10.8%	High
Building and Grounds Cleaning and Maintenance	Chau et al., 2020[126]	n=42	Building Cleaning and Pest Control Workers	Cross-sectional survey	08/23 - 08/30	Viet Nam	0%	High

For peer review only

Occupations (37-0000)								
Building and Grounds Cleaning and Maintenance Occupations (37-0000)	Finkenzeller et al., 2020[158]	n=57	Building Cleaning and Pest Control Workers	Prospective cohort	06/29 - 07/29	Germany	19.3%	Moderate
Building and Grounds Cleaning and Maintenance Occupations (37-0000)	Chau et al., 2020[126]	n=6	Janitors and Cleaners, Except Maids and Housekeeping Cleaners	Cross-sectional survey	08/23 - 08/30	Viet Nam	0%	High
Building and Grounds Cleaning and Maintenance Occupations (37-0000)	Epstude et al., 2020[184]	n=45	Janitors and Cleaners, Except Maids and Housekeeping Cleaners	Cross-sectional survey	06/15 - 06/30	Germany	0%	High
Building and Grounds Cleaning and Maintenance Occupations (37-0000)	Thani et al., 2020[183]	n=105	Janitors and Cleaners, Except Maids and Housekeeping Cleaners	Cross-sectional survey	07/26 - 09/09	Qatar	54.5%	Moderate
Building and Grounds Cleaning and Maintenance Occupations (37-0000)	Brunner et al., 2020[54]	n=23	Maids and Housekeeping Cleaners	Cross-sectional survey	05/04 - 05/29	United States of America	0%	High
Building and Grounds Cleaning and Maintenance Occupations (37-0000)	Goenka et al., 2020[25]	n=226	Maids and Housekeeping Cleaners	Cross-sectional survey	07/12 - 08/23	India	26.11%	Moderate
Building and Grounds Cleaning and Maintenance Occupations (37-0000)	Goenka et al., 2020[26]	n=10	Maids and Housekeeping Cleaners	Cross-sectional survey	08/01 - 08/31	India	10%	High

For peer review only

Building and Grounds Cleaning and Maintenance Occupations (37-0000)	Hanrath et al., 2020[32]	n=515	Maids and Housekeeping Cleaners	Cross-sectional survey	05/29 - 07/06	The United Kingdom	13.2%	High
Building and Grounds Cleaning and Maintenance Occupations (37-0000)	Khan et al., 2020[127]	n=276	Maids and Housekeeping Cleaners	Cross-sectional survey	06/15 - 06/29	India	3.3% (1.7-6.2%)	Moderate
Building and Grounds Cleaning and Maintenance Occupations (37-0000)	Leidner et al., 2020[22]	n=137	Maids and Housekeeping Cleaners	Cross sectional study with prospective cohort follow up of a subset of the sample	04/08 - 05/22	United States of America	8.03%	High
Building and Grounds Cleaning and Maintenance Occupations (37-0000)	Moscola et al., 2020[89]	n=7314	Maids and Housekeeping Cleaners	Cross-sectional survey	04/20 - 06/23	United States of America	20.9%	High
Building and Grounds Cleaning and Maintenance Occupations (37-0000)	Shakiba et al., 2020[10]	n=159	Maids and Housekeeping Cleaners	Cross-sectional survey	04/11 - 04/19	Iran (Islamic Republic of)	25% (13.6-37.5%)	Moderate
Building and Grounds Cleaning and Maintenance Occupations (37-0000)	Shields et al., 2020[97]	n=29	Maids and Housekeeping Cleaners	Cross-sectional survey	04/24 - 04/25	The United Kingdom	34.5%	High
Building and Grounds Cleaning and Maintenance Occupations (37-0000)	Siddiqui et al., 2020[2]	n=46	Maids and Housekeeping Cleaners	Prospective cohort	04/15 - 08/15	India	21.7%	High

For peer review only

Personal Care and Service Occupations (39-0000)	Biggs et al., 2020[3]	n=10	Hairdressers, Hairstylists, and Cosmetologists	Cross-sectional survey	04/28 - 05/03	United States of America	10%	Moderate
Personal Care and Service Occupations (39-0000)	Biggs et al., 2020[3]	n=48	Childcare Workers	Cross-sectional survey	04/28 - 05/03	United States of America	0%	Moderate
Personal Care and Service Occupations (39-0000)	Chen et al., 2020[135]	n=11	Personal Care Aides	Cross-sectional survey	02/19 - 02/19	China	9.09%	High
Personal Care and Service Occupations (39-0000)	Galan et al., 2020[20]	n=337	Personal Care Aides	Cross-sectional survey	04/14 - 04/27	Spain	27.89%	High
Personal Care and Service Occupations (39-0000)	Galan et al., 2020[20]	n=168	Personal Care Aides	Cross-sectional survey	04/14 - 04/27	Spain	27.38%	High
Personal Care and Service Occupations (39-0000)	Godbout et al., 2020[138]	n=86	Personal Care Aides	Cross-sectional survey	07/27 - 10/02	United States of America	2.32%	High
Personal Care and Service Occupations (39-0000)	Hassan et al., 2020[185]	n=403	Personal Care Aides	Cross-sectional survey	05/11 - 06/17	Sweden	20.1%	High
Personal Care and Service Occupations (39-0000)	Kumar et al., 2020[142]	n=292	Personal Care Aides	Cross-sectional survey	06/01 - 06/30	India	18.5% (14.5-23.3%)	High
Personal Care and Service Occupations (39-0000)	Ladhani et al., 2020[186]	n=208	Personal Care Aides	Prospective cohort	04/10 - 04/13	The United Kingdom	75% (68.7-80.4%)	High

For peer review only

Personal Care and Service Occupations (39-0000)	Lindahl et al., 2020[187]	n=1005	Personal Care Aides	Cross-sectional survey	04/01 - 04/20	Sweden	22.9% (20.4-25.7%)	High
Personal Care and Service Occupations (39-0000)	Regan et al., 2020[188]	n=305	Personal Care Aides	Cross-sectional survey	04/15 - 05/06	United States of America	23.6%	Unclear
Personal Care and Service Occupations (39-0000)	Siddiqui et al., 2020[2]	n=5	Personal Care Aides	Prospective cohort	04/15 - 08/15	India	40%	High
Personal Care and Service Occupations (39-0000)	Venugopal et al., 2020[150]	n=72	Personal Care Aides	Cross-sectional survey	03/01 - 05/01	United States of America	28%	Moderate
Personal Care and Service Occupations (39-0000)	Viegas et al., 2020[110]	n=85	Personal Care Aides	Cross-sectional survey	08/03 - 08/21	Mozambique	1.18%	High
Sales and Related Occupations (41-0000)	Arnaldo et al., 2020[13]	n=928	Sales and Related Occupations	Cross-sectional survey	07/06 - 07/13	Mozambique	6.5%	High
Sales and Related Occupations (41-0000)	Arnaldo et al., 2020[48]	n=1123	Sales and Related Occupations	Cross-sectional survey	08/10 - 08/21	Mozambique	1.6%	High
Sales and Related Occupations (41-0000)	Langa et al., 2020[181]	n=871	Sales and Related Occupations	Cross-sectional survey	09/28 - 10/09	Mozambique	0.2%	High
Sales and Related Occupations (41-0000)	Mabunda et al., 2020[15]	n=1585	Sales and Related Occupations	Cross-sectional survey	09/21 - 10/02	Mozambique	8.3%	High
Sales and Related Occupations (41-0000)	Macicame et al., 2020[182]	n=1288	Sales and Related Occupations	Cross-sectional survey	09/14 - 09/30	Mozambique	4.97%	High

For peer review only

Sales and Related Occupations (41-0000)	Mahomed et al., 2020[81]	n=1556	Sales and Related Occupations	Cross-sectional survey	08/31 - 10/12	Mozambique	0.8%	High
Sales and Related Occupations (41-0000)	Mahumane et al., 2020[82]	n=643	Sales and Related Occupations	Cross-sectional survey	11/02 - 11/17	Mozambique	1.9%	High
Sales and Related Occupations (41-0000)	Arnaldo et al., 2020[14]	n=472	Sales and Related Occupations	Cross-sectional survey	11/16 - 11/21	Mozambique	6.8%	High
Sales and Related Occupations (41-0000)	Arnaldo et al., 2020[14]	n=460	Sales and Related Occupations	Cross-sectional survey	11/02 - 11/12	Mozambique	5.9%	High
Sales and Related Occupations (41-0000)	Mahomed et al., 2020[16]	n=517	Sales and Related Occupations	Cross-sectional survey	11/26 - 12/03	Mozambique	8.9%	High
Sales and Related Occupations (41-0000)	Mahomed et al., 2020[16]	n=1001	Sales and Related Occupations	Cross-sectional survey	11/07 - 11/21	Mozambique	4.5%	High
Sales and Related Occupations (41-0000)	Biggs et al., 2020[3]	n=19	Retail Sales Workers	Cross-sectional survey	04/28 - 05/03	United States of America	0%	Moderate
Sales and Related Occupations (41-0000)	Poustchi et al., 2020[28]	n=753	Cashiers	Cross-sectional survey	04/17 - 06/02	Iran (Islamic Republic of)	16.1% (12.9-19.2%)	Moderate
Sales and Related Occupations (41-0000)	Alali et al., 2020[189]	n=525	Cashiers	Cross-sectional survey	05/23 - 06/26	Kuwait	38.1% (34-42.3%)	High
Sales and Related Occupations (41-0000)	Denyer et al., 2020[60]	n=19075	Retail Salespersons	Cross-sectional survey	05/12 - 05/18	Japan	0.04%	Unclear
Sales and Related Occupations (41-0000)	Kern et al., 2020[73]	n=300	Retail Salespersons	Cross-sectional survey	04/09 - 04/16	Germany	0.33% (0.01-1.84%)	High

For peer review only

Sales and Related Occupations (41-0000)	Khan et al., 2020[45]	n=132	Retail Salespersons	Cross-sectional survey	07/01 - 07/15	India	5.3% (2.5-10.7%)	Moderate
Sales and Related Occupations (41-0000)	Thani et al., 2020[183]	n=171	Retail Salespersons	Cross-sectional survey	07/26 - 09/09	Qatar	40.3%	Moderate
Sales and Related Occupations (41-0000)	Siddiqui et al., 2020[2]	n=4	Sales Representatives, Wholesale and Manufacturing, Except Technical and Scientific Products	Prospective cohort	04/15 - 08/15	India	25%	High
Sales and Related Occupations (41-0000)	Biggs et al., 2020[3]	n=34	Real Estate Sales Agents	Cross-sectional survey	04/28 - 05/03	United States of America	0%	Moderate
Sales and Related Occupations (41-0000)	Gudo et al., 2020[65]	n=1493	Door-to-Door Sales Workers, News and Street Vendors, and Related Workers	Cross-sectional survey	06/17 - 06/30	Mozambique	10% (8-11%)	High
Sales and Related Occupations (41-0000)	Viegas et al., 2020[110]	n=1246	Door-to-Door Sales Workers, News and Street Vendors, and Related Workers	Cross-sectional survey	08/03 - 08/21	Mozambique	5.22%	High
Sales and Related Occupations (41-0000)	Shakiba et al., 2020[10]	n=46	Sales and Related Workers, All Other	Cross-sectional survey	04/11 - 04/19	Iran (Islamic Republic of)	8.7% (0.8-20%)	Moderate
Office and Administrative Support Occupations (43-0000)	Calcagno et al., 2020[124]	n=539	Office and Administrative Support Occupations	Cross-sectional survey	04/17 - 05/20	Italy	3.34%	Moderate
Office and Administrative Support Occupations (43-0000)	Costa et al., 2020[128]	n=120	Office and Administrative Support Occupations	Cross-sectional survey	05/14 - 05/28	Brazil	14.2%	Moderate

For peer review only

Office and Administrative Support Occupations (43-0000)	Rosser et al., 2020[33]	n=972	Office and Administrative Support Occupations	Cross-sectional survey	04/20 - 05/20	United States of America	1.34%	High
Office and Administrative Support Occupations (43-0000)	Tsitsilonis et al., 2020[12]	n=504	Office and Administrative Support Occupations	Cross-sectional survey	06/15 - 07/15	Greece	0.48% (0-2.37%)	Moderate
Office and Administrative Support Occupations (43-0000)	Khan et al., 2020[45]	n=37	Hotel, Motel, and Resort Desk Clerks	Cross-sectional survey	07/01 - 07/15	India	10.8% (4.1-25.5%)	Moderate
Office and Administrative Support Occupations (43-0000)	Brunner et al., 2020[54]	n=26	Receptionists and Information Clerks	Cross-sectional survey	05/04 - 05/29	United States of America	0%	High
Office and Administrative Support Occupations (43-0000)	Favara et al., 2020[136]	n=10	Receptionists and Information Clerks	Prospective cohort	06/01 - 06/07	The United Kingdom	0%	High
Office and Administrative Support Occupations (43-0000)	Moscola et al., 2020[89]	n=9645	Receptionists and Information Clerks	Cross-sectional survey	04/20 - 06/23	United States of America	12.6%	High
Office and Administrative Support Occupations (43-0000)	Biggs et al., 2020[3]	n=11	Shipping, Receiving, and Traffic Clerks	Cross-sectional survey	04/28 - 05/03	United States of America	18.18%	Moderate
Office and Administrative	Silva et al., 2020[34]	n=82	Stock Clerks and Order Fillers	Cross-sectional survey	06/05 - 07/31	Brazil	4.88%	High

For peer review only

Support Occupations (43-0000)								
Office and Administrative Support Occupations (43-0000)	Khan et al., 2020[45]	n=186	Secretaries and Administrative Assistants	Cross-sectional survey	07/01 - 07/15	India	3.8% (1.8-7.7%)	Moderate
Office and Administrative Support Occupations (43-0000)	Alemu et al., 2020[6]	n=48	Executive Secretaries and Executive Administrative Assistants	Cross-sectional survey	04/23 - 04/28	Ethiopia	2.1%	Moderate
Office and Administrative Support Occupations (43-0000)	Barallat et al., 2020[50]	n=1181	Executive Secretaries and Executive Administrative Assistants	Cross-sectional survey	05/04 - 05/22	Spain	6.52%	High
Office and Administrative Support Occupations (43-0000)	Lumley et al., 2020[9]	n=1557	Executive Secretaries and Executive Administrative Assistants	Prospective cohort	04/23 - 11/30	The United Kingdom	6.74%	Moderate
Office and Administrative Support Occupations (43-0000)	Reuben et al., 2020[168]	n=18	Executive Secretaries and Executive Administrative Assistants	Cross-sectional survey	05/28 - 07/15	United States of America	0%	High
Office and Administrative Support Occupations (43-0000)	Akinbami et al., 2020[46]	n=964	Medical Secretaries	Cross-sectional survey	05/18 - 06/13	United States of America	8% (6.4-9.9%)	Moderate
Office and Administrative Support	Alharbi et al., 2020[125]	n=8	Medical Secretaries	Cross-sectional survey	04/18 - 06/17	Saudi Arabia	25%	High

For peer review only

Occupations (43-0000)								
Office and Administrative Support Occupations (43-0000)	Dimcheff et al., 2020[157]	n=357	Medical Secretaries	Cross-sectional survey	06/08 - 07/08	United States of America	4.2%	Moderate
Office and Administrative Support Occupations (43-0000)	Erber et al., 2020[31]	n=557	Medical Secretaries	Cross-sectional survey	04/14 - 05/29	Germany	3.78%	High
Office and Administrative Support Occupations (43-0000)	Finkenzeller et al., 2020[158]	n=240	Medical Secretaries	Prospective cohort	06/29 - 07/29	Germany	7.1%	Moderate
Office and Administrative Support Occupations (43-0000)	Goenka et al., 2020[25]	n=75	Medical Secretaries	Cross-sectional survey	07/12 - 08/23	India	8%	Moderate
Office and Administrative Support Occupations (43-0000)	Goenka et al., 2020[25]	n=75	Medical Secretaries	Cross-sectional survey	07/12 - 08/23	India	8%	Moderate
Office and Administrative Support Occupations (43-0000)	Iversen et al., 2020[8]	n=2631	Medical Secretaries	Cross-sectional survey	04/15 - 04/22	Denmark	2.7%	Low
Office and Administrative Support Occupations (43-0000)	Leidner et al., 2020[22]	n=793	Medical Secretaries	Cross sectional study with prospective cohort follow up of a	04/08 - 05/22	United States of America	3.15%	High

For peer review only

				subset of the sample				
Office and Administrative Support Occupations (43-0000)	Mesnil et al., 2020[143]	n=184	Medical Secretaries	Cross-sectional survey	06/08 - 06/22	France	14.13%	High
Office and Administrative Support Occupations (43-0000)	Nishida et al., 2020[90]	n=98	Medical Secretaries	Cross-sectional survey	06/12 - 06/19	Japan	1% (0.18-5.6%)	Moderate
Office and Administrative Support Occupations (43-0000)	Noor et al., 2020[130]	n=91	Medical Secretaries	Cross-sectional survey	07/13 - 07/15	Pakistan	43.96%	Moderate
Office and Administrative Support Occupations (43-0000)	Thani et al., 2020[183]	n=82	Medical Secretaries	Cross-sectional survey	07/26 - 09/09	Qatar	31.6%	Moderate
Office and Administrative Support Occupations (43-0000)	Zhou et al., 2020[166]	n=505	Medical Secretaries	Cross-sectional survey	03/16 - 03/25	China	1.39%	Moderate
Office and Administrative Support Occupations (43-0000)	Chau et al., 2020[126]	n=20	Data Entry Keyers	Cross-sectional survey	08/23 - 08/30	Viet Nam	0%	High
Office and Administrative Support Occupations (43-0000)	Jones et al., 2020[29]	n=1233	Office Clerks, General	Cross-sectional survey	01/15 - 06/15	The United Kingdom	6.1%	High

For peer review only

Office and Administrative Support Occupations (43-0000)	Rosser et al., 2020[33]	n=218	Office Clerks, General	Cross-sectional survey	04/20 - 05/20	United States of America	0%	High
Office and Administrative Support Occupations (43-0000)	Satpati et al., 2020[27]	n=47	Office Clerks, General	Cross-sectional survey	07/26 - 08/08	India	4.26%	Moderate
Office and Administrative Support Occupations (43-0000)	Baracco et al., 2020[24]	n=194	Office and Administrative Support Workers, All Other	Cross-sectional survey	04/23 - 05/05	Italy	14.4%	High
Office and Administrative Support Occupations (43-0000)	Brzostek et al., 2020[151]	n=286	Office and Administrative Support Workers, All Other	Cross-sectional survey	04/17 - 05/07	United States of America	45.5%	Moderate
Office and Administrative Support Occupations (43-0000)	Kassem et al., 2020[72]	n=7	Office and Administrative Support Workers, All Other	Cross-sectional survey	06/01 - 06/14	Egypt	14.28%	High
Office and Administrative Support Occupations (43-0000)	Kassem et al., 2020[72]	n=7	Office and Administrative Support Workers, All Other	Cross-sectional survey	06/01 - 06/14	Egypt	0%	High
Office and Administrative Support Occupations (43-0000)	Kassem et al., 2020[72]	n=7	Office and Administrative Support Workers, All Other	Cross-sectional survey	06/01 - 06/14	Egypt	0%	High

For peer review only

Office and Administrative Support Occupations (43-0000)	Kassem et al., 2020[72]	n=7	Office and Administrative Support Workers, All Other	Cross-sectional survey	06/01 - 06/14	Egypt	14.28%	High
Farming, Fishing, and Forestry Occupations (45-0000)	Satpati et al., 2020[27]	n=53	Agricultural Workers	Cross-sectional survey	07/26 - 08/08	India	0%	Moderate
Farming, Fishing, and Forestry Occupations (45-0000)	Addetia et al., 2020[190]	n=120	Fishers and Related Fishing Workers	Retrospective cohort	05/01 - 05/31	United States of America	5%	High
Farming, Fishing, and Forestry Occupations (45-0000)	Arnaldo et al., 2020[13]	n=80	Fishers and Related Fishing Workers	Cross-sectional survey	07/06 - 07/13	Mozambique	5%	High
Construction and Extraction Occupations (47-0000)	Biggs et al., 2020[3]	n=42	Construction Trades Workers	Cross-sectional survey	04/28 - 05/03	United States of America	0%	Moderate
Installation, Maintenance, and Repair Occupations (49-0000)	Blairon et al., 2020[52]	n=134	Other Installation, Maintenance, and Repair Occupations	Cross-sectional survey	05/25 - 06/19	Belgium	16.4%	High
Production Occupations (51-0000)	Picon et al., 2020[191]	n=40	Butchers and Other Meat, Poultry, and Fish Processing Workers	Cross-sectional survey	06/13 - 06/17	Brazil	15%	Moderate
Production Occupations (51-0000)	Picon et al., 2020[191]	n=1087	Miscellaneous Food Processing Workers	Cross-sectional survey	06/13 - 06/17	Brazil	1.47%	Moderate
Production Occupations (51-0000)	Bontadi et al., 2020[192]	n=1267	Production Workers, All Other	Cross-sectional survey	04/11 - 04/29	Italy	1.58%	High

For peer review only

Production Occupations (51-0000)	Xu et al., 2020[193]	n=442	Production Workers, All Other	Cross-sectional survey	03/09 - 04/10	China	1.4% (0.6-2.9%)	High
Transportation and Material Moving Occupations (53-0000)	Arnaldo et al., 2020[13]	n=248	Transportation and Material Moving Occupations	Cross-sectional survey	07/06 - 07/13	Mozambique	4.8%	High
Transportation and Material Moving Occupations (53-0000)	Arnaldo et al., 2020[48]	n=367	Transportation and Material Moving Occupations	Cross-sectional survey	08/10 - 08/21	Mozambique	7.4%	High
Transportation and Material Moving Occupations (53-0000)	Arnaldo et al., 2020[14]	n=112	Transportation and Material Moving Occupations	Cross-sectional survey	11/16 - 11/21	Mozambique	16.1%	High
Transportation and Material Moving Occupations (53-0000)	Biggs et al., 2020[3]	n=14	Transportation and Material Moving Occupations	Cross-sectional survey	04/28 - 05/03	United States of America	0%	Moderate
Transportation and Material Moving Occupations (53-0000)	Gudo et al., 2020[65]	n=554	Transportation and Material Moving Occupations	Cross-sectional survey	06/17 - 06/30	Mozambique	3% (1-4%)	High
Transportation and Material Moving Occupations (53-0000)	Langa et al., 2020[181]	n=230	Transportation and Material Moving Occupations	Cross-sectional survey	09/28 - 10/09	Mozambique	0.4%	High
Transportation and Material Moving Occupations (53-0000)	Mabunda et al., 2020[15]	n=473	Transportation and Material Moving Occupations	Cross-sectional survey	09/21 - 10/02	Mozambique	8.7%	High
Transportation and Material Moving Occupations (53-0000)	Macicame et al., 2020[182]	n=282	Transportation and Material Moving Occupations	Cross-sectional survey	09/14 - 09/30	Mozambique	3.19%	High

For peer review only

Transportation and Material Moving Occupations (53-0000)	Mahomed et al., 2020[81]	n=334	Transportation and Material Moving Occupations	Cross-sectional survey	08/31 - 10/12	Mozambique	1.5%	High
Transportation and Material Moving Occupations (53-0000)	Mahumane et al., 2020[82]	n=287	Transportation and Material Moving Occupations	Cross-sectional survey	11/02 - 11/17	Mozambique	1%	High
Transportation and Material Moving Occupations (53-0000)	Thani et al., 2020[183]	n=435	Transportation and Material Moving Occupations	Cross-sectional survey	07/26 - 09/09	Qatar	53.4%	Moderate
Transportation and Material Moving Occupations (53-0000)	Halatoko et al., 2020[41]	n=212	Air Transportation Workers	Cross-sectional survey	04/23 - 05/08	Togo	0.9%	High
Transportation and Material Moving Occupations (53-0000)	Viegas et al., 2020[110]	n=623	Air Transportation Workers	Cross-sectional survey	08/03 - 08/21	Mozambique	2.25%	High
Transportation and Material Moving Occupations (53-0000)	Viegas et al., 2020[110]	n=362	Air Transportation Workers	Cross-sectional survey	08/03 - 08/21	Mozambique	3.31%	High
Transportation and Material Moving Occupations (53-0000)	Khan et al., 2020[127]	n=57	Ambulance Drivers and Attendants, Except Emergency Medical Technicians	Cross-sectional survey	06/15 - 06/29	India	3.5% (0.9-13.3%)	Moderate
Transportation and Material Moving Occupations (53-0000)	Martinez et al., 2020[121]	n=30	Heavy and Tractor-Trailer Truck Drivers	Cross-sectional survey	04/16 - 04/17	United States of America	16.67%	High
Transportation and Material Moving Occupations (53-0000)	Siddiqui et al., 2020[2]	n=9	Heavy and Tractor-Trailer Truck Drivers	Prospective cohort	04/15 - 08/15	India	11.1%	High

For peer review only

Transportation and Material Moving Occupations (53-0000)	Halatoko et al., 2020[41]	n=122	Taxi Drivers and Chauffeurs	Cross-sectional survey	04/23 - 05/08	Togo	0.8%	High
Transportation and Material Moving Occupations (53-0000)	Poustchi et al., 2020[28]	n=718	Taxi Drivers and Chauffeurs	Cross-sectional survey	04/17 - 06/02	Iran (Islamic Republic of)	14.1% (11.4-16.9%)	Moderate
Transportation and Material Moving Occupations (53-0000)	Alemu et al., 2020[6]	n=8	Parking Lot Attendants	Cross-sectional survey	04/23 - 04/28	Ethiopia	12.5%	Moderate
Transportation and Material Moving Occupations (53-0000)	Alemu et al., 2020[6]	n=110	Laborers and Freight, Stock, and Material Movers, Hand	Cross-sectional survey	04/23 - 04/28	Ethiopia	10%	Moderate
Transportation and Material Moving Occupations (53-0000)	Khan et al., 2020[45]	n=97	Laborers and Freight, Stock, and Material Movers, Hand	Cross-sectional survey	07/01 - 07/15	India	2.1% (0.5-7.9%)	Moderate
Transportation and Material Moving Occupations (53-0000)	Satpati et al., 2020[27]	n=63	Laborers and Freight, Stock, and Material Movers, Hand	Cross-sectional survey	07/26 - 08/08	India	12.7%	Moderate
Not employed (mixed)*	Carrat et al., 2020[4]	n=6295	Unemployed	Prospective cohort	05/04 - 06/23	France	4.9% (4.1-5.6%)	Moderate
Not employed (mixed)*	Carrat et al., 2020[4]	n=1457	Unemployed	Prospective cohort	05/04 - 06/23	France	8.3% (6.4-10%)	Moderate
Not employed (mixed)*	Carrat et al., 2020[4]	n=306	Unemployed	Prospective cohort	05/04 - 06/23	France	7.2% (2.3-11.1%)	Moderate
Not employed (mixed)*	Carrat et al., 2020[4]	n=125	Unemployed	Prospective cohort	05/04 - 06/23	France	3.8% (0.5-6.3%)	Moderate
Not employed (mixed)*	Carrat et al., 2020[4]	n=402	Unemployed	Prospective cohort	05/04 - 06/23	France	7.8% (4.7-10.4%)	Moderate

For peer review only

Not employed (mixed)*	Chamie et al., 2020[194]	n=230	Unemployed	Cross-sectional survey	04/25 - 04/28	United States of America	4.3%	Moderate
Not employed (mixed)*	McLaughlin et al., 2020[195]	n=241	Unemployed	Cross-sectional survey	05/04 - 05/19	United States of America	19.3% (14.6-24.5%)	Moderate
Not employed (mixed)*	Merkely et al., 2020[1]	n=1095	Unemployed	Cross-sectional survey	05/01 - 05/16	Hungary	0.43% (0.16-0.84%)	Moderate
Not employed (mixed)*	Munoz et al., 2020[196]	n=905	Unemployed	Cross-sectional survey	07/15 - 07/16	Argentina	20%	Moderate
Not employed (mixed)*	Richard et al., 2020[5]	n=549	Unemployed	Cross-sectional survey	04/06 - 06/30	Switzerland	6%	Low
Not employed (mixed)*	Satpati et al., 2020[27]	n=47	Unemployed	Cross-sectional survey	07/26 - 08/08	India	2.13%	Moderate
Not employed (mixed)*	Ward et al., 2020[113]	n=59369	Unemployed	Cross-sectional survey	09/15 - 09/28	The United Kingdom	3.35%	Moderate

1. Merkely B, Szabó AJ, Kosztin A, et al. Novel coronavirus epidemic in the Hungarian population, a cross-sectional nationwide survey to support the exit policy in Hungary. *GeroScience*. 2020;42(4):1063-1074. doi:10.1007/s11357-020-00226-9

2. Siddiqui S, Naushin S, Pradhan S, et al. SARS-CoV-2 antibody seroprevalence and stability in a tertiary care hospital-setting. *medRxiv*. Published online September 2020. doi:10.1101/2020.09.02.20186486

3. Biggs HM, Harris JB, Breakwell L, et al. Estimated Community Seroprevalence of SARS-CoV-2 Antibodies Two Georgia Counties, April 28May 3, 2020. *MMWR Morbidity and Mortality Weekly Report*. 2020;69(29):965-970. doi:10.15585/mmwr.mm6929e2

4. Carrat F, Lamballerie X de, Rahib D, et al. Seroprevalence of SARS-CoV-2 among adults in three regions of France following the lockdown and associated risk factors: A multicohort study. *medRxiv*. Published online September 2020:2020.09.16.20195693. doi:10.1101/2020.09.16.20195693

5. Richard A, Wisniak A, Perez-Saez J, et al. Seroprevalence of anti-SARS-CoV-2 IgG antibodies, risk factors for infection and associated symptoms in Geneva, Switzerland: A population-based study. *medRxiv*. Published online December 2020. doi:10.1101/2020.12.16.20248180

6. Alemu BN, Addissie A, Mamo G, et al. *Sero-Prevalence of Anti-SARS-CoV-2 Antibodies in Addis Ababa, Ethiopia*. Microbiology; 2020. doi:10.1101/2020.10.13.337287

7. Brehm T, Schwinge D, Lampalzer S, et al. Seroprevalence of SARS-CoV-2 antibodies among hospital workers in a German tertiary care center: A sequential follow-up study. *International Journal of Hygiene and Environmental Health*. 2021;232:113671. doi:10.1016/j.ijheh.2020.113671

8. Iversen K, Bundgaard H, Hasselbalch RB, et al. Risk of COVID-19 in health-care workers in Denmark: An observational cohort study. *The Lancet Infectious diseases*. Published online August 2020. doi:10.1016/S1473-3099(20)30589-2

For peer review only

9. Lumley SF, O'Donnell D, Stoesser NE, et al. Antibody Status and Incidence of SARS-CoV-2 Infection in Health Care Workers. *New England Journal of Medicine*. Published online December 2020:NEJMoa2034545. doi:10.1056/NEJMoa2034545

10. Shakiba M, Nazemipour M, Salari A, et al. Seroprevalence of SARS-CoV-2 in Guilan Province, Iran, April 2020. *Emerging Infectious Disease journal*. 2021;27(2). doi:10.3201/eid2702.201960

11. Tilley K, Ayvazyan V, Martinez L, et al. A Cross-Sectional Study Examining the Seroprevalence of Severe Acute Respiratory Syndrome Coronavirus 2 Antibodies in a University Student Population. *Journal of Adolescent Health*. 2020;67(6):763-768. doi:10.1016/j.jadohealth.2020.09.001

12. Tsitsilonis OE, Paraskevis D, Lianidou E, et al. Seroprevalence of Antibodies against SARS-CoV-2 among the Personnel and Students of the National and Kapodistrian University of Athens, Greece: A Preliminary Report. *Life*. 2020;10(9):214. doi:10.3390/life10090214

13. Paulo Arnaldo. *Inquérito Sero-Epidemiológico de SARS-CoV-2 Na Cidade de Pemba (InCOVID 2020)*. República de Moçambique Ministério da Saúde; 2020.

14. Paulo Arnaldo. *Inquérito Sero-Epidemiológico de SARS-CoV-2 Nas Cidades de Xai-Xai E Chókwè (InCOVID 2020)*. República de Moçambique Ministério da Saúde; 2020.

15. Nedio Mabunda. *Inquérito Sero-Epidemiológico de SARS-CoV-2 Na Cidade de Beira (InCOVID 2020)*. República de Moçambique Ministério da Saúde; 2020.

16. Mussagy Mahomed. *Inquérito Sero-Epidemiológico de SARS-CoV-2 Na Cidade de Maxixe E Vila de Massinga (InCOVID 2020)*. República de Moçambique Ministério da Saúde; 2020.

17. Payne DC, Smith-Jeffcoat SE, Nowak G, et al. SARS-CoV-2 Infections and Serologic Responses from a Sample of U.S. Navy Service Members - USS Theodore Roosevelt, April 2020. *MMWR Morbidity and mortality weekly report*. 2020;69(23):714-721. doi:10.15585/mmwr.mm6923e4

18. COVID-19 Serology Tests Still Show Low Antibody Rate of 0.07%. *KBS World Radio*.

19. Favara DM, McAdam K, Cooke A, et al. SARS-CoV-2 antigen and antibody prevalence among UK staff working with cancer patients during the COVID-19 pandemic. *medRxiv*. Published online September 2020:2020.09.18.20197590. doi:10.1101/2020.09.18.20197590

20. Galán MI, Velasco M, Casas ML, et al. Hospital-Wide SARS-CoV-2 seroprevalence in health care workers in a Spanish teaching hospital. *Enfermedades Infecciosas y Microbiología Clínica*. Published online December 2020:S0213005X20304183. doi:10.1016/j.eimc.2020.11.015

21. Hunter BR, Dbeibo L, Weaver CS, et al. Seroprevalence of severe acute respiratory coronavirus virus 2 (SARS-CoV-2) antibodies among healthcare workers with differing levels of coronavirus disease 2019 (COVID-19) patient exposure. *Infection Control & Hospital Epidemiology*. Published online August 2020:1-2. doi:10.1017/ice.2020.390

22. Leidner R, Frary A, Cramer J, et al. Longitudinal SARS-CoV-2 serosurveillance of over ten thousand health care workers in the Providence Oregon cohort. *medRxiv*. Published online August 2020:2020.08.16.20176107. doi:10.1101/2020.08.16.20176107

23. Martin CA, Patel P, Goss C, et al. Demographic and occupational determinants of anti-SARS-CoV-2 IgG seropositivity in hospital staff. *Journal of Public Health*. 2020;(fdaa199). doi:10.1093/pubmed/fdaa199

For peer review only

24. Baracco A, Perotti G, Filippin A, et al. *SARS-CoV-2 Antibody Prevalence in Health Care Workers of Lodi Hospital, the COVID-19 Italian Epicentre*. Social Science Research Network; 2020.

25. Goenka M, Afzalpurkar S, Goenka U, et al. Seroprevalence of COVID-19 Amongst Health Care Workers in a Tertiary Care Hospital of a Metropolitan City from India. *The Journal of the Association of Physicians of India*. 2020;68(11):14-19.

26. Goenka MK, Shah BB, Goenka U, et al. COVID-19 prevalence among health-care workers of Gastroenterology department: An audit from a tertiary-care hospital in India. *JGH Open*. 2021;5(1):56-63. doi:10.1002/jgh3.12447

27. Satpati P, Sarangi S, Gantait K, et al. *Sero-Surveillance (IgG) of SARS-CoV-2 Among Asymptomatic General Population of Paschim Medinipur, West Bengal, India*. Infectious Diseases (except HIV/AIDS); 2020. doi:10.1101/2020.09.12.20193219

28. Poustchi H, Darvishian M, Mohammadi Z, et al. SARS-CoV-2 antibody seroprevalence in the general population and high-risk occupational groups across 18 cities in Iran: A population-based cross-sectional study. *The Lancet Infectious Diseases*. 2020;0(0). doi:10.1016/S1473-3099(20)30858-6

29. Jones CR, Hamilton FW, Thompson A, Morris TT, Moran E. SARS-CoV-2 IgG seroprevalence in healthcare workers and other staff at North Bristol NHS Trust: A sociodemographic analysis. *Journal of Infection*. 2020;0(0). doi:10.1016/j.jinf.2020.11.036

30. Anna F, Goyard S, Lalanne AI, et al. High seroprevalence but short-lived immune response to SARS-CoV-2 infection in Paris. *medRxiv*. Published online November 2020:2020.10.25.20219030. doi:10.1101/2020.10.25.20219030

31. Erber J, Kappler V, Haller B, et al. Strategies for infection control and prevalence of anti-SARS-CoV-2 IgG in 4,554 employees of a university hospital in Munich, Germany. Published online October 2020. doi:10.1101/2020.10.04.20206136

32. Hanrath AT, Loeff IS van der, Lendrem DW, et al. SARS-CoV-2 testing of 11,884 healthcare workers at an acute NHS hospital trust in England: A retrospective analysis. *medRxiv*. Published online December 2020:2020.12.22.20242362. doi:10.1101/2020.12.22.20242362

33. Rosser JI, Röltgen K, Dymock M, et al. Severe acute respiratory coronavirus virus 2 (SARS-CoV-2) seroprevalence in healthcare personnel in northern California early in the coronavirus disease 2019 (COVID-19) pandemic. *Infection Control & Hospital Epidemiology*. Published online December 2020:1-7. doi:10.1017/ice.2020.1358

34. Silva VO, de Oliveira EL, Castejon MJ, et al. Prevalence of antibodies against sars-cov-2 in professionals of a public health laboratory at são paulo, sp, brazil. *medRxiv*. Published online October 2020. doi:10.1101/2020.10.19.20213421

35. Sabourin KR, Schultz J, Romero J, et al. Risk Factors of SARS-CoV-2 Antibodies in Arapahoe County First Responders - the COVID-19 Arapahoe SErosurveillance Study (CASES) Project. *Journal of Occupational and Environmental Medicine*. Published online December 2020. doi:10.1097/JOM.0000000000002099

36. Yogo N, Greenwood KL, Thompson L, et al. Point prevalence survey to evaluate the seropositivity for coronavirus disease 2019 (COVID-19) among high-risk healthcare workers. *Infection Control and Hospital Epidemiology*. Published online December 2020:1-6. doi:10.1017/ice.2020.1370

37. Figueiredo-Campos P, Blankenhaus B, Mota C, et al. Seroprevalence of anti-SARS-CoV-2 antibodies in COVID-19 patients and healthy volunteers up to 6 months post disease onset. *European Journal of Immunology*. 2020;50(12):2025-2040. doi:10.1002/eji.202048970

For peer review only

38. Gonçalves J, Sousa RL, Jacinto MJ, et al. Evaluating SARS-CoV-2 Seroconversion Following Relieve of Confinement Measures. *Frontiers in Medicine*. 2020;7. doi:10.3389/fmed.2020.603996

39. Fontanet A, Grant R, Tondeur L, et al. SARS-CoV-2 infection in primary schools in northern France: A retrospective cohort study in an area of high transmission. *medRxiv*. Published online June 2020:2020.06.25.20140178. doi:10.1101/2020.06.25.20140178

40. Torres JP, Piñera C, De La Maza V, et al. Severe Acute Respiratory Syndrome Coronavirus 2 Antibody Prevalence in Blood in a Large School Community Subject to a Coronavirus Disease 2019 Outbreak: A Cross-sectional Study. *Clinical Infectious Diseases*. Published online July 2020:ciaa955. doi:10.1093/cid/ciaa955

41. Halatoko WA, KONU YR, Gbeasor-Komlanvi FA, et al. Prevalence of SARS-CoV-2 among high-risk populations in Lom&Eacute (Togo) in 2020. *medRxiv*. Published online August 2020:2020.08.07.20163840. doi:10.1101/2020.08.07.20163840

42. Slusser S. MLB antibody study: 0.7% of those tested had been exposed to coronavirus. *San Francisco Chronicle*. Published online May 2020.

43. Vince A, Zadro R, Šostar Z, et al. SARS-CoV-2 Seroprevalence in a Cohort of Asymptomatic, RT-PCR Negative Croatian First League Football Players. *medRxiv*. Published online November 2020:2020.10.30.20223230. doi:10.1101/2020.10.30.20223230

44. Mack D, Gärtner BC, Rössler A, et al. Prevalence of SARS-CoV-2 IgG antibodies in a large prospective cohort study of elite football players in Germany (MayJune 2020): Implications for a testing protocol in asymptomatic individuals and estimation of the rate of undetected cases. *Clinical Microbiology and Infection*. 2020;27(3):473.e1-473.e4. doi:10.1016/j.cmi.2020.11.033

45. Khan SMS, Qurieshi MA, Haq I, et al. Seroprevalence of SARS-CoV-2 specific IgG antibodies in District Srinagar, northern India A cross-sectional study. *PLOS ONE*. 2020;15(11):e0239303. doi:10.1371/journal.pone.0239303

46. Akinbami LJ, Vuong N, Petersen LR, et al. SARS-CoV-2 Seroprevalence among Healthcare, First Response, and Public Safety Personnel, Detroit Metropolitan Area, Michigan, USA, MayJune 2020 - Volume 26, Number 12December 2020 - Emerging Infectious Diseases journal - CDC. Published online December 2020. doi:10.3201/eid2612.203764

47. Amendola A, Tanzi E, Folgori L, et al. Low seroprevalence of SARS-CoV-2 infection among healthcare workers of the largest children hospital in Milan during the pandemic wave. *Infection Control & Hospital Epidemiology*. Published online August 2020:1-2. doi:10.1017/ice.2020.401

48. Paulo Arnaldo. *Inquérito Sero-Epidemiológico de SARS-CoV-2 Na Cidade de Quelimane (InCOVID 2020)*. República de Moçambique Ministério da Saúde; 2020.

49. Bal A, Brengel-Pesce K, Gaymard A, et al. Clinical and microbiological assessments of COVID-19 in healthcare workers: A prospective longitudinal study. *medRxiv*. Published online November 2020:2020.11.04.20225862. doi:10.1101/2020.11.04.20225862

50. Fernández-Rivas G, Quirant-Sánchez B, González V, et al. Seroprevalence of SARS-CoV-2 IgG Specific Antibodies among Healthcare Workers in the Northern Metropolitan Area of Barcelona, Spain, after the first pandemic wave. *medRxiv*. Published online June 2020:2020.06.24.20135673. doi:10.1101/2020.06.24.20135673

51. Bardai G, Ouellet J, Engelhardt T, Bertolizio G, Wu Z, Rauch F. Prevalence of SARS-CoV-2 infections in a pediatric orthopedic hospital. von Ungern-Sternberg B, ed. *Pediatric Anesthesia*. 2021;31(2):247-248. doi:10.1111/pan.14047

For peer review only

52. Blairon L, Mokrane S, Wilmet A, et al. Large-scale, molecular and serological SARS-CoV-2 screening of healthcare workers in a 4-site public hospital in Belgium after COVID-19 outbreak. *Journal of Infection*. Published online July 2020:S0163445320305144. doi:10.1016/j.jinf.2020.07.033

53. Moreno Borraz LA, Giménez López M, Carrera Lasfuentes P, et al. Prevalencia de infección por coronavirus SARS-CoV-2 en pacientes y profesionales de un hospital de media y larga estancia en España. *Revista Española de Geriatría y Gerontología*. 2020;56(2):75-80. doi:10.1016/j.regg.2020.10.005

54. Brunner WM, Hirabayashi L, Krupa NL, et al. Severe acute respiratory coronavirus virus 2 (SARS-CoV-2) IgG results among healthcare workers in a rural upstate New York hospital system. *Infection Control & Hospital Epidemiology*. Published online October 2020:1-4. doi:10.1017/ice.2020.1296

55. Carozzi FM, Cusi MG, Pistello M, et al. Detection of asymptomatic SARS-CoV-2 infections among healthcare workers: Results from a large-scale screening program based on rapid serological testing. *medRxiv*. Published online August 2020. doi:10.1101/2020.07.30.20149567

56. Vilibic-Cavlek T, Stevanovic V, Tabain I, et al. Severe acute respiratory syndrome coronavirus 2 seroprevalence among personnel in the healthcare facilities of Croatia, 2020. *Revista da Sociedade Brasileira de Medicina Tropical*. 2020;53. doi:10.1590/0037-8682-0458-2020

57. Chibwana MG, Jere KC, kamng'ona R, et al. High SARS-CoV-2 seroprevalence in Health Care Workers but relatively low numbers of deaths in urban Malawi. *medRxiv*. Published online August 2020:2020.07.30.20164970. doi:10.1101/2020.07.30.20164970

58. Coffman B. New Co-Immunity Project data show COVID-19 infection among health care workers may be lower than the general population | UofL News. *UofLNews*. Published online August 2020.

59. Cooper DJ, Lear S, Watson L, et al. A prospective study of risk factors associated with seroprevalence of SARS-CoV-2 antibodies in healthcare workers at a large UK teaching hospital. *medRxiv*. Published online November 2020:2020.11.03.20220699. doi:10.1101/2020.11.03.20220699

60. Denyer S. Japanese firm's blanket testing of employees could serve as model. *LMT Online*. Published online June 2020.

61. Dimeglio C, Herin F, Miedougé M, et al. Screening for SARS-CoV-2 antibodies among healthcare workers in a university hospital in southern France. *Journal of Infection*. 2020;0(0). doi:10.1016/j.jinf.2020.09.035

62. Fuereder T, Berghoff AS, Heller G, et al. SARS-CoV-2 seroprevalence in oncology healthcare professionals and patients with cancer at a tertiary care centre during the COVID-19 pandemic. *ESMO Open*. 2020;5(5). doi:10.1136/esmoopen-2020-000889

63. Fusco FM, Pisaturo M, Iodice V, et al. COVID-19 among healthcare workers in a specialist infectious diseases setting in Naples, Southern Italy: Results of a cross-sectional surveillance study. *Journal of Hospital Infection*. 2020;105(4):596-600. doi:10.1016/j.jhin.2020.06.021

64. Geraci L. Antibody tests show just 2% exposure rate to COVID-19. *The Lancaster News*. Published online May 2020.

65. Eduardo Samo Gudo. *Inquérito Sero-epidemiológico de SARS-CoV-2 na Cidade de Nampula*. República de Moçambique Ministério da Saúde; 2020:19.

66. Hackner K, Errhalt P, Willheim M, et al. Diagnostic accuracy of two commercially available rapid assays for detection of IgG and IgM antibodies to SARS-CoV-2 compared to ELISA in a low-prevalence population. *Research Square*. Published online August 2020. doi:10.21203/rs.3.rs-50887/v1

For peer review only

67. Haq M, Rehman A, Noor M, et al. Seroprevalence and Risk Factors of SARS CoV-2 in Health Care Workers of Tertiary-Care Hospitals in the Province of Khyber Pakhtunkhwa, Pakistan. *medRxiv*. Published online September 2020:2020.09.29.20203125. doi:10.1101/2020.09.29.20203125

68. He L, Zeng Y, Zeng C, et al. Positive Rate of Serology and RT-PCR for COVID-19 among healthcare workers during different periods in Wuhan, China. *Journal of Infection*. Published online August 2020. doi:10.1016/j.jinf.2020.08.027

69. Herzberg J, Vollmer T, Fischer B, et al. Prospective Sero-epidemiological Evaluation of SARS-CoV-2 among Health Care Workers in a German Secondary Care Hospital. *International Journal of Infectious Diseases*. 2021;102:136-143. doi:10.1016/j.ijid.2020.10.026

70. Jeremias A, Nguyen J, Levine J, et al. Prevalence of SARS-CoV-2 Infection Among Health Care Workers in a Tertiary Community Hospital. *JAMA Internal Medicine*. Published online August 2020. doi:10.1001/jamainternmed.2020.4214

71. Jespersen S, Mikkelsen S, Greve T, et al. Severe Acute Respiratory Syndrome Coronavirus 2 Seroprevalence Survey Among 17 971 Healthcare and Administrative Personnel at Hospitals, Prehospital Services, and Specialist Practitioners in the Central Denmark Region. *Clinical Infectious Diseases*. Published online October 2020:ciaa1471. doi:10.1093/cid/ciaa1471

72. Kassem AM, Talaat H, Shawky S, et al. SARS-CoV-2 infection among healthcare workers of a gastroenterological service in a tertiary care facility. *Arab Journal of Gastroenterology*. 2020;21(3):151-155. doi:10.1016/j.ajg.2020.07.005

73. Kern PM, Müller H-H, Menzel T, Weisser H. Studie zur Immunität gegen SARS-CoV-2: Keine signifikante humorale Immunität gegen SARS-CoV-2 im medizinischen Personal eines Klinikums der Maximalversorgung und in der Stadtregion Fulda. *Der Klinikarzt*. 2020;49(06):268-273. doi:10.1055/a-1198-1243

74. Khalil A, Hill R, Wright A, Ladhani S, O'Brien P. SARS-CoV-2-Specific Antibody Detection in Healthcare Workers in a UK Maternity Hospital: Correlation With SARS-CoV-2 RT-PCR Results. *Clinical Infectious Diseases*. 2020;(ciaa893). doi:10.1093/cid/ciaa893

75. Kumar A, Sathyapalan D, Ramachandran A, Subhash K, Biswas L, Beena KV. SARS-CoV-2 antibodies in healthcare workers in a large university hospital, Kerala, India. *Clinical Microbiology and Infection*. 2021;27(3):481-483. doi:10.1016/j.cmi.2020.09.013

76. Lackermair K, William F, Grzanna N, et al. Infection with SARS-CoV-2 in primary care health care workers assessed by antibody testing. *Family Practice*. Published online August 2020:cmaa078. doi:10.1093/fampra/cmaa078

77. Lahner E, Dilaghi E, Prestigiacomo C, et al. Prevalence of Sars-Cov-2 Infection in Health Workers (HWs) and Diagnostic Test Performance: The Experience of a Teaching Hospital in Central Italy. *International Journal of Environmental Research and Public Health*. 2020;17(12). doi:10.3390/ijerph17124417

78. Liu M, Cheng S-Z, Xu K-W, et al. Use of personal protective equipment against coronavirus disease 2019 by healthcare professionals in Wuhan, China: Cross sectional study. *BMJ*. 2020;369. doi:10.1136/bmj.m2195

79. Liu T, Wu S, Tao H, Zeng G, Zhou F, Wang X. Prevalence of IgG Antibodies to SARS-CoV-2 in Wuhan Implications for the Longevity of Antibodies Against SARS-CoV-2. *Research Square*. Published online November 2020. doi:10.21203/rs.3.rs-99748/v1

80. Lorenzo D, Carrisi C. COVID-19 exposure risk for family members of healthcare workers: An observational study. *International Journal of Infectious Diseases*. 2020;98:287-289. doi:10.1016/j.ijid.2020.06.106

For peer review only

81. Mussagy Mahomed. *Inquérito Sero-Epidemiológico de SARS-CoV-2 Na Cidade de Tete (InCOVID 2020)*. República de Moçambique Ministério da Saúde; 2020.

82. Arlete Mahumane. *Inquérito Sero-Epidemiológico de SARS-CoV-2 Na Cidade de Chimoio (InCOVID 2020)*. República de Moçambique Ministério da Saúde; 2020.

83. Majdoubi A, Michalski C, O'Connell SE, et al. Antibody reactivity to SARS-CoV-2 is common in unexposed adults and infants under 6 months. *medRxiv*. Published online November 2020:2020.10.05.20206664. doi:10.1101/2020.10.05.20206664

84. Majiya H, Aliyu-Paiko M, Balogu VT, et al. Seroprevalence of COVID-19 in Niger State. *medRxiv*. Published online August 2020. doi:10.1101/2020.08.04.20168112

85. Fill Malfertheiner S, Brandstetter S, Roth S, et al. Immune response to SARS-CoV-2 in health care workers following a COVID-19 outbreak: A prospective longitudinal study. *Journal of Clinical Virology*. 2020;130:104575. doi:10.1016/j.jcv.2020.104575

86. Martin C, Montesinos I, Dauby N, et al. Dynamics of SARS-CoV-2 RT-PCR positivity and seroprevalence among high-risk healthcare workers and hospital staff. *Journal of Hospital Infection*. 2020;106(1):102-106. doi:10.1016/j.jhin.2020.06.028

87. de Melo MS, Borges LP, de Souza DRV, et al. *Anti-SARS-CoV-2 IgM and IgG Antibodies in Health Workers in Sergipe, Brazil*. Infectious Diseases (except HIV/AIDS); 2020. doi:10.1101/2020.09.24.20200873

88. Morcuende M, Guglielminotti J, Landau R. Anesthesiologists' and Intensive Care Providers' Exposure to COVID-19 Infection in a New York City Academic Center: A Prospective Cohort Study Assessing Symptoms and COVID-19 Antibody Testing. *Anesthesia and analgesia*. 2020;131(3):669-676. doi:10.1213/ANE.0000000000005056

89. Moscola J, Sembajwe G, Jarrett M, et al. Prevalence of SARS-CoV-2 Antibodies in Health Care Personnel in the New York City Area. *JAMA*. 2020;324(9):893-895. doi:10.1001/jama.2020.14765

90. Nishida T, Iwahashi H, Yamauchi K, et al. Seroprevalence of SARS-CoV-2 Antibodies Among 925 Staff Members in an Urban Hospital Accepting COVID-19 Patients in Osaka Prefecture, Japan. *medRxiv*. Published online January 2020:2020.09.10.20191866. doi:10.1101/2020.09.10.20191866

91. Olalla J, Correa AM, Martín-Escalante MD, et al. Search for asymptomatic carriers of SARS-CoV-2 in healthcare workers during the pandemic: A Spanish experience. *QJM: An International Journal of Medicine*. 2020;(hcaa238). doi:10.1093/qjmed/hcaa238

92. Pallett SJC, Rayment M, Patel A, et al. Point-of-care serological assays for delayed SARS-CoV-2 case identification among health-care workers in the UK: A prospective multicentre cohort study. *The Lancet Respiratory Medicine*. 2020;8(9):885-894. doi:10.1016/S2213-2600(20)30315-5

93. Péré H, Wack M, Védie B, et al. Sequential SARS-CoV-2 IgG assays as confirmatory strategy to confirm equivocal results: Hospital-wide antibody screening in 3,569 staff health care workers in Paris. *Journal of Clinical Virology*. 2020;132:104617. doi:10.1016/j.jcv.2020.104617

94. Poulikakos D, Sinha S, Kalra PA. SARS-CoV-2 antibody screening in healthcare workers in a tertiary centre in North West England. *Journal of clinical virology : the official publication of the Pan American Society for Clinical Virology*. 2020;129:104545-104545. doi:10.1016/j.jcv.2020.104545

95. Psichogiou M, Karabinis A, Pavlopoulou I, et al. Antibodies against SARS-CoV-2 among health care workers in a country with low burden of COVID-19. *medRxiv*. Published online June 2020. doi:10.1101/2020.06.23.20137620

For peer review only

96. Kolthur-Seetharam U, Shah D, Shastri J, et al. *SARS-CoV2 Serological Survey in Mumbai by NITI-BMC-TIFR: Preliminary Report of Round-2*. NITI-BMC-TIFR; 2020.

97. Shields AM, Faustini SE, Perez-Toledo M, et al. SARS-CoV-2 seroconversion in health care workers. *medRxiv*. Published online May 2020:2020.05.18.20105197. doi:10.1101/2020.05.18.20105197

98. Ismael Amaral Silva PA, Ismael C, Marchon da Silva C, Domenge C. 1761P Universal screening of SARS-CoV-2 of oncology healthcare workers a Brazilian experience. *Annals of Oncology*. 2020;31:S1024. doi:10.1016/j.annonc.2020.08.1825

99. Solodky ML, Galvez C, Russias B, et al. Lower detection rates of SARS-COV2 antibodies in cancer patients versus health care workers after symptomatic COVID-19. *Annals of Oncology*. 2020;31(8):1087-1088. doi:10.1016/j.annonc.2020.04.475

100. Soriano V, Meiriño R, Corral O, Guallar MP. Severe Acute Respiratory Syndrome Coronavirus 2 Antibodies in Adults in Madrid, Spain. *Clinical Infectious Diseases*. 2020;(ciaa769). doi:10.1093/cid/ciaa769

101. Instituto Nazionale di Statistica. *PRIMI RISULTATI DELL'INDAGINE DI SIEROPREVALENZA SUL SARS-CoV-2*. Instituto Nazionale di Statistica; 2020.

102. Steensels D, Oris E, Coninx L, et al. Hospital-Wide SARS-CoV-2 Antibody Screening in 3056 Staff in a Tertiary Center in Belgium. *JAMA*. 2020;(7501160). doi:10.1001/jama.2020.11160

103. Stock AD, Bader ER, Cezayirli P, et al. COVID-19 Infection Among Healthcare Workers: Serological Findings Supporting Routine Testing. *Frontiers in Medicine*. 2020;7. doi:10.3389/fmed.2020.00471

104. Takita M, Matsumura T, Yamamoto K, et al. Geographical Profiles of COVID-19 Outbreak in Tokyo: An Analysis of the Primary Care ClinicBased Point-of-Care Antibody Testing. *Journal of Primary Care & Community Health*. 2020;11:215013272094269. doi:10.1177/2150132720942695

105. Tong X, Ning M, Huang R, et al. Surveillance of SARS-CoV-2 infection among frontline health care workers in Wuhan during COVID-19 outbreak. *Immunity, Inflammation and Disease*. 2020;8(4):840-843. doi:10.1002/iid3.340

106. Trieu M-C, Bansal A, Madsen A, et al. SARS-CoV-2Specific Neutralizing Antibody Responses in Norwegian Health Care Workers After the First Wave of COVID-19 Pandemic: A Prospective Cohort Study. *The Journal of Infectious Diseases*. 2020;2021-(jiaa737). doi:10.1093/infdis/jiaa737

107. Tu D, Shu J, Wu X, et al. Immunological detection of serum antibodies in pediatric medical workers exposed to varying levels of SARS-CoV-2. *The Journal of Infection*. 2021;82(1):159-198. doi:10.1016/j.jinf.2020.07.023

108. Valdivia A, Torres I, Huntley D, et al. Caveats in interpreting SARS-CoV-2 IgM+/IgG- antibody profile in asymptomatic health care workers. *Journal of Medical Virology*. 2020;n/a(n/a). doi:10.1002/jmv.26400

109. Chafloque-Vasquez RA, Pampa-Espinoza L, Salinas JCC. Seroprevalence of COVID-19 in workers in a hospital in the Peruvian Amazon. *ACTA MEDICA PERUANA*. 2020;37(3). doi:10.35663/amp.2020.373.1050

110. Edna Viegas. *Inquérito Sero-Epidemiológico de SARS-CoV-2 Na Cidade de Maputo (InCOVID 2020)*. República de Moçambique Ministério da Saúde; 2020.

For peer review only

111. Vlachoyiannopoulos P, Alexopoulos H, Apostolidi I, et al. Anti-SARS-CoV-2 antibody detection in healthcare workers of two tertiary hospitals in Athens, Greece. *Clinical Immunology*. 2020;221:108619. doi:10.1016/j.clim.2020.108619

112. Dalla Volta A, Valcamonico F, Pedersini R, et al. The Spread of SARS-CoV-2 Infection Among the Medical Oncology Staff of ASST Spedali Civili of Brescia: Efficacy of Preventive Measures. *Frontiers in Oncology*. 2020;10:1574. doi:10.3389/fonc.2020.01574

113. Ward H, Cooke G, Atchison C, et al. Declining prevalence of antibody positivity to SARS-CoV-2: A community study of 365,000 adults. *medRxiv*. Published online October 2020:2020.10.26.20219725. doi:10.1101/2020.10.26.20219725

114. Xiong S, Guo C, Dittmer U, Zheng X, Wang B. The prevalence of antibodies to SARS-CoV-2 in asymptomatic healthcare workers with intensive exposure to COVID-19. *medRxiv*. Published online June 2020:2020.05.28.20110767. doi:10.1101/2020.05.28.20110767

115. Zhang J, Liu J, Li N, et al. Serological detection of 2019-nCoV respond to the epidemic: A useful complement to nucleic acid testing. *medRxiv*. Published online March 2020:2020.03.04.20030916. doi:10.1101/2020.03.04.20030916

116. Zhao D, Wang M, Wang M, et al. Asymptomatic infection by SARS-CoV-2 in healthcare workers: A study in a large teaching hospital in Wuhan, China. *International Journal of Infectious Diseases*. 2020;99:219-225. doi:10.1016/j.ijid.2020.07.082

117. Ahmad K, Rezvanizadeh V, Dahal S, et al. COVID-19 IgG/IgM antibody testing in Los Angeles County, California. *European Journal of Clinical Microbiology & Infectious Diseases*. Published online November 2020. doi:10.1007/s10096-020-04111-3

118. Halbrook M, Gadoth A, Martin-Blais R, et al. Incidence of SARS-CoV-2 infection among asymptomatic frontline health workers in Los Angeles County, California. *medRxiv*. Published online November 2020:2020.11.18.20234211. doi:10.1101/2020.11.18.20234211

119. Iwuji K, Islam E, Berdine G, Nugent K, Test V, Tijerina A. Prevalence of Coronavirus Antibody Among First Responders in Lubbock, Texas. *Journal of Primary Care & Community Health*. 11:2150132720971390. doi:10.1177/2150132720971390

120. Parker-Magyar A. Few among Long Hill first responders test positive for COVID-19 antibodies. *Echoes Sentinel*. Published online June 2020.

121. Caban-Martinez AJ, Schaefer-Solle N, Santiago K, et al. Epidemiology of SARS-CoV-2 antibodies among firefighters/paramedics of a US fire department: A cross-sectional study. *Occupational and Environmental Medicine*. 2020;77(12):857-861. doi:10.1136/oemed-2020-106676

122. Staletovich J. South Florida Cities Begin Testing Employees For COVID-19 Antibodies. *WLRN*. Published online May 2020.

123. Hibino M, Iwabuchi S, Munakata H. SARS-CoV-2 IgG seroprevalence among medical staff in a general hospital that treated patients with COVID-19 in Japan: Retrospective evaluation of nosocomial infection control. *Journal of Hospital Infection*. 2020;107:103-104. doi:10.1016/j.jhin.2020.10.001

124. Calcagno A, Ghisetti V, Emanuele T, et al. Risk for SARS-CoV-2 Infection in Healthcare Workers, Turin, Italy. *Emerging Infectious Diseases*. 2021;27(1):303-305. doi:10.3201/eid2701.203027

125. Alharbi SA, Almutairi AZ, Jan AA, Alkhalify AM. Enzyme-Linked Immunosorbent Assay for the Detection of Severe Acute Respiratory Syndrome Coronavirus 2 (SARS-CoV-2) IgM/IgA and IgG Antibodies Among Healthcare Workers. *Cureus*. Published online September 2020. doi:10.7759/cureus.10285

For peer review only

126. Chau NVV, Toan LM, Man DNH, et al. Absence of SARS-CoV-2 antibodies in health care workers of a tertiary referral hospital for COVID-19 in southern Vietnam. *Journal of Infection*. 2020;82(1):e36-e37. doi:10.1016/j.jinf.2020.11.018

127. Khan MS, Haq I, Qurieshi MA, et al. SARS-CoV-2 seroprevalence in healthcare workers of dedicated-COVID hospitals and nonCOVID hospitals of District Srinagar, Kashmir. *medRxiv*. Published online October 2020:2020.10.23.20218164. doi:10.1101/2020.10.23.20218164

128. Costa SF, Giavina-Bianchi P, Buss L, et al. SARS-CoV-2 seroprevalence and risk factors among oligo/asymptomatic healthcare workers(HCW): Estimating the impact of community transmission. *Clinical Infectious Diseases*. 2020;(ciaa1845). doi:10.1093/cid/ciaa1845

129. Mohr N, Harland K, Krishnadasan A, Santibanez S, Talan D. Diagnosed and Undiagnosed COVID-19 in US Emergency Department Health Care Personnel: A Cross-sectional Analysis. *Annals of Emergency Medicine*. Published online December 2020. doi:10.1016/j.annemergmed.2020.12.007

130. Noor M, Haq M, Ul Haq N, et al. Does Working in a COVID-19 Receiving Health Facility Influence Seroprevalence to SARS-CoV-2? *Cureus*. Published online November 2020. doi:10.7759/cureus.11389

131. Singhal T, Shah S, Naik R, Kazi A, Thakkar P. Prevalence of COVID-19 Antibodies in Healthcare Workers at the Peak of the Pandemic in Mumbai, India: A Preliminary Study. *Indian Journal of Medical Microbiology*. 2020;38(3):461-463. doi:10.4103/ijmm.IJMM_20_308

132. Dacosta-Urbieta A, Rivero-Calle I, Pardo-Seco J, et al. Seroprevalence of SARS-CoV-2 Among Pediatric Healthcare Workers in Spain. *Frontiers in Pediatrics*. 2020;8. doi:10.3389/fped.2020.00547

133. Sartore-Bianchi A, Patelli G, Tosi F, et al. INCIDENCE OF SARS-COV-2 INFECTION IN PATIENTS WITH ACTIVE CANCER: MONO-INSTITUTIONAL SERIES OF A COMPREHENSIVE CANCER INSTITUTION IN LOMBARDY DURING THE COVID-19 PANDEMIC (NIGUARDA CANCER CENTER, MILANO, ITALY). In: *Tumori Journal*. Vol 106. AIOM Abstracts.; 2020:1-215. doi:10.1177/0300891620953388

134. Brousseau N, Morin L, Ouakki M, et al. *COVID-19 : Étude de séroprévalence chez des travailleurs de la santé de centres hospitaliers au Québec*. Institut National de Sante Publique du Quebec; 2020:20.

135. Chen Y, Tong X, Wang J, et al. High SARS-CoV-2 antibody prevalence among healthcare workers exposed to COVID-19 patients. *The Journal of Infection*. 2020;81(3):420-426. doi:10.1016/j.jinf.2020.05.067

136. Favara DM, Cooke A, Doffinger R, McAdam K, Corrie P, Ainsworth NL. COVID-19 Serology in Oncology Staff Study: Understanding SARS-CoV-2 in the Oncology Workforce. *Clinical Oncology (Royal College of Radiologists (Great Britain)*. 2021;33(1):e61-e63. doi:10.1016/j.clon.2020.07.015

137. Fujita K, Shinpei Kada, Osamu Kanai, et al. Quantitative SARS-CoV-2 antibody screening of healthcare workers in the southern part of Kyoto city during the COVID-19 peri-pandemic period. *medRxiv*. Published online May 2020.

138. Godbout EJ, Pryor R, Harmon M, et al. Severe acute respiratory coronavirus virus 2 (SARS-CoV-2) seroprevalence among healthcare workers in a low prevalence region. *Infection Control & Hospital Epidemiology*. Published online December 2020:1-3. doi:10.1017/ice.2020.1374

139. Houlihan CF, Vora N, Byrne T, et al. Pandemic peak SARS-CoV-2 infection and seroconversion rates in London frontline health-care workers. *The Lancet*. 2020;396(10246):e6-e7. doi:10.1016/S0140-6736(20)31484-7

For peer review only

140. Insúa C, Stedile G, Figueroa V, et al. Seroprevalence of SARS-CoV-2 antibodies among physicians from a children's hospital. *Archivos Argentinos De Pediatria*. 2020;118(6):381-385. doi:10.5546/aap.2020.eng.381

141. Kohler PP, Kahlert CR, Sumer J, et al. Prevalence of SARS-CoV-2 antibodies among Swiss hospital workers: Results of a prospective cohort study. *Infection Control & Hospital Epidemiology*. Published online October 2020:1-5. doi:10.1017/ice.2020.1244

142. Kumar N, Bhartiya S, Singh T. Duration of anti-SARS-CoV-2 antibodies much shorter in India. *Vaccine*. 2021;39(6):886-888. doi:10.1016/j.vaccine.2020.10.094

143. Mesnil M, Joubel K, Yavchitz A, Miklaszewski N, Devys J-M. Seroprevalence of SARS-Cov-2 in 646 professionals at the Rothschild Foundation Hospital (ProSeCoV study). *Anaesthesia Critical Care & Pain Medicine*. 2020;39(5):595-596. doi:10.1016/j.accpm.2020.08.003

144. Missaglia R, Belingheri M, Antolini L, et al. SARS-CoV-2 pandemia in Lombardy: The impact on family Paediatricians. *Italian Journal of Pediatrics*. 2020;46(1):184. doi:10.1186/s13052-020-00950-0

145. Orth-Höller D, Eigentler A, Weseslindtner L, Möst J. Antibody kinetics in primary- and secondary-care physicians with mild to moderate SARS-CoV-2 infection. *Emerging Microbes & Infections*. 2020;9(1):1692-1694. doi:10.1080/22221751.2020.1793690

146. Plebani M, Padoan A, Fedeli U, et al. SARS-CoV-2 serosurvey in health care workers of the Veneto Region. *Clinical Chemistry and Laboratory Medicine (CCLM)*. 2020;58(12):2107-2111. doi:10.1515/cclm-2020-1236

147. Rudberg A-S, Havervall S, Månberg A, et al. SARS-CoV-2 exposure, symptoms and seroprevalence in healthcare workers in Sweden. *Nature Communications*. 2020;11(1):5064. doi:10.1038/s41467-020-18848-0

148. Schmidt SB, Grüter L, Boltzmann M, Rollnik JD. Prevalence of serum IgG antibodies against SARS-CoV-2 among clinic staff. Adrish M, ed. *PLOS ONE*. 2020;15(6):e0235417. doi:10.1371/journal.pone.0235417

149. Sotgiu G, Barassi A, Miozzo M, et al. SARS-CoV-2 specific serological pattern in healthcare workers of an Italian COVID-19 forefront hospital. *BMC Pulmonary Medicine*. 2020;20(1):203. doi:10.1186/s12890-020-01237-0

150. Venugopal U, Jilani N, Rabah S, et al. SARS-CoV-2 seroprevalence among health care workers in a New York City hospital: A cross-sectional analysis during the COVID-19 pandemic. *International Journal of Infectious Diseases*. 2020;102:63-69. doi:10.1016/j.ijid.2020.10.036

151. Racine-Brzostek SE, Yang HS, Chadburn A, et al. COVID-19 Viral and Serology Testing in New York City Health Care Workers. *American Journal of Clinical Pathology*. 2020;154(5):592-595. doi:10.1093/ajcp/aqaa142

152. Hoffmann S, Spallek J, Heinz-Detlef G, Schiebel J, Hufert F. Testing the backbone of the healthcare system: A prospective serological-epidemiological cohort study of healthcare workers in rural Germany. Published online September 2020. doi:10.21203/rs.3.rs-84703/v1

153. Patel MM, Thornburg NJ, Stubblefield WB, et al. Change in Antibodies to SARS-CoV-2 Over 60 Days Among Health Care Personnel in Nashville, Tennessee. *JAMA*. 2020;324(17):1781. doi:10.1001/jama.2020.18796

For peer review only

154. Self WH, Tenforde MW, Stubblefield WB, et al. Seroprevalence of SARS-CoV-2 Among Frontline Health Care Personnel in a Multistate Hospital Network 13 Academic Medical Centers, AprilJune 2020. *MMWR Morbidity and Mortality Weekly Report*. 2020;69(35):1221-1226. doi:10.15585/mmwr.mm6935e2

155. Shah VP, Hainy CM, Swift MD, Breeher LE, Theel ES, Sampathkumar P. Unrecognized severe acute respiratory coronavirus virus 2 (SARS-CoV-2) seroprevalence among healthcare personnel in a low-prevalence area. *Infection Control & Hospital Epidemiology*. Published online November 2020:1-3. doi:10.1017/ice.2020.1341

156. Bampoe S, Lucas DN, Neall G, et al. A cross-sectional study of immune seroconversion to SARS-CoV-2 in front-line maternity health professionals. *medRxiv*. Published online June 2020. doi:10.1101/2020.06.24.20139352

157. Dimcheff DE, Schildhouse RJ, Hausman MS, et al. Seroprevalence of severe acute respiratory syndrome coronavirus-2 (SARS-CoV-2) infection among Veterans Affairs healthcare system employees suggests higher risk of infection when exposed to SARS-CoV-2 outside the work environment. *Infection Control & Hospital Epidemiology*.:1-7. doi:10.1017/ice.2020.1220

158. Finkenzeller T, Faltlhauser A, Dietl K-H, et al. SARS-CoV-2-Antikörper bei Intensiv- und Klinikpersonal. *Medizinische Klinik - Intensivmedizin und Notfallmedizin*. 2020;115(3):139-145. doi:10.1007/s00063-020-00761-5

159. Grant JJ, Wilmore SMS, McCann NS, et al. Seroprevalence of SARS-CoV-2 antibodies in healthcare workers at a London NHS Trust. *Infection Control & Hospital Epidemiology*. Published online August 2020:1-3. doi:10.1017/ice.2020.402

160. Mansour M, Leven E, Muellers K, Stone K, Mendu DR, Wajnberg A. Prevalence of SARS-CoV-2 Antibodies Among Healthcare Workers at a Tertiary Academic Hospital in New York City. *Journal of General Internal Medicine*. 2020;35(8):2485-2486. doi:10.1007/s11606-020-05926-8

161. Martín V, Fernández-Villa T, Lamuedra Gil de Gomez M, et al. Prevalence of SARS-CoV-2 infection in general practitioners and nurses in primary care and nursing homes in the Healthcare Area of León and associated factors. *COVID19 en Atención Primaria*. 2020;46:35-39. doi:10.1016/j.semerg.2020.05.014

162. Meissner EG, Litwin C, Crocker T, Mack E, Card L. 460. Point-of-Care, In-Home SARS-CoV-2 IgG Antibody Testing to Assess Seroprevalence in At-Risk Health Care Workers. *Open Forum Infectious Diseases*. 2020;7(Supplement_1):S297-S297. doi:10.1093/ofid/ofaa439.653

163. Mostafa A, Kandil S, El-Sayed MH, et al. Universal COVID-19 screening of 4040 health care workers in a resource-limited setting: An Egyptian pilot model in a university with 12 public hospitals and medical centers. *International Journal of Epidemiology*. 2020;(dyaa173). doi:10.1093/ije/dyaa173

164. Paradiso AV, Summa simona D, Silvestris N, et al. COVID-19 SCREENING AND MONITORING OF ASYMPTOMATIC HEALTH WORKERS WITH A RAPID SEROLOGICAL TEST. *medRxiv*. Published online May 2020:2020.05.05.20086017. doi:10.1101/2020.05.05.20086017

165. Sydney ER, Kishore P, Laniado I, Rucker LM, Bajaj K, Zinaman MJ. Antibody evidence of SARS-CoV-2 infection in healthcare workers in the Bronx. *Infection Control & Hospital Epidemiology*. 2020;41(11):1348-1349. doi:10.1017/ice.2020.437

166. Zhou F, Li J, Lu M, et al. Tracing asymptomatic SARS-CoV-2 carriers among 3674 hospital staff:A cross-sectional survey. *EClinicalMedicine*. 2020;26. doi:10.1016/j.eclinm.2020.100510

167. Buntinx F, Claes P, Gulikers M, et al. Added value of anti-SARS-CoV-2 antibody testing in a Flemish nursing home during an acute COVID-19 outbreak in April 2020. *Acta Clinica Belgica*. 2020;0(0):1-6. doi:10.1080/17843286.2020.1834285

For peer review only

168. Reuben J, Sherman A, Ellison JA, et al. SARS-CoV-2 Seroprevalence among First Responders in the District of Columbia, May July 2020. *medRxiv*. Published online November 2020:2020.11.25.20225490. doi:10.1101/2020.11.25.20225490

169. Saberian P, Mireskandari SM, Baratloo A, et al. Antibody Rapid Test Results in Emergency Medical Services Personnel during COVID-19 Pandemic; a Cross Sectional study. *Archives of Academic Emergency Medicine*. 2020;9(1).

170. Tarabichi Y, Watts B, Collins T, et al. SARS-CoV-2 Infection among Serially Tested Emergency Medical Services Workers. *Prehospital Emergency Care*. 2020;0(0):1-7. doi:10.1080/10903127.2020.1831668

171. Vijh R, Ghafari C, Hayden A, et al. Serological survey following SARS-COV-2 outbreaks at long-term care facilities in metro Vancouver, British Columbia: Implications for outbreak management and infection control policies. *American Journal of Infection Control*. Published online October 2020. doi:10.1016/j.ajic.2020.10.009

172. Bhattacharya D, Winnett A, Fulcher JA, et al. 70. Lack of SARS-CoV-2 Antibody Seroconversion After Prompt Identification and Cohorting of Sentinel sars-cov-2-positive Residents in a Skilled Nursing Facility. *Open Forum Infectious Diseases*. 2020;7(Supplement_1):S165-S166. doi:10.1093/ofid/ofaa439.380

173. Pérez-García F, Pérez-Zapata A, Arcos N, et al. Severe acute respiratory coronavirus virus 2 (SARS-CoV-2) infection among hospital workers in a severely affected institution in Madrid, Spain: A surveillance cross-sectional study. *Infection Control & Hospital Epidemiology*. Published online October 2020:1-7. doi:10.1017/ice.2020.1303

174. Pérez-García F, Pérez-Zapata A, Arcos N, et al. Severe acute respiratory coronavirus virus 2 (SARS-CoV-2) infection among hospital workers in a severely affected institution in Madrid, Spain: A surveillance cross-sectional study. *Infection Control & Hospital Epidemiology*. 2021;42(7):803-809. doi:10.1017/ice.2020.1303

175. Mughal MS, Kaur IP, Patton CD, Mikhail NH, Vareechon C, Granet KM. The prevalence of severe acute respiratory coronavirus virus 2 (SARS-CoV-2) IgG antibodies in intensive care unit (ICU) healthcare personnel (HCP) and its implicationsa single-center, prospective, pilot study. *Infection Control & Hospital Epidemiology*. Published online June 2020:1-2. doi:10.1017/ice.2020.298

176. Rao S. Covid-19: Jayadeva says its survey hints at herd immunity. *The Times of India*. Published online June 2020.

177. Shukla V, Lau CSM, Towns M, et al. COVID-19 Exposure Among First Responders in Arizona. *Journal of Occupational and Environmental Medicine*. 2020;62(12).

178. Gray A. Prevalence Of COVID-19 Antibodies In Washoe Co. Expected To Be Low. *KUNR*. Published online June 2020.

179. Chughtai O, Batool H, Khan M, Chughtai A. Frequency of COVID-19 IgG Antibodies among Special Police Squad Lahore, Pakistan. *Journal of the College of Physicians and Surgeons Pakistan*. 2020;30(7):735-739. doi:10.29271/jcpsp.2020.07.735

180. Gujski M, Jankowski M, Pinkas J, et al. Prevalence of Current and Past SARS-CoV-2 Infections among Police Employees in Poland, JuneJuly 2020. *Journal of Clinical Medicine*. 2020;9(10):3245. doi:10.3390/jcm9103245

181. Jerónimo Langa. *Inquérito Sero-Epidemiológico de SARS-CoV-2 Na Cidade de Lichinga (InCOVID 2020)*. República de Moçambique Ministério da Saúde; 2020.

For peer review only

182. Ivalda Macicame. *Inquérito Sero-Epidemiológico de SARS-CoV-2 Na Província de Maputo (InCOVID 2020)*. República de Moçambique Ministério da Saúde; 2020.

183. Al-Thani MH, Farag E, Bertollini R, et al. Seroprevalence of SARS-CoV-2 infection in the craft and manual worker population of Qatar. *medRxiv*. Published online November 2020:2020.11.24.20237719. doi:10.1101/2020.11.24.20237719

184. Epstude J, Harsch IA. Seroprevalence of COVID-19 antibodies in the cleaning and oncological staff of a municipal clinic. *GMS Hygiene and Infection Control; 15:Doc18*. Published online July 2020. doi:10.3205/DGKH000353

185. Hassan SS, Seigerud Å, Mühr LSA, et al. SARS-CoV-2 infections among personnel providing home care services for the elderly in Stockholm, Sweden. *medRxiv*. Published online December 2020. doi:10.1101/2020.12.18.20248511

186. Ladhani SN, Jeffery-Smith A, Patel M, et al. High prevalence of SARS-CoV-2 antibodies in care homes affected by COVID-19: Prospective cohort study, England. *EClinicalMedicine*. 2020;28. doi:10.1016/j.eclinm.2020.100597

187. Lindahl JF, Hoffman T, Esmaeilzadeh M, et al. High seroprevalence of SARS-CoV-2 in elderly care employees in Sweden. *Infection Ecology & Epidemiology*. 2020;10(1):1789036. doi:10.1080/20008686.2020.1789036

188. Regan T. Fellowship Village Benefits from Covid-19 Antibody Tests. *Senior Housing News*. Published online June 2020.

189. Alali WQ, Bastaki H, Longenecker JC, et al. Seroprevalence of SARS-CoV-2 in migrant workers in Kuwait. *Journal of Travel Medicine*. 2020;(taaa223). doi:10.1093/jtm/taaa223

190. Addetia A, Crawford KHD, Dingens A, et al. Neutralizing Antibodies Correlate with Protection from SARS-CoV-2 in Humans during a Fishery Vessel Outbreak with a High Attack Rate. McAdam AJ, ed. *Journal of Clinical Microbiology*. 2020;58(11):e02107-20, /jcm/58/11/JCM.02107-20.atom. doi:10.1128/JCM.02107-20

191. Picon RV, Carreno I, da Silva AA, et al. Coronavirus disease 2019 population-based prevalence, risk factors, hospitalization, and fatality rates in southern Brazil. *International Journal of Infectious Diseases*. 2020;100:402-410. doi:10.1016/j.ijid.2020.09.028

192. D B, L B, P T, Pa P, A B, U L. Effectiveness of the measures aimed at containing Sars-cov-2 virus spreading in work settings: A survey in companies based in the Veneto region of Italy. *La Medicina del lavoro*. Published online October 2020. doi:10.23749/mdl.v111i5.10037

193. Xu X, Sun J, Nie S, et al. Seroprevalence of immunoglobulin M and G antibodies against SARS-CoV-2 in China. *Nature Medicine*. 2020;26(8):1193-1195. doi:10.1038/s41591-020-0949-6

194. Chamie G, Marquez C, Crawford E, et al. Community Transmission of Severe Acute Respiratory Syndrome Coronavirus 2 Disproportionately Affects the Latinx Population During Shelter-in-Place in San Francisco. *Clinical Infectious Diseases*. Published online August 2020:ciaa1234. doi:10.1093/cid/ciaa1234

195. McLaughlin C, Doll MK, Morrison KT, et al. High Community SARS-CoV-2 Antibody Seroprevalence in a Ski Resort Community, Blaine County, Idaho, US. Preliminary Results. *medRxiv*. Published online July 2020. doi:10.1101/2020.07.19.20157198

196. Muñoz L, Pífano M, Bolzán A, et al. *Surveillance and Seroprevalence: Evaluation of IgG Antibodies for SARS-Cov2 by ELISA in the Popular Neighborhood Villa Azul, Quilmes, Province of Buenos Aires, Argentina*.; 2020. doi:10.1590/SciELOPreprints.1147

For peer review only

Occupation and SARS-CoV-2 seroprevalence studies: a systematic review

Journal:	*BMJ Open*
Manuscript ID	bmjopen-2022-063771.R2
Article Type:	Original research
Date Submitted by the Author:	03-Feb-2023
Complete List of Authors:	Boucher, Emily; University of Calgary Cumming School of Medicine,<br>Cao, Christian; University of Calgary, Cumming School of Medicine<br>D'Mello, Sean; University of Waterloo<br>Duarte, Nathan; McGill University, Faculty of Engineering<br>Donnici, Claire; University of Calgary, Cumming School of Medicine<br>Duarte, Natalie; University of Toronto, Faculty of Arts and Science<br>Bennett, Graham; McGill University, Department of Economics<br>Consortium, SeroTracker ; University of Calgary<br>Adisesh, Anil; Unity Health Toronto, St. Michael's Hospital; University of Toronto, Division of Occupational Medicine<br>Arora, Rahul; Oxford University, Institute of Biomedical Engineering<br>Kodama, David; Unity Health Toronto, St. Michael's Hospital; University of Toronto Department of Medicine, Division of Emergency Medicine<br>Bobrovitz, Niklas; University of Toronto Temerty Faculty of Medicine; University of Calgary, Department of Critical Care Medicine
**Primary Subject Heading**:	Occupational and environmental medicine
Secondary Subject Heading:	Infectious diseases, Public health
Keywords:	COVID-19, Public health < INFECTIOUS DISEASES, OCCUPATIONAL & INDUSTRIAL MEDICINE

I, the Submitting Author has the right to grant and does grant on behalf of all authors of the Work (as defined in the below author licence), an exclusive licence and/or a non-exclusive licence for contributions from authors who are: i) UK Crown employees; ii) where BMJ has agreed a CC-BY licence shall apply, and/or iii) in accordance with the terms applicable for US Federal Government officers or employees acting as part of their official duties; on a worldwide, perpetual, irrevocable, royalty-free basis to BMJ Publishing Group Ltd ("BMJ") its licensees and where the relevant Journal is co-owned by BMJ to the co-owners of the Journal, to publish the Work in this journal and any other BMJ products and to exploit all rights, as set out in our licence.

The Submitting Author accepts and understands that any supply made under these terms is made by BMJ to the Submitting Author unless you are acting as an employee on behalf of your employer or a postgraduate student of an affiliated institution which is paying any applicable article publishing charge ("APC") for Open Access articles. Where the Submitting Author wishes to make the Work available on an Open Access basis (and intends to pay the relevant APC), the terms of reuse of such Open Access shall be governed by a Creative Commons licence – details of these licences and which Creative Commons licence will apply to this Work are set out in our licence referred to above.

Other than as permitted in any relevant BMJ Author's Self Archiving Policies, I confirm this Work has not been accepted for publication elsewhere, is not being considered for publication elsewhere and does not duplicate material already published. I confirm all authors consent to publication of this Work and authorise the granting of this licence.

For peer review only

Occupation and SARS-CoV-2 seroprevalence studies: a systematic review

Emily Boucher,[1] Christian Cao[1], Sean D'Mello,[2] Nathan Duarte,[3] Claire Donnici[1], Natalie Duarte,[4] Graham Bennett,[5] SeroTracker Consortium, Anil Adisesh,[6-8] Rahul K. Arora,[1,9] David Kodama,[6,10] Niklas Bobrovitz[11,12]

1. Cumming School of Medicine, University of Calgary, Calgary, AB, Canada
2. Faculty of Engineering, University of Waterloo, Waterloo, ON, Canada
3. Faculty of Engineering, McGill University, Montreal, QC, Canada
4. Faculty of Arts and Science, University of Toronto, ON, Canada
5. Department of Economics, Faculty of Arts, McGill University, Montreal, QC, Canada
6. St. Michael's Hospital, Unity Health Toronto, Toronto, ON, Canada
7. Division of Occupational Medicine, Department of Medicine, University of Toronto, Toronto, ON, Canada
8. Canadian Health Solutions, Saint John, NB, Canada
9. Institute of Biomedical Engineering, University of Oxford, Oxford, UK
10. Division of Emergency Medicine, Department of Medicine, University of Toronto, Toronto, ON, Canada
11. Temerty Faculty of Medicine, University of Toronto, Toronto, ON, Canada
12. Department of Critical Care Medicine, University of Calgary, Calgary, AB, Canada

*Correspondence to Emily Boucher, Cumming School of Medicine, University of Calgary, Calgary, AB, Canada; emily.boucher@ucalgary.ca

For peer review only - http://bmjopen.bmj.com/site/about/guidelines.xhtml

Word Count 1411

Key Words Covid-19, Infectious diseases**,** Occupational & industrial medicine

For peer review only

ABSTRACT

Objective. To describe and synthesize studies of SARS-CoV-2 seroprevalence by occupation prior to the widespread vaccine rollout.

Methods. We identified studies of occupational seroprevalence from a living systematic review (PROSPERO CRD42020183634). Electronic databases, gray literature, and news media were searched for studies published January-December 2020. Seroprevalence estimates and a free text description of the occupation were extracted and classified according to the Standard Occupational Classification (SOC) 2010 system using a machine-learning algorithm. Due to heterogeneity, results were synthesized narratively.

Results. We identified 196 studies including 591,940 participants from 38 countries. Most studies (n=162; 83%) were conducted locally vs regionally or nationally. Sample sizes were generally small (median=220 participants per occupation) and 135 studies (69%) were at a high risk of bias. One or more estimates were available for 21/23 major SOC occupation groups, but over half of the estimates identified (n=359/600) were for healthcare-related occupations. 'Personal Care and Service Occupations' (median 22% [IQR 9-28%]; n=14) had the highest median seroprevalence.

Conclusions. Many seroprevalence studies covering a broad range of occupations were published in the first year of the pandemic. Results suggest considerable differences in seroprevalence between occupations, although few large, high-quality studies were done. Well-designed studies are required to improve our understanding of the occupational risk of SARS-CoV-2 and should be considered as an element of pandemic preparedness for future respiratory pathogens.

For peer review only

For peer review only - http://bmjopen.bmj.com/site/about/guidelines.xhtml

Strengths and limitations

- We conducted a comprehensive search of the COVID-19 seroprevalence literature, including non-English articles, government reports, unpublished data.
- Occupations were classified using the Standard Occupational Classification (SOC) 2010 coding system to improve interpretability and facilitate comparison with other datasets.
- Seroprevalence may underestimate the true prevalence of infection because antibody titres decline over time, but where possible we prioritized prevalence estimates for IgG antibodies, which appear to be more robust than other immunoglobulin types.
- We did not adjust for differences in serologic test performance.

For peer review only

INTRODUCTION

Occupation is a social determinant of health and an important risk factor for SARS-CoV-2 infection. Essential workers in health and social care occupations have an increased risk of COVID-19 compared to non-essential workers, but the risks for other occupations are not well defined.[1-3] Studies examining confirmed COVID-19 cases to examine occupational COVID-19 risk are affected by variable testing rates. For example, testing rates may be higher in workplaces offering testing or paid sick leave, and are impacted by geographic (e.g., urban versus rural) and socio-economic factors (e.g., deprivation), potentially biasing results.[4-6] Few high-quality, prospective studies using frequent, serial molecular or antigen testing covering a broad range of occupations having been conducted, in part due to the costs and administrative burden of such studies.[7,8]

Serologic testing for SARS-CoV-2 antibodies provides evidence of previous infection and/or vaccination depending on vaccination status and the specific antigens targeted and can be used to obtain more accurate estimates of the cumulative incidence of infection.[9] Accurate data on the occupational risks of COVID-19 and other respiratory infections are essential for informing the development of occupational safety guidelines and regulations, transmission control measures and resource allocation (testing, personal protective equipment (PPE), etc.). The objectives of this review were to describe and synthesize studies of SARS-CoV-2 seroprevalence across a broad range of occupations globally prior to the widespread rollout of vaccines.

For peer review only

METHODS

We identified seroprevalence studies with sample frames or subgrouping variables related to occupation or employment status from a database compiled via a living systematic review (PROSPERO CRD42020183634). The database has been described previously and includes >1000 cohort and cross-sectional studies reporting antibody testing for SARS-CoV-2 in humans identified from electronic databases, grey literature, and news media.[10-12] We restricted the current review to studies published January-December 2020 before vaccines were rolled-out, because differential vaccination rates by occupation may obscure results. We excluded studies that only reported seroprevalence for mixed occupation groups or workplaces (e.g., "hospital staff") rather than specific occupations, included children <18 years and that could not be machine-translated using Google Translate if unavailable in English or French (**Supplementary File 1**).

We extracted study information, sample characteristics, seroprevalence estimates and study-level risk of bias from the living review database. Risk of bias was assessed with a modified Joanna Briggs Institute Checklist for Prevalence Studies by one reviewer and verified independently as described previously. Overall risk of bias was assessed qualitatively based on whether seroprevalence estimates were very likely (corresponding to a low risk of bias), likely (moderate risk) or unlikely (low risk) to be correct for the author's stated target population (**Supplementary File 1**).[12,13] If multiple estimates were reported, the most recent estimate using laboratory-based methods (e.g. ELISA), and anti-spike and/or IgG antibodies were prioritized, because non-IgG and anti-nucleocapsid antibodies may decline more rapidly.[14] Free-text

For peer review only - http://bmjopen.bmj.com/site/about/guidelines.xhtml

descriptions of occupations were extracted from the original studies by one researcher and reviewed by a second.

For each seroprevalence estimate, we identified the relevant Standard Occupational Classification (SOC) 2010 codes by applying the National Institute for Occupational Safety & Health (NIOSH) Industry and Occupation Computerized Coding System (NIOCCS) to occupation descriptions.[15] NIOCCS was chosen, because many studies were conducted in the USA. Coding was manually verified if there was insufficient information for NIOCCS classification, or if the probability of correct classification to the six-digit level was <0.8 based on our review of a subset of the NIOCCS coded data (**Supplementary File 1**). Anticipating substantial heterogeneity and an insufficient number of estimates relative to covariates for meta-regression, we planned to summarize data using the median/IQR.

Patient and Public Involvement: It was not possible or appropriate to involve patients or the public in this study.

RESULTS

We identified 196 studies of occupational seroprevalence conducted in 2020 during the first and second waves of the pandemic **(Figure 1)**. There were 591,940 participants from 38 countries, including the USA (n=44 studies), UK (n=16) and Italy (n=15). Most studies (n=162; 83%) were conducted locally (e.g. city, county) as opposed to regionally (e.g. state; n=20; 10%) or nationally (n=14; 7%). Most were restricted to one occupational group (n=103), limiting direct comparisons (i.e. using the same reference group). Sample sizes were often small (median=220, IQR 64-568 participants). Overall, 135 studies (69%) were at a high risk of bias, 47 moderate

For peer review only - http://bmjopen.bmj.com/site/about/guidelines.xhtml

(24%), 2 low (1%) and 12 unclear (6%). Common reasons for bias were inadequate statistical analysis (i.e. no adjustment for test or sample characteristics; 92%), non-probability sampling (74%), and small sample-size (46%).

At least one estimate was available for all 23 major SOC occupation groups, except for ‘Legal’ and ‘Military-Specific’ occupations **(Figure 2**; all studies**)**. Over half of the 600 estimates identified (n=359) were for healthcare-related occupations. For SOC groups with three or more estimates, the highest median seroprevalence was reported for ‘Personal Care and Service Occupations’ (median 22% [IQR 9-28%]; n=14, e.g. ‘Personal Care Aids’). The next highest was reported for ‘Building and Grounds Cleaning and Maintenance’ occupations (11% [3-22%]; n=17, e.g. ‘Maids and Housekeeping Cleaners’), and ‘Healthcare Support’ (11% [2-20%]; n=39, e.g. ‘Nursing Assistants’) occupations. The lowest median seroprevalence was 1% (0-11%; n=6, e.g. ‘Athletes’) for ‘Arts, Design, Entertainment, Sports, and Media Occupations.’ Individual estimates are listed in **Supplementary File 2**.

DISCUSSION

This review is the first comprehensive synthesis of occupational COVID-19 seroprevalence studies world-wide. We identified 196 studies representing 21 out of 23 major SOC groups conducted during the first and second waves of the SARS-CoV-2 pandemic in 2020, prior to the widespread rollout of vaccines, and described occupational groups with high seroprevalence.

Seroprevalence studies may estimate the cumulative incidence of infection more accurately than diagnostic testing studies when access to testing and test performance are poor, and also can

For peer review only - http://bmjopen.bmj.com/site/about/guidelines.xhtml

identify asymptomatic infections.[6,8] The data identified suggest considerable differences in seroprevalence by occupation, though we did not statistically test for differences due to considerable variation in geography, study dates and workplace determinants of infection (e.g. PPE, ventilation). 'Caring and Personal Service' occupations had the highest median seroprevalence (22%), which was four-times higher than the unemployed (5%) and median seroprevalence across all occupational groups (5%). The UK Office for National Statistics reported a slightly lower cumulative incidence for positive diagnostic or rapid tests for COVID-19 across 25 occupational groups of 4% (mean),[4] but the discrepancy between the true cumulative incidence and confirmed infections is likely greater in regions with less access to testing: national, population-based serosurveys have estimated there are 10-20 serologically identifiable cases per one confirmed case.[12]

In future pandemics, large, well-reported, high-quality seroprevalence studies across a broad range of occupations are needed at an early stage to inform appropriate workplace policy. It has been suggested that 20% of the US workforce was exposed to disease or infection at work at least once a month prior to the pandemic.[16] Accurate data on the occupational risks of respiratory infections, including SARS-CoV-2 are needed to inform understanding of transmission, occupational health and safety agency guidelines and allocation of resources (e.g., personal protective equipment and vaccines) during outbreaks and pandemics. For governments, there are also issues of occupational disease recognition and compensation to be considered.

As such, future population-based studies on respiratory infections should collect data on occupation. In the case of epidemic infection, collaboration between academic centres with the

For peer review only - http://bmjopen.bmj.com/site/about/guidelines.xhtml

capacity to conduct large-scale studies and government agencies with expertise in disease surveillance and access to workplace data (e.g., public health, occupational health and safety) may be beneficial.[12] Other authors have suggested the utility of occupational surveillance systems.[17] However, the routine completion of the occupation field in electronic health records would also serve this purpose as well as informing patient reported outcome measures.

Strengths and Limitations

Despite the large number of studies of occupational seroprevalence conducted, many studies had methodological limitations. Only two studies were at a low risk of bias and most occupational subgroups had small sample sizes (median 220 participants). Many were limited to one major SOC group (n=103 studies), which precluded comparisons. Detailed descriptions of occupations were often lacking, potentially contributing to coding errors and misclassification, and workplace determinants of infection (e.g. use of PPE) were poorly reported.

In conclusion, our review shows that a large number of seroprevalence studies covering a broad range of occupations were published in the first year of the pandemic. Results suggest considerable differences in seroprevalence between occupations, although few large, well-reported, high-quality studies were done. Carefully-designed, adequately powered seroprevalence studies with coverage of a broad range of occupations could improve our understanding of the occupational risk of SARS-CoV-2 and other respiratory infections and should be considered an element of pandemic preparedness and response.

For peer review only

Funding Statement

SeroTracker receives funding for SARS-CoV-2 seroprevalence study evidence synthesis from the Public Health Agency of Canada through Canada's COVID-19 Immunity Task Force (Grant Number 2021-HQ-000056), the World Health Organization Health Emergencies Programme, the Robert Koch Institute, and the Canadian Medical Association Joule Innovation Fund. No funding source had any role in the design of this study, its execution, analyses, interpretation of the data, or decision to submit results. This manuscript does not necessarily reflect the views of the World Health Organization or any other funder.

Statement of author's contributions

This secondary analysis of the SeroTracker database was conceived by NB, EB, DK and AA. Senior authors on this paper were NB, DK, RA and AA. The protocol was developed by EB, NB and DK. Data cleaning was performed by CC, CD, NatalieD, SD and EB and verification by EB, SD, NathanD and GB. Analysis was performed by EB and RA. The first draft of the manuscript was written by EB and revised by EB, RA, NB, NathanD, GB, SD, CC, AA, DK. The SeroTracker Consortium maintained the living systematic review database used in the study. All authors reviewed and agreed to the findings, and also provided critical revisions to the paper.

Disclosure of potential and actual conflicts of interest

RKA was previously a Technical Consultant for the Bill and Melinda Gates Foundation Strategic Investment Fund, is a minority shareholder of Alethea Medical, and was a former Senior Policy Advisor at Health Canada. Each of these relationships is unrelated to the present work.

For peer review only - http://bmjopen.bmj.com/site/about/guidelines.xhtml

JP reports grants to his institution from MedImmune, Sanofi Pasteur, Merck and AbbVie, and personal fees for lectures from AbbVie and Astra-Zeneca, all outside of the submitted work.

MPC reports grants from McGill Interdisciplinary Initiative in Infection and Immunity, grants from Canadian Institutes of Health Research, during the conduct of the study; personal fees from GEn1E Lifesciences, personal fees from nplex biosciences, personal fees from Kanvas biosciences, personal fees from AstraZeneca, non-financial support from Cidara therapeutics, non-financial support from Scynexis, Inc., non-financial support from Amplyx Pharmaceutics, outside the submitted work. In addition, MPC has a patent for methods detecting tissue damage, graft versus host disease, and infections using cell-free DNA profiling pending, a patent for methods assessing the severity and progression of SARS-CoV-2 infections using cell-free DNA pending, a patent for rapid identification of antimicrobial resistance and other microbial phenotypes using highly-multiplexed fluorescence in situ hybridization pending, and a patent highly multiplexed detection of gene expression with hybridization chain reaction pending, all outside the submitted work.

Ethics approval: Not applicable. This study did not involve human participants or animals.

Dating sharing: Seroprevalence data can be downloaded (or requested) from https://serotracker.com.

For peer review only

For peer review only - http://bmjopen.bmj.com/site/about/guidelines.xhtml

REFERENCES

1. Magnusson K, Nygard KM, Methi F, Vold L, Telle K. Occupational risk of COVID-19 in the first versus second epidemic wave in Norway, 2020. *Euro Surveill* 2021;26:2001875.

2. Mutambudzi M, Niedwiedz C, Macdonald EB, et al. Occupation and risk of severe COVID-19: prospective cohort study of 120 075 UK Biobank participants. *Occup Environ Med* 2021;78:307-14.

3. Nguyen LH, Drew DA, Graham MS, et al. Risk of COVID-19 among front-line health-care workers and the general community: a prospective cohort study. *Lancet Public Health* 2020;5:e475-83.

4. Seo E, Mun E, Kim W, Lee C. Fighting the COVID-19 pandemic: onsite mass workplace testing for COVID-19 in the Republic of Korea. *Ann Occup Environ Med* 2020;32:e22.

5. Tan TQ, Kullar R, Swartz TH, Mathew TA, Piggott DA, Berthaud V. Location matters: geographic disparities and impact of coronavirus disease 2019. *J Infect Dis* 2020;222:1951-4.

6. Duarte N, D'Mello S, Duarte NA, et al. Uptake of SARS-CoV-2 workplace testing programs, March 2020 to March 2021. *MedRxiv* 21259730 [Preprint]. July 31 2021 [cited December 28 2021] doi: https://doi.org/10.1101/2021.06.29.21259730

7. Office for National Statistics. Coronavirus (COVID-19) Infection Survey: characteristics of people testing positive for COVID-19 in England. 2021 Feb 22. Available from: https://www.ons.gov.uk/peoplepopulationandcommunity/healthandsocialcare/conditionsanddiseases/articles/coronaviruscovid19infectionsinthecommunityinengland/characteristicsofpeopletestingpositiveforcovid19inengland22february2021

8. Pearce N, Rhodes S, Stocking K, et al. Occupational differences in COVID-19 incidence, severity, and mortality in the United Kingdom: Available data and framework for analyses. *Wellcome Open Res* 2021;6:102.

9. Duarte N, Yanes-Lane M, Arora RK, et al. Adapting Serosurveys for the SARS-CoV-2 Vaccine Era. *Open Forum Infect Dis* 2021;9:ofab632.

10. Arora RK, Joseph A, Van Wyk J, et al. SeroTracker: a global SARS-CoV-2 seroprevalence dashboard. *Lancet Infec Dis* 2021;21:e75–6.

[dataset] 11. SeroTracker Consortium. Data from: Our Data. November 7, 2021. https://serotracker.com/data

For peer review only - http://bmjopen.bmj.com/site/about/guidelines.xhtml

12. Bobrovitz N, Arora RK, Cao C, et al. Global seroprevalence of SARS-CoV-2 antibodies: a systematic review and meta-analysis. *PloS ONE* 2021;16:e0252617.

13. Munn Z, Moola S, Lisy K, Riitano D, Tufanaru C. Methodological guidance for systematic reviews of observational epidemiological studies reporting prevalence and incidence data. *Int J Evid Based Healthc* 2015;13:147–153.

14. Isho B, Abe KT, Zuo M, et al. Persistence of serum and saliva antibody responses to SARS-CoV-2 spike antigens in COVID-19 patients. *Sci Immunol* 2020;5:eabe5511.

15. NIOSH. NIOSH Industry and Occupation Computerized Coding System (NIOCCS). U.S. Department of Health and Human Services, Public Health Service, Centers for Disease Control and Prevention, National Institute for Occupational Safety and Health, Division of Field Studies & Engineering, Health Informatics Branch. https://csams.cdc.gov/nioccs/About.aspx. Date accessed Sept 1, 2021.

16. Baker MG, Peckham TK, Seixas NS. Estimating the burden of United States workers exposed to infection or disease: a key factor in containing risk of COVID-19 infection. *PloS ONE* 2020;15:e0232452.

17. Marinaccio A, Boccuni F, Rondinone BM, Brusco A, D'Amario S, Iavicoli S. Occupational factors in the COVID-19 pandemic in Italy: compensation claims applications support establishing an occupational surveillance system. *Occup Environ Med* 2020;77:818-21.

For peer review only

Figure Legends

Figure 1. PRISMA flow diagram

Figure 2. Seroprevalence by SOC 2010 major occupation group. *Estimates are a mix of 'Healthcare Practitioners and Technical Occupations' and 'Healthcare Support Occupations' (see next page)

Supplementary File 1. Supplementary methods

Supplementary File 2. Summary of included studies and references

For peer review only

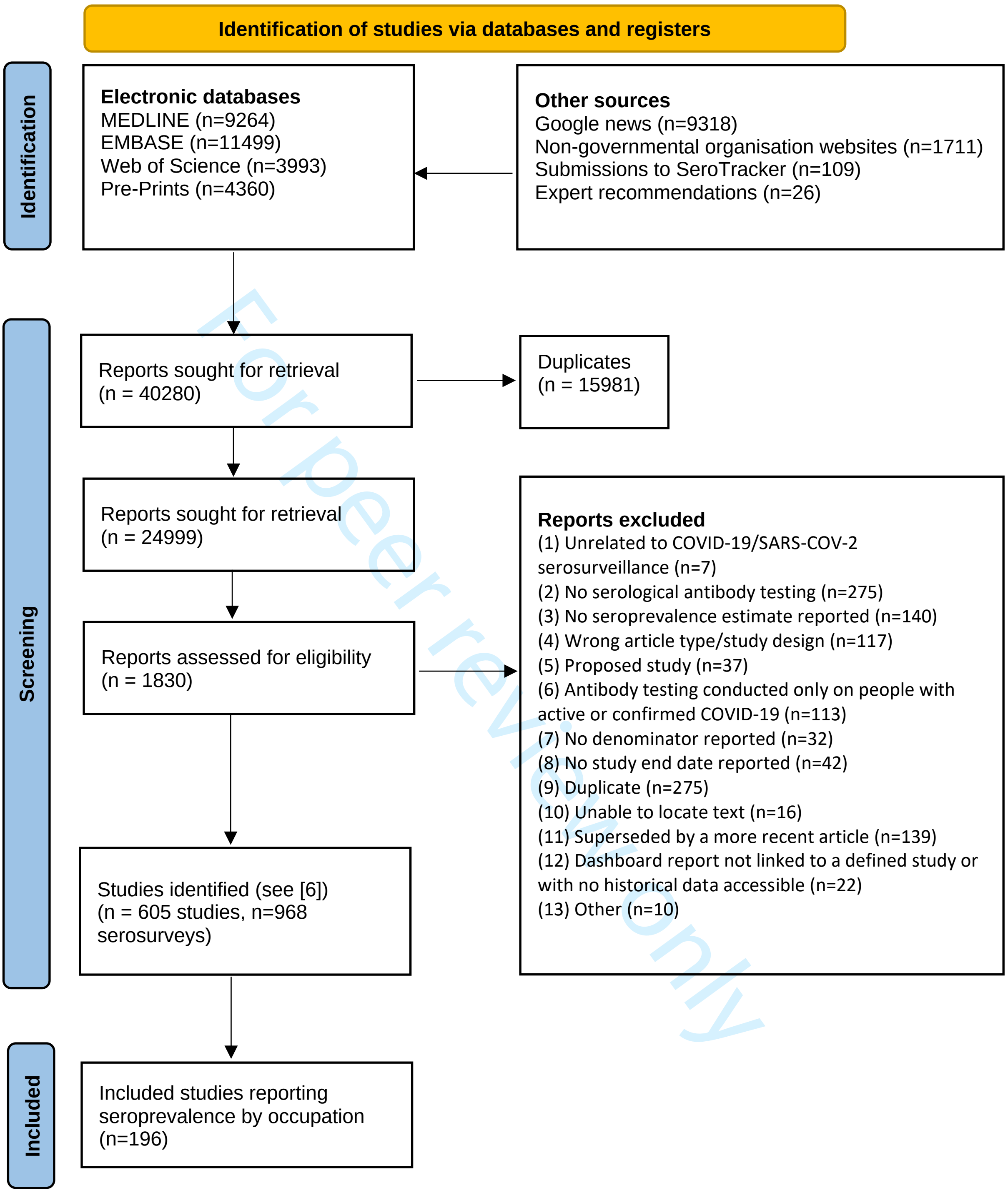

For peer review only

From: Page MJ, McKenzie JE, Bossuyt PM, Boutron I, Hoffmann TC, Mulrow CD, et al. The PRISMA 2020 statement: an updated guideline for reporting systematic reviews. BMJ 2021;372:n71. doi: 10.1136/bmj.n71

For more information, visit: http://www.prisma-statement.org/

SOC 2010 Major Occupation Group	Total		Median, IQR		Seroprevalence %		N, %
	Estimates	Countries	Study dates, midpoint	Sample size	(Median, IQR)	(Scale 0-75%)	Low-Moderate RoB
Architecture and Engineering Occupations (17-0000)	1	1	15/08 (15/08-15/08)	21 (21-21)	42.9 (42.9-42.9)		0 (0%)
Personal Care and Service Occupations (39-0000)	14	7	03/05 (02/04-02/06)	127 (54-302)	21.5 (9.32-27.76)		3 (21%)
Installation, Maintenance, and Repair Occupations (49-0000)	1	1	19/06 (19/06-19/06)	134 (134-134)	16.4 (16.4-16.4)		0 (0%)
Building and Grounds Cleaning and Maintenance Occupations (37-0000)	17	8	13/07 (09/06-16/08)	102 (42-226)	10.8 (3.3-21.7)		6 (35%)
Healthcare Support Occupations (31-0000)	39	12	05/06 (19/05-21/06)	263 (122-562)	10.7 (2-20.05)		12 (31%)
Business and Financial Operations Occupations (13-0000)	2	2	05/07 (18/06-22/07)	462 (252-671)	8.27 (5.3-11.23)		2 (100%)
Management Occupations (11-0000)	10	6	17/06 (01/05-02/08)	44 (23-145)	8.17 (6.7-19.93)		3 (30%)
Food Preparation and Serving Related Occupations (35-0000)	6	4	17/06 (11/05-23/07)	58 (12-108)	6.35 (2.37-24.03)		2 (33%)
Healthcare Practitioners and Technical Occupations (29-0000)	222	23	13/06 (13/05-13/07)	215 (64-482)	5.91 (1.83-11.71)		84 (38%)
*Healthcare Practitioners and Technical Occupations, 5-digit codes***							
Miscellaneous Health Technologists and Technicians	4	3	26/08 (09/08-12/09)	60 (20-121)	12.96 (9.09-27.54)		1 (25%)
Registered Nurses	78	18	05/06 (05/05-05/07)	329 (71-1000)	8.44 (3.68-15.5)		22 (28%)
Clinical Laboratory Technologists and Technicians	18	12	15/06 (19/05-11/07)	204 (86-284)	6.22 (2.07-11.94)		12 (67%)
Physicians and Surgeons	65	21	09/06 (10/05-09/07)	214 (59-564)	5.88 (1.85-11.8)		23 (35%)
Emergency Medical Technicians and Paramedics	9	6	13/06 (27/05-30/06)	157 (56-243)	5.41 (5.2-11)		4 (44%)
Therapists	15	4	08/06 (19/05-28/06)	121 (61-235)	3.75 (0-9.45)		7 (47%)
Physician Assistants	9	2	27/06 (26/05-28/07)	230 (156-320)	3.48 (0.64-9.43)		3 (33%)
Pharmacists	9	7	29/06 (14/06-14/07)	113 (29-213)	0.5 (0-3.45)		4 (44%)
Healthcare Occupations (mixed)*	94	25	05/06 (29/04-12/07)	375 (110-1012)	5.66 (2.35-11.6)		23 (24%)
Sales and Related Occupations (41-0000)	23	8	21/08 (22/06-19/10)	643 (236-1184)	5.3 (1.2-8.8)		6 (26%)
Education, Training, and Library Occupations (25-0000)	6	5	05/07 (12/06-27/07)	238 (73-1305)	5.07 (2.71-17.22)		3 (50%)
Farming, Fishing, and Forestry Occupations (45-0000)	3	3	13/07 (25/06-30/07)	80 (66-100)	5 (2.5-5)		1 (33%)
Not employed (mixed)*	37	14	23/06 (12/05-04/08)	382 (116-905)	4.9 (2.7-14.97)		28 (76%)
Office and Administrative Support Occupations (43-0000)	39	18	14/06 (18/05-11/07)	120 (32-522)	4.88 (1.36-13.36)		20 (51%)
First responders (mixed)*	6	1	18/05 (13/05-22/05)	219 (72-599)	4.67 (1.6-7.34)		1 (17%)
Community and Social Service Occupations (21-0000)	6	2	30/05 (18/05-11/06)	104 (49-188)	4.45 (2.13-6.1)		1 (17%)
Protective Service Occupations (33-0000)	28	9	04/07 (21/05-16/08)	190 (46-555)	4.29 (2.17-7.47)		6 (21%)
Transportation and Material Moving Occupations (53-0000)	23	7	08/08 (08/06-08/10)	230 (80-364)	3.5 (1.8-11.8)		8 (35%)
Life, Physical, and Social Science Occupations (19-0000)	11	7	06/07 (11/06-30/07)	343 (174-570)	2.6 (1.66-6.46)		4 (36%)
Production Occupations (51-0000)	4	3	23/05 (26/04-19/06)	764 (342-1132)	1.52 (1.45-4.93)		2 (50%)
Arts, Design, Entertainment, Sports, and Media Occupations (27-0000)	6	5	07/07 (04/06-09/08)	164 (47-823)	1.39 (0.18-11.02)		3 (50%)
Computer and Mathematical Occupations (15-0000)	1	1	03/05 (03/05-03/05)	47 (47-47)	0 (0-0)		1 (100%)
Construction and Extraction Occupations (47-0000)	1	1	03/05 (03/05-03/05)	42 (42-42)	0 (0-0)		1 (100%)

For peer review only - http://bmjopen.bmj.com/site/about/guidelines.xhtml

Supplementary File 1 Materials

Table of Contents

Supplementary files ... 2
S1. PRISMA checklist ... 2
S2. Search strategy ... 4
S3. Detailed eligibility criteria ... 8
S4. Tool for assessing study risk of bias ... 9
S5. Details of occupational coding ... 12
References for supplementary files ... 14

For peer review only

Supplementary Material

S1. PRISMA checklist

Section/topic	#	Checklist item	Reported on page #
**TITLE**			
Title	1	Identify the report as a systematic review, meta-analysis, or both.	0
**ABSTRACT**			
Structured summary	2	Provide a structured summary including, as applicable: background; objectives; data sources; study eligibility criteria, participants, and interventions; study appraisal and synthesis methods; results; limitations; conclusions and implications of key findings; systematic review registration number.	1
**INTRODUCTION**			
Rationale	3	Describe the rationale for the review in the context of what is already known.	3, lines 14-30
Objectives	4	Provide an explicit statement of questions being addressed with reference to participants, interventions, comparisons, outcomes, and study design (PICOS).	3, line 30-32
**METHODS**			
Protocol and registration	5	Indicate if a review protocol exists, if and where it can be accessed (e.g., Web address), and, if available, provide registration information including registration number.	3, line 39
Eligibility criteria	6	Specify study characteristics (e.g., PICOS, length of follow-up) and report characteristics (e.g., years considered, language, publication status) used as criteria for eligibility, giving rationale.	4, lines 39-45
Information sources	7	Describe all information sources (e.g., databases with dates of coverage, contact with study authors to identify additional studies) in the search and date last searched.	4, lines 39-40
Search	8	Present full electronic search strategy for at least one database, including any limits used, such that it could be repeated.	Suppl. File 2
Study selection	9	State the process for selecting studies (i.e., screening, eligibility, included in systematic review, and, if applicable, included in the meta-analysis).	4, lines 41-43
Data collection process	10	Describe method of data extraction from reports (e.g., piloted forms, independently, in duplicate) and any processes for obtaining and confirming data from investigators.	4, lines 41-49, 57-58
Data items	11	List and define all variables for which data were sought (e.g., PICOS, funding sources) and any assumptions and simplifications made.	4, lines 44-45 (see reference to previous study)
Risk of bias in individual studies	12	Describe methods used for assessing risk of bias of individual studies (including specification of whether this was done at the study or outcome level), and how this information is to be used in any data synthesis.	4, see reference and Suppl. File 1
Summary measures	13	State the principal summary measures (e.g., risk ratio, difference in means).	4, lines 57-78
Synthesis of results	14	Describe the methods of handling data and combining results of studies, if done, including measures of consistency (e.g., I^2) for each meta-analysis.	4, lines 57-58
Risk of bias across studies	15	Specify any assessment of risk of bias that may affect the cumulative evidence (e.g., publication bias, selective reporting within studies).	4, lines 47-48
Additional analyses	16	Describe methods of additional analyses (e.g., sensitivity or subgroup analyses, meta-regression), if done, indicating which were pre-specified.	NA
**RESULTS**			
Study selection	17	Give numbers of studies screened, assessed for eligibility, and included in the review, with reasons for exclusions at each stage, ideally with a flow diagram.	Suppl File 1
Study characteristics	18	For each study, present characteristics for which data were extracted (e.g., study size, PICOS, follow-up period) and provide the citations.	Suppl. File 2
Risk of bias within studies	19	Present data on risk of bias of each study and, if available, any outcome level assessment (see item 12).	Suppl. File 2
Results of individual studies	20	For all outcomes considered (benefits or harms), present, for each study: (a) simple summary data for each intervention group (b) effect estimates and confidence intervals, ideally with a forest plot.	Suppl. File 2
Synthesis of results	21	Present results of each meta-analysis done, including confidence intervals and measures of consistency.	NA – see narrative synthesis on page 5 & Figure 1
Risk of bias across studies	22	Present results of any assessment of risk of bias across studies (see Item 15).	5, lines 72-75 Figure 1
Additional analysis	23	Give results of additional analyses, if done (e.g., sensitivity or subgroup analyses, meta-regression [see Item 16]).	NA
**DISCUSSION**			
Summary of evidence	24	Summarize the main findings including the strength of evidence for each main outcome; consider their relevance to key groups (e.g., healthcare providers, users, and	6, lines 110-118

For peer review only - http://bmjopen.bmj.com/site/about/guidelines.xhtml

		policy makers).	
Limitations	25	Discuss limitations at study and outcome level (e.g., risk of bias), and at review-level (e.g., incomplete retrieval of identified research, reporting bias).	6, lines 131-136
Conclusions	26	Provide a general interpretation of the results in the context of other evidence, and implications for future research.	6, lines 119-120
**FUNDING**			
Funding	27	Describe sources of funding for the systematic review and other support (e.g., supply of data); role of funders for the systematic review.	9

16

For peer review only

For peer review only - http://bmjopen.bmj.com/site/about/guidelines.xhtml

S2. Search strategy

Database: Ovid MEDLINE(R) and Epub Ahead of Print, In-Process & Other Non-Indexed Citations and Daily
Dates: January 1, 2020 to December 31, 2020
Notes: Covid-19 search terms were adapted from Ovid Expert Searches

#	Search terms
1	exp Coronavirus/
2	exp Coronavirus Infections/
3	(coronavirus* or corona virus* or OC43 or NL63 or 229E or HKU1 or HCoV* or ncov* or covid* or sars-cov* or sarscov* or Sars-coronavirus* or Severe Acute Respiratory Syndrome Coronavirus*).tw,kf.[EB2]
4	or/1-3
5	4 not ((MERS or MERS-CoV or Middle East respiratory syndrome or camel* or dromedar* or equine or coronary or coronal or covidence* or covidien or influenza virus or HIV or bovine or calves or TGEV or feline or porcine or BCoV or PED or PEDV or PDCoV or FIPV or FCoV or SADS-CoV or canine or CCov or zoonotic or avian influenza or H1N1 or H5N1 or H5N6 or IBV).mp. or (animals/ not humans/))
6	((pneumonia or covid* or coronavirus* or corona virus* or ncov* or 2019-ncov or sars* or virus).tw,kf. or exp pneumonia/) and Wuhan.tw,kf.
7	(2019-ncov* or 2019nCov* or ncov19 or ncov-19 or 2019-novel CoV or sars-cov2* or sars-cov-2* or sarscov2* or sarscov-2* or Sars-coronavirus2 or Sars-coronavirus-2 or SARS-like coronavirus* or coronavirus 2 or coronavirus2* or corona or coronavirus-19 or covid19 or covid-19 or covid 2019 or ((novel or new or nouveau) adj2 (CoV or nCoV or covid or coronavirus* or corona virus or Pandemi*2)) or ((covid or covid19* or covid-19) and pandemic*2) or (coronavirus* and pneumonia)).tw,kf.
8	COVID-19.rx,px,ox. or severe acute respiratory syndrome coronavirus 2.os.
9	or/6-8
10	5 or 9
11	immunoglobulins/ or antibodies/ or antibodies, blocking/ or exp antibodies, neutralizing/ or antibodies, viral/ or antigen-antibody complex/ or immune sera/ or exp immunoglobulin isotypes/ or immunoglobulin a/ or immunoglobulin d/ or immunoglobulin e/ or immunoglobulin g/ or immunoglobulin m/
12	serologic tests/ or complement fixation tests/ or hemagglutination inhibition tests/ or neutralization tests/
13	immunoassay/ or fluoroimmunoassay/ or exp immunoblotting/ or immunoenzyme techniques/ or exp enzyme-linked immunosorbent assay/ or exp enzyme-linked immunospot assay/ or immunosorbent techniques/ or serologic tests/ or complement fixation tests/ or hemagglutination inhibition tests/ or neutralization tests/ or Serology/di
14	(enzyme linked immunosorbent or enzyme-linked immunosorbent or ELISA or immunofluorescence or complement fixation or hemagglutination inhibition or immunoblot or western blot or neutrali*).tw,kf.
15	(antibod* or immunoglobulin* or immune globulin* or titer* or isotype* or IgG or IgM or IgA or neutrali* or sera or serum or serolog* or saliva).tw,kf.
16	or/11-14
17	seroepidemiologic studies/
18	incidence/ or prevalence/
19	(seroconver* or seroprevalence or sero-prevalence or seroincidence or sero-incidence or seroepidemiolog* or sero-epidemiolog*).mp.
20	(inciden* or prevalen* or count* or rate*).mp.
21	(serosurvey or sero-survey or screen* or diagnostic).mp.
22	(seroconver* or seroprevalence or sero-prevalence or seroincidence or sero-incidence or seroepidemiolog* or sero-epidemiolog* or inciden* or prevalen* or silent or asymptomatic or serosurvey or sero-survey).tw,kf.
23	or/17-21
24	10 and (16 and 23)
25	10 and 15
26	10 and 22
27	or/24-26
28	limit 27 to yr="2020-Current"
29	remove duplicates from 28

For peer review only - http://bmjopen.bmj.com/site/about/guidelines.xhtml

Database: Embase
Dates: January 1, 2020 to December 31, 2020
Notes: Covid-19 search terms were adapted from Ovid Expert Searches

#	Searches
1	exp Coronavirus/
2	exp Coronavirus Infections/
3	(coronavirus* or corona virus* or OC43 or NL63 or 229E or HKU1 or HCoV* or ncov* or covid* or sars-cov* or sarscov* or Sars-coronavirus* or Severe Acute Respiratory Syndrome Coronavirus*).tw,kw.
4	or/1-3
5	4 not ((MERS or MERS-CoV or Middle East respiratory syndrome or camel* or dromedar* or equine or coronary or coronal or covidence* or covidien or influenza virus or HIV or bovine or calves or TGEV or feline or porcine or BCoV or PED or PEDV or PDCoV or FIPV or FCoV or SADS-CoV or canine or CCov or zoonotic or avian influenza or H1N1 or H5N1 or H5N6 or IBV).mp. or (animals/ not humans/))
6	((pneumonia or covid* or coronavirus* or corona virus* or ncov* or 2019-ncov or sars*).tw,kw. or exp pneumonia/) and Wuhan.tw,kw.
7	(2019-ncov or ncov19 or ncov-19 or 2019-novel CoV or sars-cov2 or sars-cov-2 or sarscov2 or sarscov-2 or Sars-coronavirus2 or Sars-coronavirus-2 or SARS-like coronavirus* or coronavirus-19 or covid19 or covid-19 or covid 2019 or ((novel or new or nouveau) adj2 (CoV or nCoV or covid or coronavirus* or corona virus or Pandemi*2)) or ((covid or covid19 or covid-19) and pandemic*2) or (coronavirus* and pneumonia)).tw,kw.
8	(coronavirus disease 2019 or severe acute respiratory syndrome coronavirus 2).sh,dj.
9	6 or 7 or 8
10	5 or 9
11	virus antibody/ec [Endogenous Compound]
12	neutralizing antibody/ec [Endogenous Compound]
13	exp immunoglobulin/ or exp immunoglobulin A antibody/ or exp immunoglobulin class/ or exp immunoglobulin M antibody/ or exp immunoglobulin G antibody/ or exp immunoglobulin antibody/
14	11 or 12 or 13
15	serology/
16	serodiagnosis/ or complement fixation test/ or hemagglutination inhibition test/ or hemolytic plaque assay/
17	fluorescent antibody technique/
18	immunofluorescence test/ or viral disease immunofluorescence assay/
19	enzyme linked immunosorbent assay/
20	western blotting/
21	(enzyme linked immunosorbent or enzyme-linked immunosorbent or ELISA or immunoassay or immunofluorescence or fluorescent antibody or complement fixation or hemagglutination inhibition or hemolytic plaque assay or immunoblot or western blot or neutrali*).tw,kw.
22	(antibod* or immunoglobulin* or immune globulin* or titer* or isotype* or IgG or IgM or IgA or neutrali* or sera or serolog* or serum or saliva).tw,kw.
23	15 or 16 or 17 or 18 or 19 or 20 or 21
24	14 or 23
25	exp seroepidemiology/
26	*prevalence/
27	*incidence/
28	(seroconver* or seroprevalence or sero-prevalence or seroincidence or sero-incidence or seroepidemiolog* or sero-epidemiolog* or inciden* or prevalen* or count* or rate* or serosurvey or sero-survey or screen* or diagnostic).mp.
29	(seroconver* or seroprevalence or sero-prevalence or seroincidence or sero-incidence or seroepidemiolog* or sero-epidemiolog* or inciden* or prevalen* or silent or asymptomatic or serosurvey or sero-survey).tw,kw.
30	25 or 26 or 27 or 28
31	10 and (24 and 30)
32	10 and 22
33	10 and 29
34	31 or 32 or 33
35	limit 34 to yr="2020-Current"
36	remove duplicates from 35

For peer review only

Database: Web of Science Core Collection
Date: January 1, 2020 to December 31, 2020

#	Searches
1	TS=(coronavirus* or corona virus* or OC43 or NL63 or 229E or HKU1 or HCoV* or ncov* or covid* or sars-cov* or sarscov* or Sars-coronavirus* or Severe Acute Respiratory Syndrome Coronavirus*)
2	TS=(MERS or MERS-CoV or Middle East respiratory syndrome or camel* or dromedar* or equine or coronary or coronal or covidence* or covidien or influenza virus or HIV or bovine or calves or TGEV or feline or porcine or BCoV or PED or PEDV or PDCoV or FIPV or FCoV or SADS-CoV or canine or CCov or zoonotic or avian influenza or H1N1 or H5N1 or H5N6 or IBV)
3	#1 NOT #2
4	TS=((pneumonia or covid* or coronavirus* or corona virus* or ncov* or 2019-ncov or sars* or virus) AND Wuhan)
5	TS=(2019-ncov* or 2019nCov* or ncov19 or ncov-19 or 2019-novel CoV or sars-cov2* or sars-cov-2* or sarscov2* or sarscov-2* or Sars-coronavirus2 or Sars-coronavirus-2 or SARS-like coronavirus* or corona or coronavirus-19 or covid19 or covid-19 or covid 2019 or ((novel or new or nouveau) adj2 (CoV or nCoV or covid or coronavirus*)) or (coronavirus* and pneumonia)).
6	TS=(COVID-19 or "severe acute respiratory syndrome coronavirus")
7	#6 OR #5 OR #4 OR #3
8	TS=(antibod* or immunoglobulin* or immune globulin* or titer* or isotype* or IgG or IgM or IgA or neutralization or sera or serolog* or saliva or serum).
9	TS=("enzyme linked immunosorbent assay" or "enzyme-linked immunosorbent assay" or "immunoenzyme" or ELISA or "lateral flow immunoassay" or LFIA or "immunofluorescence assay" or immunochromatography or "complement fixation test" or "hemagglutination inhibition" or immunoblot or "western blot" or "neutralization assay")
10	#9 OR #8
11	TI=(seroconversion or seroprevalence or seroincidence or seroepidemiolog* or incidence or prevalence or asymptomatic or sero-survey*) or AK=(seroconversion or seroprevalence or seroincidence or seroepidemiolog* or incidence or prevalence or asymptomatic or sero-survey*)
12	ALL=(prevalence or incidence or seroconversion or seroconvert or seroprevalence or seroincidence or seroepidemiolog* or serosurvey or sero-survey or survey or screen* or diagnostic test)
13	#12 AND #10 AND #7
14	#11 AND #7
15	TI=(antibod* or immunoglobulin* or immune globulin* or titer* or isotype* or IgG or IgM or IgA or neutralization or sera or serolog* or saliva or serum).
16	#15 AND #7
17	#16 OR #14 OR #13

Database: Europe PMC [Secondary search for pre-prints]
Dates: January 1, 2020 to December 31, 2020

#	Searches
	("2019-nCoV" OR "2019nCoV" OR "COVID-19" OR "SARS-CoV-2" OR "COVID19" OR "COVID" OR "SARS-nCoV" OR ("wuhan" AND "coronavirus") OR "Coronavirus" OR "Corona virus" OR "corona-virus" OR "corona viruses" OR "coronaviruses" OR "SARS-CoV" OR "Severe Acute Respiratory Syndrome Coronavirus" OR ("SARS" AND "coronavirus")) AND ABSTRACT:(sera* OR sero* OR immun* OR Ig* OR “enzyme-linked immunosorbent assay” OR ELISA OR “neutralization assay" OR seroprevalence) AND (SRC:"PPR")

For peer review only - http://bmjopen.bmj.com/site/about/guidelines.xhtml

Sources: Health organizations
Dates: January 1, 2020 to December 31, 2020

Source		Search strategy
WHO Situation Reports	1	“antibod”, “sero”, “immun”, “ELISA”
National Institutes of Health	1	("COVID" OR "SARS-CoV-2")
	2	("sero*" OR "antibod*" OR "immun*" OR "RDT" OR "ELISA" OR "LFIA")
	3	allintext:(1 AND 2) site:nih.gov -site:ncbi.nlm.nih.gov
	3	2 AND 3
United States Centres for Disease Control and Prevention	1	("COVID" OR "SARS-CoV-2")
	2	("sero*" OR "antibod*" OR "immun*" OR "RDT" OR "ELISA" OR "LFIA")
	3	allintext:(1 AND 2) site:cdc.gov
	5	2 AND 3
European Centres for Disease Control and Prevention	1	("COVID" OR "SARS-CoV-2")
	2	("sero*" OR "antibod*" OR "immun*" OR "RDT" OR "ELISA" OR "LFIA")
	3	allintext:(1 AND 2) site:ecdc.europa.eu
	5	2 AND 3

Sources: Google News
Dates: January 1, 2020 to December 31, 2020

Source		Search strategy
Google news	1	(antibody OR antibodies OR surveillance OR screen OR serology OR serological OR serosurvey OR ELISA OR LFIA OR assay OR blood OR serum OR immune OR immunity OR herd immunity OR random test)

For peer review only

For peer review only - http://bmjopen.bmj.com/site/about/guidelines.xhtml

S3. Detailed eligibility criteria

This study included eligible studies from the SeroTracker database. Eligibility criteria for the database and also for this review specifically are outlined below:

Eligibility criteria for inclusion in SeroTracker database	Eligibility criteria for inclusion in this review
Study performed serologic testing to determine the prevalence of SARS-CoV-2 antibodies in a human population over a specified time period.	Studies included in the SeroTracker database (https://serotracker.com) with relevant subgrouping (i.e., "Occupation," or "Employment status") and/or sample frame variables (i.e., "Healthcare workers and caregivers," "Non-essential workers and unemployed persons," "Essential non-healthcare workers," or "Multiple populations") variables. We also manually searched for potentially relevant studies not falling into these categories.
Reported sample size, sampling date, location and prevalence.	Study published between January 01 and December 31, 2020.
Article in English or French or could be fully extracted using machine translation.	Article written in English or French or machine-translatable using Google Translate.
Article did not report identical information to previously included studies (peer-reviewed studies were prioritised over news stories and pre-prints where available).	Reported seroprevalence data that could be fit into the 23 major SOC 2010 occupation categories or combined categories for healthcare workers, first-responders or unemployed persons.<br><br>Studies that only reported seroprevalence for mixed occupation groups or workplaces rather than specific occupations (e.g., "hospital staff") were excluded.
Studies conducted only in people previously diagnosed with COVID-19 (molecular or antigen testing, or clinical or self-assessment).	Seroprevalence estimates did not include people <18 years (i.e., possibly affected by COVID-19 exposure at school, which could impact occupational seroprevalence estimates).
Cohort or cross-sectional design (case reports, case-control studies, trials, and reviews were excluded, as were dashboards not associated with a defined serology study).	

For peer review only

S4. Tool for assessing study risk of bias

**Item 1: Was the sample frame appropriate to address the target population?**	
Yes	Sample frame described and it approximated the target population
No	Sample frame did not approximate the target population (e.g., blood donors do not represent general population, doctors do not represent all health care providers)
Exclude	Sample frame not described
*Notes	The term "target population" should not be taken to infer every individual from everywhere or with similar disease or exposure characteristics. Instead, give consideration to specific population characteristics in the study, including age range, gender, morbidities, medications, and other potentially influential factors. For example, a sample frame may not be appropriate to address the target population if a certain group has been used (such as those working for one organisation, or one profession) and the results then inferred to the target population (i.e. working adults). A sample frame may be appropriate when it includes almost all the members of the target population (i.e. a census, or a complete list of participants or complete registry data).

**Item 2: Were study participants recruited in an appropriate way?**	
Yes	Probability sampling method (simple or stratified random) or entire sample (e.g., an entire town) was used
No	Non-probability sampling
Exclude	Sampling method not reported

**Item 3: Was the sample size adequate?**	
Yes	$\geq$599
No	<599
Exclude	Sample size not reported
*Notes	To calculate the required sample size we used an assumed prevalence of 2.5%, which was the global average estimated by the WHO in April, 2020.[1] Based on guidance by the Joanna Briggs Institute and published medical statistical recommendations we selected a precision value that was half the assumed prevalence (1.25%) [2,3]. We calculated a minimum sample size of 599 using these inputs:<br>Sample size calculation: $n = \frac{Z^2 P(1-P)}{d^2}$<br>Where n = sample size;<br>Z = Z statistic for level of confidence (95%);<br>P = expected prevalence (2.5% WHO global estimate);<br>d = precision (1.25%)<br><br>In cases where the sample size calculation was provided and the required sample for 80% power was below our threshold (n<599), this item was marked as yes.

**Item 4: Were the study subjects and setting described in detail?**	
Yes	Average age and distribution of gender/sex provided
No	Neither age or gender/sex is provided, or only one of age and gender/sex is provided

**Item 5: Was data analysis conducted with sufficient coverage of the identified sample?**	
Yes	The demographic characteristics (gender/sex, age, and ethnicity) of the sample is at least somewhat representative of the population
No	The demographic characteristics (gender/sex, age, and ethnicity) of the sample is not representative of the population

For peer review only

For peer review only - http://bmjopen.bmj.com/site/about/guidelines.xhtml

Unclear	Information is not provided about demographic characteristics of the sample (gender/sex, age, and ethnicity)

**Item 6: Were valid methods used for the identification of the condition?**	
Yes	The test used met the FDA standards for Emergency Use Authorizations for COVID-19 serological tests: sensitivity minimum 90%, specificity minimum 95%, as reported in the study [4].
No	The test used did not meet the FDA standards for Emergency Use Authorizations for COVID-19 serological tests: sensitivity minimum 90%, specificity minimum 95%.
Exclude	Test sensitivity and specificity not reported

**Item 7: Was the condition measured in a standard, reliable way for all participants?**	
Yes	The same serology test was used for all participants
No	Different serology tests were used for participants
Unclear	No details were provided about which participants received which serology tests

**Item 8: Was there appropriate statistical analysis?**	
Yes	Does all of the following: corrects for population characteristics or the sample is somewhat representative of the population (probability sampling), corrects for test characteristics), and provides the information necessary to determine the numerator, denominator, prevalence estimate, and confidence interval.
No	Does not correct for population characteristics and the sample is not likely representative of the population (non-probability sampling), does not correct for test or provide the information necessary to correct for test characteristics, or does not provide the information necessary to determine the numerator, denominator, prevalence estimate, and confidence interval.

**Item 9: Was the response rate adequate, and if not, was the low response rate managed appropriately?**	
Yes	Response rate > 60% or the demographics of the sample were a reasonable match to those of the target population [5]
No	Response rate < 60% and the demographics of the sample were not a reasonable match to those of the target population
Unclear	Response rate not provided and it was unclear if the demographics of the sample differed from the target population

**Item 10: Overall risk of bias**	
Low	The estimates are very likely correct for the target population. To obtain a low risk of bias classification, all criteria must be met or departures from the criteria must be minimal and unlikely to impact on the validity and reliability of the prevalence estimate. These include sample sizes that are just below the threshold when all other criteria are met, reporting only some of characteristics of the sample, test characteristics below the threshold but corrections for the test performance, and response rates that are just below the threshold in the context of probability based sampling of an appropriate sampling frame with population weighted seroprevalence estimates.
Moderate	The estimates are likely correct for the target population. To obtain a moderate risk of bias classification, most criteria must be met and departures from the criteria are likely to have only a small impact on the validity and reliability of the prevalence estimates.
High	The estimates are not likely correct for the target population. To obtain a high risk of bias, many criteria must not be met or departures from criteria are likely to have a major impact on the validity and reliability of the prevalence estimates.
Unclear	There was insufficient information to assess the risk of bias.

For peer review only - http://bmjopen.bmj.com/site/about/guidelines.xhtml

S5. Details of occupational coding

For each seroprevalence estimate, we identified the relevant Standard Occupational Classification (SOC) 2010 codes. This was done by applying the National Institute for Occupational Safety & Health (NIOSH) Industry and Occupation Computerized Coding System (NIOCCS) to text occupation descriptions extracted by members of the research team. There is no standard cut-off for manually verifying results from the National Institute for Occupational Safety & Health (NIOSH) Industry and Occupation Computerized Coding System (NIOCCS). However, NIOCCS reports the probability of correct classification to the six-digit level. After manually verifying a subset of records from the first round of classification, we decided to manual perform a second round of classification for any observations for which the probability of correct classification was <0.8. This cut-off was chosen based on the observation that that most codes with a probability of correct classification to of ≥0.8 to the six-digit level were correctly coded at the two- and three-digit level, which we used in our main analyses and are more likely to be coded correctly than the more granular, 6-digit codes and consideration of the number of records that could feasibly be verified manually

For peer review only - http://bmjopen.bmj.com/site/about/guidelines.xhtml

References for supplementary files

1. Boseley S. WHO warns that few have developed antibodies to Covid-19. The Guardian [Internet]. 2020 Apr 20; Available from: https://www.theguardian.com/society/2020/apr/20/studies-suggest-very-few-have-had-covid-19-without-symptoms
2. Munn Z, Moola S, Lisy K, Riitano D, Tufanaru C. Methodological guidance for systematic reviews of observational epidemiological studies reporting prevalence and cumulative incidence data. Int J Evid Based Healthc. 2015 Sep;13(3):147–53.
3. Naing L, Winn T, Ruslil B. Practical issues in calculating the sample size for prevalence studies. Arch Orofac Sci. 2006;1:9–14.
4. U.S. Food & Drug Administration. Emergency Use Authorization for SARS-CoV-2 Antibody Tests [Internet]. 2020 [cited 2020 May 5]. Available from: https://www.fda.gov/media/137470/download.
5. Morton MBS, Bandara DK, Robinson EM, Carr PEA. In the 21st century, what is an acceptable response rate? Aust N Z J Public Health. 2012 April; 36 (2): 106-8.
6 Bobrovitz N, Arora RK, Cao C, Boucher E, Liu M, Donnici C, Yanes-Lane M, Whelan M, Perlman-Arrow S, Chen J, Rahim H. Global seroprevalence of SARS-CoV-2 antibodies: A systematic review and meta-analysis. PloS one. 2021 Jun 23;16(6):e0252617.

For peer review only

33

For peer review only

For peer review only - http://bmjopen.bmj.com/site/about/guidelines.xhtml

Supplementary File I. List of all estimates, included studies and references

SOC 2010 Major Group	Study	N	SOC 2010 Occupation Title	Study Type	Study Dates	Country	Serum positive prevalence (95% CIs)	Overall Risk of Bias (JBI)
Not employed (mixed)*	Merkely et al., 2020[1]	n=209	Homemaker (Unpaid)	Cross-sectional survey	05/01 - 05/16	Hungary	0.73% (0-1.74%)	Moderate
Not employed (mixed)*	Siddiqui et al., 2020[2]	n=37	Homemaker (Unpaid)	Prospective cohort	04/15 - 08/15	India	18.9%	High
Not employed (mixed)*	Biggs et al., 2020[3]	n=157	Retired (Unpaid)	Cross-sectional survey	04/28 - 05/03	United States of America	1.91%	Moderate
Not employed (mixed)*	Carrat et al., 2020[4]	n=5381	Retired (Unpaid)	Prospective cohort	05/04 - 06/23	France	4.3% (3.5-5%)	Moderate
Not employed (mixed)*	Merkely et al., 2020[1]	n=2767	Retired (Unpaid)	Cross-sectional survey	05/01 - 05/16	Hungary	1.09% (0.66-1.52%)	Moderate
Not employed (mixed)*	Richard et al., 2020[5]	n=1635	Retired (Unpaid)	Cross-sectional survey	04/06 - 06/30	Switzerland	4.3%	Low
Not employed (mixed)*	Siddiqui et al., 2020[2]	n=10	Retired (Unpaid)	Prospective cohort	04/15 - 08/15	India	20%	High
Not employed (mixed)*	Alemu et al., 2020[6]	n=32	Student (Unpaid)	Cross-sectional survey	04/23 - 04/28	Ethiopia	15.6%	Moderate
Not employed (mixed)*	Biggs et al., 2020[3]	n=16	Student (Unpaid)	Cross-sectional survey	04/28 - 05/03	United States of America	12.5%	Moderate
Not employed (mixed)*	Brehm et al., 2020[7]	n=73	Student (Unpaid)	Cross sectional study with prospective cohort follow up of a subset of the sample	03/20 - 07/17	Germany	2.7%	Moderate
Not employed (mixed)*	Carrat et al., 2020[4]	n=81	Student (Unpaid)	Prospective cohort	05/04 - 06/23	France	7.2% (0.1-12.6%)	Moderate

For peer review only

Not employed (mixed)*	Iversen et al., 2020[8]	n=688	Student (Unpaid)	Cross-sectional survey	04/15 - 04/22	Denmark	14.97%	Low
Not employed (mixed)*	Lumley et al., 2020[9]	n=620	Student (Unpaid)	Prospective cohort	04/23 - 11/30	The United Kingdom	6.77%	Moderate
Not employed (mixed)*	Merkely et al., 2020[1]	n=774	Student (Unpaid)	Cross-sectional survey	05/01 - 05/16	Hungary	0.69% (0-1.49%)	Moderate
Not employed (mixed)*	Richard et al., 2020[5]	n=666	Student (Unpaid)	Cross-sectional survey	04/06 - 06/30	Switzerland	10.5%	Low
Not employed (mixed)*	Shakiba et al., 2020[10]	n=114	Student (Unpaid)	Cross-sectional survey	04/11 - 04/19	Iran (Islamic Republic of)	17.5% (11.3-23.7%)	Moderate
Not employed (mixed)*	Siddiqui et al., 2020[2]	n=14	Student (Unpaid)	Prospective cohort	04/15 - 08/15	India	21.4%	High
Not employed (mixed)*	Tilley et al., 2020[11]	n=790	Student (Unpaid)	Cross-sectional survey	04/29 - 05/08	United States of America	4% (3-5.1%)	Moderate
Not employed (mixed)*	Tsitsilonis et al., 2020[12]	n=1395	Student (Unpaid)	Cross-sectional survey	06/15 - 07/15	Greece	0.42% (0.03-1.5%)	Moderate
Not employed (mixed)*	Arnaldo et al., 2020[13]	n=513	Military, Rank Not Specified	Cross-sectional survey	07/06 - 07/13	Mozambique	3.7%	High
Not employed (mixed)*	Arnaldo et al., 2020[14]	n=116	Military, Rank Not Specified	Cross-sectional survey	11/02 - 11/12	Mozambique	1.7%	High
Not employed (mixed)*	Mabunda et al., 2020[15]	n=324	Military, Rank Not Specified	Cross-sectional survey	09/21 - 10/02	Mozambique	2.8%	High
Not employed (mixed)*	Mahomed et al., 2020[16]	n=116	Military, Rank Not Specified	Cross-sectional survey	11/26 - 12/03	Mozambique	18.1%	High
Not employed (mixed)*	Payne et al., 2020[17]	n=382	Military, Rank Not Specified	Cross-sectional survey	04/20 - 04/24	United States of America	59.7%	High
Not employed (mixed)*	World et al., 2020[18]	n=6900	Military, Rank Not Specified	Cross-sectional survey	08/15 - 10/15	Republic of Korea	0.36%	Unclear
Management Occupations (11-0000)	Shakiba et al., 2020[10]	n=16	Farmers, Ranchers, and Other Agricultural Managers	Cross-sectional survey	04/11 - 04/19	Iran (Islamic Republic of)	19.7% (9.1-31%)	Moderate
Management Occupations (11-	Favara et al., 2020[19]	n=43	Medical and Health Services Managers	Cross-sectional survey	07/13 - 07/13	The United Kingdom	9.3%	High

For peer review only

0000)								
Management Occupations (11-0000)	Galan et al., 2020[20]	n=170	Medical and Health Services Managers	Cross-sectional survey	04/14 - 04/27	Spain	27.6%	High
Management Occupations (11-0000)	Hunter et al., 2020[21]	n=44	Medical and Health Services Managers	Cross-sectional survey	04/29 - 05/08	United States of America	4.55%	High
Management Occupations (11-0000)	Leidner et al., 2020[22]	n=257	Medical and Health Services Managers	Cross sectional study with prospective cohort follow up of a subset of the sample	04/08 - 05/22	United States of America	3.11%	High
Management Occupations (11-0000)	Martin et al., 2020[23]	n=2078	Medical and Health Services Managers	Cross-sectional survey	05/29 - 07/13	The United Kingdom	6.79%	Moderate
Management Occupations (11-0000)	Siddiqui et al., 2020[2]	n=15	Medical and Health Services Managers	Prospective cohort	04/15 - 08/15	India	20%	High
Management Occupations (11-0000)	Baracco et al., 2020[24]	n=45	Managers, All Other	Cross-sectional survey	04/23 - 05/05	Italy	6.67%	High
Management Occupations (11-0000)	Goenka et al., 2020[25]	n=71	Managers, All Other	Cross-sectional survey	07/12 - 08/23	India	7.04%	Moderate
Management Occupations (11-0000)	Goenka et al., 2020[26]	n=13	Managers, All Other	Cross-sectional survey	08/01 - 08/31	India	38.46%	High
Business and Financial Operations Occupations (13-0000)	Satpati et al., 2020[27]	n=43	Management Analysts	Cross-sectional survey	07/26 - 08/08	India	2.33%	Moderate
Business and Financial	Poustchi et al., 2020[28]	n=880	Financial Specialists	Cross-sectional survey	04/17 - 06/02	Iran (Islamic Republic of)	14.2% (12.1-16.5%)	Moderate

For peer review only

Operations Occupations (13-0000)								
Computer and Mathematical Occupations (15-0000)	Biggs et al., 2020[3]	n=47	Computer User Support Specialists	Cross-sectional survey	04/28 - 05/03	United States of America	0%	Moderate
Architecture and Engineering Occupations (17-0000)	Siddiqui et al., 2020[2]	n=21	Engineers	Prospective cohort	04/15 - 08/15	India	42.9%	High
Life, Physical, and Social Science Occupations (19-0000)	Jones et al., 2020[29]	n=245	Medical Scientists	Cross-sectional survey	01/15 - 06/15	The United Kingdom	1.9%	High
Life, Physical, and Social Science Occupations (19-0000)	Anna et al., 2020[30]	n=505	Medical Scientists, Except Epidemiologists	Prospective cohort	04/28 - 07/31	France	8.71%	Moderate
Life, Physical, and Social Science Occupations (19-0000)	Erber et al., 2020[31]	n=635	Medical Scientists, Except Epidemiologists	Cross-sectional survey	04/14 - 05/29	Germany	1.24%	High
Life, Physical, and Social Science Occupations (19-0000)	Favara et al., 2020[19]	n=38	Medical Scientists, Except Epidemiologists	Cross-sectional survey	07/13 - 07/13	The United Kingdom	2.6%	High
Life, Physical, and Social Science Occupations (19-0000)	Hanrath et al., 2020[32]	n=468	Medical Scientists, Except Epidemiologists	Cross-sectional survey	05/29 - 07/06	The United Kingdom	6.2%	High
Life, Physical, and Social Science Occupations (19-0000)	Leidner et al., 2020[22]	n=2654	Medical Scientists, Except Epidemiologists	Cross sectional study with prospective cohort follow up of a subset of the sample	04/08 - 05/22	United States of America	2.22%	High

For peer review only

Life, Physical, and Social Science Occupations (19-0000)	Martin et al., 2020[23]	n=1154	Medical Scientists, Except Epidemiologists	Cross-sectional survey	05/29 - 07/13	The United Kingdom	9.71%	Moderate
Life, Physical, and Social Science Occupations (19-0000)	Rosser et al., 2020[33]	n=102	Medical Scientists, Except Epidemiologists	Cross-sectional survey	04/20 - 05/20	United States of America	0.98%	High
Life, Physical, and Social Science Occupations (19-0000)	Silva et al., 2020[34]	n=69	Chemists	Cross-sectional survey	06/05 - 07/31	Brazil	4%	High
Life, Physical, and Social Science Occupations (19-0000)	Tsitsilonis et al., 2020[12]	n=250	Physical Scientists, All Other	Cross-sectional survey	06/15 - 07/15	Greece	1.42% (0-7.24%)	Moderate
Community and Social Service Occupations (21-0000)	Jones et al., 2020[29]	n=211	Healthcare Social Workers	Cross-sectional survey	01/15 - 06/15	The United Kingdom	6.3%	High
Community and Social Service Occupations (21-0000)	Leidner et al., 2020[22]	n=235	Social Workers, All Other	Cross sectional study with prospective cohort follow up of a subset of the sample	04/08 - 05/22	United States of America	3.4%	High
Community and Social Service Occupations (21-0000)	Rosser et al., 2020[33]	n=117	Social Workers, All Other	Cross-sectional survey	04/20 - 05/20	United States of America	1.71%	High
Community and Social Service Occupations (21-0000)	Sabourin et al., 2020[35]	n=91	Social Workers, All Other	Cross-sectional survey	07/15 - 08/15	United States of America	5.49%	High
Community and Social Service	Yogo et al., 2020[36]	n=35	Social Workers, All Other	Cross-sectional survey	05/20 - 06/08	United States of America	0%	High

For peer review only

Occupations (21-0000)								
Community and Social Service Occupations (21-0000)	Biggs et al., 2020[3]	n=6	Religious Workers	Cross-sectional survey	04/28 - 05/03	United States of America	16.67%	Moderate
Education, Training, and Library Occupations (25-0000)	Campos et al., 2020[37]	n=2715	Postsecondary Teachers	Cross-sectional survey	05/13 - 07/10	Portugal	2.6%	High
Education, Training, and Library Occupations (25-0000)	Goncalves et al., 2020[38]	n=1636	Postsecondary Teachers	Cross-sectional survey	06/15 - 06/30	Portugal	3.05%	Moderate
Education, Training, and Library Occupations (25-0000)	Tsitsilonis et al., 2020[12]	n=312	Postsecondary Teachers	Cross-sectional survey	06/15 - 07/15	Greece	1.2% (0.14-3.7%)	Moderate
Education, Training, and Library Occupations (25-0000)	Fontanet et al., 2020[39]	n=42	Elementary and Middle School Teachers	Retrospective cohort	04/28 - 04/30	France	7.1%	Moderate
Education, Training, and Library Occupations (25-0000)	Siddiqui et al., 2020[2]	n=8	Elementary and Middle School Teachers	Prospective cohort	04/15 - 08/15	India	25%	High
Education, Training, and Library Occupations (25-0000)	Torres et al., 2020[40]	n=165	Elementary and Middle School Teachers	Cross-sectional survey	05/04 - 05/19	Chile	20.6% (14.7-27.6%)	High

For peer review only

Arts, Design, Entertainment, Sports, and Media Occupations (27-0000)	Halatoko et al., 2020[41]	n=55	Fine Artists, Including Painters, Sculptors, and Illustrators	Cross-sectional survey	04/23 - 05/08	Togo	0%	High
Arts, Design, Entertainment, Sports, and Media Occupations (27-0000)	Slusser et al., 2020[42]	n=5603	Athletes, Coaches, Umpires, and Related Workers	Cross-sectional survey	04/08 - 04/21	United States of America	0.7% (0.28-1.15%)	Unclear
Arts, Design, Entertainment, Sports, and Media Occupations (27-0000)	Vince et al., 2020[43]	n=272	Athletes, Coaches, Umpires, and Related Workers	Prospective cohort	05/29 - 07/31	Croatia	14%	Moderate
Arts, Design, Entertainment, Sports, and Media Occupations (27-0000)	Vince et al., 2020[43]	n=43	Coaches and Scouts	Prospective cohort	05/29 - 07/31	Croatia	16.3%	Moderate
Arts, Design, Entertainment, Sports, and Media Occupations (27-0000)	Mack et al., 2020[44]	n=1007	Umpires, Referees, and Other Sports Officials	Prospective cohort	06/16 - 06/30	Germany	2.09% (1.37-3.17%)	High
Arts, Design, Entertainment, Sports, and Media Occupations (27-0000)	Khan et al., 2020[45]	n=44	Media and Communication Workers	Cross-sectional survey	07/01 - 07/15	India	0%	Moderate
Healthcare Practitioners and Technical Occupations (29-0000)	Akinbami et al., 2020[46]	n=566	Healthcare Practitioners and Technical Occupations	Cross-sectional survey	05/18 - 06/13	United States of America	4.6% (3-6.7%)	Moderate

For peer review only

Healthcare Practitioners and Technical Occupations (29-0000)	Khan et al., 2020[45]	n=355	Healthcare Practitioners and Technical Occupations	Cross-sectional survey	07/01 - 07/15	India	4.8% (3-7.6%)	Moderate
Healthcare Practitioners and Technical Occupations (29-0000)	Leidner et al., 2020[22]	n=402	Healthcare Practitioners and Technical Occupations	Cross sectional study with prospective cohort follow up of a subset of the sample	04/08 - 05/22	United States of America	1.49%	High
Healthcare Occupations (mixed)*	Hanrath et al., 2020[32]	n=102	Healthcare Practitioners and Technical Occupations and Healthcare Support Occupations*	Cross-sectional survey	05/29 - 07/06	The United Kingdom	6.62%	High
Healthcare Occupations (mixed)*	Jones et al., 2020[29]	n=413	Healthcare Practitioners and Technical Occupations and Healthcare Support Occupations*	Cross-sectional survey	01/15 - 06/15	The United Kingdom	7.8%	High
Healthcare Occupations (mixed)*	Martin et al., 2020[23]	n=550	Healthcare Practitioners and Technical Occupations and Healthcare Support Occupations*	Cross-sectional survey	05/29 - 07/13	The United Kingdom	10.36%	Moderate
Healthcare Occupations (mixed)*	Amendola et al., 2020[47]	n=117	Healthcare Practitioners and Technical Occupations and Healthcare Support Occupations*	Cross-sectional survey	04/15 - 04/15	Italy	4.27%	High
Healthcare Occupations (mixed)*	Arnaldo et al., 2020[48]	n=543	Healthcare Practitioners and Technical Occupations and Healthcare Support Occupations*	Cross-sectional survey	08/10 - 08/21	Mozambique	3.7%	High

For peer review only

Healthcare Occupations (mixed)*	Bal et al., 2020[49]	n=190	Healthcare Practitioners and Technical Occupations and Healthcare Support Occupations*	Cross-sectional survey	04/10 - 05/28	France	3.68%	High
Healthcare Occupations (mixed)*	Barallat et al., 2020[50]	n=429	Healthcare Practitioners and Technical Occupations and Healthcare Support Occupations*	Cross-sectional survey	05/04 - 05/22	Spain	7.69%	High
Healthcare Occupations (mixed)*	Bardai et al., 2020[51]	n=35	Healthcare Practitioners and Technical Occupations and Healthcare Support Occupations*	Cross-sectional survey	06/10 - 07/27	Canada	11%	High
Healthcare Occupations (mixed)*	Bardai et al., 2020[51]	n=20	Healthcare Practitioners and Technical Occupations and Healthcare Support Occupations*	Cross-sectional survey	06/10 - 07/27	Canada	15%	High
Healthcare Occupations (mixed)*	Bardai et al., 2020[51]	n=44	Healthcare Practitioners and Technical Occupations and Healthcare Support Occupations*	Cross-sectional survey	06/10 - 07/27	Canada	11%	High
Healthcare Occupations (mixed)*	Bardai et al., 2020[51]	n=99	Healthcare Practitioners and Technical Occupations and Healthcare Support Occupations*	Cross-sectional survey	06/10 - 07/27	Canada	12%	High
Healthcare Occupations (mixed)*	Biggs et al., 2020[3]	n=59	Healthcare Practitioners and Technical Occupations and Healthcare Support Occupations*	Cross-sectional survey	04/28 - 05/03	United States of America	10.17%	Moderate

For peer review only

Healthcare Occupations (mixed)*	Blairon et al., 2020[52]	n=588	Healthcare Practitioners and Technical Occupations and Healthcare Support Occupations*	Cross-sectional survey	05/25 - 06/19	Belgium	19.2%	High
Healthcare Occupations (mixed)*	Borraz et al., 2020[53]	n=289	Healthcare Practitioners and Technical Occupations and Healthcare Support Occupations*	Prospective cohort	03/20 - 04/21	Spain	5.88%	High
Healthcare Occupations (mixed)*	Brunner et al., 2020[54]	n=762	Healthcare Practitioners and Technical Occupations and Healthcare Support Occupations*	Cross-sectional survey	05/04 - 05/29	United States of America	4.5%	High
Healthcare Occupations (mixed)*	Brunner et al., 2020[54]	n=764	Healthcare Practitioners and Technical Occupations and Healthcare Support Occupations*	Cross-sectional survey	05/04 - 05/29	United States of America	2%	High
Healthcare Occupations (mixed)*	Carozzi et al., 2020[55]	n=17098	Healthcare Practitioners and Technical Occupations and Healthcare Support Occupations*	Cross-sectional survey	04/01 - 04/30	Italy	3.1%	High
Healthcare Occupations (mixed)*	Carrat et al., 2020[4]	n=568	Healthcare Practitioners and Technical Occupations and Healthcare Support Occupations*	Prospective cohort	05/04 - 06/23	France	11.6% (8.3-14.4%)	Moderate
Healthcare Occupations (mixed)*	Cavlek et al., 2020[56]	n=558	Healthcare Practitioners and Technical Occupations and Healthcare Support Occupations*	Cross-sectional survey	04/25 - 05/24	Croatia	1.25%	High

For peer review only

Healthcare Occupations (mixed)*	Chibwana et al., 2020[57]	n=500	Healthcare Practitioners and Technical Occupations and Healthcare Support Occupations*	Prospective cohort	05/22 - 06/19	Malawi	12.3% (8.2-16.5%)	High
Healthcare Occupations (mixed)*	Coffman et al., 2020[58]	n=1100	Healthcare Practitioners and Technical Occupations and Healthcare Support Occupations*	Cross-sectional survey	07/01 - 07/31	United States of America	2.2%	Unclear
Healthcare Occupations (mixed)*	Cooper et al., 2020[59]	n=118	Healthcare Practitioners and Technical Occupations and Healthcare Support Occupations*	Cross-sectional survey	06/10 - 08/07	The United Kingdom	8.47%	Moderate
Healthcare Occupations (mixed)*	Cooper et al., 2020[59]	n=27	Healthcare Practitioners and Technical Occupations and Healthcare Support Occupations*	Cross-sectional survey	06/10 - 08/07	The United Kingdom	14.81%	Moderate
Healthcare Occupations (mixed)*	Cooper et al., 2020[59]	n=24	Healthcare Practitioners and Technical Occupations and Healthcare Support Occupations*	Cross-sectional survey	06/10 - 08/07	The United Kingdom	12.5%	Moderate
Healthcare Occupations (mixed)*	Cooper et al., 2020[59]	n=1068	Healthcare Practitioners and Technical Occupations and Healthcare Support Occupations*	Cross-sectional survey	06/10 - 08/07	The United Kingdom	5.43%	Moderate
Healthcare Occupations (mixed)*	Cooper et al., 2020[59]	n=174	Healthcare Practitioners and Technical Occupations and Healthcare Support Occupations*	Cross-sectional survey	06/10 - 08/07	The United Kingdom	5.75%	Moderate

For peer review only

Healthcare Occupations (mixed)*	Cooper et al., 2020[59]	n=319	Healthcare Practitioners and Technical Occupations and Healthcare Support Occupations*	Cross-sectional survey	06/10 - 08/07	The United Kingdom	11.29%	Moderate
Healthcare Occupations (mixed)*	Cooper et al., 2020[59]	n=5698	Healthcare Practitioners and Technical Occupations and Healthcare Support Occupations*	Cross-sectional survey	06/10 - 08/07	The United Kingdom	7.2%	Moderate
Healthcare Occupations (mixed)*	Cooper et al., 2020[59]	n=412	Healthcare Practitioners and Technical Occupations and Healthcare Support Occupations*	Cross-sectional survey	06/10 - 08/07	The United Kingdom	4.61%	Moderate
Healthcare Occupations (mixed)*	Denyer et al., 2020[60]	n=5850	Healthcare Practitioners and Technical Occupations and Healthcare Support Occupations*	Cross-sectional survey	05/12 - 05/18	Japan	1.79%	Unclear
Healthcare Occupations (mixed)*	Dimeglio et al., 2020[61]	n=8758	Healthcare Practitioners and Technical Occupations and Healthcare Support Occupations*	Cross-sectional survey	06/10 - 07/10	France	3.2% (2.8-3.5%)	High
Healthcare Occupations (mixed)*	Erber et al., 2020[31]	n=603	Healthcare Practitioners and Technical Occupations and Healthcare Support Occupations*	Cross-sectional survey	04/14 - 05/29	Germany	2.8%	High
Healthcare Occupations (mixed)*	Fuereder et al., 2020[62]	n=62	Healthcare Practitioners and Technical Occupations and Healthcare Support Occupations*	Retrospective cohort	04/01 - 06/04	Austria	3.2% (0.4-11.2%)	High

For peer review only

Healthcare Occupations (mixed)*	Fusco et al., 2020[63]	n=115	Healthcare Practitioners and Technical Occupations and Healthcare Support Occupations*	Cross-sectional survey	03/23 - 04/02	Italy	1.74%	High
Healthcare Occupations (mixed)*	Geraci et al., 2020[64]	n=230	Healthcare Practitioners and Technical Occupations and Healthcare Support Occupations*	Cross-sectional survey	03/16 - 05/20	United States of America	2.17%	High
Healthcare Occupations (mixed)*	Gudo et al., 2020[65]	n=1427	Healthcare Practitioners and Technical Occupations and Healthcare Support Occupations*	Cross-sectional survey	06/17 - 06/30	Mozambique	7% (6-9%)	High
Healthcare Occupations (mixed)*	Hackner et al., 2020[66]	n=130	Healthcare Practitioners and Technical Occupations and Healthcare Support Occupations*	Cross-sectional survey	04/01 - 04/30	Austria	2.3%	High
Healthcare Occupations (mixed)*	Halatoko et al., 2020[41]	n=370	Healthcare Practitioners and Technical Occupations and Healthcare Support Occupations*	Cross-sectional survey	04/23 - 05/08	Togo	1.4%	High
Healthcare Occupations (mixed)*	Haq et al., 2020[67]	n=76	Healthcare Practitioners and Technical Occupations and Healthcare Support Occupations*	Cross-sectional survey	06/15 - 06/29	Pakistan	35.5% (24.8-47.3%)	Moderate
Healthcare Occupations (mixed)*	He et al., 2020[68]	n=1059	Healthcare Practitioners and Technical Occupations and Healthcare Support Occupations*	Repeated cross sectional study	05/13 - 06/10	China	9.3%	High

For peer review only

Healthcare Occupations (mixed)*	Herzberg et al., 2020[69]	n=871	Healthcare Practitioners and Technical Occupations and Healthcare Support Occupations*	Prospective cohort	04/14 - 06/16	Germany	2.64%	High
Healthcare Occupations (mixed)*	Jeremias et al., 2020[70]	n=100	Healthcare Practitioners and Technical Occupations and Healthcare Support Occupations*	Cross-sectional survey	03/01 - 04/30	United States of America	12%	High
Healthcare Occupations (mixed)*	Jespersen et al., 2020[71]	n=17948	Healthcare Practitioners and Technical Occupations and Healthcare Support Occupations*	Cross-sectional survey	05/18 - 06/19	Denmark	3.36% (2.38-3.82%)	Moderate
Healthcare Occupations (mixed)*	Kassem et al., 2020[72]	n=74	Healthcare Practitioners and Technical Occupations and Healthcare Support Occupations*	Cross-sectional survey	06/01 - 06/14	Egypt	12.2%	High
Healthcare Occupations (mixed)*	Kern et al., 2020[73]	n=1316	Healthcare Practitioners and Technical Occupations and Healthcare Support Occupations*	Cross-sectional survey	04/09 - 04/16	Germany	1.06% (0.58-1.78%)	High
Healthcare Occupations (mixed)*	Khalil et al., 2020[74]	n=190	Healthcare Practitioners and Technical Occupations and Healthcare Support Occupations*	Cross-sectional survey	05/15 - 05/28	The United Kingdom	22%	High
Healthcare Occupations (mixed)*	Kumar et al., 2020[75]	n=635	Healthcare Practitioners and Technical Occupations and Healthcare Support Occupations*	Repeated cross sectional study	07/11 - 07/24	India	0%	High

For peer review only

Healthcare Occupations (mixed)*	Lackermair et al., 2020[76]	n=151	Healthcare Practitioners and Technical Occupations and Healthcare Support Occupations*	Cross-sectional survey	04/02 - 04/06	Germany	2.6% (0.8-7.1%)	High
Healthcare Occupations (mixed)*	Lahner et al., 2020[77]	n=1084	Healthcare Practitioners and Technical Occupations and Healthcare Support Occupations*	Cross-sectional survey	04/07 - 04/27	Italy	0.7%	High
Healthcare Occupations (mixed)*	Liu et al., 2020[78]	n=116	Healthcare Practitioners and Technical Occupations and Healthcare Support Occupations*	Cross-sectional survey	02/07 - 04/21	China	0%	High
Healthcare Occupations (mixed)*	Liu et al., 2020[78]	n=304	Healthcare Practitioners and Technical Occupations and Healthcare Support Occupations*	Cross-sectional survey	02/07 - 04/21	China	0%	High
Healthcare Occupations (mixed)*	Liu et al., 2020[79]	n=3832	Healthcare Practitioners and Technical Occupations and Healthcare Support Occupations*	Cross-sectional survey	02/29 - 04/29	China	4% (3.4-4.7%)	Moderate
Healthcare Occupations (mixed)*	Lorenzo et al., 2020[80]	n=38	Healthcare Practitioners and Technical Occupations and Healthcare Support Occupations*	Cross-sectional survey	05/02 - 05/31	Italy	5.3%	High
Healthcare Occupations (mixed)*	Mahomed et al., 2020[81]	n=569	Healthcare Practitioners and Technical Occupations and Healthcare Support Occupations*	Cross-sectional survey	08/31 - 10/12	Mozambique	0.7%	High

For peer review only

Healthcare Occupations (mixed)*	Mahumane et al., 2020[82]	n=380	Healthcare Practitioners and Technical Occupations and Healthcare Support Occupations*	Cross-sectional survey	11/02 - 11/17	Mozambique	1.3%	High
Healthcare Occupations (mixed)*	Majdoubi et al., 2020[83]	n=276	Healthcare Practitioners and Technical Occupations and Healthcare Support Occupations*	Cross-sectional survey	05/17 - 06/19	Canada	0.6% (0-2.71%)	High
Healthcare Occupations (mixed)*	Majiya et al., 2020[84]	n=185	Healthcare Practitioners and Technical Occupations and Healthcare Support Occupations*	Cross-sectional survey	06/26 - 06/30	Nigeria	25.41%	Moderate
Healthcare Occupations (mixed)*	Majiya et al., 2020[84]	n=43	Healthcare Practitioners and Technical Occupations and Healthcare Support Occupations*	Cross-sectional survey	06/26 - 06/30	Nigeria	37.21%	Moderate
Healthcare Occupations (mixed)*	Malfertheiner et al., 2020[85]	n=139	Healthcare Practitioners and Technical Occupations and Healthcare Support Occupations*	Prospective cohort	03/15 - 06/07	Germany	0%	High
Healthcare Occupations (mixed)*	Martin et al., 2020[86]	n=326	Healthcare Practitioners and Technical Occupations and Healthcare Support Occupations*	Cross-sectional survey	04/15 - 05/18	Belgium	11%	High
Healthcare Occupations (mixed)*	Martin et al., 2020[23]	n=4631	Healthcare Practitioners and Technical Occupations and Healthcare Support Occupations*	Cross-sectional survey	05/29 - 07/13	The United Kingdom	13.65%	Moderate

For peer review only

Healthcare Occupations (mixed)*	Melo et al., 2020[87]	n=471	Healthcare Practitioners and Technical Occupations and Healthcare Support Occupations*	Cross-sectional survey	06/01 - 06/30	Brazil	13.59%	High
Healthcare Occupations (mixed)*	Morcuende et al., 2020[88]	n=6	Healthcare Practitioners and Technical Occupations and Healthcare Support Occupations*	Cross-sectional survey	03/01 - 04/21	United States of America	0%	High
Healthcare Occupations (mixed)*	Moscola et al., 2020[89]	n=8156	Healthcare Practitioners and Technical Occupations and Healthcare Support Occupations*	Cross-sectional survey	04/20 - 06/23	United States of America	11.6%	High
Healthcare Occupations (mixed)*	Nishida et al., 2020[90]	n=49	Healthcare Practitioners and Technical Occupations and Healthcare Support Occupations*	Cross-sectional survey	06/12 - 06/19	Japan	0%	Moderate
Healthcare Occupations (mixed)*	Olalla et al., 2020[91]	n=498	Healthcare Practitioners and Technical Occupations and Healthcare Support Occupations*	Cross-sectional survey	04/15 - 04/25	Spain	2.2%	High
Healthcare Occupations (mixed)*	Pallett et al., 2020[92]	n=504	Healthcare Practitioners and Technical Occupations and Healthcare Support Occupations*	Prospective cohort	04/08 - 06/12	The United Kingdom	10.6% (7.6-13.6%)	High
Healthcare Occupations (mixed)*	Pere et al., 2020[93]	n=3569	Healthcare Practitioners and Technical Occupations and Healthcare Support Occupations*	Cross-sectional survey	05/02 - 06/26	France	11.9%	High

For peer review only

Healthcare Occupations (mixed)*	Poulikakos et al., 2020[94]	n=281	Healthcare Practitioners and Technical Occupations and Healthcare Support Occupations*	Cross-sectional survey	05/04 - 05/06	The United Kingdom	6%	High
Healthcare Occupations (mixed)*	Psichogiou et al., 2020[95]	n=1495	Healthcare Practitioners and Technical Occupations and Healthcare Support Occupations*	Cross-sectional survey	04/13 - 05/15	Greece	1.26% (0.43-3.26%)	Moderate
Healthcare Occupations (mixed)*	Satpati et al., 2020[27]	n=18	Healthcare Practitioners and Technical Occupations and Healthcare Support Occupations*	Cross-sectional survey	07/26 - 08/08	India	5.56%	Moderate
Healthcare Occupations (mixed)*	Seetharam et al., 2020[96]	n=728	Healthcare Practitioners and Technical Occupations and Healthcare Support Occupations*	Cross-sectional survey	08/16 - 08/29	India	27.3% (24.1-30.6%)	Unclear
Healthcare Occupations (mixed)*	Shakiba et al., 2020[10]	n=43	Healthcare Practitioners and Technical Occupations and Healthcare Support Occupations*	Cross-sectional survey	04/11 - 04/19	Iran (Islamic Republic of)	14.5% (4.5-25%)	Moderate
Healthcare Occupations (mixed)*	Shields et al., 2020[97]	n=516	Healthcare Practitioners and Technical Occupations and Healthcare Support Occupations*	Cross-sectional survey	04/24 - 04/25	The United Kingdom	24.4%	High
Healthcare Occupations (mixed)*	Silva et al., 2020[98]	n=61	Healthcare Practitioners and Technical Occupations and Healthcare Support Occupations*	Cross-sectional survey	04/09 - 04/29	Brazil	4.91%	High

For peer review only

Healthcare Occupations (mixed)*	Solodky et al., 2020[99]	n=85	Healthcare Practitioners and Technical Occupations and Healthcare Support Occupations*	Cross-sectional survey	03/01 - 04/16	France	5.88%	High
Healthcare Occupations (mixed)*	Soriano et al., 2020[100]	n=108	Healthcare Practitioners and Technical Occupations and Healthcare Support Occupations*	Retrospective cohort	04/26 - 05/16	Spain	13%	High
Healthcare Occupations (mixed)*	Statistica et al., 2020[101]	n=64660	Healthcare Practitioners and Technical Occupations and Healthcare Support Occupations*	Cross-sectional survey	05/25 - 07/15	Italy	2.5%	Unclear
Healthcare Occupations (mixed)*	Steensels et al., 2020[102]	n=3056	Healthcare Practitioners and Technical Occupations and Healthcare Support Occupations*	Cross-sectional survey	04/22 - 04/30	Belgium	6.4% (5.5-7.3%)	High
Healthcare Occupations (mixed)*	Stock et al., 2020[103]	n=98	Healthcare Practitioners and Technical Occupations and Healthcare Support Occupations*	Cross-sectional survey	04/04 - 04/20	United States of America	15.3%	High
Healthcare Occupations (mixed)*	Takita et al., 2020[104]	n=175	Healthcare Practitioners and Technical Occupations and Healthcare Support Occupations*	Cross-sectional survey	04/20 - 05/20	Japan	4% (1.62-8.07%)	High
Healthcare Occupations (mixed)*	Tong et al., 2020[105]	n=191	Healthcare Practitioners and Technical Occupations and Healthcare Support Occupations*	Cross-sectional survey	05/12 - 05/15	China	0%	High

For peer review only

Healthcare Occupations (mixed)*	Trieu et al., 2020[106]	n=607	Healthcare Practitioners and Technical Occupations and Healthcare Support Occupations*	Prospective cohort	03/06 - 04/09	Norway	5.27%	High
Healthcare Occupations (mixed)*	Tu et al., 2020[107]	n=325	Healthcare Practitioners and Technical Occupations and Healthcare Support Occupations*	Cross sectional study with prospective cohort follow up of a subset of the sample	03/19 - 03/20	China	43.08%	High
Healthcare Occupations (mixed)*	Valdivia et al., 2020[108]	n=1153	Healthcare Practitioners and Technical Occupations and Healthcare Support Occupations*	Cross-sectional survey	04/13 - 04/30	Spain	3.5%	High
Healthcare Occupations (mixed)*	Vasquez et al., 2020[109]	n=1147	Healthcare Practitioners and Technical Occupations and Healthcare Support Occupations*	Cross-sectional survey	05/19 - 06/06	Peru	58.3%	High
Healthcare Occupations (mixed)*	Viegas et al., 2020[110]	n=1443	Healthcare Practitioners and Technical Occupations and Healthcare Support Occupations*	Cross-sectional survey	08/03 - 08/21	Mozambique	2.63%	High
Healthcare Occupations (mixed)*	Vlachoyiannopoulosa et al., 2020[111]	n=321	Healthcare Practitioners and Technical Occupations and Healthcare Support Occupations*	Cross-sectional survey	04/25 - 05/10	Greece	2.18%	High
Healthcare Occupations (mixed)*	Volta et al., 2020[112]	n=76	Healthcare Practitioners and Technical Occupations and Healthcare Support Occupations*	Cross-sectional survey	04/27 - 04/27	Italy	11.8%	High

Healthcare Occupations (mixed)*	Ward et al., 2020[113]	n=5416	Healthcare Practitioners and Technical Occupations and Healthcare Support Occupations*	Cross-sectional survey	09/15 - 09/28	The United Kingdom	10.67%	Moderate
Healthcare Occupations (mixed)*	Ward et al., 2020[113]	n=1692	Healthcare Practitioners and Technical Occupations and Healthcare Support Occupations*	Cross-sectional survey	09/15 - 09/28	The United Kingdom	6.68%	Moderate
Healthcare Occupations (mixed)*	Xiong et al., 2020[114]	n=797	Healthcare Practitioners and Technical Occupations and Healthcare Support Occupations*	Cross-sectional survey	02/12 - 03/17	China	4.39%	Unclear
Healthcare Occupations (mixed)*	Zhang et al., 2020[115]	n=63	Healthcare Practitioners and Technical Occupations and Healthcare Support Occupations*	Cross-sectional survey	01/21 - 02/16	China	0%	High
Healthcare Occupations (mixed)*	Zhao et al., 2020[116]	n=1060	Healthcare Practitioners and Technical Occupations and Healthcare Support Occupations*	Cross-sectional survey	01/14 - 02/21	China	8.3%	High
First responders (mixed)*	Ahmad et al., 2020[117]	n=40	Healthcare Practitioners and Technical Occupations and Protective Service Occupations (i.e. first responders)*	Cross-sectional survey	04/21 - 05/22	United States of America	20%	High
First responders (mixed)*	Halbrook et al., 2020[118]	n=679	Healthcare Practitioners and Technical Occupations and Protective Service Occupations (i.e. first responders)*	Cross-sectional survey	05/19 - 08/31	United States of America	8.1%	Moderate

For peer review only

First responders (mixed)*	Iwuji et al., 2020[119]	n=683	Healthcare Practitioners and Technical Occupations and Protective Service Occupations (i.e. first responders)*	Cross-sectional survey	05/12 - 05/13	United States of America	0.7%	High
First responders (mixed)*	Magyar et al., 2020[120]	n=70	Healthcare Practitioners and Technical Occupations and Protective Service Occupations (i.e. first responders)*	Cross-sectional survey	05/01 - 05/14	United States of America	4.29%	High
First responders (mixed)*	Martinez et al., 2020[121]	n=79	Healthcare Practitioners and Technical Occupations and Protective Service Occupations (i.e. first responders)*	Cross-sectional survey	04/16 - 04/17	United States of America	5.06%	High
First responders (mixed)*	Staletovich et al., 2020[122]	n=359	Healthcare Practitioners and Technical Occupations and Protective Service Occupations (i.e. first responders)*	Cross-sectional survey	05/17 - 05/22	United States of America	0%	Unclear
Healthcare Practitioners and Technical Occupations (29-0000)	Hibino et al., 2020[123]	n=806	Health Diagnosing and Treating Practitioners	Cross-sectional survey	06/01 - 07/30	Japan	0.74% (0.27-1.61%)	Unclear
Healthcare Practitioners and Technical Occupations (29-0000)	Jones et al., 2020[29]	n=856	Dentists, General	Cross-sectional survey	01/15 - 06/15	The United Kingdom	7.9%	High
Life, Physical, and Social Science	Calcagno et al., 2020[124]	n=343	Life, Physical, and Social Science Occupations	Cross-sectional survey	04/17 - 05/20	Italy	6.71%	Moderate

For peer review only

Occupations (19-0000)								
Healthcare Practitioners and Technical Occupations (29-0000)	Goenka et al., 2020[25]	n=49	Dietitians and Nutritionists	Cross-sectional survey	07/12 - 08/23	India	18.37%	Moderate
Healthcare Practitioners and Technical Occupations (29-0000)	Goenka et al., 2020[26]	n=6	Dietitians and Nutritionists	Cross-sectional survey	08/01 - 08/31	India	0%	High
Healthcare Practitioners and Technical Occupations (29-0000)	Akinbami et al., 2020[46]	n=321	Pharmacists	Cross-sectional survey	05/18 - 06/13	United States of America	4.4% (2.4-7.2%)	Moderate
Healthcare Practitioners and Technical Occupations (29-0000)	Alharbi et al., 2020[125]	n=5	Pharmacists	Cross-sectional survey	04/18 - 06/17	Saudi Arabia	0%	High
Healthcare Practitioners and Technical Occupations (29-0000)	Calcagno et al., 2020[124]	n=29	Pharmacists	Cross-sectional survey	04/17 - 05/20	Italy	3.45%	Moderate
Healthcare Practitioners and Technical Occupations (29-0000)	Chau et al., 2020[126]	n=17	Pharmacists	Cross-sectional survey	08/23 - 08/30	Viet Nam	0%	High
Healthcare Practitioners and Technical Occupations (29-0000)	Hanrath et al., 2020[32]	n=189	Pharmacists	Cross-sectional survey	05/29 - 07/06	The United Kingdom	4.76%	High

For peer review only

Healthcare Practitioners and Technical Occupations (29-0000)	Khan et al., 2020[127]	n=109	Pharmacists	Cross-sectional survey	06/15 - 06/29	India	0%	Moderate
Healthcare Practitioners and Technical Occupations (29-0000)	Mahomed et al., 2020[81]	n=404	Pharmacists	Cross-sectional survey	08/31 - 10/12	Mozambique	0.5%	High
Healthcare Practitioners and Technical Occupations (29-0000)	Martin et al., 2020[23]	n=113	Pharmacists	Cross-sectional survey	05/29 - 07/13	The United Kingdom	0%	Moderate
Healthcare Practitioners and Technical Occupations (29-0000)	Rosser et al., 2020[33]	n=213	Pharmacists	Cross-sectional survey	04/20 - 05/20	United States of America	1.88%	High
Healthcare Practitioners and Technical Occupations (29-0000)	Costa et al., 2020[128]	n=652	Physicians and Surgeons	Cross-sectional survey	05/14 - 05/28	Brazil	5.8%	Moderate
Healthcare Practitioners and Technical Occupations (29-0000)	Mohr et al., 2020[129]	n=372	Physicians and Surgeons	Cross-sectional survey	05/13 - 07/08	United States of America	1.61%	Moderate
Healthcare Practitioners and Technical Occupations (29-0000)	Nishida et al., 2020[90]	n=63	Physicians and Surgeons	Cross-sectional survey	06/12 - 06/19	Japan	3.2% (0.88-11%)	Moderate
Healthcare Practitioners and	Noor et al., 2020[130]	n=157	Physicians and Surgeons	Cross-sectional survey	07/13 - 07/15	Pakistan	17.83%	Moderate

For peer review only

Technical Occupations (29-0000)								
Healthcare Practitioners and Technical Occupations (29-0000)	Singhal et al., 2020[131]	n=208	Physicians and Surgeons	Cross-sectional survey	06/01 - 06/30	India	12.5%	High
Healthcare Practitioners and Technical Occupations (29-0000)	Morcuende et al., 2020[88]	n=23	Anesthesiologists	Cross-sectional survey	03/01 - 04/21	United States of America	13.04%	High
Healthcare Practitioners and Technical Occupations (29-0000)	Morcuende et al., 2020[88]	n=3	Obstetricians and Gynecologists	Cross-sectional survey	03/01 - 04/21	United States of America	100%	High
Healthcare Practitioners and Technical Occupations (29-0000)	Urbieta et al., 2020[132]	n=23	Pediatricians, General	Cross-sectional survey	04/14 - 04/16	Spain	4.3%	High
Healthcare Practitioners and Technical Occupations (29-0000)	Iversen et al., 2020[8]	n=1944	Psychiatrists	Cross-sectional survey	04/15 - 04/22	Denmark	1.85%	Low
Healthcare Practitioners and Technical Occupations (29-0000)	Leidner et al., 2020[22]	n=301	Surgeons	Cross sectional study with prospective cohort follow up of a subset of the sample	04/08 - 05/22	United States of America	2.66%	High
Healthcare Practitioners and Technical	Akinbami et al., 2020[46]	n=2297	Physicians and Surgeons, All Other	Cross-sectional survey	05/18 - 06/13	United States of America	6.1% (5.1-7.1%)	Moderate

For peer review only

Occupations (29-0000)								
Healthcare Practitioners and Technical Occupations (29-0000)	Alharbi et al., 2020[125]	n=18	Physicians and Surgeons, All Other	Cross-sectional survey	04/18 - 06/17	Saudi Arabia	27.78%	High
Healthcare Practitioners and Technical Occupations (29-0000)	Amendola et al., 2020[47]	n=214	Physicians and Surgeons, All Other	Cross-sectional survey	04/15 - 04/15	Italy	4.67%	High
Healthcare Practitioners and Technical Occupations (29-0000)	Baracco et al., 2020[24]	n=417	Physicians and Surgeons, All Other	Cross-sectional survey	04/23 - 05/05	Italy	17%	High
Healthcare Practitioners and Technical Occupations (29-0000)	Barallat et al., 2020[50]	n=1821	Physicians and Surgeons, All Other	Cross-sectional survey	05/04 - 05/22	Spain	11.81%	High
Healthcare Practitioners and Technical Occupations (29-0000)	Bianchi et al., 2020[133]	n=34	Physicians and Surgeons, All Other	Cross-sectional survey	04/15 - 05/15	Italy	5.88%	Unclear
Healthcare Practitioners and Technical Occupations (29-0000)	Blairon et al., 2020[52]	n=323	Physicians and Surgeons, All Other	Cross-sectional survey	05/25 - 06/19	Belgium	11.8%	High
Healthcare Practitioners and Technical Occupations (29-0000)	Brehm et al., 2020[7]	n=275	Physicians and Surgeons, All Other	Cross sectional study with prospective cohort follow up of a	03/20 - 07/17	Germany	3.3%	Moderate

For peer review only

				subset of the sample				
Healthcare Practitioners and Technical Occupations (29-0000)	Brousseau et al., 2020[134]	n=432	Physicians and Surgeons, All Other	Cross-sectional survey	07/06 - 09/24	Canada	7.2%	High
Healthcare Practitioners and Technical Occupations (29-0000)	Calcagno et al., 2020[124]	n=700	Physicians and Surgeons, All Other	Cross-sectional survey	04/17 - 05/20	Italy	7.86%	Moderate
Healthcare Practitioners and Technical Occupations (29-0000)	Chau et al., 2020[126]	n=64	Physicians and Surgeons, All Other	Cross-sectional survey	08/23 - 08/30	Viet Nam	0%	High
Healthcare Practitioners and Technical Occupations (29-0000)	Chen et al., 2020[135]	n=17	Physicians and Surgeons, All Other	Cross-sectional survey	02/19 - 02/19	China	41.18%	High
Healthcare Practitioners and Technical Occupations (29-0000)	Erber et al., 2020[31]	n=860	Physicians and Surgeons, All Other	Cross-sectional survey	04/14 - 05/29	Germany	1.63%	High
Healthcare Practitioners and Technical Occupations (29-0000)	Favara et al., 2020[136]	n=15	Physicians and Surgeons, All Other	Prospective cohort	06/01 - 06/07	The United Kingdom	13.33%	High
Healthcare Practitioners and Technical Occupations (29-0000)	Favara et al., 2020[19]	n=82	Physicians and Surgeons, All Other	Cross-sectional survey	07/13 - 07/13	The United Kingdom	10.9%	High

For peer review only

Healthcare Practitioners and Technical Occupations (29-0000)	Fujita et al., 2020[137]	n=42	Physicians and Surgeons, All Other	Cross-sectional survey	04/10 - 04/20	Japan	4.7%	High
Healthcare Practitioners and Technical Occupations (29-0000)	Galan et al., 2020[20]	n=564	Physicians and Surgeons, All Other	Cross-sectional survey	04/14 - 04/27	Spain	39.36%	High
Healthcare Practitioners and Technical Occupations (29-0000)	Godbout et al., 2020[138]	n=490	Physicians and Surgeons, All Other	Cross-sectional survey	07/27 - 10/02	United States of America	1.43%	High
Healthcare Practitioners and Technical Occupations (29-0000)	Goenka et al., 2020[25]	n=255	Physicians and Surgeons, All Other	Cross-sectional survey	07/12 - 08/23	India	3.92%	Moderate
Healthcare Practitioners and Technical Occupations (29-0000)	Goenka et al., 2020[26]	n=29	Physicians and Surgeons, All Other	Cross-sectional survey	08/01 - 08/31	India	20.69%	High
Healthcare Practitioners and Technical Occupations (29-0000)	Hanrath et al., 2020[32]	n=899	Physicians and Surgeons, All Other	Cross-sectional survey	05/29 - 07/06	The United Kingdom	7.01%	High
Healthcare Practitioners and Technical Occupations (29-0000)	Houlihan et al., 2020[139]	n=72	Physicians and Surgeons, All Other	Cross-sectional survey	03/26 - 04/08	The United Kingdom	22%	High
Healthcare Practitioners and	Hunter et al., 2020[21]	n=279	Physicians and Surgeons, All Other	Cross-sectional survey	04/29 - 05/08	United States of America	1.08%	High

For peer review only

Technical Occupations (29-0000)								
Healthcare Practitioners and Technical Occupations (29-0000)	Insua et al., 2020[140]	n=116	Physicians and Surgeons, All Other	Cross-sectional survey	06/08 - 06/09	Argentina	0.9% (0.1-5.5%)	High
Healthcare Practitioners and Technical Occupations (29-0000)	Iversen et al., 2020[8]	n=4698	Physicians and Surgeons, All Other	Cross-sectional survey	04/15 - 04/22	Denmark	4.07%	Low
Healthcare Practitioners and Technical Occupations (29-0000)	Iversen et al., 2020[8]	n=113	Physicians and Surgeons, All Other	Cross-sectional survey	04/15 - 04/22	Denmark	7.08%	Low
Healthcare Practitioners and Technical Occupations (29-0000)	Jeremias et al., 2020[70]	n=79	Physicians and Surgeons, All Other	Cross-sectional survey	03/01 - 04/30	United States of America	11.4%	High
Healthcare Practitioners and Technical Occupations (29-0000)	Kassem et al., 2020[72]	n=30	Physicians and Surgeons, All Other	Cross-sectional survey	06/01 - 06/14	Egypt	6.66%	High
Healthcare Practitioners and Technical Occupations (29-0000)	Kassem et al., 2020[72]	n=30	Physicians and Surgeons, All Other	Cross-sectional survey	06/01 - 06/14	Egypt	3.33%	High
Healthcare Practitioners and Technical	Kassem et al., 2020[72]	n=30	Physicians and Surgeons, All Other	Cross-sectional survey	06/01 - 06/14	Egypt	0%	High

For peer review only

Occupations (29-0000)								
Healthcare Practitioners and Technical Occupations (29-0000)	Kassem et al., 2020[72]	n=30	Physicians and Surgeons, All Other	Cross-sectional survey	06/01 - 06/14	Egypt	3.33%	High
Healthcare Practitioners and Technical Occupations (29-0000)	Khan et al., 2020[127]	n=980	Physicians and Surgeons, All Other	Cross-sectional survey	06/15 - 06/29	India	2.8% (1.9-4%)	Moderate
Healthcare Practitioners and Technical Occupations (29-0000)	Kohler et al., 2020[141]	n=268	Physicians and Surgeons, All Other	Cross-sectional survey	03/19 - 04/03	Switzerland	1.49%	High
Healthcare Practitioners and Technical Occupations (29-0000)	Kumar et al., 2020[142]	n=201	Physicians and Surgeons, All Other	Cross-sectional survey	06/01 - 06/30	India	7% (4.2-11.4%)	High
Healthcare Practitioners and Technical Occupations (29-0000)	Leidner et al., 2020[22]	n=1081	Physicians and Surgeons, All Other	Cross sectional study with prospective cohort follow up of a subset of the sample	04/08 - 05/22	United States of America	3.33%	High
Healthcare Practitioners and Technical Occupations (29-0000)	Lumley et al., 2020[9]	n=1859	Physicians and Surgeons, All Other	Prospective cohort	04/23 - 11/30	The United Kingdom	10.11%	Moderate
Healthcare Practitioners and Technical	Martin et al., 2020[23]	n=1243	Physicians and Surgeons, All Other	Cross-sectional survey	05/29 - 07/13	The United Kingdom	10.3%	Moderate

For peer review only

Occupations (29-0000)								
Healthcare Practitioners and Technical Occupations (29-0000)	Mesnil et al., 2020[143]	n=111	Physicians and Surgeons, All Other	Cross-sectional survey	06/08 - 06/22	France	11%	High
Healthcare Practitioners and Technical Occupations (29-0000)	Missaglia et al., 2020[144]	n=377	Physicians and Surgeons, All Other	Cross-sectional survey	04/01 - 04/30	Italy	14.9%	High
Healthcare Practitioners and Technical Occupations (29-0000)	Mohr et al., 2020[129]	n=272	Physicians and Surgeons, All Other	Cross-sectional survey	05/13 - 07/08	United States of America	2.94%	Moderate
Healthcare Practitioners and Technical Occupations (29-0000)	Moscola et al., 2020[89]	n=3746	Physicians and Surgeons, All Other	Cross-sectional survey	04/20 - 06/23	United States of America	8.7%	High
Healthcare Practitioners and Technical Occupations (29-0000)	Nishida et al., 2020[90]	n=149	Physicians and Surgeons, All Other	Cross-sectional survey	06/12 - 06/19	Japan	1.3% (0.37-4.8%)	Moderate
Healthcare Practitioners and Technical Occupations (29-0000)	Nishida et al., 2020[90]	n=46	Physicians and Surgeons, All Other	Cross-sectional survey	06/12 - 06/19	Japan	0%	Moderate
Healthcare Practitioners and Technical Occupations (29-0000)	Nishida et al., 2020[90]	n=40	Physicians and Surgeons, All Other	Cross-sectional survey	06/12 - 06/19	Japan	0%	Moderate

For peer review only

Healthcare Practitioners and Technical Occupations (29-0000)	Nishida et al., 2020[90]	n=59	Physicians and Surgeons, All Other	Cross-sectional survey	06/12 - 06/19	Japan	1.7% (0.3-9%)	Moderate
Healthcare Practitioners and Technical Occupations (29-0000)	Nishida et al., 2020[90]	n=925	Physicians and Surgeons, All Other	Cross-sectional survey	06/12 - 06/19	Japan	0.43% (0.17-1.1%)	Moderate
Healthcare Practitioners and Technical Occupations (29-0000)	Noor et al., 2020[130]	n=303	Physicians and Surgeons, All Other	Cross-sectional survey	07/13 - 07/15	Pakistan	19.8%	Moderate
Healthcare Practitioners and Technical Occupations (29-0000)	Orth-Holler et al., 2020[145]	n=377	Physicians and Surgeons, All Other	Cross-sectional survey	03/20 - 03/27	Austria	0.3% (0.01-1.5%)	High
Healthcare Practitioners and Technical Occupations (29-0000)	Plebani et al., 2020[146]	n=2337	Physicians and Surgeons, All Other	Cross-sectional survey	02/22 - 05/29	Italy	3.6% (2.8-4.4%)	High
Healthcare Practitioners and Technical Occupations (29-0000)	Rosser et al., 2020[33]	n=2533	Physicians and Surgeons, All Other	Cross-sectional survey	04/20 - 05/20	United States of America	1.07%	High
Healthcare Practitioners and Technical Occupations (29-0000)	Rudberg et al., 2020[147]	n=439	Physicians and Surgeons, All Other	Cross-sectional survey	04/14 - 05/08	Sweden	19.1%	Moderate
Healthcare Practitioners and	Schmidt et al., 2020[148]	n=34	Physicians and Surgeons, All Other	Cross-sectional survey	04/20 - 04/30	Germany	8.82%	High

For peer review only

Technical Occupations (29-0000)								
Healthcare Practitioners and Technical Occupations (29-0000)	Sotgiu et al., 2020[149]	n=115	Physicians and Surgeons, All Other	Cross-sectional survey	04/02 - 04/16	Italy	6.09%	High
Healthcare Practitioners and Technical Occupations (29-0000)	Venugopal et al., 2020[150]	n=157	Physicians and Surgeons, All Other	Cross-sectional survey	03/01 - 05/01	United States of America	25%	Moderate
Healthcare Practitioners and Technical Occupations (29-0000)	Yogo et al., 2020[36]	n=110	Physicians and Surgeons, All Other	Cross-sectional survey	05/20 - 06/08	United States of America	1.82%	High
Healthcare Practitioners and Technical Occupations (29-0000)	Brzostek et al., 2020[151]	n=998	Physician Assistants	Cross-sectional survey	04/17 - 05/07	United States of America	28.3%	Moderate
Healthcare Practitioners and Technical Occupations (29-0000)	Hoffmann et al., 2020[152]	n=156	Physician Assistants	Prospective cohort	07/01 - 07/31	Germany	1.3%	High
Healthcare Practitioners and Technical Occupations (29-0000)	Mohr et al., 2020[129]	n=156	Physician Assistants	Cross-sectional survey	05/13 - 07/08	United States of America	0.64%	Moderate
Healthcare Practitioners and Technical	Morcuende et al., 2020[88]	n=6	Physician Assistants	Cross-sectional survey	03/01 - 04/21	United States of America	9.43%	High

For peer review only

Occupations (29-0000)								
Healthcare Practitioners and Technical Occupations (29-0000)	Morcuende et al., 2020[88]	n=53	Physician Assistants	Cross-sectional survey	03/01 - 04/21	United States of America	9.43%	High
Healthcare Practitioners and Technical Occupations (29-0000)	Patel et al., 2020[153]	n=230	Physician Assistants	Prospective cohort	06/02 - 06/27	United States of America	3.48%	High
Healthcare Practitioners and Technical Occupations (29-0000)	Self et al., 2020[154]	n=919	Physician Assistants	Cross-sectional survey	04/03 - 06/19	United States of America	5.66%	Moderate
Healthcare Practitioners and Technical Occupations (29-0000)	Shah et al., 2020[155]	n=248	Physician Assistants	Cross-sectional survey	05/25 - 07/09	United States of America	0%	High
Healthcare Practitioners and Technical Occupations (29-0000)	Shah et al., 2020[155]	n=320	Physician Assistants	Cross-sectional survey	05/25 - 07/09	United States of America	0.63%	High
Healthcare Practitioners and Technical Occupations (29-0000)	Lumley et al., 2020[9]	n=386	Occupational Therapists	Prospective cohort	04/23 - 11/30	The United Kingdom	11.4%	Moderate
Healthcare Practitioners and Technical Occupations (29-0000)	Akinbami et al., 2020[46]	n=235	Physical Therapists	Cross-sectional survey	05/18 - 06/13	United States of America	10.6% (7-15.3%)	Moderate

For peer review only

Healthcare Practitioners and Technical Occupations (29-0000)	Brehm et al., 2020[7]	n=15	Physical Therapists	Cross sectional study with prospective cohort follow up of a subset of the sample	03/20 - 07/17	Germany	0%	Moderate
Healthcare Practitioners and Technical Occupations (29-0000)	Cooper et al., 2020[59]	n=84	Physical Therapists	Cross-sectional survey	06/10 - 08/07	The United Kingdom	10.71%	Moderate
Healthcare Practitioners and Technical Occupations (29-0000)	Costa et al., 2020[128]	n=159	Physical Therapists	Cross-sectional survey	05/14 - 05/28	Brazil	10.7%	Moderate
Healthcare Practitioners and Technical Occupations (29-0000)	Akinbami et al., 2020[46]	n=409	Respiratory Therapists	Cross-sectional survey	05/18 - 06/13	United States of America	8.3% (5.8-11.4%)	Moderate
Healthcare Practitioners and Technical Occupations (29-0000)	Brunner et al., 2020[54]	n=42	Respiratory Therapists	Cross-sectional survey	05/04 - 05/29	United States of America	0%	High
Healthcare Practitioners and Technical Occupations (29-0000)	Godbout et al., 2020[138]	n=25	Respiratory Therapists	Cross-sectional survey	07/27 - 10/02	United States of America	0%	High
Healthcare Practitioners and Technical Occupations (29-0000)	Hunter et al., 2020[21]	n=94	Respiratory Therapists	Cross-sectional survey	04/29 - 05/08	United States of America	0%	High

For peer review only

Healthcare Practitioners and Technical Occupations (29-0000)	Rosser et al., 2020[33]	n=135	Respiratory Therapists	Cross-sectional survey	04/20 - 05/20	United States of America	0%	High
Healthcare Practitioners and Technical Occupations (29-0000)	Self et al., 2020[154]	n=235	Respiratory Therapists	Cross-sectional survey	04/03 - 06/19	United States of America	4.26%	Moderate
Healthcare Practitioners and Technical Occupations (29-0000)	Yogo et al., 2020[36]	n=121	Respiratory Therapists	Cross-sectional survey	05/20 - 06/08	United States of America	0%	High
Healthcare Practitioners and Technical Occupations (29-0000)	Rosser et al., 2020[33]	n=253	Therapists, All Other	Cross-sectional survey	04/20 - 05/20	United States of America	1.58%	High
Healthcare Practitioners and Technical Occupations (29-0000)	Schmidt et al., 2020[148]	n=80	Therapists, All Other	Cross-sectional survey	04/20 - 04/30	Germany	3.75%	High
Healthcare Practitioners and Technical Occupations (29-0000)	Yogo et al., 2020[36]	n=22	Therapists, All Other	Cross-sectional survey	05/20 - 06/08	United States of America	4.55%	High
Healthcare Practitioners and Technical Occupations (29-0000)	Calcagno et al., 2020[124]	n=13	Veterinarians	Cross-sectional survey	04/17 - 05/20	Italy	0%	Moderate
Healthcare Practitioners and	Akinbami et al., 2020[46]	n=6426	Registered Nurses	Cross-sectional survey	05/18 - 06/13	United States of America	7.7% (7.1-8.4%)	Moderate

For peer review only

Technical Occupations (29-0000)								
Healthcare Practitioners and Technical Occupations (29-0000)	Alharbi et al., 2020[125]	n=70	Registered Nurses	Cross-sectional survey	04/18 - 06/17	Saudi Arabia	10%	High
Healthcare Practitioners and Technical Occupations (29-0000)	Alharbi et al., 2020[125]	n=9	Registered Nurses	Cross-sectional survey	04/18 - 06/17	Saudi Arabia	33.33%	High
Healthcare Practitioners and Technical Occupations (29-0000)	Alharbi et al., 2020[125]	n=76	Registered Nurses	Cross-sectional survey	04/18 - 06/17	Saudi Arabia	26.32%	High
Healthcare Practitioners and Technical Occupations (29-0000)	Alharbi et al., 2020[125]	n=21	Registered Nurses	Cross-sectional survey	04/18 - 06/17	Saudi Arabia	14.29%	High
Healthcare Practitioners and Technical Occupations (29-0000)	Alharbi et al., 2020[125]	n=43	Registered Nurses	Cross-sectional survey	04/18 - 06/17	Saudi Arabia	27.91%	High
Healthcare Practitioners and Technical Occupations (29-0000)	Amendola et al., 2020[47]	n=216	Registered Nurses	Cross-sectional survey	04/15 - 04/15	Italy	6.02%	High
Healthcare Practitioners and Technical	Bampoe et al., 2020[156]	n=52	Registered Nurses	Cross-sectional survey	05/11 - 06/05	The United Kingdom	13.5% (5.6-25.8%)	High

For peer review only

Occupations (29-0000)								
Healthcare Practitioners and Technical Occupations (29-0000)	Bampoe et al., 2020[156]	n=40	Registered Nurses	Cross-sectional survey	05/11 - 06/05	The United Kingdom	12.5% (4.2-26.8%)	High
Healthcare Practitioners and Technical Occupations (29-0000)	Baracco et al., 2020[24]	n=1014	Registered Nurses	Cross-sectional survey	04/23 - 05/05	Italy	17.9%	High
Healthcare Practitioners and Technical Occupations (29-0000)	Barallat et al., 2020[50]	n=2243	Registered Nurses	Cross-sectional survey	05/04 - 05/22	Spain	10.64%	High
Healthcare Practitioners and Technical Occupations (29-0000)	Brehm et al., 2020[7]	n=444	Registered Nurses	Cross sectional study with prospective cohort follow up of a subset of the sample	03/20 - 07/17	Germany	2.3%	Moderate
Healthcare Practitioners and Technical Occupations (29-0000)	Brousseau et al., 2020[134]	n=1189	Registered Nurses	Cross-sectional survey	07/06 - 09/24	Canada	11.9%	High
Healthcare Practitioners and Technical Occupations (29-0000)	Calcagno et al., 2020[124]	n=1833	Registered Nurses	Cross-sectional survey	04/17 - 05/20	Italy	8.18%	Moderate
Healthcare Practitioners and Technical	Chau et al., 2020[126]	n=144	Registered Nurses	Cross-sectional survey	08/23 - 08/30	Viet Nam	0%	High

For peer review only

Occupations (29-0000)								
Healthcare Practitioners and Technical Occupations (29-0000)	Chen et al., 2020[135]	n=25	Registered Nurses	Cross-sectional survey	02/19 - 02/19	China	8%	High
Healthcare Practitioners and Technical Occupations (29-0000)	Cooper et al., 2020[59]	n=3471	Registered Nurses	Cross-sectional survey	06/10 - 08/07	The United Kingdom	7.52%	Moderate
Healthcare Practitioners and Technical Occupations (29-0000)	Costa et al., 2020[128]	n=370	Registered Nurses	Cross-sectional survey	05/14 - 05/28	Brazil	11.4%	Moderate
Healthcare Practitioners and Technical Occupations (29-0000)	Dimcheff et al., 2020[157]	n=412	Registered Nurses	Cross-sectional survey	06/08 - 07/08	United States of America	7%	Moderate
Healthcare Practitioners and Technical Occupations (29-0000)	Erber et al., 2020[31]	n=958	Registered Nurses	Cross-sectional survey	04/14 - 05/29	Germany	2.5%	High
Healthcare Practitioners and Technical Occupations (29-0000)	Favara et al., 2020[136]	n=45	Registered Nurses	Prospective cohort	06/01 - 06/07	The United Kingdom	28.89%	High
Healthcare Practitioners and Technical Occupations (29-0000)	Favara et al., 2020[19]	n=237	Registered Nurses	Cross-sectional survey	07/13 - 07/13	The United Kingdom	16.5%	High

For peer review only

Healthcare Practitioners and Technical Occupations (29-0000)	Finkenzeller et al., 2020[158]	n=251	Registered Nurses	Prospective cohort	06/29 - 07/29	Germany	12%	Moderate
Healthcare Practitioners and Technical Occupations (29-0000)	Finkenzeller et al., 2020[158]	n=887	Registered Nurses	Prospective cohort	06/29 - 07/29	Germany	20%	Moderate
Healthcare Practitioners and Technical Occupations (29-0000)	Fujita et al., 2020[137]	n=50	Registered Nurses	Cross-sectional survey	04/10 - 04/20	Japan	6%	High
Healthcare Practitioners and Technical Occupations (29-0000)	Galan et al., 2020[20]	n=687	Registered Nurses	Cross-sectional survey	04/14 - 04/27	Spain	30.71%	High
Healthcare Practitioners and Technical Occupations (29-0000)	Godbout et al., 2020[138]	n=937	Registered Nurses	Cross-sectional survey	07/27 - 10/02	United States of America	1.39%	High
Healthcare Practitioners and Technical Occupations (29-0000)	Goenka et al., 2020[25]	n=224	Registered Nurses	Cross-sectional survey	07/12 - 08/23	India	9.38%	Moderate
Healthcare Practitioners and Technical Occupations (29-0000)	Goenka et al., 2020[26]	n=43	Registered Nurses	Cross-sectional survey	08/01 - 08/31	India	34.88%	High
Healthcare Practitioners and	Grant et al., 2020[159]	n=1345	Registered Nurses	Cross-sectional survey	05/15 - 06/05	The United Kingdom	34.7%	High

For peer review only

Technical Occupations (29-0000)								
Healthcare Practitioners and Technical Occupations (29-0000)	Grant et al., 2020[159]	n=108	Registered Nurses	Cross-sectional survey	05/15 - 06/05	The United Kingdom	25%	High
Healthcare Practitioners and Technical Occupations (29-0000)	Hanrath et al., 2020[32]	n=749	Registered Nurses	Cross-sectional survey	05/29 - 07/06	The United Kingdom	8.99%	High
Healthcare Practitioners and Technical Occupations (29-0000)	Haq et al., 2020[67]	n=209	Registered Nurses	Cross-sectional survey	06/15 - 06/29	Pakistan	38.8% (32.1-45.7%)	Moderate
Healthcare Practitioners and Technical Occupations (29-0000)	Houlihan et al., 2020[139]	n=106	Registered Nurses	Cross-sectional survey	03/26 - 04/08	The United Kingdom	24%	High
Healthcare Practitioners and Technical Occupations (29-0000)	Houlihan et al., 2020[139]	n=22	Registered Nurses	Cross-sectional survey	03/26 - 04/08	The United Kingdom	23%	High
Healthcare Practitioners and Technical Occupations (29-0000)	Hunter et al., 2020[21]	n=317	Registered Nurses	Cross-sectional survey	04/29 - 05/08	United States of America	2.2%	High
Healthcare Practitioners and Technical	Iversen et al., 2020[8]	n=9963	Registered Nurses	Cross-sectional survey	04/15 - 04/22	Denmark	4.03%	Low

For peer review only

Occupations (29-0000)								
Healthcare Practitioners and Technical Occupations (29-0000)	Iversen et al., 2020[8]	n=1786	Registered Nurses	Cross-sectional survey	04/15 - 04/22	Denmark	4.65%	Low
Healthcare Practitioners and Technical Occupations (29-0000)	Jeremias et al., 2020[70]	n=1043	Registered Nurses	Cross-sectional survey	03/01 - 04/30	United States of America	9.5%	High
Healthcare Practitioners and Technical Occupations (29-0000)	Jones et al., 2020[29]	n=1962	Registered Nurses	Cross-sectional survey	01/15 - 06/15	The United Kingdom	10.5%	High
Healthcare Practitioners and Technical Occupations (29-0000)	Kassem et al., 2020[72]	n=28	Registered Nurses	Cross-sectional survey	06/01 - 06/14	Egypt	10.71%	High
Healthcare Practitioners and Technical Occupations (29-0000)	Kassem et al., 2020[72]	n=28	Registered Nurses	Cross-sectional survey	06/01 - 06/14	Egypt	7.14%	High
Healthcare Practitioners and Technical Occupations (29-0000)	Kassem et al., 2020[72]	n=28	Registered Nurses	Cross-sectional survey	06/01 - 06/14	Egypt	3.57%	High
Healthcare Practitioners and Technical Occupations (29-0000)	Kassem et al., 2020[72]	n=28	Registered Nurses	Cross-sectional survey	06/01 - 06/14	Egypt	0%	High

For peer review only

Healthcare Practitioners and Technical Occupations (29-0000)	Khan et al., 2020[127]	n=321	Registered Nurses	Cross-sectional survey	06/15 - 06/29	India	2.8% (1.5-5.3%)	Moderate
Healthcare Practitioners and Technical Occupations (29-0000)	Kohler et al., 2020[141]	n=398	Registered Nurses	Cross-sectional survey	03/19 - 04/03	Switzerland	0.75%	High
Healthcare Practitioners and Technical Occupations (29-0000)	Kumar et al., 2020[142]	n=308	Registered Nurses	Cross-sectional survey	06/01 - 06/30	India	6.8% (4.5-10.2%)	High
Healthcare Practitioners and Technical Occupations (29-0000)	Leidner et al., 2020[22]	n=110	Registered Nurses	Cross sectional study with prospective cohort follow up of a subset of the sample	04/08 - 05/22	United States of America	0%	High
Healthcare Practitioners and Technical Occupations (29-0000)	Leidner et al., 2020[22]	n=3504	Registered Nurses	Cross sectional study with prospective cohort follow up of a subset of the sample	04/08 - 05/22	United States of America	2.34%	High
Healthcare Practitioners and Technical Occupations (29-0000)	Lumley et al., 2020[9]	n=4528	Registered Nurses	Prospective cohort	04/23 - 11/30	The United Kingdom	13.21%	Moderate
Healthcare Practitioners and Technical Occupations (29-0000)	Mansour et al., 2020[160]	n=285	Registered Nurses	Cross-sectional survey	03/24 - 04/04	United States of America	32.63%	High

For peer review only

Healthcare Practitioners and Technical Occupations (29-0000)	Martin et al., 2020[161]	n=580	Registered Nurses	Cross-sectional survey	04/01 - 04/15	Spain	5.52%	High
Healthcare Practitioners and Technical Occupations (29-0000)	Martin et al., 2020[161]	n=74	Registered Nurses	Cross-sectional survey	04/01 - 04/15	Spain	9.46%	High
Healthcare Practitioners and Technical Occupations (29-0000)	Martin et al., 2020[161]	n=676	Registered Nurses	Cross-sectional survey	04/01 - 04/15	Spain	5.92%	High
Healthcare Practitioners and Technical Occupations (29-0000)	Martin et al., 2020[161]	n=337	Registered Nurses	Cross-sectional survey	04/01 - 04/15	Spain	5.93%	High
Healthcare Practitioners and Technical Occupations (29-0000)	Martin et al., 2020[161]	n=339	Registered Nurses	Cross-sectional survey	04/01 - 04/15	Spain	5.9%	High
Healthcare Practitioners and Technical Occupations (29-0000)	Meissner et al., 2020[162]	n=439	Registered Nurses	Cross-sectional survey	04/14 - 05/06	United States of America	1.37%	High
Healthcare Practitioners and Technical Occupations (29-0000)	Mohr et al., 2020[129]	n=410	Registered Nurses	Cross-sectional survey	05/13 - 07/08	United States of America	1.46%	Moderate
Healthcare Practitioners and	Moscola et al., 2020[89]	n=11468	Registered Nurses	Cross-sectional survey	04/20 - 06/23	United States of America	13.1%	High

For peer review only

Technical Occupations (29-0000)								
Healthcare Practitioners and Technical Occupations (29-0000)	Mostafa et al., 2020[163]	n=4040	Registered Nurses	Cross-sectional survey	04/22 - 05/14	Egypt	1.31%	High
Healthcare Practitioners and Technical Occupations (29-0000)	Nishida et al., 2020[90]	n=489	Registered Nurses	Cross-sectional survey	06/12 - 06/19	Japan	0.2% (0.04-1.1%)	Moderate
Healthcare Practitioners and Technical Occupations (29-0000)	Noor et al., 2020[130]	n=460	Registered Nurses	Cross-sectional survey	07/13 - 07/15	Pakistan	39.78%	Moderate
Healthcare Practitioners and Technical Occupations (29-0000)	Paradiso et al., 2020[164]	n=606	Registered Nurses	Cross sectional study with prospective cohort follow up of a subset of the sample	03/26 - 04/17	Italy	0.33%	High
Healthcare Practitioners and Technical Occupations (29-0000)	Plebani et al., 2020[146]	n=3230	Registered Nurses	Cross-sectional survey	02/22 - 05/29	Italy	4.7% (4-5.5%)	High
Healthcare Practitioners and Technical Occupations (29-0000)	Poustchi et al., 2020[28]	n=1245	Registered Nurses	Cross-sectional survey	04/17 - 06/02	Iran (Islamic Republic of)	15.9% (13.9-18%)	Moderate
Healthcare Practitioners and Technical	Rudberg et al., 2020[147]	n=636	Registered Nurses	Cross-sectional survey	04/14 - 05/08	Sweden	21.9%	Moderate

For peer review only

Occupations (29-0000)								
Healthcare Practitioners and Technical Occupations (29-0000)	Schmidt et al., 2020[148]	n=154	Registered Nurses	Cross-sectional survey	04/20 - 04/30	Germany	0%	High
Healthcare Practitioners and Technical Occupations (29-0000)	Self et al., 2020[154]	n=1445	Registered Nurses	Cross-sectional survey	04/03 - 06/19	United States of America	5.05%	Moderate
Healthcare Practitioners and Technical Occupations (29-0000)	Siddiqui et al., 2020[2]	n=59	Registered Nurses	Prospective cohort	04/15 - 08/15	India	10.2%	High
Healthcare Practitioners and Technical Occupations (29-0000)	Siddiqui et al., 2020[2]	n=70	Registered Nurses	Prospective cohort	04/15 - 08/15	India	10%	High
Healthcare Practitioners and Technical Occupations (29-0000)	Sotgiu et al., 2020[149]	n=64	Registered Nurses	Cross-sectional survey	04/02 - 04/16	Italy	7.8% (1.2-14.4%)	High
Healthcare Practitioners and Technical Occupations (29-0000)	Sydney et al., 2020[165]	n=81	Registered Nurses	Cross-sectional survey	04/28 - 05/04	United States of America	18.52%	High
Healthcare Practitioners and Technical Occupations (29-0000)	Urbieta et al., 2020[132]	n=83	Registered Nurses	Cross-sectional survey	04/14 - 04/16	Spain	4.8%	High

For peer review only

Healthcare Practitioners and Technical Occupations (29-0000)	Urbieta et al., 2020[132]	n=23	Registered Nurses	Cross-sectional survey	04/14 - 04/16	Spain	8.7%	High
Healthcare Practitioners and Technical Occupations (29-0000)	Venugopal et al., 2020[150]	n=142	Registered Nurses	Cross-sectional survey	03/01 - 05/01	United States of America	28%	Moderate
Healthcare Practitioners and Technical Occupations (29-0000)	Yogo et al., 2020[36]	n=1129	Registered Nurses	Cross-sectional survey	05/20 - 06/08	United States of America	2.48%	High
Healthcare Practitioners and Technical Occupations (29-0000)	Yogo et al., 2020[36]	n=12	Registered Nurses	Cross-sectional survey	05/20 - 06/08	United States of America	0%	High
Healthcare Practitioners and Technical Occupations (29-0000)	Zhou et al., 2020[166]	n=2406	Registered Nurses	Cross-sectional survey	03/16 - 03/25	China	1.37%	Moderate
Healthcare Practitioners and Technical Occupations (29-0000)	Godbout et al., 2020[138]	n=141	Nurse Practitioners	Cross-sectional survey	07/27 - 10/02	United States of America	1.42%	High
Healthcare Practitioners and Technical Occupations (29-0000)	Dimcheff et al., 2020[157]	n=214	Nurse Practitioners	Cross-sectional survey	06/08 - 07/08	United States of America	3.7%	Moderate
Healthcare Practitioners and	Akinbami et al., 2020[46]	n=719	Health Technologists and Technicians	Cross-sectional survey	05/18 - 06/13	United States of America	4.2% (2.8-5.9%)	Moderate

For peer review only

Technical Occupations (29-0000)								
Healthcare Practitioners and Technical Occupations (29-0000)	Blairon et al., 2020[52]	n=61	Health Technologists and Technicians	Cross-sectional survey	05/25 - 06/19	Belgium	6.6%	High
Healthcare Practitioners and Technical Occupations (29-0000)	Yogo et al., 2020[36]	n=65	Health Technologists and Technicians	Cross-sectional survey	05/20 - 06/08	United States of America	4.62%	High
Healthcare Practitioners and Technical Occupations (29-0000)	Silva et al., 2020[34]	n=224	Clinical Laboratory Technologists and Technicians	Cross-sectional survey	06/05 - 07/31	Brazil	7.59%	High
Healthcare Practitioners and Technical Occupations (29-0000)	Costa et al., 2020[128]	n=66	Medical and Clinical Laboratory Technologists	Cross-sectional survey	05/14 - 05/28	Brazil	3%	Moderate
Healthcare Practitioners and Technical Occupations (29-0000)	Akinbami et al., 2020[46]	n=293	Medical and Clinical Laboratory Technicians	Cross-sectional survey	05/18 - 06/13	United States of America	3.4% (1.7-6.2%)	Moderate
Healthcare Practitioners and Technical Occupations (29-0000)	Akinbami et al., 2020[46]	n=365	Medical and Clinical Laboratory Technicians	Cross-sectional survey	05/18 - 06/13	United States of America	5.5% (3.4-8.3%)	Moderate
Healthcare Practitioners and Technical	Alharbi et al., 2020[125]	n=80	Medical and Clinical Laboratory Technicians	Cross-sectional survey	04/18 - 06/17	Saudi Arabia	20%	High

For peer review only

Occupations (29-0000)								
Healthcare Practitioners and Technical Occupations (29-0000)	Baracco et al., 2020[24]	n=256	Medical and Clinical Laboratory Technicians	Cross-sectional survey	04/23 - 05/05	Italy	12.1%	High
Healthcare Practitioners and Technical Occupations (29-0000)	Brehm et al., 2020[7]	n=105	Medical and Clinical Laboratory Technicians	Cross sectional study with prospective cohort follow up of a subset of the sample	03/20 - 07/17	Germany	0%	Moderate
Healthcare Practitioners and Technical Occupations (29-0000)	Calcagno et al., 2020[124]	n=216	Medical and Clinical Laboratory Technicians	Cross-sectional survey	04/17 - 05/20	Italy	6.94%	Moderate
Healthcare Practitioners and Technical Occupations (29-0000)	Calcagno et al., 2020[124]	n=157	Medical and Clinical Laboratory Technicians	Cross-sectional survey	04/17 - 05/20	Italy	11.46%	Moderate
Healthcare Practitioners and Technical Occupations (29-0000)	Chau et al., 2020[126]	n=33	Medical and Clinical Laboratory Technicians	Cross-sectional survey	08/23 - 08/30	Viet Nam	0%	High
Healthcare Practitioners and Technical Occupations (29-0000)	Galan et al., 2020[20]	n=192	Medical and Clinical Laboratory Technicians	Cross-sectional survey	04/14 - 04/27	Spain	21.35%	High
Healthcare Practitioners and Technical	Goenka et al., 2020[25]	n=72	Medical and Clinical Laboratory Technicians	Cross-sectional survey	07/12 - 08/23	India	15.28%	Moderate

For peer review only

Occupations (29-0000)								
Healthcare Practitioners and Technical Occupations (29-0000)	Haq et al., 2020[67]	n=32	Medical and Clinical Laboratory Technicians	Cross-sectional survey	06/15 - 06/29	Pakistan	50% (31.8-68.1%)	Moderate
Healthcare Practitioners and Technical Occupations (29-0000)	Iversen et al., 2020[8]	n=1292	Medical and Clinical Laboratory Technicians	Cross-sectional survey	04/15 - 04/22	Denmark	1.93%	Low
Healthcare Practitioners and Technical Occupations (29-0000)	Khan et al., 2020[127]	n=397	Medical and Clinical Laboratory Technicians	Cross-sectional survey	06/15 - 06/29	India	2.5% (1.4-4.6%)	Moderate
Healthcare Practitioners and Technical Occupations (29-0000)	Lumley et al., 2020[9]	n=452	Medical and Clinical Laboratory Technicians	Prospective cohort	04/23 - 11/30	The United Kingdom	8.63%	Moderate
Healthcare Practitioners and Technical Occupations (29-0000)	Nishida et al., 2020[90]	n=140	Medical and Clinical Laboratory Technicians	Cross-sectional survey	06/12 - 06/19	Japan	0%	Moderate
Healthcare Practitioners and Technical Occupations (29-0000)	Rosser et al., 2020[33]	n=225	Medical and Clinical Laboratory Technicians	Cross-sectional survey	04/20 - 05/20	United States of America	0.44%	High
Healthcare Practitioners and Technical Occupations (29-0000)	Iversen et al., 2020[8]	n=342	Radiologic Technologists	Cross-sectional survey	04/15 - 04/22	Denmark	3.51%	Low

For peer review only

Healthcare Practitioners and Technical Occupations (29-0000)	Martin et al., 2020[23]	n=241	Radiologic Technologists	Cross-sectional survey	05/29 - 07/13	The United Kingdom	9.96%	Moderate
Healthcare Practitioners and Technical Occupations (29-0000)	Akinbami et al., 2020[46]	n=1158	Emergency Medical Technicians and Paramedics	Cross-sectional survey	05/18 - 06/13	United States of America	5.2% (4-6.6%)	Moderate
Healthcare Practitioners and Technical Occupations (29-0000)	Buntinx et al., 2020[167]	n=10	Emergency Medical Technicians and Paramedics	Cross-sectional survey	04/14 - 04/16	Belgium	10%	High
Healthcare Practitioners and Technical Occupations (29-0000)	Haq et al., 2020[67]	n=157	Emergency Medical Technicians and Paramedics	Cross-sectional survey	06/15 - 06/29	Pakistan	42% (34.2-50.1%)	Moderate
Healthcare Practitioners and Technical Occupations (29-0000)	Iversen et al., 2020[8]	n=323	Emergency Medical Technicians and Paramedics	Cross-sectional survey	04/15 - 04/22	Denmark	4.95%	Low
Healthcare Practitioners and Technical Occupations (29-0000)	Mesnil et al., 2020[143]	n=212	Emergency Medical Technicians and Paramedics	Cross-sectional survey	06/08 - 06/22	France	11%	High
Healthcare Practitioners and Technical Occupations (29-0000)	Reuben et al., 2020[168]	n=10	Emergency Medical Technicians and Paramedics	Cross-sectional survey	05/28 - 07/15	United States of America	0%	High

For peer review only

Healthcare Practitioners and Technical Occupations (29-0000)	Saberian et al., 2020[169]	n=243	Emergency Medical Technicians and Paramedics	Cross-sectional survey	03/20 - 05/20	Iran (Islamic Republic of)	41.56%	High
Healthcare Practitioners and Technical Occupations (29-0000)	Self et al., 2020[154]	n=56	Emergency Medical Technicians and Paramedics	Cross-sectional survey	04/03 - 06/19	United States of America	5.36%	Moderate
Healthcare Practitioners and Technical Occupations (29-0000)	Tarabichi et al., 2020[170]	n=111	Emergency Medical Technicians and Paramedics	Cross-sectional survey	04/20 - 05/19	United States of America	5.41%	High
Healthcare Practitioners and Technical Occupations (29-0000)	Baracco et al., 2020[24]	n=188	Health Technologists and Technicians, All Other	Cross-sectional survey	04/23 - 05/05	Italy	13.8%	High
Healthcare Practitioners and Technical Occupations (29-0000)	Chau et al., 2020[126]	n=22	Health Technologists and Technicians, All Other	Cross-sectional survey	08/23 - 08/30	Viet Nam	0%	High
Healthcare Practitioners and Technical Occupations (29-0000)	Goenka et al., 2020[25]	n=99	Health Technologists and Technicians, All Other	Cross-sectional survey	07/12 - 08/23	India	12.12%	Moderate
Healthcare Practitioners and Technical Occupations (29-0000)	Goenka et al., 2020[26]	n=16	Health Technologists and Technicians, All Other	Cross-sectional survey	08/01 - 08/31	India	68.75%	High
Healthcare Support	Jeremias et al., 2020[70]	n=155	Healthcare Support Occupations	Cross-sectional survey	03/01 - 04/30	United States of America	5.8%	High

For peer review only

Occupations (31-0000)								
Healthcare Support Occupations (31-0000)	Ward et al., 2020[113]	n=979	Nursing, Psychiatric, and Home Health Aides	Cross-sectional survey	09/15 - 09/28	The United Kingdom	11.09% (8.96-13.59%)	Moderate
Healthcare Support Occupations (31-0000)	Ward et al., 2020[113]	n=257	Nursing, Psychiatric, and Home Health Aides	Cross-sectional survey	09/15 - 09/28	The United Kingdom	8.95%	Moderate
Healthcare Support Occupations (31-0000)	Vijh et al., 2020[171]	n=169	Nursing, Psychiatric, and Home Health Aides	Cross-sectional survey	05/04 - 05/14	Canada	26.63%	High
Healthcare Support Occupations (31-0000)	Akinbami et al., 2020[46]	n=641	Nursing Assistants	Cross-sectional survey	05/18 - 06/13	United States of America	12.8% (10.3-15.6%)	Moderate
Healthcare Support Occupations (31-0000)	Bampoe et al., 2020[156]	n=108	Nursing Assistants	Cross-sectional survey	05/11 - 06/05	The United Kingdom	15.7% (9.5-24%)	High
Healthcare Support Occupations (31-0000)	Baracco et al., 2020[24]	n=257	Nursing Assistants	Cross-sectional survey	04/23 - 05/05	Italy	22.2%	High
Healthcare Support Occupations (31-0000)	Barallat et al., 2020[50]	n=832	Nursing Assistants	Cross-sectional survey	05/04 - 05/22	Spain	13.94%	High
Healthcare Support Occupations (31-0000)	Bhattacharya et al., 2020[172]	n=121	Nursing Assistants	Cross-sectional survey	06/01 - 06/15	United States of America	1.65%	High
Healthcare Support	Brousseau et al., 2020[134]	n=132	Nursing Assistants	Cross-sectional survey	07/06 - 09/24	Canada	16.7%	High

For peer review only

Occupations (31-0000)								
Healthcare Support Occupations (31-0000)	Brunner et al., 2020[54]	n=95	Nursing Assistants	Cross-sectional survey	05/04 - 05/29	United States of America	1.05%	High
Healthcare Support Occupations (31-0000)	Brzostek et al., 2020[151]	n=570	Nursing Assistants	Cross-sectional survey	04/17 - 05/07	United States of America	39.5%	Moderate
Healthcare Support Occupations (31-0000)	Brzostek et al., 2020[151]	n=263	Nursing Assistants	Cross-sectional survey	04/17 - 05/07	United States of America	45.6%	Moderate
Healthcare Support Occupations (31-0000)	Calcagno et al., 2020[124]	n=476	Nursing Assistants	Cross-sectional survey	04/17 - 05/20	Italy	9.24%	Moderate
Healthcare Support Occupations (31-0000)	Costa et al., 2020[128]	n=553	Nursing Assistants	Cross-sectional survey	05/14 - 05/28	Brazil	10.5%	Moderate
Healthcare Support Occupations (31-0000)	Galan et al., 2020[20]	n=472	Nursing Assistants	Cross-sectional survey	04/14 - 04/27	Spain	33.26%	High
Healthcare Support Occupations (31-0000)	Garcia et al., 2020[173]	n=2424	Nursing Assistants	Cross-sectional survey	05/01 - 05/30	Spain	22.4%	High
Healthcare Support Occupations (31-0000)	Garcia et al., 2020[174]	n=2424	Nursing Assistants	Cross-sectional survey	05/01 - 05/30	Spain	22.4%	High
Healthcare Support	Hanrath et al., 2020[32]	n=1434	Nursing Assistants	Cross-sectional survey	05/29 - 07/06	The United Kingdom	11.44%	High

For peer review only

Occupations (31-0000)								
Healthcare Support Occupations (31-0000)	Iversen et al., 2020[8]	n=501	Nursing Assistants	Cross-sectional survey	04/15 - 04/22	Denmark	1.2%	Low
Healthcare Support Occupations (31-0000)	Khan et al., 2020[127]	n=624	Nursing Assistants	Cross-sectional survey	06/15 - 06/29	India	2.4% (1.5-4%)	Moderate
Healthcare Support Occupations (31-0000)	Mughal et al., 2020[175]	n=121	Nursing Assistants	Cross-sectional survey	05/14 - 05/19	United States of America	0.83%	High
Healthcare Support Occupations (31-0000)	Rao et al., 2020[176]	n=1000	Nursing Assistants	Cross-sectional survey	05/23 - 06/06	India	1%	Unclear
Healthcare Support Occupations (31-0000)	Rudberg et al., 2020[147]	n=428	Nursing Assistants	Cross-sectional survey	04/14 - 05/08	Sweden	25.5%	Moderate
Healthcare Support Occupations (31-0000)	Siddiqui et al., 2020[2]	n=28	Nursing Assistants	Prospective cohort	04/15 - 08/15	India	10.7%	High
Healthcare Support Occupations (31-0000)	Yogo et al., 2020[36]	n=154	Nursing Assistants	Cross-sectional survey	05/20 - 06/08	United States of America	3.24%	High
Healthcare Support Occupations (31-0000)	Brousseau et al., 2020[134]	n=201	Orderlies	Cross-sectional survey	07/06 - 09/24	Canada	17.9%	High
Healthcare Support	Kassem et al., 2020[72]	n=9	Orderlies	Cross-sectional survey	06/01 - 06/14	Egypt	0%	High

For peer review only

Occupations (31-0000)								
Healthcare Support Occupations (31-0000)	Kassem et al., 2020[72]	n=9	Orderlies	Cross-sectional survey	06/01 - 06/14	Egypt	33.33%	High
Healthcare Support Occupations (31-0000)	Kassem et al., 2020[72]	n=9	Orderlies	Cross-sectional survey	06/01 - 06/14	Egypt	11.11%	High
Healthcare Support Occupations (31-0000)	Kassem et al., 2020[72]	n=9	Orderlies	Cross-sectional survey	06/01 - 06/14	Egypt	22.22%	High
Healthcare Support Occupations (31-0000)	Hanrath et al., 2020[32]	n=122	Orderlies	Cross-sectional survey	05/29 - 07/06	The United Kingdom	9.02%	High
Healthcare Support Occupations (31-0000)	Lumley et al., 2020[9]	n=377	Orderlies	Prospective cohort	04/23 - 11/30	The United Kingdom	15.38%	Moderate
Healthcare Support Occupations (31-0000)	Rosser et al., 2020[33]	n=3959	Medical Assistants	Cross-sectional survey	04/20 - 05/20	United States of America	1.39%	High
Healthcare Support Occupations (31-0000)	Yogo et al., 2020[36]	n=106	Phlebotomists	Cross-sectional survey	05/20 - 06/08	United States of America	0%	High
Healthcare Support Occupations (31-0000)	Cavlek et al., 2020[56]	n=300	Healthcare Support Workers, All Other	Cross-sectional survey	04/25 - 05/24	Croatia	0.67%	High
Healthcare Support	Erber et al., 2020[31]	n=383	Healthcare Support Workers, All Other	Cross-sectional survey	04/14 - 05/29	Germany	2.34%	High

For peer review only

Occupations (31-0000)								
Healthcare Support Occupations (31-0000)	Khan et al., 2020[127]	n=141	Healthcare Support Workers, All Other	Cross-sectional survey	06/15 - 06/29	India	0%	Moderate
Protective Service Occupations (33-0000)	Shukla et al., 2020[177]	n=1713	Protective Service Occupations	Cross-sectional survey	04/24 - 05/21	United States of America	1.46%	Moderate
Protective Service Occupations (33-0000)	Martinez et al., 2020[121]	n=18	First-Line Supervisors of Fire Fighting and Prevention Workers	Cross-sectional survey	04/16 - 04/17	United States of America	0%	High
Protective Service Occupations (33-0000)	Martinez et al., 2020[121]	n=47	First-Line Supervisors of Fire Fighting and Prevention Workers	Cross-sectional survey	04/16 - 04/17	United States of America	14.89%	High
Protective Service Occupations (33-0000)	Martinez et al., 2020[121]	n=13	First-Line Supervisors of Fire Fighting and Prevention Workers	Cross-sectional survey	04/16 - 04/17	United States of America	7.69%	High
Protective Service Occupations (33-0000)	Akinbami et al., 2020[46]	n=330	Firefighters	Cross-sectional survey	05/18 - 06/13	United States of America	6.7% (4.2-9.9%)	Moderate
Protective Service Occupations (33-0000)	Gray et al., 2020[178]	n=132	Firefighters	Cross-sectional survey	05/01 - 05/31	United States of America	14%	High
Protective Service Occupations (33-0000)	Reuben et al., 2020[168]	n=62	Firefighters	Cross-sectional survey	05/28 - 07/15	United States of America	4.84%	High
Protective Service Occupations (33-0000)	Sabourin et al., 2020[35]	n=42	Firefighters	Cross-sectional survey	07/15 - 08/15	United States of America	2.38%	High
Protective Service Occupations (33-0000)	Tarabichi et al., 2020[170]	n=185	Firefighters	Cross-sectional survey	04/20 - 05/19	United States of America	5.41%	High

For peer review only

Protective Service Occupations (33-0000)	Martinez et al., 2020[121]	n=7	Fire Inspectors and Investigators	Cross-sectional survey	04/16 - 04/17	United States of America	14.29%	High
Protective Service Occupations (33-0000)	Akinbami et al., 2020[46]	n=785	Police and Sheriff's Patrol Officers	Cross-sectional survey	05/18 - 06/13	United States of America	4% (2.7-5.6%)	Moderate
Protective Service Occupations (33-0000)	Chughtai et al., 2020[179]	n=154	Police and Sheriff's Patrol Officers	Cross-sectional survey	05/20 - 05/30	Pakistan	15.6%	High
Protective Service Occupations (33-0000)	Gudo et al., 2020[65]	n=564	Police and Sheriff's Patrol Officers	Cross-sectional survey	06/17 - 06/30	Mozambique	6% (4-8%)	High
Protective Service Occupations (33-0000)	Gujski et al., 2020[180]	n=4026	Police and Sheriff's Patrol Officers	Cross-sectional survey	06/22 - 07/08	Poland	4.2%	Moderate
Protective Service Occupations (33-0000)	Halatoko et al., 2020[41]	n=196	Police and Sheriff's Patrol Officers	Cross-sectional survey	04/23 - 05/08	Togo	0%	High
Protective Service Occupations (33-0000)	Langa et al., 2020[181]	n=471	Police and Sheriff's Patrol Officers	Cross-sectional survey	09/28 - 10/09	Mozambique	1.5%	High
Protective Service Occupations (33-0000)	Macicame et al., 2020[182]	n=456	Police and Sheriff's Patrol Officers	Cross-sectional survey	09/14 - 09/30	Mozambique	4.39%	High
Protective Service Occupations (33-0000)	Mahomed et al., 2020[81]	n=554	Police and Sheriff's Patrol Officers	Cross-sectional survey	08/31 - 10/12	Mozambique	2.9%	High
Protective Service Occupations (33-0000)	Reuben et al., 2020[168]	n=220	Police and Sheriff's Patrol Officers	Cross-sectional survey	05/28 - 07/15	United States of America	3.64%	High
Protective Service Occupations (33-0000)	Sabourin et al., 2020[35]	n=125	Police and Sheriff's Patrol Officers	Cross-sectional survey	07/15 - 08/15	United States of America	4%	High

For peer review only

Protective Service Occupations (33-0000)	Shukla et al., 2020[177]	n=1643	Police and Sheriff's Patrol Officers	Cross-sectional survey	04/24 - 05/21	United States of America	1.52%	Moderate
Protective Service Occupations (33-0000)	Siddiqui et al., 2020[2]	n=27	Police and Sheriff's Patrol Officers	Prospective cohort	04/15 - 08/15	India	7.4%	High
Protective Service Occupations (33-0000)	Viegas et al., 2020[110]	n=559	Police and Sheriff's Patrol Officers	Cross-sectional survey	08/03 - 08/21	Mozambique	3.94%	High
Protective Service Occupations (33-0000)	Denyer et al., 2020[60]	n=38216	Security Guards	Cross-sectional survey	05/12 - 05/18	Japan	0.23%	Unclear
Protective Service Occupations (33-0000)	Mahumane et al., 2020[82]	n=407	Security Guards	Cross-sectional survey	11/02 - 11/17	Mozambique	4.9%	High
Protective Service Occupations (33-0000)	Siddiqui et al., 2020[2]	n=9	Security Guards	Prospective cohort	04/15 - 08/15	India	0%	High
Protective Service Occupations (33-0000)	Silva et al., 2020[34]	n=32	Security Guards	Cross-sectional survey	06/05 - 07/31	Brazil	34%	High
Protective Service Occupations (33-0000)	Thani et al., 2020[183]	n=61	Security Guards	Cross-sectional survey	07/26 - 09/09	Qatar	60.1%	Moderate
Food Preparation and Serving Related Occupations (35-0000)	Thani et al., 2020[183]	n=93	Food Preparation and Serving Related Occupations	Cross-sectional survey	07/26 - 09/09	Qatar	29.2%	Moderate
Food Preparation and Serving Related Occupations (35-0000)	Siddiqui et al., 2020[2]	n=8	Cooks, All Other	Prospective cohort	04/15 - 08/15	India	37.5%	High
Food Preparation and Serving	Brunner et al., 2020[54]	n=8	Food Preparation Workers	Cross-sectional survey	05/04 - 05/29	United States of America	0%	High

For peer review only

Related Occupations (35-0000)								
Healthcare Support Occupations (31-0000)	Rosser et al., 2020[33]	n=335	Healthcare Support Occupations	Cross-sectional survey	04/20 - 05/20	United States of America	3.58%	High
Food Preparation and Serving Related Occupations (35-0000)	Biggs et al., 2020[3]	n=24	Food Servers, Nonrestaurant	Cross-sectional survey	04/28 - 05/03	United States of America	4.17%	Moderate
Food Preparation and Serving Related Occupations (35-0000)	Leidner et al., 2020[22]	n=113	Food Servers, Nonrestaurant	Cross sectional study with prospective cohort follow up of a subset of the sample	04/08 - 05/22	United States of America	1.77%	High
Food Preparation and Serving Related Occupations (35-0000)	Hanrath et al., 2020[32]	n=340	Other Food Preparation and Serving Related Workers	Cross-sectional survey	05/29 - 07/06	The United Kingdom	8.53%	High
Building and Grounds Cleaning and Maintenance Occupations (37-0000)	Martin et al., 2020[23]	n=528	Building and Grounds Cleaning and Maintenance Occupations	Cross-sectional survey	05/29 - 07/13	The United Kingdom	8.14%	Moderate
Building and Grounds Cleaning and Maintenance Occupations (37-0000)	Brousseau et al., 2020[134]	n=102	Building Cleaning and Pest Control Workers	Cross-sectional survey	07/06 - 09/24	Canada	10.8%	High
Building and Grounds Cleaning and Maintenance	Chau et al., 2020[126]	n=42	Building Cleaning and Pest Control Workers	Cross-sectional survey	08/23 - 08/30	Viet Nam	0%	High

For peer review only

Occupations (37-0000)								
Building and Grounds Cleaning and Maintenance Occupations (37-0000)	Finkenzeller et al., 2020[158]	n=57	Building Cleaning and Pest Control Workers	Prospective cohort	06/29 - 07/29	Germany	19.3%	Moderate
Building and Grounds Cleaning and Maintenance Occupations (37-0000)	Chau et al., 2020[126]	n=6	Janitors and Cleaners, Except Maids and Housekeeping Cleaners	Cross-sectional survey	08/23 - 08/30	Viet Nam	0%	High
Building and Grounds Cleaning and Maintenance Occupations (37-0000)	Epstude et al., 2020[184]	n=45	Janitors and Cleaners, Except Maids and Housekeeping Cleaners	Cross-sectional survey	06/15 - 06/30	Germany	0%	High
Building and Grounds Cleaning and Maintenance Occupations (37-0000)	Thani et al., 2020[183]	n=105	Janitors and Cleaners, Except Maids and Housekeeping Cleaners	Cross-sectional survey	07/26 - 09/09	Qatar	54.5%	Moderate
Building and Grounds Cleaning and Maintenance Occupations (37-0000)	Brunner et al., 2020[54]	n=23	Maids and Housekeeping Cleaners	Cross-sectional survey	05/04 - 05/29	United States of America	0%	High
Building and Grounds Cleaning and Maintenance Occupations (37-0000)	Goenka et al., 2020[25]	n=226	Maids and Housekeeping Cleaners	Cross-sectional survey	07/12 - 08/23	India	26.11%	Moderate
Building and Grounds Cleaning and Maintenance Occupations (37-0000)	Goenka et al., 2020[26]	n=10	Maids and Housekeeping Cleaners	Cross-sectional survey	08/01 - 08/31	India	10%	High

For peer review only

Building and Grounds Cleaning and Maintenance Occupations (37-0000)	Hanrath et al., 2020[32]	n=515	Maids and Housekeeping Cleaners	Cross-sectional survey	05/29 - 07/06	The United Kingdom	13.2%	High
Building and Grounds Cleaning and Maintenance Occupations (37-0000)	Khan et al., 2020[127]	n=276	Maids and Housekeeping Cleaners	Cross-sectional survey	06/15 - 06/29	India	3.3% (1.7-6.2%)	Moderate
Building and Grounds Cleaning and Maintenance Occupations (37-0000)	Leidner et al., 2020[22]	n=137	Maids and Housekeeping Cleaners	Cross sectional study with prospective cohort follow up of a subset of the sample	04/08 - 05/22	United States of America	8.03%	High
Building and Grounds Cleaning and Maintenance Occupations (37-0000)	Moscola et al., 2020[89]	n=7314	Maids and Housekeeping Cleaners	Cross-sectional survey	04/20 - 06/23	United States of America	20.9%	High
Building and Grounds Cleaning and Maintenance Occupations (37-0000)	Shakiba et al., 2020[10]	n=159	Maids and Housekeeping Cleaners	Cross-sectional survey	04/11 - 04/19	Iran (Islamic Republic of)	25% (13.6-37.5%)	Moderate
Building and Grounds Cleaning and Maintenance Occupations (37-0000)	Shields et al., 2020[97]	n=29	Maids and Housekeeping Cleaners	Cross-sectional survey	04/24 - 04/25	The United Kingdom	34.5%	High
Building and Grounds Cleaning and Maintenance Occupations (37-0000)	Siddiqui et al., 2020[2]	n=46	Maids and Housekeeping Cleaners	Prospective cohort	04/15 - 08/15	India	21.7%	High

For peer review only

Personal Care and Service Occupations (39-0000)	Biggs et al., 2020[3]	n=10	Hairdressers, Hairstylists, and Cosmetologists	Cross-sectional survey	04/28 - 05/03	United States of America	10%	Moderate
Personal Care and Service Occupations (39-0000)	Biggs et al., 2020[3]	n=48	Childcare Workers	Cross-sectional survey	04/28 - 05/03	United States of America	0%	Moderate
Personal Care and Service Occupations (39-0000)	Chen et al., 2020[135]	n=11	Personal Care Aides	Cross-sectional survey	02/19 - 02/19	China	9.09%	High
Personal Care and Service Occupations (39-0000)	Galan et al., 2020[20]	n=337	Personal Care Aides	Cross-sectional survey	04/14 - 04/27	Spain	27.89%	High
Personal Care and Service Occupations (39-0000)	Galan et al., 2020[20]	n=168	Personal Care Aides	Cross-sectional survey	04/14 - 04/27	Spain	27.38%	High
Personal Care and Service Occupations (39-0000)	Godbout et al., 2020[138]	n=86	Personal Care Aides	Cross-sectional survey	07/27 - 10/02	United States of America	2.32%	High
Personal Care and Service Occupations (39-0000)	Hassan et al., 2020[185]	n=403	Personal Care Aides	Cross-sectional survey	05/11 - 06/17	Sweden	20.1%	High
Personal Care and Service Occupations (39-0000)	Kumar et al., 2020[142]	n=292	Personal Care Aides	Cross-sectional survey	06/01 - 06/30	India	18.5% (14.5-23.3%)	High
Personal Care and Service Occupations (39-0000)	Ladhani et al., 2020[186]	n=208	Personal Care Aides	Prospective cohort	04/10 - 04/13	The United Kingdom	75% (68.7-80.4%)	High

Personal Care and Service Occupations (39-0000)	Lindahl et al., 2020[187]	n=1005	Personal Care Aides	Cross-sectional survey	04/01 - 04/20	Sweden	22.9% (20.4-25.7%)	High
Personal Care and Service Occupations (39-0000)	Regan et al., 2020[188]	n=305	Personal Care Aides	Cross-sectional survey	04/15 - 05/06	United States of America	23.6%	Unclear
Personal Care and Service Occupations (39-0000)	Siddiqui et al., 2020[2]	n=5	Personal Care Aides	Prospective cohort	04/15 - 08/15	India	40%	High
Personal Care and Service Occupations (39-0000)	Venugopal et al., 2020[150]	n=72	Personal Care Aides	Cross-sectional survey	03/01 - 05/01	United States of America	28%	Moderate
Personal Care and Service Occupations (39-0000)	Viegas et al., 2020[110]	n=85	Personal Care Aides	Cross-sectional survey	08/03 - 08/21	Mozambique	1.18%	High
Sales and Related Occupations (41-0000)	Arnaldo et al., 2020[13]	n=928	Sales and Related Occupations	Cross-sectional survey	07/06 - 07/13	Mozambique	6.5%	High
Sales and Related Occupations (41-0000)	Arnaldo et al., 2020[48]	n=1123	Sales and Related Occupations	Cross-sectional survey	08/10 - 08/21	Mozambique	1.6%	High
Sales and Related Occupations (41-0000)	Langa et al., 2020[181]	n=871	Sales and Related Occupations	Cross-sectional survey	09/28 - 10/09	Mozambique	0.2%	High
Sales and Related Occupations (41-0000)	Mabunda et al., 2020[15]	n=1585	Sales and Related Occupations	Cross-sectional survey	09/21 - 10/02	Mozambique	8.3%	High
Sales and Related Occupations (41-0000)	Macicame et al., 2020[182]	n=1288	Sales and Related Occupations	Cross-sectional survey	09/14 - 09/30	Mozambique	4.97%	High

For peer review only

Sales and Related Occupations (41-0000)	Mahomed et al., 2020[81]	n=1556	Sales and Related Occupations	Cross-sectional survey	08/31 - 10/12	Mozambique	0.8%	High
Sales and Related Occupations (41-0000)	Mahumane et al., 2020[82]	n=643	Sales and Related Occupations	Cross-sectional survey	11/02 - 11/17	Mozambique	1.9%	High
Sales and Related Occupations (41-0000)	Arnaldo et al., 2020[14]	n=472	Sales and Related Occupations	Cross-sectional survey	11/16 - 11/21	Mozambique	6.8%	High
Sales and Related Occupations (41-0000)	Arnaldo et al., 2020[14]	n=460	Sales and Related Occupations	Cross-sectional survey	11/02 - 11/12	Mozambique	5.9%	High
Sales and Related Occupations (41-0000)	Mahomed et al., 2020[16]	n=517	Sales and Related Occupations	Cross-sectional survey	11/26 - 12/03	Mozambique	8.9%	High
Sales and Related Occupations (41-0000)	Mahomed et al., 2020[16]	n=1001	Sales and Related Occupations	Cross-sectional survey	11/07 - 11/21	Mozambique	4.5%	High
Sales and Related Occupations (41-0000)	Biggs et al., 2020[3]	n=19	Retail Sales Workers	Cross-sectional survey	04/28 - 05/03	United States of America	0%	Moderate
Sales and Related Occupations (41-0000)	Poustchi et al., 2020[28]	n=753	Cashiers	Cross-sectional survey	04/17 - 06/02	Iran (Islamic Republic of)	16.1% (12.9-19.2%)	Moderate
Sales and Related Occupations (41-0000)	Alali et al., 2020[189]	n=525	Cashiers	Cross-sectional survey	05/23 - 06/26	Kuwait	38.1% (34-42.3%)	High
Sales and Related Occupations (41-0000)	Denyer et al., 2020[60]	n=19075	Retail Salespersons	Cross-sectional survey	05/12 - 05/18	Japan	0.04%	Unclear
Sales and Related Occupations (41-0000)	Kern et al., 2020[73]	n=300	Retail Salespersons	Cross-sectional survey	04/09 - 04/16	Germany	0.33% (0.01-1.84%)	High

For peer review only

Sales and Related Occupations (41-0000)	Khan et al., 2020[45]	n=132	Retail Salespersons	Cross-sectional survey	07/01 - 07/15	India	5.3% (2.5-10.7%)	Moderate
Sales and Related Occupations (41-0000)	Thani et al., 2020[183]	n=171	Retail Salespersons	Cross-sectional survey	07/26 - 09/09	Qatar	40.3%	Moderate
Sales and Related Occupations (41-0000)	Siddiqui et al., 2020[2]	n=4	Sales Representatives, Wholesale and Manufacturing, Except Technical and Scientific Products	Prospective cohort	04/15 - 08/15	India	25%	High
Sales and Related Occupations (41-0000)	Biggs et al., 2020[3]	n=34	Real Estate Sales Agents	Cross-sectional survey	04/28 - 05/03	United States of America	0%	Moderate
Sales and Related Occupations (41-0000)	Gudo et al., 2020[65]	n=1493	Door-to-Door Sales Workers, News and Street Vendors, and Related Workers	Cross-sectional survey	06/17 - 06/30	Mozambique	10% (8-11%)	High
Sales and Related Occupations (41-0000)	Viegas et al., 2020[110]	n=1246	Door-to-Door Sales Workers, News and Street Vendors, and Related Workers	Cross-sectional survey	08/03 - 08/21	Mozambique	5.22%	High
Sales and Related Occupations (41-0000)	Shakiba et al., 2020[10]	n=46	Sales and Related Workers, All Other	Cross-sectional survey	04/11 - 04/19	Iran (Islamic Republic of)	8.7% (0.8-20%)	Moderate
Office and Administrative Support Occupations (43-0000)	Calcagno et al., 2020[124]	n=539	Office and Administrative Support Occupations	Cross-sectional survey	04/17 - 05/20	Italy	3.34%	Moderate
Office and Administrative Support Occupations (43-0000)	Costa et al., 2020[128]	n=120	Office and Administrative Support Occupations	Cross-sectional survey	05/14 - 05/28	Brazil	14.2%	Moderate

For peer review only

Office and Administrative Support Occupations (43-0000)	Rosser et al., 2020[33]	n=972	Office and Administrative Support Occupations	Cross-sectional survey	04/20 - 05/20	United States of America	1.34%	High
Office and Administrative Support Occupations (43-0000)	Tsitsilonis et al., 2020[12]	n=504	Office and Administrative Support Occupations	Cross-sectional survey	06/15 - 07/15	Greece	0.48% (0-2.37%)	Moderate
Office and Administrative Support Occupations (43-0000)	Khan et al., 2020[45]	n=37	Hotel, Motel, and Resort Desk Clerks	Cross-sectional survey	07/01 - 07/15	India	10.8% (4.1-25.5%)	Moderate
Office and Administrative Support Occupations (43-0000)	Brunner et al., 2020[54]	n=26	Receptionists and Information Clerks	Cross-sectional survey	05/04 - 05/29	United States of America	0%	High
Office and Administrative Support Occupations (43-0000)	Favara et al., 2020[136]	n=10	Receptionists and Information Clerks	Prospective cohort	06/01 - 06/07	The United Kingdom	0%	High
Office and Administrative Support Occupations (43-0000)	Moscola et al., 2020[89]	n=9645	Receptionists and Information Clerks	Cross-sectional survey	04/20 - 06/23	United States of America	12.6%	High
Office and Administrative Support Occupations (43-0000)	Biggs et al., 2020[3]	n=11	Shipping, Receiving, and Traffic Clerks	Cross-sectional survey	04/28 - 05/03	United States of America	18.18%	Moderate
Office and Administrative	Silva et al., 2020[34]	n=82	Stock Clerks and Order Fillers	Cross-sectional survey	06/05 - 07/31	Brazil	4.88%	High

For peer review only

Support Occupations (43-0000)								
Office and Administrative Support Occupations (43-0000)	Khan et al., 2020[45]	n=186	Secretaries and Administrative Assistants	Cross-sectional survey	07/01 - 07/15	India	3.8% (1.8-7.7%)	Moderate
Office and Administrative Support Occupations (43-0000)	Alemu et al., 2020[6]	n=48	Executive Secretaries and Executive Administrative Assistants	Cross-sectional survey	04/23 - 04/28	Ethiopia	2.1%	Moderate
Office and Administrative Support Occupations (43-0000)	Barallat et al., 2020[50]	n=1181	Executive Secretaries and Executive Administrative Assistants	Cross-sectional survey	05/04 - 05/22	Spain	6.52%	High
Office and Administrative Support Occupations (43-0000)	Lumley et al., 2020[9]	n=1557	Executive Secretaries and Executive Administrative Assistants	Prospective cohort	04/23 - 11/30	The United Kingdom	6.74%	Moderate
Office and Administrative Support Occupations (43-0000)	Reuben et al., 2020[168]	n=18	Executive Secretaries and Executive Administrative Assistants	Cross-sectional survey	05/28 - 07/15	United States of America	0%	High
Office and Administrative Support Occupations (43-0000)	Akinbami et al., 2020[46]	n=964	Medical Secretaries	Cross-sectional survey	05/18 - 06/13	United States of America	8% (6.4-9.9%)	Moderate
Office and Administrative Support	Alharbi et al., 2020[125]	n=8	Medical Secretaries	Cross-sectional survey	04/18 - 06/17	Saudi Arabia	25%	High

For peer review only

Occupations (43-0000)								
Office and Administrative Support Occupations (43-0000)	Dimcheff et al., 2020[157]	n=357	Medical Secretaries	Cross-sectional survey	06/08 - 07/08	United States of America	4.2%	Moderate
Office and Administrative Support Occupations (43-0000)	Erber et al., 2020[31]	n=557	Medical Secretaries	Cross-sectional survey	04/14 - 05/29	Germany	3.78%	High
Office and Administrative Support Occupations (43-0000)	Finkenzeller et al., 2020[158]	n=240	Medical Secretaries	Prospective cohort	06/29 - 07/29	Germany	7.1%	Moderate
Office and Administrative Support Occupations (43-0000)	Goenka et al., 2020[25]	n=75	Medical Secretaries	Cross-sectional survey	07/12 - 08/23	India	8%	Moderate
Office and Administrative Support Occupations (43-0000)	Goenka et al., 2020[25]	n=75	Medical Secretaries	Cross-sectional survey	07/12 - 08/23	India	8%	Moderate
Office and Administrative Support Occupations (43-0000)	Iversen et al., 2020[8]	n=2631	Medical Secretaries	Cross-sectional survey	04/15 - 04/22	Denmark	2.7%	Low
Office and Administrative Support Occupations (43-0000)	Leidner et al., 2020[22]	n=793	Medical Secretaries	Cross sectional study with prospective cohort follow up of a	04/08 - 05/22	United States of America	3.15%	High

				subset of the sample				
Office and Administrative Support Occupations (43-0000)	Mesnil et al., 2020[143]	n=184	Medical Secretaries	Cross-sectional survey	06/08 - 06/22	France	14.13%	High
Office and Administrative Support Occupations (43-0000)	Nishida et al., 2020[90]	n=98	Medical Secretaries	Cross-sectional survey	06/12 - 06/19	Japan	1% (0.18-5.6%)	Moderate
Office and Administrative Support Occupations (43-0000)	Noor et al., 2020[130]	n=91	Medical Secretaries	Cross-sectional survey	07/13 - 07/15	Pakistan	43.96%	Moderate
Office and Administrative Support Occupations (43-0000)	Thani et al., 2020[183]	n=82	Medical Secretaries	Cross-sectional survey	07/26 - 09/09	Qatar	31.6%	Moderate
Office and Administrative Support Occupations (43-0000)	Zhou et al., 2020[166]	n=505	Medical Secretaries	Cross-sectional survey	03/16 - 03/25	China	1.39%	Moderate
Office and Administrative Support Occupations (43-0000)	Chau et al., 2020[126]	n=20	Data Entry Keyers	Cross-sectional survey	08/23 - 08/30	Viet Nam	0%	High
Office and Administrative Support Occupations (43-0000)	Jones et al., 2020[29]	n=1233	Office Clerks, General	Cross-sectional survey	01/15 - 06/15	The United Kingdom	6.1%	High

For peer review only

Office and Administrative Support Occupations (43-0000)	Rosser et al., 2020[33]	n=218	Office Clerks, General	Cross-sectional survey	04/20 - 05/20	United States of America	0%	High
Office and Administrative Support Occupations (43-0000)	Satpati et al., 2020[27]	n=47	Office Clerks, General	Cross-sectional survey	07/26 - 08/08	India	4.26%	Moderate
Office and Administrative Support Occupations (43-0000)	Baracco et al., 2020[24]	n=194	Office and Administrative Support Workers, All Other	Cross-sectional survey	04/23 - 05/05	Italy	14.4%	High
Office and Administrative Support Occupations (43-0000)	Brzostek et al., 2020[151]	n=286	Office and Administrative Support Workers, All Other	Cross-sectional survey	04/17 - 05/07	United States of America	45.5%	Moderate
Office and Administrative Support Occupations (43-0000)	Kassem et al., 2020[72]	n=7	Office and Administrative Support Workers, All Other	Cross-sectional survey	06/01 - 06/14	Egypt	14.28%	High
Office and Administrative Support Occupations (43-0000)	Kassem et al., 2020[72]	n=7	Office and Administrative Support Workers, All Other	Cross-sectional survey	06/01 - 06/14	Egypt	0%	High
Office and Administrative Support Occupations (43-0000)	Kassem et al., 2020[72]	n=7	Office and Administrative Support Workers, All Other	Cross-sectional survey	06/01 - 06/14	Egypt	0%	High

For peer review only

Office and Administrative Support Occupations (43-0000)	Kassem et al., 2020[72]	n=7	Office and Administrative Support Workers, All Other	Cross-sectional survey	06/01 - 06/14	Egypt	14.28%	High
Farming, Fishing, and Forestry Occupations (45-0000)	Satpati et al., 2020[27]	n=53	Agricultural Workers	Cross-sectional survey	07/26 - 08/08	India	0%	Moderate
Farming, Fishing, and Forestry Occupations (45-0000)	Addetia et al., 2020[190]	n=120	Fishers and Related Fishing Workers	Retrospective cohort	05/01 - 05/31	United States of America	5%	High
Farming, Fishing, and Forestry Occupations (45-0000)	Arnaldo et al., 2020[13]	n=80	Fishers and Related Fishing Workers	Cross-sectional survey	07/06 - 07/13	Mozambique	5%	High
Construction and Extraction Occupations (47-0000)	Biggs et al., 2020[3]	n=42	Construction Trades Workers	Cross-sectional survey	04/28 - 05/03	United States of America	0%	Moderate
Installation, Maintenance, and Repair Occupations (49-0000)	Blairon et al., 2020[52]	n=134	Other Installation, Maintenance, and Repair Occupations	Cross-sectional survey	05/25 - 06/19	Belgium	16.4%	High
Production Occupations (51-0000)	Picon et al., 2020[191]	n=40	Butchers and Other Meat, Poultry, and Fish Processing Workers	Cross-sectional survey	06/13 - 06/17	Brazil	15%	Moderate
Production Occupations (51-0000)	Picon et al., 2020[191]	n=1087	Miscellaneous Food Processing Workers	Cross-sectional survey	06/13 - 06/17	Brazil	1.47%	Moderate
Production Occupations (51-0000)	Bontadi et al., 2020[192]	n=1267	Production Workers, All Other	Cross-sectional survey	04/11 - 04/29	Italy	1.58%	High

For peer review only

Production Occupations (51-0000)	Xu et al., 2020[193]	n=442	Production Workers, All Other	Cross-sectional survey	03/09 - 04/10	China	1.4% (0.6-2.9%)	High
Transportation and Material Moving Occupations (53-0000)	Arnaldo et al., 2020[13]	n=248	Transportation and Material Moving Occupations	Cross-sectional survey	07/06 - 07/13	Mozambique	4.8%	High
Transportation and Material Moving Occupations (53-0000)	Arnaldo et al., 2020[48]	n=367	Transportation and Material Moving Occupations	Cross-sectional survey	08/10 - 08/21	Mozambique	7.4%	High
Transportation and Material Moving Occupations (53-0000)	Arnaldo et al., 2020[14]	n=112	Transportation and Material Moving Occupations	Cross-sectional survey	11/16 - 11/21	Mozambique	16.1%	High
Transportation and Material Moving Occupations (53-0000)	Biggs et al., 2020[3]	n=14	Transportation and Material Moving Occupations	Cross-sectional survey	04/28 - 05/03	United States of America	0%	Moderate
Transportation and Material Moving Occupations (53-0000)	Gudo et al., 2020[65]	n=554	Transportation and Material Moving Occupations	Cross-sectional survey	06/17 - 06/30	Mozambique	3% (1-4%)	High
Transportation and Material Moving Occupations (53-0000)	Langa et al., 2020[181]	n=230	Transportation and Material Moving Occupations	Cross-sectional survey	09/28 - 10/09	Mozambique	0.4%	High
Transportation and Material Moving Occupations (53-0000)	Mabunda et al., 2020[15]	n=473	Transportation and Material Moving Occupations	Cross-sectional survey	09/21 - 10/02	Mozambique	8.7%	High
Transportation and Material Moving Occupations (53-0000)	Macicame et al., 2020[182]	n=282	Transportation and Material Moving Occupations	Cross-sectional survey	09/14 - 09/30	Mozambique	3.19%	High

For peer review only

Transportation and Material Moving Occupations (53-0000)	Mahomed et al., 2020[81]	n=334	Transportation and Material Moving Occupations	Cross-sectional survey	08/31 - 10/12	Mozambique	1.5%	High
Transportation and Material Moving Occupations (53-0000)	Mahumane et al., 2020[82]	n=287	Transportation and Material Moving Occupations	Cross-sectional survey	11/02 - 11/17	Mozambique	1%	High
Transportation and Material Moving Occupations (53-0000)	Thani et al., 2020[183]	n=435	Transportation and Material Moving Occupations	Cross-sectional survey	07/26 - 09/09	Qatar	53.4%	Moderate
Transportation and Material Moving Occupations (53-0000)	Halatoko et al., 2020[41]	n=212	Air Transportation Workers	Cross-sectional survey	04/23 - 05/08	Togo	0.9%	High
Transportation and Material Moving Occupations (53-0000)	Viegas et al., 2020[110]	n=623	Air Transportation Workers	Cross-sectional survey	08/03 - 08/21	Mozambique	2.25%	High
Transportation and Material Moving Occupations (53-0000)	Viegas et al., 2020[110]	n=362	Air Transportation Workers	Cross-sectional survey	08/03 - 08/21	Mozambique	3.31%	High
Transportation and Material Moving Occupations (53-0000)	Khan et al., 2020[127]	n=57	Ambulance Drivers and Attendants, Except Emergency Medical Technicians	Cross-sectional survey	06/15 - 06/29	India	3.5% (0.9-13.3%)	Moderate
Transportation and Material Moving Occupations (53-0000)	Martinez et al., 2020[121]	n=30	Heavy and Tractor-Trailer Truck Drivers	Cross-sectional survey	04/16 - 04/17	United States of America	16.67%	High
Transportation and Material Moving Occupations (53-0000)	Siddiqui et al., 2020[2]	n=9	Heavy and Tractor-Trailer Truck Drivers	Prospective cohort	04/15 - 08/15	India	11.1%	High

For peer review only

Transportation and Material Moving Occupations (53-0000)	Halatoko et al., 2020[41]	n=122	Taxi Drivers and Chauffeurs	Cross-sectional survey	04/23 - 05/08	Togo	0.8%	High
Transportation and Material Moving Occupations (53-0000)	Poustchi et al., 2020[28]	n=718	Taxi Drivers and Chauffeurs	Cross-sectional survey	04/17 - 06/02	Iran (Islamic Republic of)	14.1% (11.4-16.9%)	Moderate
Transportation and Material Moving Occupations (53-0000)	Alemu et al., 2020[6]	n=8	Parking Lot Attendants	Cross-sectional survey	04/23 - 04/28	Ethiopia	12.5%	Moderate
Transportation and Material Moving Occupations (53-0000)	Alemu et al., 2020[6]	n=110	Laborers and Freight, Stock, and Material Movers, Hand	Cross-sectional survey	04/23 - 04/28	Ethiopia	10%	Moderate
Transportation and Material Moving Occupations (53-0000)	Khan et al., 2020[45]	n=97	Laborers and Freight, Stock, and Material Movers, Hand	Cross-sectional survey	07/01 - 07/15	India	2.1% (0.5-7.9%)	Moderate
Transportation and Material Moving Occupations (53-0000)	Satpati et al., 2020[27]	n=63	Laborers and Freight, Stock, and Material Movers, Hand	Cross-sectional survey	07/26 - 08/08	India	12.7%	Moderate
Not employed (mixed)*	Carrat et al., 2020[4]	n=6295	Unemployed	Prospective cohort	05/04 - 06/23	France	4.9% (4.1-5.6%)	Moderate
Not employed (mixed)*	Carrat et al., 2020[4]	n=1457	Unemployed	Prospective cohort	05/04 - 06/23	France	8.3% (6.4-10%)	Moderate
Not employed (mixed)*	Carrat et al., 2020[4]	n=306	Unemployed	Prospective cohort	05/04 - 06/23	France	7.2% (2.3-11.1%)	Moderate
Not employed (mixed)*	Carrat et al., 2020[4]	n=125	Unemployed	Prospective cohort	05/04 - 06/23	France	3.8% (0.5-6.3%)	Moderate
Not employed (mixed)*	Carrat et al., 2020[4]	n=402	Unemployed	Prospective cohort	05/04 - 06/23	France	7.8% (4.7-10.4%)	Moderate

For peer review only

Not employed (mixed)*	Chamie et al., 2020[194]	n=230	Unemployed	Cross-sectional survey	04/25 - 04/28	United States of America	4.3%	Moderate
Not employed (mixed)*	McLaughlin et al., 2020[195]	n=241	Unemployed	Cross-sectional survey	05/04 - 05/19	United States of America	19.3% (14.6-24.5%)	Moderate
Not employed (mixed)*	Merkely et al., 2020[1]	n=1095	Unemployed	Cross-sectional survey	05/01 - 05/16	Hungary	0.43% (0.16-0.84%)	Moderate
Not employed (mixed)*	Munoz et al., 2020[196]	n=905	Unemployed	Cross-sectional survey	07/15 - 07/16	Argentina	20%	Moderate
Not employed (mixed)*	Richard et al., 2020[5]	n=549	Unemployed	Cross-sectional survey	04/06 - 06/30	Switzerland	6%	Low
Not employed (mixed)*	Satpati et al., 2020[27]	n=47	Unemployed	Cross-sectional survey	07/26 - 08/08	India	2.13%	Moderate
Not employed (mixed)*	Ward et al., 2020[113]	n=59369	Unemployed	Cross-sectional survey	09/15 - 09/28	The United Kingdom	3.35%	Moderate

1. Merkely B, Szabó AJ, Kosztin A, et al. Novel coronavirus epidemic in the Hungarian population, a cross-sectional nationwide survey to support the exit policy in Hungary. *GeroScience*. 2020;42(4):1063-1074. doi:10.1007/s11357-020-00226-9

2. Siddiqui S, Naushin S, Pradhan S, et al. SARS-CoV-2 antibody seroprevalence and stability in a tertiary care hospital-setting. *medRxiv*. Published online September 2020. doi:10.1101/2020.09.02.20186486

3. Biggs HM, Harris JB, Breakwell L, et al. Estimated Community Seroprevalence of SARS-CoV-2 Antibodies Two Georgia Counties, April 28May 3, 2020. *MMWR Morbidity and Mortality Weekly Report*. 2020;69(29):965-970. doi:10.15585/mmwr.mm6929e2

4. Carrat F, Lamballerie X de, Rahib D, et al. Seroprevalence of SARS-CoV-2 among adults in three regions of France following the lockdown and associated risk factors: A multicohort study. *medRxiv*. Published online September 2020:2020.09.16.20195693. doi:10.1101/2020.09.16.20195693

5. Richard A, Wisniak A, Perez-Saez J, et al. Seroprevalence of anti-SARS-CoV-2 IgG antibodies, risk factors for infection and associated symptoms in Geneva, Switzerland: A population-based study. *medRxiv*. Published online December 2020. doi:10.1101/2020.12.16.20248180

6. Alemu BN, Addissie A, Mamo G, et al. *Sero-Prevalence of Anti-SARS-CoV-2 Antibodies in Addis Ababa, Ethiopia*. Microbiology; 2020. doi:10.1101/2020.10.13.337287

7. Brehm T, Schwinge D, Lampalzer S, et al. Seroprevalence of SARS-CoV-2 antibodies among hospital workers in a German tertiary care center: A sequential follow-up study. *International Journal of Hygiene and Environmental Health*. 2021;232:113671. doi:10.1016/j.ijheh.2020.113671

8. Iversen K, Bundgaard H, Hasselbalch RB, et al. Risk of COVID-19 in health-care workers in Denmark: An observational cohort study. *The Lancet Infectious diseases*. Published online August 2020. doi:10.1016/S1473-3099(20)30589-2

For peer review only

9. Lumley SF, O'Donnell D, Stoesser NE, et al. Antibody Status and Incidence of SARS-CoV-2 Infection in Health Care Workers. *New England Journal of Medicine*. Published online December 2020:NEJMoa2034545. doi:10.1056/NEJMoa2034545

10. Shakiba M, Nazemipour M, Salari A, et al. Seroprevalence of SARS-CoV-2 in Guilan Province, Iran, April 2020. *Emerging Infectious Disease journal*. 2021;27(2). doi:10.3201/eid2702.201960

11. Tilley K, Ayvazyan V, Martinez L, et al. A Cross-Sectional Study Examining the Seroprevalence of Severe Acute Respiratory Syndrome Coronavirus 2 Antibodies in a University Student Population. *Journal of Adolescent Health*. 2020;67(6):763-768. doi:10.1016/j.jadohealth.2020.09.001

12. Tsitsilonis OE, Paraskevis D, Lianidou E, et al. Seroprevalence of Antibodies against SARS-CoV-2 among the Personnel and Students of the National and Kapodistrian University of Athens, Greece: A Preliminary Report. *Life*. 2020;10(9):214. doi:10.3390/life10090214

13. Paulo Arnaldo. *Inquérito Sero-Epidemiológico de SARS-CoV-2 Na Cidade de Pemba (InCOVID 2020)*. República de Moçambique Ministério da Saúde; 2020.

14. Paulo Arnaldo. *Inquérito Sero-Epidemiológico de SARS-CoV-2 Nas Cidades de Xai-Xai E Chókwè (InCOVID 2020)*. República de Moçambique Ministério da Saúde; 2020.

15. Nedio Mabunda. *Inquérito Sero-Epidemiológico de SARS-CoV-2 Na Cidade de Beira (InCOVID 2020)*. República de Moçambique Ministério da Saúde; 2020.

16. Mussagy Mahomed. *Inquérito Sero-Epidemiológico de SARS-CoV-2 Na Cidade de Maxixe E Vila de Massinga (InCOVID 2020)*. República de Moçambique Ministério da Saúde; 2020.

17. Payne DC, Smith-Jeffcoat SE, Nowak G, et al. SARS-CoV-2 Infections and Serologic Responses from a Sample of U.S. Navy Service Members - USS Theodore Roosevelt, April 2020. *MMWR Morbidity and mortality weekly report*. 2020;69(23):714-721. doi:10.15585/mmwr.mm6923e4

18. COVID-19 Serology Tests Still Show Low Antibody Rate of 0.07%. *KBS World Radio*.

19. Favara DM, McAdam K, Cooke A, et al. SARS-CoV-2 antigen and antibody prevalence among UK staff working with cancer patients during the COVID-19 pandemic. *medRxiv*. Published online September 2020:2020.09.18.20197590. doi:10.1101/2020.09.18.20197590

20. Galán MI, Velasco M, Casas ML, et al. Hospital-Wide SARS-CoV-2 seroprevalence in health care workers in a Spanish teaching hospital. *Enfermedades Infecciosas y Microbiología Clínica*. Published online December 2020:S0213005X20304183. doi:10.1016/j.eimc.2020.11.015

21. Hunter BR, Dbeibo L, Weaver CS, et al. Seroprevalence of severe acute respiratory coronavirus virus 2 (SARS-CoV-2) antibodies among healthcare workers with differing levels of coronavirus disease 2019 (COVID-19) patient exposure. *Infection Control & Hospital Epidemiology*. Published online August 2020:1-2. doi:10.1017/ice.2020.390

22. Leidner R, Frary A, Cramer J, et al. Longitudinal SARS-CoV-2 serosurveillance of over ten thousand health care workers in the Providence Oregon cohort. *medRxiv*. Published online August 2020:2020.08.16.20176107. doi:10.1101/2020.08.16.20176107

23. Martin CA, Patel P, Goss C, et al. Demographic and occupational determinants of anti-SARS-CoV-2 IgG seropositivity in hospital staff. *Journal of Public Health*. 2020;(fdaa199). doi:10.1093/pubmed/fdaa199

For peer review only

24. Baracco A, Perotti G, Filippin A, et al. *SARS-CoV-2 Antibody Prevalence in Health Care Workers of Lodi Hospital, the COVID-19 Italian Epicentre*. Social Science Research Network; 2020.

25. Goenka M, Afzalpurkar S, Goenka U, et al. Seroprevalence of COVID-19 Amongst Health Care Workers in a Tertiary Care Hospital of a Metropolitan City from India. *The Journal of the Association of Physicians of India*. 2020;68(11):14-19.

26. Goenka MK, Shah BB, Goenka U, et al. COVID-19 prevalence among health-care workers of Gastroenterology department: An audit from a tertiary-care hospital in India. *JGH Open*. 2021;5(1):56-63. doi:10.1002/jgh3.12447

27. Satpati P, Sarangi S, Gantait K, et al. *Sero-Surveillance (IgG) of SARS-CoV-2 Among Asymptomatic General Population of Paschim Medinipur, West Bengal, India*. Infectious Diseases (except HIV/AIDS); 2020. doi:10.1101/2020.09.12.20193219

28. Poustchi H, Darvishian M, Mohammadi Z, et al. SARS-CoV-2 antibody seroprevalence in the general population and high-risk occupational groups across 18 cities in Iran: A population-based cross-sectional study. *The Lancet Infectious Diseases*. 2020;0(0). doi:10.1016/S1473-3099(20)30858-6

29. Jones CR, Hamilton FW, Thompson A, Morris TT, Moran E. SARS-CoV-2 IgG seroprevalence in healthcare workers and other staff at North Bristol NHS Trust: A sociodemographic analysis. *Journal of Infection*. 2020;0(0). doi:10.1016/j.jinf.2020.11.036

30. Anna F, Goyard S, Lalanne AI, et al. High seroprevalence but short-lived immune response to SARS-CoV-2 infection in Paris. *medRxiv*. Published online November 2020:2020.10.25.20219030. doi:10.1101/2020.10.25.20219030

31. Erber J, Kappler V, Haller B, et al. Strategies for infection control and prevalence of anti-SARS-CoV-2 IgG in 4,554 employees of a university hospital in Munich, Germany. Published online October 2020. doi:10.1101/2020.10.04.20206136

32. Hanrath AT, Loeff IS van der, Lendrem DW, et al. SARS-CoV-2 testing of 11,884 healthcare workers at an acute NHS hospital trust in England: A retrospective analysis. *medRxiv*. Published online December 2020:2020.12.22.20242362. doi:10.1101/2020.12.22.20242362

33. Rosser JI, Röltgen K, Dymock M, et al. Severe acute respiratory coronavirus virus 2 (SARS-CoV-2) seroprevalence in healthcare personnel in northern California early in the coronavirus disease 2019 (COVID-19) pandemic. *Infection Control & Hospital Epidemiology*. Published online December 2020:1-7. doi:10.1017/ice.2020.1358

34. Silva VO, de Oliveira EL, Castejon MJ, et al. Prevalence of antibodies against sars-cov-2 in professionals of a public health laboratory at são paulo, sp, brazil. *medRxiv*. Published online October 2020. doi:10.1101/2020.10.19.20213421

35. Sabourin KR, Schultz J, Romero J, et al. Risk Factors of SARS-CoV-2 Antibodies in Arapahoe County First Responders - the COVID-19 Arapahoe SErosurveillance Study (CASES) Project. *Journal of Occupational and Environmental Medicine*. Published online December 2020. doi:10.1097/JOM.0000000000002099

36. Yogo N, Greenwood KL, Thompson L, et al. Point prevalence survey to evaluate the seropositivity for coronavirus disease 2019 (COVID-19) among high-risk healthcare workers. *Infection Control and Hospital Epidemiology*. Published online December 2020:1-6. doi:10.1017/ice.2020.1370

37. Figueiredo-Campos P, Blankenhaus B, Mota C, et al. Seroprevalence of anti-SARS-CoV-2 antibodies in COVID-19 patients and healthy volunteers up to 6 months post disease onset. *European Journal of Immunology*. 2020;50(12):2025-2040. doi:10.1002/eji.202048970

For peer review only

38. Gonçalves J, Sousa RL, Jacinto MJ, et al. Evaluating SARS-CoV-2 Seroconversion Following Relieve of Confinement Measures. *Frontiers in Medicine*. 2020;7. doi:10.3389/fmed.2020.603996

39. Fontanet A, Grant R, Tondeur L, et al. SARS-CoV-2 infection in primary schools in northern France: A retrospective cohort study in an area of high transmission. *medRxiv*. Published online June 2020:2020.06.25.20140178. doi:10.1101/2020.06.25.20140178

40. Torres JP, Piñera C, De La Maza V, et al. Severe Acute Respiratory Syndrome Coronavirus 2 Antibody Prevalence in Blood in a Large School Community Subject to a Coronavirus Disease 2019 Outbreak: A Cross-sectional Study. *Clinical Infectious Diseases*. Published online July 2020:ciaa955. doi:10.1093/cid/ciaa955

41. Halatoko WA, KONU YR, Gbeasor-Komlanvi FA, et al. Prevalence of SARS-CoV-2 among high-risk populations in Lom&Eacute (Togo) in 2020. *medRxiv*. Published online August 2020:2020.08.07.20163840. doi:10.1101/2020.08.07.20163840

42. Slusser S. MLB antibody study: 0.7% of those tested had been exposed to coronavirus. *San Francisco Chronicle*. Published online May 2020.

43. Vince A, Zadro R, Šostar Z, et al. SARS-CoV-2 Seroprevalence in a Cohort of Asymptomatic, RT-PCR Negative Croatian First League Football Players. *medRxiv*. Published online November 2020:2020.10.30.20223230. doi:10.1101/2020.10.30.20223230

44. Mack D, Gärtner BC, Rössler A, et al. Prevalence of SARS-CoV-2 IgG antibodies in a large prospective cohort study of elite football players in Germany (MayJune 2020): Implications for a testing protocol in asymptomatic individuals and estimation of the rate of undetected cases. *Clinical Microbiology and Infection*. 2020;27(3):473.e1-473.e4. doi:10.1016/j.cmi.2020.11.033

45. Khan SMS, Qurieshi MA, Haq I, et al. Seroprevalence of SARS-CoV-2 specific IgG antibodies in District Srinagar, northern India A cross-sectional study. *PLOS ONE*. 2020;15(11):e0239303. doi:10.1371/journal.pone.0239303

46. Akinbami LJ, Vuong N, Petersen LR, et al. SARS-CoV-2 Seroprevalence among Healthcare, First Response, and Public Safety Personnel, Detroit Metropolitan Area, Michigan, USA, MayJune 2020 - Volume 26, Number 12December 2020 - Emerging Infectious Diseases journal - CDC. Published online December 2020. doi:10.3201/eid2612.203764

47. Amendola A, Tanzi E, Folgori L, et al. Low seroprevalence of SARS-CoV-2 infection among healthcare workers of the largest children hospital in Milan during the pandemic wave. *Infection Control & Hospital Epidemiology*. Published online August 2020:1-2. doi:10.1017/ice.2020.401

48. Paulo Arnaldo. *Inquérito Sero-Epidemiológico de SARS-CoV-2 Na Cidade de Quelimane (InCOVID 2020)*. República de Moçambique Ministério da Saúde; 2020.

49. Bal A, Brengel-Pesce K, Gaymard A, et al. Clinical and microbiological assessments of COVID-19 in healthcare workers: A prospective longitudinal study. *medRxiv*. Published online November 2020:2020.11.04.20225862. doi:10.1101/2020.11.04.20225862

50. Fernández-Rivas G, Quirant-Sánchez B, González V, et al. Seroprevalence of SARS-CoV-2 IgG Specific Antibodies among Healthcare Workers in the Northern Metropolitan Area of Barcelona, Spain, after the first pandemic wave. *medRxiv*. Published online June 2020:2020.06.24.20135673. doi:10.1101/2020.06.24.20135673

51. Bardai G, Ouellet J, Engelhardt T, Bertolizio G, Wu Z, Rauch F. Prevalence of SARS-CoV-2 infections in a pediatric orthopedic hospital. von Ungern-Sternberg B, ed. *Pediatric Anesthesia*. 2021;31(2):247-248. doi:10.1111/pan.14047

For peer review only

52. Blairon L, Mokrane S, Wilmet A, et al. Large-scale, molecular and serological SARS-CoV-2 screening of healthcare workers in a 4-site public hospital in Belgium after COVID-19 outbreak. *Journal of Infection*. Published online July 2020:S0163445320305144. doi:10.1016/j.jinf.2020.07.033

53. Moreno Borraz LA, Giménez López M, Carrera Lasfuentes P, et al. Prevalencia de infección por coronavirus SARS-CoV-2 en pacientes y profesionales de un hospital de media y larga estancia en España. *Revista Española de Geriatría y Gerontología*. 2020;56(2):75-80. doi:10.1016/j.regg.2020.10.005

54. Brunner WM, Hirabayashi L, Krupa NL, et al. Severe acute respiratory coronavirus virus 2 (SARS-CoV-2) IgG results among healthcare workers in a rural upstate New York hospital system. *Infection Control & Hospital Epidemiology*. Published online October 2020:1-4. doi:10.1017/ice.2020.1296

55. Carozzi FM, Cusi MG, Pistello M, et al. Detection of asymptomatic SARS-CoV-2 infections among healthcare workers: Results from a large-scale screening program based on rapid serological testing. *medRxiv*. Published online August 2020. doi:10.1101/2020.07.30.20149567

56. Vilibic-Cavlek T, Stevanovic V, Tabain I, et al. Severe acute respiratory syndrome coronavirus 2 seroprevalence among personnel in the healthcare facilities of Croatia, 2020. *Revista da Sociedade Brasileira de Medicina Tropical*. 2020;53. doi:10.1590/0037-8682-0458-2020

57. Chibwana MG, Jere KC, kamng'ona R, et al. High SARS-CoV-2 seroprevalence in Health Care Workers but relatively low numbers of deaths in urban Malawi. *medRxiv*. Published online August 2020:2020.07.30.20164970. doi:10.1101/2020.07.30.20164970

58. Coffman B. New Co-Immunity Project data show COVID-19 infection among health care workers may be lower than the general population | UofL News. *UofLNews*. Published online August 2020.

59. Cooper DJ, Lear S, Watson L, et al. A prospective study of risk factors associated with seroprevalence of SARS-CoV-2 antibodies in healthcare workers at a large UK teaching hospital. *medRxiv*. Published online November 2020:2020.11.03.20220699. doi:10.1101/2020.11.03.20220699

60. Denyer S. Japanese firm's blanket testing of employees could serve as model. *LMT Online*. Published online June 2020.

61. Dimeglio C, Herin F, Miedougé M, et al. Screening for SARS-CoV-2 antibodies among healthcare workers in a university hospital in southern France. *Journal of Infection*. 2020;0(0). doi:10.1016/j.jinf.2020.09.035

62. Fuereder T, Berghoff AS, Heller G, et al. SARS-CoV-2 seroprevalence in oncology healthcare professionals and patients with cancer at a tertiary care centre during the COVID-19 pandemic. *ESMO Open*. 2020;5(5). doi:10.1136/esmoopen-2020-000889

63. Fusco FM, Pisaturo M, Iodice V, et al. COVID-19 among healthcare workers in a specialist infectious diseases setting in Naples, Southern Italy: Results of a cross-sectional surveillance study. *Journal of Hospital Infection*. 2020;105(4):596-600. doi:10.1016/j.jhin.2020.06.021

64. Geraci L. Antibody tests show just 2% exposure rate to COVID-19. *The Lancaster News*. Published online May 2020.

65. Eduardo Samo Gudo. *Inquérito Sero-epidemiológico de SARS-CoV-2 na Cidade de Nampula*. República de Moçambique Ministério da Saúde; 2020:19.

66. Hackner K, Errhalt P, Willheim M, et al. Diagnostic accuracy of two commercially available rapid assays for detection of IgG and IgM antibodies to SARS-CoV-2 compared to ELISA in a low-prevalence population. *Research Square*. Published online August 2020. doi:10.21203/rs.3.rs-50887/v1

For peer review only

67. Haq M, Rehman A, Noor M, et al. Seroprevalence and Risk Factors of SARS CoV-2 in Health Care Workers of Tertiary-Care Hospitals in the Province of Khyber Pakhtunkhwa, Pakistan. *medRxiv*. Published online September 2020:2020.09.29.20203125. doi:10.1101/2020.09.29.20203125

68. He L, Zeng Y, Zeng C, et al. Positive Rate of Serology and RT-PCR for COVID-19 among healthcare workers during different periods in Wuhan, China. *Journal of Infection*. Published online August 2020. doi:10.1016/j.jinf.2020.08.027

69. Herzberg J, Vollmer T, Fischer B, et al. Prospective Sero-epidemiological Evaluation of SARS-CoV-2 among Health Care Workers in a German Secondary Care Hospital. *International Journal of Infectious Diseases*. 2021;102:136-143. doi:10.1016/j.ijid.2020.10.026

70. Jeremias A, Nguyen J, Levine J, et al. Prevalence of SARS-CoV-2 Infection Among Health Care Workers in a Tertiary Community Hospital. *JAMA Internal Medicine*. Published online August 2020. doi:10.1001/jamainternmed.2020.4214

71. Jespersen S, Mikkelsen S, Greve T, et al. Severe Acute Respiratory Syndrome Coronavirus 2 Seroprevalence Survey Among 17 971 Healthcare and Administrative Personnel at Hospitals, Prehospital Services, and Specialist Practitioners in the Central Denmark Region. *Clinical Infectious Diseases*. Published online October 2020:ciaa1471. doi:10.1093/cid/ciaa1471

72. Kassem AM, Talaat H, Shawky S, et al. SARS-CoV-2 infection among healthcare workers of a gastroenterological service in a tertiary care facility. *Arab Journal of Gastroenterology*. 2020;21(3):151-155. doi:10.1016/j.ajg.2020.07.005

73. Kern PM, Müller H-H, Menzel T, Weisser H. Studie zur Immunität gegen SARS-CoV-2: Keine signifikante humorale Immunität gegen SARS-CoV-2 im medizinischen Personal eines Klinikums der Maximalversorgung und in der Stadtregion Fulda. *Der Klinikarzt*. 2020;49(06):268-273. doi:10.1055/a-1198-1243

74. Khalil A, Hill R, Wright A, Ladhani S, O'Brien P. SARS-CoV-2-Specific Antibody Detection in Healthcare Workers in a UK Maternity Hospital: Correlation With SARS-CoV-2 RT-PCR Results. *Clinical Infectious Diseases*. 2020;(ciaa893). doi:10.1093/cid/ciaa893

75. Kumar A, Sathyapalan D, Ramachandran A, Subhash K, Biswas L, Beena KV. SARS-CoV-2 antibodies in healthcare workers in a large university hospital, Kerala, India. *Clinical Microbiology and Infection*. 2021;27(3):481-483. doi:10.1016/j.cmi.2020.09.013

76. Lackermair K, William F, Grzanna N, et al. Infection with SARS-CoV-2 in primary care health care workers assessed by antibody testing. *Family Practice*. Published online August 2020:cmaa078. doi:10.1093/fampra/cmaa078

77. Lahner E, Dilaghi E, Prestigiacomo C, et al. Prevalence of Sars-Cov-2 Infection in Health Workers (HWs) and Diagnostic Test Performance: The Experience of a Teaching Hospital in Central Italy. *International Journal of Environmental Research and Public Health*. 2020;17(12). doi:10.3390/ijerph17124417

78. Liu M, Cheng S-Z, Xu K-W, et al. Use of personal protective equipment against coronavirus disease 2019 by healthcare professionals in Wuhan, China: Cross sectional study. *BMJ*. 2020;369. doi:10.1136/bmj.m2195

79. Liu T, Wu S, Tao H, Zeng G, Zhou F, Wang X. Prevalence of IgG Antibodies to SARS-CoV-2 in Wuhan Implications for the Longevity of Antibodies Against SARS-CoV-2. *Research Square*. Published online November 2020. doi:10.21203/rs.3.rs-99748/v1

80. Lorenzo D, Carrisi C. COVID-19 exposure risk for family members of healthcare workers: An observational study. *International Journal of Infectious Diseases*. 2020;98:287-289. doi:10.1016/j.ijid.2020.06.106

For peer review only

81. Mussagy Mahomed. *Inquérito Sero-Epidemiológico de SARS-CoV-2 Na Cidade de Tete (InCOVID 2020)*. República de Moçambique Ministério da Saúde; 2020.

82. Arlete Mahumane. *Inquérito Sero-Epidemiológico de SARS-CoV-2 Na Cidade de Chimoio (InCOVID 2020)*. República de Moçambique Ministério da Saúde; 2020.

83. Majdoubi A, Michalski C, O'Connell SE, et al. Antibody reactivity to SARS-CoV-2 is common in unexposed adults and infants under 6 months. *medRxiv*. Published online November 2020:2020.10.05.20206664. doi:10.1101/2020.10.05.20206664

84. Majiya H, Aliyu-Paiko M, Balogu VT, et al. Seroprevalence of COVID-19 in Niger State. *medRxiv*. Published online August 2020. doi:10.1101/2020.08.04.20168112

85. Fill Malfertheiner S, Brandstetter S, Roth S, et al. Immune response to SARS-CoV-2 in health care workers following a COVID-19 outbreak: A prospective longitudinal study. *Journal of Clinical Virology*. 2020;130:104575. doi:10.1016/j.jcv.2020.104575

86. Martin C, Montesinos I, Dauby N, et al. Dynamics of SARS-CoV-2 RT-PCR positivity and seroprevalence among high-risk healthcare workers and hospital staff. *Journal of Hospital Infection*. 2020;106(1):102-106. doi:10.1016/j.jhin.2020.06.028

87. de Melo MS, Borges LP, de Souza DRV, et al. *Anti-SARS-CoV-2 IgM and IgG Antibodies in Health Workers in Sergipe, Brazil*. Infectious Diseases (except HIV/AIDS); 2020. doi:10.1101/2020.09.24.20200873

88. Morcuende M, Guglielminotti J, Landau R. Anesthesiologists' and Intensive Care Providers' Exposure to COVID-19 Infection in a New York City Academic Center: A Prospective Cohort Study Assessing Symptoms and COVID-19 Antibody Testing. *Anesthesia and analgesia*. 2020;131(3):669-676. doi:10.1213/ANE.0000000000005056

89. Moscola J, Sembajwe G, Jarrett M, et al. Prevalence of SARS-CoV-2 Antibodies in Health Care Personnel in the New York City Area. *JAMA*. 2020;324(9):893-895. doi:10.1001/jama.2020.14765

90. Nishida T, Iwahashi H, Yamauchi K, et al. Seroprevalence of SARS-CoV-2 Antibodies Among 925 Staff Members in an Urban Hospital Accepting COVID-19 Patients in Osaka Prefecture, Japan. *medRxiv*. Published online January 2020:2020.09.10.20191866. doi:10.1101/2020.09.10.20191866

91. Olalla J, Correa AM, Martín-Escalante MD, et al. Search for asymptomatic carriers of SARS-CoV-2 in healthcare workers during the pandemic: A Spanish experience. *QJM: An International Journal of Medicine*. 2020;(hcaa238). doi:10.1093/qjmed/hcaa238

92. Pallett SJC, Rayment M, Patel A, et al. Point-of-care serological assays for delayed SARS-CoV-2 case identification among health-care workers in the UK: A prospective multicentre cohort study. *The Lancet Respiratory Medicine*. 2020;8(9):885-894. doi:10.1016/S2213-2600(20)30315-5

93. Péré H, Wack M, Védie B, et al. Sequential SARS-CoV-2 IgG assays as confirmatory strategy to confirm equivocal results: Hospital-wide antibody screening in 3,569 staff health care workers in Paris. *Journal of Clinical Virology*. 2020;132:104617. doi:10.1016/j.jcv.2020.104617

94. Poulikakos D, Sinha S, Kalra PA. SARS-CoV-2 antibody screening in healthcare workers in a tertiary centre in North West England. *Journal of clinical virology : the official publication of the Pan American Society for Clinical Virology*. 2020;129:104545-104545. doi:10.1016/j.jcv.2020.104545

95. Psichogiou M, Karabinis A, Pavlopoulou I, et al. Antibodies against SARS-CoV-2 among health care workers in a country with low burden of COVID-19. *medRxiv*. Published online June 2020. doi:10.1101/2020.06.23.20137620

For peer review only

96. Kolthur-Seetharam U, Shah D, Shastri J, et al. *SARS-CoV2 Serological Survey in Mumbai by NITI-BMC-TIFR: Preliminary Report of Round-2*. NITI-BMC-TIFR; 2020.

97. Shields AM, Faustini SE, Perez-Toledo M, et al. SARS-CoV-2 seroconversion in health care workers. *medRxiv*. Published online May 2020:2020.05.18.20105197. doi:10.1101/2020.05.18.20105197

98. Ismael Amaral Silva PA, Ismael C, Marchon da Silva C, Domenge C. 1761P Universal screening of SARS-CoV-2 of oncology healthcare workers a Brazilian experience. *Annals of Oncology*. 2020;31:S1024. doi:10.1016/j.annonc.2020.08.1825

99. Solodky ML, Galvez C, Russias B, et al. Lower detection rates of SARS-COV2 antibodies in cancer patients versus health care workers after symptomatic COVID-19. *Annals of Oncology*. 2020;31(8):1087-1088. doi:10.1016/j.annonc.2020.04.475

100. Soriano V, Meiriño R, Corral O, Guallar MP. Severe Acute Respiratory Syndrome Coronavirus 2 Antibodies in Adults in Madrid, Spain. *Clinical Infectious Diseases*. 2020;(ciaa769). doi:10.1093/cid/ciaa769

101. Instituto Nazionale di Statistica. *PRIMI RISULTATI DELL'INDAGINE DI SIEROPREVALENZA SUL SARS-CoV-2*. Instituto Nazionale di Statistica; 2020.

102. Steensels D, Oris E, Coninx L, et al. Hospital-Wide SARS-CoV-2 Antibody Screening in 3056 Staff in a Tertiary Center in Belgium. *JAMA*. 2020;(7501160). doi:10.1001/jama.2020.11160

103. Stock AD, Bader ER, Cezayirli P, et al. COVID-19 Infection Among Healthcare Workers: Serological Findings Supporting Routine Testing. *Frontiers in Medicine*. 2020;7. doi:10.3389/fmed.2020.00471

104. Takita M, Matsumura T, Yamamoto K, et al. Geographical Profiles of COVID-19 Outbreak in Tokyo: An Analysis of the Primary Care ClinicBased Point-of-Care Antibody Testing. *Journal of Primary Care & Community Health*. 2020;11:215013272094269. doi:10.1177/2150132720942695

105. Tong X, Ning M, Huang R, et al. Surveillance of SARS-CoV-2 infection among frontline health care workers in Wuhan during COVID-19 outbreak. *Immunity, Inflammation and Disease*. 2020;8(4):840-843. doi:10.1002/iid3.340

106. Trieu M-C, Bansal A, Madsen A, et al. SARS-CoV-2Specific Neutralizing Antibody Responses in Norwegian Health Care Workers After the First Wave of COVID-19 Pandemic: A Prospective Cohort Study. *The Journal of Infectious Diseases*. 2020;2021-(jiaa737). doi:10.1093/infdis/jiaa737

107. Tu D, Shu J, Wu X, et al. Immunological detection of serum antibodies in pediatric medical workers exposed to varying levels of SARS-CoV-2. *The Journal of Infection*. 2021;82(1):159-198. doi:10.1016/j.jinf.2020.07.023

108. Valdivia A, Torres I, Huntley D, et al. Caveats in interpreting SARS-CoV-2 IgM+/IgG- antibody profile in asymptomatic health care workers. *Journal of Medical Virology*. 2020;n/a(n/a). doi:10.1002/jmv.26400

109. Chafloque-Vasquez RA, Pampa-Espinoza L, Salinas JCC. Seroprevalence of COVID-19 in workers in a hospital in the Peruvian Amazon. *ACTA MEDICA PERUANA*. 2020;37(3). doi:10.35663/amp.2020.373.1050

110. Edna Viegas. *Inquérito Sero-Epidemiológico de SARS-CoV-2 Na Cidade de Maputo (InCOVID 2020)*. República de Moçambique Ministério da Saúde; 2020.

For peer review only

111. Vlachoyiannopoulos P, Alexopoulos H, Apostolidi I, et al. Anti-SARS-CoV-2 antibody detection in healthcare workers of two tertiary hospitals in Athens, Greece. *Clinical Immunology*. 2020;221:108619. doi:10.1016/j.clim.2020.108619

112. Dalla Volta A, Valcamonico F, Pedersini R, et al. The Spread of SARS-CoV-2 Infection Among the Medical Oncology Staff of ASST Spedali Civili of Brescia: Efficacy of Preventive Measures. *Frontiers in Oncology*. 2020;10:1574. doi:10.3389/fonc.2020.01574

113. Ward H, Cooke G, Atchison C, et al. Declining prevalence of antibody positivity to SARS-CoV-2: A community study of 365,000 adults. *medRxiv*. Published online October 2020:2020.10.26.20219725. doi:10.1101/2020.10.26.20219725

114. Xiong S, Guo C, Dittmer U, Zheng X, Wang B. The prevalence of antibodies to SARS-CoV-2 in asymptomatic healthcare workers with intensive exposure to COVID-19. *medRxiv*. Published online June 2020:2020.05.28.20110767. doi:10.1101/2020.05.28.20110767

115. Zhang J, Liu J, Li N, et al. Serological detection of 2019-nCoV respond to the epidemic: A useful complement to nucleic acid testing. *medRxiv*. Published online March 2020:2020.03.04.20030916. doi:10.1101/2020.03.04.20030916

116. Zhao D, Wang M, Wang M, et al. Asymptomatic infection by SARS-CoV-2 in healthcare workers: A study in a large teaching hospital in Wuhan, China. *International Journal of Infectious Diseases*. 2020;99:219-225. doi:10.1016/j.ijid.2020.07.082

117. Ahmad K, Rezvanizadeh V, Dahal S, et al. COVID-19 IgG/IgM antibody testing in Los Angeles County, California. *European Journal of Clinical Microbiology & Infectious Diseases*. Published online November 2020. doi:10.1007/s10096-020-04111-3

118. Halbrook M, Gadoth A, Martin-Blais R, et al. Incidence of SARS-CoV-2 infection among asymptomatic frontline health workers in Los Angeles County, California. *medRxiv*. Published online November 2020:2020.11.18.20234211. doi:10.1101/2020.11.18.20234211

119. Iwuji K, Islam E, Berdine G, Nugent K, Test V, Tijerina A. Prevalence of Coronavirus Antibody Among First Responders in Lubbock, Texas. *Journal of Primary Care & Community Health*. 11:2150132720971390. doi:10.1177/2150132720971390

120. Parker-Magyar A. Few among Long Hill first responders test positive for COVID-19 antibodies. *Echoes Sentinel*. Published online June 2020.

121. Caban-Martinez AJ, Schaefer-Solle N, Santiago K, et al. Epidemiology of SARS-CoV-2 antibodies among firefighters/paramedics of a US fire department: A cross-sectional study. *Occupational and Environmental Medicine*. 2020;77(12):857-861. doi:10.1136/oemed-2020-106676

122. Staletovich J. South Florida Cities Begin Testing Employees For COVID-19 Antibodies. *WLRN*. Published online May 2020.

123. Hibino M, Iwabuchi S, Munakata H. SARS-CoV-2 IgG seroprevalence among medical staff in a general hospital that treated patients with COVID-19 in Japan: Retrospective evaluation of nosocomial infection control. *Journal of Hospital Infection*. 2020;107:103-104. doi:10.1016/j.jhin.2020.10.001

124. Calcagno A, Ghisetti V, Emanuele T, et al. Risk for SARS-CoV-2 Infection in Healthcare Workers, Turin, Italy. *Emerging Infectious Diseases*. 2021;27(1):303-305. doi:10.3201/eid2701.203027

125. Alharbi SA, Almutairi AZ, Jan AA, Alkhalify AM. Enzyme-Linked Immunosorbent Assay for the Detection of Severe Acute Respiratory Syndrome Coronavirus 2 (SARS-CoV-2) IgM/IgA and IgG Antibodies Among Healthcare Workers. *Cureus*. Published online September 2020. doi:10.7759/cureus.10285

For peer review only

126. Chau NVV, Toan LM, Man DNH, et al. Absence of SARS-CoV-2 antibodies in health care workers of a tertiary referral hospital for COVID-19 in southern Vietnam. *Journal of Infection*. 2020;82(1):e36-e37. doi:10.1016/j.jinf.2020.11.018

127. Khan MS, Haq I, Qurieshi MA, et al. SARS-CoV-2 seroprevalence in healthcare workers of dedicated-COVID hospitals and nonCOVID hospitals of District Srinagar, Kashmir. *medRxiv*. Published online October 2020:2020.10.23.20218164. doi:10.1101/2020.10.23.20218164

128. Costa SF, Giavina-Bianchi P, Buss L, et al. SARS-CoV-2 seroprevalence and risk factors among oligo/asymptomatic healthcare workers(HCW): Estimating the impact of community transmission. *Clinical Infectious Diseases*. 2020;(ciaa1845). doi:10.1093/cid/ciaa1845

129. Mohr N, Harland K, Krishnadasan A, Santibanez S, Talan D. Diagnosed and Undiagnosed COVID-19 in US Emergency Department Health Care Personnel: A Cross-sectional Analysis. *Annals of Emergency Medicine*. Published online December 2020. doi:10.1016/j.annemergmed.2020.12.007

130. Noor M, Haq M, Ul Haq N, et al. Does Working in a COVID-19 Receiving Health Facility Influence Seroprevalence to SARS-CoV-2? *Cureus*. Published online November 2020. doi:10.7759/cureus.11389

131. Singhal T, Shah S, Naik R, Kazi A, Thakkar P. Prevalence of COVID-19 Antibodies in Healthcare Workers at the Peak of the Pandemic in Mumbai, India: A Preliminary Study. *Indian Journal of Medical Microbiology*. 2020;38(3):461-463. doi:10.4103/ijmm.IJMM_20_308

132. Dacosta-Urbieta A, Rivero-Calle I, Pardo-Seco J, et al. Seroprevalence of SARS-CoV-2 Among Pediatric Healthcare Workers in Spain. *Frontiers in Pediatrics*. 2020;8. doi:10.3389/fped.2020.00547

133. Sartore-Bianchi A, Patelli G, Tosi F, et al. INCIDENCE OF SARS-COV-2 INFECTION IN PATIENTS WITH ACTIVE CANCER: MONO-INSTITUTIONAL SERIES OF A COMPREHENSIVE CANCER INSTITUTION IN LOMBARDY DURING THE COVID-19 PANDEMIC (NIGUARDA CANCER CENTER, MILANO, ITALY). In: *Tumori Journal*. Vol 106. AIOM Abstracts.; 2020:1-215. doi:10.1177/0300891620953388

134. Brousseau N, Morin L, Ouakki M, et al. *COVID-19 : Étude de séroprévalence chez des travailleurs de la santé de centres hospitaliers au Québec*. Institut National de Sante Publique du Quebec; 2020:20.

135. Chen Y, Tong X, Wang J, et al. High SARS-CoV-2 antibody prevalence among healthcare workers exposed to COVID-19 patients. *The Journal of Infection*. 2020;81(3):420-426. doi:10.1016/j.jinf.2020.05.067

136. Favara DM, Cooke A, Doffinger R, McAdam K, Corrie P, Ainsworth NL. COVID-19 Serology in Oncology Staff Study: Understanding SARS-CoV-2 in the Oncology Workforce. *Clinical Oncology (Royal College of Radiologists (Great Britain)*. 2021;33(1):e61-e63. doi:10.1016/j.clon.2020.07.015

137. Fujita K, Shinpei Kada, Osamu Kanai, et al. Quantitative SARS-CoV-2 antibody screening of healthcare workers in the southern part of Kyoto city during the COVID-19 peri-pandemic period. *medRxiv*. Published online May 2020.

138. Godbout EJ, Pryor R, Harmon M, et al. Severe acute respiratory coronavirus virus 2 (SARS-CoV-2) seroprevalence among healthcare workers in a low prevalence region. *Infection Control & Hospital Epidemiology*. Published online December 2020:1-3. doi:10.1017/ice.2020.1374

139. Houlihan CF, Vora N, Byrne T, et al. Pandemic peak SARS-CoV-2 infection and seroconversion rates in London frontline health-care workers. *The Lancet*. 2020;396(10246):e6-e7. doi:10.1016/S0140-6736(20)31484-7

For peer review only

140. Insúa C, Stedile G, Figueroa V, et al. Seroprevalence of SARS-CoV-2 antibodies among physicians from a children's hospital. *Archivos Argentinos De Pediatria*. 2020;118(6):381-385. doi:10.5546/aap.2020.eng.381

141. Kohler PP, Kahlert CR, Sumer J, et al. Prevalence of SARS-CoV-2 antibodies among Swiss hospital workers: Results of a prospective cohort study. *Infection Control & Hospital Epidemiology*. Published online October 2020:1-5. doi:10.1017/ice.2020.1244

142. Kumar N, Bhartiya S, Singh T. Duration of anti-SARS-CoV-2 antibodies much shorter in India. *Vaccine*. 2021;39(6):886-888. doi:10.1016/j.vaccine.2020.10.094

143. Mesnil M, Joubel K, Yavchitz A, Miklaszewski N, Devys J-M. Seroprevalence of SARS-Cov-2 in 646 professionals at the Rothschild Foundation Hospital (ProSeCoV study). *Anaesthesia Critical Care & Pain Medicine*. 2020;39(5):595-596. doi:10.1016/j.accpm.2020.08.003

144. Missaglia R, Belingheri M, Antolini L, et al. SARS-CoV-2 pandemia in Lombardy: The impact on family Paediatricians. *Italian Journal of Pediatrics*. 2020;46(1):184. doi:10.1186/s13052-020-00950-0

145. Orth-Höller D, Eigentler A, Weseslindtner L, Möst J. Antibody kinetics in primary- and secondary-care physicians with mild to moderate SARS-CoV-2 infection. *Emerging Microbes & Infections*. 2020;9(1):1692-1694. doi:10.1080/22221751.2020.1793690

146. Plebani M, Padoan A, Fedeli U, et al. SARS-CoV-2 serosurvey in health care workers of the Veneto Region. *Clinical Chemistry and Laboratory Medicine (CCLM)*. 2020;58(12):2107-2111. doi:10.1515/cclm-2020-1236

147. Rudberg A-S, Havervall S, Månberg A, et al. SARS-CoV-2 exposure, symptoms and seroprevalence in healthcare workers in Sweden. *Nature Communications*. 2020;11(1):5064. doi:10.1038/s41467-020-18848-0

148. Schmidt SB, Grüter L, Boltzmann M, Rollnik JD. Prevalence of serum IgG antibodies against SARS-CoV-2 among clinic staff. Adrish M, ed. *PLOS ONE*. 2020;15(6):e0235417. doi:10.1371/journal.pone.0235417

149. Sotgiu G, Barassi A, Miozzo M, et al. SARS-CoV-2 specific serological pattern in healthcare workers of an Italian COVID-19 forefront hospital. *BMC Pulmonary Medicine*. 2020;20(1):203. doi:10.1186/s12890-020-01237-0

150. Venugopal U, Jilani N, Rabah S, et al. SARS-CoV-2 seroprevalence among health care workers in a New York City hospital: A cross-sectional analysis during the COVID-19 pandemic. *International Journal of Infectious Diseases*. 2020;102:63-69. doi:10.1016/j.ijid.2020.10.036

151. Racine-Brzostek SE, Yang HS, Chadburn A, et al. COVID-19 Viral and Serology Testing in New York City Health Care Workers. *American Journal of Clinical Pathology*. 2020;154(5):592-595. doi:10.1093/ajcp/aqaa142

152. Hoffmann S, Spallek J, Heinz-Detlef G, Schiebel J, Hufert F. Testing the backbone of the healthcare system: A prospective serological-epidemiological cohort study of healthcare workers in rural Germany. Published online September 2020. doi:10.21203/rs.3.rs-84703/v1

153. Patel MM, Thornburg NJ, Stubblefield WB, et al. Change in Antibodies to SARS-CoV-2 Over 60 Days Among Health Care Personnel in Nashville, Tennessee. *JAMA*. 2020;324(17):1781. doi:10.1001/jama.2020.18796

For peer review only

154. Self WH, Tenforde MW, Stubblefield WB, et al. Seroprevalence of SARS-CoV-2 Among Frontline Health Care Personnel in a Multistate Hospital Network 13 Academic Medical Centers, AprilJune 2020. *MMWR Morbidity and Mortality Weekly Report*. 2020;69(35):1221-1226. doi:10.15585/mmwr.mm6935e2

155. Shah VP, Hainy CM, Swift MD, Breeher LE, Theel ES, Sampathkumar P. Unrecognized severe acute respiratory coronavirus virus 2 (SARS-CoV-2) seroprevalence among healthcare personnel in a low-prevalence area. *Infection Control & Hospital Epidemiology*. Published online November 2020:1-3. doi:10.1017/ice.2020.1341

156. Bampoe S, Lucas DN, Neall G, et al. A cross-sectional study of immune seroconversion to SARS-CoV-2 in front-line maternity health professionals. *medRxiv*. Published online June 2020. doi:10.1101/2020.06.24.20139352

157. Dimcheff DE, Schildhouse RJ, Hausman MS, et al. Seroprevalence of severe acute respiratory syndrome coronavirus-2 (SARS-CoV-2) infection among Veterans Affairs healthcare system employees suggests higher risk of infection when exposed to SARS-CoV-2 outside the work environment. *Infection Control & Hospital Epidemiology*.:1-7. doi:10.1017/ice.2020.1220

158. Finkenzeller T, Faltlhauser A, Dietl K-H, et al. SARS-CoV-2-Antikörper bei Intensiv- und Klinikpersonal. *Medizinische Klinik - Intensivmedizin und Notfallmedizin*. 2020;115(3):139-145. doi:10.1007/s00063-020-00761-5

159. Grant JJ, Wilmore SMS, McCann NS, et al. Seroprevalence of SARS-CoV-2 antibodies in healthcare workers at a London NHS Trust. *Infection Control & Hospital Epidemiology*. Published online August 2020:1-3. doi:10.1017/ice.2020.402

160. Mansour M, Leven E, Muellers K, Stone K, Mendu DR, Wajnberg A. Prevalence of SARS-CoV-2 Antibodies Among Healthcare Workers at a Tertiary Academic Hospital in New York City. *Journal of General Internal Medicine*. 2020;35(8):2485-2486. doi:10.1007/s11606-020-05926-8

161. Martín V, Fernández-Villa T, Lamuedra Gil de Gomez M, et al. Prevalence of SARS-CoV-2 infection in general practitioners and nurses in primary care and nursing homes in the Healthcare Area of León and associated factors. *COVID19 en Atención Primaria*. 2020;46:35-39. doi:10.1016/j.semerg.2020.05.014

162. Meissner EG, Litwin C, Crocker T, Mack E, Card L. 460. Point-of-Care, In-Home SARS-CoV-2 IgG Antibody Testing to Assess Seroprevalence in At-Risk Health Care Workers. *Open Forum Infectious Diseases*. 2020;7(Supplement_1):S297-S297. doi:10.1093/ofid/ofaa439.653

163. Mostafa A, Kandil S, El-Sayed MH, et al. Universal COVID-19 screening of 4040 health care workers in a resource-limited setting: An Egyptian pilot model in a university with 12 public hospitals and medical centers. *International Journal of Epidemiology*. 2020;(dyaa173). doi:10.1093/ije/dyaa173

164. Paradiso AV, Summa simona D, Silvestris N, et al. COVID-19 SCREENING AND MONITORING OF ASYMPTOMATIC HEALTH WORKERS WITH A RAPID SEROLOGICAL TEST. *medRxiv*. Published online May 2020:2020.05.05.20086017. doi:10.1101/2020.05.05.20086017

165. Sydney ER, Kishore P, Laniado I, Rucker LM, Bajaj K, Zinaman MJ. Antibody evidence of SARS-CoV-2 infection in healthcare workers in the Bronx. *Infection Control & Hospital Epidemiology*. 2020;41(11):1348-1349. doi:10.1017/ice.2020.437

166. Zhou F, Li J, Lu M, et al. Tracing asymptomatic SARS-CoV-2 carriers among 3674 hospital staff:A cross-sectional survey. *EClinicalMedicine*. 2020;26. doi:10.1016/j.eclinm.2020.100510

167. Buntinx F, Claes P, Gulikers M, et al. Added value of anti-SARS-CoV-2 antibody testing in a Flemish nursing home during an acute COVID-19 outbreak in April 2020. *Acta Clinica Belgica*. 2020;0(0):1-6. doi:10.1080/17843286.2020.1834285

For peer review only

168. Reuben J, Sherman A, Ellison JA, et al. SARS-CoV-2 Seroprevalence among First Responders in the District of Columbia, May July 2020. *medRxiv*. Published online November 2020:2020.11.25.20225490. doi:10.1101/2020.11.25.20225490

169. Saberian P, Mireskandari SM, Baratloo A, et al. Antibody Rapid Test Results in Emergency Medical Services Personnel during COVID-19 Pandemic; a Cross Sectional study. *Archives of Academic Emergency Medicine*. 2020;9(1).

170. Tarabichi Y, Watts B, Collins T, et al. SARS-CoV-2 Infection among Serially Tested Emergency Medical Services Workers. *Prehospital Emergency Care*. 2020;0(0):1-7. doi:10.1080/10903127.2020.1831668

171. Vijh R, Ghafari C, Hayden A, et al. Serological survey following SARS-COV-2 outbreaks at long-term care facilities in metro Vancouver, British Columbia: Implications for outbreak management and infection control policies. *American Journal of Infection Control*. Published online October 2020. doi:10.1016/j.ajic.2020.10.009

172. Bhattacharya D, Winnett A, Fulcher JA, et al. 70. Lack of SARS-CoV-2 Antibody Seroconversion After Prompt Identification and Cohorting of Sentinel sars-cov-2-positive Residents in a Skilled Nursing Facility. *Open Forum Infectious Diseases*. 2020;7(Supplement_1):S165-S166. doi:10.1093/ofid/ofaa439.380

173. Pérez-García F, Pérez-Zapata A, Arcos N, et al. Severe acute respiratory coronavirus virus 2 (SARS-CoV-2) infection among hospital workers in a severely affected institution in Madrid, Spain: A surveillance cross-sectional study. *Infection Control & Hospital Epidemiology*. Published online October 2020:1-7. doi:10.1017/ice.2020.1303

174. Pérez-García F, Pérez-Zapata A, Arcos N, et al. Severe acute respiratory coronavirus virus 2 (SARS-CoV-2) infection among hospital workers in a severely affected institution in Madrid, Spain: A surveillance cross-sectional study. *Infection Control & Hospital Epidemiology*. 2021;42(7):803-809. doi:10.1017/ice.2020.1303

175. Mughal MS, Kaur IP, Patton CD, Mikhail NH, Vareechon C, Granet KM. The prevalence of severe acute respiratory coronavirus virus 2 (SARS-CoV-2) IgG antibodies in intensive care unit (ICU) healthcare personnel (HCP) and its implicationsa single-center, prospective, pilot study. *Infection Control & Hospital Epidemiology*. Published online June 2020:1-2. doi:10.1017/ice.2020.298

176. Rao S. Covid-19: Jayadeva says its survey hints at herd immunity. *The Times of India*. Published online June 2020.

177. Shukla V, Lau CSM, Towns M, et al. COVID-19 Exposure Among First Responders in Arizona. *Journal of Occupational and Environmental Medicine*. 2020;62(12).

178. Gray A. Prevalence Of COVID-19 Antibodies In Washoe Co. Expected To Be Low. *KUNR*. Published online June 2020.

179. Chughtai O, Batool H, Khan M, Chughtai A. Frequency of COVID-19 IgG Antibodies among Special Police Squad Lahore, Pakistan. *Journal of the College of Physicians and Surgeons Pakistan*. 2020;30(7):735-739. doi:10.29271/jcpsp.2020.07.735

180. Gujski M, Jankowski M, Pinkas J, et al. Prevalence of Current and Past SARS-CoV-2 Infections among Police Employees in Poland, JuneJuly 2020. *Journal of Clinical Medicine*. 2020;9(10):3245. doi:10.3390/jcm9103245

181. Jerónimo Langa. *Inquérito Sero-Epidemiológico de SARS-CoV-2 Na Cidade de Lichinga (InCOVID 2020)*. República de Moçambique Ministério da Saúde; 2020.

For peer review only

182. Ivalda Macicame. *Inquérito Sero-Epidemiológico de SARS-CoV-2 Na Província de Maputo (InCOVID 2020)*. República de Moçambique Ministério da Saúde; 2020.

183. Al-Thani MH, Farag E, Bertollini R, et al. Seroprevalence of SARS-CoV-2 infection in the craft and manual worker population of Qatar. *medRxiv*. Published online November 2020:2020.11.24.20237719. doi:10.1101/2020.11.24.20237719

184. Epstude J, Harsch IA. Seroprevalence of COVID-19 antibodies in the cleaning and oncological staff of a municipal clinic. *GMS Hygiene and Infection Control; 15:Doc18*. Published online July 2020. doi:10.3205/DGKH000353

185. Hassan SS, Seigerud Å, Mühr LSA, et al. SARS-CoV-2 infections among personnel providing home care services for the elderly in Stockholm, Sweden. *medRxiv*. Published online December 2020. doi:10.1101/2020.12.18.20248511

186. Ladhani SN, Jeffery-Smith A, Patel M, et al. High prevalence of SARS-CoV-2 antibodies in care homes affected by COVID-19: Prospective cohort study, England. *EClinicalMedicine*. 2020;28. doi:10.1016/j.eclinm.2020.100597

187. Lindahl JF, Hoffman T, Esmaeilzadeh M, et al. High seroprevalence of SARS-CoV-2 in elderly care employees in Sweden. *Infection Ecology & Epidemiology*. 2020;10(1):1789036. doi:10.1080/20008686.2020.1789036

188. Regan T. Fellowship Village Benefits from Covid-19 Antibody Tests. *Senior Housing News*. Published online June 2020.

189. Alali WQ, Bastaki H, Longenecker JC, et al. Seroprevalence of SARS-CoV-2 in migrant workers in Kuwait. *Journal of Travel Medicine*. 2020;(taaa223). doi:10.1093/jtm/taaa223

190. Addetia A, Crawford KHD, Dingens A, et al. Neutralizing Antibodies Correlate with Protection from SARS-CoV-2 in Humans during a Fishery Vessel Outbreak with a High Attack Rate. McAdam AJ, ed. *Journal of Clinical Microbiology*. 2020;58(11):e02107-20, /jcm/58/11/JCM.02107-20.atom. doi:10.1128/JCM.02107-20

191. Picon RV, Carreno I, da Silva AA, et al. Coronavirus disease 2019 population-based prevalence, risk factors, hospitalization, and fatality rates in southern Brazil. *International Journal of Infectious Diseases*. 2020;100:402-410. doi:10.1016/j.ijid.2020.09.028

192. D B, L B, P T, Pa P, A B, U L. Effectiveness of the measures aimed at containing Sars-cov-2 virus spreading in work settings: A survey in companies based in the Veneto region of Italy. *La Medicina del lavoro*. Published online October 2020. doi:10.23749/mdl.v111i5.10037

193. Xu X, Sun J, Nie S, et al. Seroprevalence of immunoglobulin M and G antibodies against SARS-CoV-2 in China. *Nature Medicine*. 2020;26(8):1193-1195. doi:10.1038/s41591-020-0949-6

194. Chamie G, Marquez C, Crawford E, et al. Community Transmission of Severe Acute Respiratory Syndrome Coronavirus 2 Disproportionately Affects the Latinx Population During Shelter-in-Place in San Francisco. *Clinical Infectious Diseases*. Published online August 2020:ciaa1234. doi:10.1093/cid/ciaa1234

195. McLaughlin C, Doll MK, Morrison KT, et al. High Community SARS-CoV-2 Antibody Seroprevalence in a Ski Resort Community, Blaine County, Idaho, US. Preliminary Results. *medRxiv*. Published online July 2020. doi:10.1101/2020.07.19.20157198

196. Muñoz L, Pífano M, Bolzán A, et al. *Surveillance and Seroprevalence: Evaluation of IgG Antibodies for SARS-Cov2 by ELISA in the Popular Neighborhood Villa Azul, Quilmes, Province of Buenos Aires, Argentina*.; 2020. doi:10.1590/SciELOPreprints.1147

For peer review only
